# Identification of Human Cell Cycle Phase Markers Based on Single-Cell RNA-Seq Data by Using Machine Learning Methods

**DOI:** 10.1155/2022/2516653

**Published:** 2022-08-13

**Authors:** FeiMing Huang, Lei Chen, Wei Guo, Tao Huang, Yu-dong Cai

**Affiliations:** ^1^School of Life Sciences, Shanghai University, Shanghai 200444, China; ^2^College of Information Engineering, Shanghai Maritime University, Shanghai 201306, China; ^3^Key Laboratory of Stem Cell Biology, Shanghai Jiao Tong University School of Medicine (SJTUSM) & Shanghai Institutes for Biological Sciences (SIBS), Chinese Academy of Sciences (CAS), Shanghai 200030, China; ^4^Bio-Med Big Data Center, CAS Key Laboratory of Computational Biology, Shanghai Institute of Nutrition and Health, University of Chinese Academy of Sciences, Chinese Academy of Sciences, Shanghai 200031, China; ^5^CAS Key Laboratory of Tissue Microenvironment and Tumor, Shanghai Institute of Nutrition and Health, University of Chinese Academy of Sciences, Chinese Academy of Sciences, Shanghai 200031, China

## Abstract

The cell cycle is composed of a series of ordered, highly regulated processes through which a cell grows and duplicates its genome and eventually divides into two daughter cells. According to the complex changes in cell structure and biosynthesis, the cell cycle is divided into four phases: gap 1 (G1), DNA synthesis (S), gap 2 (G2), and mitosis (M). Determining which cell cycle phases a cell is in is critical to the research of cancer development and pharmacy for targeting cell cycle. However, current detection methods have the following problems: (1) they are complicated and time consuming to perform, and (2) they cannot detect the cell cycle on a large scale. Rapid developments in single-cell technology have made dissecting cells on a large scale possible with unprecedented resolution. In the present research, we construct efficient classifiers and identify essential gene biomarkers based on single-cell RNA sequencing data through Boruta and three feature ranking algorithms (e.g., mRMR, MCFS, and SHAP by LightGBM) by utilizing four advanced classification algorithms. Meanwhile, we mine a series of classification rules that can distinguish different cell cycle phases. Collectively, we have provided a novel method for determining the cell cycle and identified new potential cell cycle-related genes, thereby contributing to the understanding of the processes that regulate the cell cycle.

## 1. Introduction

Cell proliferation is one of the basic biological activities in the growth and development of living organisms. The sequence of events that occur during the cell proliferation process is generally termed as the cell cycle, which is usually divided into four phases, namely, gap 1 (G1), DNA synthesis (S), gap 2 (G2), and mitosis (M) [1]. In eukaryotes, cells in different cell cycle phases have specific gene expression patterns that help them perform functions that are specific to that phase [2]. Several researchers have reported that the dysregulation of such cell cycle-specific gene expressions leads to the deregulation of the cell cycle, thereby causing abnormal cell proliferation and the production of cancer-associated cells [3, 4]. In addition, drugs that target the cell cycle to treat cancer tend to target cells that are in specific cell cycle phases [5]. The identification of the cell cycle phases and their associated genes has not only made a significant contribution to the study of cancer development but has also had an indelible role in the development of targeted cell cycle therapeutics.

Cell cycle phase transitions are tightly coordinated, thereby making it difficult to efficiently isolate cells from one phase to the next. From the past to the present, researchers have mainly detected cells at different phases by using the following methods. Initially, Potten et al. used the percentage-labeled mitosis method to determine the cell cycle by counting the percentage of labeled mitotic cells [6]. Subsequently, researchers combined PI staining and flow cytometry to distinguish the cells of different phases by using the characteristics of different DNA and RNA contents in each phase [7]. Nowadays, the most commonly used FUCCI technique focuses on differentiating the cell cycle by identifying two overexpressed cell cycle-regulating proteins, geminin and Cdt1, which are combined with fluorescent motifs to make cells appear different colors at various times [8]. However, all these methods have the following disadvantages: time consuming and difficult to identify cells on a large scale.

Cell cycle regulatory genes are expressed periodically during the cell cycle phase, and the identification and investigation of these genes can provide insight into the cell cycle process. In the past, cell cycle regulatory genes have been identified by microarray technology that identifies differentially expressed genes in synchronized cells that are in different cell cycle phases [9]. However, even with the advanced synchronization techniques, synchronization loss can occur within a few cycles [10]. This loss of synchronization and the noise of the microarray analysis can eventually lead to biased results. In recent years, with the use of machine learning methods on biological data, several computational methods for mining cell cycle genes have emerged. Liu et al. used a convolutional neural network to dissect the expression pattern of cell cycle genes in yeast [11]. Cui et al. constructed a deep learning computational framework for identifying periodically expressed genes [12]. However, the identification of cell cycle phase marker genes in humans with high resolution is still a challenge.

Rapid developments in single-cell technology have made it possible to dissect cells on a large scale with unprecedented resolution. Sequencing for individual cells facilitates our study of gene expression within cells. Meanwhile, how to assign cells to various stages of the cell cycle based on their transcriptional profiles has become a research hotspot [13]. In the Seurat and Scran packages, discriminating the stage of the cell cycle is mainly performed by using a priori cell cycle-specific expression gene set to score individual cells for each phase [14, 15]. Grun et al. identified oscillatory genes and used them to determine the cell cycle phase of a single cell [16]. These methods use known gene sets to make determinations and are unable to mine new cell cycle-related genes. Cyclum and cyclops, as two unsupervised learning methods, identify cell cycle phases in the scRNA-seq data by projecting high-dimensional single cells onto a low-dimensional cyclic periodic trajectory [17, 18]. However, the process of dimensionality reduction often leads to information loss. Therefore, we need to continuously explore new sets of cell cycle-related genes by employing efficient and accurate classification methods based on the significance of cell cycle studies.

In this study, we identify a number of cell cycle-related genes and classification rules using supervised machine learning approaches based on a publicly released single-cell dataset. First, we filter the gene features using Boruta filtering method and then rank the filtered features using three effective feature selection methods (e.g., max-relevance and min-redundancy (mRMR) [19], Monte Carlo feature selection (MCFS) [20], and SHAP by LightGBM [21]). The sequenced feature lists are then used as input to four efficient classification algorithms using incremental feature selection (IFS) methods [22]. After analyzing the performance of classification algorithms and optimal features, we obtain some genes that have been reported and confirmed to be associated with the cell cycle, demonstrating the accuracy of our analysis. Also, some genes that have not been explicitly reported to be associated with the cell cycle are discovered, thereby demonstrating the potential of our analysis in mining new cell cycle-associated genes. In addition, we establish rules for distinguishing different cell cycle phases. Overall, our study provides a novel computational method to predict the cell cycle phase at the single-cell level and identifies several cell cycle-related genes that have not been reported yet. The top-ranked features and decision rules identified by our analysis contribute to a better understanding about the underlying mechanism in cell cycle regulation.

## 2. Materials and Methods

### 2.1. Data

The single-cell RNA sequencing data of human U2OS cells at different cell cycles are downloaded from the GEO database under the accession number GSE146773 (https://www.ncbi.nlm.nih.gov/geo/query/acc.cgi?acc=GSE146773) [23]. This data includes cells from three different cell cycle phases with the following numbers: 346 G1 phase, 387 G2/M phase, and 334 S phase. U2OS FUCCI cells in either G2 or M phase exhibit green signals, so we refer to the cells from these two periods collectively as G2/M. For each cell, the expression levels of 42,728 genes are quantified using transcript per million (TPM) method.

### 2.2. Boruta Feature Filtering

Among all features, most of them are irrelevant to the classification. If all features are selected for subsequent analysis, it will introduce redundancy and noise, which can lead to biased calculations. Here, we apply the Boruta [24] method to filter out irrelevant features.

Boruta is a fully correlated feature selection method based on random forest (RF). That is, it attempts to find all features that carry significant information used for prediction rather than finding some compact subset of features that have the least possible classification error. The Boruta algorithm consists of the following steps: (1) a shadow variable is created for each explanatory variable, thereby generating the expanded data. (2) RF model is applied to this expanded data for evaluating the importance of each variable. (3) A z-score of accuracy loss is calculated for each variable, including the original and shadow variables. (4) Track the original attribute z-scores that are significantly higher than the maximum of the shadow attribute z-score. The above steps were repeated until all features are confirmed or rejected. Original attributes with a statistically significant higher z-score than the shadow attribute's maximum z-score are considered relevant for prediction.

In this study, we opt for the Boruta program from https://github.com/scikit-learn-contrib/boruta_py and select the default parameters for the subsequent analysis.

### 2.3. Feature Ranking Algorithms

Here, we use three feature ranking algorithms (mRMR, MCFS, and SHAP values by LightGBM) to rank the features filtered by Boruta. The details of three methods are described as follows.

#### 2.3.1. Max-Relevance and Min-Redundancy

mRMR is a filtered feature selection algorithm that maximizes the correlation between features and targets and reduces redundancy between selected features [19]. The algorithm treats each feature and output category as separate variables and uses mutual information to measure the similarity between the two variables, with the following expression:

(1) $$\begin{array}{c} MI\left(x,y\right)=\iint p\left(x,y\right)\log\frac{p\left(x,y\right)}{p\left(x\right)p\left(y\right)}dxdy, \end{array}$$

where *p*(*x*, *y*) represents the joint probabilistic density of *x* and *y*. *p*(*x*) and *p*(*y*) represent the marginal probabilistic densities of *x* and *y*, respectively. In the mRMR process, the correlation between the feature set and the target needs to be calculated each time when a feature is added. Although the correlation between features and targets is high, the interfeature dependence could also be very high. Thus, achieving minimum redundancy is necessary. The formulas for maximum correlation and minimum redundancy are expressed as follows:

(2) $$\begin{array}{c} \max D\left(S,c\right),D=\frac{1}{\left|S\right|}\underset{f_{i}\in S}{\sum }MI\left(f_{i},c\right),\\ \min R\left(S\right),R=\frac{1}{\left|S\right|}\underset{f_{i},f_{j}\in S}{\sum }MI\left(f_{i},f_{j}\right), \end{array}$$

where *S* is the feature subset, |*S*| is the number of features, *f*_*i*_ is the *i*-th feature, and *c* is the target variable. Finally, the maximum correlation *D* is combined with the minimum redundancy *R* to make an optimization equation. Features are selected by maximizing the equation.

However, such feature subset is difficult to find out. mRMR adopted a feasible scheme to sort all investigated features. Features are selected one by one using the criteria of maximum correlation and minimum redundancy; that is, one feature is selected in each round, which has maximum correlation to target variable and minimum redundancy to already-selected features. After all features have been selected, a feature list is generated according to the selection order of features. In the present study, we use the mRMR program from http://home.penglab.com/proj/mRMR/ and perform the analysis according to the default parameters.

#### 2.3.2. Monte Carlo Feature Selection

The MCFS algorithm is a method for importance assessment of features based on decision trees (DTs) [20]. The algorithm is originally designed to address the problem of small sample size and large number of features in biological data. Therefore, it is widely used on sequencing features. Here, we select this approach to facilitate the data mining of significant cell cycle genes.

The first step of MCFS is to randomly generate *s* feature subsets from the initial *d* features, each of which contains different *m* features. The samples that correspond to each subset are split into a training set and a test set in the ratio of 2 : 1, respectively. This division is performed *t* times, and each dataset is used to train a DT. Finally, a total of *t* × *s* DTs are constructed, and their performance is evaluated in the test set. A score called relative importance (RI) is assigned to feature *g* based on how well it performs in these classifiers and is given by the following formula:

(3) $$\begin{array}{c} {RI}_{g}=\overset{st}{\underset{\tau =1}{\sum }}{\left(wAcc\right)}^{u}\underset{n_{g}\left(\tau \right)}{\sum }IG\left(n_{g}\left(\tau \right)\right){\left(\frac{\text{no}.\text{ in }n_{g}\left(\tau \right)}{\text{no}.\text{ in }\tau }\right)}^{v}, \end{array}$$

where *wAcc* represents the weighted accuracy, *IG*(*n*_*g*_(*τ*)) is the information gain (IG) of *n*_*g*_(*τ*) (a DT node *n* with the attribute *g*), (no. in *n*_*g*_(*τ*)) is the number of samples in *n*_*g*_(*τ*), and (no.in *τ*) is the number of samples in the tree root. *u* and *v* represent two fixed positive integers. All features are ranked in all features in the decreasing order of their RI values.

In this research, we utilize the MCFS program, which are obtained from http://www.ipipan.eu/staff/m.draminski/mcfs.html and run such program with the default parameters.

#### 2.3.3. SHAP Values by LightGBM

SHAP (Shapley Additive Explanations), which is first proposed in 2017 by Lundberg and Lee, attempts to calculate the Shapley value of each feature of an instance, which estimates the contribution of the feature to the prediction of the instance [25, 26]. Given an instance *x*, its SHAP model is defined by the following equation:

(4) $$\begin{array}{c} \text{g}\left(z^{\prime }\right)=\emptyset_{0}+\overset{k}{\underset{j=1}{\sum }}\emptyset_{j}z_{j}^{\prime }, \end{array}$$

where *z*′ ∈ {0, 1}^*k*^  is the coalition vector where 1 or 0 denotes that a feature is present or absent; *k* is the total number of features; g(*z*′) is the explanation model, which approximates the original model *f*(*z*) when *z* = *h*_*z*_(*z*′), where *h*_*z*_ : {0, 1}^*k*^⟶ℝ^*p*^ maps *z*′ to a valid instance *z*, ∅_0_ represents the expected value of the original model *f*, ∅_*j*_ is the SHA*P* value of the *j*-*th* feature, and *z*_*j*_′ ∈ {0, 1} denotes whether the *j*-th feature is present or not. Notably, *x* = *z* = *h*_*z*_(*z*′) when each element in *z*′ equals to 1. The SHAP values of *x* can be calculated by fitting a weighted linear model given a series of *z*′ and g(*z*′), and the coefficients of the linear model are taken as the SHAP values. Suppose that the SHAP value of *j*-th feature of *i*-th sample is ∅_*j*_(*x*_*i*_); if ∅_*j*_(*x*_*i*_) > 0, then the feature increases the prediction value; conversely, it means that the feature decreases the prediction value. The absolute value |∅_*j*_(*x*_*i*_)| indicates the contribution of the *j*-th feature towards the prediction of *x*_*i*_. Thus, the importance of the *j*-th feature in predicting all the instances can be calculated by the following equation:

(5) $$\begin{array}{c} I_{j}=\frac{1}{n}\overset{n}{\underset{i=1}{\sum }}\left|\emptyset_{j}\left(x_{i}\right)\right|, \end{array}$$

where *n* is the total number of the instances. The features in this research are ranked according to *I*_*j*_. SHAP can be realized through any prediction model. LightGBM is a gradient boosting model that uses DTs as the base learner [21], which is rather effective and efficient in training and testing. Thus, LightGBM is adopted as the prediction model to calculate the SHAP values.

This study adopts the program of LightGBM obtained from https://lightgbm.readthedocs.io/en/latest/. Such program is executed using its default parameters.

### 2.4. Incremental Feature Selection

IFS is a strategy used to search for the optimal number of features [22]. It is widely used for feature selection because of its efficiency and simplicity. Previously, we have obtained some feature lists by using feature ranking algorithms, but the optimal subsets cannot be determined yet. We combine the results of the feature ranking algorithms with the IFS to obtain the optimal feature subsets for subsequent analysis. Given a feature list, several feature subsets are constructed, each of which contains some top features in the list. Then, a classifier is built on samples represented by features in each subset. All classifiers are further evaluated by tenfold cross-validation [27]. The classifier with best performance, measured by Matthew's correlation coefficient (MCC) [28–31] in this study, is picked up. Such classifier is called the optimal classifier and the feature subset used in this classifier is termed as the optimal feature set for this classifier.

### 2.5. Classification Algorithm

IFS requires supervised classification algorithms as a basis, so we select four efficient classification algorithms: RF [32], support vector machine (SVM) [33], k-nearest neighbor (KNN) [34], and DT [35]. These algorithms have wide applications [36–43]. They are briefly described as follows.

#### 2.5.1. Random Forest

RF is an ensemble classification algorithm, which consists of several DTs. Each DT is built by randomly selecting features and samples. To make a classification, the voting method is used, where the final output is obtained by choosing the category with the highest votes. Here, we use RF from the Scikit-learn [44] package in Python. Default parameters are used to execute this package, where the number of DTs is 100.

#### 2.5.2. Support Vector Machine

SVM is a classic machine learning algorithm with excellent performance and is of high popularity. It is often used to solve classification problems. The SVM process can be roughly divided into three parts: maximum interval, high-dimensional mapping, and kernel methods. The maximum interval refers to the distance that maximizes the data to the decision boundary, which is the core idea of SVM. High-dimensional mappings are the key to SVM that can be used in linear methods to solve nonlinear classification problems. The introduction of kernel methods allows SVMs to handle problems in a more diverse way to achieve higher efficiency. In this study, we use the SVM in the Scikit-learn [44] package. RBF function is set as the kernel of SVM.

#### 2.5.3. k-Nearest Neighbor

The core idea of the KNN algorithm is that if the majority of the *k* most similar samples in the feature space of a sample belong to a certain class, then that sample also belongs to that class. This similarity is measured by calculating the distance between samples. Therefore, the calculation of the distance and the choice of *k* values are the focus of this algorithm. We do this by using the KNN in the Scikit-learn [44] package and setting the default parameters.

#### 2.5.4. Decision Tree

As a machine learning algorithm with good interpretation, high training efficiency, and easy understanding, DTs are widely used in areas, such as classification and feature selection [35]. The DT recursively performs splitting and produces a tree structure that consists of nodes and directed edges. Moreover, the classification of an instance is made by sorting down the tree until reaching a leaf node. We use DT to generate a set of interpretable rules for subsequent analysis. Here, we use the Scikit-learn [44] package to perform DT using the CART algorithm with Gini coefficients as the information gain.

### 2.6. Performance Evaluation

The MCC is a relatively balanced indicator and can be used for cases where the sample size is unbalanced. The range of MCC is [−1, 1], where a value of 1 means that the predictions are exactly the same as the actual results, a value of 0 means that the predictions are like random predictions, and −1 means that the predictions are the opposite of the actual results. The MCC describes the strength of the correlation between the predicted and actual results. For multiclassification problems, the MCC can be calculated by the following formula [28]:

(6) $$\begin{array}{c} MCC=\frac{\mathrm{cov}\left(X,Y\right)}{\sqrt{\mathrm{cov}\left(X,X\right)\mathrm{cov}\left(Y,Y\right)}}=\frac{1/K\sum_{n=1}^{N}\sum_{k=1}^{K}\left(X_{nk}-{\bar{X}}_{k}\right)\left(Y_{nk}-{\bar{Y}}_{k}\right)}{\sqrt{\sum_{n=1}^{N}\sum_{k=1}^{K}{\left(X_{nk}-{\bar{X}}_{k}\right)}^{2}\sum_{n=1}^{N}\sum_{k=1}^{K}{\left(Y_{nk}-{\bar{Y}}_{k}\right)}^{2}}}, \end{array}$$

where *X* represents the binary matrix (*N* × *K*, where *N* and *K* stand for the numbers of samples and classes, respectively) into which the predicted class of each sample is converted by one-hot encoding, *Y*  represents another binary matrix (*N* × *K*) into which the true class of each sample is converted by one-hot encoding, and cov(*X*, *Y*) represents the covariance of two matrices. ${\bar{X}}_{k}$ and ${\bar{Y}}_{k}$ represent the means of the *k* column of matrices *X* and *Y*, respectively. *X*_*nk*_ and *Y*_*nk*_  are the elements in the *n*-th row and *k*-th column of the matrices *X* and *Y*, respectively.

In addition, the accuracy on each class (different cell cycle phases in this study) and overall accuracy (ACC) are also computed to give a full evaluation on different classifiers. The accuracy on one class is the ratio of correctly classified samples and all samples in this class, where ACC is the ratio of correctly classified samples and all samples.

### 2.7. Functional Enrichment Analysis

Through the IFS method, the optimal feature subsets for a classification algorithm can be obtained. Functional enrichment analysis is important as a means of unraveling the molecular mechanisms of biomedicine and is important for revealing other processes influenced by cell cycle-related genes. Here, we use R package clusterProfiler [45] to perform Gene Ontology (GO) and Kyoto Encyclopedia of Genes and Genomes (KEGG) pathway enrichment analyses.

## 3. Results

**Figure 1:**
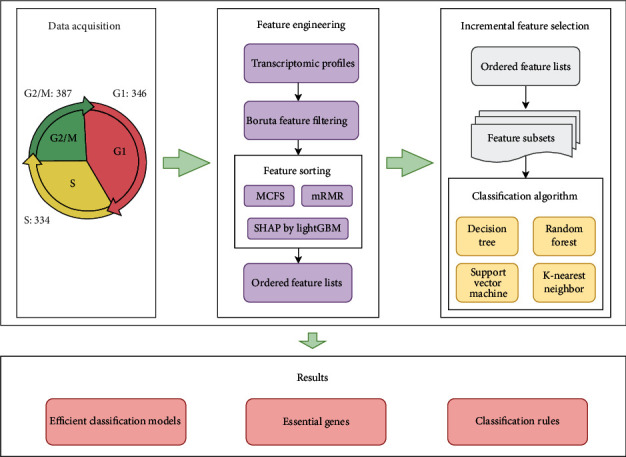
Flow chart of the whole analysis process of this study. Single-cell RNA sequencing data acquired through the GEO database includes cells from three different cell cycle phases with the following numbers: 346 G1 phase, 387 G2/M phase, and 334 S phase. Subsequently, sorted feature list from the single-cell atlas is generated through feature selection methods. Each list is partitioned into feature subsets which are fed into the four classification algorithms to retrieve the efficient genes, build effective classifiers, and construct classification rules.

In this study, we mine cell cycle-related genes and rules for cell cycle classification through some feature selection methods and classification algorithms. The overall computational procedures are illustrated in Figure 1. The results of each step are described in detail below.

### 3.1. Results of Feature Selection Methods

First, Boruta is applied on single-cell RNA sequencing of human U2OS cells for feature filtering. A total of 788 gene features are retained and are displayed in Table S1. Then, these genes are analyzed by three feature ranking algorithms (MCFS, mRMR and SHAP by LightGBM) to generate feature lists according to their importance. These feature lists are also provided in Table S1.

### 3.2. Results of IFS Method

The three ordered feature lists produced by three feature ranking algorithms are fed into the IFS method with four classification algorithms (e.g., RF, SVM, KNN, and DT) one by one. From each feature list, all possible feature subsets are constructed with step interval of 1. A classifier is built on each feature subset with a given classification algorithm. All classifiers are assessed by tenfold cross-validation. The predicted results are provided in Table S2.

**Figure 2:**
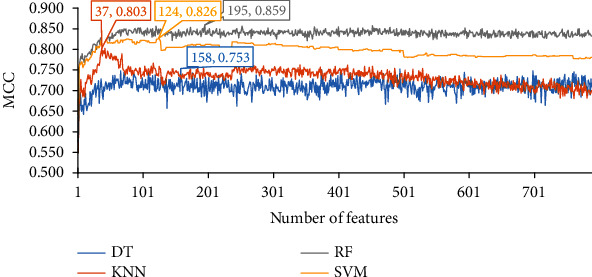
IFS curves of four classification algorithms on the feature list yielded by the mRMR method. The highest MCC values for four classification algorithms are 0.753, 0.803, 0.859, and 0.826, respectively. Such performance is obtained by using top 158, 37, 195, and 124 features in the list.

**Figure 3:**
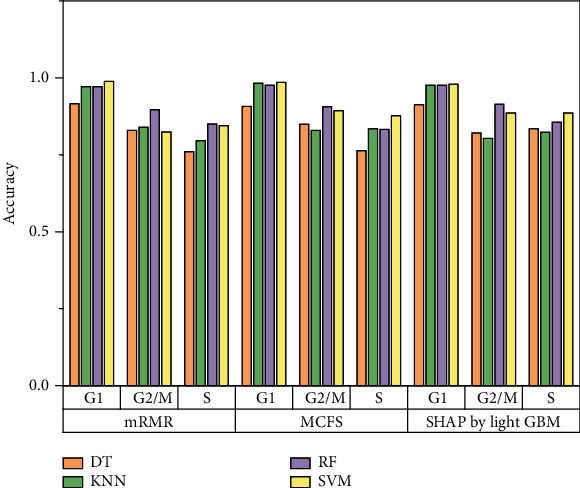
Performance of the optimal classifiers on three different phases. The optimal classifiers based on random forest (RF) and support vector machine (SVM) have better classification performance than the optimal classifiers based on decision tree (DT) and k-nearest neighbor (KNN).

Of the feature list yielded by the mRMR method, the performance of four classification algorithms on all feature subsets is illustrated in four IFS curves, as shown in Figure 2, from which we can see that the highest MCC values for DT, KNN, RF, and SVM are 0.753, 0.803, 0.859, and 0.826, respectively. These MCC values are obtained by using top 158, 37, 195, and 124 features, respectively, in the list. Thus, these features comprise the optimal feature subset for four classification algorithms, and the optimal DT, KNN, RF, and SVM classifiers are set up with their corresponding optimal feature subset. The ACC values of these classifiers are 0.836, 0.869, 0.906, and 0.884, respectively, as listed in Table 1. Their performance on three classes is shown in Figure 3. Evidently, among all four optimal classifiers, the optimal RF classifier gives the best performance and the optimal DT classifier yields the lowest performance.

**Figure 4:**
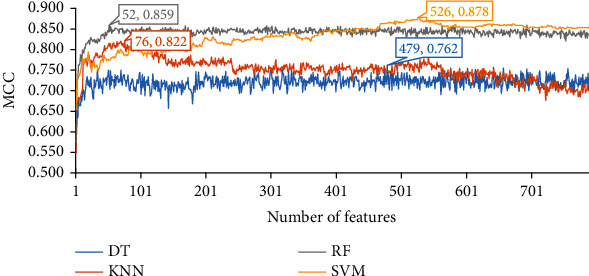
IFS curves of four classification algorithms on the feature list yielded by the MCFS method. The highest MCC values for four classification algorithms are 0.762, 0.822, 0.859, and 0.878, respectively. Such performance is obtained by using top 479, 76, 52, and 526 features in the list.

As for the feature list produced by the MCFS method, we also plot an IFS curve for each classification algorithm, as illustrated in Figure 4. When top 479, 76, 52, and 526 features in the list are adopted, the DT, KNN, RF, and SVM provide the highest MCC values of 0.762, 0.822, 0.859, and 0.878. Accordingly, the optimal feature subset for each of four classification algorithms is determined, which consists of top 479, 76, 52, and 526 features, respectively, in the list. Furthermore, the optimal DT, KNN, RF, and SVM classifiers are built on their optimal feature subsets, respectively. The ACC values of these classifiers are listed in Table 1, and their performance on three classes is listed in Figure 3. The optimal SVM classifier produces the best performance, and the performance of the optimal DT classifier is much lower than other three classifiers.

**Figure 5:**
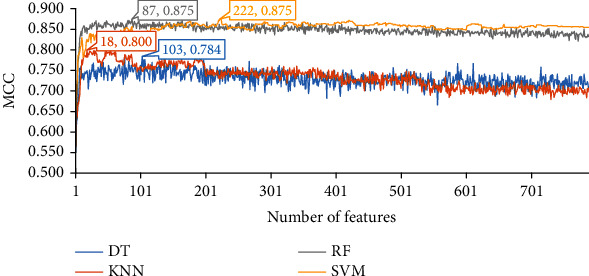
IFS curves of four classification algorithms on the feature list yielded by the SHAP by LightGBM. The highest MCC values for four classification algorithms are 0.784, 0.800, 0.875, and 0.875, respectively. Such performance is obtained by using top 103, 18, 87, and 222 features in the list.

For the last feature list generated by SHAP by LightGBM, the performance of four classification algorithms on different feature subsets is also summarized in four IFS curves, as shown in Figure 5. Four classification algorithms give the best performance with MCC values of 0.784, 0.800, 0.875, and 0.875, respectively, when top 103, 18, 87, and 222, respectively, features in the list are used. These features constitute the optimal feature subset for each classification algorithm, with which the optimal DT/KNN/RF/SVM classifier is built. Their detailed performance is shown in Table 1 and Figure 3. The optimal RF and SVM give equal performance and they are much superior to other two optimal classifiers. The optimal DT classifier again provides the lowest performance.

**Figure 6:**
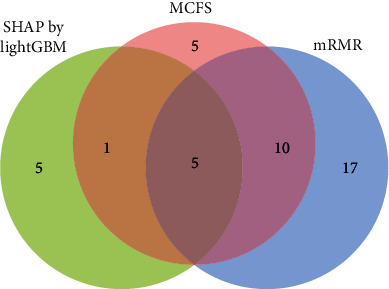
Venn diagram to show the intersection of three feasible feature subsets for three feature ranking algorithms. Five features are contained in all three feature subsets.

Among all optimal classifiers based on different classification algorithms and feature lists, the optimal SVM classifier obtained by using feature list generated by MCFS provided the highest MCC of 0.878. Such classifier can be a latent useful tool to identify cell cycle phases. On the other hand, according to Figures 2, 4, and 5 and Table 1, the optimal feature subsets always contain lots of features. They are almost useless for us to extract most essential features. In view of this, we try to find out one feasible feature subset for each feature list, on which one classifier can still provide satisfied performance. By observing IFS curves in Figures 2, 4, and 5, we can find that the IFS curve of RF always follows a sharp increasing trend at the beginning of the curve and the RF always provides better performance than other three classification algorithms at this stage. Thus, we determine the feasible feature subset according to the IFS curve of RF. By carefully checking the IFS curves of RF, we determine that the top 32 features in the list yielded by the mRMR method constitute the feasible feature subset for such list. On such subset, RF provides the MCC of 0.821, which is a little lower than that of the optimal RF/SVM classifier. As for the feature list yielded by MCFS method, top 21 features comprise the feasible feature subset, whereas the top 11 features constitute the feasible feature subset for the list yielded by SHAP by LightGBM. The RF classifiers on these feature subsets provide the MCC values of 0.823 and 0.851, respectively, which are also a little lower than those of the optimal RF/SVM classifiers. These feasible feature subsets can be seen in Table S3. As shown in Figure 6, a Venn diagram is plotted for these three feasible feature subsets. It can be observed that five features are included in all three subsets. Their corresponding genes are listed in Table 2. A detailed analysis has been conducted for these overlapping genes, which can be seen in Section 4.

### 3.3. Classification Rules

DT, as a white-box algorithm, has good interpretability, although it is generally not as good as other black-box algorithms in terms of overall model performance. The results in Section 3.2 further confirm this fact; that is, the optimal DT classifiers are always inferior to other optimal classifiers. However, it can provide more clues to uncover hidden information in the single-cell RNA sequencing data. In detail, owing to DT's unique single tree structure, we can extract the classification rules to obtain a quantitative representation of the features used for cell cycle classification.

**Figure 7:**
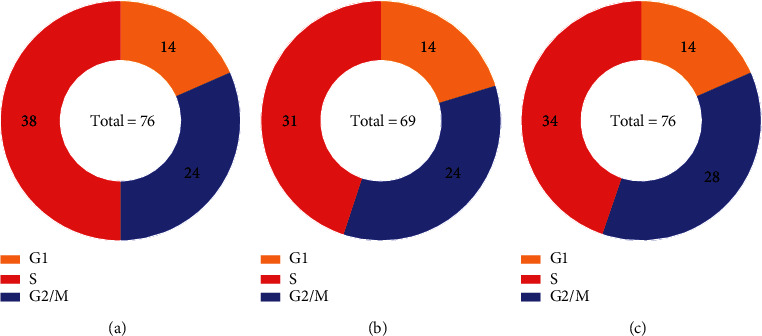
The ring diagram to show the distribution of classification rules over three cell cycle phases, which are extracted from the DTs for the feature lists obtained by three feature ranking algorithms. (a) Rules on mRMR method. (b) Rules on MCFS method. (d) Rules on SHAP by LightGBM.

Here, in IFS results on the feature lists generated by the mRMR, MCFS, and SHAP by LightGBM methods, the optimal DT classifiers use the top 158, 479, and 103 features, respectively. Therefore, we construct the DTs with these features and extract rules form these trees. 76, 69, and 76 classification rules are obtained, respectively, which can be seen in Table S4. The number of rules that distinguish each cell cycle phase is shown in Figure 7. Limits on the amount of gene expression are set in each rule, thereby revealing the importance of high or low gene expression in differentiating the cell cycle phase. The classification rules generated by three feature ranking methods are similar to some extent. A detailed discussion of these rules can be found in Section 4.

### 3.4. Functional Enrichment Analyses

**Figure 8:**
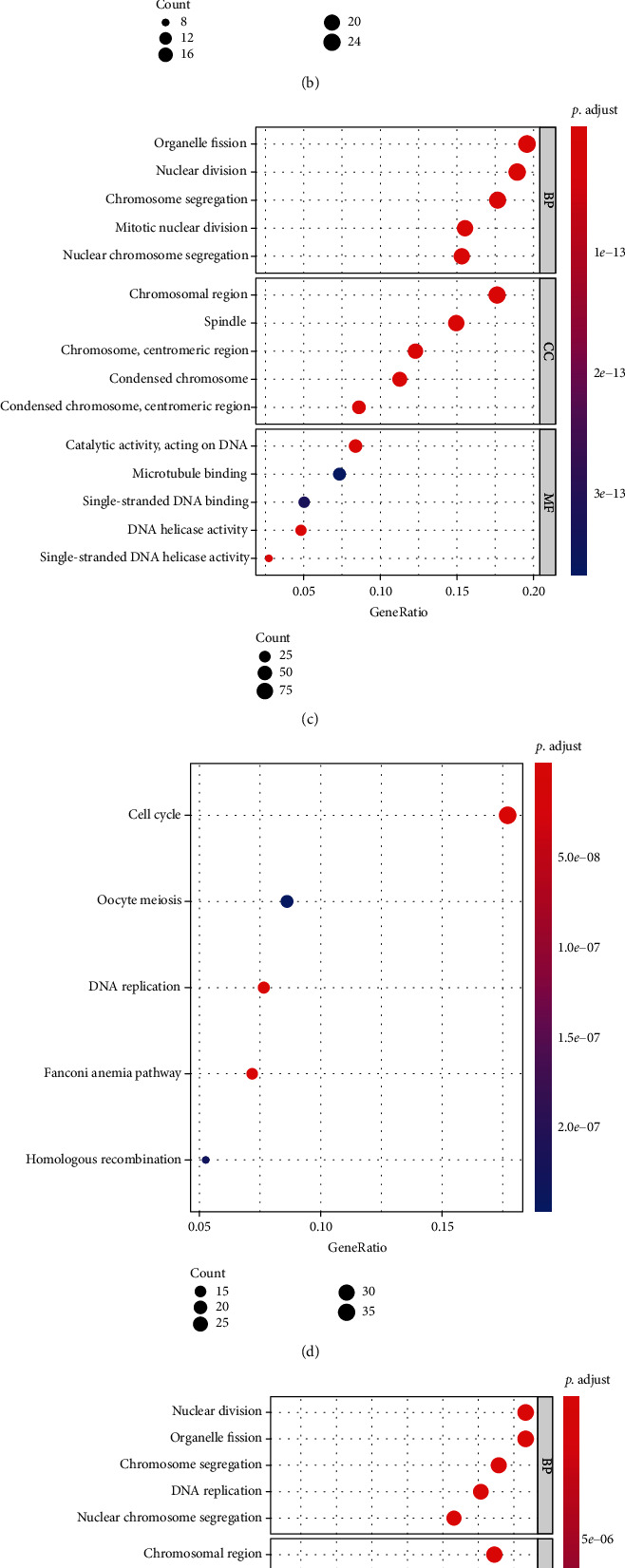
Gene Ontology enrichment and KEGG pathway enrichment analysis on the optimal genes based on three feature ranking algorithms. The FDR < 0.05 criterion was used to filter GO terms and KEGG pathways. (a) The top 5 GO enrichment results based on mRMR. (b) The top 5 KEGG pathway enrichment results based on mRMR. (c) The top 5 GO enrichment results based on MCFS. (d) The top 5 KEGG pathway enrichment results based on MCFS. (e) The top 5 GO enrichment results based on SHAP by LightGBM. (f) The top 5 KEGG pathway enrichment results based on SHAP by LightGBM.

For each feature list yielded by one feature ranking algorithm, the optimal feature subset that can provide the best performance for four classification algorithms is selected. According to Table 1, top 195, 526, and 222 features in the feature lists yielded by mRMR, MCFS, and SHAP by LightGBM are picked up. The clusterProfiler package is used to perform GO and KEGG enrichment analyses on above three optimal feature subsets. GO terms and KEGG pathway are filtered according to the condition of FDR < 0.05. Subsequently, the top 5 of each GO term and KEGG pathway are selected for visualization, which can be seen in Figure 8. Among these enrichment results, results like DNA replication activity and the p53 signaling pathway have been reported to be associated with the cell cycle. A detailed analysis of the enrichment results can be seen in Section 4.

## 4. Discussion

We use several advanced computational methods to identify the genes and rules for distinguishing cells from different cell cycle phases at the single-cell level. In the above, we identified the feasible feature set from each feature list, which make important contributions in classifiers and are considered potential cell cycle phase markers. These feasible feature sets consist of top 32, 21, and 11, respectively, features in the corresponding list. Then, we used the cell cycle-related gene sets in Seurat and Scran to screen all genes in the feasible feature sets that nearly 70% of the genes could be found in these datasets [14, 15]. This illustrates the accuracy of our results and implies a portion of potential cell cycle-related gene markers. Detailed analyses of the correlations between cell cycle and features that overlap in the three feature subsets or rules are provided below.

### 4.1. Features Found by All Three Feature Ranking Algorithms

A total of five genes are present in all three feature subsets, as listed in Table 2. These five genes are found to be qualitatively better for classifier performance in different feature filtering approaches. Therefore, these five genes are considered to be important for the classification of cell cycle phases. These genes have been reported in other studies as follows.

The first gene in the predicted list is *CDK1* (ENSG00000170312), which encodes a cyclin-dependent kinase. CDK1 kinase coordinates the processes of mitosis, DNA replication, and transcription by binding to other cell cycle proteins [46]. The *CDK1* plays a crucial role in regulating the cellular transition from G1 to S and from G2 to M [47]. The abnormal regulation of the cell cycle often leads to the development of cancer. As early as 2007, it was discovered that sustained activation of *CDK1* causes cells to fail to exit mitosis normally, resulting in the appearance of abnormal cells that have been shown to be associated with the development of cancer [48]. In 2018, tumor cells with radioactive inhibition of CDK1 activity were found to have a weaker clone level, further demonstrating the potential of *CDK1* in tumor therapy [49]. In addition, CDK1 drives phosphorylation of phosphorylation sites near protein nuclear localization signals, thereby assisting nuclear pore transport and completing nuclear proteome remodeling in the late M phase [50]. *CDK1* not only broadens the approach to tumor therapy based on control of the cell cycle but also plays an important role in cell cycle development.

Apart from *CDK1*, *FAM111B* (ENSG00000189057) is also found in our prediction list, and it encodes a homologous serine protease. Notably, FAM111A appears as a homologous protein to FAM111B in both feature subsets. Previous studies have shown that protein obstacles exist on genomic DNA, and such protein obstacles will affect the replication fork advancement, causing DNA replication to stall [51]. *FAM111A* mitigates the effects of protein obstacles on replication forks, thereby ensuring proper DNA replication [51]. In addition, mutants of *FAM111A* and *FAM111B* exhibit the inhibition of chromosome-associated processes, which leads to the shutdown of DNA replication and transcription, impaired microtubule network integrity, and even cell death [52]. Moreover, mutations in *FAM111B* cause hereditary fibrous dermatosis combined with tendon contracture, myopathy, and pulmonary fibrosis, as well as hereditary pancreatic exocrine dysfunction, among other disorders [53, 54]. Our study suggests the important role of *FAM111B* and *FAM111A* in cell cycle classification, which is consistent with previous studies. Meanwhile, in the future, we should focus on the association between rare diseases caused by these two genes and the cell cycle.

The next identified gene is *PIF1* (ENSG00000140451), which produces a DNA-dependent ATP-metabolizing enzyme that acts as a 5′ to 3′ DNA helicase. *PIF1* plays a role in maintaining telomeres and assisting DNA replication in humans [55, 56]. In addition, PIF1 contributes to the excision of G-quadruplex structures, thus assisting in DNA damage repair [57]. There is a significantly high expression during the S phase of the cell cycle, especially when DNA is damaged.

*H4C3* (ENSG00000197061) is a member of the histone H4 family, and its primary function is to aid DNA replication-dependent nucleosome assembly. Mutations of *H4C3* have been reported to cause errors in the DNA replication process and DNA damage repair process, which will affect the cell cycle progression [58]. In addition, numerous studies have shown that posttranslational modifications of histone H4 are closely associated with cell cycle progression [59, 60]. This finding demonstrates the superiority of our approach, suggesting that future studies of genes associated with the cell cycle phases should be pursued at the multiomic level.

The last gene that ranked highly in all three feature ranking methods was *UBE2C* (ENSG00000175063), which encodes a member of the E2 ubiquitin-conjugating enzyme family. *UBE2C* promotes the degradation of various target proteins during cell cycle progression, including mitotic cyclins, as a component of the anaphase promoting complex/cyclosome (APC/C) [61, 62]. FOXM1 (Forkhead Box Protein M1) is a transcription factor that regulates the expression of genes related to G1/S, G2/M transition, and M phase progression, and *UBE2C* is one of the transcriptional targets of FOXM1 [63]. These studies further illustrate the important role of *UBE2C* in regulating the transition between cell cycle phases.

### 4.2. Highly Ranked Features Identified by Three Algorithms

The first identified gene is *FBXO5*, which encodes a protein that is a member of the F-box family. Cell cycle regulation relies on protein degradation by the ubiquitin-proteasome system, of which APC/C is a major ubiquitin ligase [64]. As early as 2005, it was found that FBXO5 acted as a cell cycle regulator that could regulate the timing of cell entry into the S phase by inhibiting APC/C [65]. In 2018, two transcriptional isoforms of *FBXO5* were found to contribute to the migration and differentiation of human periodontal membrane stem cells (HPDLSCs), which provides a potential target for periodontal tissue regeneration [66]. This further illustrates the important role of *FBXO5* in cell growth and development. Studies have also found that *FBXO5* is associated with poor prognosis in ovarian cancer, prostate cancer, and hepatocellular carcinoma [67–69]. Reviewing previous studies, we can find that *FBXO5* not only plays an important role in the cell cycle but also plays a key role in cancer development, which clarifies the association between the cell cycle and cancer. This finding also illustrates the superiority of our approach in discovering this potential connection.

*TOP2A*, another gene on both of the prediction lists, encodes a DNA topoisomerase, an enzyme required for superhelical DNA dehiscence that alters the topology of DNA during transcription. Previous studies have shown that the exercise of the normal function of *TOP2A* is essential for late G2 and M phase chromosome formation [70]. The treatment of *TOP2A* with conditional depletion in mammals revealed a longer duration of the cell cycle, which was attributed to the fact that *TOP2A* depletion led to impaired chromosome condensation and segregation, thereby resulting in the frequent failure of cell division [71]. While the above studies illustrate the key role of *TOP2A* in the G2/M transition, scientists have discovered that *TOP2A* also functions in other periods of the cell cycle as research continues. In 2016, scientists discovered that *TOP2A* plays a role in the recruitment of mitophagy in the S phase [72]. Phosphorylation-deficient *TOP2A* accelerates the recruitment of mitophagy in S phase, which leads to the premature segregation of mitotic DNA [72]. In recent years, increasing studies have found that *TOP2A* plays an important role in the development of cancer. One study found that *TOP2A* promotes the proliferation and migration of gallbladder cancer cells through the PI3K/Akt/mTOR pathway, suggesting that *TOP2A* is associated with the poor prognosis of patients [73]. Changes in DNA structure during the cell cycle are complex and diverse, and *TOP2A* alters the structure of DNA along with the potential function of many genes, thereby resulting in a range of cancer-associated functional loci. Our approach has superior performance in finding such genes associated with cell cycle and disease.

*FHL2*, four and a half LIM domain protein 2, is a protein that consists of only the LIM structural domain. Based on the functional diversity of LIM structural domains, FHL2 can interact with various proteins and thus play an important role in cell proliferation, differentiation, and signaling [74, 75]. Findings suggest that the interaction of FHL2 and iASPP inhibits apoptosis and promotes cell proliferation [76]. If *FHL2* is knocked out, then it causes the cell cycle to stay in the G0/G1 phase, thereby inhibiting cell proliferation [76]. In addition, FHL2 can also interact directly with HIF-1alpha to inhibit transcriptional activity, suggesting that FHL2 is a negative regulator of HIF-1 activity [77]. The specificity of the FHL2 structure allows it to act as a transcriptional cofactor that links to various signaling pathways to transcriptional regulation. Interestingly, the expression level of FHL2 is also associated with the development of cancer [78]. *FHL2* knockdown downregulated the expression of key proteins in the PI3K/AKT/mTOR signaling pathway, thereby promoting apoptosis and inhibiting the proliferation of cervical cancer cells [79]. This instance suggests that *FHL2* may serve as a potential target for cancer therapy.

*CDCA2* and *CDCA5* encode two cell cycle proteins, both of which can interact with lectins during the cell cycle, thereby stabilizing the chromosome structure [80, 81]. CDCA5 was coexpressed with CDK1 in gastric cancer, thereby affecting the normal cell cycle transition and thus leading to the proliferation of gastric cancer cells [82]. CDCA5 and CDK1 were ranked as top-ranked genes that affect the cell cycle in our approach, indicating the potential role of our approach in revealing this coexpression relationship. In colorectal cancer, Feng et al. found that the upregulation of CDCA2 expression led to the upregulation of CCND1, which affected cell cycle progression and activated the PI3K/AKT pathway, leading to the proliferation of cancer cells [83]. Notably, the inhibition of CDCA2 and CDCA5 activities in lung and gastric adenocarcinoma, respectively, resulted in the downregulation of CCNE1 expression, thereby inhibiting cancer cell proliferation and blocking it in the G1 phase [84, 85]. As two proteins closely related to the cell cycle, they share a similar mechanism in regulating cell proliferation. The ability of our method to identify such genes with similar mechanisms further illustrates the superiority of our approach.

### 4.3. Functional Enrichment Analyses on Optimum Gene Features

Here, we find that mRMR, MCFS, and SHAP by LightGBM achieve optimal classification in the IFS method when the number of features is 195, 526, and 222, respectively. Therefore, we selected the 195, 526, and 222 genes in each ranked gene list for GO terms and KEGG pathway analysis and filtered enriched entries of interest based on the FDR < 0.05 threshold. The enrichment analysis results show that all GO terms are directly associated with the cell cycle. The most significantly enriched pathway for KEGG is the cell cycle, thereby confirming the reliability of our selection of these genes as a criterion for differentiating cells with various cell cycles. The detailed discussion on significant GO terms and KEGG pathways can be observed below.

Top GO terms and KEGG pathways are selected for analysis. We find that enrichment in biological processes (BP) is related to the separation of chromosomes and nucleus, such as GO:0098813, which refers to the biological process of nuclear chromosome segregation. In mitosis, the normal segregation of chromosomes is the basis for daughter cells to have a complete genome [86]. The main enrichment results of cellular component (CC) are chromosomes and nucleus, such as GO:0098687, which refers to the CC of the chromosomal region. The enrichment results of molecular function (MF) are mainly related to the activity and topology of DNA, such as GO:0017116, which refers to the MF of single-stranded DNA helicase activity. The central process of the cell cycle is the replication of the genome and its correct transmission to the daughter cells. The activity and topology of the DNA ensure that it initiates the DNA replication process with the correct structure in the S phase [87–89]. hsa04115, which refers to the p53 signaling pathway, appeared in our KEGG pathway enrichment results. Many studies in the past have shown that the activation of p53 leads to cell cycle arrest and apoptosis [90, 91]. Through the detailed analysis of GO and KEGG, we find that they are both linked to the cell cycle, thereby indicating the accuracy of our prediction method.

After verifying the reliability of genes selected by computational methods in differentiating the cell cycle, we are more interested in determining the existence of genes that regulate the cell cycle while participating in other processes, such as disease development and apoptosis because this has the potential to instruct the cell to influence these processes in a way that regulates the cell cycle. In the KEGG pathway analysis, we find that the Fanconi anemia pathway is significantly enriched. Fanconi anemia is a rare genetic disease characterized by genomic instability and cellular sensitivity to DNA cross-linking agents [92]. In addition, when the Fanconi anemia pathway is damaged, it causes the S phase DNA interstrand cross-link repair process to fail to proceed properly [92, 93]. This finding suggests the interaction between the development of disease and cell cycle. Our study provides several new cell cycle-related genes that may be involved in disease progression. In the future, we should pay more attention to these other processes mediated by cell cycle genes.

### 4.4. Rules for Quantitative Cell Cycle Classification

Apart from the qualitative analysis of genes described above, we also establish a series of rules for cells in each specific cell cycle. If a classification rule is applicable in at least two DT models, then we consider such rule to be extremely important for its role, which is analyzed in detail as follows.

The first decision rule is aimed at identifying the cells in the G1 phase from the three other phases. This purpose is specifically indicated by the low expression of CDK1, H4C3, CDC45, and E2F2 and the high expression of TXNL1. CDK1 gradually began to accumulate after G1 and is inactivated upon exit from mitosis, thereby maintaining a low expression in G1 [48]. H4C3 belongs to the histone H4 family, and its main function relates to its involvement in DNA replication-dependent nucleosome assembly [94]. Histone synthesis occurs mainly in the S phase. Thus, the expression level remains low in the G1 phase. *CDC45* and *E2F2* have been the key genes from the G1 to S phase. The high expression of CDC45 leads to the activation of S phase initiation and induces an early entry from G1 to S phase [95]. The binding of E2F2 to its inhibitory miRNA decreases the level of E2F2 expression, thereby blocking cell division in the G1 phase [96, 97]. This finding suggests that high expressions of CDC45 and E2F2 cause the cell cycle to advance from the G1 to the S phase. The requirement for low expression levels of these two genes in our rule to distinguish G1 cells is consistent with the above pattern and demonstrates the reliability of our rule. In previous studies, high levels of TXNL1 expression would enhance the action of transcriptional repressors by binding to the transcription factor B-Myb [98]. This finding suggests that the high expression level of TXNL1 leads to the failure of normal transition to the G1 phase, which is consistent with our classification rules.

The second rule is mainly related to distinguishing G2/M phase cells. This rule is demonstrated by the high expression of CDK1, PIF1, CENPF, and SRRM2 and the low expression of CCNE1, CCNE2, ZWINT, and ARPC1B. As discussed previously, *CDK1* and *H4C3* play important roles in the G2 to M phase transition process and S to G2 phase histone synthesis and nucleosome assembly, respectively. Therefore, these two genes are abundantly expressed in cells during this period. The expression of PIF1 is regulated by the cell cycle [99]. Its expression in cells in the G1 phase is low and rises as the cell cycle progresses, peaking at the end of the S and G2 phases [99]. Similar to PIF1, CENPF expression accumulates at the S phase and peaks at G2 and M phases [100]. SRRM2 has been reported to form complexes with other proteins and contribute to chromosome segregation and genome stabilization by ensuring the splicing of specific pre-mRNAs [101]. Thus, high expression of SRRM2 promotes better binding to other proteins to form complexes, thereby assisting in M phase functions. These genes with high expression levels all exhibit cell cycle-specific expression properties, which are useful for identifying cells that are in a specific cell cycle. The proteins encoded by *CCNE1* and *CCNE2* are collectively known as cyclin E [102]. They facilitate the transition from the G1 to S phase by binding to CDK2 kinase [102]. CCNE1 exhibits cell cycle-specific expression. It begins to accumulate during the G1 to S phase transition and begins to degrade upon entry into the S phase [103]. In addition, high expression of CCNE1 and CCNE2 leads to genomic instability, which affects the normal progression of the M phase [104]. This finding is consistent with the restriction on the expression of these two genes in our rules to distinguish normal G2/M phase cells [105, 106]. *ZWINT* affects the meiotic process by recruiting kinetochores and assisting in the segregation of homologous chromosomes [107]. ARPC1B is an activator and substrate of Aurora A, which regulates centrosome stability [108]. In addition, small interfering RNA-mediated depletion of *ARPC1B* inhibited G2-M phase cell cycle progression [108].

The last rule is associated with cells in the S phase. It is specifically indicated by the high expression of CDK1, MCM5, H4C3, BRCA1, and PKMYT1 and the low expression of PIF1, ANKRD20A8P, AXL, and TNFAIP8L1. As introduced above, CDK1 begins to accumulate after G1 and plays a role in the G1 to S phase transition. Thus, CDK1 has high expression in the S phase. *H4C3* is also consistent with the previously discussed results. It mainly encodes histones, and the synthesis of histones occurs mainly in the S phase. Hence, it is highly expressed in this period. *MCM5* has been shown to regulate the cell cycle by assisting in DNA replication and DNA damage repair [109, 110]. High MCM5 expression regulates the DNA damage checkpoint [109, 110]. This instance suggests that high MCM5 expression helps S phase DNA perform normal synthetic functions and repair damaged DNA. Mutations in *BRCA1* have been widely associated with the development of breast cancer [111]. In addition, BRCA1 itself has been associated with gene regulation. It can induce *GADD45*, a p53-regulated and stress-inducible gene that plays an important role in responding to DNA damage [112]. This finding shows that high expression of BRCA1 ensures that DNA can be repaired in a timely manner when it is damaged, thereby allowing DNA to be replicated in a correct and orderly manner. PKMYT1 knockdown increased the expression of DNA damage markers [113]. In addition, PKMYT1 overexpression is found in Kaposi's sarcoma-associated herpesvirus-infected S phase cells, indicating its potential role in S phase transition [114]. Among these low expressed genes, *PIF1* is cycle-specific in its expression, as previously described, and the expression begins to accumulate in the S phase. Thus, the expression is not excessive. Knockdown of TNFAIP8L1 and its homologs resulted in a cell cycle stuck in the S phase, suggesting that its low expression caused the cells to show more S phase characteristics [115]. In previous studies, the association of *ANKRD20A8P* and *AXL* with the cell cycle is not clearly indicated, so they may serve as potential targets for indicating the cell cycle.

## 5. Conclusions

In the present study, we apply advanced and widely used computational methods to single-cell RNA sequencing data to explore additional cell cycle information. In summary, our study has identified a series of potential cell cycle-related genes that can serve as novel cell cycle markers and contribute to exploring the molecular mechanisms of cell cycle regulation further. In addition, we present an optimal classifier for cell cycle prediction, which has been trained with a large amount of single-cell data to achieve excellent classification performance. Finally, the quantitative decision rules in the classification model provide direct clues for distinguishing cells in different phases.

## Figures and Tables

**Table 1:** Performance of optimal classifiers corresponding to the optimal feature subsets based on different classification algorithms and feature ranking algorithms.

Feature ranking algorithms	Classification algorithm	Number of features	ACC	MCC
mRMR	Random forest	195	0.906	0.859
	Support vector machine	124	0.884	0.826
	k-nearest neighbor	37	0.869	0.803
	Decision tree	158	0.836	0.753

MCFS	Random forest	52	0.906	0.859
	Support vector machine	526	0.918	0.878
	k-nearest neighbor	76	0.881	0.822
	Decision tree	479	0.842	0.762

SHAP by LightGBM	Random forest	87	0.917	0.875
	Support vector machine	222	0.917	0.875
	k-nearest neighbor	18	0.866	0.800
	Decision tree	103	0.856	0.784

**Table 2:** Five genes identified by the IFS method on all feature lists generated by three feature ranking algorithms.

Ensembl ID	Gene symbol	Description
ENSG00000140451	PIF1	PIF1 5′-to-3′ DNA helicase
ENSG00000170312	CDK1	Cyclin-dependent kinase 1
ENSG00000175063	UBE2C	Ubiquitin-conjugating enzyme E2 C
ENSG00000189057	FAM111B	FAM111 trypsin like peptidase B
ENSG00000197061	H4C3	H4 clustered histone 3

## Data Availability

The original data used to support the findings of this study are available at Gene Expression Omnibus database (https://www.ncbi.nlm.nih.gov/geo/query/acc.cgi?acc=GSE146773).

## Supplementary Materials

Supplementary 1 Table S1: the feature list filtered by Boruta and ordered by three feature ranking algorithms.

Supplementary 2 Table S2: performance of IFS with different classification algorithms.

Supplementary 3 Table S3: feasible feature subsets selected by three feature selection methods and their intersection.

Supplementary 4 Table S4: classification rules generated by the optimal DT classifier based on the feature lists generated by three feature ranking algorithms.
