# Supplementary material for: Identification of Human Cell Cycle Phase Markers Based on Single-Cell RNA-Seq Data by Using Machine Learning Methods
Source: Biomed Res Int. 2022 Aug 13;2022:2516653. doi: 10.1155/2022/2516653 (PMC9393965; doi:10.1155/2022/2516653)
Supplement: Supplementary 1 — Table S1: the feature list filtered by Boruta and ordered by three feature ranking algorithms. [file 2516653.f1.pdf]

Table S1. The feature list filtered by Boruta and ordered by three feature ranking algorithms

(1) Feature list yielded by mRMR method

Rank	Ensembl ID	Gene symbol
1	ENSG00000170312	CDK1
2	ENSG00000189057	FAM111B
3	ENSG00000112029	FBXO5
4	ENSG00000131747	TOP2A
5	ENSG00000166801	FAM111A
6	ENSG00000115641	FHL2
7	ENSG00000123485	HJURP
8	ENSG00000171320	ESCO2
9	ENSG00000131475	VPS25
10	ENSG00000175063	UBE2C
11	ENSG00000051341	POLQ
12	ENSG00000112118	MCM3
13	ENSG00000178999	AURKB
14	ENSG00000198331	HYLS1
15	ENSG00000171848	RRM2
16	ENSG00000085999	RAD54L
17	ENSG00000184661	CDCA2
18	ENSG00000177943	MAMDC4
19	ENSG00000156802	ATAD2
20	ENSG00000093009	CDC45
21	ENSG00000140451	PIF1
22	ENSG00000165304	MELK
23	ENSG00000197061	H4C3
24	ENSG00000186185	KIF18B
25	ENSG00000076003	MCM6
26	ENSG00000076248	UNG
27	ENSG00000146670	CDCA5
28	ENSG00000173207	CKS1B
29	ENSG00000102384	CENPI
30	ENSG00000274997	H2AC12
31	ENSG00000137807	KIF23
32	ENSG00000105011	ASF1B
33	ENSG00000101447	FAM83D
34	ENSG00000121152	NCAPH
35	ENSG00000259848	LOC442028
36	ENSG00000182481	KPNA2
37	ENSG00000012048	BRCA1
38	ENSG00000229089	ANKRD20A8P

39	ENSG00000198056	PRIM1
40	ENSG00000162063	CCNF
41	ENSG00000101104	PABPC1L
42	ENSG00000178719	GRINA
43	ENSG00000166803	PCLAF
44	ENSG00000135451	TROAP
45	ENSG00000094804	CDC6
46	ENSG00000077152	UBE2T
47	ENSG00000276368	H2AC14
48	ENSG00000121716	PILRB
49	ENSG00000169607	CKAP2L
50	ENSG00000175305	CCNE2
51	ENSG00000164104	HMGB2
52	ENSG00000123473	STIL
53	ENSG00000176890	TYMS
54	ENSG00000145386	CCNA2
55	ENSG00000100297	MCM5
56	ENSG00000149636	DSN1
57	ENSG00000168298	H1-4
58	ENSG00000122952	ZWINT
59	ENSG00000171793	CTPS1
60	ENSG00000111247	RAD51AP1
61	ENSG00000106367	AP1S1
62	ENSG00000196230	TUBB
63	ENSG00000178966	RMI1
64	ENSG00000075218	GTSE1
65	ENSG00000146278	PNRC1
66	ENSG00000132646	PCNA
67	ENSG00000134057	CCNB1
68	ENSG00000105173	CCNE1
69	ENSG00000100097	LGALS1
70	ENSG00000104738	MCM4
71	ENSG00000134690	CDCA8
72	ENSG00000137310	TCF19
73	ENSG00000127483	HP1BP3
74	ENSG00000148773	MKI67
75	ENSG00000077514	POLD3
76	ENSG00000183856	IQGAP3
77	ENSG00000031691	CENPQ
78	ENSG00000101412	E2F1
79	ENSG00000111665	CDCA3
80	ENSG00000213853	EMP2

81	ENSG00000007968	E2F2
82	ENSG00000065328	MCM10
83	ENSG00000118193	KIF14
84	ENSG00000088986	DYNLL1
85	ENSG00000119801	YPEL5
86	ENSG00000101773	RBBP8
87	ENSG00000142945	KIF2C
88	ENSG00000106211	HSPB1
89	ENSG00000130299	GTPBP3
90	ENSG00000163808	KIF15
91	ENSG00000123975	CKS2
92	ENSG00000128510	CPA4
93	ENSG00000013573	DDX11
94	ENSG00000121211	MND1
95	ENSG00000127922	SEM1
96	ENSG00000006327	TNFRSF12A
97	ENSG00000178074	C2orf69
98	ENSG00000139211	AMIGO2
99	ENSG00000135476	ESPL1
100	ENSG00000165480	SKA3
101	ENSG00000205352	PRR13
102	ENSG00000126368	NR1D1
103	ENSG00000163535	SGO2
104	ENSG00000178607	ERN1
105	ENSG00000119669	IRF2BPL
106	ENSG00000129810	SGO1
107	ENSG00000170889	RPS9
108	ENSG00000137804	NUSAP1
109	ENSG00000070950	RAD18
110	ENSG00000124610	H1-1
111	ENSG00000138182	KIF20B
112	ENSG00000117724	CENPF
113	ENSG00000136982	DSCC1
114	ENSG00000091164	TXNL1
115	ENSG00000112742	TTK
116	ENSG00000278259	MYO19
117	ENSG00000105355	PLIN3
118	ENSG00000115163	CENPA
119	ENSG00000118655	DCLRE1B
120	ENSG00000235571	SNX18P14
121	ENSG00000163918	RFC4
122	ENSG00000164611	PTTG1

123	ENSG00000151725	CENPU
124	ENSG00000128951	DUT
125	ENSG00000120802	TMPO
126	ENSG00000134291	TMEM106C
127	ENSG00000100714	MTHFD1
128	ENSG00000198763	ND2
129	ENSG00000133119	RFC3
130	ENSG00000213024	NUP62
131	ENSG00000142731	PLK4
132	ENSG00000025770	NCAPH2
133	ENSG00000103995	CEP152
134	ENSG00000141232	TOB1
135	ENSG00000166508	MCM7
136	ENSG00000167553	TUBA1C
137	ENSG00000102362	SYTL4
138	ENSG00000091651	ORC6
139	ENSG00000118263	KLF7
140	ENSG00000178974	FBXO34
141	ENSG00000152253	SPC25
142	ENSG00000221829	FANCG
143	ENSG00000170734	POLH
144	ENSG00000088325	TPX2
145	ENSG00000132823	OSER1
146	ENSG00000175573	C11orf68
147	ENSG00000178913	TAF7
148	ENSG00000161800	RACGAP1
149	ENSG00000129484	PARP2
150	ENSG00000198033	TUBA3C
151	ENSG00000263513	FAM72C
152	ENSG00000130816	DNMT1
153	ENSG00000175130	MARCKSL1
154	ENSG00000162607	USP1
155	ENSG00000168078	PBK
156	ENSG00000169242	EFNA1
157	ENSG00000154473	BUB3
158	ENSG00000100100	PIK3IP1
159	ENSG00000111206	FOXM1
160	ENSG00000183864	TOB2
161	ENSG00000101608	MYL12A
162	ENSG00000074266	EED
163	ENSG00000149257	SERPINH1
164	ENSG00000175606	TMEM70

165	ENSG00000123737	EXOSC9
166	ENSG00000165490	DDIAS
167	ENSG00000143933	CALM2
168	ENSG00000054654	SYNE2
169	ENSG00000122483	CCDC18
170	ENSG00000033030	ZCCHC8
171	ENSG00000101945	SUV39H1
172	ENSG00000121388	Pseudogene
173	ENSG00000170779	CDCA4
174	ENSG00000146425	DYNLT1
175	ENSG00000087586	AURKA
176	ENSG00000112576	CCND3
177	ENSG00000049541	RFC2
178	ENSG00000080986	NDC80
179	ENSG00000091436	MAP3K20
180	ENSG00000131470	PSMC3IP
181	ENSG00000117399	CDC20
182	ENSG00000180304	OAZ2
183	ENSG00000165501	LRR1
184	ENSG00000169679	BUB1
185	ENSG00000116717	GADD45A
186	ENSG00000154640	BTG3
187	ENSG00000079482	OPHN1
188	ENSG00000100479	POLE2
189	ENSG00000188985	DHFRP1
190	ENSG00000171241	SHCBP1
191	ENSG00000156970	BUB1B
192	ENSG00000184678	H2BC21
193	ENSG00000051180	RAD51
194	ENSG00000198826	ARHGAP11A
195	ENSG00000188529	SRSF10
196	ENSG00000185787	MORF4L1
197	ENSG00000108106	UBE2S
198	ENSG00000078900	TP73
199	ENSG00000101003	GINS1
200	ENSG00000126226	PCID2
201	ENSG00000117069	ST6GALNAC5
202	ENSG00000137812	KNL1
203	ENSG00000166483	WEE1
204	ENSG00000171223	JUNB
205	ENSG00000204382	XAGE1B
206	ENSG00000090889	KIF4A

207	ENSG00000070761	CFAP20
208	ENSG00000114315	HES1
209	ENSG00000077684	JADE1
210	ENSG00000162073	PAQR4
211	ENSG00000165891	E2F7
212	ENSG00000144554	FANCD2
213	ENSG00000049192	ADAMTS6
214	ENSG00000166851	PLK1
215	ENSG00000165502	RPL36AL
216	ENSG00000131153	GINS2
217	ENSG00000284906	ARHGAP11B
218	ENSG00000109971	HSPA8
219	ENSG00000120334	CENPL
220	ENSG00000166965	RCCD1
221	ENSG00000235109	ZSCAN31
222	ENSG00000072571	HMMR
223	ENSG00000170425	ADORA2B
224	ENSG00000116830	TTF2
225	ENSG00000104064	GABPB1
226	ENSG00000130202	NECTIN2
227	ENSG00000183963	SMTN
228	ENSG00000105516	DBP
229	ENSG00000118971	CCND2
230	ENSG00000213551	DNAJC9
231	ENSG00000126787	DLGAP5
232	ENSG00000075618	FSCN1
233	ENSG00000206625	RNU6-1
234	ENSG00000152359	POC5
235	ENSG00000176222	ZNF404
236	ENSG00000131462	TUBG1
237	ENSG00000150991	UBC
238	ENSG00000113456	RAD1
239	ENSG00000158373	H2BC5
240	ENSG00000148019	CEP78
241	ENSG00000167601	AXL
242	ENSG00000013810	TACC3
243	ENSG00000144227	NXPH2
244	ENSG00000075702	WDR62
245	ENSG00000177426	TGIF1
246	ENSG00000011426	ANLN
247	ENSG00000196866	H2AC7
248	ENSG00000136492	BRIP1

249	ENSG00000282988	Pseudogene
250	ENSG00000163507	CIP2A
251	ENSG00000129534	MIS18BP1
252	ENSG00000102804	TSC22D1
253	ENSG00000104147	OIP5
254	ENSG00000184047	DIABLO
255	ENSG00000162614	NEXN
256	ENSG00000112759	SLC29A1
257	ENSG00000198901	PRC1
258	ENSG00000168140	VASN
259	ENSG00000223564	CYP4F32P
260	ENSG00000176974	SHMT1
261	ENSG00000117616	RSRP1
262	ENSG00000136108	CKAP2
263	ENSG00000105270	CLIP3
264	ENSG00000147813	NAPRT
265	ENSG00000119969	HELLS
266	ENSG00000006625	GGCT
267	ENSG00000182197	EXT1
268	ENSG00000100162	CENPM
269	ENSG00000213347	MXD3
270	ENSG00000142871	CCN1
271	ENSG00000179958	DCTPP1
272	ENSG00000065548	ZC3H15
273	ENSG00000109805	NCAPG
274	ENSG00000166002	SMCO4
275	ENSG00000164087	POC1A
276	ENSG00000123933	MXD4
277	ENSG00000143367	TUFT1
278	ENSG00000101639	CEP192
279	ENSG00000159335	PTMS
280	ENSG00000101138	CSTF1
281	ENSG00000169490	TM2D2
282	ENSG00000164251	F2RL1
283	ENSG00000128965	CHAC1
284	ENSG00000177917	ARL6IP6
285	ENSG00000040275	SPDL1
286	ENSG00000146410	MTFR2
287	ENSG00000126945	HNRNPH2
288	ENSG00000149483	TMEM138
289	ENSG00000121621	KIF18A
290	ENSG00000122644	ARL4A

291	ENSG00000182378	PLCXD1
292	ENSG00000130695	CEP85
293	ENSG00000188229	TUBB4B
294	ENSG00000232502	Pseudogene
295	ENSG00000147155	EBP
296	ENSG00000140332	TLE3
297	ENSG00000142541	RPL13A
298	ENSG00000101057	MYBL2
299	ENSG00000130921	MTRFR
300	ENSG00000088305	DNMT3B
301	ENSG00000164754	RAD21
302	ENSG00000101574	METTL4
303	ENSG00000117318	ID3
304	ENSG00000065911	MTHFD2
305	ENSG00000128016	ZFP36
306	ENSG00000163655	GMPS
307	ENSG00000042088	TDP1
308	ENSG00000143761	ARF1
309	ENSG00000085840	ORC1
310	ENSG00000249859	PVT1
311	ENSG00000125898	FAM110A
312	ENSG00000135211	TMEM60
313	ENSG00000205212	CCDC144NL
314	ENSG00000123416	TUBA1B
315	ENSG00000198680	TUSC1
316	ENSG00000123146	ADGRE5
317	ENSG00000136699	SMPD4
318	ENSG00000186472	PCLO
319	ENSG00000225630	MTND2P28
320	ENSG00000113810	SMC4
321	ENSG00000111321	LTBR
322	ENSG00000113369	ARRDC3
323	ENSG00000149548	CCDC15
324	ENSG00000109255	NMU
325	ENSG00000129195	PIMREG
326	ENSG00000124216	SNAI1
327	ENSG00000175592	FOSL1
328	ENSG00000214756	CSKMT
329	ENSG00000132485	ZRANB2
330	ENSG00000009954	BAZ1B
331	ENSG00000134802	SLC43A3
332	ENSG00000176105	YES1

333	ENSG00000113569	NUP155
334	ENSG00000188610	FAM72B
335	ENSG00000131446	MGAT1
336	ENSG00000138385	SSB
337	ENSG00000188486	H2AX
338	ENSG00000162231	NXF1
339	ENSG00000117450	PRDX1
340	ENSG00000117650	NEK2
341	ENSG00000164649	CDCA7L
342	ENSG00000183255	PTTG1IP
343	ENSG00000120694	HSPH1
344	ENSG00000174371	EXO1
345	ENSG00000109674	NEIL3
346	ENSG00000204580	DDR1
347	ENSG00000105856	HBP1
348	ENSG00000138346	DNA2
349	ENSG00000145779	TNFAIP8
350	ENSG00000006634	DBF4
351	ENSG00000132341	RAN
352	ENSG00000118680	MYL12B
353	ENSG00000140319	SRP14
354	ENSG00000024526	DEPDC1
355	ENSG00000105821	DNAJC2
356	ENSG00000100526	CDKN3
357	ENSG00000103121	CMC2
358	ENSG00000186283	TOR3A
359	ENSG00000092140	G2E3
360	ENSG00000179094	PER1
361	ENSG00000196787	H2AC11
362	ENSG00000256618	MTRNR2L1
363	ENSG00000116133	DHCR24
364	ENSG00000163950	SLBP
365	ENSG00000152518	ZFP36L2
366	ENSG00000118181	RPS25
367	ENSG00000165886	UBTD1
368	ENSG00000145526	CDH18
369	ENSG00000115946	PNO1
370	ENSG00000170540	ARL6IP1
371	ENSG00000104267	CA2
372	ENSG00000130429	ARPC1B
373	ENSG00000158402	CDC25C
374	ENSG00000168916	ZNF608

375	ENSG00000010292	NCAPD2
376	ENSG00000162433	AK4
377	ENSG00000127337	YEATS4
378	ENSG00000101868	POLA1
379	ENSG00000143621	ILF2
380	ENSG00000187231	SESTD1
381	ENSG00000178105	DDX10
382	ENSG00000164211	STARD4
383	ENSG00000204899	MZT1
384	ENSG00000127948	POR
385	ENSG00000087077	TRIP6
386	ENSG00000164924	YWHAZ
387	ENSG00000167978	SRRM2
388	ENSG00000173848	NET1
389	ENSG00000108055	SMC3
390	ENSG00000095777	MYO3A
391	ENSG00000096093	EFHC1
392	ENSG00000161980	POLR3K
393	ENSG00000075426	FOSL2
394	ENSG00000139697	SBNO1
395	ENSG00000140350	ANP32A
396	ENSG00000089685	BIRC5
397	ENSG00000265790	RNASEH1P1
398	ENSG00000143476	DTL
399	ENSG00000184260	H2AC20
400	ENSG00000135046	ANXA1
401	ENSG00000115306	SPTBN1
402	ENSG00000100242	SUN2
403	ENSG00000172893	DHCR7
404	ENSG00000122565	CBX3
405	ENSG00000146834	MEPCE
406	ENSG00000232388	SMIM26
407	ENSG00000138160	KIF11
408	ENSG00000112984	KIF20A
409	ENSG00000163002	NUP35
410	ENSG00000146263	MMS22L
411	ENSG00000158246	TENT5B
412	ENSG00000173456	RNF26
413	ENSG00000109321	AREG
414	ENSG00000207507	RNU6-9
415	ENSG00000109861	CTSC
416	ENSG00000198890	PRMT6

417	ENSG00000057019	DCBLD2
418	ENSG00000136122	BORA
419	ENSG00000129474	AJUBA
420	ENSG00000106462	EZH2
421	ENSG00000134375	TIMM17A
422	ENSG00000105372	RPS19
423	ENSG00000276180	H4C9
424	ENSG00000104671	DCTN6
425	ENSG00000109099	PMP22
426	ENSG00000198554	WDHD1
427	ENSG00000100401	RANGAP1
428	ENSG00000100941	PNN
429	ENSG00000096433	ITPR3
430	ENSG00000121957	GPSM2
431	ENSG00000138175	ARL3
432	ENSG00000176014	TUBB6
433	ENSG00000092969	TGFB2
434	ENSG00000137135	ARHGEF39
435	ENSG00000165181	SHOC1
436	ENSG00000160789	LMNA
437	ENSG00000100632	ERH
438	ENSG00000013588	GPRC5A
439	ENSG00000173068	BNC2
440	ENSG00000129173	E2F8
441	ENSG00000128245	YWHAH
442	ENSG00000136997	MYC
443	ENSG00000124172	ATP5F1E
444	ENSG00000117139	KDM5B
445	ENSG00000102977	ACD
446	ENSG00000197956	S100A6
447	ENSG00000138180	CEP55
448	ENSG00000061337	LZTS1
449	ENSG00000166949	SMAD3
450	ENSG00000197256	KANK2
451	ENSG00000115875	SRSF7
452	ENSG00000148677	ANKRD1
453	ENSG00000078401	EDN1
454	ENSG00000172432	GTPBP2
455	ENSG00000128708	HAT1
456	ENSG00000171490	RSL1D1
457	ENSG00000121741	ZMYM2
458	ENSG00000198088	NUP62CL

459	ENSG00000143815	LBR
460	ENSG00000104635	SLC39A14
461	ENSG00000161888	SPC24
462	ENSG00000197299	BLM
463	ENSG00000171552	BCL2L1
464	ENSG00000144136	SLC20A1
465	ENSG00000100519	PSMC6
466	ENSG00000164985	PSIP1
467	ENSG00000173559	NABP1
468	ENSG00000204370	SDHD
469	ENSG00000167552	TUBA1A
470	ENSG00000196550	FAM72A
471	ENSG00000114346	ECT2
472	ENSG00000171867	PRNP
473	ENSG00000068489	PRR11
474	ENSG00000183337	BCOR
475	ENSG00000157191	NECAP2
476	ENSG00000185650	ZFP36L1
477	ENSG00000133121	STARD13
478	ENSG00000189241	TSPYL1
479	ENSG00000170264	FAM161A
480	ENSG00000136436	CALCOCO2
481	ENSG00000072864	NDE1
482	ENSG00000087086	FTL
483	ENSG00000167325	RRM1
484	ENSG00000207041	RNU6-3P
485	ENSG00000131737	KRT34
486	ENSG00000197172	MAGEA6
487	ENSG00000166140	ZFYVE19
488	ENSG00000196531	NACA
489	ENSG00000180573	H2AC6
490	ENSG00000170545	SMAGP
491	ENSG00000115738	ID2
492	ENSG00000182541	LIMK2
493	ENSG00000166441	RPL27A
494	ENSG00000187051	RPS19BP1
495	ENSG00000132475	H3-3B
496	ENSG00000231007	CDC20P1
497	ENSG00000270882	H4C14
498	ENSG00000124766	SOX4
499	ENSG00000153233	PTPRR
500	ENSG00000034510	TMSB10

501	ENSG00000120129	DUSP1
502	ENSG00000101224	CDC25B
503	ENSG00000186193	SAPCD2
504	ENSG00000276043	UHRF1
505	ENSG00000149798	CDC42EP2
506	ENSG00000168061	SAC3D1
507	ENSG00000106305	AIMP2
508	ENSG00000188312	CENPP
509	ENSG00000120539	MASTL
510	ENSG00000056097	ZFR
511	ENSG00000151466	SCLT1
512	ENSG00000140443	IGF1R
513	ENSG00000103540	CCP110
514	ENSG00000112972	HMGCS1
515	ENSG00000204389	HSPA1A
516	ENSG00000162062	TEDC2
517	ENSG00000117143	UAP1
518	ENSG00000116667	C1orf21
519	ENSG00000105991	HOXA1
520	ENSG00000073111	MCM2
521	ENSG00000068028	RASSF1
522	ENSG00000124635	H2BC11
523	ENSG00000125319	HROB
524	ENSG00000186638	KIF24
525	ENSG00000160753	RUSC1
526	ENSG00000176225	RTTN
527	ENSG00000115687	PASK
528	ENSG00000185567	AHNAK2
529	ENSG00000115392	FANCL
530	ENSG00000183741	CBX6
531	ENSG00000104783	KCNN4
532	ENSG00000178343	SHISA3
533	ENSG00000122566	HNRNPA2B1
534	ENSG00000146592	CREB5
535	ENSG00000187741	FANCA
536	ENSG00000161011	SQSTM1
537	ENSG00000151692	RNF144A
538	ENSG00000105325	FZR1
539	ENSG00000096384	HSP90AB1
540	ENSG00000153395	LPCAT1
541	ENSG00000138658	ZGRF1
542	ENSG00000116062	MSH6

543	ENSG00000207357	RNU6-2
544	ENSG00000119397	CNTRL
545	ENSG00000125871	MGME1
546	ENSG00000168496	FEN1
547	ENSG00000108518	PFN1
548	ENSG00000104047	DTWD1
549	ENSG00000184349	EFNA5
550	ENSG00000182010	RTKN2
551	ENSG00000167565	SERTAD3
552	ENSG00000119333	DYNC2I2
553	ENSG00000130844	ZNF331
554	ENSG00000109062	SLC9A3R1
555	ENSG00000143878	RHOB
556	ENSG00000131652	THOC6
557	ENSG00000198900	TOP1
558	ENSG00000167670	CHAF1A
559	ENSG00000137727	ARHGAP20
560	ENSG00000165732	DDX21
561	ENSG00000081320	STK17B
562	ENSG00000099194	SCD
563	ENSG00000116679	IVNS1ABP
564	ENSG00000099810	MTAP
565	ENSG00000277778	PGM5P2
566	ENSG00000143228	NUF2
567	ENSG00000213186	TRIM59
568	ENSG00000189060	H1-0
569	ENSG00000128944	KNSTRN
570	ENSG00000177084	POLE
571	ENSG00000014138	POLA2
572	ENSG00000144381	HSPD1
573	ENSG00000084733	RAB10
574	ENSG00000277161	PIGW
575	ENSG00000275616	LOC124904135
576	ENSG00000198355	PIM3
577	ENSG00000274862	LOC124904141
578	ENSG00000130669	PAK4
579	ENSG00000185803	SLC52A2
580	ENSG00000081019	RSBN1
581	ENSG00000228716	DHFR
582	ENSG00000157456	CCNB2
583	ENSG00000274585	RNU2-1
584	ENSG00000144395	CCDC150

585	ENSG00000115159	GPD2
586	ENSG00000111859	NEDD9
587	ENSG00000124422	USP22
588	ENSG00000142149	HUNK
589	ENSG00000092853	CLSPN
590	ENSG00000129355	CDKN2D
591	ENSG00000120053	GOT1
592	ENSG00000181544	FANCB
593	ENSG00000119771	KLHL29
594	ENSG00000139354	GAS2L3
595	ENSG00000176208	ATAD5
596	ENSG00000166004	CEP295
597	ENSG00000186871	ERCC6L
598	ENSG00000158201	ABHD3
599	ENSG00000274432	LOC124904137
600	ENSG00000131351	HAUS8
601	ENSG00000168010	ATG16L2
602	ENSG00000183763	TRAIP
603	ENSG00000278048	LOC124904138
604	ENSG00000172667	ZMAT3
605	ENSG00000139734	DIAPH3
606	ENSG00000112081	SRSF3
607	ENSG00000107816	LZTS2
608	ENSG00000076382	SPAG5
609	ENSG00000175455	CCDC14
610	ENSG00000115541	HSPE1
611	ENSG00000277903	LOC124904140
612	ENSG00000206899	RNU6-36P
613	ENSG00000118523	CCN2
614	ENSG00000173846	PLK3
615	ENSG00000149503	INCENP
616	ENSG00000092470	WDR76
617	ENSG00000183150	GPR19
618	ENSG00000122641	INHBA
619	ENSG00000167011	NAT16
620	ENSG00000162772	ATF3
621	ENSG00000123219	CENPK
622	ENSG00000128340	RAC2
623	ENSG00000285730	Pseudogene
624	ENSG00000140400	MAN2C1
625	ENSG00000278591	LOC124904144
626	ENSG00000138092	CENPO

627	ENSG00000186834	HEXIM1
628	ENSG00000145241	CENPC
629	ENSG00000132780	NASP
630	ENSG00000112414	ADGRG6
631	ENSG00000177879	AP3S1
632	ENSG00000138496	PARP9
633	ENSG00000274062	LOC124904142
634	ENSG00000138376	BARD1
635	ENSG00000217555	CKLF
636	ENSG00000214944	ARHGEF28
637	ENSG00000162783	IER5
638	ENSG00000273709	LOC124904136
639	ENSG00000095002	MSH2
640	ENSG00000171421	MRPL36
641	ENSG00000203811	H3C14
642	ENSG00000255152	MSH5-SAPCD1
643	ENSG00000117713	ARID1A
644	ENSG00000206932	RNU6-4P
645	ENSG00000155506	LARP1
646	ENSG00000276966	H4C5
647	ENSG00000137936	BCAR3
648	ENSG00000143942	CHAC2
649	ENSG00000160113	NR2F6
650	ENSG00000152117	SMPD4BP
651	ENSG00000140534	TICRR
652	ENSG00000082438	COBLL1
653	ENSG00000095383	TBC1D2
654	ENSG00000237649	KIFC1
655	ENSG00000139618	BRCA2
656	ENSG00000172244	C5orf34
657	ENSG00000131435	PDLIM4
658	ENSG00000206965	RNU6-5P
659	ENSG00000189337	KAZN
660	ENSG00000174738	NR1D2
661	ENSG00000151503	NCAPD3
662	ENSG00000135334	AKIRIN2
663	ENSG00000144354	CDCA7
664	ENSG00000154920	EME1
665	ENSG00000137497	NUMA1
666	ENSG00000275219	LOC124904143
667	ENSG00000214826	DDX12P

668	ENSG00000214776	Pseudogene
669	ENSG00000148248	SURF4
670	ENSG00000172765	TMCC1
671	ENSG00000068885	IFT80
672	ENSG00000221978	CCNL2
673	ENSG00000175265	GOLGA8A
674	ENSG00000066279	ASPM
675	ENSG00000214357	NEURL1B
676	ENSG00000163072	NOSTRIN
677	ENSG00000177602	HASPIN
678	ENSG00000276596	LOC124904139
679	ENSG00000169252	ADRB2
680	ENSG00000128294	TPST2
681	ENSG00000181722	ZBTB20
682	ENSG00000178295	GEN1
683	ENSG00000204388	HSPA1B
684	ENSG00000001460	STPG1
685	ENSG00000111276	CDKN1B
686	ENSG00000111788	Pseudogene
687	ENSG00000111696	NT5DC3
688	ENSG00000134222	PSRC1
689	ENSG00000149639	SOGA1
690	ENSG00000156463	SH3RF2
691	ENSG00000154839	SKA1
692	ENSG00000161692	DBF4B
693	ENSG00000160949	TONSL
694	ENSG00000160298	C21orf58
695	ENSG00000162419	GMEB1
696	ENSG00000164796	CSMD3
697	ENSG00000165494	PCF11
698	ENSG00000167513	CDT1
699	ENSG00000168282	MGAT2
700	ENSG00000169188	APEX2
701	ENSG00000171105	INSR
702	ENSG00000172167	MTBP
703	ENSG00000089693	MLF2
704	ENSG00000177595	PIDD1
705	ENSG00000180611	MB21D2
706	ENSG00000184445	KNTC1
707	ENSG00000184992	BRI3BP
708	ENSG00000187266	EPOR
709	ENSG00000186767	SPIN4

710	ENSG00000283269	Pseudogene
711	ENSG00000070882	OSBPL3
712	ENSG00000146555	SDK1
713	ENSG00000153048	CARHSP1
714	ENSG00000144048	DUSP11
715	ENSG00000215252	GOLGA8B
716	ENSG00000125885	MCM8
717	ENSG00000182752	PAPPA
718	ENSG00000250067	YJEFN3
719	ENSG00000168476	REEP4
720	ENSG00000127564	PKMYT1
721	ENSG00000160957	RECQL4
722	ENSG00000135185	TMEM243
723	ENSG00000162222	TTC9C
724	ENSG00000079616	KIF22
725	ENSG00000170852	KBTBD2
726	ENSG00000174136	RGMB
727	ENSG00000142765	SYTL1
728	ENSG00000196235	SUPT5H
729	ENSG00000171792	RHNO1
730	ENSG00000169683	LRRC45
731	ENSG00000166925	TSC22D4
732	ENSG00000026103	FAS
733	ENSG00000162929	SANBR
734	ENSG00000183955	KMT5A
735	ENSG00000075131	TIPIN
736	ENSG00000127586	CHTF18
737	ENSG00000205560	CPT1B
738	ENSG00000134030	CTIF
739	ENSG00000187837	H1-2
740	ENSG00000151276	MAGI1
741	ENSG00000143631	FLG
742	ENSG00000184602	SNN
743	ENSG00000170160	CCDC144A
744	ENSG00000284946	Pseudogene
745	ENSG00000004897	CDC27
746	ENSG00000100629	CEP128
747	ENSG00000067082	KLF6
748	ENSG00000175216	CKAP5
749	ENSG00000181467	RAP2B
750	ENSG00000183726	TMEM50A
751	ENSG00000183814	LIN9

752	ENSG00000143322	ABL2
753	ENSG00000165244	ZNF367
754	ENSG00000138778	CENPE
755	ENSG00000167747	C19orf48
756	ENSG00000171940	ZNF217
757	ENSG00000169689	CENPX
758	ENSG00000114861	FOXP1
759	ENSG00000143217	NECTIN4
760	ENSG00000137818	RPLP1
761	ENSG00000142102	PGGHG
762	ENSG00000185361	TNFAIP8L1
763	ENSG00000176148	TCP11L1
764	ENSG00000184156	KCNQ3
765	ENSG00000170759	KIF5B
766	ENSG00000124762	CDKN1A
767	ENSG00000185324	CDK10
768	ENSG00000164109	MAD2L1
769	ENSG00000167900	TK1
770	ENSG00000169813	HNRNPF
771	ENSG00000162521	RBBP4
772	ENSG00000198258	UBL5
773	ENSG00000075391	RASAL2
774	ENSG00000111445	RFC5
775	ENSG00000133454	MYO18B
776	ENSG00000124207	CSE1L
777	ENSG00000145604	SKP2
778	ENSG00000074696	HACD3
779	ENSG00000112312	GMNN
780	ENSG00000145495	MARCHF6
781	ENSG00000145425	RPS3A
782	ENSG00000188549	CCDC9B
783	ENSG00000169021	UQCRFS1
784	ENSG00000066322	ELOVL1
785	ENSG00000140264	SERF2
786	ENSG00000204435	CSNK2B
787	ENSG00000110108	TMEM109
788	ENSG00000077549	CAPZB

(2) Feature list yielded by MCFS method

Rank	Ensembl ID	Gene symbol
1	ENSG00000170312	CDK1
2	ENSG00000175063	UBE2C
3	ENSG00000197061	H4C3

4	ENSG00000123485	HJURP
5	ENSG00000131747	TOP2A
6	ENSG00000112029	FBXO5
7	ENSG00000186185	KIF18B
8	ENSG00000101447	FAM83D
9	ENSG00000146670	CDCA5
10	ENSG00000166801	FAM111A
11	ENSG00000140451	PIF1
12	ENSG00000182481	KPNA2
13	ENSG00000274997	H2AC12
14	ENSG00000169607	CKAP2L
15	ENSG00000189057	FAM111B
16	ENSG00000171320	ESCO2
17	ENSG00000276368	H2AC14
18	ENSG00000137807	KIF23
19	ENSG00000122952	ZWINT
20	ENSG00000134057	CCNB1
21	ENSG00000178999	AURKB
22	ENSG00000184661	CDCA2
23	ENSG00000162063	CCNF
24	ENSG00000171848	RRM2
25	ENSG00000117399	CDC20
26	ENSG00000115641	FHL2
27	ENSG00000173207	CKS1B
28	ENSG00000132646	PCNA
29	ENSG00000051341	POLQ
30	ENSG00000156802	ATAD2
31	ENSG00000134690	CDCA8
32	ENSG00000168298	H1-4
33	ENSG00000145386	CCNA2
34	ENSG00000121152	NCAPH
35	ENSG00000131475	VPS25
36	ENSG00000174371	EXO1
37	ENSG00000165304	MELK
38	ENSG00000229089	ANKRD20A8P
39	ENSG00000175305	CCNE2
40	ENSG00000164104	HMGB2
41	ENSG00000168496	FEN1
42	ENSG00000123975	CKS2
43	ENSG00000157456	CCNB2
44	ENSG00000170540	ARL6IP1
45	ENSG00000077152	UBE2T

46	ENSG00000148773	MKI67
47	ENSG00000214357	NEURL1B
48	ENSG00000166851	PLK1
49	ENSG00000105173	CCNE1
50	ENSG00000112118	MCM3
51	ENSG00000123473	STIL
52	ENSG00000100297	MCM5
53	ENSG00000076248	UNG
54	ENSG00000080986	NDC80
55	ENSG00000085999	RAD54L
56	ENSG00000112984	KIF20A
57	ENSG00000087586	AURKA
58	ENSG00000075218	GTSE1
59	ENSG00000185361	TNFAIP8L1
60	ENSG00000093009	CDC45
61	ENSG00000183856	IQGAP3
62	ENSG00000198331	HYLS1
63	ENSG00000088325	TPX2
64	ENSG00000124610	H1-1
65	ENSG00000128944	KNSTRN
66	ENSG00000118193	KIF14
67	ENSG00000102384	CENPI
68	ENSG00000136982	DSCC1
69	ENSG00000259848	LOC442028
70	ENSG00000094804	CDC6
71	ENSG00000121716	PILRB
72	ENSG00000111665	CDCA3
73	ENSG00000177943	MAMDC4
74	ENSG00000134222	PSRC1
75	ENSG00000164611	PTTG1
76	ENSG00000076003	MCM6
77	ENSG00000100100	PIK3IP1
78	ENSG00000143228	NUF2
79	ENSG00000104738	MCM4
80	ENSG00000138160	KIF11
81	ENSG00000137310	TCF19
82	ENSG00000109674	NEIL3
83	ENSG00000105011	ASF1B
84	ENSG00000116062	MSH6
85	ENSG00000117650	NEK2
86	ENSG00000146410	MTFR2
87	ENSG00000088986	DYNLL1

88	ENSG00000175265	GOLGA8A
89	ENSG00000125319	HROB
90	ENSG00000142945	KIF2C
91	ENSG00000139734	DIAPH3
92	ENSG00000149503	INCENP
93	ENSG00000196787	H2AC11
94	ENSG00000135451	TROAP
95	ENSG00000117724	CENPF
96	ENSG00000111247	RAD51AP1
97	ENSG00000223564	CYP4F32P
98	ENSG00000092853	CLSPN
99	ENSG00000166803	PCLAF
100	ENSG00000012048	BRCA1
101	ENSG00000184678	H2BC21
102	ENSG00000167553	TUBA1C
103	ENSG00000130844	ZNF331
104	ENSG00000144048	DUSP11
105	ENSG00000237649	KIFC1
106	ENSG00000235571	SNX18P14
107	ENSG00000163918	RFC4
108	ENSG00000127564	PKMYT1
109	ENSG00000007968	E2F2
110	ENSG00000198056	PRIM1
111	ENSG00000177426	TGIF1
112	ENSG00000169679	BUB1
113	ENSG00000165490	DDIAS
114	ENSG00000131153	GINS2
115	ENSG00000231007	CDC20P1
116	ENSG00000168078	PBK
117	ENSG00000137804	NUSAP1
118	ENSG00000196230	TUBB
119	ENSG00000073111	MCM2
120	ENSG00000101104	PABPC1L
121	ENSG00000173848	NET1
122	ENSG00000091651	ORC6
123	ENSG00000276966	H4C5
124	ENSG00000014138	POLA2
125	ENSG00000156970	BUB1B
126	ENSG00000140534	TICRR
127	ENSG00000161888	SPC24
128	ENSG00000101224	CDC25B
129	ENSG00000108106	UBE2S

130	ENSG00000135476	ESPL1
131	ENSG00000143933	CALM2
132	ENSG00000146278	PNRC1
133	ENSG00000176890	TYMS
134	ENSG00000125871	MGME1
135	ENSG00000282988	Pseudogene
136	ENSG00000116717	GADD45A
137	ENSG00000125885	MCM8
138	ENSG00000138376	BARD1
139	ENSG00000152253	SPC25
140	ENSG00000109971	HSPA8
141	ENSG00000124216	SNAI1
142	ENSG00000164109	MAD2L1
143	ENSG00000095383	TBC1D2
144	ENSG00000173456	RNF26
145	ENSG00000154839	SKA1
146	ENSG00000163950	SLBP
147	ENSG00000160298	C21orf58
148	ENSG00000092470	WDR76
149	ENSG00000116830	TTF2
150	ENSG00000112742	TTK
151	ENSG00000142102	PGGHG
152	ENSG00000188486	H2AX
153	ENSG00000113369	ARRDC3
154	ENSG00000136492	BRIP1
155	ENSG00000066279	ASPM
156	ENSG00000125898	FAM110A
157	ENSG00000072571	HMMR
158	ENSG00000101412	E2F1
159	ENSG00000126787	DLGAP5
160	ENSG00000183150	GPR19
161	ENSG00000115163	CENPA
162	ENSG00000188229	TUBB4B
163	ENSG00000213347	MXD3
164	ENSG00000169188	APEX2
165	ENSG00000143217	NECTIN4
166	ENSG00000065328	MCM10
167	ENSG00000120802	TMPO
168	ENSG00000198033	TUBA3C
169	ENSG00000081320	STK17B
170	ENSG00000165480	SKA3
171	ENSG00000167513	CDT1

172	ENSG00000185324	CDK10
173	ENSG00000100526	CDKN3
174	ENSG00000138180	CEP55
175	ENSG00000049192	ADAMTS6
176	ENSG00000138658	ZGRF1
177	ENSG00000120539	MASTL
178	ENSG00000171241	SHCBP1
179	ENSG00000100097	LGALS1
180	ENSG00000175130	MARCKSL1
181	ENSG00000175455	CCDC14
182	ENSG00000118523	CCN2
183	ENSG00000138346	DNA2
184	ENSG00000171793	CTPS1
185	ENSG00000122483	CCDC18
186	ENSG00000129534	MIS18BP1
187	ENSG00000136122	BORA
188	ENSG00000167978	SRRM2
189	ENSG00000091164	TXNL1
190	ENSG00000084733	RAB10
191	ENSG00000144354	CDCA7
192	ENSG00000127483	HP1BP3
193	ENSG00000121621	KIF18A
194	ENSG00000188985	DHFRP1
195	ENSG00000150991	UBC
196	ENSG00000106367	AP1S1
197	ENSG00000128016	ZFP36
198	ENSG00000051180	RAD51
199	ENSG00000249859	PVT1
200	ENSG00000137135	ARHGEF39
201	ENSG00000152518	ZFP36L2
202	ENSG00000149636	DSN1
203	ENSG00000170734	POLH
204	ENSG00000141232	TOB1
205	ENSG00000090889	KIF4A
206	ENSG00000138778	CENPE
207	ENSG00000126368	NR1D1
208	ENSG00000129810	SGO1
209	ENSG00000121957	GPSM2
210	ENSG00000128340	RAC2
211	ENSG00000196866	H2AC7
212	ENSG00000205352	PRR13
213	ENSG00000178719	GRINA

214	ENSG00000167900	TK1
215	ENSG00000078900	TP73
216	ENSG00000089685	BIRC5
217	ENSG00000232502	Pseudogene
218	ENSG00000168476	REEP4
219	ENSG00000163072	NOSTRIN
220	ENSG00000162073	PAQR4
221	ENSG00000143476	DTL
222	ENSG00000077514	POLD3
223	ENSG00000123219	CENPK
224	ENSG00000105355	PLIN3
225	ENSG00000144554	FANCD2
226	ENSG00000136108	CKAP2
227	ENSG00000183864	TOB2
228	ENSG00000139211	AMIGO2
229	ENSG00000115392	FANCL
230	ENSG00000013573	DDX11
231	ENSG00000163535	SGO2
232	ENSG00000277778	PGM5P2
233	ENSG00000165891	E2F7
234	ENSG00000166483	WEE1
235	ENSG00000067082	KLF6
236	ENSG00000165244	ZNF367
237	ENSG00000117139	KDM5B
238	ENSG00000118263	KLF7
239	ENSG00000102362	SYTL4
240	ENSG00000146592	CREB5
241	ENSG00000123416	TUBA1B
242	ENSG00000186767	SPIN4
243	ENSG00000167601	AXL
244	ENSG00000164754	RAD21
245	ENSG00000138182	KIF20B
246	ENSG00000115738	ID2
247	ENSG00000111859	NEDD9
248	ENSG00000175573	C11orf68
249	ENSG00000137812	KNL1
250	ENSG00000132475	H3-3B
251	ENSG00000096093	EFHC1
252	ENSG00000159335	PTMS
253	ENSG00000144227	NXPH2
254	ENSG00000145241	CENPC
255	ENSG00000198900	TOP1

256	ENSG00000221829	FANCG
257	ENSG00000178607	ERN1
258	ENSG00000263513	FAM72C
259	ENSG00000165502	RPL36AL
260	ENSG00000187051	RPS19BP1
261	ENSG00000119801	YPEL5
262	ENSG00000143322	ABL2
263	ENSG00000070950	RAD18
264	ENSG00000166002	SMCO4
265	ENSG00000284906	ARHGAP11B
266	ENSG00000138092	CENPO
267	ENSG00000129195	PIMREG
268	ENSG00000203811	H3C14
269	ENSG00000169242	EFNA1
270	ENSG00000198901	PRC1
271	ENSG00000040275	SPDL1
272	ENSG00000187741	FANCA
273	ENSG00000156463	SH3RF2
274	ENSG00000013810	TACC3
275	ENSG00000088305	DNMT3B
276	ENSG00000140264	SERF2
277	ENSG00000121211	MND1
278	ENSG00000100242	SUN2
279	ENSG00000100629	CEP128
280	ENSG00000120694	HSPH1
281	ENSG00000170779	CDCA4
282	ENSG00000169683	LRRC45
283	ENSG00000171223	JUNB
284	ENSG00000184445	KNTC1
285	ENSG00000106462	EZH2
286	ENSG00000117069	ST6GALNAC5
287	ENSG00000126945	HNRNPH2
288	ENSG00000213853	EMP2
289	ENSG00000170425	ADORA2B
290	ENSG00000131470	PSMC3IP
291	ENSG00000101773	RBBP8
292	ENSG00000122641	INHBA
293	ENSG00000188549	CCDC9B
294	ENSG00000076382	SPAG5
295	ENSG00000167325	RRM1
296	ENSG00000160957	RECQL4
297	ENSG00000075391	RASAL2

298	ENSG00000133121	STARD13
299	ENSG00000181722	ZBTB20
300	ENSG00000128294	TPST2
301	ENSG00000198826	ARHGAP11A
302	ENSG00000154473	BUB3
303	ENSG00000115946	PNO1
304	ENSG00000024526	DEPDC1
305	ENSG00000104267	CA2
306	ENSG00000103995	CEP152
307	ENSG00000119969	HELLS
308	ENSG00000162231	NXF1
309	ENSG00000172765	TMCC1
310	ENSG00000011426	ANLN
311	ENSG00000139697	SBNO1
312	ENSG00000133454	MYO18B
313	ENSG00000124766	SOX4
314	ENSG00000130299	GTPBP3
315	ENSG00000205212	CCDC144NL
316	ENSG00000117713	ARID1A
317	ENSG00000054654	SYNE2
318	ENSG00000180304	OAZ2
319	ENSG00000106211	HSPB1
320	ENSG00000115541	HSPE1
321	ENSG00000250067	YJEFN3
322	ENSG00000177602	HASPIN
323	ENSG00000123146	ADGRE5
324	ENSG00000176222	ZNF404
325	ENSG00000162783	IER5
326	ENSG00000182010	RTKN2
327	ENSG00000075618	FSCN1
328	ENSG00000178913	TAF7
329	ENSG00000160949	TONSL
330	ENSG00000095777	MYO3A
331	ENSG00000101608	MYL12A
332	ENSG00000183741	CBX6
333	ENSG00000111445	RFC5
334	ENSG00000111696	NT5DC3
335	ENSG00000185803	SLC52A2
336	ENSG00000111206	FOXM1
337	ENSG00000186193	SAPCD2
338	ENSG00000198763	ND2
339	ENSG00000134291	TMEM106C

340	ENSG00000101003	GINS1
341	ENSG00000197172	MAGEA6
342	ENSG00000100941	PNN
343	ENSG00000204382	XAGE1B
344	ENSG00000143631	FLG
345	ENSG00000180573	H2AC6
346	ENSG00000013588	GPRC5A
347	ENSG00000122565	CBX3
348	ENSG00000006327	TNFRSF12A
349	ENSG00000099194	SCD
350	ENSG00000178343	SHISA3
351	ENSG00000114315	HES1
352	ENSG00000135334	AKIRIN2
353	ENSG00000175216	CKAP5
354	ENSG00000154640	BTG3
355	ENSG00000169490	TM2D2
356	ENSG00000109805	NCAPG
357	ENSG00000184602	SNN
358	ENSG00000119333	DYNC2I2
359	ENSG00000101057	MYBL2
360	ENSG00000120334	CENPL
361	ENSG00000232388	SMIM26
362	ENSG00000130429	ARPC1B
363	ENSG00000256618	MTRNR2L1
364	ENSG00000170889	RPS9
365	ENSG00000213024	NUP62
366	ENSG00000221978	CCNL2
367	ENSG00000128951	DUT
368	ENSG00000167011	NAT16
369	ENSG00000161800	RACGAP1
370	ENSG00000168140	VASN
371	ENSG00000101945	SUV39H1
372	ENSG00000185787	MORF4L1
373	ENSG00000148677	ANKRD1
374	ENSG00000214756	CSKMT
375	ENSG00000184349	EFNA5
376	ENSG00000170264	FAM161A
377	ENSG00000171421	MRPL36
378	ENSG00000184992	BRI3BP
379	ENSG00000228716	DHFR
380	ENSG00000163808	KIF15
381	ENSG00000117450	PRDX1

382	ENSG00000214776	Pseudogene
383	ENSG00000278591	LOC124904144
384	ENSG00000133119	RFC3
385	ENSG00000127922	SEM1
386	ENSG00000168010	ATG16L2
387	ENSG00000104147	OIP5
388	ENSG00000149548	CCDC15
389	ENSG00000131652	THOC6
390	ENSG00000134375	TIMM17A
391	ENSG00000145604	SKP2
392	ENSG00000129474	AJUBA
393	ENSG00000181467	RAP2B
394	ENSG00000114346	ECT2
395	ENSG00000025770	NCAPH2
396	ENSG00000166508	MCM7
397	ENSG00000119669	IRF2BPL
398	ENSG00000164796	CSMD3
399	ENSG00000235109	ZSCAN31
400	ENSG00000171792	RHNO1
401	ENSG00000140319	SRP14
402	ENSG00000066322	ELOVL1
403	ENSG00000184260	H2AC20
404	ENSG00000056097	ZFR
405	ENSG00000276596	LOC124904139
406	ENSG00000162419	GMEB1
407	ENSG00000176148	TCP11L1
408	ENSG00000154920	EME1
409	ENSG00000113810	SMC4
410	ENSG00000135185	TMEM243
411	ENSG00000140443	IGF1R
412	ENSG00000128510	CPA4
413	ENSG00000124762	CDKN1A
414	ENSG00000275219	LOC124904143
415	ENSG00000149257	SERPINH1
416	ENSG00000124422	USP22
417	ENSG00000178074	C2orf69
418	ENSG00000006634	DBF4
419	ENSG00000178966	RMI1
420	ENSG00000139618	BRCA2
421	ENSG00000151466	SCLT1
422	ENSG00000100714	MTHFD1
423	ENSG00000117318	ID3

424	ENSG00000274062	LOC124904142
425	ENSG00000034510	TMSB10
426	ENSG00000142731	PLK4
427	ENSG00000160113	NR2F6
428	ENSG00000274862	LOC124904141
429	ENSG00000275616	LOC124904135
430	ENSG00000197956	S100A6
431	ENSG00000182541	LIMK2
432	ENSG00000075702	WDR62
433	ENSG00000204899	MZT1
434	ENSG00000158373	H2BC5
435	ENSG00000172667	ZMAT3
436	ENSG00000118655	DCLRE1B
437	ENSG00000278259	MYO19
438	ENSG00000108518	PFN1
439	ENSG00000130202	NECTIN2
440	ENSG00000170545	SMAGP
441	ENSG00000102804	TSC22D1
442	ENSG00000278048	LOC124904138
443	ENSG00000130669	PAK4
444	ENSG00000145495	MARCHF6
445	ENSG00000132341	RAN
446	ENSG00000204435	CSNK2B
447	ENSG00000169813	HNRNPF
448	ENSG00000170759	KIF5B
449	ENSG00000168282	MGAT2
450	ENSG00000213551	DNAJC9
451	ENSG00000129173	E2F8
452	ENSG00000151503	NCAPD3
453	ENSG00000146263	MMS22L
454	ENSG00000089693	MLF2
455	ENSG00000010292	NCAPD2
456	ENSG00000186871	ERCC6L
457	ENSG00000107816	LZTS2
458	ENSG00000198554	WDHD1
459	ENSG00000145779	TNFAIP8
460	ENSG00000273709	LOC124904136
461	ENSG00000106305	AIMP2
462	ENSG00000183726	TMEM50A
463	ENSG00000079616	KIF22
464	ENSG00000132780	NASP
465	ENSG00000065548	ZC3H15

466	ENSG00000178974	FBXO34
467	ENSG00000187266	EPOR
468	ENSG00000049541	RFC2
469	ENSG00000186834	HEXIM1
470	ENSG00000176208	ATAD5
471	ENSG00000124207	CSE1L
472	ENSG00000274432	LOC124904137
473	ENSG00000215252	GOLGA8B
474	ENSG00000136997	MYC
475	ENSG00000185650	ZFP36L1
476	ENSG00000196550	FAM72A
477	ENSG00000255152	MSH5-SAPCD1
478	ENSG00000139354	GAS2L3
479	ENSG00000111321	LTBR
480	ENSG00000146555	SDK1
481	ENSG00000162607	USP1
482	ENSG00000117143	UAP1
483	ENSG00000122644	ARL4A
484	ENSG00000171867	PRNP
485	ENSG00000151725	CENPU
486	ENSG00000274585	RNU2-1
487	ENSG00000162772	ATF3
488	ENSG00000285730	Pseudogene
489	ENSG00000171490	RSL1D1
490	ENSG00000198258	UBL5
491	ENSG00000112312	GMNN
492	ENSG00000109099	PMP22
493	ENSG00000143621	ILF2
494	ENSG00000026103	FAS
495	ENSG00000265790	RNASEH1P1
496	ENSG00000164211	STARD4
497	ENSG00000167747	C19orf48
498	ENSG00000135046	ANXA1
499	ENSG00000131446	MGAT1
500	ENSG00000111788	Pseudogene
501	ENSG00000277903	LOC124904140
502	ENSG00000042088	TDP1
503	ENSG00000172432	GTPBP2
504	ENSG00000183337	BCOR
505	ENSG00000175606	TMEM70
506	ENSG00000144395	CCDC150
507	ENSG00000153048	CARHSP1

508	ENSG00000115875	SRSF7
509	ENSG00000149798	CDC42EP2
510	ENSG00000198890	PRMT6
511	ENSG00000276043	UHRF1
512	ENSG00000197299	BLM
513	ENSG00000180611	MB21D2
514	ENSG00000138385	SSB
515	ENSG00000128965	CHAC1
516	ENSG00000124172	ATP5F1E
517	ENSG00000091436	MAP3K20
518	ENSG00000196235	SUPT5H
519	ENSG00000142765	SYTL1
520	ENSG00000102977	ACD
521	ENSG00000031691	CENPQ
522	ENSG00000138175	ARL3
523	ENSG00000179958	DCTPP1
524	ENSG00000158402	CDC25C
525	ENSG00000131435	PDLIM4
526	ENSG00000168061	SAC3D1
527	ENSG00000174136	RGMB
528	ENSG00000166004	CEP295
529	ENSG00000078401	EDN1
530	ENSG00000143761	ARF1
531	ENSG00000149639	SOGA1
532	ENSG00000137727	ARHGAP20
533	ENSG00000075131	TIPIN
534	ENSG00000162222	TTC9C
535	ENSG00000214944	ARHGEF28
536	ENSG00000105821	DNAJC2
537	ENSG00000077684	JADE1
538	ENSG00000112972	HMGCS1
539	ENSG00000164251	F2RL1
540	ENSG00000198680	TUSC1
541	ENSG00000196531	NACA
542	ENSG00000162614	NEXN
543	ENSG00000177084	POLE
544	ENSG00000109255	NMU
545	ENSG00000144381	HSPD1
546	ENSG00000164924	YWHAZ
547	ENSG00000198088	NUP62CL
548	ENSG00000198355	PIM3
549	ENSG00000171552	BCL2L1

550	ENSG00000186638	KIF24
551	ENSG00000153395	LPCAT1
552	ENSG00000100632	ERH
553	ENSG00000120129	DUSP1
554	ENSG00000204389	HSPA1A
555	ENSG00000173846	PLK3
556	ENSG00000129484	PARP2
557	ENSG00000165886	UBTD1
558	ENSG00000165501	LRR1
559	ENSG00000169021	UQCRFS1
560	ENSG00000172893	DHCR7
561	ENSG00000214826	DDX12P
562	ENSG00000105372	RPS19
563	ENSG00000068028	RASSF1
564	ENSG00000189241	TSPYL1
565	ENSG00000126226	PCID2
566	ENSG00000033030	ZCCHC8
567	ENSG00000109321	AREG
568	ENSG00000134030	CTIF
569	ENSG00000166925	TSC22D4
570	ENSG00000132823	OSER1
571	ENSG00000183255	PTTG1IP
572	ENSG00000206625	RNU6-1
573	ENSG00000065911	MTHFD2
574	ENSG00000118971	CCND2
575	ENSG00000167552	TUBA1A
576	ENSG00000169689	CENPX
577	ENSG00000182752	PAPPA
578	ENSG00000074266	EED
579	ENSG00000127948	POR
580	ENSG00000137936	BCAR3
581	ENSG00000123737	EXOSC9
582	ENSG00000207357	RNU6-2
583	ENSG00000144136	SLC20A1
584	ENSG00000075426	FOSL2
585	ENSG00000166441	RPL27A
586	ENSG00000163507	CIP2A
587	ENSG00000109062	SLC9A3R1
588	ENSG00000123933	MXD4
589	ENSG00000183963	SMTN
590	ENSG00000145425	RPS3A
591	ENSG00000115687	PASK

592	ENSG00000072864	NDE1
593	ENSG00000177595	PIDD1
594	ENSG00000206899	RNU6-36P
595	ENSG00000165181	SHOC1
596	ENSG00000120053	GOT1
597	ENSG00000140400	MAN2C1
598	ENSG00000101639	CEP192
599	ENSG00000158246	TENT5B
600	ENSG00000077549	CAPZB
601	ENSG00000079482	OPHN1
602	ENSG00000009954	BAZ1B
603	ENSG00000082438	COBLL1
604	ENSG00000105516	DBP
605	ENSG00000174738	NR1D2
606	ENSG00000176225	RTTN
607	ENSG00000173559	NABP1
608	ENSG00000188610	FAM72B
609	ENSG00000131462	TUBG1
610	ENSG00000184047	DIABLO
611	ENSG00000001460	STPG1
612	ENSG00000104635	SLC39A14
613	ENSG00000160789	LMNA
614	ENSG00000121388	Pseudogene
615	ENSG00000155506	LARP1
616	ENSG00000137497	NUMA1
617	ENSG00000166965	RCCD1
618	ENSG00000116679	IVNS1ABP
619	ENSG00000057019	DCBLD2
620	ENSG00000177879	AP3S1
621	ENSG00000176105	YES1
622	ENSG00000143878	RHOB
623	ENSG00000117616	RSRP1
624	ENSG00000115306	SPTBN1
625	ENSG00000112576	CCND3
626	ENSG00000104783	KCNN4
627	ENSG00000225630	MTND2P28
628	ENSG00000087077	TRIP6
629	ENSG00000153233	PTPRR
630	ENSG00000173068	BNC2
631	ENSG00000085840	ORC1
632	ENSG00000179094	PER1
633	ENSG00000276180	H4C9

634	ENSG00000101868	POLA1
635	ENSG00000170160	CCDC144A
636	ENSG00000284946	Pseudogene
637	ENSG00000105325	FZR1
638	ENSG00000206965	RNU6-5P
639	ENSG00000104671	DCTN6
640	ENSG00000096384	HSP90AB1
641	ENSG00000116133	DHCR24
642	ENSG00000183814	LIN9
643	ENSG00000146425	DYNLT1
644	ENSG00000197256	KANK2
645	ENSG00000152117	SMPD4BP
646	ENSG00000118680	MYL12B
647	ENSG00000188529	SRSF10
648	ENSG00000182197	EXT1
649	ENSG00000140350	ANP32A
650	ENSG00000130816	DNMT1
651	ENSG00000092140	G2E3
652	ENSG00000112759	SLC29A1
653	ENSG00000124635	H2BC11
654	ENSG00000147155	EBP
655	ENSG00000172244	C5orf34
656	ENSG00000140332	TLE3
657	ENSG00000129355	CDKN2D
658	ENSG00000169252	ADRB2
659	ENSG00000189337	KAZN
660	ENSG00000183763	TRAIP
661	ENSG00000183955	KMT5A
662	ENSG00000112081	SRSF3
663	ENSG00000104064	GABPB1
664	ENSG00000207507	RNU6-9
665	ENSG00000168916	ZNF608
666	ENSG00000270882	H4C14
667	ENSG00000111276	CDKN1B
668	ENSG00000119771	KLHL29
669	ENSG00000070882	OSBPL3
670	ENSG00000162521	RBBP4
671	ENSG00000137818	RPLP1
672	ENSG00000165732	DDX21
673	ENSG00000283269	Pseudogene
674	ENSG00000161011	SQSTM1
675	ENSG00000142541	RPL13A

676	ENSG00000186283	TOR3A
677	ENSG00000206932	RNU6-4P
678	ENSG00000100479	POLE2
679	ENSG00000146834	MEPCE
680	ENSG00000207041	RNU6-3P
681	ENSG00000162062	TEDC2
682	ENSG00000142871	CCN1
683	ENSG00000167565	SERTAD3
684	ENSG00000171940	ZNF217
685	ENSG00000162929	SANBR
686	ENSG00000148248	SURF4
687	ENSG00000100401	RANGAP1
688	ENSG00000136436	CALCOCO2
689	ENSG00000147813	NAPRT
690	ENSG00000189060	H1-0
691	ENSG00000277161	PIGW
692	ENSG00000187837	H1-2
693	ENSG00000149483	TMEM138
694	ENSG00000004897	CDC27
695	ENSG00000152359	POC5
696	ENSG00000157191	NECAP2
697	ENSG00000204370	SDHD
698	ENSG00000163655	GMPS
699	ENSG00000160753	RUSC1
700	ENSG00000142149	HUNK
701	ENSG00000130695	CEP85
702	ENSG00000170852	KBTBD2
703	ENSG00000132485	ZRANB2
704	ENSG00000135211	TMEM60
705	ENSG00000108055	SMC3
706	ENSG00000100519	PSMC6
707	ENSG00000116667	C1orf21
708	ENSG00000115159	GPD2
709	ENSG00000167670	CHAF1A
710	ENSG00000087086	FTL
711	ENSG00000127586	CHTF18
712	ENSG00000101574	METTL4
713	ENSG00000176974	SHMT1
714	ENSG00000121741	ZMYM2
715	ENSG00000166949	SMAD3
716	ENSG00000105856	HBP1
717	ENSG00000145526	CDH18

718	ENSG00000164087	POC1A
719	ENSG00000095002	MSH2
720	ENSG00000130921	MTRFR
721	ENSG00000204580	DDR1
722	ENSG00000068489	PRR11
723	ENSG00000185567	AHNAK2
724	ENSG00000006625	GGCT
725	ENSG00000162433	AK4
726	ENSG00000061337	LZTS1
727	ENSG00000070761	CFAP20
728	ENSG00000163002	NUP35
729	ENSG00000175592	FOSL1
730	ENSG00000187231	SESTD1
731	ENSG00000113456	RAD1
732	ENSG00000131737	KRT34
733	ENSG00000136699	SMPD4
734	ENSG00000161692	DBF4B
735	ENSG00000092969	TGFB2
736	ENSG00000114861	FOXP1
737	ENSG00000186472	PCLO
738	ENSG00000118181	RPS25
739	ENSG00000113569	NUP155
740	ENSG00000205560	CPT1B
741	ENSG00000143942	CHAC2
742	ENSG00000176014	TUBB6
743	ENSG00000158201	ABHD3
744	ENSG00000103540	CCP110
745	ENSG00000112414	ADGRG6
746	ENSG00000110108	TMEM109
747	ENSG00000122566	HNRNPA2B1
748	ENSG00000096433	ITPR3
749	ENSG00000138496	PARP9
750	ENSG00000166140	ZFYVE19
751	ENSG00000119397	CNTRL
752	ENSG00000177917	ARL6IP6
753	ENSG00000148019	CEP78
754	ENSG00000104047	DTWD1
755	ENSG00000161980	POLR3K
756	ENSG00000182378	PLCXD1
757	ENSG00000128245	YWHAH
758	ENSG00000143367	TUFT1
759	ENSG00000143815	LBR

760	ENSG00000171105	INSR
761	ENSG00000074696	HACD3
762	ENSG00000101138	CSTF1
763	ENSG00000204388	HSPA1B
764	ENSG00000172167	MTBP
765	ENSG00000164985	PSIP1
766	ENSG00000213186	TRIM59
767	ENSG00000134802	SLC43A3
768	ENSG00000181544	FANCB
769	ENSG00000164649	CDCA7L
770	ENSG00000099810	MTAP
771	ENSG00000100162	CENPM
772	ENSG00000103121	CMC2
773	ENSG00000127337	YEATS4
774	ENSG00000165494	PCF11
775	ENSG00000131351	HAUS8
776	ENSG00000105270	CLIP3
777	ENSG00000178295	GEN1
778	ENSG00000151692	RNF144A
779	ENSG00000081019	RSBN1
780	ENSG00000188312	CENPP
781	ENSG00000105991	HOXA1
782	ENSG00000068885	IFT80
783	ENSG00000128708	HAT1
784	ENSG00000178105	DDX10
785	ENSG00000184156	KCNQ3
786	ENSG00000151276	MAGI1
787	ENSG00000217555	CKLF
788	ENSG00000109861	CTSC

(3) Feature list yielded by SHAP by lightGBM

Rank	Ensembl ID	Gene symbol
1	ENSG00000170312	CDK1
2	ENSG00000197061	H4C3
3	ENSG00000131652	THOC6
4	ENSG00000140451	PIF1
5	ENSG00000175063	UBE2C
6	ENSG00000229089	ANKRD20A8P
7	ENSG00000150991	UBC
8	ENSG00000189057	FAM111B
9	ENSG00000105173	CCNE1
10	ENSG00000132646	PCNA

11	ENSG00000101447	FAM83D
12	ENSG00000182481	KPNA2
13	ENSG00000012048	BRCA1
14	ENSG00000259848	LOC442028
15	ENSG00000274997	H2AC12
16	ENSG00000075426	FOSL2
17	ENSG00000118680	MYL12B
18	ENSG00000014138	POLA2
19	ENSG00000112029	FBXO5
20	ENSG00000131747	TOP2A
21	ENSG00000116830	TTF2
22	ENSG00000117399	CDC20
23	ENSG00000170425	ADORA2B
24	ENSG00000140319	SRP14
25	ENSG00000134057	CCNB1
26	ENSG00000119801	YPEL5
27	ENSG00000223564	CYP4F32P
28	ENSG00000173207	CKS1B
29	ENSG00000100297	MCM5
30	ENSG00000276368	H2AC14
31	ENSG00000167601	AXL
32	ENSG00000155506	LARP1
33	ENSG00000189241	TSPYL1
34	ENSG00000117450	PRDX1
35	ENSG00000080986	NDC80
36	ENSG00000175305	CCNE2
37	ENSG00000115641	FHL2
38	ENSG00000177426	TGIF1
39	ENSG00000169021	UQCRFS1
40	ENSG00000127948	POR
41	ENSG00000186472	PCLO
42	ENSG00000171552	BCL2L1
43	ENSG00000104267	CA2
44	ENSG00000143933	CALM2
45	ENSG00000277161	PIGW
46	ENSG00000171848	RRM2
47	ENSG00000145425	RPS3A
48	ENSG00000087586	AURKA
49	ENSG00000167978	SRRM2
50	ENSG00000131351	HAUS8
51	ENSG00000115946	PNO1
52	ENSG00000105355	PLIN3

53	ENSG00000167552	TUBA1A
54	ENSG00000126368	NR1D1
55	ENSG00000122952	ZWINT
56	ENSG00000105011	ASF1B
57	ENSG00000054654	SYNE2
58	ENSG00000170540	ARL6IP1
59	ENSG00000164924	YWHAZ
60	ENSG00000144354	CDCA7
61	ENSG00000140264	SERF2
62	ENSG00000117724	CENPF
63	ENSG00000197956	S100A6
64	ENSG00000158246	TENT5B
65	ENSG00000129474	AJUBA
66	ENSG00000101003	GINS1
67	ENSG00000146670	CDCA5
68	ENSG00000145386	CCNA2
69	ENSG00000007968	E2F2
70	ENSG00000188229	TUBB4B
71	ENSG00000171421	MRPL36
72	ENSG00000087077	TRIP6
73	ENSG00000076248	UNG
74	ENSG00000167513	CDT1
75	ENSG00000114346	ECT2
76	ENSG00000092853	CLSPN
77	ENSG00000178913	TAF7
78	ENSG00000160957	RECQL4
79	ENSG00000078401	EDN1
80	ENSG00000187051	RPS19BP1
81	ENSG00000176890	TYMS
82	ENSG00000168476	REEP4
83	ENSG00000161980	POLR3K
84	ENSG00000149548	CCDC15
85	ENSG00000146263	MMS22L
86	ENSG00000128340	RAC2
87	ENSG00000127564	PKMYT1
88	ENSG00000087086	FTL
89	ENSG00000205352	PRR13
90	ENSG00000186767	SPIN4
91	ENSG00000183726	TMEM50A
92	ENSG00000174738	NR1D2
93	ENSG00000169683	LRRC45
94	ENSG00000130429	ARPC1B

95	ENSG00000124610	H1-1
96	ENSG00000124172	ATP5F1E
97	ENSG00000196866	H2AC7
98	ENSG00000185361	TNFAIP8L1
99	ENSG00000175265	GOLGA8A
100	ENSG00000166002	SMCO4
101	ENSG00000163918	RFC4
102	ENSG00000138385	SSB
103	ENSG00000123485	HJURP
104	ENSG00000024526	DEPDC1
105	ENSG00000013573	DDX11
106	ENSG00000011426	ANLN
107	ENSG00000177943	MAMDC4
108	ENSG00000173456	RNF26
109	ENSG00000152117	SMPD4BP
110	ENSG00000108518	PFN1
111	ENSG00000092969	TGFB2
112	ENSG00000051180	RAD51
113	ENSG00000232388	SMIM26
114	ENSG00000178719	GRINA
115	ENSG00000166965	RCCD1
116	ENSG00000136997	MYC
117	ENSG00000136982	DSCC1
118	ENSG00000129534	MIS18BP1
119	ENSG00000120129	DUSP1
120	ENSG00000109062	SLC9A3R1
121	ENSG00000221829	FANCG
122	ENSG00000214826	DDX12P
123	ENSG00000204370	SDHD
124	ENSG00000196531	NACA
125	ENSG00000184992	BRI3BP
126	ENSG00000184349	EFNA5
127	ENSG00000175573	C11orf68
128	ENSG00000175455	CCDC14
129	ENSG00000165480	SKA3
130	ENSG00000116717	GADD45A
131	ENSG00000116133	DHCR24
132	ENSG00000112972	HMGCS1
133	ENSG00000104783	KCNN4
134	ENSG00000091651	ORC6
135	ENSG00000076003	MCM6
136	ENSG00000263513	FAM72C

137	ENSG00000198033	TUBA3C
138	ENSG00000186283	TOR3A
139	ENSG00000179958	DCTPP1
140	ENSG00000172893	DHCR7
141	ENSG00000166801	FAM111A
142	ENSG00000166140	ZFYVE19
143	ENSG00000156802	ATAD2
144	ENSG00000148773	MKI67
145	ENSG00000143815	LBR
146	ENSG00000137807	KIF23
147	ENSG00000131470	PSMC3IP
148	ENSG00000124766	SOX4
149	ENSG00000122641	INHBA
150	ENSG00000103995	CEP152
151	ENSG00000095002	MSH2
152	ENSG00000231007	CDC20P1
153	ENSG00000213024	NUP62
154	ENSG00000205560	CPT1B
155	ENSG00000198258	UBL5
156	ENSG00000185567	AHNAK2
157	ENSG00000183963	SMTN
158	ENSG00000175216	CKAP5
159	ENSG00000168298	H1-4
160	ENSG00000168282	MGAT2
161	ENSG00000166949	SMAD3
162	ENSG00000166851	PLK1
163	ENSG00000166803	PCLAF
164	ENSG00000135185	TMEM243
165	ENSG00000134690	CDCA8
166	ENSG00000104635	SLC39A14
167	ENSG00000100526	CDKN3
168	ENSG00000001460	STPG1
169	ENSG00000183814	LIN9
170	ENSG00000182541	LIMK2
171	ENSG00000180573	H2AC6
172	ENSG00000176105	YES1
173	ENSG00000168496	FEN1
174	ENSG00000166483	WEE1
175	ENSG00000153048	CARHSP1
176	ENSG00000145779	TNFAIP8
177	ENSG00000132823	OSER1
178	ENSG00000128965	CHAC1

179	ENSG00000121716	PILRB
180	ENSG00000117143	UAP1
181	ENSG00000115541	HSPE1
182	ENSG00000096093	EFHC1
183	ENSG00000065328	MCM10
184	ENSG00000282988	Pseudogene
185	ENSG00000232502	Pseudogene
186	ENSG00000214944	ARHGEF28
187	ENSG00000214776	Pseudogene
188	ENSG00000184047	DIABLO
189	ENSG00000176222	ZNF404
190	ENSG00000169813	HNRNPF
191	ENSG00000169689	CENPX
192	ENSG00000169188	APEX2
193	ENSG00000163655	GMPS
194	ENSG00000149798	CDC42EP2
195	ENSG00000136436	CALCOCO2
196	ENSG00000135046	ANXA1
197	ENSG00000128016	ZFP36
198	ENSG00000123416	TUBA1B
199	ENSG00000120053	GOT1
200	ENSG00000109861	CTSC
201	ENSG00000109099	PMP22
202	ENSG00000106367	AP1S1
203	ENSG00000101608	MYL12A
204	ENSG00000100629	CEP128
205	ENSG00000100401	RANGAP1
206	ENSG00000085999	RAD54L
207	ENSG00000196235	SUPT5H
208	ENSG00000187266	EPOR
209	ENSG00000186871	ERCC6L
210	ENSG00000171490	RSL1D1
211	ENSG00000162419	GMEB1
212	ENSG00000162073	PAQR4
213	ENSG00000157456	CCNB2
214	ENSG00000152253	SPC25
215	ENSG00000149636	DSN1
216	ENSG00000146592	CREB5
217	ENSG00000143878	RHOB
218	ENSG00000126226	PCID2
219	ENSG00000125898	FAM110A
220	ENSG00000123975	CKS2

221	ENSG00000123146	ADGRE5
222	ENSG00000121388	Pseudogene
223	ENSG00000117616	RSRP1
224	ENSG00000115306	SPTBN1
225	ENSG00000112312	GMNN
226	ENSG00000110108	TMEM109
227	ENSG00000106462	EZH2
228	ENSG00000101773	RBBP8
229	ENSG00000100941	PNN
230	ENSG00000100100	PIK3IP1
231	ENSG00000093009	CDC45
232	ENSG00000075131	TIPIN
233	ENSG00000049192	ADAMTS6
234	ENSG00000034510	TMSB10
235	ENSG00000025770	NCAPH2
236	ENSG00000010292	NCAPD2
237	ENSG00000006327	TNFRSF12A
238	ENSG00000273709	LOC124904136
239	ENSG00000213186	TRIM59
240	ENSG00000204389	HSPA1A
241	ENSG00000198331	HYLS1
242	ENSG00000197256	KANK2
243	ENSG00000196787	H2AC11
244	ENSG00000186834	HEXIM1
245	ENSG00000181467	RAP2B
246	ENSG00000178607	ERN1
247	ENSG00000171105	INSR
248	ENSG00000167900	TK1
249	ENSG00000167670	CHAF1A
250	ENSG00000164251	F2RL1
251	ENSG00000152359	POC5
252	ENSG00000143476	DTL
253	ENSG00000142945	KIF2C
254	ENSG00000141232	TOB1
255	ENSG00000137310	TCF19
256	ENSG00000135451	TROAP
257	ENSG00000135334	AKIRIN2
258	ENSG00000131737	KRT34
259	ENSG00000128944	KNSTRN
260	ENSG00000124635	H2BC11
261	ENSG00000124422	USP22
262	ENSG00000124207	CSE1L

263	ENSG00000122565	CBX3
264	ENSG00000121211	MND1
265	ENSG00000119969	HELLS
266	ENSG00000117713	ARID1A
267	ENSG00000117139	KDM5B
268	ENSG00000114861	FOXP1
269	ENSG00000112742	TTK
270	ENSG00000112081	SRSF3
271	ENSG00000104738	MCM4
272	ENSG00000077514	POLD3
273	ENSG00000073111	MCM2
274	ENSG00000049541	RFC2
275	ENSG00000276043	UHRF1
276	ENSG00000206625	RNU6-1
277	ENSG00000196230	TUBB
278	ENSG00000188549	CCDC9B
279	ENSG00000183955	KMT5A
280	ENSG00000183150	GPR19
281	ENSG00000176148	TCP11L1
282	ENSG00000171223	JUNB
283	ENSG00000170779	CDCA4
284	ENSG00000170160	CCDC144A
285	ENSG00000169242	EFNA1
286	ENSG00000167565	SERTAD3
287	ENSG00000165501	LRR1
288	ENSG00000165304	MELK
289	ENSG00000165244	ZNF367
290	ENSG00000164611	PTTG1
291	ENSG00000162062	TEDC2
292	ENSG00000160789	LMNA
293	ENSG00000159335	PTMS
294	ENSG00000156970	BUB1B
295	ENSG00000152518	ZFP36L2
296	ENSG00000148248	SURF4
297	ENSG00000144554	FANCD2
298	ENSG00000144136	SLC20A1
299	ENSG00000143217	NECTIN4
300	ENSG00000139618	BRCA2
301	ENSG00000134375	TIMM17A
302	ENSG00000131475	VPS25
303	ENSG00000130816	DNMT1
304	ENSG00000129173	E2F8

305	ENSG00000126945	HNRNPH2
306	ENSG00000120539	MASTL
307	ENSG00000116679	IVNS1ABP
308	ENSG00000116667	C1orf21
309	ENSG00000115687	PASK
310	ENSG00000113456	RAD1
311	ENSG00000113369	ARRDC3
312	ENSG00000112118	MCM3
313	ENSG00000109971	HSPA8
314	ENSG00000109255	NMU
315	ENSG00000105516	DBP
316	ENSG00000104064	GABPB1
317	ENSG00000101224	CDC25B
318	ENSG00000099194	SCD
319	ENSG00000088986	DYNLL1
320	ENSG00000085840	ORC1
321	ENSG00000070761	CFAP20
322	ENSG00000013588	GPRC5A
323	ENSG00000228716	DHFR
324	ENSG00000198763	ND2
325	ENSG00000198680	TUSC1
326	ENSG00000198056	PRIM1
327	ENSG00000188985	DHFRP1
328	ENSG00000187741	FANCA
329	ENSG00000186185	KIF18B
330	ENSG00000183255	PTTG1IP
331	ENSG00000182752	PAPPA
332	ENSG00000180304	OAZ2
333	ENSG00000175130	MARCKSL1
334	ENSG00000172244	C5orf34
335	ENSG00000171867	PRNP
336	ENSG00000170545	SMAGP
337	ENSG00000164754	RAD21
338	ENSG00000162614	NEXN
339	ENSG00000162607	USP1
340	ENSG00000162433	AK4
341	ENSG00000158201	ABHD3
342	ENSG00000149257	SERPINH1
343	ENSG00000147155	EBP
344	ENSG00000146425	DYNLT1
345	ENSG00000144381	HSPD1
346	ENSG00000142765	SYTL1

347	ENSG00000142541	RPL13A
348	ENSG00000139211	AMIGO2
349	ENSG00000138496	PARP9
350	ENSG00000137936	BCAR3
351	ENSG00000137727	ARHGAP20
352	ENSG00000135211	TMEM60
353	ENSG00000131153	GINS2
354	ENSG00000129195	PIMREG
355	ENSG00000128951	DUT
356	ENSG00000118971	CCND2
357	ENSG00000116062	MSH6
358	ENSG00000113810	SMC4
359	ENSG00000111665	CDCA3
360	ENSG00000111276	CDKN1B
361	ENSG00000111247	RAD51AP1
362	ENSG00000109674	NEIL3
363	ENSG00000109321	AREG
364	ENSG00000104047	DTWD1
365	ENSG00000102804	TSC22D1
366	ENSG00000079482	OPHN1
367	ENSG00000075391	RASAL2
368	ENSG00000265790	RNASEH1P1
369	ENSG00000256618	MTRNR2L1
370	ENSG00000249859	PVT1
371	ENSG00000197299	BLM
372	ENSG00000185787	MORF4L1
373	ENSG00000185324	CDK10
374	ENSG00000184678	H2BC21
375	ENSG00000182197	EXT1
376	ENSG00000181722	ZBTB20
377	ENSG00000178074	C2orf69
378	ENSG00000177595	PIDD1
379	ENSG00000173846	PLK3
380	ENSG00000171320	ESCO2
381	ENSG00000170759	KIF5B
382	ENSG00000165181	SHOC1
383	ENSG00000164796	CSMD3
384	ENSG00000163002	NUP35
385	ENSG00000162222	TTC9C
386	ENSG00000160949	TONSL
387	ENSG00000157191	NECAP2
388	ENSG00000148019	CEP78

389	ENSG00000147813	NAPRT
390	ENSG00000146410	MTFR2
391	ENSG00000140534	TICRR
392	ENSG00000140400	MAN2C1
393	ENSG00000138180	CEP55
394	ENSG00000138175	ARL3
395	ENSG00000138092	CENPO
396	ENSG00000134802	SLC43A3
397	ENSG00000134030	CTIF
398	ENSG00000132475	H3-3B
399	ENSG00000131435	PDLIM4
400	ENSG00000130921	MTRFR
401	ENSG00000130844	ZNF331
402	ENSG00000130695	CEP85
403	ENSG00000130669	PAK4
404	ENSG00000130202	NECTIN2
405	ENSG00000128708	HAT1
406	ENSG00000128294	TPST2
407	ENSG00000119669	IRF2BPL
408	ENSG00000119397	CNTRL
409	ENSG00000118523	CCN2
410	ENSG00000111859	NEDD9
411	ENSG00000107816	LZTS2
412	ENSG00000102977	ACD
413	ENSG00000101057	MYBL2
414	ENSG00000100632	ERH
415	ENSG00000095383	TBC1D2
416	ENSG00000092470	WDR76
417	ENSG00000089685	BIRC5
418	ENSG00000081320	STK17B
419	ENSG00000077152	UBE2T
420	ENSG00000074696	HACD3
421	ENSG00000026103	FAS
422	ENSG00000277778	PGM5P2
423	ENSG00000225630	MTND2P28
424	ENSG00000215252	GOLGA8B
425	ENSG00000214756	CSKMT
426	ENSG00000213347	MXD3
427	ENSG00000204382	XAGE1B
428	ENSG00000198901	PRC1
429	ENSG00000198890	PRMT6
430	ENSG00000188486	H2AX

431	ENSG00000185803	SLC52A2
432	ENSG00000184661	CDCA2
433	ENSG00000184445	KNTC1
434	ENSG00000183763	TRAIP
435	ENSG00000183337	BCOR
436	ENSG00000179094	PER1
437	ENSG00000177602	HASPIN
438	ENSG00000177084	POLE
439	ENSG00000174136	RGMB
440	ENSG00000172432	GTPBP2
441	ENSG00000170889	RPS9
442	ENSG00000170264	FAM161A
443	ENSG00000169607	CKAP2L
444	ENSG00000168140	VASN
445	ENSG00000167553	TUBA1C
446	ENSG00000167325	RRM1
447	ENSG00000166508	MCM7
448	ENSG00000164649	CDCA7L
449	ENSG00000162063	CCNF
450	ENSG00000160113	NR2F6
451	ENSG00000151503	NCAPD3
452	ENSG00000148677	ANKRD1
453	ENSG00000146555	SDK1
454	ENSG00000145241	CENPC
455	ENSG00000144227	NXPH2
456	ENSG00000143631	FLG
457	ENSG00000143621	ILF2
458	ENSG00000143367	TUFT1
459	ENSG00000142871	CCN1
460	ENSG00000140350	ANP32A
461	ENSG00000138778	CENPE
462	ENSG00000137804	NUSAP1
463	ENSG00000136699	SMPD4
464	ENSG00000136122	BORA
465	ENSG00000132341	RAN
466	ENSG00000128245	YWHAH
467	ENSG00000127922	SEM1
468	ENSG00000127483	HP1BP3
469	ENSG00000125885	MCM8
470	ENSG00000123473	STIL
471	ENSG00000123219	CENPK
472	ENSG00000122483	CCDC18

473	ENSG00000121152	NCAPH
474	ENSG00000120802	TMPO
475	ENSG00000120334	CENPL
476	ENSG00000117650	NEK2
477	ENSG00000115875	SRSF7
478	ENSG00000115392	FANCL
479	ENSG00000111445	RFC5
480	ENSG00000111321	LTBR
481	ENSG00000108106	UBE2S
482	ENSG00000108055	SMC3
483	ENSG00000106305	AIMP2
484	ENSG00000102384	CENPI
485	ENSG00000100162	CENPM
486	ENSG00000096433	ITPR3
487	ENSG00000091436	MAP3K20
488	ENSG00000091164	TXNL1
489	ENSG00000090889	KIF4A
490	ENSG00000084733	RAB10
491	ENSG00000082438	COBLL1
492	ENSG00000079616	KIF22
493	ENSG00000072571	HMMR
494	ENSG00000056097	ZFR
495	ENSG00000009954	BAZ1B
496	ENSG00000276180	H4C9
497	ENSG00000270882	H4C14
498	ENSG00000250067	YJEFN3
499	ENSG00000217555	CKLF
500	ENSG00000213853	EMP2
501	ENSG00000204899	MZT1
502	ENSG00000204388	HSPA1B
503	ENSG00000185650	ZFP36L1
504	ENSG00000182378	PLCXD1
505	ENSG00000182010	RTKN2
506	ENSG00000176974	SHMT1
507	ENSG00000176014	TUBB6
508	ENSG00000173068	BNC2
509	ENSG00000172167	MTBP
510	ENSG00000171241	SHCBP1
511	ENSG00000169679	BUB1
512	ENSG00000169252	ADRB2
513	ENSG00000167747	C19orf48
514	ENSG00000165732	DDX21

515	ENSG00000164104	HMGB2
516	ENSG00000163950	SLBP
517	ENSG00000162772	ATF3
518	ENSG00000162231	NXF1
519	ENSG00000160753	RUSC1
520	ENSG00000154839	SKA1
521	ENSG00000153395	LPCAT1
522	ENSG00000153233	PTPRR
523	ENSG00000149483	TMEM138
524	ENSG00000145604	SKP2
525	ENSG00000145495	MARCHF6
526	ENSG00000143761	ARF1
527	ENSG00000142102	PGGHG
528	ENSG00000139734	DIAPH3
529	ENSG00000139697	SBNO1
530	ENSG00000138346	DNA2
531	ENSG00000137818	RPLP1
532	ENSG00000137497	NUMA1
533	ENSG00000135476	ESPL1
534	ENSG00000131446	MGAT1
535	ENSG00000129484	PARP2
536	ENSG00000129355	CDKN2D
537	ENSG00000126787	DLGAP5
538	ENSG00000117318	ID3
539	ENSG00000115738	ID2
540	ENSG00000114315	HES1
541	ENSG00000112984	KIF20A
542	ENSG00000112576	CCND3
543	ENSG00000112414	ADGRG6
544	ENSG00000111788	Pseudogene
545	ENSG00000105372	RPS19
546	ENSG00000103121	CMC2
547	ENSG00000102362	SYTL4
548	ENSG00000101639	CEP192
549	ENSG00000101412	E2F1
550	ENSG00000100479	POLE2
551	ENSG00000100097	LGALS1
552	ENSG00000096384	HSP90AB1
553	ENSG00000094804	CDC6
554	ENSG00000077684	JADE1
555	ENSG00000075218	GTSE1
556	ENSG00000068489	PRR11

557	ENSG00000065911	MTHFD2
558	ENSG00000065548	ZC3H15
559	ENSG00000057019	DCBLD2
560	ENSG00000013810	TACC3
561	ENSG00000006625	GGCT
562	ENSG00000235571	SNX18P14
563	ENSG00000235109	ZSCAN31
564	ENSG00000214357	NEURL1B
565	ENSG00000198900	TOP1
566	ENSG00000198826	ARHGAP11A
567	ENSG00000198554	WDHD1
568	ENSG00000189337	KAZN
569	ENSG00000187837	H1-2
570	ENSG00000183741	CBX6
571	ENSG00000173848	NET1
572	ENSG00000171792	RHNO1
573	ENSG00000170734	POLH
574	ENSG00000168916	ZNF608
575	ENSG00000168078	PBK
576	ENSG00000166004	CEP295
577	ENSG00000164211	STARD4
578	ENSG00000163072	NOSTRIN
579	ENSG00000162783	IER5
580	ENSG00000161888	SPC24
581	ENSG00000161800	RACGAP1
582	ENSG00000160298	C21orf58
583	ENSG00000156463	SH3RF2
584	ENSG00000154640	BTG3
585	ENSG00000151276	MAGI1
586	ENSG00000149503	INCENP
587	ENSG00000146278	PNRC1
588	ENSG00000143322	ABL2
589	ENSG00000142149	HUNK
590	ENSG00000140332	TLE3
591	ENSG00000138658	ZGRF1
592	ENSG00000138376	BARD1
593	ENSG00000136108	CKAP2
594	ENSG00000134222	PSRC1
595	ENSG00000132780	NASP
596	ENSG00000128510	CPA4
597	ENSG00000127586	CHTF18
598	ENSG00000124216	SNAI1

599	ENSG00000123933	MXD4
600	ENSG00000122644	ARL4A
601	ENSG00000122566	HNRNPA2B1
602	ENSG00000118263	KLF7
603	ENSG00000118193	KIF14
604	ENSG00000112759	SLC29A1
605	ENSG00000105991	HOXA1
606	ENSG00000105325	FZR1
607	ENSG00000105270	CLIP3
608	ENSG00000104147	OIP5
609	ENSG00000103540	CCP110
610	ENSG00000101104	PABPC1L
611	ENSG00000100714	MTHFD1
612	ENSG00000099810	MTAP
613	ENSG00000095777	MYO3A
614	ENSG00000092140	G2E3
615	ENSG00000089693	MLF2
616	ENSG00000088325	TPX2
617	ENSG00000081019	RSBN1
618	ENSG00000077549	CAPZB
619	ENSG00000076382	SPAG5
620	ENSG00000075702	WDR62
621	ENSG00000070882	OSBPL3
622	ENSG00000068028	RASSF1
623	ENSG00000066279	ASPM
624	ENSG00000004897	CDC27
625	ENSG00000285730	Pseudogene
626	ENSG00000283269	Pseudogene
627	ENSG00000278259	MYO19
628	ENSG00000221978	CCNL2
629	ENSG00000213551	DNAJC9
630	ENSG00000196550	FAM72A
631	ENSG00000188610	FAM72B
632	ENSG00000186638	KIF24
633	ENSG00000183856	IQGAP3
634	ENSG00000181544	FANCB
635	ENSG00000180611	MB21D2
636	ENSG00000178999	AURKB
637	ENSG00000178343	SHISA3
638	ENSG00000178295	GEN1
639	ENSG00000178105	DDX10
640	ENSG00000176225	RTTN

641	ENSG00000175606	TMEM70
642	ENSG00000174371	EXO1
643	ENSG00000172667	ZMAT3
644	ENSG00000171940	ZNF217
645	ENSG00000170852	KBTBD2
646	ENSG00000169490	TM2D2
647	ENSG00000168061	SAC3D1
648	ENSG00000168010	ATG16L2
649	ENSG00000166925	TSC22D4
650	ENSG00000165886	UBTD1
651	ENSG00000165494	PCF11
652	ENSG00000165490	DDIAS
653	ENSG00000164985	PSIP1
654	ENSG00000164109	MAD2L1
655	ENSG00000164087	POC1A
656	ENSG00000162929	SANBR
657	ENSG00000162521	RBBP4
658	ENSG00000161011	SQSTM1
659	ENSG00000154473	BUB3
660	ENSG00000151725	CENPU
661	ENSG00000151692	RNF144A
662	ENSG00000146834	MEPCE
663	ENSG00000145526	CDH18
664	ENSG00000144395	CCDC150
665	ENSG00000144048	DUSP11
666	ENSG00000143228	NUF2
667	ENSG00000140443	IGF1R
668	ENSG00000138160	KIF11
669	ENSG00000136492	BRIP1
670	ENSG00000133454	MYO18B
671	ENSG00000133121	STARD13
672	ENSG00000133119	RFC3
673	ENSG00000130299	GTPBP3
674	ENSG00000124762	CDKN1A
675	ENSG00000123737	EXOSC9
676	ENSG00000121957	GPSM2
677	ENSG00000121741	ZMYM2
678	ENSG00000119333	DYNC2I2
679	ENSG00000111696	NT5DC3
680	ENSG00000106211	HSPB1
681	ENSG00000105856	HBP1
682	ENSG00000104671	DCTN6

683	ENSG00000101868	POLA1
684	ENSG00000101574	METTL4
685	ENSG00000101138	CSTF1
686	ENSG00000078900	TP73
687	ENSG00000075618	FSCN1
688	ENSG00000074266	EED
689	ENSG00000067082	KLF6
690	ENSG00000042088	TDP1
691	ENSG00000006634	DBF4
692	ENSG00000284946	Pseudogene
693	ENSG00000276966	H4C5
694	ENSG00000255152	MSH5-SAPCD1
695	ENSG00000237649	KIFC1
696	ENSG00000205212	CCDC144NL
697	ENSG00000204580	DDR1
698	ENSG00000204435	CSNK2B
699	ENSG00000197172	MAGEA6
700	ENSG00000186193	SAPCD2
701	ENSG00000184156	KCNQ3
702	ENSG00000178966	RMI1
703	ENSG00000177917	ARL6IP6
704	ENSG00000175592	FOSL1
705	ENSG00000173559	NABP1
706	ENSG00000171793	CTPS1
707	ENSG00000165891	E2F7
708	ENSG00000165502	RPL36AL
709	ENSG00000163808	KIF15
710	ENSG00000163535	SGO2
711	ENSG00000163507	CIP2A
712	ENSG00000151466	SCLT1
713	ENSG00000149639	SOGA1
714	ENSG00000142731	PLK4
715	ENSG00000139354	GAS2L3
716	ENSG00000137812	KNL1
717	ENSG00000137135	ARHGEF39
718	ENSG00000132485	ZRANB2
719	ENSG00000131462	TUBG1
720	ENSG00000129810	SGO1
721	ENSG00000125319	HROB
722	ENSG00000120694	HSPH1
723	ENSG00000119771	KLHL29

724	ENSG00000118655	DCLRE1B
725	ENSG00000118181	RPS25
726	ENSG00000115159	GPD2
727	ENSG00000113569	NUP155
728	ENSG00000109805	NCAPG
729	ENSG00000101945	SUV39H1
730	ENSG00000100242	SUN2
731	ENSG00000072864	NDE1
732	ENSG00000068885	IFT80
733	ENSG00000066322	ELOVL1
734	ENSG00000040275	SPDL1
735	ENSG00000033030	ZCCHC8
736	ENSG00000284906	ARHGAP11B
737	ENSG00000203811	H3C14
738	ENSG00000198355	PIM3
739	ENSG00000198088	NUP62CL
740	ENSG00000189060	H1-0
741	ENSG00000188529	SRSF10
742	ENSG00000188312	CENPP
743	ENSG00000187231	SESTD1
744	ENSG00000183864	TOB2
745	ENSG00000178974	FBXO34
746	ENSG00000177879	AP3S1
747	ENSG00000172765	TMCC1
748	ENSG00000166441	RPL27A
749	ENSG00000161692	DBF4B
750	ENSG00000158402	CDC25C
751	ENSG00000158373	H2BC5
752	ENSG00000154920	EME1
753	ENSG00000143942	CHAC2
754	ENSG00000138182	KIF20B
755	ENSG00000134291	TMEM106C
756	ENSG00000127337	YEATS4
757	ENSG00000125871	MGME1
758	ENSG00000121621	KIF18A
759	ENSG00000117069	ST6GALNAC5
760	ENSG00000115163	CENPA
761	ENSG00000111206	FOXM1
762	ENSG00000100519	PSMC6
763	ENSG00000070950	RAD18
764	ENSG00000051341	POLQ
765	ENSG00000278591	LOC124904144

766	ENSG00000278048	LOC124904138
767	ENSG00000277903	LOC124904140
768	ENSG00000276596	LOC124904139
769	ENSG00000275616	LOC124904135
770	ENSG00000275219	LOC124904143
771	ENSG00000274862	LOC124904141
772	ENSG00000274585	RNU2-1
773	ENSG00000274432	LOC124904137
774	ENSG00000274062	LOC124904142
775	ENSG00000207507	RNU6-9
776	ENSG00000207357	RNU6-2
777	ENSG00000207041	RNU6-3P
778	ENSG00000206965	RNU6-5P
779	ENSG00000206932	RNU6-4P
780	ENSG00000206899	RNU6-36P
781	ENSG00000184602	SNN
782	ENSG00000184260	H2AC20
783	ENSG00000176208	ATAD5
784	ENSG00000167011	NAT16
785	ENSG00000105821	DNAJC2
786	ENSG00000088305	DNMT3B
787	ENSG00000061337	LZTS1
788	ENSG00000031691	CENPQ
