# Supplementary material for: Identification of Human Cell Cycle Phase Markers Based on Single-Cell RNA-Seq Data by Using Machine Learning Methods
Source: Biomed Res Int. 2022 Aug 13;2022:2516653. doi: 10.1155/2022/2516653 (PMC9393965; doi:10.1155/2022/2516653)
Supplement: Supplementary 2 — Table S2: performance of IFS with different classification algorithms. [file 2516653.f2.pdf]

Table S2. Performance of IFS with different classification algorithms

(1) Performance of DT on top features yielded by three feature selection methods

mRMR					
**Numbers of features**	**G1**	**G2/M**	**S**	**ACC**	**MCC**
1	0.913	0.643	0.557	0.704	0.555
2	0.922	0.762	0.677	0.787	0.680
3	0.919	0.716	0.671	0.768	0.651
4	0.925	0.762	0.704	0.797	0.695
5	0.899	0.765	0.695	0.786	0.679
6	0.902	0.734	0.665	0.767	0.649
7	0.890	0.739	0.701	0.776	0.664
8	0.893	0.739	0.695	0.775	0.662
9	0.896	0.705	0.686	0.761	0.642
10	0.905	0.760	0.692	0.785	0.678
11	0.902	0.749	0.680	0.777	0.665
12	0.910	0.783	0.692	0.796	0.693
13	0.913	0.726	0.692	0.776	0.664
14	0.928	0.680	0.701	0.767	0.651
15	0.882	0.739	0.698	0.772	0.659
16	0.908	0.744	0.680	0.777	0.665
17	0.916	0.749	0.674	0.780	0.669
18	0.919	0.755	0.656	0.777	0.665
19	0.899	0.793	0.701	0.799	0.697
20	0.913	0.775	0.695	0.795	0.692
21	0.899	0.744	0.683	0.775	0.662
22	0.919	0.755	0.707	0.793	0.689
23	0.925	0.780	0.698	0.801	0.701
24	0.928	0.742	0.716	0.794	0.691
25	0.893	0.755	0.751	0.799	0.698
26	0.934	0.783	0.704	0.807	0.710
27	0.948	0.770	0.719	0.812	0.717
28	0.910	0.731	0.731	0.789	0.684
29	0.919	0.765	0.722	0.801	0.702
30	0.931	0.783	0.710	0.808	0.711
31	0.916	0.778	0.716	0.803	0.704
32	0.913	0.791	0.746	0.816	0.724
33	0.913	0.755	0.671	0.780	0.669
34	0.934	0.755	0.707	0.798	0.696
35	0.925	0.770	0.734	0.809	0.713
36	0.922	0.793	0.743	0.819	0.728
37	0.899	0.780	0.737	0.805	0.707

38	0.910	0.762	0.707	0.793	0.689
39	0.896	0.775	0.686	0.786	0.679
40	0.936	0.775	0.707	0.806	0.709
41	0.913	0.773	0.713	0.799	0.699
42	0.910	0.791	0.716	0.806	0.708
43	0.916	0.811	0.713	0.814	0.721
44	0.925	0.765	0.749	0.812	0.718
45	0.905	0.778	0.725	0.802	0.703
46	0.919	0.762	0.734	0.804	0.706
47	0.922	0.765	0.749	0.811	0.716
48	0.925	0.780	0.734	0.813	0.719
49	0.905	0.767	0.731	0.800	0.700
50	0.931	0.778	0.731	0.813	0.718
51	0.922	0.780	0.734	0.812	0.717
52	0.910	0.788	0.710	0.803	0.704
53	0.913	0.783	0.737	0.811	0.716
54	0.919	0.806	0.749	0.825	0.737
55	0.902	0.798	0.740	0.813	0.720
56	0.902	0.760	0.746	0.801	0.702
57	0.910	0.798	0.766	0.825	0.737
58	0.893	0.793	0.749	0.812	0.717
59	0.902	0.801	0.749	0.817	0.726
60	0.908	0.824	0.722	0.819	0.728
61	0.910	0.791	0.749	0.816	0.724
62	0.922	0.814	0.719	0.819	0.728
63	0.922	0.796	0.707	0.809	0.713
64	0.910	0.780	0.722	0.804	0.706
65	0.916	0.773	0.737	0.808	0.712
66	0.919	0.824	0.751	0.832	0.748
67	0.899	0.765	0.754	0.805	0.708
68	0.916	0.804	0.728	0.816	0.724
69	0.925	0.778	0.728	0.810	0.714
70	0.902	0.773	0.775	0.815	0.724
71	0.925	0.809	0.754	0.829	0.744
72	0.908	0.806	0.760	0.825	0.737
73	0.919	0.780	0.737	0.812	0.717
74	0.913	0.780	0.751	0.814	0.722
75	0.925	0.775	0.737	0.812	0.717
76	0.919	0.806	0.740	0.822	0.732
77	0.908	0.791	0.734	0.811	0.716
78	0.908	0.783	0.754	0.814	0.722
79	0.905	0.804	0.751	0.820	0.730

80	0.913	0.786	0.743	0.813	0.720
81	0.913	0.786	0.754	0.817	0.726
82	0.902	0.796	0.716	0.805	0.707
83	0.913	0.798	0.731	0.814	0.721
84	0.873	0.791	0.749	0.804	0.706
85	0.916	0.791	0.731	0.813	0.718
86	0.905	0.814	0.763	0.828	0.741
87	0.916	0.806	0.754	0.826	0.738
88	0.922	0.793	0.751	0.822	0.733
89	0.916	0.806	0.743	0.822	0.732
90	0.908	0.793	0.737	0.813	0.718
91	0.908	0.783	0.713	0.801	0.701
92	0.916	0.762	0.749	0.808	0.712
93	0.902	0.762	0.734	0.799	0.698
94	0.925	0.775	0.731	0.810	0.714
95	0.919	0.786	0.710	0.805	0.707
96	0.905	0.780	0.731	0.805	0.707
97	0.913	0.817	0.710	0.814	0.721
98	0.896	0.773	0.749	0.805	0.708
99	0.925	0.809	0.728	0.821	0.731
100	0.902	0.793	0.743	0.813	0.719
101	0.899	0.770	0.737	0.801	0.702
102	0.902	0.798	0.728	0.810	0.714
103	0.916	0.798	0.698	0.805	0.707
104	0.925	0.786	0.746	0.818	0.727
105	0.908	0.791	0.713	0.804	0.706
106	0.916	0.783	0.734	0.811	0.716
107	0.905	0.796	0.704	0.802	0.703
108	0.919	0.793	0.746	0.819	0.728
109	0.925	0.809	0.760	0.831	0.747
110	0.902	0.752	0.740	0.797	0.695
111	0.934	0.788	0.743	0.821	0.731
112	0.905	0.811	0.743	0.820	0.730
113	0.910	0.780	0.749	0.813	0.719
114	0.905	0.788	0.737	0.810	0.714
115	0.925	0.811	0.737	0.825	0.737
116	0.913	0.770	0.746	0.809	0.713
117	0.913	0.796	0.734	0.814	0.721
118	0.887	0.780	0.731	0.799	0.699
119	0.910	0.814	0.751	0.826	0.738
120	0.905	0.796	0.692	0.799	0.697
121	0.884	0.773	0.751	0.802	0.704

122	0.899	0.778	0.734	0.803	0.705
123	0.910	0.809	0.740	0.820	0.730
124	0.913	0.778	0.763	0.817	0.726
125	0.922	0.773	0.707	0.800	0.700
126	0.893	0.801	0.710	0.802	0.703
127	0.896	0.793	0.728	0.806	0.708
128	0.899	0.817	0.749	0.822	0.732
129	0.902	0.783	0.763	0.815	0.723
130	0.910	0.801	0.728	0.813	0.720
131	0.913	0.788	0.713	0.805	0.707
132	0.919	0.780	0.731	0.810	0.714
133	0.916	0.780	0.710	0.802	0.703
134	0.916	0.760	0.719	0.798	0.696
135	0.928	0.788	0.743	0.819	0.728
136	0.922	0.817	0.710	0.817	0.725
137	0.873	0.739	0.704	0.771	0.657
138	0.902	0.770	0.746	0.805	0.708
139	0.928	0.762	0.734	0.807	0.711
140	0.931	0.791	0.734	0.818	0.727
141	0.913	0.780	0.716	0.803	0.704
142	0.910	0.806	0.737	0.818	0.727
143	0.905	0.770	0.743	0.805	0.708
144	0.910	0.798	0.689	0.800	0.700
145	0.919	0.788	0.734	0.813	0.720
146	0.905	0.755	0.737	0.798	0.697
147	0.919	0.760	0.704	0.794	0.690
148	0.916	0.786	0.734	0.812	0.717
149	0.910	0.798	0.698	0.803	0.704
150	0.931	0.804	0.683	0.807	0.710
151	0.910	0.814	0.695	0.808	0.711
152	0.931	0.775	0.734	0.813	0.718
153	0.916	0.806	0.704	0.810	0.714
154	0.910	0.770	0.740	0.806	0.709
155	0.919	0.788	0.757	0.821	0.731
156	0.908	0.796	0.743	0.815	0.723
157	0.908	0.747	0.746	0.799	0.698
158	0.916	0.829	0.760	0.836	0.753
159	0.916	0.786	0.743	0.814	0.721
160	0.919	0.806	0.725	0.817	0.725
161	0.916	0.749	0.719	0.794	0.691
162	0.896	0.788	0.728	0.804	0.706
163	0.913	0.786	0.722	0.807	0.710

164	0.910	0.783	0.731	0.808	0.711
165	0.916	0.783	0.746	0.814	0.721
166	0.899	0.793	0.746	0.813	0.719
167	0.922	0.796	0.743	0.820	0.730
168	0.910	0.773	0.722	0.801	0.701
169	0.910	0.747	0.746	0.799	0.700
170	0.925	0.809	0.710	0.815	0.722
171	0.925	0.762	0.722	0.802	0.703
172	0.905	0.773	0.719	0.799	0.697
173	0.908	0.793	0.734	0.812	0.717
174	0.902	0.796	0.719	0.806	0.708
175	0.916	0.783	0.716	0.805	0.707
176	0.925	0.783	0.781	0.828	0.743
177	0.916	0.739	0.740	0.797	0.696
178	0.899	0.796	0.716	0.804	0.706
179	0.902	0.819	0.740	0.821	0.731
180	0.908	0.793	0.707	0.803	0.704
181	0.916	0.765	0.740	0.806	0.709
182	0.922	0.775	0.719	0.805	0.707
183	0.934	0.793	0.734	0.820	0.730
184	0.913	0.749	0.734	0.798	0.696
185	0.925	0.770	0.701	0.799	0.697
186	0.916	0.775	0.725	0.805	0.707
187	0.916	0.775	0.704	0.799	0.697
188	0.925	0.811	0.728	0.822	0.732
189	0.899	0.778	0.728	0.801	0.702
190	0.922	0.760	0.689	0.790	0.684
191	0.916	0.793	0.725	0.812	0.717
192	0.913	0.783	0.722	0.806	0.709
193	0.931	0.786	0.734	0.816	0.724
194	0.934	0.747	0.695	0.791	0.686
195	0.905	0.752	0.725	0.793	0.689
196	0.919	0.793	0.754	0.822	0.733
197	0.928	0.788	0.719	0.812	0.717
198	0.905	0.760	0.680	0.782	0.672
199	0.936	0.752	0.695	0.794	0.690
200	0.931	0.773	0.716	0.806	0.709
201	0.925	0.796	0.743	0.821	0.731
202	0.910	0.775	0.716	0.800	0.700
203	0.910	0.767	0.719	0.799	0.697
204	0.919	0.765	0.746	0.809	0.713
205	0.931	0.809	0.707	0.816	0.724

206	0.913	0.739	0.737	0.795	0.692
207	0.931	0.775	0.713	0.806	0.709
208	0.939	0.783	0.734	0.818	0.727
209	0.922	0.770	0.707	0.799	0.699
210	0.925	0.793	0.722	0.813	0.720
211	0.905	0.814	0.692	0.805	0.707
212	0.942	0.773	0.734	0.815	0.723
213	0.916	0.788	0.719	0.808	0.711
214	0.934	0.793	0.713	0.813	0.720
215	0.908	0.755	0.719	0.793	0.689
216	0.934	0.788	0.754	0.825	0.737
217	0.913	0.778	0.710	0.800	0.700
218	0.928	0.783	0.722	0.811	0.716
219	0.913	0.770	0.707	0.797	0.694
220	0.919	0.786	0.728	0.811	0.716
221	0.934	0.778	0.722	0.811	0.716
222	0.928	0.788	0.731	0.815	0.722
223	0.913	0.817	0.740	0.824	0.735
224	0.902	0.757	0.754	0.803	0.706
225	0.916	0.783	0.722	0.807	0.710
226	0.931	0.786	0.725	0.813	0.720
227	0.936	0.783	0.743	0.820	0.730
228	0.910	0.793	0.728	0.811	0.715
229	0.913	0.762	0.743	0.805	0.708
230	0.925	0.778	0.716	0.806	0.709
231	0.936	0.809	0.704	0.817	0.725
232	0.919	0.765	0.740	0.807	0.710
233	0.939	0.736	0.722	0.798	0.696
234	0.913	0.778	0.707	0.799	0.699
235	0.913	0.775	0.707	0.799	0.697
236	0.936	0.788	0.707	0.811	0.716
237	0.905	0.760	0.734	0.799	0.698
238	0.896	0.778	0.722	0.799	0.698
239	0.919	0.775	0.677	0.791	0.686
240	0.922	0.778	0.725	0.808	0.711
241	0.910	0.773	0.749	0.810	0.715
242	0.916	0.804	0.704	0.809	0.713
243	0.905	0.780	0.728	0.804	0.706
244	0.893	0.744	0.689	0.775	0.662
245	0.919	0.796	0.740	0.818	0.727
246	0.910	0.762	0.719	0.797	0.695
247	0.931	0.786	0.728	0.814	0.721

248	0.916	0.747	0.728	0.796	0.693
249	0.916	0.788	0.680	0.796	0.693
250	0.905	0.814	0.731	0.817	0.725
251	0.931	0.798	0.754	0.828	0.741
252	0.908	0.770	0.760	0.812	0.718
253	0.925	0.770	0.725	0.806	0.709
254	0.910	0.798	0.731	0.813	0.720
255	0.893	0.798	0.725	0.806	0.709
256	0.922	0.765	0.698	0.795	0.692
257	0.934	0.755	0.710	0.799	0.697
258	0.913	0.760	0.722	0.798	0.696
259	0.913	0.775	0.689	0.793	0.689
260	0.931	0.747	0.710	0.795	0.692
261	0.908	0.791	0.707	0.802	0.703
262	0.890	0.791	0.737	0.806	0.709
263	0.934	0.752	0.749	0.810	0.715
264	0.939	0.791	0.713	0.814	0.721
265	0.925	0.806	0.707	0.813	0.720
266	0.928	0.775	0.710	0.804	0.706
267	0.916	0.742	0.689	0.782	0.672
268	0.919	0.752	0.728	0.799	0.698
269	0.899	0.783	0.760	0.813	0.720
270	0.922	0.796	0.713	0.811	0.715
271	0.925	0.773	0.737	0.811	0.716
272	0.928	0.778	0.698	0.801	0.702
273	0.919	0.747	0.737	0.799	0.699
274	0.922	0.819	0.734	0.826	0.738
275	0.919	0.775	0.728	0.807	0.710
276	0.916	0.786	0.722	0.808	0.711
277	0.913	0.798	0.743	0.818	0.727
278	0.928	0.770	0.704	0.800	0.700
279	0.908	0.780	0.751	0.813	0.719
280	0.913	0.788	0.710	0.804	0.706
281	0.908	0.775	0.734	0.805	0.707
282	0.934	0.801	0.740	0.825	0.737
283	0.916	0.788	0.740	0.814	0.721
284	0.905	0.778	0.707	0.797	0.694
285	0.899	0.788	0.698	0.796	0.693
286	0.916	0.731	0.754	0.799	0.699
287	0.928	0.767	0.710	0.801	0.702
288	0.908	0.791	0.728	0.809	0.713
289	0.910	0.783	0.731	0.808	0.712

290	0.913	0.798	0.698	0.804	0.705
291	0.899	0.793	0.707	0.800	0.700
292	0.908	0.793	0.701	0.801	0.701
293	0.905	0.798	0.698	0.801	0.701
294	0.919	0.809	0.728	0.819	0.728
295	0.908	0.798	0.737	0.814	0.721
296	0.925	0.752	0.725	0.799	0.699
297	0.922	0.773	0.728	0.807	0.710
298	0.922	0.793	0.749	0.821	0.731
299	0.910	0.773	0.737	0.806	0.709
300	0.925	0.773	0.701	0.799	0.699
301	0.931	0.780	0.704	0.805	0.707
302	0.905	0.760	0.719	0.794	0.691
303	0.913	0.744	0.722	0.792	0.688
304	0.913	0.798	0.719	0.811	0.715
305	0.905	0.778	0.737	0.806	0.709
306	0.934	0.757	0.722	0.803	0.705
307	0.931	0.770	0.719	0.806	0.709
308	0.905	0.757	0.740	0.799	0.699
309	0.908	0.775	0.719	0.800	0.700
310	0.899	0.775	0.719	0.798	0.696
311	0.925	0.786	0.689	0.800	0.700
312	0.908	0.775	0.772	0.817	0.726
313	0.910	0.801	0.731	0.814	0.721
314	0.910	0.767	0.743	0.806	0.709
315	0.925	0.809	0.728	0.821	0.731
316	0.916	0.755	0.701	0.790	0.685
317	0.913	0.804	0.728	0.815	0.722
318	0.922	0.791	0.728	0.813	0.720
319	0.939	0.778	0.704	0.807	0.710
320	0.896	0.788	0.746	0.810	0.714
321	0.902	0.778	0.707	0.796	0.693
322	0.916	0.793	0.704	0.805	0.707
323	0.902	0.793	0.749	0.814	0.721
324	0.922	0.798	0.713	0.812	0.717
325	0.925	0.780	0.719	0.808	0.711
326	0.916	0.798	0.731	0.815	0.722
327	0.916	0.780	0.725	0.807	0.710
328	0.916	0.791	0.751	0.819	0.728
329	0.922	0.788	0.695	0.802	0.703
330	0.925	0.801	0.716	0.814	0.721
331	0.896	0.775	0.740	0.803	0.704

332	0.913	0.736	0.734	0.793	0.690
333	0.922	0.765	0.719	0.801	0.702
334	0.910	0.752	0.734	0.798	0.696
335	0.931	0.770	0.722	0.807	0.710
336	0.910	0.783	0.746	0.813	0.719
337	0.905	0.770	0.710	0.795	0.692
338	0.916	0.773	0.722	0.803	0.704
339	0.922	0.770	0.737	0.809	0.713
340	0.925	0.793	0.734	0.817	0.725
341	0.916	0.783	0.710	0.803	0.704
342	0.928	0.762	0.710	0.799	0.699
343	0.913	0.755	0.743	0.802	0.704
344	0.919	0.783	0.746	0.815	0.723
345	0.902	0.773	0.719	0.798	0.696
346	0.931	0.783	0.710	0.808	0.711
347	0.934	0.765	0.719	0.805	0.707
348	0.908	0.775	0.728	0.803	0.705
349	0.919	0.755	0.704	0.792	0.687
350	0.922	0.791	0.722	0.812	0.717
351	0.936	0.778	0.719	0.811	0.715
352	0.896	0.729	0.734	0.784	0.678
353	0.913	0.749	0.707	0.789	0.683
354	0.916	0.747	0.725	0.795	0.692
355	0.908	0.760	0.713	0.793	0.689
356	0.931	0.804	0.710	0.815	0.722
357	0.916	0.783	0.707	0.802	0.703
358	0.925	0.788	0.728	0.813	0.720
359	0.899	0.770	0.725	0.798	0.696
360	0.905	0.783	0.763	0.816	0.725
361	0.919	0.793	0.746	0.819	0.728
362	0.910	0.801	0.719	0.811	0.715
363	0.913	0.765	0.731	0.802	0.703
364	0.902	0.773	0.716	0.797	0.695
365	0.919	0.783	0.740	0.813	0.720
366	0.916	0.793	0.665	0.793	0.689
367	0.919	0.770	0.740	0.809	0.713
368	0.931	0.780	0.704	0.805	0.707
369	0.910	0.749	0.713	0.790	0.685
370	0.908	0.791	0.725	0.808	0.711
371	0.899	0.760	0.698	0.785	0.678
372	0.913	0.773	0.728	0.804	0.706
373	0.905	0.788	0.749	0.813	0.720

374	0.942	0.778	0.784	0.833	0.750
375	0.919	0.773	0.731	0.807	0.710
376	0.910	0.804	0.749	0.821	0.731
377	0.905	0.778	0.722	0.801	0.702
378	0.928	0.791	0.722	0.813	0.720
379	0.910	0.775	0.701	0.796	0.693
380	0.928	0.770	0.695	0.798	0.696
381	0.882	0.796	0.749	0.809	0.713
382	0.896	0.788	0.692	0.793	0.689
383	0.922	0.798	0.716	0.813	0.718
384	0.908	0.775	0.707	0.797	0.694
385	0.916	0.765	0.725	0.801	0.702
386	0.922	0.780	0.749	0.816	0.724
387	0.916	0.796	0.710	0.808	0.711
388	0.919	0.814	0.743	0.826	0.738
389	0.925	0.780	0.722	0.809	0.713
390	0.913	0.801	0.701	0.806	0.708
391	0.899	0.806	0.743	0.816	0.724
392	0.902	0.806	0.743	0.817	0.725
393	0.902	0.775	0.743	0.806	0.709
394	0.919	0.788	0.734	0.813	0.720
395	0.928	0.780	0.713	0.807	0.710
396	0.905	0.817	0.737	0.820	0.730
397	0.916	0.786	0.713	0.805	0.707
398	0.905	0.780	0.713	0.799	0.699
399	0.919	0.824	0.728	0.825	0.737
400	0.902	0.788	0.710	0.800	0.700
401	0.919	0.783	0.725	0.809	0.713
402	0.910	0.755	0.749	0.803	0.705
403	0.922	0.819	0.749	0.830	0.745
404	0.899	0.780	0.757	0.812	0.717
405	0.905	0.817	0.763	0.828	0.742
406	0.908	0.783	0.713	0.801	0.701
407	0.905	0.804	0.728	0.813	0.718
408	0.893	0.791	0.731	0.805	0.707
409	0.910	0.780	0.734	0.808	0.711
410	0.902	0.773	0.749	0.807	0.711
411	0.902	0.770	0.701	0.791	0.686
412	0.908	0.780	0.749	0.812	0.717
413	0.913	0.806	0.734	0.818	0.727
414	0.896	0.770	0.760	0.808	0.712
415	0.893	0.791	0.743	0.809	0.713

416	0.916	0.804	0.746	0.822	0.732
417	0.916	0.804	0.740	0.820	0.730
418	0.913	0.806	0.766	0.828	0.743
419	0.910	0.806	0.728	0.815	0.722
420	0.919	0.749	0.746	0.803	0.705
421	0.899	0.806	0.719	0.809	0.713
422	0.908	0.786	0.731	0.808	0.712
423	0.913	0.809	0.737	0.820	0.729
424	0.913	0.791	0.751	0.818	0.727
425	0.890	0.796	0.728	0.805	0.707
426	0.919	0.775	0.719	0.804	0.706
427	0.893	0.819	0.740	0.818	0.727
428	0.925	0.827	0.734	0.829	0.744
429	0.908	0.775	0.751	0.811	0.716
430	0.879	0.775	0.746	0.799	0.699
431	0.896	0.770	0.716	0.794	0.690
432	0.913	0.798	0.740	0.817	0.725
433	0.939	0.791	0.751	0.827	0.740
434	0.905	0.786	0.725	0.805	0.707
435	0.905	0.791	0.701	0.799	0.698
436	0.899	0.814	0.722	0.813	0.718
437	0.922	0.791	0.737	0.816	0.724
438	0.910	0.793	0.731	0.812	0.717
439	0.913	0.804	0.713	0.811	0.715
440	0.896	0.778	0.766	0.813	0.719
441	0.896	0.806	0.740	0.814	0.721
442	0.908	0.786	0.722	0.805	0.707
443	0.910	0.783	0.754	0.815	0.723
444	0.899	0.796	0.731	0.809	0.713
445	0.910	0.765	0.766	0.813	0.719
446	0.905	0.791	0.737	0.811	0.716
447	0.905	0.819	0.725	0.817	0.725
448	0.908	0.786	0.757	0.816	0.724
449	0.905	0.806	0.743	0.818	0.727
450	0.922	0.765	0.734	0.806	0.709
451	0.922	0.801	0.743	0.822	0.732
452	0.890	0.786	0.746	0.807	0.710
453	0.896	0.804	0.725	0.809	0.713
454	0.896	0.811	0.763	0.824	0.735
455	0.934	0.786	0.757	0.825	0.737
456	0.910	0.814	0.719	0.815	0.722
457	0.905	0.804	0.731	0.813	0.720

458	0.922	0.765	0.769	0.817	0.726
459	0.913	0.786	0.740	0.813	0.718
460	0.916	0.801	0.766	0.828	0.741
461	0.913	0.811	0.716	0.814	0.721
462	0.905	0.804	0.740	0.816	0.724
463	0.908	0.804	0.728	0.813	0.720
464	0.919	0.809	0.725	0.818	0.727
465	0.899	0.809	0.722	0.811	0.715
466	0.899	0.783	0.719	0.800	0.700
467	0.890	0.804	0.734	0.810	0.714
468	0.916	0.780	0.760	0.818	0.727
469	0.908	0.811	0.763	0.828	0.741
470	0.905	0.770	0.734	0.802	0.703
471	0.908	0.796	0.701	0.802	0.703
472	0.910	0.721	0.707	0.778	0.667
473	0.905	0.770	0.754	0.809	0.713
474	0.919	0.775	0.722	0.805	0.707
475	0.928	0.796	0.728	0.817	0.725
476	0.890	0.742	0.716	0.782	0.673
477	0.899	0.796	0.731	0.809	0.713
478	0.890	0.775	0.763	0.809	0.714
479	0.905	0.786	0.728	0.806	0.708
480	0.913	0.798	0.772	0.828	0.741
481	0.910	0.778	0.754	0.813	0.720
482	0.910	0.755	0.746	0.802	0.704
483	0.908	0.801	0.760	0.823	0.734
484	0.905	0.778	0.728	0.803	0.704
485	0.922	0.757	0.766	0.813	0.721
486	0.913	0.798	0.728	0.813	0.720
487	0.922	0.804	0.754	0.827	0.740
488	0.899	0.806	0.734	0.813	0.720
489	0.910	0.778	0.757	0.814	0.722
490	0.913	0.829	0.731	0.826	0.738
491	0.902	0.783	0.731	0.805	0.707
492	0.931	0.793	0.728	0.817	0.725
493	0.925	0.773	0.713	0.803	0.704
494	0.913	0.819	0.772	0.835	0.752
495	0.908	0.786	0.722	0.805	0.707
496	0.910	0.770	0.710	0.797	0.695
497	0.913	0.757	0.734	0.800	0.701
498	0.919	0.780	0.757	0.818	0.727
499	0.913	0.770	0.760	0.813	0.721

500	0.910	0.786	0.722	0.806	0.709
501	0.910	0.783	0.778	0.823	0.735
502	0.916	0.767	0.731	0.804	0.706
503	0.922	0.786	0.737	0.814	0.721
504	0.905	0.801	0.746	0.817	0.725
505	0.916	0.783	0.731	0.810	0.714
506	0.902	0.752	0.716	0.789	0.684
507	0.916	0.783	0.716	0.805	0.707
508	0.905	0.775	0.754	0.811	0.716
509	0.922	0.804	0.728	0.818	0.727
510	0.896	0.793	0.749	0.813	0.718
511	0.902	0.762	0.740	0.800	0.701
512	0.908	0.778	0.698	0.795	0.692
513	0.905	0.796	0.722	0.808	0.711
514	0.931	0.762	0.751	0.813	0.720
515	0.905	0.801	0.743	0.816	0.724
516	0.905	0.767	0.713	0.795	0.692
517	0.908	0.775	0.746	0.809	0.713
518	0.908	0.770	0.728	0.801	0.702
519	0.916	0.765	0.719	0.799	0.699
520	0.910	0.773	0.683	0.789	0.683
521	0.902	0.778	0.704	0.795	0.692
522	0.910	0.773	0.725	0.802	0.703
523	0.905	0.752	0.701	0.785	0.678
524	0.913	0.773	0.731	0.805	0.707
525	0.896	0.757	0.695	0.783	0.673
526	0.899	0.770	0.746	0.804	0.706
527	0.913	0.773	0.731	0.805	0.707
528	0.913	0.801	0.728	0.814	0.721
529	0.905	0.760	0.737	0.799	0.699
530	0.908	0.767	0.743	0.805	0.707
531	0.908	0.783	0.725	0.805	0.707
532	0.919	0.793	0.731	0.814	0.721
533	0.934	0.783	0.716	0.811	0.716
534	0.905	0.806	0.757	0.823	0.734
535	0.919	0.796	0.710	0.809	0.713
536	0.913	0.767	0.713	0.798	0.696
537	0.925	0.767	0.731	0.807	0.710
538	0.908	0.729	0.710	0.781	0.671
539	0.902	0.793	0.722	0.806	0.708
540	0.916	0.780	0.766	0.820	0.730
541	0.919	0.783	0.731	0.811	0.716

542	0.916	0.778	0.695	0.797	0.694
543	0.908	0.773	0.754	0.811	0.716
544	0.910	0.747	0.704	0.786	0.679
545	0.908	0.760	0.728	0.798	0.696
546	0.908	0.747	0.766	0.805	0.709
547	0.916	0.783	0.704	0.801	0.701
548	0.919	0.775	0.725	0.806	0.709
549	0.913	0.796	0.716	0.809	0.713
550	0.910	0.778	0.740	0.809	0.713
551	0.899	0.801	0.760	0.820	0.730
552	0.916	0.798	0.734	0.816	0.724
553	0.913	0.778	0.754	0.814	0.722
554	0.919	0.778	0.716	0.804	0.706
555	0.908	0.788	0.728	0.808	0.711
556	0.919	0.801	0.749	0.823	0.734
557	0.910	0.780	0.766	0.818	0.727
558	0.905	0.811	0.734	0.817	0.725
559	0.916	0.775	0.725	0.805	0.707
560	0.925	0.793	0.749	0.822	0.733
561	0.910	0.780	0.704	0.799	0.697
562	0.910	0.788	0.704	0.801	0.701
563	0.902	0.775	0.722	0.799	0.699
564	0.893	0.775	0.719	0.796	0.693
565	0.908	0.765	0.701	0.791	0.686
566	0.922	0.783	0.710	0.805	0.707
567	0.913	0.796	0.734	0.814	0.721
568	0.919	0.796	0.707	0.808	0.711
569	0.902	0.806	0.722	0.811	0.715
570	0.902	0.811	0.734	0.816	0.724
571	0.922	0.793	0.704	0.807	0.710
572	0.908	0.770	0.740	0.805	0.708
573	0.902	0.783	0.740	0.808	0.712
574	0.913	0.809	0.704	0.810	0.714
575	0.902	0.773	0.743	0.805	0.708
576	0.928	0.791	0.722	0.813	0.720
577	0.922	0.796	0.713	0.811	0.715
578	0.922	0.757	0.716	0.798	0.696
579	0.910	0.767	0.707	0.795	0.692
580	0.905	0.757	0.719	0.793	0.689
581	0.899	0.747	0.695	0.780	0.669
582	0.908	0.798	0.707	0.805	0.707
583	0.913	0.796	0.731	0.813	0.720

584	0.913	0.773	0.728	0.804	0.706
585	0.905	0.780	0.737	0.807	0.710
586	0.919	0.788	0.746	0.817	0.726
587	0.899	0.809	0.734	0.814	0.721
588	0.908	0.755	0.701	0.787	0.681
589	0.925	0.793	0.707	0.809	0.713
590	0.908	0.780	0.731	0.806	0.709
591	0.916	0.775	0.686	0.793	0.689
592	0.905	0.793	0.737	0.812	0.717
593	0.905	0.809	0.775	0.829	0.744
594	0.922	0.796	0.728	0.815	0.723
595	0.931	0.780	0.725	0.812	0.717
596	0.913	0.804	0.728	0.815	0.722
597	0.919	0.786	0.731	0.812	0.717
598	0.916	0.778	0.728	0.807	0.710
599	0.919	0.770	0.760	0.815	0.723
600	0.908	0.770	0.743	0.806	0.709
601	0.902	0.786	0.763	0.816	0.724
602	0.905	0.793	0.692	0.798	0.696
603	0.913	0.786	0.769	0.822	0.733
604	0.916	0.788	0.737	0.813	0.720
605	0.905	0.775	0.698	0.793	0.689
606	0.902	0.791	0.740	0.811	0.716
607	0.916	0.765	0.701	0.794	0.690
608	0.913	0.783	0.716	0.804	0.706
609	0.908	0.765	0.713	0.795	0.692
610	0.908	0.793	0.740	0.813	0.720
611	0.916	0.762	0.734	0.803	0.705
612	0.910	0.762	0.754	0.808	0.712
613	0.910	0.780	0.722	0.804	0.706
614	0.902	0.786	0.737	0.808	0.711
615	0.896	0.783	0.710	0.797	0.694
616	0.908	0.791	0.737	0.812	0.717
617	0.916	0.767	0.728	0.803	0.705
618	0.902	0.796	0.743	0.813	0.720
619	0.910	0.793	0.719	0.808	0.711
620	0.902	0.749	0.734	0.794	0.691
621	0.908	0.788	0.743	0.813	0.718
622	0.913	0.775	0.731	0.806	0.709
623	0.908	0.801	0.737	0.815	0.722
624	0.922	0.788	0.734	0.814	0.721
625	0.916	0.767	0.686	0.790	0.684

626	0.919	0.778	0.740	0.812	0.717
627	0.905	0.780	0.734	0.806	0.709
628	0.910	0.778	0.740	0.809	0.713
629	0.910	0.806	0.710	0.810	0.714
630	0.905	0.798	0.740	0.814	0.721
631	0.905	0.767	0.746	0.805	0.708
632	0.908	0.791	0.725	0.808	0.711
633	0.925	0.783	0.749	0.818	0.727
634	0.908	0.786	0.722	0.805	0.707
635	0.905	0.762	0.728	0.798	0.696
636	0.908	0.788	0.728	0.808	0.711
637	0.931	0.752	0.737	0.805	0.708
638	0.910	0.786	0.689	0.796	0.693
639	0.905	0.775	0.740	0.806	0.709
640	0.928	0.793	0.722	0.814	0.721
641	0.936	0.762	0.710	0.802	0.703
642	0.925	0.762	0.766	0.816	0.725
643	0.905	0.773	0.722	0.799	0.699
644	0.913	0.809	0.740	0.821	0.731
645	0.928	0.778	0.725	0.810	0.714
646	0.919	0.817	0.698	0.813	0.718
647	0.899	0.778	0.737	0.804	0.706
648	0.905	0.778	0.737	0.806	0.709
649	0.913	0.778	0.710	0.800	0.700
650	0.928	0.798	0.701	0.810	0.714
651	0.916	0.814	0.728	0.820	0.730
652	0.902	0.760	0.737	0.799	0.698
653	0.916	0.773	0.737	0.808	0.711
654	0.908	0.811	0.716	0.813	0.718
655	0.905	0.798	0.707	0.804	0.705
656	0.913	0.798	0.766	0.826	0.739
657	0.908	0.783	0.722	0.804	0.706
658	0.902	0.804	0.760	0.822	0.733
659	0.890	0.806	0.737	0.812	0.717
660	0.899	0.793	0.716	0.803	0.704
661	0.913	0.786	0.731	0.810	0.714
662	0.913	0.798	0.740	0.817	0.725
663	0.910	0.762	0.743	0.804	0.706
664	0.913	0.780	0.734	0.809	0.713
665	0.899	0.798	0.749	0.815	0.723
666	0.919	0.775	0.725	0.806	0.709
667	0.916	0.804	0.710	0.811	0.715

668	0.908	0.822	0.695	0.810	0.714
669	0.934	0.788	0.704	0.809	0.713
670	0.905	0.791	0.716	0.804	0.706
671	0.902	0.791	0.737	0.810	0.714
672	0.916	0.780	0.731	0.809	0.713
673	0.910	0.809	0.716	0.813	0.718
674	0.916	0.798	0.754	0.823	0.734
675	0.899	0.798	0.692	0.798	0.696
676	0.896	0.780	0.722	0.799	0.699
677	0.916	0.806	0.719	0.814	0.721
678	0.899	0.791	0.740	0.810	0.715
679	0.913	0.786	0.734	0.811	0.716
680	0.922	0.786	0.719	0.809	0.713
681	0.910	0.788	0.751	0.816	0.724
682	0.931	0.801	0.740	0.824	0.735
683	0.913	0.762	0.725	0.799	0.699
684	0.899	0.824	0.737	0.821	0.731
685	0.905	0.773	0.713	0.797	0.695
686	0.902	0.767	0.719	0.796	0.693
687	0.902	0.760	0.749	0.802	0.704
688	0.916	0.773	0.749	0.812	0.717
689	0.899	0.809	0.716	0.809	0.713
690	0.908	0.806	0.749	0.821	0.731
691	0.902	0.775	0.689	0.789	0.683
692	0.916	0.770	0.731	0.805	0.707
693	0.902	0.793	0.719	0.805	0.707
694	0.887	0.817	0.713	0.807	0.710
695	0.902	0.762	0.695	0.786	0.679
696	0.913	0.775	0.728	0.805	0.707
697	0.890	0.798	0.719	0.803	0.704
698	0.919	0.822	0.754	0.832	0.748
699	0.908	0.811	0.734	0.818	0.727
700	0.916	0.726	0.722	0.786	0.680
701	0.934	0.798	0.740	0.824	0.735
702	0.908	0.744	0.695	0.782	0.672
703	0.928	0.819	0.725	0.825	0.737
704	0.902	0.791	0.740	0.811	0.716
705	0.910	0.788	0.737	0.812	0.717
706	0.922	0.796	0.734	0.817	0.725
707	0.925	0.783	0.710	0.806	0.708
708	0.922	0.804	0.722	0.816	0.724
709	0.908	0.796	0.731	0.812	0.717

710	0.910	0.775	0.719	0.801	0.701
711	0.925	0.747	0.671	0.781	0.671
712	0.908	0.824	0.704	0.813	0.720
713	0.910	0.822	0.707	0.814	0.721
714	0.905	0.798	0.713	0.806	0.708
715	0.910	0.767	0.749	0.808	0.712
716	0.916	0.773	0.728	0.805	0.708
717	0.902	0.734	0.695	0.776	0.664
718	0.919	0.749	0.722	0.796	0.693
719	0.902	0.788	0.737	0.809	0.713
720	0.896	0.778	0.731	0.801	0.702
721	0.922	0.783	0.704	0.803	0.704
722	0.913	0.786	0.740	0.813	0.719
723	0.902	0.786	0.737	0.808	0.711
724	0.905	0.767	0.731	0.800	0.700
725	0.905	0.780	0.719	0.801	0.701
726	0.908	0.760	0.743	0.802	0.703
727	0.893	0.780	0.716	0.797	0.695
728	0.899	0.778	0.731	0.802	0.703
729	0.910	0.765	0.713	0.796	0.693
730	0.893	0.804	0.695	0.799	0.697
731	0.905	0.796	0.757	0.819	0.728
732	0.908	0.765	0.749	0.806	0.709
733	0.910	0.752	0.731	0.797	0.695
734	0.908	0.749	0.737	0.797	0.695
735	0.922	0.798	0.734	0.818	0.727
736	0.913	0.786	0.760	0.819	0.729
737	0.908	0.747	0.731	0.794	0.691
738	0.913	0.814	0.725	0.818	0.727
739	0.919	0.793	0.751	0.821	0.731
740	0.902	0.780	0.734	0.805	0.707
741	0.919	0.765	0.728	0.803	0.704
742	0.922	0.762	0.763	0.814	0.722
743	0.902	0.806	0.743	0.817	0.726
744	0.922	0.773	0.716	0.803	0.704
745	0.919	0.793	0.728	0.813	0.720
746	0.919	0.806	0.740	0.822	0.732
747	0.916	0.798	0.766	0.827	0.740
748	0.925	0.739	0.734	0.798	0.697
749	0.916	0.778	0.719	0.804	0.706
750	0.919	0.767	0.731	0.805	0.707
751	0.910	0.814	0.725	0.817	0.725

752	0.902	0.770	0.719	0.797	0.695
753	0.931	0.801	0.731	0.821	0.731
754	0.910	0.793	0.710	0.805	0.707
755	0.925	0.793	0.728	0.815	0.722
756	0.934	0.762	0.710	0.801	0.702
757	0.899	0.755	0.713	0.788	0.682
758	0.916	0.796	0.725	0.813	0.718
759	0.902	0.809	0.772	0.828	0.741
760	0.905	0.783	0.754	0.813	0.720
761	0.936	0.806	0.749	0.830	0.745
762	0.908	0.783	0.731	0.807	0.710
763	0.905	0.786	0.749	0.813	0.719
764	0.922	0.801	0.757	0.827	0.740
765	0.919	0.773	0.737	0.809	0.713
766	0.910	0.793	0.731	0.812	0.717
767	0.919	0.786	0.701	0.802	0.703
768	0.922	0.783	0.760	0.821	0.731
769	0.908	0.778	0.754	0.813	0.719
770	0.922	0.796	0.713	0.811	0.715
771	0.905	0.798	0.719	0.808	0.711
772	0.910	0.739	0.722	0.789	0.684
773	0.922	0.806	0.746	0.825	0.737
774	0.893	0.762	0.772	0.808	0.712
775	0.925	0.793	0.749	0.822	0.733
776	0.919	0.796	0.754	0.823	0.734
777	0.905	0.786	0.728	0.806	0.709
778	0.922	0.811	0.740	0.825	0.737
779	0.925	0.798	0.716	0.813	0.720
780	0.905	0.783	0.734	0.807	0.710
781	0.910	0.783	0.713	0.802	0.703
782	0.902	0.742	0.737	0.792	0.688
783	0.928	0.793	0.731	0.817	0.725
784	0.902	0.786	0.751	0.813	0.719
785	0.899	0.788	0.749	0.812	0.717
786	0.910	0.749	0.722	0.793	0.689
787	0.913	0.811	0.731	0.819	0.728
788	0.913	0.770	0.737	0.806	0.709

**MCFS**					
**Numbers of features**	**G1**	**G2/M**	**S**	**ACC**	**MCC**
1	0.919	0.654	0.560	0.710	0.565
2	0.916	0.700	0.593	0.737	0.604
3	0.948	0.703	0.653	0.767	0.650

4	0.934	0.698	0.641	0.756	0.634
5	0.951	0.726	0.695	0.789	0.684
6	0.934	0.726	0.674	0.777	0.665
7	0.931	0.736	0.662	0.776	0.663
8	0.936	0.716	0.680	0.776	0.664
9	0.951	0.736	0.689	0.791	0.686
10	0.934	0.718	0.701	0.783	0.674
11	0.922	0.736	0.728	0.794	0.691
12	0.925	0.744	0.740	0.801	0.702
13	0.916	0.752	0.731	0.799	0.698
14	0.934	0.739	0.686	0.785	0.677
15	0.928	0.760	0.704	0.797	0.694
16	0.928	0.757	0.740	0.807	0.710
17	0.896	0.791	0.763	0.816	0.725
18	0.925	0.783	0.763	0.823	0.734
19	0.919	0.770	0.749	0.812	0.717
20	0.925	0.747	0.757	0.808	0.712
21	0.919	0.786	0.749	0.817	0.726
22	0.919	0.778	0.722	0.806	0.709
23	0.925	0.762	0.740	0.808	0.712
24	0.934	0.780	0.754	0.822	0.733
25	0.925	0.760	0.731	0.804	0.706
26	0.916	0.767	0.725	0.802	0.703
27	0.908	0.744	0.716	0.788	0.682
28	0.910	0.742	0.710	0.786	0.679
29	0.913	0.793	0.749	0.818	0.727
30	0.922	0.775	0.719	0.805	0.707
31	0.916	0.775	0.734	0.808	0.711
32	0.910	0.775	0.766	0.816	0.724
33	0.928	0.770	0.754	0.816	0.724
34	0.902	0.819	0.781	0.834	0.751
35	0.919	0.796	0.740	0.818	0.727
36	0.916	0.747	0.751	0.803	0.705
37	0.919	0.765	0.737	0.806	0.709
38	0.916	0.767	0.778	0.819	0.729
39	0.928	0.788	0.725	0.813	0.720
40	0.919	0.757	0.740	0.804	0.706
41	0.913	0.780	0.731	0.808	0.711
42	0.922	0.749	0.719	0.796	0.693
43	0.925	0.806	0.731	0.821	0.731
44	0.919	0.767	0.757	0.813	0.720
45	0.913	0.780	0.757	0.816	0.724

46	0.931	0.742	0.787	0.817	0.727
47	0.913	0.788	0.763	0.821	0.732
48	0.922	0.762	0.746	0.809	0.713
49	0.934	0.798	0.734	0.822	0.732
50	0.942	0.791	0.743	0.825	0.737
51	0.939	0.793	0.769	0.833	0.750
52	0.910	0.786	0.757	0.817	0.726
53	0.896	0.778	0.734	0.802	0.703
54	0.934	0.793	0.772	0.832	0.748
55	0.922	0.762	0.746	0.809	0.713
56	0.916	0.796	0.760	0.824	0.736
57	0.919	0.793	0.737	0.816	0.724
58	0.925	0.786	0.740	0.816	0.724
59	0.919	0.793	0.743	0.818	0.727
60	0.913	0.765	0.737	0.804	0.706
61	0.925	0.798	0.757	0.827	0.740
62	0.931	0.767	0.728	0.808	0.712
63	0.913	0.804	0.751	0.823	0.734
64	0.905	0.786	0.769	0.819	0.729
65	0.925	0.814	0.734	0.825	0.737
66	0.922	0.767	0.754	0.813	0.721
67	0.905	0.791	0.746	0.813	0.720
68	0.902	0.798	0.757	0.819	0.728
69	0.916	0.770	0.763	0.815	0.723
70	0.916	0.806	0.778	0.833	0.750
71	0.913	0.796	0.740	0.816	0.724
72	0.902	0.778	0.749	0.809	0.713
73	0.942	0.786	0.740	0.822	0.733
74	0.934	0.793	0.751	0.826	0.738
75	0.916	0.811	0.751	0.827	0.739
76	0.908	0.775	0.769	0.816	0.725
77	0.919	0.798	0.734	0.817	0.725
78	0.916	0.770	0.737	0.807	0.710
79	0.905	0.824	0.716	0.816	0.724
80	0.916	0.780	0.757	0.817	0.726
81	0.916	0.773	0.790	0.825	0.738
82	0.910	0.775	0.746	0.810	0.715
83	0.896	0.775	0.737	0.802	0.703
84	0.913	0.755	0.734	0.799	0.699
85	0.913	0.773	0.754	0.813	0.719
86	0.908	0.760	0.766	0.810	0.715
87	0.916	0.786	0.757	0.819	0.728

88	0.919	0.788	0.751	0.819	0.728
89	0.902	0.798	0.737	0.813	0.718
90	0.902	0.765	0.737	0.800	0.701
91	0.928	0.767	0.725	0.806	0.709
92	0.919	0.780	0.701	0.800	0.700
93	0.905	0.755	0.740	0.799	0.698
94	0.908	0.780	0.746	0.811	0.716
95	0.910	0.793	0.751	0.818	0.727
96	0.913	0.780	0.746	0.813	0.719
97	0.902	0.793	0.740	0.812	0.717
98	0.934	0.796	0.751	0.827	0.740
99	0.936	0.786	0.769	0.829	0.744
100	0.919	0.780	0.749	0.815	0.723
101	0.893	0.780	0.710	0.795	0.692
102	0.939	0.778	0.713	0.810	0.714
103	0.919	0.786	0.775	0.826	0.739
104	0.899	0.765	0.751	0.804	0.707
105	0.928	0.775	0.746	0.815	0.723
106	0.919	0.801	0.749	0.823	0.734
107	0.916	0.749	0.713	0.792	0.688
108	0.916	0.780	0.740	0.812	0.717
109	0.896	0.796	0.754	0.815	0.723
110	0.931	0.778	0.746	0.817	0.726
111	0.916	0.780	0.731	0.809	0.713
112	0.939	0.788	0.743	0.823	0.734
113	0.908	0.804	0.713	0.809	0.713
114	0.919	0.760	0.749	0.808	0.712
115	0.893	0.747	0.737	0.791	0.687
116	0.890	0.752	0.743	0.794	0.691
117	0.902	0.755	0.737	0.797	0.695
118	0.922	0.757	0.760	0.812	0.718
119	0.931	0.798	0.754	0.828	0.741
120	0.934	0.778	0.734	0.814	0.721
121	0.919	0.765	0.728	0.803	0.705
122	0.925	0.770	0.754	0.815	0.723
123	0.908	0.736	0.763	0.800	0.702
124	0.934	0.773	0.749	0.817	0.726
125	0.905	0.767	0.751	0.807	0.711
126	0.905	0.791	0.754	0.816	0.724
127	0.934	0.729	0.740	0.799	0.698
128	0.916	0.770	0.760	0.814	0.722
129	0.893	0.752	0.754	0.799	0.698

130	0.925	0.760	0.728	0.803	0.705
131	0.896	0.752	0.716	0.787	0.681
132	0.928	0.765	0.763	0.817	0.726
133	0.925	0.788	0.704	0.806	0.708
134	0.919	0.747	0.775	0.812	0.719
135	0.910	0.762	0.763	0.811	0.716
136	0.910	0.760	0.769	0.812	0.718
137	0.925	0.778	0.751	0.817	0.726
138	0.916	0.765	0.725	0.801	0.702
139	0.922	0.773	0.740	0.811	0.716
140	0.925	0.786	0.737	0.815	0.723
141	0.934	0.760	0.722	0.804	0.706
142	0.922	0.755	0.740	0.804	0.706
143	0.890	0.721	0.707	0.771	0.657
144	0.931	0.749	0.749	0.808	0.712
145	0.925	0.778	0.728	0.810	0.714
146	0.913	0.755	0.737	0.800	0.700
147	0.910	0.749	0.728	0.795	0.692
148	0.893	0.796	0.754	0.814	0.721
149	0.910	0.780	0.743	0.811	0.716
150	0.919	0.773	0.728	0.806	0.709
151	0.916	0.762	0.719	0.799	0.697
152	0.922	0.775	0.746	0.813	0.720
153	0.908	0.780	0.737	0.808	0.712
154	0.931	0.757	0.740	0.808	0.712
155	0.928	0.755	0.749	0.809	0.714
156	0.916	0.749	0.728	0.797	0.695
157	0.902	0.770	0.740	0.803	0.705
158	0.939	0.755	0.754	0.814	0.722
159	0.922	0.773	0.746	0.813	0.719
160	0.916	0.791	0.707	0.805	0.707
161	0.902	0.786	0.746	0.811	0.716
162	0.908	0.773	0.746	0.808	0.712
163	0.910	0.762	0.710	0.794	0.690
164	0.916	0.798	0.737	0.817	0.725
165	0.916	0.752	0.728	0.798	0.696
166	0.931	0.767	0.737	0.811	0.716
167	0.922	0.786	0.707	0.805	0.707
168	0.916	0.770	0.722	0.802	0.703
169	0.910	0.775	0.734	0.806	0.709
170	0.925	0.783	0.737	0.814	0.721
171	0.925	0.793	0.716	0.812	0.717

172	0.908	0.806	0.716	0.811	0.715
173	0.931	0.775	0.760	0.821	0.732
174	0.896	0.780	0.737	0.804	0.706
175	0.922	0.773	0.716	0.803	0.704
176	0.905	0.767	0.734	0.801	0.702
177	0.928	0.760	0.722	0.802	0.703
178	0.908	0.770	0.719	0.799	0.697
179	0.913	0.770	0.701	0.795	0.691
180	0.913	0.726	0.701	0.779	0.668
181	0.913	0.783	0.698	0.799	0.697
182	0.928	0.757	0.743	0.808	0.712
183	0.928	0.780	0.707	0.805	0.707
184	0.913	0.755	0.728	0.798	0.697
185	0.939	0.822	0.731	0.831	0.746
186	0.910	0.793	0.734	0.813	0.718
187	0.905	0.765	0.737	0.801	0.702
188	0.910	0.778	0.746	0.811	0.716
189	0.936	0.798	0.757	0.830	0.745
190	0.916	0.837	0.698	0.819	0.728
191	0.908	0.780	0.722	0.803	0.704
192	0.919	0.786	0.743	0.815	0.723
193	0.925	0.773	0.734	0.810	0.714
194	0.913	0.788	0.746	0.815	0.723
195	0.905	0.806	0.734	0.815	0.723
196	0.908	0.775	0.743	0.808	0.712
197	0.916	0.762	0.743	0.806	0.709
198	0.916	0.809	0.743	0.823	0.734
199	0.922	0.791	0.746	0.819	0.728
200	0.931	0.798	0.763	0.830	0.745
201	0.916	0.773	0.781	0.822	0.734
202	0.913	0.811	0.751	0.826	0.738
203	0.910	0.798	0.740	0.816	0.724
204	0.916	0.791	0.751	0.819	0.729
205	0.925	0.793	0.751	0.823	0.734
206	0.919	0.809	0.731	0.820	0.730
207	0.910	0.801	0.757	0.823	0.734
208	0.908	0.798	0.731	0.813	0.718
209	0.913	0.752	0.749	0.803	0.705
210	0.934	0.783	0.746	0.820	0.730
211	0.919	0.783	0.772	0.824	0.736
212	0.896	0.791	0.734	0.807	0.710
213	0.919	0.755	0.757	0.809	0.714

214	0.910	0.786	0.737	0.811	0.716
215	0.922	0.765	0.746	0.810	0.715
216	0.928	0.798	0.734	0.820	0.730
217	0.922	0.806	0.743	0.824	0.735
218	0.913	0.762	0.740	0.804	0.706
219	0.908	0.796	0.760	0.821	0.731
220	0.916	0.796	0.740	0.817	0.725
221	0.913	0.786	0.722	0.807	0.710
222	0.905	0.778	0.716	0.799	0.699
223	0.922	0.783	0.743	0.815	0.723
224	0.919	0.796	0.737	0.817	0.725
225	0.919	0.770	0.751	0.813	0.719
226	0.910	0.804	0.757	0.824	0.735
227	0.910	0.765	0.749	0.807	0.710
228	0.910	0.770	0.743	0.807	0.711
229	0.922	0.801	0.749	0.824	0.735
230	0.936	0.760	0.746	0.813	0.719
231	0.931	0.796	0.769	0.831	0.747
232	0.910	0.780	0.728	0.806	0.709
233	0.913	0.788	0.740	0.813	0.720
234	0.913	0.811	0.743	0.823	0.734
235	0.899	0.788	0.725	0.804	0.706
236	0.902	0.798	0.737	0.813	0.718
237	0.908	0.783	0.746	0.812	0.717
238	0.908	0.757	0.763	0.808	0.713
239	0.910	0.801	0.740	0.817	0.725
240	0.916	0.780	0.725	0.807	0.710
241	0.908	0.793	0.737	0.813	0.718
242	0.922	0.767	0.749	0.812	0.718
243	0.916	0.788	0.746	0.816	0.724
244	0.925	0.760	0.743	0.808	0.712
245	0.922	0.791	0.746	0.819	0.728
246	0.919	0.767	0.754	0.813	0.719
247	0.913	0.796	0.716	0.809	0.713
248	0.913	0.760	0.757	0.809	0.714
249	0.913	0.822	0.725	0.821	0.731
250	0.925	0.791	0.749	0.821	0.731
251	0.925	0.773	0.716	0.804	0.706
252	0.893	0.773	0.722	0.796	0.693
253	0.936	0.796	0.725	0.819	0.728
254	0.910	0.767	0.754	0.810	0.715
255	0.916	0.749	0.728	0.797	0.695

256	0.908	0.788	0.722	0.806	0.709
257	0.902	0.773	0.757	0.810	0.715
258	0.908	0.791	0.746	0.814	0.721
259	0.913	0.786	0.731	0.810	0.714
260	0.908	0.770	0.760	0.812	0.718
261	0.910	0.770	0.772	0.816	0.725
262	0.910	0.806	0.751	0.823	0.734
263	0.899	0.809	0.751	0.820	0.730
264	0.931	0.760	0.746	0.811	0.716
265	0.916	0.798	0.734	0.816	0.724
266	0.922	0.780	0.754	0.818	0.727
267	0.936	0.796	0.731	0.821	0.731
268	0.919	0.791	0.757	0.822	0.733
269	0.902	0.788	0.692	0.795	0.691
270	0.913	0.775	0.707	0.799	0.697
271	0.916	0.770	0.746	0.810	0.715
272	0.919	0.786	0.757	0.820	0.730
273	0.931	0.775	0.749	0.817	0.726
274	0.910	0.796	0.704	0.804	0.706
275	0.925	0.811	0.763	0.833	0.749
276	0.916	0.786	0.763	0.821	0.732
277	0.902	0.814	0.737	0.818	0.727
278	0.919	0.801	0.728	0.816	0.724
279	0.905	0.806	0.763	0.825	0.737
280	0.928	0.780	0.760	0.822	0.733
281	0.922	0.829	0.734	0.829	0.744
282	0.905	0.788	0.754	0.815	0.723
283	0.913	0.770	0.749	0.810	0.715
284	0.899	0.801	0.731	0.811	0.716
285	0.919	0.786	0.713	0.806	0.708
286	0.908	0.770	0.746	0.807	0.711
287	0.905	0.788	0.707	0.800	0.700
288	0.887	0.773	0.763	0.807	0.711
289	0.913	0.786	0.775	0.824	0.736
290	0.893	0.822	0.716	0.812	0.717
291	0.887	0.783	0.740	0.803	0.705
292	0.919	0.791	0.754	0.821	0.731
293	0.899	0.791	0.746	0.812	0.717
294	0.922	0.775	0.731	0.809	0.713
295	0.916	0.780	0.766	0.820	0.730
296	0.902	0.811	0.731	0.815	0.723
297	0.928	0.767	0.734	0.809	0.713

298	0.913	0.804	0.734	0.817	0.725
299	0.928	0.804	0.754	0.828	0.742
300	0.913	0.822	0.778	0.838	0.756
301	0.922	0.804	0.766	0.830	0.745
302	0.916	0.806	0.734	0.819	0.728
303	0.919	0.822	0.719	0.821	0.731
304	0.905	0.755	0.731	0.796	0.694
305	0.896	0.819	0.734	0.817	0.725
306	0.913	0.783	0.728	0.808	0.711
307	0.887	0.796	0.772	0.818	0.727
308	0.913	0.804	0.754	0.824	0.735
309	0.910	0.775	0.740	0.808	0.712
310	0.928	0.778	0.760	0.821	0.732
311	0.922	0.804	0.746	0.824	0.735
312	0.916	0.791	0.719	0.809	0.713
313	0.925	0.783	0.710	0.806	0.708
314	0.919	0.767	0.722	0.802	0.703
315	0.905	0.783	0.731	0.806	0.709
316	0.919	0.791	0.731	0.813	0.720
317	0.893	0.793	0.772	0.819	0.729
318	0.908	0.809	0.784	0.833	0.750
319	0.913	0.817	0.751	0.828	0.741
320	0.919	0.796	0.760	0.825	0.737
321	0.908	0.788	0.743	0.813	0.719
322	0.931	0.767	0.737	0.811	0.716
323	0.910	0.814	0.746	0.824	0.735
324	0.928	0.770	0.743	0.813	0.719
325	0.899	0.770	0.716	0.795	0.692
326	0.910	0.778	0.713	0.800	0.700
327	0.934	0.801	0.766	0.833	0.749
328	0.919	0.765	0.763	0.814	0.722
329	0.910	0.788	0.731	0.810	0.714
330	0.905	0.796	0.734	0.812	0.717
331	0.922	0.791	0.737	0.816	0.724
332	0.919	0.822	0.760	0.834	0.751
333	0.913	0.786	0.749	0.815	0.723
334	0.913	0.760	0.754	0.808	0.712
335	0.931	0.804	0.757	0.830	0.745
336	0.916	0.806	0.740	0.821	0.731
337	0.882	0.824	0.743	0.817	0.726
338	0.928	0.804	0.725	0.819	0.728
339	0.893	0.786	0.743	0.807	0.710

340	0.896	0.788	0.751	0.812	0.717
341	0.919	0.780	0.740	0.813	0.719
342	0.919	0.762	0.746	0.808	0.712
343	0.910	0.804	0.737	0.817	0.725
344	0.902	0.798	0.766	0.822	0.733
345	0.916	0.809	0.737	0.821	0.731
346	0.902	0.806	0.763	0.824	0.735
347	0.899	0.788	0.757	0.814	0.722
348	0.922	0.811	0.734	0.823	0.734
349	0.893	0.767	0.734	0.798	0.696
350	0.908	0.788	0.740	0.812	0.717
351	0.905	0.788	0.766	0.819	0.729
352	0.916	0.786	0.751	0.817	0.726
353	0.893	0.780	0.731	0.801	0.702
354	0.925	0.801	0.725	0.817	0.725
355	0.908	0.765	0.754	0.808	0.712
356	0.908	0.798	0.740	0.815	0.723
357	0.922	0.775	0.751	0.815	0.723
358	0.922	0.778	0.740	0.813	0.719
359	0.922	0.811	0.728	0.821	0.731
360	0.899	0.757	0.781	0.811	0.717
361	0.910	0.819	0.728	0.820	0.729
362	0.908	0.809	0.763	0.827	0.740
363	0.905	0.809	0.728	0.814	0.721
364	0.916	0.788	0.746	0.816	0.724
365	0.890	0.814	0.784	0.829	0.744
366	0.913	0.791	0.719	0.808	0.711
367	0.910	0.811	0.743	0.822	0.732
368	0.910	0.801	0.743	0.818	0.727
369	0.916	0.783	0.772	0.823	0.734
370	0.913	0.801	0.740	0.818	0.727
371	0.905	0.796	0.734	0.812	0.717
372	0.910	0.817	0.754	0.828	0.741
373	0.910	0.783	0.746	0.813	0.718
374	0.905	0.773	0.754	0.810	0.715
375	0.902	0.773	0.749	0.807	0.710
376	0.905	0.798	0.746	0.816	0.724
377	0.922	0.767	0.749	0.812	0.718
378	0.905	0.767	0.740	0.803	0.705
379	0.913	0.793	0.734	0.813	0.720
380	0.905	0.796	0.725	0.809	0.713
381	0.936	0.780	0.751	0.822	0.733

382	0.919	0.788	0.734	0.813	0.720
383	0.890	0.775	0.737	0.800	0.700
384	0.925	0.788	0.766	0.826	0.738
385	0.908	0.783	0.737	0.809	0.713
386	0.902	0.793	0.746	0.813	0.720
387	0.905	0.780	0.754	0.813	0.719
388	0.908	0.809	0.740	0.819	0.728
389	0.934	0.786	0.751	0.823	0.734
390	0.913	0.796	0.731	0.813	0.720
391	0.890	0.780	0.781	0.816	0.725
392	0.902	0.796	0.749	0.815	0.723
393	0.910	0.809	0.725	0.815	0.722
394	0.928	0.783	0.716	0.809	0.713
395	0.902	0.783	0.751	0.812	0.717
396	0.913	0.801	0.731	0.815	0.723
397	0.913	0.824	0.713	0.818	0.727
398	0.899	0.778	0.769	0.814	0.722
399	0.908	0.806	0.740	0.818	0.727
400	0.910	0.749	0.737	0.798	0.697
401	0.893	0.801	0.751	0.815	0.723
402	0.913	0.793	0.731	0.813	0.718
403	0.902	0.796	0.749	0.815	0.723
404	0.922	0.819	0.746	0.829	0.744
405	0.899	0.796	0.781	0.825	0.737
406	0.910	0.817	0.769	0.832	0.748
407	0.908	0.791	0.737	0.812	0.717
408	0.890	0.775	0.746	0.803	0.705
409	0.908	0.786	0.737	0.810	0.714
410	0.916	0.793	0.737	0.815	0.723
411	0.916	0.801	0.754	0.824	0.735
412	0.896	0.783	0.743	0.807	0.710
413	0.908	0.804	0.737	0.816	0.724
414	0.910	0.778	0.757	0.814	0.721
415	0.925	0.780	0.749	0.817	0.726
416	0.919	0.793	0.716	0.810	0.714
417	0.908	0.806	0.740	0.818	0.727
418	0.902	0.786	0.778	0.821	0.732
419	0.902	0.796	0.757	0.818	0.727
420	0.899	0.762	0.754	0.804	0.707
421	0.916	0.780	0.766	0.820	0.730
422	0.925	0.788	0.757	0.823	0.734
423	0.913	0.814	0.731	0.820	0.729

424	0.925	0.767	0.757	0.815	0.723
425	0.913	0.791	0.760	0.821	0.731
426	0.908	0.796	0.781	0.828	0.741
427	0.902	0.778	0.740	0.806	0.709
428	0.905	0.791	0.731	0.809	0.713
429	0.913	0.801	0.754	0.823	0.734
430	0.910	0.775	0.781	0.821	0.732
431	0.908	0.824	0.737	0.824	0.735
432	0.910	0.783	0.763	0.818	0.727
433	0.908	0.783	0.757	0.815	0.723
434	0.910	0.804	0.737	0.817	0.725
435	0.899	0.793	0.760	0.817	0.726
436	0.902	0.778	0.722	0.800	0.700
437	0.913	0.770	0.725	0.802	0.703
438	0.925	0.773	0.719	0.805	0.707
439	0.905	0.809	0.704	0.807	0.710
440	0.896	0.796	0.749	0.813	0.720
441	0.913	0.760	0.754	0.808	0.712
442	0.922	0.798	0.701	0.808	0.711
443	0.925	0.788	0.737	0.816	0.724
444	0.916	0.773	0.707	0.799	0.697
445	0.922	0.775	0.746	0.813	0.720
446	0.870	0.796	0.757	0.808	0.712
447	0.925	0.786	0.725	0.812	0.717
448	0.899	0.829	0.731	0.821	0.731
449	0.908	0.783	0.754	0.814	0.722
450	0.910	0.773	0.760	0.813	0.721
451	0.916	0.767	0.769	0.816	0.725
452	0.896	0.809	0.740	0.815	0.723
453	0.902	0.778	0.737	0.805	0.707
454	0.910	0.804	0.775	0.829	0.744
455	0.910	0.793	0.725	0.810	0.714
456	0.919	0.793	0.740	0.817	0.726
457	0.922	0.783	0.743	0.815	0.723
458	0.916	0.814	0.722	0.818	0.727
459	0.919	0.780	0.754	0.817	0.726
460	0.905	0.793	0.740	0.813	0.718
461	0.919	0.770	0.710	0.799	0.699
462	0.910	0.809	0.746	0.822	0.732
463	0.910	0.806	0.728	0.815	0.722
464	0.908	0.829	0.772	0.837	0.755
465	0.910	0.804	0.757	0.824	0.735

466	0.896	0.780	0.772	0.815	0.723
467	0.916	0.811	0.722	0.817	0.725
468	0.919	0.783	0.769	0.823	0.734
469	0.896	0.786	0.751	0.811	0.716
470	0.919	0.791	0.737	0.815	0.723
471	0.913	0.780	0.749	0.813	0.720
472	0.922	0.773	0.734	0.809	0.713
473	0.922	0.801	0.746	0.823	0.734
474	0.916	0.775	0.728	0.806	0.709
475	0.928	0.804	0.781	0.837	0.755
476	0.925	0.767	0.769	0.819	0.729
477	0.905	0.817	0.707	0.811	0.715
478	0.893	0.814	0.760	0.823	0.734
479	0.908	0.850	0.763	0.842	0.762
480	0.908	0.788	0.737	0.811	0.716
481	0.916	0.819	0.740	0.826	0.738
482	0.910	0.770	0.725	0.801	0.702
483	0.910	0.798	0.743	0.817	0.726
484	0.922	0.793	0.772	0.828	0.743
485	0.916	0.798	0.781	0.831	0.747
486	0.908	0.762	0.734	0.800	0.701
487	0.916	0.801	0.766	0.828	0.741
488	0.925	0.798	0.754	0.826	0.738
489	0.910	0.773	0.743	0.808	0.712
490	0.916	0.817	0.725	0.820	0.729
491	0.928	0.786	0.740	0.817	0.726
492	0.928	0.801	0.746	0.825	0.737
493	0.922	0.793	0.710	0.809	0.713
494	0.908	0.786	0.754	0.815	0.723
495	0.908	0.806	0.778	0.830	0.746
496	0.913	0.791	0.704	0.803	0.704
497	0.913	0.801	0.746	0.820	0.730
498	0.893	0.811	0.746	0.817	0.725
499	0.928	0.796	0.725	0.816	0.724
500	0.922	0.798	0.746	0.822	0.732
501	0.902	0.806	0.781	0.829	0.744
502	0.908	0.788	0.734	0.810	0.714
503	0.905	0.811	0.743	0.820	0.730
504	0.928	0.757	0.781	0.820	0.731
505	0.922	0.804	0.760	0.828	0.742
506	0.919	0.786	0.722	0.809	0.713
507	0.931	0.765	0.751	0.814	0.722

508	0.913	0.798	0.749	0.820	0.730
509	0.908	0.801	0.781	0.829	0.744
510	0.919	0.786	0.746	0.816	0.724
511	0.902	0.832	0.719	0.819	0.728
512	0.899	0.793	0.716	0.803	0.704
513	0.922	0.814	0.740	0.826	0.738
514	0.908	0.806	0.746	0.820	0.730
515	0.902	0.796	0.734	0.811	0.716
516	0.902	0.806	0.746	0.818	0.727
517	0.905	0.819	0.710	0.813	0.718
518	0.922	0.804	0.722	0.816	0.724
519	0.913	0.786	0.766	0.821	0.731
520	0.913	0.786	0.728	0.809	0.713
521	0.910	0.811	0.746	0.823	0.734
522	0.899	0.775	0.769	0.813	0.721
523	0.928	0.773	0.772	0.823	0.735
524	0.910	0.809	0.731	0.817	0.725
525	0.908	0.783	0.722	0.804	0.706
526	0.931	0.811	0.716	0.820	0.729
527	0.908	0.767	0.769	0.813	0.721
528	0.913	0.765	0.749	0.808	0.712
529	0.913	0.770	0.722	0.801	0.702
530	0.902	0.832	0.731	0.823	0.734
531	0.905	0.817	0.722	0.815	0.722
532	0.908	0.788	0.743	0.813	0.719
533	0.913	0.822	0.734	0.824	0.735
534	0.902	0.804	0.763	0.823	0.734
535	0.902	0.804	0.740	0.815	0.722
536	0.893	0.801	0.740	0.812	0.717
537	0.916	0.801	0.740	0.819	0.728
538	0.905	0.773	0.766	0.813	0.721
539	0.913	0.773	0.734	0.806	0.709
540	0.896	0.767	0.781	0.813	0.721
541	0.922	0.752	0.775	0.814	0.723
542	0.899	0.791	0.728	0.806	0.709
543	0.910	0.809	0.749	0.823	0.734
544	0.919	0.788	0.757	0.821	0.731
545	0.902	0.791	0.731	0.808	0.711
546	0.905	0.765	0.772	0.813	0.720
547	0.910	0.791	0.725	0.809	0.713
548	0.916	0.798	0.737	0.817	0.725
549	0.899	0.765	0.781	0.813	0.721

550	0.913	0.806	0.749	0.823	0.734
551	0.910	0.780	0.763	0.817	0.726
552	0.908	0.773	0.731	0.803	0.705
553	0.919	0.791	0.757	0.822	0.733
554	0.899	0.801	0.746	0.815	0.723
555	0.908	0.767	0.769	0.813	0.721
556	0.919	0.791	0.757	0.822	0.733
557	0.916	0.786	0.746	0.815	0.723
558	0.931	0.806	0.754	0.830	0.745
559	0.910	0.780	0.760	0.816	0.724
560	0.919	0.783	0.787	0.828	0.744
561	0.899	0.760	0.725	0.794	0.690
562	0.919	0.760	0.731	0.802	0.703
563	0.919	0.819	0.766	0.835	0.752
564	0.916	0.786	0.737	0.813	0.718
565	0.913	0.786	0.716	0.805	0.707
566	0.925	0.796	0.749	0.823	0.734
567	0.908	0.796	0.722	0.809	0.713
568	0.905	0.786	0.728	0.806	0.708
569	0.902	0.809	0.743	0.818	0.727
570	0.922	0.793	0.772	0.828	0.743
571	0.919	0.780	0.781	0.826	0.739
572	0.905	0.806	0.731	0.814	0.721
573	0.908	0.788	0.746	0.813	0.720
574	0.919	0.767	0.731	0.805	0.707
575	0.916	0.780	0.707	0.801	0.701
576	0.922	0.814	0.775	0.837	0.755
577	0.913	0.791	0.749	0.817	0.726
578	0.913	0.783	0.760	0.818	0.727
579	0.908	0.804	0.743	0.818	0.727
580	0.882	0.804	0.781	0.822	0.733
581	0.919	0.804	0.772	0.831	0.747
582	0.925	0.755	0.763	0.813	0.719
583	0.896	0.773	0.728	0.799	0.697
584	0.916	0.804	0.757	0.826	0.738
585	0.902	0.760	0.725	0.795	0.692
586	0.916	0.780	0.746	0.813	0.720
587	0.922	0.796	0.751	0.823	0.734
588	0.910	0.798	0.749	0.819	0.728
589	0.908	0.791	0.751	0.816	0.724
590	0.913	0.767	0.754	0.811	0.716
591	0.910	0.783	0.722	0.805	0.707

592	0.928	0.809	0.751	0.829	0.744
593	0.896	0.783	0.746	0.808	0.712
594	0.928	0.773	0.746	0.814	0.722
595	0.919	0.822	0.751	0.831	0.746
596	0.916	0.767	0.722	0.801	0.702
597	0.916	0.793	0.775	0.828	0.741
598	0.922	0.806	0.740	0.823	0.734
599	0.913	0.809	0.743	0.822	0.732
600	0.925	0.778	0.734	0.812	0.717
601	0.928	0.804	0.734	0.822	0.732
602	0.928	0.796	0.787	0.836	0.754
603	0.919	0.806	0.749	0.825	0.737
604	0.916	0.786	0.728	0.810	0.714
605	0.902	0.757	0.799	0.817	0.728
606	0.922	0.786	0.719	0.809	0.713
607	0.928	0.760	0.728	0.804	0.706
608	0.922	0.762	0.757	0.813	0.719
609	0.913	0.796	0.719	0.810	0.714
610	0.916	0.780	0.737	0.811	0.716
611	0.910	0.817	0.722	0.817	0.725
612	0.919	0.760	0.740	0.805	0.708
613	0.928	0.773	0.728	0.809	0.713
614	0.922	0.832	0.749	0.835	0.752
615	0.928	0.791	0.722	0.813	0.720
616	0.928	0.817	0.743	0.829	0.744
617	0.913	0.786	0.740	0.813	0.719
618	0.908	0.811	0.749	0.823	0.734
619	0.913	0.773	0.728	0.804	0.706
620	0.919	0.796	0.763	0.826	0.738
621	0.910	0.780	0.707	0.799	0.699
622	0.916	0.788	0.746	0.816	0.724
623	0.934	0.793	0.749	0.825	0.737
624	0.925	0.765	0.751	0.813	0.719
625	0.910	0.793	0.746	0.816	0.724
626	0.916	0.780	0.781	0.825	0.737
627	0.925	0.762	0.760	0.814	0.722
628	0.928	0.778	0.769	0.824	0.736
629	0.910	0.762	0.725	0.799	0.698
630	0.913	0.806	0.728	0.816	0.724
631	0.916	0.767	0.731	0.804	0.706
632	0.925	0.804	0.737	0.822	0.733
633	0.908	0.780	0.746	0.811	0.716

634	0.908	0.770	0.737	0.804	0.706
635	0.908	0.786	0.734	0.809	0.713
636	0.910	0.778	0.728	0.805	0.707
637	0.910	0.793	0.731	0.812	0.717
638	0.925	0.770	0.728	0.807	0.710
639	0.890	0.773	0.754	0.805	0.708
640	0.910	0.814	0.740	0.822	0.732
641	0.905	0.788	0.769	0.820	0.730
642	0.899	0.827	0.751	0.827	0.739
643	0.896	0.778	0.737	0.803	0.704
644	0.913	0.762	0.746	0.806	0.709
645	0.919	0.791	0.746	0.818	0.727
646	0.913	0.798	0.757	0.823	0.734
647	0.913	0.773	0.746	0.810	0.715
648	0.916	0.791	0.769	0.825	0.737
649	0.919	0.773	0.725	0.805	0.707
650	0.922	0.796	0.751	0.823	0.734
651	0.916	0.786	0.740	0.813	0.720
652	0.936	0.806	0.734	0.826	0.738
653	0.931	0.783	0.731	0.814	0.721
654	0.919	0.762	0.707	0.796	0.693
655	0.899	0.793	0.734	0.809	0.713
656	0.910	0.788	0.725	0.808	0.711
657	0.942	0.835	0.731	0.837	0.755
658	0.910	0.793	0.763	0.822	0.733
659	0.908	0.791	0.751	0.816	0.724
660	0.902	0.796	0.757	0.818	0.727
661	0.910	0.783	0.746	0.813	0.719
662	0.922	0.773	0.731	0.808	0.711
663	0.910	0.783	0.707	0.800	0.700
664	0.905	0.798	0.754	0.819	0.728
665	0.899	0.780	0.734	0.804	0.706
666	0.931	0.783	0.760	0.824	0.736
667	0.919	0.783	0.737	0.813	0.719
668	0.905	0.791	0.760	0.818	0.727
669	0.919	0.762	0.749	0.809	0.714
670	0.922	0.775	0.751	0.815	0.723
671	0.922	0.775	0.731	0.809	0.713
672	0.922	0.811	0.713	0.816	0.724
673	0.910	0.791	0.763	0.821	0.731
674	0.913	0.793	0.728	0.812	0.717
675	0.919	0.749	0.751	0.805	0.708

676	0.948	0.811	0.719	0.827	0.739
677	0.910	0.780	0.754	0.814	0.722
678	0.931	0.783	0.763	0.825	0.737
679	0.934	0.778	0.734	0.814	0.721
680	0.916	0.770	0.751	0.812	0.718
681	0.899	0.783	0.716	0.799	0.699
682	0.905	0.804	0.740	0.816	0.724
683	0.910	0.817	0.725	0.818	0.727
684	0.934	0.811	0.751	0.832	0.748
685	0.928	0.780	0.725	0.811	0.716
686	0.925	0.809	0.754	0.829	0.744
687	0.931	0.752	0.734	0.804	0.707
688	0.919	0.811	0.751	0.828	0.741
689	0.919	0.788	0.743	0.816	0.724
690	0.934	0.788	0.737	0.819	0.728
691	0.913	0.780	0.743	0.812	0.717
692	0.896	0.786	0.757	0.813	0.719
693	0.922	0.798	0.787	0.835	0.753
694	0.922	0.770	0.737	0.809	0.713
695	0.925	0.793	0.772	0.829	0.744
696	0.913	0.775	0.713	0.800	0.700
697	0.931	0.778	0.725	0.811	0.716
698	0.928	0.819	0.743	0.830	0.745
699	0.913	0.788	0.754	0.818	0.727
700	0.913	0.788	0.743	0.814	0.721
701	0.916	0.822	0.743	0.828	0.741
702	0.913	0.767	0.754	0.811	0.716
703	0.919	0.801	0.737	0.819	0.728
704	0.910	0.822	0.713	0.816	0.724
705	0.919	0.778	0.754	0.816	0.725
706	0.928	0.788	0.737	0.817	0.726
707	0.919	0.757	0.772	0.814	0.722
708	0.922	0.762	0.746	0.809	0.713
709	0.916	0.775	0.737	0.809	0.713
710	0.916	0.775	0.766	0.818	0.727
711	0.919	0.773	0.725	0.805	0.707
712	0.931	0.806	0.749	0.828	0.742
713	0.925	0.811	0.760	0.832	0.748
714	0.908	0.806	0.731	0.815	0.723
715	0.922	0.788	0.716	0.809	0.713
716	0.919	0.798	0.719	0.813	0.718
717	0.916	0.788	0.710	0.805	0.707

718	0.922	0.791	0.713	0.809	0.713
719	0.925	0.760	0.725	0.802	0.703
720	0.910	0.788	0.713	0.804	0.706
721	0.910	0.765	0.760	0.811	0.716
722	0.919	0.804	0.728	0.817	0.725
723	0.936	0.793	0.766	0.831	0.747
724	0.910	0.786	0.740	0.812	0.717
725	0.928	0.783	0.746	0.818	0.727
726	0.934	0.804	0.746	0.828	0.741
727	0.908	0.767	0.769	0.813	0.721
728	0.908	0.804	0.743	0.818	0.727
729	0.922	0.814	0.754	0.830	0.745
730	0.916	0.786	0.754	0.818	0.727
731	0.913	0.752	0.769	0.810	0.716
732	0.910	0.819	0.749	0.827	0.739
733	0.919	0.806	0.751	0.826	0.738
734	0.919	0.786	0.728	0.811	0.716
735	0.908	0.817	0.763	0.829	0.744
736	0.908	0.806	0.751	0.822	0.733
737	0.913	0.752	0.740	0.800	0.701
738	0.896	0.783	0.731	0.803	0.705
739	0.925	0.786	0.704	0.805	0.707
740	0.905	0.798	0.734	0.813	0.718
741	0.928	0.767	0.754	0.815	0.723
742	0.910	0.767	0.740	0.805	0.708
743	0.905	0.778	0.737	0.806	0.709
744	0.913	0.752	0.751	0.804	0.707
745	0.916	0.793	0.731	0.813	0.720
746	0.913	0.780	0.713	0.802	0.703
747	0.905	0.796	0.722	0.808	0.711
748	0.899	0.793	0.734	0.809	0.713
749	0.922	0.778	0.713	0.804	0.706
750	0.925	0.780	0.746	0.816	0.724
751	0.928	0.783	0.740	0.816	0.724
752	0.908	0.775	0.740	0.807	0.710
753	0.922	0.778	0.722	0.807	0.710
754	0.934	0.796	0.754	0.828	0.741
755	0.922	0.783	0.749	0.817	0.726
756	0.942	0.804	0.763	0.836	0.754
757	0.908	0.804	0.763	0.825	0.737
758	0.913	0.775	0.763	0.816	0.725
759	0.916	0.778	0.728	0.807	0.710

760	0.934	0.757	0.751	0.813	0.719
761	0.902	0.780	0.722	0.801	0.702
762	0.925	0.793	0.731	0.816	0.724
763	0.919	0.783	0.757	0.819	0.729
764	0.919	0.752	0.725	0.798	0.696
765	0.922	0.796	0.728	0.815	0.722
766	0.916	0.755	0.725	0.798	0.696
767	0.928	0.798	0.731	0.819	0.728
768	0.922	0.796	0.760	0.826	0.738
769	0.922	0.801	0.716	0.813	0.720
770	0.916	0.783	0.737	0.812	0.717
771	0.908	0.809	0.716	0.812	0.717
772	0.913	0.775	0.731	0.806	0.709
773	0.925	0.767	0.778	0.822	0.734
774	0.910	0.778	0.743	0.810	0.714
775	0.925	0.775	0.731	0.810	0.714
776	0.925	0.796	0.743	0.821	0.731
777	0.916	0.809	0.737	0.821	0.731
778	0.922	0.778	0.722	0.807	0.710
779	0.934	0.780	0.769	0.827	0.740
780	0.910	0.786	0.725	0.807	0.710
781	0.916	0.778	0.760	0.817	0.726
782	0.899	0.773	0.751	0.807	0.711
783	0.928	0.801	0.713	0.814	0.721
784	0.913	0.793	0.760	0.822	0.733
785	0.919	0.791	0.725	0.812	0.717
786	0.916	0.783	0.740	0.813	0.719
787	0.913	0.822	0.749	0.828	0.742
788	0.905	0.767	0.734	0.801	0.702
**SHAP by lightGBM**					
**Numbers of features**	**G1**	**G2/M**	**S**	**ACC**	**MCC**
1	0.913	0.638	0.554	0.701	0.551
2	0.942	0.698	0.635	0.757	0.635
3	0.928	0.721	0.611	0.754	0.629
4	0.931	0.752	0.689	0.790	0.685
5	0.931	0.718	0.704	0.783	0.674
6	0.942	0.770	0.728	0.813	0.719
7	0.919	0.827	0.763	0.837	0.755
8	0.908	0.832	0.763	0.835	0.752
9	0.931	0.801	0.790	0.840	0.760
10	0.922	0.788	0.751	0.820	0.730
11	0.922	0.796	0.751	0.823	0.734

12	0.913	0.798	0.775	0.828	0.743
13	0.908	0.793	0.754	0.818	0.727
14	0.931	0.801	0.746	0.826	0.738
15	0.913	0.791	0.790	0.830	0.746
16	0.902	0.814	0.769	0.828	0.743
17	0.919	0.809	0.763	0.830	0.745
18	0.925	0.814	0.760	0.833	0.749
19	0.934	0.775	0.772	0.826	0.739
20	0.919	0.765	0.728	0.803	0.705
21	0.925	0.786	0.749	0.819	0.729
22	0.931	0.801	0.772	0.834	0.751
23	0.939	0.755	0.766	0.818	0.728
24	0.931	0.773	0.778	0.826	0.739
25	0.934	0.822	0.737	0.831	0.746
26	0.928	0.811	0.790	0.843	0.764
27	0.951	0.788	0.760	0.832	0.748
28	0.928	0.827	0.781	0.845	0.768
29	0.905	0.809	0.757	0.824	0.735
30	0.919	0.786	0.793	0.831	0.748
31	0.925	0.791	0.746	0.820	0.730
32	0.919	0.817	0.766	0.834	0.751
33	0.922	0.778	0.749	0.815	0.723
34	0.934	0.783	0.757	0.824	0.736
35	0.931	0.840	0.743	0.839	0.758
36	0.936	0.824	0.746	0.836	0.753
37	0.919	0.806	0.766	0.830	0.745
38	0.922	0.835	0.731	0.830	0.745
39	0.936	0.827	0.769	0.844	0.766
40	0.908	0.814	0.749	0.824	0.735
41	0.916	0.835	0.763	0.839	0.758
42	0.939	0.819	0.749	0.836	0.753
43	0.925	0.829	0.784	0.846	0.769
44	0.925	0.793	0.737	0.818	0.727
45	0.928	0.824	0.746	0.833	0.749
46	0.925	0.822	0.769	0.839	0.758
47	0.919	0.817	0.746	0.828	0.741
48	0.931	0.806	0.754	0.830	0.745
49	0.913	0.814	0.754	0.828	0.741
50	0.934	0.791	0.728	0.817	0.725
51	0.936	0.827	0.760	0.842	0.762
52	0.919	0.845	0.746	0.838	0.756
53	0.925	0.809	0.769	0.834	0.751

54	0.922	0.809	0.763	0.831	0.747
55	0.934	0.811	0.760	0.835	0.752
56	0.922	0.809	0.719	0.817	0.725
57	0.925	0.824	0.737	0.829	0.744
58	0.919	0.804	0.737	0.820	0.730
59	0.908	0.829	0.734	0.825	0.736
60	0.919	0.824	0.763	0.836	0.754
61	0.908	0.804	0.760	0.824	0.736
62	0.928	0.814	0.775	0.839	0.758
63	0.916	0.817	0.754	0.829	0.744
64	0.908	0.822	0.763	0.831	0.747
65	0.910	0.824	0.769	0.835	0.752
66	0.928	0.824	0.766	0.840	0.759
67	0.913	0.827	0.787	0.843	0.764
68	0.925	0.829	0.772	0.843	0.763
69	0.916	0.809	0.749	0.825	0.737
70	0.925	0.824	0.731	0.828	0.741
71	0.908	0.809	0.769	0.828	0.742
72	0.934	0.842	0.740	0.840	0.759
73	0.908	0.798	0.754	0.820	0.730
74	0.908	0.775	0.751	0.811	0.716
75	0.928	0.840	0.781	0.850	0.775
76	0.922	0.848	0.796	0.856	0.783
77	0.916	0.791	0.751	0.819	0.728
78	0.913	0.819	0.781	0.838	0.757
79	0.910	0.814	0.772	0.832	0.748
80	0.922	0.788	0.760	0.823	0.734
81	0.916	0.811	0.763	0.830	0.745
82	0.934	0.827	0.784	0.848	0.772
83	0.931	0.814	0.778	0.841	0.761
84	0.931	0.817	0.749	0.832	0.748
85	0.905	0.814	0.775	0.831	0.747
86	0.919	0.827	0.778	0.842	0.762
87	0.934	0.809	0.778	0.840	0.760
88	0.908	0.835	0.754	0.833	0.749
89	0.908	0.804	0.781	0.830	0.746
90	0.908	0.827	0.766	0.834	0.751
91	0.908	0.822	0.781	0.837	0.755
92	0.913	0.822	0.784	0.840	0.759
93	0.908	0.832	0.763	0.835	0.752
94	0.928	0.796	0.754	0.826	0.738
95	0.931	0.780	0.749	0.819	0.729

96	0.905	0.804	0.754	0.821	0.731
97	0.919	0.822	0.763	0.835	0.752
98	0.919	0.796	0.766	0.827	0.740
99	0.896	0.809	0.743	0.816	0.724
100	0.902	0.837	0.784	0.842	0.762
101	0.919	0.809	0.751	0.827	0.740
102	0.910	0.837	0.793	0.847	0.771
103	0.913	0.822	0.835	0.856	0.784
104	0.934	0.811	0.772	0.839	0.758
105	0.922	0.832	0.796	0.850	0.775
106	0.913	0.791	0.740	0.814	0.721
107	0.913	0.845	0.793	0.851	0.776
108	0.916	0.822	0.775	0.838	0.756
109	0.922	0.801	0.775	0.832	0.748
110	0.934	0.817	0.760	0.837	0.755
111	0.922	0.814	0.757	0.831	0.747
112	0.922	0.773	0.796	0.828	0.744
113	0.910	0.817	0.751	0.827	0.739
114	0.913	0.765	0.781	0.818	0.728
115	0.913	0.827	0.760	0.834	0.751
116	0.908	0.832	0.784	0.842	0.762
117	0.910	0.814	0.784	0.836	0.754
118	0.916	0.837	0.784	0.846	0.769
119	0.896	0.811	0.731	0.813	0.720
120	0.910	0.835	0.760	0.836	0.753
121	0.908	0.804	0.802	0.837	0.756
122	0.922	0.780	0.749	0.816	0.724
123	0.905	0.793	0.763	0.820	0.730
124	0.899	0.801	0.796	0.831	0.747
125	0.910	0.829	0.775	0.839	0.758
126	0.905	0.801	0.781	0.828	0.743
127	0.910	0.832	0.772	0.839	0.758
128	0.934	0.804	0.784	0.840	0.760
129	0.916	0.824	0.787	0.843	0.764
130	0.922	0.780	0.757	0.819	0.729
131	0.922	0.824	0.769	0.839	0.758
132	0.928	0.798	0.728	0.818	0.727
133	0.925	0.778	0.757	0.819	0.729
134	0.910	0.822	0.766	0.833	0.749
135	0.896	0.832	0.731	0.821	0.731
136	0.896	0.822	0.781	0.833	0.750
137	0.916	0.837	0.793	0.849	0.773

138	0.913	0.814	0.760	0.829	0.744
139	0.899	0.796	0.746	0.813	0.720
140	0.931	0.814	0.778	0.841	0.761
141	0.908	0.827	0.766	0.834	0.751
142	0.913	0.837	0.772	0.842	0.762
143	0.916	0.829	0.757	0.835	0.752
144	0.928	0.837	0.769	0.845	0.768
145	0.913	0.845	0.784	0.848	0.772
146	0.922	0.829	0.772	0.842	0.762
147	0.902	0.806	0.754	0.821	0.731
148	0.893	0.824	0.757	0.826	0.738
149	0.908	0.806	0.784	0.832	0.748
150	0.928	0.835	0.760	0.842	0.762
151	0.919	0.848	0.763	0.844	0.766
152	0.931	0.809	0.737	0.826	0.738
153	0.910	0.829	0.787	0.843	0.764
154	0.925	0.832	0.772	0.843	0.765
155	0.922	0.798	0.754	0.825	0.737
156	0.908	0.819	0.763	0.830	0.745
157	0.902	0.814	0.769	0.828	0.743
158	0.908	0.835	0.734	0.827	0.739
159	0.913	0.832	0.743	0.830	0.745
160	0.916	0.819	0.760	0.832	0.748
161	0.916	0.814	0.775	0.835	0.752
162	0.902	0.804	0.743	0.816	0.724
163	0.899	0.832	0.760	0.831	0.746
164	0.902	0.822	0.731	0.819	0.728
165	0.905	0.804	0.760	0.823	0.734
166	0.919	0.796	0.769	0.828	0.741
167	0.913	0.824	0.763	0.834	0.751
168	0.908	0.796	0.751	0.818	0.727
169	0.922	0.824	0.754	0.834	0.751
170	0.905	0.829	0.751	0.829	0.744
171	0.916	0.827	0.760	0.835	0.752
172	0.919	0.804	0.766	0.829	0.744
173	0.910	0.845	0.772	0.843	0.765
174	0.905	0.827	0.766	0.833	0.749
175	0.916	0.842	0.772	0.844	0.766
176	0.925	0.817	0.757	0.833	0.749
177	0.925	0.804	0.743	0.824	0.735
178	0.902	0.806	0.740	0.816	0.724
179	0.908	0.804	0.760	0.824	0.736

180	0.908	0.832	0.743	0.828	0.742
181	0.905	0.817	0.763	0.828	0.742
182	0.925	0.832	0.766	0.842	0.762
183	0.913	0.814	0.775	0.834	0.751
184	0.919	0.809	0.796	0.841	0.761
185	0.916	0.811	0.778	0.835	0.752
186	0.902	0.832	0.778	0.838	0.756
187	0.905	0.804	0.772	0.827	0.740
188	0.893	0.819	0.749	0.821	0.731
189	0.882	0.822	0.796	0.833	0.750
190	0.913	0.814	0.716	0.815	0.722
191	0.913	0.801	0.731	0.815	0.723
192	0.905	0.798	0.778	0.827	0.740
193	0.919	0.829	0.760	0.837	0.755
194	0.908	0.832	0.746	0.829	0.744
195	0.899	0.791	0.757	0.815	0.723
196	0.913	0.780	0.746	0.813	0.719
197	0.922	0.837	0.790	0.850	0.775
198	0.902	0.819	0.778	0.833	0.750
199	0.902	0.783	0.746	0.810	0.714
200	0.916	0.817	0.769	0.834	0.751
201	0.936	0.752	0.743	0.809	0.713
202	0.916	0.829	0.760	0.836	0.754
203	0.899	0.817	0.749	0.822	0.732
204	0.896	0.801	0.737	0.812	0.717
205	0.902	0.819	0.760	0.828	0.741
206	0.910	0.819	0.743	0.825	0.737
207	0.910	0.824	0.787	0.841	0.761
208	0.916	0.783	0.728	0.809	0.713
209	0.919	0.829	0.754	0.835	0.752
210	0.913	0.814	0.760	0.829	0.744
211	0.890	0.796	0.754	0.813	0.720
212	0.890	0.804	0.760	0.818	0.727
213	0.899	0.806	0.766	0.824	0.736
214	0.899	0.786	0.763	0.815	0.723
215	0.910	0.804	0.743	0.819	0.728
216	0.928	0.798	0.754	0.827	0.740
217	0.905	0.822	0.757	0.828	0.742
218	0.913	0.829	0.772	0.839	0.758
219	0.908	0.793	0.763	0.821	0.731
220	0.913	0.829	0.740	0.828	0.742
221	0.928	0.801	0.775	0.834	0.751

222	0.908	0.804	0.722	0.812	0.717
223	0.910	0.806	0.787	0.834	0.751
224	0.908	0.835	0.749	0.831	0.746
225	0.916	0.817	0.757	0.830	0.745
226	0.902	0.775	0.772	0.815	0.724
227	0.922	0.835	0.743	0.834	0.751
228	0.922	0.783	0.775	0.826	0.739
229	0.916	0.811	0.778	0.835	0.753
230	0.919	0.788	0.731	0.813	0.718
231	0.893	0.801	0.754	0.816	0.724
232	0.908	0.842	0.763	0.839	0.758
233	0.922	0.822	0.775	0.840	0.759
234	0.902	0.801	0.754	0.819	0.728
235	0.910	0.798	0.740	0.816	0.724
236	0.908	0.837	0.760	0.836	0.753
237	0.908	0.796	0.766	0.823	0.735
238	0.913	0.817	0.781	0.837	0.755
239	0.913	0.804	0.763	0.827	0.740
240	0.919	0.809	0.763	0.830	0.745
241	0.916	0.824	0.737	0.827	0.739
242	0.896	0.796	0.769	0.820	0.730
243	0.905	0.811	0.760	0.826	0.738
244	0.916	0.811	0.722	0.817	0.725
245	0.916	0.788	0.751	0.818	0.727
246	0.919	0.842	0.754	0.840	0.759
247	0.910	0.804	0.769	0.828	0.741
248	0.905	0.806	0.757	0.823	0.734
249	0.931	0.786	0.751	0.822	0.733
250	0.928	0.801	0.743	0.824	0.735
251	0.919	0.817	0.766	0.834	0.751
252	0.919	0.796	0.737	0.817	0.726
253	0.905	0.806	0.787	0.832	0.748
254	0.913	0.798	0.757	0.823	0.734
255	0.893	0.798	0.760	0.817	0.726
256	0.905	0.793	0.775	0.824	0.736
257	0.884	0.786	0.746	0.805	0.708
258	0.922	0.783	0.775	0.826	0.739
259	0.910	0.811	0.740	0.821	0.731
260	0.916	0.780	0.743	0.813	0.719
261	0.905	0.809	0.784	0.832	0.748
262	0.908	0.798	0.760	0.822	0.733
263	0.919	0.811	0.737	0.823	0.734

264	0.908	0.801	0.781	0.829	0.744
265	0.913	0.798	0.743	0.818	0.727
266	0.887	0.804	0.778	0.823	0.734
267	0.913	0.850	0.778	0.848	0.772
268	0.887	0.788	0.731	0.802	0.703
269	0.919	0.801	0.772	0.830	0.745
270	0.887	0.788	0.769	0.814	0.722
271	0.913	0.817	0.760	0.830	0.745
272	0.908	0.773	0.769	0.815	0.724
273	0.896	0.814	0.713	0.809	0.713
274	0.934	0.817	0.760	0.837	0.755
275	0.916	0.798	0.740	0.818	0.727
276	0.905	0.780	0.716	0.800	0.700
277	0.910	0.780	0.769	0.819	0.729
278	0.913	0.814	0.784	0.837	0.755
279	0.916	0.801	0.749	0.822	0.733
280	0.919	0.817	0.754	0.830	0.745
281	0.890	0.809	0.731	0.811	0.716
282	0.922	0.814	0.760	0.832	0.748
283	0.910	0.822	0.743	0.826	0.738
284	0.913	0.806	0.784	0.834	0.751
285	0.896	0.824	0.757	0.827	0.740
286	0.928	0.804	0.760	0.830	0.745
287	0.910	0.791	0.763	0.821	0.731
288	0.916	0.804	0.760	0.827	0.740
289	0.913	0.827	0.769	0.837	0.755
290	0.928	0.783	0.749	0.819	0.729
291	0.896	0.770	0.728	0.798	0.696
292	0.925	0.773	0.746	0.813	0.720
293	0.908	0.824	0.769	0.834	0.751
294	0.902	0.775	0.754	0.810	0.715
295	0.908	0.796	0.737	0.813	0.720
296	0.879	0.827	0.754	0.821	0.731
297	0.916	0.824	0.731	0.825	0.736
298	0.905	0.778	0.757	0.813	0.719
299	0.902	0.814	0.781	0.832	0.749
300	0.925	0.809	0.754	0.829	0.744
301	0.899	0.786	0.746	0.810	0.714
302	0.910	0.798	0.763	0.824	0.735
303	0.925	0.811	0.757	0.831	0.747
304	0.887	0.801	0.769	0.819	0.729
305	0.913	0.822	0.769	0.835	0.752

306	0.913	0.806	0.778	0.832	0.748
307	0.902	0.801	0.763	0.822	0.733
308	0.910	0.814	0.751	0.826	0.738
309	0.902	0.796	0.754	0.817	0.726
310	0.905	0.786	0.746	0.812	0.717
311	0.922	0.811	0.781	0.838	0.757
312	0.910	0.767	0.766	0.813	0.721
313	0.913	0.842	0.766	0.842	0.762
314	0.910	0.804	0.769	0.828	0.741
315	0.910	0.817	0.769	0.832	0.748
316	0.908	0.775	0.740	0.807	0.710
317	0.910	0.811	0.790	0.837	0.755
318	0.896	0.806	0.769	0.824	0.736
319	0.913	0.817	0.775	0.835	0.752
320	0.913	0.796	0.743	0.817	0.725
321	0.919	0.809	0.751	0.827	0.740
322	0.910	0.796	0.751	0.819	0.729
323	0.905	0.804	0.740	0.816	0.724
324	0.902	0.780	0.749	0.810	0.714
325	0.922	0.778	0.754	0.817	0.726
326	0.922	0.809	0.772	0.834	0.751
327	0.890	0.786	0.722	0.799	0.699
328	0.916	0.749	0.772	0.811	0.717
329	0.899	0.801	0.749	0.816	0.724
330	0.899	0.762	0.743	0.800	0.701
331	0.913	0.801	0.775	0.829	0.744
332	0.905	0.793	0.757	0.818	0.727
333	0.913	0.788	0.746	0.815	0.723
334	0.908	0.827	0.740	0.826	0.738
335	0.916	0.814	0.766	0.832	0.748
336	0.916	0.757	0.757	0.809	0.713
337	0.916	0.775	0.731	0.807	0.710
338	0.925	0.804	0.757	0.828	0.742
339	0.908	0.822	0.734	0.822	0.732
340	0.896	0.809	0.754	0.820	0.730
341	0.913	0.809	0.757	0.827	0.740
342	0.919	0.806	0.760	0.828	0.743
343	0.899	0.819	0.746	0.822	0.732
344	0.908	0.793	0.746	0.815	0.723
345	0.910	0.801	0.731	0.814	0.721
346	0.910	0.811	0.707	0.811	0.715
347	0.913	0.811	0.754	0.827	0.740

348	0.925	0.780	0.769	0.824	0.736
349	0.890	0.793	0.746	0.810	0.714
350	0.899	0.796	0.737	0.811	0.716
351	0.910	0.783	0.737	0.810	0.714
352	0.910	0.817	0.749	0.826	0.738
353	0.916	0.775	0.719	0.803	0.705
354	0.908	0.793	0.710	0.804	0.706
355	0.916	0.817	0.746	0.827	0.739
356	0.913	0.804	0.766	0.828	0.741
357	0.905	0.811	0.743	0.820	0.730
358	0.908	0.749	0.734	0.796	0.694
359	0.893	0.755	0.763	0.802	0.704
360	0.913	0.819	0.751	0.828	0.742
361	0.925	0.798	0.766	0.829	0.744
362	0.899	0.811	0.781	0.830	0.746
363	0.908	0.798	0.775	0.827	0.740
364	0.925	0.798	0.772	0.831	0.747
365	0.910	0.786	0.740	0.812	0.717
366	0.908	0.798	0.728	0.812	0.717
367	0.905	0.798	0.754	0.819	0.728
368	0.919	0.788	0.737	0.814	0.721
369	0.910	0.817	0.740	0.823	0.734
370	0.916	0.788	0.757	0.820	0.730
371	0.899	0.783	0.749	0.810	0.714
372	0.919	0.801	0.740	0.820	0.730
373	0.905	0.775	0.746	0.808	0.712
374	0.925	0.796	0.725	0.815	0.723
375	0.908	0.796	0.746	0.816	0.724
376	0.928	0.827	0.725	0.828	0.741
377	0.936	0.788	0.749	0.824	0.736
378	0.910	0.788	0.751	0.816	0.724
379	0.908	0.801	0.746	0.818	0.727
380	0.913	0.788	0.749	0.816	0.724
381	0.913	0.801	0.778	0.830	0.745
382	0.908	0.783	0.757	0.815	0.723
383	0.908	0.814	0.716	0.813	0.720
384	0.910	0.788	0.757	0.818	0.727
385	0.919	0.801	0.757	0.826	0.738
386	0.893	0.767	0.704	0.788	0.682
387	0.916	0.809	0.766	0.830	0.745
388	0.916	0.814	0.757	0.829	0.744
389	0.908	0.793	0.740	0.813	0.720

390	0.902	0.798	0.740	0.813	0.720
391	0.896	0.770	0.754	0.806	0.709
392	0.910	0.798	0.743	0.817	0.725
393	0.931	0.791	0.731	0.817	0.725
394	0.913	0.788	0.763	0.821	0.731
395	0.913	0.822	0.743	0.827	0.739
396	0.919	0.778	0.737	0.811	0.716
397	0.896	0.765	0.746	0.801	0.702
398	0.931	0.819	0.749	0.833	0.749
399	0.908	0.796	0.716	0.807	0.710
400	0.913	0.780	0.751	0.814	0.722
401	0.934	0.788	0.757	0.826	0.738
402	0.934	0.783	0.740	0.818	0.727
403	0.910	0.791	0.749	0.816	0.724
404	0.919	0.804	0.740	0.821	0.731
405	0.908	0.783	0.757	0.815	0.723
406	0.908	0.780	0.713	0.800	0.700
407	0.910	0.736	0.731	0.791	0.687
408	0.905	0.786	0.778	0.822	0.733
409	0.910	0.778	0.734	0.807	0.710
410	0.905	0.817	0.754	0.826	0.738
411	0.916	0.819	0.766	0.834	0.751
412	0.922	0.778	0.751	0.816	0.724
413	0.922	0.775	0.743	0.813	0.719
414	0.913	0.806	0.757	0.826	0.738
415	0.913	0.832	0.743	0.830	0.745
416	0.910	0.809	0.766	0.828	0.743
417	0.908	0.804	0.751	0.821	0.731
418	0.919	0.793	0.734	0.815	0.723
419	0.893	0.778	0.731	0.800	0.701
420	0.916	0.809	0.757	0.828	0.741
421	0.913	0.793	0.725	0.811	0.716
422	0.896	0.801	0.710	0.803	0.704
423	0.910	0.806	0.737	0.818	0.727
424	0.934	0.806	0.763	0.834	0.751
425	0.899	0.827	0.743	0.824	0.735
426	0.922	0.804	0.731	0.819	0.728
427	0.902	0.804	0.766	0.824	0.736
428	0.908	0.757	0.772	0.811	0.717
429	0.919	0.791	0.749	0.819	0.729
430	0.925	0.804	0.734	0.821	0.731
431	0.899	0.775	0.740	0.804	0.706

432	0.902	0.811	0.728	0.814	0.721
433	0.908	0.817	0.731	0.819	0.728
434	0.899	0.783	0.749	0.810	0.715
435	0.899	0.786	0.757	0.813	0.720
436	0.902	0.804	0.743	0.816	0.724
437	0.916	0.765	0.746	0.808	0.712
438	0.913	0.773	0.740	0.808	0.712
439	0.922	0.819	0.737	0.827	0.739
440	0.905	0.804	0.769	0.826	0.739
441	0.910	0.798	0.737	0.815	0.723
442	0.928	0.801	0.760	0.829	0.744
443	0.931	0.798	0.749	0.826	0.738
444	0.887	0.767	0.760	0.804	0.706
445	0.910	0.757	0.737	0.800	0.701
446	0.916	0.778	0.749	0.813	0.720
447	0.913	0.806	0.734	0.818	0.727
448	0.916	0.804	0.722	0.814	0.721
449	0.916	0.801	0.751	0.823	0.734
450	0.893	0.796	0.751	0.813	0.720
451	0.913	0.801	0.719	0.812	0.717
452	0.913	0.788	0.769	0.823	0.735
453	0.896	0.767	0.740	0.800	0.701
454	0.913	0.811	0.751	0.826	0.738
455	0.919	0.809	0.766	0.831	0.747
456	0.934	0.788	0.757	0.826	0.738
457	0.916	0.814	0.763	0.831	0.747
458	0.919	0.788	0.763	0.823	0.734
459	0.908	0.780	0.722	0.803	0.704
460	0.913	0.809	0.749	0.824	0.735
461	0.919	0.786	0.766	0.823	0.735
462	0.916	0.801	0.737	0.818	0.727
463	0.899	0.775	0.763	0.812	0.718
464	0.905	0.773	0.757	0.811	0.716
465	0.925	0.770	0.749	0.813	0.720
466	0.936	0.786	0.719	0.813	0.720
467	0.919	0.775	0.757	0.816	0.724
468	0.916	0.788	0.734	0.813	0.718
469	0.913	0.780	0.746	0.813	0.719
470	0.913	0.765	0.754	0.810	0.715
471	0.908	0.809	0.737	0.818	0.727
472	0.902	0.783	0.754	0.813	0.719
473	0.928	0.780	0.740	0.815	0.723

474	0.905	0.793	0.778	0.825	0.737
475	0.916	0.780	0.775	0.823	0.735
476	0.925	0.801	0.751	0.826	0.738
477	0.905	0.793	0.766	0.821	0.732
478	0.910	0.773	0.746	0.809	0.713
479	0.902	0.786	0.769	0.818	0.728
480	0.922	0.804	0.749	0.825	0.737
481	0.913	0.778	0.772	0.820	0.731
482	0.896	0.819	0.757	0.825	0.737
483	0.908	0.806	0.772	0.828	0.743
484	0.905	0.801	0.743	0.816	0.724
485	0.916	0.760	0.772	0.814	0.723
486	0.908	0.778	0.743	0.809	0.713
487	0.916	0.793	0.746	0.818	0.727
488	0.922	0.819	0.772	0.838	0.757
489	0.925	0.832	0.725	0.828	0.742
490	0.913	0.767	0.725	0.801	0.702
491	0.910	0.801	0.760	0.824	0.735
492	0.910	0.809	0.743	0.821	0.731
493	0.925	0.783	0.760	0.822	0.733
494	0.905	0.765	0.766	0.811	0.717
495	0.913	0.788	0.740	0.813	0.720
496	0.905	0.780	0.778	0.820	0.731
497	0.896	0.801	0.775	0.824	0.736
498	0.916	0.796	0.749	0.820	0.730
499	0.916	0.796	0.740	0.817	0.725
500	0.919	0.801	0.766	0.828	0.743
501	0.922	0.788	0.760	0.823	0.734
502	0.925	0.804	0.746	0.825	0.737
503	0.922	0.798	0.680	0.801	0.701
504	0.916	0.791	0.772	0.826	0.739
505	0.910	0.778	0.763	0.816	0.725
506	0.905	0.832	0.751	0.830	0.745
507	0.910	0.798	0.746	0.818	0.727
508	0.899	0.804	0.760	0.821	0.731
509	0.928	0.804	0.775	0.835	0.753
510	0.919	0.752	0.722	0.797	0.695
511	0.916	0.806	0.751	0.825	0.737
512	0.899	0.791	0.757	0.815	0.723
513	0.913	0.801	0.763	0.826	0.738
514	0.902	0.809	0.743	0.818	0.727
515	0.908	0.832	0.751	0.831	0.747

516	0.913	0.827	0.725	0.823	0.734
517	0.902	0.793	0.778	0.824	0.736
518	0.908	0.762	0.763	0.810	0.715
519	0.899	0.832	0.746	0.827	0.739
520	0.913	0.822	0.757	0.831	0.747
521	0.916	0.770	0.766	0.816	0.725
522	0.910	0.778	0.743	0.810	0.714
523	0.902	0.835	0.749	0.829	0.744
524	0.919	0.801	0.749	0.823	0.734
525	0.925	0.806	0.728	0.820	0.730
526	0.925	0.786	0.760	0.823	0.734
527	0.916	0.786	0.757	0.819	0.729
528	0.908	0.773	0.746	0.808	0.712
529	0.902	0.765	0.769	0.811	0.716
530	0.919	0.801	0.766	0.828	0.743
531	0.919	0.762	0.746	0.808	0.712
532	0.896	0.801	0.743	0.813	0.720
533	0.919	0.806	0.728	0.818	0.727
534	0.910	0.801	0.754	0.822	0.733
535	0.908	0.765	0.725	0.799	0.697
536	0.896	0.765	0.722	0.794	0.691
537	0.902	0.780	0.772	0.817	0.726
538	0.916	0.796	0.737	0.816	0.724
539	0.919	0.765	0.763	0.814	0.722
540	0.916	0.752	0.740	0.801	0.702
541	0.925	0.798	0.743	0.822	0.733
542	0.931	0.793	0.763	0.828	0.743
543	0.910	0.767	0.766	0.813	0.721
544	0.908	0.793	0.751	0.817	0.726
545	0.896	0.801	0.749	0.815	0.723
546	0.934	0.804	0.757	0.831	0.747
547	0.910	0.780	0.737	0.809	0.713
548	0.893	0.783	0.760	0.812	0.718
549	0.913	0.793	0.754	0.820	0.730
550	0.916	0.778	0.731	0.808	0.711
551	0.919	0.775	0.743	0.812	0.717
552	0.939	0.742	0.716	0.798	0.697
553	0.913	0.778	0.746	0.812	0.717
554	0.922	0.806	0.680	0.804	0.706
555	0.902	0.811	0.713	0.810	0.714
556	0.908	0.718	0.710	0.777	0.666
557	0.905	0.788	0.710	0.801	0.701

558	0.905	0.791	0.757	0.817	0.726
559	0.919	0.765	0.725	0.802	0.704
560	0.928	0.780	0.719	0.809	0.713
561	0.890	0.801	0.707	0.800	0.700
562	0.908	0.762	0.763	0.810	0.715
563	0.925	0.783	0.749	0.818	0.727
564	0.905	0.798	0.751	0.818	0.727
565	0.908	0.806	0.737	0.817	0.725
566	0.922	0.796	0.746	0.821	0.731
567	0.902	0.783	0.737	0.807	0.710
568	0.922	0.778	0.737	0.812	0.717
569	0.925	0.796	0.778	0.832	0.749
570	0.908	0.783	0.719	0.803	0.704
571	0.913	0.773	0.754	0.813	0.719
572	0.919	0.801	0.740	0.820	0.730
573	0.910	0.817	0.763	0.830	0.745
574	0.957	0.783	0.737	0.825	0.737
575	0.922	0.806	0.763	0.830	0.745
576	0.919	0.796	0.725	0.813	0.720
577	0.908	0.752	0.749	0.801	0.703
578	0.908	0.798	0.731	0.813	0.718
579	0.910	0.783	0.749	0.813	0.720
580	0.910	0.801	0.760	0.824	0.736
581	0.916	0.788	0.731	0.812	0.717
582	0.902	0.788	0.763	0.817	0.726
583	0.896	0.809	0.746	0.817	0.726
584	0.916	0.819	0.743	0.827	0.739
585	0.919	0.811	0.751	0.828	0.741
586	0.919	0.773	0.743	0.811	0.716
587	0.922	0.822	0.790	0.844	0.766
588	0.919	0.796	0.740	0.818	0.727
589	0.928	0.817	0.749	0.831	0.747
590	0.916	0.804	0.728	0.816	0.724
591	0.905	0.806	0.754	0.822	0.733
592	0.910	0.817	0.775	0.834	0.751
593	0.913	0.767	0.778	0.818	0.728
594	0.908	0.780	0.772	0.819	0.729
595	0.925	0.801	0.728	0.818	0.727
596	0.919	0.775	0.737	0.810	0.715
597	0.899	0.786	0.734	0.806	0.709
598	0.910	0.775	0.713	0.799	0.699
599	0.908	0.801	0.740	0.816	0.724

600	0.919	0.773	0.760	0.816	0.725
601	0.916	0.786	0.728	0.810	0.714
602	0.922	0.791	0.769	0.827	0.740
603	0.902	0.796	0.757	0.818	0.727
604	0.919	0.780	0.728	0.809	0.713
605	0.905	0.783	0.719	0.802	0.703
606	0.922	0.811	0.737	0.824	0.735
607	0.905	0.796	0.740	0.813	0.720
608	0.916	0.791	0.734	0.813	0.720
609	0.925	0.765	0.737	0.808	0.712
610	0.922	0.786	0.769	0.825	0.737
611	0.931	0.809	0.796	0.844	0.767
612	0.893	0.780	0.731	0.801	0.702
613	0.893	0.798	0.760	0.817	0.726
614	0.913	0.762	0.749	0.807	0.711
615	0.913	0.744	0.743	0.799	0.698
616	0.910	0.775	0.713	0.799	0.699
617	0.908	0.765	0.769	0.813	0.719
618	0.905	0.791	0.722	0.806	0.708
619	0.919	0.783	0.707	0.803	0.704
620	0.916	0.806	0.757	0.827	0.740
621	0.919	0.783	0.757	0.819	0.729
622	0.913	0.809	0.719	0.814	0.721
623	0.922	0.811	0.731	0.822	0.732
624	0.919	0.819	0.737	0.826	0.738
625	0.928	0.765	0.746	0.812	0.718
626	0.910	0.775	0.781	0.821	0.732
627	0.913	0.752	0.743	0.801	0.702
628	0.928	0.791	0.766	0.828	0.741
629	0.916	0.780	0.757	0.817	0.726
630	0.916	0.801	0.754	0.824	0.735
631	0.916	0.767	0.722	0.801	0.702
632	0.925	0.783	0.734	0.813	0.720
633	0.910	0.775	0.760	0.814	0.722
634	0.910	0.757	0.743	0.802	0.703
635	0.905	0.798	0.725	0.810	0.714
636	0.925	0.801	0.751	0.826	0.738
637	0.922	0.793	0.740	0.818	0.727
638	0.908	0.770	0.725	0.800	0.700
639	0.916	0.762	0.725	0.800	0.701
640	0.910	0.811	0.734	0.819	0.728
641	0.925	0.804	0.722	0.817	0.725

642	0.919	0.801	0.731	0.817	0.725
643	0.934	0.793	0.754	0.827	0.740
644	0.931	0.811	0.731	0.825	0.737
645	0.919	0.757	0.701	0.792	0.687
646	0.916	0.809	0.728	0.818	0.727
647	0.908	0.760	0.719	0.795	0.692
648	0.922	0.809	0.737	0.823	0.734
649	0.893	0.786	0.769	0.815	0.723
650	0.896	0.804	0.719	0.807	0.710
651	0.908	0.786	0.731	0.808	0.711
652	0.916	0.814	0.716	0.816	0.724
653	0.922	0.788	0.737	0.815	0.723
654	0.925	0.773	0.713	0.803	0.704
655	0.922	0.806	0.760	0.829	0.744
656	0.913	0.817	0.722	0.818	0.727
657	0.934	0.780	0.746	0.819	0.729
658	0.922	0.757	0.719	0.799	0.698
659	0.910	0.767	0.731	0.802	0.703
660	0.908	0.760	0.725	0.797	0.695
661	0.899	0.798	0.754	0.817	0.726
662	0.890	0.806	0.716	0.805	0.707
663	0.896	0.780	0.743	0.806	0.709
664	0.934	0.796	0.754	0.828	0.741
665	0.899	0.788	0.775	0.820	0.730
666	0.922	0.778	0.719	0.806	0.709
667	0.931	0.788	0.754	0.824	0.736
668	0.902	0.767	0.731	0.799	0.699
669	0.916	0.773	0.728	0.805	0.707
670	0.919	0.791	0.743	0.817	0.725
671	0.910	0.783	0.751	0.814	0.721
672	0.910	0.780	0.743	0.811	0.716
673	0.916	0.773	0.757	0.814	0.722
674	0.908	0.796	0.707	0.804	0.706
675	0.893	0.762	0.716	0.790	0.685
676	0.913	0.788	0.769	0.823	0.735
677	0.931	0.793	0.719	0.814	0.721
678	0.908	0.804	0.728	0.813	0.720
679	0.922	0.801	0.737	0.820	0.730
680	0.910	0.801	0.766	0.826	0.738
681	0.922	0.778	0.740	0.813	0.719
682	0.928	0.765	0.740	0.810	0.715
683	0.905	0.804	0.757	0.822	0.733

684	0.913	0.811	0.749	0.825	0.737
685	0.905	0.770	0.728	0.800	0.701
686	0.919	0.770	0.751	0.813	0.719
687	0.910	0.791	0.766	0.822	0.733
688	0.905	0.798	0.743	0.815	0.723
689	0.913	0.791	0.704	0.803	0.704
690	0.925	0.801	0.749	0.825	0.737
691	0.928	0.767	0.713	0.802	0.703
692	0.902	0.801	0.749	0.817	0.726
693	0.916	0.786	0.740	0.813	0.720
694	0.913	0.804	0.737	0.818	0.727
695	0.919	0.786	0.692	0.799	0.698
696	0.913	0.780	0.722	0.805	0.707
697	0.905	0.788	0.710	0.801	0.701
698	0.910	0.791	0.751	0.817	0.726
699	0.916	0.796	0.749	0.820	0.730
700	0.902	0.760	0.743	0.800	0.701
701	0.910	0.736	0.731	0.791	0.687
702	0.913	0.775	0.754	0.813	0.721
703	0.922	0.786	0.734	0.813	0.720
704	0.922	0.783	0.737	0.813	0.720
705	0.916	0.755	0.740	0.802	0.704
706	0.913	0.749	0.763	0.807	0.711
707	0.913	0.770	0.749	0.810	0.715
708	0.893	0.765	0.710	0.789	0.683
709	0.922	0.765	0.731	0.805	0.708
710	0.910	0.817	0.719	0.816	0.724
711	0.913	0.770	0.746	0.809	0.713
712	0.916	0.749	0.763	0.808	0.713
713	0.910	0.755	0.766	0.809	0.714
714	0.908	0.809	0.763	0.827	0.740
715	0.910	0.835	0.769	0.839	0.758
716	0.910	0.775	0.737	0.807	0.710
717	0.916	0.780	0.763	0.819	0.729
718	0.931	0.801	0.740	0.824	0.735
719	0.934	0.793	0.746	0.824	0.735
720	0.908	0.806	0.737	0.817	0.725
721	0.922	0.778	0.734	0.811	0.716
722	0.939	0.793	0.749	0.827	0.740
723	0.910	0.734	0.754	0.798	0.697
724	0.913	0.809	0.719	0.814	0.721
725	0.916	0.791	0.722	0.810	0.714

726	0.913	0.786	0.731	0.810	0.714
727	0.899	0.811	0.737	0.816	0.724
728	0.913	0.770	0.731	0.804	0.706
729	0.913	0.791	0.766	0.823	0.734
730	0.928	0.796	0.754	0.826	0.738
731	0.910	0.775	0.760	0.814	0.722
732	0.913	0.801	0.766	0.827	0.740
733	0.913	0.814	0.749	0.826	0.738
734	0.925	0.775	0.731	0.810	0.714
735	0.919	0.786	0.707	0.804	0.706
736	0.925	0.791	0.743	0.819	0.728
737	0.916	0.786	0.754	0.818	0.727
738	0.916	0.780	0.760	0.818	0.727
739	0.890	0.780	0.763	0.811	0.716
740	0.922	0.793	0.769	0.828	0.741
741	0.905	0.793	0.707	0.802	0.703
742	0.916	0.801	0.734	0.817	0.725
743	0.919	0.801	0.728	0.816	0.724
744	0.925	0.762	0.749	0.811	0.716
745	0.905	0.760	0.743	0.801	0.702
746	0.916	0.780	0.743	0.813	0.719
747	0.925	0.788	0.716	0.810	0.714
748	0.928	0.767	0.754	0.815	0.723
749	0.896	0.744	0.751	0.796	0.694
750	0.919	0.809	0.722	0.817	0.725
751	0.910	0.721	0.760	0.795	0.694
752	0.922	0.775	0.763	0.819	0.729
753	0.902	0.809	0.754	0.822	0.733
754	0.910	0.788	0.731	0.810	0.714
755	0.908	0.788	0.763	0.819	0.729
756	0.913	0.786	0.713	0.804	0.706
757	0.919	0.773	0.716	0.802	0.703
758	0.913	0.788	0.775	0.825	0.738
759	0.910	0.786	0.728	0.808	0.711
760	0.902	0.793	0.743	0.813	0.719
761	0.910	0.788	0.719	0.806	0.709
762	0.922	0.796	0.763	0.827	0.740
763	0.896	0.775	0.722	0.798	0.696
764	0.908	0.767	0.722	0.799	0.698
765	0.913	0.775	0.713	0.800	0.700
766	0.916	0.811	0.719	0.816	0.724
767	0.919	0.806	0.716	0.814	0.721

768	0.916	0.775	0.746	0.812	0.717
769	0.919	0.788	0.728	0.812	0.717
770	0.916	0.786	0.746	0.815	0.723
771	0.910	0.786	0.746	0.813	0.720
772	0.922	0.793	0.707	0.808	0.711
773	0.916	0.798	0.740	0.818	0.727
774	0.925	0.809	0.731	0.822	0.732
775	0.928	0.798	0.746	0.824	0.735
776	0.905	0.788	0.740	0.811	0.716
777	0.913	0.791	0.766	0.823	0.735
778	0.910	0.783	0.754	0.815	0.723
779	0.916	0.786	0.713	0.805	0.707
780	0.899	0.778	0.737	0.804	0.706
781	0.928	0.804	0.751	0.828	0.741
782	0.922	0.796	0.740	0.819	0.728
783	0.934	0.775	0.725	0.811	0.716
784	0.910	0.786	0.737	0.811	0.716
785	0.913	0.757	0.766	0.811	0.717
786	0.908	0.793	0.746	0.815	0.723
787	0.916	0.770	0.751	0.812	0.718
788	0.936	0.773	0.701	0.803	0.704

(2) Performance of KNN on top features yielded by three feature selection methods

**mRMR**					
**Numbers of features**	**G1**	**G2/M**	**S**	**ACC**	**MCC**
1	0.902	0.643	0.554	0.699	0.549
2	0.916	0.755	0.674	0.782	0.672
3	0.870	0.752	0.716	0.779	0.669
4	0.899	0.767	0.749	0.804	0.707
5	0.925	0.798	0.731	0.818	0.727
6	0.936	0.765	0.692	0.798	0.696
7	0.925	0.757	0.722	0.800	0.701
8	0.939	0.757	0.674	0.790	0.685
9	0.948	0.778	0.686	0.804	0.706
10	0.939	0.747	0.692	0.792	0.688
11	0.945	0.749	0.677	0.790	0.685
12	0.957	0.778	0.707	0.813	0.720
13	0.957	0.770	0.710	0.812	0.717
14	0.960	0.755	0.737	0.815	0.723
15	0.954	0.780	0.728	0.820	0.730
16	0.957	0.773	0.751	0.826	0.738
17	0.945	0.770	0.746	0.819	0.729
18	0.951	0.778	0.734	0.820	0.730

19	0.957	0.770	0.751	0.825	0.737
20	0.945	0.744	0.751	0.812	0.718
21	0.939	0.752	0.743	0.810	0.715
22	0.951	0.757	0.749	0.817	0.726
23	0.962	0.780	0.737	0.826	0.738
24	0.960	0.791	0.754	0.834	0.751
25	0.962	0.786	0.734	0.827	0.739
26	0.960	0.783	0.757	0.832	0.748
27	0.957	0.793	0.754	0.834	0.751
28	0.954	0.804	0.737	0.831	0.746
29	0.954	0.814	0.719	0.829	0.744
30	0.954	0.819	0.746	0.840	0.759
31	0.954	0.819	0.757	0.843	0.765
32	0.948	0.829	0.749	0.843	0.763
33	0.962	0.827	0.731	0.841	0.761
34	0.957	0.827	0.754	0.846	0.769
35	0.954	0.840	0.781	0.858	0.787
36	0.971	0.832	0.784	0.862	0.793
37	0.971	0.840	0.796	0.869	0.803
38	0.974	0.827	0.769	0.857	0.784
39	0.962	0.842	0.790	0.865	0.797
40	0.960	0.840	0.784	0.861	0.792
41	0.960	0.837	0.784	0.860	0.790
42	0.968	0.848	0.790	0.869	0.803
43	0.957	0.832	0.772	0.854	0.780
44	0.951	0.835	0.772	0.853	0.779
45	0.957	0.829	0.784	0.857	0.785
46	0.951	0.853	0.787	0.864	0.796
47	0.962	0.835	0.784	0.860	0.790
48	0.962	0.829	0.778	0.857	0.784
49	0.962	0.835	0.760	0.853	0.779
50	0.960	0.832	0.763	0.852	0.777
51	0.957	0.832	0.793	0.860	0.790
52	0.957	0.835	0.784	0.858	0.787
53	0.962	0.804	0.796	0.853	0.779
54	0.957	0.793	0.802	0.849	0.774
55	0.954	0.788	0.826	0.854	0.782
56	0.954	0.798	0.805	0.851	0.777
57	0.960	0.801	0.811	0.856	0.784
58	0.962	0.791	0.799	0.849	0.774
59	0.962	0.791	0.817	0.855	0.783
60	0.962	0.798	0.796	0.851	0.777

61	0.960	0.798	0.790	0.848	0.772
62	0.957	0.788	0.811	0.850	0.776
63	0.951	0.783	0.778	0.836	0.754
64	0.948	0.778	0.802	0.841	0.762
65	0.948	0.775	0.793	0.837	0.756
66	0.968	0.791	0.796	0.850	0.775
67	0.965	0.811	0.781	0.852	0.778
68	0.965	0.811	0.805	0.859	0.789
69	0.971	0.765	0.778	0.836	0.755
70	0.974	0.767	0.763	0.833	0.750
71	0.968	0.757	0.778	0.832	0.749
72	0.977	0.757	0.760	0.829	0.745
73	0.974	0.744	0.763	0.825	0.738
74	0.974	0.749	0.781	0.832	0.750
75	0.974	0.757	0.778	0.834	0.752
76	0.971	0.762	0.751	0.827	0.740
77	0.977	0.767	0.772	0.837	0.756
78	0.971	0.760	0.793	0.839	0.759
79	0.977	0.762	0.769	0.834	0.752
80	0.977	0.752	0.769	0.830	0.746
81	0.974	0.762	0.784	0.838	0.758
82	0.974	0.757	0.766	0.830	0.746
83	0.965	0.757	0.754	0.824	0.736
84	0.983	0.749	0.772	0.832	0.749
85	0.980	0.752	0.757	0.828	0.742
86	0.977	0.749	0.760	0.827	0.740
87	0.983	0.755	0.766	0.832	0.749
88	0.971	0.757	0.775	0.832	0.749
89	0.977	0.752	0.784	0.835	0.754
90	0.980	0.749	0.775	0.832	0.749
91	0.977	0.765	0.757	0.831	0.747
92	0.977	0.739	0.781	0.829	0.746
93	0.974	0.742	0.766	0.825	0.738
94	0.977	0.752	0.760	0.828	0.742
95	0.974	0.752	0.790	0.836	0.755
96	0.980	0.749	0.784	0.835	0.754
97	0.983	0.734	0.749	0.819	0.730
98	0.974	0.752	0.754	0.825	0.737
99	0.971	0.747	0.763	0.825	0.738
100	0.977	0.752	0.757	0.827	0.740
101	0.980	0.744	0.772	0.829	0.745
102	0.968	0.752	0.754	0.823	0.735

103	0.980	0.752	0.793	0.839	0.759
104	0.968	0.755	0.775	0.830	0.746
105	0.977	0.755	0.778	0.834	0.752
106	0.977	0.744	0.766	0.827	0.741
107	0.977	0.760	0.743	0.825	0.737
108	0.983	0.760	0.766	0.834	0.752
109	0.977	0.762	0.772	0.835	0.753
110	0.968	0.757	0.763	0.828	0.742
111	0.974	0.749	0.757	0.825	0.738
112	0.977	0.762	0.769	0.834	0.752
113	0.986	0.752	0.787	0.839	0.759
114	0.974	0.744	0.772	0.828	0.742
115	0.965	0.747	0.784	0.829	0.745
116	0.974	0.762	0.802	0.843	0.767
117	0.971	0.760	0.784	0.836	0.755
118	0.977	0.762	0.775	0.836	0.754
119	0.971	0.765	0.775	0.835	0.753
120	0.971	0.757	0.769	0.830	0.746
121	0.965	0.757	0.757	0.825	0.738
122	0.974	0.744	0.760	0.824	0.737
123	0.980	0.752	0.754	0.827	0.740
124	0.977	0.739	0.760	0.823	0.735
125	0.986	0.739	0.799	0.838	0.759
126	0.977	0.752	0.781	0.834	0.752
127	0.971	0.752	0.790	0.835	0.754
128	0.962	0.718	0.787	0.819	0.731
129	0.960	0.716	0.763	0.810	0.716
130	0.965	0.718	0.787	0.820	0.732
131	0.960	0.718	0.751	0.807	0.712
132	0.951	0.718	0.781	0.813	0.723
133	0.965	0.721	0.766	0.814	0.723
134	0.957	0.724	0.757	0.810	0.716
135	0.957	0.724	0.766	0.813	0.720
136	0.965	0.744	0.763	0.822	0.734
137	0.960	0.747	0.778	0.826	0.740
138	0.960	0.734	0.802	0.828	0.745
139	0.960	0.742	0.799	0.830	0.747
140	0.960	0.749	0.778	0.827	0.741
141	0.965	0.744	0.775	0.826	0.740
142	0.968	0.742	0.787	0.829	0.746
143	0.962	0.742	0.781	0.826	0.740
144	0.962	0.739	0.772	0.822	0.734

145	0.960	0.749	0.802	0.834	0.753
146	0.962	0.747	0.802	0.834	0.753
147	0.957	0.749	0.784	0.828	0.743
148	0.960	0.760	0.766	0.827	0.740
149	0.968	0.749	0.775	0.828	0.744
150	0.960	0.749	0.790	0.830	0.747
151	0.960	0.749	0.793	0.831	0.748
152	0.965	0.742	0.787	0.828	0.744
153	0.968	0.744	0.781	0.828	0.744
154	0.960	0.742	0.796	0.829	0.746
155	0.957	0.739	0.781	0.823	0.736
156	0.962	0.734	0.784	0.824	0.737
157	0.968	0.744	0.760	0.822	0.734
158	0.960	0.729	0.793	0.824	0.738
159	0.957	0.734	0.772	0.818	0.729
160	0.965	0.742	0.760	0.820	0.731
161	0.951	0.744	0.790	0.826	0.740
162	0.948	0.736	0.790	0.822	0.735
163	0.945	0.726	0.793	0.818	0.730
164	0.954	0.744	0.784	0.825	0.738
165	0.960	0.739	0.781	0.824	0.737
166	0.962	0.747	0.775	0.826	0.739
167	0.962	0.760	0.760	0.826	0.739
168	0.960	0.734	0.799	0.828	0.743
169	0.951	0.755	0.784	0.828	0.742
170	0.957	0.760	0.778	0.829	0.745
171	0.954	0.744	0.763	0.818	0.728
172	0.960	0.749	0.793	0.831	0.748
173	0.971	0.765	0.772	0.834	0.752
174	0.948	0.739	0.775	0.818	0.729
175	0.954	0.742	0.769	0.819	0.730
176	0.948	0.752	0.781	0.825	0.738
177	0.957	0.739	0.793	0.827	0.742
178	0.957	0.752	0.775	0.826	0.739
179	0.960	0.757	0.757	0.823	0.735
180	0.945	0.744	0.778	0.820	0.731
181	0.951	0.744	0.793	0.827	0.742
182	0.957	0.757	0.793	0.833	0.751
183	0.948	0.747	0.793	0.827	0.742
184	0.954	0.762	0.796	0.835	0.754
185	0.968	0.744	0.784	0.829	0.745
186	0.954	0.755	0.787	0.829	0.745

187	0.945	0.744	0.784	0.822	0.734
188	0.948	0.731	0.784	0.818	0.729
189	0.954	0.747	0.790	0.828	0.743
190	0.962	0.749	0.775	0.827	0.741
191	0.951	0.752	0.781	0.826	0.740
192	0.951	0.726	0.787	0.818	0.730
193	0.965	0.752	0.799	0.836	0.755
194	0.965	0.749	0.769	0.826	0.739
195	0.951	0.749	0.772	0.822	0.734
196	0.960	0.744	0.784	0.827	0.741
197	0.954	0.736	0.799	0.827	0.742
198	0.960	0.765	0.769	0.829	0.744
199	0.945	0.755	0.781	0.825	0.738
200	0.962	0.742	0.790	0.828	0.744
201	0.954	0.749	0.775	0.824	0.737
202	0.960	0.747	0.766	0.822	0.734
203	0.957	0.755	0.781	0.828	0.744
204	0.957	0.749	0.772	0.824	0.737
205	0.962	0.742	0.766	0.821	0.733
206	0.957	0.752	0.763	0.822	0.734
207	0.954	0.755	0.760	0.821	0.732
208	0.954	0.744	0.781	0.824	0.737
209	0.957	0.747	0.760	0.819	0.729
210	0.951	0.747	0.760	0.817	0.727
211	0.960	0.760	0.766	0.827	0.740
212	0.960	0.757	0.772	0.828	0.742
213	0.948	0.744	0.784	0.823	0.736
214	0.951	0.760	0.757	0.821	0.732
215	0.954	0.747	0.772	0.822	0.734
216	0.954	0.742	0.781	0.823	0.736
217	0.945	0.744	0.778	0.820	0.731
218	0.962	0.749	0.769	0.825	0.738
219	0.968	0.749	0.754	0.822	0.733
220	0.968	0.742	0.775	0.826	0.740
221	0.960	0.744	0.772	0.823	0.735
222	0.960	0.744	0.784	0.827	0.741
223	0.957	0.747	0.766	0.821	0.732
224	0.957	0.749	0.751	0.817	0.726
225	0.962	0.742	0.751	0.816	0.725
226	0.962	0.752	0.754	0.821	0.732
227	0.965	0.755	0.760	0.825	0.737
228	0.957	0.742	0.793	0.828	0.743

229	0.968	0.749	0.754	0.822	0.733
230	0.960	0.752	0.778	0.828	0.742
231	0.962	0.747	0.757	0.820	0.731
232	0.968	0.749	0.763	0.825	0.738
233	0.957	0.752	0.766	0.823	0.735
234	0.962	0.749	0.769	0.825	0.738
235	0.962	0.721	0.775	0.816	0.726
236	0.962	0.736	0.766	0.819	0.730
237	0.960	0.773	0.772	0.833	0.750
238	0.965	0.767	0.763	0.830	0.746
239	0.960	0.775	0.772	0.834	0.751
240	0.965	0.773	0.763	0.832	0.748
241	0.968	0.775	0.775	0.838	0.757
242	0.968	0.767	0.778	0.836	0.754
243	0.965	0.788	0.772	0.841	0.761
244	0.960	0.760	0.784	0.832	0.749
245	0.957	0.765	0.769	0.828	0.743
246	0.962	0.767	0.778	0.834	0.752
247	0.965	0.775	0.778	0.838	0.757
248	0.968	0.780	0.769	0.838	0.757
249	0.971	0.767	0.746	0.827	0.740
250	0.962	0.775	0.760	0.831	0.747
251	0.957	0.765	0.784	0.833	0.750
252	0.957	0.773	0.781	0.835	0.753
253	0.968	0.773	0.763	0.833	0.750
254	0.957	0.765	0.772	0.829	0.744
255	0.960	0.783	0.796	0.844	0.767
256	0.962	0.775	0.769	0.834	0.751
257	0.960	0.767	0.766	0.829	0.744
258	0.962	0.775	0.751	0.828	0.743
259	0.962	0.767	0.781	0.835	0.753
260	0.962	0.778	0.760	0.832	0.748
261	0.971	0.762	0.766	0.831	0.747
262	0.957	0.755	0.802	0.835	0.754
263	0.962	0.775	0.775	0.836	0.754
264	0.962	0.778	0.766	0.834	0.751
265	0.971	0.780	0.766	0.838	0.757
266	0.968	0.773	0.760	0.832	0.748
267	0.960	0.773	0.763	0.830	0.746
268	0.965	0.775	0.784	0.840	0.760
269	0.965	0.755	0.781	0.831	0.748
270	0.962	0.786	0.778	0.841	0.761

271	0.965	0.775	0.772	0.836	0.754
272	0.965	0.783	0.760	0.835	0.752
273	0.962	0.775	0.784	0.839	0.759
274	0.954	0.773	0.763	0.828	0.743
275	0.960	0.757	0.790	0.833	0.751
276	0.968	0.770	0.772	0.835	0.753
277	0.957	0.757	0.778	0.828	0.743
278	0.965	0.770	0.757	0.829	0.744
279	0.957	0.767	0.751	0.824	0.736
280	0.957	0.773	0.772	0.832	0.749
281	0.960	0.755	0.772	0.827	0.741
282	0.968	0.770	0.763	0.832	0.748
283	0.962	0.778	0.772	0.836	0.754
284	0.968	0.773	0.760	0.832	0.748
285	0.968	0.775	0.781	0.840	0.760
286	0.960	0.778	0.754	0.829	0.744
287	0.957	0.755	0.778	0.828	0.742
288	0.971	0.773	0.763	0.834	0.751
289	0.965	0.770	0.763	0.831	0.747
290	0.965	0.755	0.775	0.829	0.745
291	0.962	0.757	0.778	0.830	0.746
292	0.957	0.783	0.749	0.828	0.742
293	0.965	0.778	0.760	0.833	0.750
294	0.974	0.767	0.757	0.831	0.747
295	0.960	0.775	0.740	0.824	0.735
296	0.965	0.755	0.775	0.829	0.745
297	0.965	0.747	0.787	0.830	0.747
298	0.957	0.778	0.775	0.835	0.753
299	0.960	0.780	0.760	0.832	0.748
300	0.960	0.765	0.784	0.834	0.752
301	0.954	0.786	0.760	0.832	0.748
302	0.965	0.780	0.775	0.839	0.758
303	0.960	0.773	0.781	0.836	0.754
304	0.962	0.752	0.790	0.832	0.749
305	0.960	0.767	0.751	0.825	0.737
306	0.962	0.744	0.781	0.827	0.741
307	0.960	0.773	0.772	0.833	0.750
308	0.957	0.773	0.781	0.835	0.753
309	0.960	0.736	0.796	0.828	0.743
310	0.965	0.762	0.790	0.837	0.756
311	0.968	0.760	0.793	0.838	0.758
312	0.962	0.744	0.790	0.829	0.746

313	0.968	0.762	0.763	0.829	0.744
314	0.960	0.755	0.784	0.830	0.746
315	0.962	0.765	0.760	0.828	0.741
316	0.960	0.760	0.790	0.834	0.752
317	0.965	0.757	0.775	0.830	0.746
318	0.960	0.762	0.769	0.828	0.743
319	0.954	0.757	0.760	0.822	0.733
320	0.965	0.767	0.775	0.834	0.751
321	0.968	0.760	0.743	0.822	0.733
322	0.957	0.755	0.799	0.834	0.752
323	0.965	0.767	0.781	0.836	0.754
324	0.948	0.762	0.787	0.830	0.746
325	0.965	0.762	0.766	0.829	0.744
326	0.965	0.749	0.778	0.828	0.744
327	0.954	0.757	0.760	0.822	0.733
328	0.948	0.780	0.749	0.825	0.737
329	0.960	0.752	0.796	0.833	0.751
330	0.957	0.770	0.787	0.836	0.754
331	0.957	0.767	0.784	0.834	0.752
332	0.960	0.770	0.784	0.836	0.754
333	0.965	0.767	0.781	0.836	0.754
334	0.954	0.752	0.754	0.818	0.727
335	0.965	0.765	0.778	0.834	0.752
336	0.962	0.770	0.763	0.830	0.746
337	0.951	0.770	0.775	0.830	0.746
338	0.960	0.778	0.778	0.837	0.755
339	0.962	0.757	0.763	0.826	0.739
340	0.965	0.724	0.790	0.823	0.736
341	0.965	0.757	0.808	0.841	0.762
342	0.962	0.752	0.772	0.827	0.741
343	0.960	0.755	0.790	0.832	0.750
344	0.954	0.739	0.778	0.821	0.733
345	0.962	0.760	0.784	0.833	0.750
346	0.951	0.760	0.772	0.826	0.739
347	0.965	0.760	0.793	0.837	0.756
348	0.960	0.749	0.799	0.833	0.751
349	0.951	0.770	0.787	0.834	0.752
350	0.957	0.762	0.778	0.830	0.746
351	0.968	0.760	0.772	0.831	0.747
352	0.962	0.767	0.778	0.834	0.752
353	0.962	0.770	0.746	0.825	0.737
354	0.948	0.765	0.772	0.827	0.740

355	0.962	0.770	0.772	0.833	0.750
356	0.968	0.786	0.754	0.835	0.752
357	0.957	0.752	0.760	0.821	0.732
358	0.954	0.770	0.763	0.828	0.741
359	0.951	0.770	0.772	0.829	0.744
360	0.957	0.773	0.760	0.828	0.743
361	0.962	0.765	0.769	0.830	0.746
362	0.954	0.752	0.769	0.823	0.735
363	0.960	0.757	0.766	0.826	0.739
364	0.974	0.744	0.760	0.824	0.736
365	0.957	0.760	0.760	0.824	0.736
366	0.962	0.755	0.769	0.827	0.741
367	0.965	0.749	0.784	0.830	0.747
368	0.957	0.739	0.769	0.819	0.730
369	0.945	0.778	0.763	0.828	0.741
370	0.974	0.734	0.802	0.833	0.752
371	0.948	0.762	0.784	0.829	0.745
372	0.962	0.778	0.778	0.838	0.757
373	0.954	0.757	0.769	0.825	0.738
374	0.962	0.760	0.781	0.832	0.749
375	0.951	0.755	0.775	0.825	0.738
376	0.960	0.742	0.760	0.818	0.728
377	0.954	0.755	0.766	0.823	0.735
378	0.954	0.762	0.772	0.828	0.742
379	0.951	0.762	0.772	0.827	0.740
380	0.951	0.755	0.746	0.815	0.723
381	0.965	0.767	0.760	0.829	0.744
382	0.951	0.765	0.769	0.827	0.740
383	0.957	0.775	0.763	0.830	0.746
384	0.962	0.760	0.778	0.831	0.748
385	0.951	0.773	0.784	0.834	0.752
386	0.962	0.755	0.772	0.828	0.742
387	0.962	0.767	0.778	0.834	0.752
388	0.951	0.765	0.787	0.832	0.749
389	0.957	0.757	0.766	0.825	0.738
390	0.948	0.762	0.778	0.828	0.742
391	0.954	0.749	0.772	0.823	0.735
392	0.960	0.767	0.763	0.828	0.743
393	0.954	0.762	0.793	0.834	0.752
394	0.960	0.752	0.778	0.828	0.742
395	0.971	0.775	0.775	0.839	0.759
396	0.954	0.767	0.760	0.826	0.739

397	0.960	0.760	0.769	0.828	0.742
398	0.957	0.760	0.778	0.829	0.745
399	0.957	0.742	0.790	0.827	0.742
400	0.954	0.757	0.793	0.832	0.749
401	0.962	0.775	0.763	0.832	0.748
402	0.965	0.783	0.763	0.836	0.754
403	0.957	0.760	0.790	0.833	0.751
404	0.971	0.744	0.769	0.826	0.739
405	0.948	0.765	0.787	0.831	0.748
406	0.960	0.749	0.802	0.834	0.753
407	0.960	0.765	0.781	0.833	0.750
408	0.968	0.765	0.778	0.835	0.753
409	0.962	0.744	0.775	0.825	0.738
410	0.962	0.770	0.778	0.835	0.753
411	0.957	0.773	0.775	0.833	0.750
412	0.951	0.773	0.790	0.836	0.755
413	0.957	0.765	0.775	0.830	0.746
414	0.954	0.773	0.802	0.841	0.762
415	0.948	0.755	0.772	0.823	0.735
416	0.948	0.739	0.778	0.819	0.730
417	0.951	0.755	0.784	0.828	0.742
418	0.954	0.749	0.787	0.828	0.743
419	0.939	0.762	0.787	0.828	0.742
420	0.942	0.767	0.749	0.818	0.727
421	0.939	0.721	0.799	0.816	0.727
422	0.948	0.775	0.766	0.828	0.743
423	0.957	0.783	0.769	0.835	0.753
424	0.954	0.773	0.766	0.829	0.744
425	0.931	0.773	0.775	0.825	0.738
426	0.957	0.770	0.763	0.828	0.743
427	0.931	0.775	0.775	0.826	0.739
428	0.951	0.780	0.760	0.829	0.744
429	0.945	0.757	0.778	0.825	0.738
430	0.945	0.775	0.781	0.832	0.749
431	0.945	0.755	0.775	0.823	0.735
432	0.954	0.770	0.778	0.832	0.749
433	0.948	0.770	0.757	0.824	0.736
434	0.957	0.783	0.754	0.830	0.745
435	0.965	0.765	0.766	0.830	0.746
436	0.942	0.762	0.769	0.823	0.735
437	0.945	0.760	0.787	0.828	0.744
438	0.948	0.757	0.775	0.825	0.738

439	0.948	0.755	0.769	0.822	0.734
440	0.957	0.773	0.793	0.839	0.759
441	0.942	0.762	0.772	0.824	0.736
442	0.960	0.773	0.757	0.828	0.743
443	0.971	0.767	0.754	0.829	0.744
444	0.954	0.739	0.790	0.825	0.739
445	0.951	0.749	0.796	0.829	0.746
446	0.948	0.770	0.775	0.829	0.745
447	0.962	0.765	0.790	0.837	0.756
448	0.954	0.762	0.781	0.830	0.746
449	0.942	0.773	0.749	0.820	0.730
450	0.954	0.783	0.751	0.828	0.743
451	0.954	0.765	0.751	0.822	0.733
452	0.957	0.773	0.793	0.839	0.759
453	0.957	0.762	0.775	0.829	0.745
454	0.948	0.773	0.754	0.824	0.736
455	0.957	0.765	0.766	0.828	0.742
456	0.945	0.744	0.781	0.821	0.733
457	0.951	0.757	0.766	0.823	0.735
458	0.948	0.747	0.769	0.819	0.730
459	0.960	0.749	0.754	0.819	0.729
460	0.954	0.765	0.781	0.831	0.748
461	0.954	0.760	0.754	0.821	0.732
462	0.957	0.755	0.749	0.818	0.727
463	0.954	0.755	0.784	0.828	0.744
464	0.960	0.767	0.766	0.829	0.744
465	0.957	0.757	0.769	0.826	0.739
466	0.951	0.773	0.778	0.832	0.749
467	0.954	0.755	0.757	0.820	0.730
468	0.942	0.773	0.772	0.828	0.742
469	0.945	0.762	0.751	0.818	0.728
470	0.960	0.742	0.778	0.824	0.737
471	0.954	0.762	0.763	0.825	0.737
472	0.954	0.755	0.772	0.825	0.738
473	0.948	0.747	0.793	0.827	0.742
474	0.962	0.744	0.781	0.827	0.741
475	0.945	0.760	0.802	0.833	0.751
476	0.939	0.713	0.793	0.812	0.720
477	0.957	0.744	0.769	0.821	0.732
478	0.960	0.765	0.775	0.831	0.747
479	0.962	0.749	0.760	0.822	0.733
480	0.942	0.755	0.760	0.817	0.726

481	0.948	0.762	0.778	0.828	0.742
482	0.936	0.760	0.754	0.815	0.723
483	0.936	0.752	0.751	0.812	0.718
484	0.942	0.778	0.757	0.825	0.737
485	0.934	0.742	0.749	0.806	0.709
486	0.954	0.780	0.778	0.836	0.754
487	0.942	0.773	0.778	0.829	0.744
488	0.934	0.755	0.757	0.813	0.721
489	0.931	0.762	0.772	0.820	0.731
490	0.934	0.744	0.746	0.806	0.709
491	0.939	0.760	0.740	0.812	0.717
492	0.919	0.762	0.754	0.811	0.716
493	0.942	0.739	0.769	0.814	0.723
494	0.934	0.757	0.775	0.820	0.731
495	0.957	0.762	0.766	0.827	0.740
496	0.939	0.762	0.772	0.823	0.735
497	0.939	0.780	0.766	0.828	0.741
498	0.934	0.760	0.749	0.813	0.719
499	0.928	0.760	0.772	0.818	0.728
500	0.925	0.767	0.769	0.819	0.729
501	0.931	0.765	0.784	0.825	0.738
502	0.948	0.762	0.793	0.832	0.749
503	0.942	0.752	0.766	0.818	0.728
504	0.928	0.736	0.787	0.814	0.723
505	0.942	0.770	0.769	0.826	0.739
506	0.939	0.760	0.766	0.820	0.730
507	0.934	0.765	0.793	0.828	0.744
508	0.934	0.757	0.787	0.824	0.737
509	0.905	0.760	0.784	0.814	0.723
510	0.922	0.760	0.772	0.816	0.725
511	0.925	0.762	0.787	0.823	0.735
512	0.922	0.760	0.775	0.817	0.727
513	0.928	0.767	0.772	0.821	0.732
514	0.934	0.778	0.796	0.834	0.752
515	0.931	0.760	0.763	0.816	0.725
516	0.928	0.773	0.763	0.820	0.730
517	0.928	0.760	0.781	0.821	0.732
518	0.922	0.767	0.787	0.824	0.737
519	0.936	0.739	0.793	0.820	0.732
520	0.919	0.770	0.787	0.824	0.737
521	0.928	0.775	0.751	0.817	0.726
522	0.939	0.749	0.763	0.815	0.724

523	0.928	0.775	0.796	0.831	0.748
524	0.939	0.767	0.763	0.822	0.733
525	0.936	0.770	0.787	0.829	0.745
526	0.939	0.773	0.778	0.828	0.743
527	0.919	0.749	0.784	0.815	0.725
528	0.939	0.765	0.772	0.824	0.736
529	0.942	0.765	0.805	0.835	0.754
530	0.919	0.760	0.787	0.820	0.731
531	0.916	0.734	0.796	0.813	0.721
532	0.936	0.744	0.793	0.822	0.734
533	0.928	0.778	0.763	0.822	0.733
534	0.922	0.767	0.778	0.821	0.732
535	0.931	0.778	0.775	0.827	0.740
536	0.916	0.765	0.757	0.812	0.718
537	0.928	0.757	0.781	0.820	0.731
538	0.925	0.760	0.796	0.825	0.738
539	0.931	0.762	0.766	0.818	0.728
540	0.919	0.755	0.760	0.810	0.716
541	0.931	0.752	0.778	0.818	0.728
542	0.939	0.739	0.754	0.809	0.714
543	0.905	0.734	0.772	0.801	0.704
544	0.913	0.752	0.787	0.815	0.725
545	0.922	0.747	0.802	0.821	0.734
546	0.913	0.773	0.766	0.816	0.725
547	0.934	0.762	0.757	0.816	0.725
548	0.925	0.747	0.784	0.816	0.726
549	0.939	0.747	0.784	0.821	0.733
550	0.919	0.736	0.802	0.816	0.727
551	0.925	0.770	0.763	0.818	0.728
552	0.928	0.770	0.769	0.821	0.732
553	0.913	0.757	0.749	0.805	0.708
554	0.919	0.757	0.799	0.823	0.736
555	0.931	0.747	0.778	0.816	0.726
556	0.931	0.724	0.766	0.804	0.708
557	0.925	0.747	0.769	0.812	0.719
558	0.945	0.742	0.760	0.813	0.721
559	0.928	0.736	0.802	0.819	0.731
560	0.913	0.765	0.787	0.820	0.731
561	0.916	0.739	0.784	0.811	0.718
562	0.928	0.739	0.757	0.806	0.710
563	0.905	0.770	0.754	0.809	0.714
564	0.913	0.749	0.808	0.821	0.734

565	0.922	0.752	0.775	0.814	0.723
566	0.928	0.734	0.778	0.811	0.718
567	0.925	0.770	0.751	0.814	0.722
568	0.925	0.749	0.760	0.810	0.715
569	0.925	0.729	0.781	0.809	0.715
570	0.925	0.742	0.796	0.818	0.729
571	0.919	0.760	0.751	0.809	0.714
572	0.931	0.739	0.793	0.818	0.729
573	0.925	0.749	0.769	0.813	0.720
574	0.939	0.757	0.763	0.818	0.728
575	0.931	0.734	0.790	0.815	0.725
576	0.934	0.757	0.772	0.819	0.729
577	0.925	0.731	0.781	0.810	0.717
578	0.934	0.770	0.769	0.823	0.735
579	0.931	0.760	0.784	0.823	0.735
580	0.936	0.724	0.763	0.805	0.709
581	0.936	0.752	0.760	0.814	0.722
582	0.931	0.742	0.763	0.810	0.716
583	0.931	0.747	0.754	0.809	0.714
584	0.928	0.757	0.760	0.813	0.721
585	0.931	0.770	0.775	0.824	0.736
586	0.931	0.742	0.790	0.818	0.729
587	0.928	0.742	0.778	0.813	0.722
588	0.931	0.742	0.769	0.812	0.719
589	0.919	0.757	0.775	0.815	0.724
590	0.919	0.739	0.790	0.813	0.722
591	0.919	0.755	0.740	0.803	0.705
592	0.934	0.705	0.766	0.799	0.700
593	0.945	0.744	0.772	0.818	0.728
594	0.922	0.742	0.787	0.814	0.723
595	0.934	0.757	0.772	0.819	0.729
596	0.922	0.744	0.769	0.810	0.716
597	0.928	0.736	0.763	0.807	0.712
598	0.919	0.734	0.781	0.809	0.715
599	0.934	0.752	0.784	0.821	0.733
600	0.925	0.734	0.772	0.808	0.714
601	0.936	0.770	0.743	0.815	0.723
602	0.919	0.747	0.769	0.810	0.716
603	0.931	0.721	0.781	0.808	0.714
604	0.919	0.755	0.757	0.809	0.714
605	0.928	0.747	0.775	0.814	0.723
606	0.931	0.747	0.757	0.810	0.715

607	0.925	0.734	0.760	0.804	0.707
608	0.922	0.739	0.757	0.804	0.707
609	0.939	0.752	0.769	0.818	0.728
610	0.902	0.744	0.772	0.804	0.708
611	0.928	0.731	0.790	0.813	0.722
612	0.928	0.747	0.772	0.813	0.721
613	0.913	0.760	0.760	0.810	0.715
614	0.916	0.742	0.775	0.809	0.715
615	0.916	0.744	0.772	0.809	0.715
616	0.913	0.734	0.769	0.803	0.706
617	0.905	0.731	0.775	0.801	0.704
618	0.919	0.770	0.769	0.818	0.728
619	0.922	0.749	0.766	0.811	0.717
620	0.936	0.747	0.769	0.815	0.724
621	0.916	0.747	0.763	0.807	0.711
622	0.910	0.760	0.766	0.811	0.717
623	0.902	0.726	0.757	0.793	0.691
624	0.925	0.731	0.754	0.801	0.703
625	0.916	0.742	0.775	0.809	0.715
626	0.908	0.755	0.778	0.812	0.719
627	0.919	0.744	0.781	0.813	0.720
628	0.910	0.747	0.769	0.807	0.712
629	0.925	0.734	0.781	0.811	0.718
630	0.910	0.716	0.784	0.800	0.704
631	0.925	0.757	0.772	0.816	0.725
632	0.910	0.736	0.769	0.803	0.706
633	0.922	0.731	0.766	0.804	0.708
634	0.925	0.755	0.749	0.808	0.712
635	0.905	0.726	0.760	0.795	0.694
636	0.931	0.747	0.769	0.813	0.721
637	0.908	0.744	0.760	0.802	0.705
638	0.931	0.749	0.787	0.820	0.731
639	0.916	0.755	0.760	0.809	0.714
640	0.942	0.757	0.757	0.817	0.726
641	0.919	0.765	0.763	0.814	0.722
642	0.922	0.718	0.757	0.797	0.696
643	0.931	0.742	0.766	0.811	0.717
644	0.910	0.744	0.790	0.813	0.721
645	0.925	0.695	0.769	0.793	0.692
646	0.922	0.708	0.778	0.799	0.702
647	0.919	0.749	0.778	0.813	0.722
648	0.910	0.760	0.749	0.805	0.708

649	0.922	0.755	0.802	0.824	0.737
650	0.922	0.765	0.769	0.817	0.726
651	0.919	0.731	0.787	0.810	0.717
652	0.916	0.752	0.751	0.805	0.708
653	0.934	0.755	0.757	0.813	0.721
654	0.928	0.749	0.757	0.810	0.715
655	0.910	0.749	0.763	0.806	0.710
656	0.928	0.765	0.749	0.813	0.719
657	0.910	0.736	0.772	0.804	0.708
658	0.908	0.749	0.760	0.804	0.707
659	0.925	0.765	0.757	0.814	0.722
660	0.919	0.760	0.763	0.813	0.719
661	0.922	0.747	0.772	0.812	0.718
662	0.919	0.765	0.734	0.805	0.707
663	0.910	0.744	0.772	0.807	0.712
664	0.922	0.705	0.778	0.799	0.701
665	0.916	0.736	0.778	0.808	0.714
666	0.919	0.736	0.763	0.804	0.708
667	0.910	0.739	0.754	0.799	0.700
668	0.928	0.747	0.778	0.815	0.724
669	0.916	0.742	0.751	0.801	0.703
670	0.913	0.747	0.781	0.812	0.719
671	0.939	0.749	0.751	0.812	0.718
672	0.899	0.718	0.787	0.799	0.701
673	0.919	0.762	0.743	0.807	0.710
674	0.910	0.734	0.787	0.808	0.714
675	0.922	0.724	0.772	0.803	0.707
676	0.910	0.724	0.784	0.803	0.707
677	0.922	0.760	0.781	0.819	0.730
678	0.905	0.729	0.772	0.799	0.701
679	0.916	0.711	0.787	0.801	0.705
680	0.887	0.742	0.760	0.795	0.694
681	0.902	0.734	0.790	0.806	0.712
682	0.913	0.731	0.754	0.798	0.698
683	0.910	0.739	0.787	0.810	0.717
684	0.919	0.755	0.781	0.816	0.725
685	0.910	0.747	0.787	0.813	0.721
686	0.919	0.731	0.751	0.799	0.699
687	0.928	0.724	0.787	0.810	0.717
688	0.922	0.742	0.781	0.813	0.720
689	0.910	0.749	0.772	0.809	0.714
690	0.936	0.742	0.757	0.810	0.715

691	0.905	0.742	0.760	0.800	0.702
692	0.905	0.752	0.769	0.807	0.712
693	0.908	0.731	0.760	0.798	0.698
694	0.905	0.742	0.749	0.797	0.696
695	0.922	0.731	0.775	0.807	0.712
696	0.925	0.729	0.763	0.803	0.706
697	0.908	0.747	0.763	0.804	0.707
698	0.928	0.724	0.775	0.806	0.711
699	0.910	0.718	0.769	0.797	0.697
700	0.913	0.729	0.766	0.800	0.702
701	0.902	0.731	0.754	0.794	0.692
702	0.916	0.731	0.751	0.798	0.697
703	0.908	0.744	0.772	0.806	0.710
704	0.899	0.724	0.772	0.796	0.696
705	0.922	0.755	0.743	0.805	0.708
706	0.905	0.752	0.763	0.805	0.709
707	0.916	0.755	0.781	0.815	0.724
708	0.928	0.742	0.760	0.808	0.713
709	0.934	0.711	0.784	0.806	0.712
710	0.928	0.739	0.763	0.808	0.713
711	0.905	0.744	0.769	0.804	0.708
712	0.934	0.744	0.763	0.812	0.718
713	0.925	0.760	0.749	0.810	0.715
714	0.945	0.744	0.772	0.818	0.728
715	0.902	0.752	0.757	0.802	0.704
716	0.913	0.749	0.772	0.810	0.716
717	0.922	0.744	0.757	0.806	0.710
718	0.913	0.736	0.760	0.801	0.703
719	0.908	0.739	0.787	0.809	0.715
720	0.896	0.749	0.760	0.800	0.702
721	0.922	0.721	0.781	0.805	0.710
722	0.905	0.770	0.757	0.810	0.715
723	0.916	0.757	0.751	0.807	0.711
724	0.922	0.755	0.778	0.816	0.726
725	0.919	0.724	0.769	0.801	0.704
726	0.916	0.742	0.805	0.818	0.730
727	0.905	0.747	0.790	0.812	0.719
728	0.916	0.752	0.749	0.804	0.707
729	0.919	0.731	0.772	0.805	0.709
730	0.910	0.742	0.766	0.804	0.707
731	0.931	0.734	0.784	0.813	0.722
732	0.905	0.736	0.769	0.801	0.704

733	0.925	0.711	0.772	0.799	0.701
734	0.936	0.744	0.760	0.812	0.718
735	0.913	0.724	0.799	0.809	0.716
736	0.916	0.744	0.778	0.811	0.717
737	0.899	0.726	0.766	0.795	0.694
738	0.919	0.744	0.769	0.809	0.714
739	0.913	0.747	0.775	0.810	0.716
740	0.908	0.690	0.769	0.785	0.682
741	0.910	0.713	0.787	0.800	0.704
742	0.925	0.729	0.772	0.806	0.711
743	0.919	0.726	0.781	0.806	0.711
744	0.890	0.734	0.751	0.790	0.686
745	0.942	0.721	0.760	0.805	0.709
746	0.919	0.734	0.760	0.802	0.705
747	0.908	0.713	0.757	0.790	0.687
748	0.893	0.744	0.775	0.802	0.705
749	0.910	0.731	0.751	0.796	0.695
750	0.910	0.708	0.772	0.794	0.693
751	0.919	0.739	0.772	0.808	0.713
752	0.922	0.744	0.754	0.805	0.708
753	0.908	0.752	0.769	0.808	0.713
754	0.919	0.726	0.772	0.803	0.707
755	0.908	0.736	0.763	0.800	0.702
756	0.916	0.731	0.772	0.804	0.708
757	0.910	0.726	0.760	0.797	0.697
758	0.934	0.747	0.763	0.813	0.720
759	0.925	0.716	0.760	0.798	0.698
760	0.910	0.734	0.754	0.798	0.698
761	0.913	0.716	0.737	0.786	0.681
762	0.908	0.734	0.766	0.800	0.702
763	0.916	0.731	0.757	0.799	0.700
764	0.916	0.729	0.784	0.807	0.713
765	0.910	0.739	0.772	0.805	0.709
766	0.913	0.716	0.772	0.798	0.698
767	0.922	0.744	0.784	0.814	0.723
768	0.902	0.755	0.757	0.803	0.705
769	0.890	0.736	0.754	0.792	0.689
770	0.910	0.729	0.769	0.800	0.702
771	0.908	0.742	0.769	0.804	0.707
772	0.922	0.747	0.763	0.809	0.714
773	0.919	0.705	0.778	0.798	0.699
774	0.896	0.729	0.754	0.791	0.688

775	0.922	0.736	0.763	0.805	0.709
776	0.905	0.736	0.754	0.797	0.696
777	0.928	0.721	0.793	0.811	0.719
778	0.905	0.742	0.757	0.799	0.700
779	0.928	0.739	0.760	0.807	0.711
780	0.916	0.757	0.731	0.800	0.700
781	0.925	0.677	0.766	0.785	0.682
782	0.905	0.713	0.772	0.794	0.693
783	0.922	0.718	0.766	0.799	0.701
784	0.910	0.747	0.749	0.800	0.701
785	0.893	0.729	0.775	0.797	0.697
786	0.919	0.700	0.766	0.792	0.691
787	0.908	0.731	0.754	0.796	0.695
788	0.925	0.711	0.769	0.799	0.700
**MCFS**					
**Numbers of features**	**G1**	**G2/M**	**S**	**ACC**	**MCC**
1	0.913	0.630	0.557	0.699	0.549
2	0.908	0.729	0.650	0.762	0.643
3	0.954	0.690	0.698	0.778	0.668
4	0.948	0.690	0.716	0.782	0.674
5	0.960	0.695	0.716	0.787	0.682
6	0.954	0.700	0.707	0.784	0.678
7	0.960	0.700	0.707	0.786	0.680
8	0.957	0.716	0.704	0.790	0.685
9	0.954	0.726	0.710	0.795	0.692
10	0.960	0.729	0.713	0.799	0.698
11	0.954	0.724	0.731	0.800	0.701
12	0.960	0.788	0.781	0.842	0.763
13	0.968	0.791	0.790	0.848	0.772
14	0.968	0.791	0.790	0.848	0.772
15	0.965	0.801	0.787	0.850	0.775
16	0.954	0.798	0.790	0.846	0.769
17	0.957	0.798	0.802	0.851	0.777
18	0.960	0.778	0.787	0.840	0.760
19	0.954	0.788	0.799	0.845	0.769
20	0.960	0.806	0.811	0.858	0.787
21	0.960	0.793	0.823	0.857	0.786
22	0.960	0.793	0.799	0.849	0.774
23	0.968	0.798	0.784	0.849	0.774
24	0.951	0.775	0.787	0.836	0.755
25	0.965	0.801	0.784	0.849	0.774
26	0.960	0.778	0.796	0.843	0.764

27	0.965	0.814	0.766	0.848	0.772
28	0.965	0.801	0.781	0.848	0.772
29	0.965	0.814	0.787	0.855	0.782
30	0.962	0.811	0.790	0.854	0.781
31	0.965	0.804	0.811	0.858	0.788
32	0.965	0.824	0.796	0.861	0.792
33	0.965	0.814	0.793	0.857	0.785
34	0.962	0.824	0.796	0.860	0.790
35	0.962	0.811	0.790	0.854	0.781
36	0.965	0.817	0.781	0.854	0.780
37	0.960	0.819	0.784	0.854	0.780
38	0.971	0.809	0.793	0.857	0.785
39	0.957	0.809	0.805	0.856	0.784
40	0.968	0.806	0.793	0.855	0.782
41	0.960	0.822	0.784	0.855	0.782
42	0.968	0.827	0.793	0.862	0.793
43	0.965	0.822	0.802	0.862	0.793
44	0.971	0.819	0.811	0.866	0.799
45	0.971	0.819	0.832	0.873	0.809
46	0.977	0.827	0.793	0.865	0.797
47	0.968	0.819	0.820	0.868	0.802
48	0.968	0.835	0.805	0.869	0.803
49	0.965	0.837	0.805	0.869	0.803
50	0.968	0.837	0.811	0.872	0.807
51	0.968	0.835	0.823	0.874	0.812
52	0.965	0.842	0.826	0.877	0.816
53	0.971	0.835	0.811	0.872	0.807
54	0.968	0.840	0.823	0.876	0.814
55	0.974	0.827	0.805	0.868	0.802
56	0.971	0.835	0.799	0.868	0.802
57	0.968	0.829	0.823	0.873	0.809
58	0.974	0.835	0.826	0.877	0.816
59	0.971	0.837	0.826	0.877	0.816
60	0.965	0.819	0.832	0.871	0.806
61	0.980	0.837	0.805	0.873	0.810
62	0.965	0.832	0.814	0.870	0.805
63	0.977	0.829	0.811	0.872	0.807
64	0.971	0.827	0.844	0.879	0.819
65	0.971	0.817	0.832	0.872	0.808
66	0.962	0.837	0.832	0.876	0.815
67	0.968	0.824	0.844	0.877	0.817
68	0.974	0.829	0.829	0.876	0.815

69	0.977	0.827	0.835	0.878	0.818
70	0.971	0.835	0.838	0.880	0.820
71	0.968	0.822	0.844	0.876	0.815
72	0.971	0.824	0.826	0.873	0.809
73	0.968	0.824	0.841	0.876	0.815
74	0.971	0.822	0.838	0.875	0.814
75	0.968	0.832	0.811	0.870	0.805
76	0.983	0.829	0.835	0.881	0.822
77	0.968	0.829	0.799	0.865	0.797
78	0.971	0.796	0.826	0.862	0.794
79	0.960	0.817	0.841	0.871	0.807
80	0.971	0.822	0.835	0.874	0.812
81	0.968	0.811	0.829	0.868	0.802
82	0.971	0.798	0.832	0.865	0.799
83	0.968	0.817	0.835	0.872	0.808
84	0.974	0.814	0.829	0.871	0.807
85	0.968	0.822	0.814	0.867	0.801
86	0.968	0.814	0.835	0.871	0.807
87	0.971	0.806	0.832	0.868	0.803
88	0.965	0.814	0.820	0.865	0.798
89	0.962	0.814	0.838	0.870	0.805
90	0.962	0.817	0.829	0.868	0.802
91	0.971	0.817	0.832	0.872	0.808
92	0.965	0.809	0.826	0.865	0.798
93	0.965	0.814	0.838	0.871	0.807
94	0.965	0.809	0.829	0.866	0.800
95	0.971	0.798	0.835	0.866	0.800
96	0.974	0.809	0.823	0.867	0.801
97	0.971	0.814	0.823	0.868	0.802
98	0.965	0.811	0.841	0.871	0.807
99	0.977	0.806	0.826	0.868	0.802
100	0.983	0.798	0.823	0.866	0.800
101	0.983	0.796	0.826	0.866	0.800
102	0.980	0.827	0.829	0.877	0.816
103	0.983	0.822	0.826	0.875	0.813
104	0.968	0.827	0.832	0.874	0.812
105	0.986	0.814	0.823	0.873	0.809
106	0.980	0.819	0.826	0.873	0.811
107	0.968	0.817	0.841	0.873	0.811
108	0.977	0.814	0.832	0.873	0.809
109	0.968	0.819	0.847	0.876	0.815
110	0.974	0.822	0.826	0.873	0.809

111	0.974	0.827	0.835	0.877	0.816
112	0.977	0.814	0.850	0.878	0.818
113	0.968	0.811	0.832	0.869	0.804
114	0.977	0.814	0.850	0.878	0.818
115	0.974	0.822	0.850	0.880	0.821
116	0.977	0.811	0.820	0.868	0.802
117	0.977	0.827	0.829	0.876	0.815
118	0.960	0.801	0.808	0.855	0.783
119	0.962	0.786	0.826	0.856	0.785
120	0.962	0.778	0.817	0.850	0.776
121	0.957	0.796	0.826	0.858	0.787
122	0.974	0.793	0.823	0.861	0.793
123	0.965	0.791	0.817	0.856	0.784
124	0.965	0.791	0.817	0.856	0.784
125	0.962	0.798	0.844	0.866	0.800
126	0.968	0.798	0.802	0.855	0.782
127	0.962	0.798	0.826	0.860	0.792
128	0.960	0.775	0.805	0.844	0.768
129	0.971	0.786	0.811	0.854	0.782
130	0.957	0.793	0.814	0.853	0.780
131	0.965	0.804	0.811	0.858	0.788
132	0.971	0.798	0.805	0.857	0.785
133	0.980	0.817	0.784	0.859	0.789
134	0.974	0.806	0.784	0.854	0.781
135	0.971	0.796	0.793	0.852	0.778
136	0.983	0.793	0.781	0.851	0.777
137	0.977	0.804	0.799	0.858	0.788
138	0.974	0.806	0.799	0.858	0.788
139	0.974	0.778	0.787	0.844	0.767
140	0.962	0.783	0.790	0.843	0.766
141	0.962	0.778	0.787	0.841	0.762
142	0.957	0.788	0.796	0.845	0.768
143	0.971	0.798	0.787	0.851	0.776
144	0.954	0.798	0.784	0.844	0.767
145	0.960	0.783	0.799	0.845	0.769
146	0.957	0.783	0.808	0.847	0.772
147	0.954	0.791	0.790	0.843	0.766
148	0.962	0.801	0.796	0.852	0.778
149	0.957	0.793	0.772	0.840	0.760
150	0.960	0.780	0.793	0.843	0.764
151	0.960	0.780	0.802	0.845	0.769
152	0.951	0.775	0.793	0.838	0.758

153	0.954	0.780	0.796	0.842	0.763
154	0.960	0.791	0.805	0.850	0.776
155	0.962	0.778	0.793	0.843	0.764
156	0.957	0.783	0.790	0.842	0.763
157	0.957	0.770	0.793	0.838	0.758
158	0.960	0.778	0.802	0.844	0.768
159	0.957	0.773	0.814	0.845	0.769
160	0.960	0.788	0.787	0.843	0.765
161	0.954	0.791	0.784	0.842	0.763
162	0.965	0.793	0.796	0.850	0.775
163	0.968	0.798	0.787	0.850	0.775
164	0.960	0.796	0.805	0.852	0.778
165	0.954	0.804	0.778	0.844	0.766
166	0.965	0.809	0.760	0.844	0.766
167	0.957	0.798	0.790	0.847	0.771
168	0.962	0.791	0.796	0.848	0.773
169	0.957	0.791	0.784	0.843	0.764
170	0.957	0.793	0.808	0.851	0.777
171	0.960	0.788	0.793	0.845	0.768
172	0.957	0.786	0.823	0.853	0.780
173	0.945	0.801	0.784	0.843	0.764
174	0.965	0.801	0.805	0.856	0.784
175	0.957	0.791	0.775	0.840	0.760
176	0.962	0.804	0.778	0.847	0.771
177	0.962	0.804	0.811	0.858	0.787
178	0.954	0.804	0.790	0.848	0.772
179	0.968	0.780	0.784	0.843	0.764
180	0.965	0.775	0.808	0.847	0.772
181	0.971	0.780	0.778	0.842	0.762
182	0.960	0.788	0.775	0.840	0.760
183	0.957	0.775	0.799	0.842	0.763
184	0.957	0.786	0.796	0.844	0.767
185	0.954	0.793	0.790	0.844	0.767
186	0.974	0.796	0.775	0.847	0.771
187	0.962	0.793	0.787	0.846	0.769
188	0.965	0.788	0.784	0.844	0.767
189	0.962	0.793	0.787	0.846	0.769
190	0.960	0.791	0.790	0.845	0.768
191	0.951	0.793	0.784	0.842	0.762
192	0.954	0.788	0.811	0.849	0.774
193	0.957	0.791	0.775	0.840	0.759
194	0.960	0.786	0.805	0.848	0.773

195	0.962	0.778	0.793	0.843	0.764
196	0.968	0.788	0.805	0.852	0.778
197	0.962	0.786	0.808	0.850	0.776
198	0.968	0.783	0.790	0.845	0.768
199	0.974	0.783	0.793	0.848	0.773
200	0.968	0.775	0.790	0.843	0.764
201	0.965	0.780	0.799	0.846	0.770
202	0.980	0.780	0.775	0.843	0.765
203	0.968	0.786	0.781	0.843	0.765
204	0.974	0.767	0.784	0.840	0.760
205	0.968	0.765	0.799	0.842	0.763
206	0.971	0.786	0.769	0.841	0.761
207	0.965	0.788	0.781	0.843	0.765
208	0.965	0.778	0.799	0.845	0.769
209	0.968	0.778	0.823	0.854	0.782
210	0.971	0.773	0.805	0.847	0.772
211	0.971	0.780	0.784	0.843	0.765
212	0.968	0.780	0.802	0.848	0.773
213	0.971	0.783	0.778	0.843	0.764
214	0.965	0.780	0.796	0.845	0.768
215	0.971	0.773	0.820	0.852	0.779
216	0.971	0.770	0.787	0.841	0.761
217	0.965	0.770	0.790	0.840	0.760
218	0.977	0.780	0.793	0.848	0.773
219	0.965	0.783	0.802	0.848	0.773
220	0.971	0.788	0.802	0.852	0.778
221	0.968	0.786	0.784	0.844	0.767
222	0.968	0.778	0.802	0.847	0.772
223	0.968	0.773	0.814	0.849	0.775
224	0.983	0.783	0.763	0.842	0.762
225	0.974	0.783	0.802	0.851	0.777
226	0.971	0.770	0.802	0.845	0.769
227	0.980	0.778	0.811	0.854	0.781
228	0.977	0.765	0.802	0.845	0.769
229	0.971	0.767	0.790	0.841	0.761
230	0.968	0.770	0.805	0.845	0.769
231	0.968	0.780	0.790	0.844	0.767
232	0.980	0.783	0.772	0.843	0.765
233	0.965	0.770	0.808	0.845	0.769
234	0.962	0.780	0.802	0.846	0.770
235	0.974	0.786	0.793	0.849	0.774
236	0.971	0.778	0.796	0.846	0.770

237	0.965	0.773	0.802	0.844	0.767
238	0.968	0.783	0.793	0.846	0.770
239	0.974	0.778	0.811	0.852	0.779
240	0.965	0.780	0.793	0.844	0.767
241	0.962	0.773	0.787	0.839	0.759
242	0.957	0.783	0.754	0.830	0.745
243	0.957	0.786	0.766	0.835	0.753
244	0.960	0.788	0.737	0.828	0.741
245	0.962	0.783	0.754	0.832	0.748
246	0.962	0.775	0.746	0.827	0.740
247	0.954	0.775	0.757	0.828	0.741
248	0.957	0.780	0.772	0.835	0.753
249	0.957	0.780	0.763	0.832	0.748
250	0.960	0.783	0.757	0.832	0.748
251	0.951	0.791	0.763	0.834	0.751
252	0.948	0.793	0.787	0.842	0.763
253	0.954	0.778	0.775	0.834	0.752
254	0.962	0.783	0.769	0.837	0.755
255	0.948	0.793	0.754	0.831	0.747
256	0.960	0.786	0.778	0.840	0.760
257	0.962	0.778	0.769	0.835	0.753
258	0.957	0.804	0.757	0.839	0.758
259	0.954	0.788	0.766	0.835	0.753
260	0.962	0.788	0.763	0.837	0.755
261	0.965	0.788	0.778	0.843	0.764
262	0.951	0.780	0.775	0.834	0.751
263	0.948	0.791	0.781	0.839	0.758
264	0.957	0.796	0.778	0.843	0.764
265	0.965	0.798	0.754	0.839	0.758
266	0.951	0.798	0.763	0.837	0.755
267	0.954	0.793	0.772	0.839	0.758
268	0.957	0.773	0.796	0.840	0.760
269	0.951	0.780	0.790	0.839	0.759
270	0.954	0.793	0.781	0.842	0.763
271	0.954	0.798	0.778	0.843	0.764
272	0.957	0.783	0.784	0.840	0.760
273	0.951	0.786	0.775	0.836	0.754
274	0.960	0.775	0.757	0.829	0.744
275	0.954	0.767	0.763	0.827	0.740
276	0.957	0.783	0.784	0.840	0.760
277	0.957	0.791	0.787	0.843	0.765
278	0.954	0.775	0.769	0.831	0.747

279	0.954	0.778	0.787	0.838	0.757
280	0.960	0.773	0.769	0.832	0.749
281	0.960	0.767	0.802	0.841	0.762
282	0.960	0.765	0.775	0.831	0.747
283	0.965	0.775	0.754	0.830	0.746
284	0.962	0.783	0.760	0.834	0.751
285	0.960	0.788	0.775	0.840	0.760
286	0.962	0.770	0.787	0.838	0.758
287	0.954	0.788	0.766	0.835	0.753
288	0.954	0.783	0.772	0.835	0.753
289	0.951	0.778	0.778	0.834	0.751
290	0.948	0.786	0.751	0.828	0.741
291	0.951	0.786	0.766	0.833	0.750
292	0.960	0.780	0.778	0.838	0.757
293	0.948	0.788	0.760	0.831	0.747
294	0.957	0.778	0.763	0.831	0.747
295	0.965	0.775	0.757	0.831	0.747
296	0.948	0.793	0.749	0.829	0.744
297	0.960	0.778	0.790	0.841	0.761
298	0.957	0.780	0.775	0.836	0.754
299	0.954	0.786	0.775	0.837	0.755
300	0.957	0.783	0.769	0.835	0.753
301	0.954	0.773	0.772	0.831	0.747
302	0.962	0.780	0.769	0.836	0.754
303	0.954	0.773	0.763	0.828	0.743
304	0.962	0.773	0.790	0.840	0.760
305	0.960	0.780	0.766	0.834	0.751
306	0.954	0.780	0.775	0.835	0.753
307	0.962	0.796	0.784	0.846	0.770
308	0.962	0.786	0.808	0.850	0.776
309	0.954	0.786	0.757	0.831	0.747
310	0.960	0.791	0.734	0.828	0.741
311	0.960	0.783	0.775	0.838	0.757
312	0.951	0.786	0.766	0.833	0.750
313	0.957	0.773	0.781	0.835	0.753
314	0.962	0.767	0.775	0.833	0.750
315	0.951	0.788	0.775	0.837	0.755
316	0.957	0.780	0.757	0.830	0.746
317	0.960	0.786	0.775	0.839	0.758
318	0.954	0.796	0.769	0.839	0.758
319	0.954	0.780	0.769	0.833	0.750
320	0.960	0.788	0.781	0.842	0.763

321	0.957	0.780	0.772	0.835	0.753
322	0.968	0.762	0.799	0.841	0.762
323	0.960	0.788	0.772	0.839	0.758
324	0.965	0.780	0.787	0.843	0.764
325	0.957	0.780	0.787	0.840	0.760
326	0.957	0.770	0.772	0.831	0.747
327	0.954	0.755	0.790	0.830	0.747
328	0.960	0.767	0.781	0.834	0.752
329	0.957	0.786	0.793	0.843	0.766
330	0.954	0.783	0.775	0.836	0.754
331	0.971	0.775	0.760	0.834	0.751
332	0.962	0.786	0.763	0.836	0.754
333	0.960	0.791	0.772	0.840	0.760
334	0.965	0.780	0.766	0.836	0.754
335	0.957	0.786	0.793	0.843	0.766
336	0.957	0.775	0.757	0.828	0.743
337	0.965	0.780	0.751	0.831	0.747
338	0.954	0.752	0.781	0.827	0.741
339	0.954	0.780	0.769	0.833	0.750
340	0.957	0.775	0.769	0.832	0.749
341	0.960	0.765	0.772	0.830	0.746
342	0.957	0.767	0.769	0.829	0.745
343	0.960	0.783	0.757	0.832	0.748
344	0.948	0.786	0.743	0.825	0.737
345	0.951	0.773	0.766	0.828	0.743
346	0.960	0.788	0.790	0.844	0.767
347	0.954	0.788	0.740	0.827	0.739
348	0.951	0.788	0.781	0.839	0.758
349	0.957	0.798	0.781	0.844	0.766
350	0.957	0.791	0.793	0.845	0.768
351	0.951	0.783	0.784	0.838	0.757
352	0.957	0.786	0.766	0.835	0.752
353	0.954	0.778	0.772	0.833	0.750
354	0.954	0.786	0.769	0.835	0.752
355	0.960	0.804	0.775	0.845	0.768
356	0.968	0.770	0.784	0.839	0.759
357	0.957	0.788	0.790	0.843	0.765
358	0.960	0.783	0.793	0.843	0.766
359	0.960	0.788	0.763	0.836	0.754
360	0.954	0.783	0.778	0.837	0.755
361	0.962	0.788	0.769	0.839	0.758
362	0.954	0.783	0.790	0.841	0.761

363	0.948	0.778	0.778	0.833	0.750
364	0.936	0.786	0.781	0.833	0.750
365	0.931	0.806	0.772	0.836	0.754
366	0.942	0.793	0.778	0.837	0.755
367	0.942	0.788	0.778	0.835	0.753
368	0.939	0.791	0.769	0.832	0.748
369	0.948	0.786	0.778	0.836	0.754
370	0.942	0.801	0.769	0.837	0.755
371	0.939	0.798	0.781	0.839	0.758
372	0.951	0.798	0.766	0.838	0.756
373	0.948	0.770	0.781	0.831	0.747
374	0.948	0.796	0.769	0.837	0.755
375	0.954	0.793	0.781	0.842	0.762
376	0.942	0.793	0.760	0.831	0.747
377	0.945	0.793	0.781	0.839	0.758
378	0.951	0.791	0.778	0.839	0.758
379	0.942	0.783	0.796	0.839	0.759
380	0.948	0.798	0.781	0.842	0.762
381	0.962	0.773	0.775	0.835	0.753
382	0.954	0.765	0.772	0.828	0.743
383	0.960	0.783	0.754	0.831	0.747
384	0.951	0.775	0.743	0.822	0.733
385	0.948	0.778	0.757	0.827	0.740
386	0.948	0.770	0.769	0.828	0.742
387	0.948	0.783	0.749	0.826	0.738
388	0.951	0.767	0.778	0.830	0.746
389	0.945	0.780	0.778	0.833	0.750
390	0.951	0.762	0.766	0.825	0.738
391	0.960	0.755	0.787	0.831	0.748
392	0.951	0.778	0.793	0.839	0.759
393	0.945	0.778	0.790	0.836	0.754
394	0.945	0.755	0.787	0.827	0.741
395	0.948	0.770	0.784	0.832	0.749
396	0.945	0.775	0.784	0.833	0.750
397	0.960	0.788	0.754	0.833	0.749
398	0.951	0.770	0.799	0.838	0.758
399	0.954	0.780	0.784	0.838	0.757
400	0.951	0.770	0.772	0.829	0.744
401	0.954	0.783	0.790	0.841	0.761
402	0.951	0.788	0.775	0.837	0.755
403	0.948	0.767	0.775	0.828	0.743
404	0.951	0.786	0.769	0.834	0.751

405	0.954	0.786	0.793	0.843	0.764
406	0.957	0.778	0.775	0.835	0.753
407	0.971	0.767	0.775	0.836	0.754
408	0.945	0.775	0.787	0.834	0.752
409	0.957	0.778	0.775	0.835	0.753
410	0.960	0.780	0.799	0.844	0.767
411	0.957	0.770	0.775	0.832	0.749
412	0.948	0.770	0.796	0.836	0.755
413	0.948	0.783	0.784	0.837	0.756
414	0.948	0.783	0.778	0.835	0.753
415	0.945	0.780	0.778	0.833	0.750
416	0.957	0.775	0.784	0.837	0.756
417	0.951	0.773	0.799	0.839	0.759
418	0.962	0.778	0.778	0.838	0.757
419	0.951	0.780	0.790	0.839	0.759
420	0.954	0.783	0.781	0.838	0.757
421	0.951	0.778	0.799	0.841	0.762
422	0.951	0.773	0.784	0.834	0.752
423	0.948	0.773	0.784	0.833	0.750
424	0.948	0.786	0.769	0.833	0.750
425	0.925	0.780	0.749	0.817	0.726
426	0.931	0.783	0.763	0.825	0.737
427	0.942	0.773	0.772	0.828	0.742
428	0.957	0.770	0.781	0.834	0.752
429	0.928	0.773	0.784	0.827	0.741
430	0.951	0.775	0.778	0.833	0.750
431	0.954	0.765	0.781	0.831	0.748
432	0.948	0.788	0.781	0.838	0.757
433	0.951	0.767	0.778	0.830	0.746
434	0.960	0.765	0.760	0.827	0.740
435	0.945	0.775	0.784	0.833	0.750
436	0.954	0.780	0.769	0.833	0.750
437	0.951	0.780	0.766	0.831	0.747
438	0.948	0.788	0.778	0.837	0.755
439	0.957	0.775	0.772	0.833	0.750
440	0.954	0.770	0.746	0.822	0.733
441	0.942	0.783	0.778	0.833	0.750
442	0.942	0.762	0.796	0.831	0.748
443	0.948	0.791	0.778	0.838	0.757
444	0.948	0.778	0.766	0.829	0.744
445	0.954	0.786	0.766	0.834	0.751
446	0.948	0.786	0.766	0.832	0.748

447	0.960	0.786	0.760	0.834	0.751
448	0.962	0.778	0.763	0.833	0.750
449	0.957	0.788	0.769	0.837	0.755
450	0.957	0.783	0.775	0.837	0.756
451	0.951	0.786	0.772	0.835	0.753
452	0.960	0.786	0.778	0.840	0.760
453	0.954	0.786	0.778	0.838	0.757
454	0.965	0.783	0.787	0.843	0.765
455	0.965	0.786	0.763	0.837	0.755
456	0.951	0.793	0.787	0.843	0.764
457	0.960	0.786	0.763	0.835	0.752
458	0.942	0.760	0.787	0.828	0.742
459	0.951	0.796	0.766	0.837	0.755
460	0.942	0.791	0.772	0.834	0.751
461	0.960	0.783	0.787	0.842	0.763
462	0.957	0.783	0.787	0.841	0.761
463	0.957	0.757	0.772	0.827	0.740
464	0.948	0.786	0.763	0.831	0.747
465	0.962	0.786	0.778	0.841	0.761
466	0.954	0.791	0.769	0.837	0.755
467	0.942	0.762	0.781	0.827	0.741
468	0.951	0.791	0.769	0.836	0.754
469	0.954	0.780	0.778	0.836	0.754
470	0.962	0.783	0.772	0.838	0.757
471	0.945	0.788	0.775	0.835	0.753
472	0.948	0.786	0.766	0.832	0.748
473	0.942	0.783	0.763	0.828	0.743
474	0.957	0.786	0.784	0.841	0.761
475	0.960	0.770	0.775	0.833	0.750
476	0.960	0.775	0.775	0.835	0.753
477	0.965	0.778	0.757	0.832	0.748
478	0.951	0.775	0.769	0.830	0.746
479	0.951	0.793	0.751	0.831	0.747
480	0.962	0.783	0.754	0.832	0.748
481	0.951	0.765	0.772	0.828	0.742
482	0.948	0.773	0.772	0.829	0.744
483	0.951	0.788	0.760	0.832	0.748
484	0.939	0.786	0.769	0.830	0.746
485	0.962	0.762	0.763	0.828	0.742
486	0.957	0.783	0.787	0.841	0.761
487	0.948	0.780	0.772	0.832	0.748
488	0.939	0.773	0.769	0.826	0.739

489	0.945	0.760	0.778	0.826	0.739
490	0.951	0.778	0.763	0.829	0.744
491	0.968	0.780	0.778	0.841	0.761
492	0.962	0.798	0.778	0.845	0.768
493	0.962	0.793	0.766	0.840	0.759
494	0.954	0.786	0.760	0.832	0.748
495	0.957	0.778	0.796	0.842	0.763
496	0.960	0.786	0.793	0.844	0.767
497	0.954	0.788	0.787	0.842	0.763
498	0.954	0.788	0.784	0.841	0.761
499	0.965	0.788	0.793	0.847	0.771
500	0.960	0.780	0.787	0.841	0.762
501	0.957	0.778	0.808	0.845	0.769
502	0.962	0.778	0.805	0.846	0.770
503	0.957	0.775	0.799	0.842	0.763
504	0.957	0.793	0.781	0.843	0.764
505	0.948	0.778	0.784	0.835	0.753
506	0.951	0.788	0.793	0.843	0.764
507	0.960	0.796	0.775	0.843	0.764
508	0.954	0.788	0.778	0.839	0.758
509	0.974	0.773	0.778	0.840	0.760
510	0.968	0.786	0.781	0.843	0.765
511	0.968	0.796	0.781	0.847	0.771
512	0.965	0.798	0.772	0.844	0.767
513	0.957	0.770	0.775	0.832	0.749
514	0.957	0.793	0.784	0.843	0.765
515	0.948	0.791	0.781	0.839	0.758
516	0.962	0.780	0.787	0.842	0.763
517	0.948	0.788	0.790	0.841	0.761
518	0.945	0.788	0.769	0.833	0.750
519	0.945	0.796	0.778	0.839	0.758
520	0.951	0.770	0.772	0.829	0.745
521	0.962	0.783	0.793	0.844	0.767
522	0.962	0.791	0.778	0.843	0.764
523	0.954	0.775	0.796	0.840	0.760
524	0.960	0.770	0.787	0.837	0.756
525	0.960	0.791	0.784	0.843	0.765
526	0.954	0.765	0.799	0.837	0.756
527	0.954	0.770	0.775	0.831	0.747
528	0.948	0.767	0.778	0.829	0.745
529	0.954	0.804	0.799	0.851	0.777
530	0.957	0.786	0.790	0.843	0.764

531	0.960	0.757	0.796	0.835	0.754
532	0.960	0.796	0.784	0.845	0.768
533	0.948	0.783	0.802	0.843	0.765
534	0.962	0.788	0.790	0.845	0.768
535	0.957	0.788	0.814	0.851	0.777
536	0.965	0.770	0.793	0.841	0.762
537	0.960	0.801	0.781	0.846	0.769
538	0.942	0.788	0.811	0.845	0.769
539	0.954	0.786	0.787	0.841	0.761
540	0.957	0.778	0.796	0.842	0.763
541	0.954	0.783	0.796	0.843	0.764
542	0.960	0.798	0.808	0.854	0.781
543	0.960	0.775	0.808	0.845	0.769
544	0.962	0.780	0.793	0.843	0.766
545	0.948	0.765	0.817	0.841	0.763
546	0.957	0.786	0.796	0.844	0.767
547	0.951	0.765	0.790	0.833	0.751
548	0.954	0.778	0.799	0.842	0.763
549	0.960	0.770	0.787	0.837	0.756
550	0.954	0.767	0.796	0.837	0.756
551	0.957	0.773	0.799	0.841	0.762
552	0.954	0.783	0.775	0.836	0.754
553	0.951	0.775	0.790	0.837	0.756
554	0.945	0.767	0.796	0.834	0.752
555	0.957	0.775	0.811	0.845	0.769
556	0.960	0.778	0.790	0.841	0.762
557	0.960	0.788	0.769	0.838	0.757
558	0.965	0.778	0.784	0.841	0.761
559	0.957	0.783	0.784	0.840	0.760
560	0.942	0.778	0.796	0.837	0.756
561	0.965	0.773	0.793	0.842	0.763
562	0.951	0.765	0.775	0.828	0.743
563	0.948	0.747	0.778	0.822	0.734
564	0.945	0.767	0.802	0.836	0.755
565	0.945	0.767	0.784	0.830	0.746
566	0.945	0.765	0.763	0.823	0.735
567	0.948	0.778	0.775	0.832	0.749
568	0.945	0.744	0.787	0.823	0.736
569	0.945	0.744	0.784	0.822	0.734
570	0.936	0.757	0.772	0.820	0.731
571	0.948	0.765	0.769	0.826	0.739
572	0.951	0.767	0.787	0.833	0.750

573	0.957	0.752	0.769	0.824	0.736
574	0.936	0.775	0.760	0.823	0.735
575	0.945	0.770	0.763	0.825	0.737
576	0.962	0.755	0.793	0.834	0.752
577	0.942	0.760	0.790	0.828	0.744
578	0.951	0.749	0.778	0.824	0.737
579	0.942	0.757	0.781	0.825	0.738
580	0.939	0.762	0.775	0.824	0.736
581	0.910	0.762	0.766	0.812	0.718
582	0.962	0.767	0.769	0.831	0.747
583	0.942	0.767	0.778	0.828	0.742
584	0.960	0.765	0.754	0.825	0.737
585	0.942	0.765	0.784	0.828	0.744
586	0.948	0.731	0.781	0.817	0.728
587	0.954	0.765	0.778	0.830	0.746
588	0.936	0.762	0.749	0.814	0.722
589	0.954	0.773	0.769	0.830	0.746
590	0.954	0.749	0.811	0.835	0.755
591	0.945	0.749	0.769	0.819	0.729
592	0.954	0.747	0.784	0.826	0.740
593	0.954	0.762	0.778	0.829	0.745
594	0.954	0.757	0.757	0.821	0.732
595	0.942	0.755	0.772	0.821	0.732
596	0.945	0.767	0.772	0.827	0.740
597	0.948	0.760	0.769	0.824	0.736
598	0.945	0.757	0.772	0.823	0.735
599	0.945	0.760	0.790	0.829	0.745
600	0.951	0.760	0.772	0.826	0.739
601	0.948	0.765	0.778	0.828	0.743
602	0.951	0.770	0.781	0.832	0.749
603	0.951	0.752	0.784	0.827	0.741
604	0.948	0.749	0.760	0.817	0.727
605	0.948	0.752	0.793	0.828	0.744
606	0.960	0.752	0.784	0.829	0.745
607	0.960	0.765	0.766	0.828	0.743
608	0.939	0.765	0.772	0.824	0.736
609	0.960	0.765	0.784	0.834	0.752
610	0.942	0.721	0.772	0.809	0.715
611	0.951	0.778	0.749	0.825	0.737
612	0.945	0.752	0.793	0.828	0.743
613	0.945	0.770	0.772	0.828	0.742
614	0.957	0.778	0.751	0.828	0.741

615	0.962	0.757	0.772	0.828	0.743
616	0.942	0.767	0.787	0.830	0.746
617	0.945	0.767	0.790	0.832	0.749
618	0.945	0.778	0.784	0.834	0.752
619	0.948	0.749	0.766	0.819	0.729
620	0.945	0.775	0.766	0.828	0.742
621	0.936	0.767	0.790	0.829	0.745
622	0.951	0.773	0.778	0.832	0.749
623	0.934	0.744	0.784	0.818	0.729
624	0.945	0.783	0.772	0.832	0.749
625	0.957	0.775	0.778	0.835	0.753
626	0.942	0.755	0.763	0.818	0.728
627	0.936	0.770	0.805	0.835	0.754
628	0.951	0.765	0.784	0.831	0.748
629	0.948	0.747	0.781	0.823	0.736
630	0.951	0.760	0.790	0.831	0.748
631	0.942	0.775	0.766	0.827	0.740
632	0.962	0.747	0.778	0.827	0.741
633	0.942	0.762	0.778	0.826	0.739
634	0.948	0.742	0.805	0.828	0.745
635	0.945	0.739	0.778	0.818	0.729
636	0.939	0.762	0.799	0.831	0.748
637	0.945	0.767	0.778	0.828	0.743
638	0.948	0.744	0.793	0.826	0.740
639	0.945	0.762	0.787	0.829	0.745
640	0.942	0.767	0.784	0.829	0.745
641	0.928	0.752	0.787	0.820	0.732
642	0.951	0.788	0.778	0.838	0.757
643	0.948	0.765	0.781	0.829	0.745
644	0.945	0.757	0.766	0.821	0.732
645	0.934	0.765	0.793	0.828	0.744
646	0.936	0.747	0.787	0.821	0.733
647	0.922	0.731	0.769	0.805	0.710
648	0.928	0.760	0.757	0.813	0.721
649	0.925	0.755	0.769	0.814	0.723
650	0.936	0.765	0.751	0.816	0.725
651	0.925	0.742	0.763	0.808	0.713
652	0.939	0.767	0.787	0.829	0.745
653	0.951	0.778	0.751	0.826	0.738
654	0.934	0.736	0.778	0.813	0.722
655	0.931	0.770	0.766	0.821	0.732
656	0.939	0.770	0.754	0.820	0.730

657	0.945	0.767	0.784	0.830	0.746
658	0.939	0.765	0.769	0.823	0.735
659	0.910	0.770	0.775	0.817	0.727
660	0.934	0.762	0.757	0.816	0.725
661	0.945	0.770	0.769	0.827	0.740
662	0.948	0.752	0.787	0.827	0.741
663	0.936	0.765	0.766	0.821	0.732
664	0.925	0.742	0.781	0.813	0.722
665	0.934	0.760	0.775	0.821	0.732
666	0.939	0.736	0.793	0.820	0.732
667	0.951	0.757	0.796	0.832	0.750
668	0.916	0.752	0.790	0.817	0.728
669	0.928	0.744	0.799	0.821	0.734
670	0.934	0.762	0.787	0.826	0.740
671	0.936	0.755	0.787	0.824	0.737
672	0.942	0.742	0.760	0.813	0.720
673	0.939	0.770	0.754	0.820	0.730
674	0.942	0.757	0.746	0.813	0.720
675	0.922	0.739	0.760	0.805	0.709
676	0.945	0.757	0.734	0.811	0.716
677	0.939	0.731	0.763	0.809	0.715
678	0.928	0.744	0.766	0.811	0.717
679	0.934	0.767	0.766	0.821	0.732
680	0.928	0.749	0.763	0.812	0.718
681	0.931	0.744	0.734	0.801	0.702
682	0.931	0.762	0.751	0.813	0.720
683	0.934	0.742	0.760	0.810	0.716
684	0.936	0.747	0.737	0.805	0.708
685	0.936	0.742	0.754	0.809	0.714
686	0.919	0.760	0.760	0.812	0.718
687	0.942	0.760	0.763	0.820	0.731
688	0.934	0.755	0.766	0.816	0.725
689	0.936	0.742	0.746	0.806	0.710
690	0.931	0.718	0.754	0.799	0.699
691	0.951	0.724	0.766	0.811	0.718
692	0.939	0.757	0.749	0.813	0.721
693	0.934	0.742	0.772	0.813	0.722
694	0.939	0.760	0.749	0.814	0.722
695	0.948	0.736	0.775	0.817	0.727
696	0.948	0.742	0.757	0.813	0.721
697	0.948	0.749	0.787	0.826	0.740
698	0.948	0.760	0.763	0.822	0.733

699	0.934	0.731	0.781	0.813	0.721
700	0.925	0.734	0.772	0.808	0.714
701	0.942	0.765	0.754	0.819	0.729
702	0.939	0.742	0.793	0.822	0.735
703	0.931	0.749	0.772	0.815	0.724
704	0.942	0.760	0.769	0.822	0.734
705	0.931	0.760	0.766	0.817	0.727
706	0.942	0.755	0.757	0.816	0.725
707	0.945	0.755	0.757	0.817	0.726
708	0.925	0.780	0.746	0.816	0.724
709	0.948	0.770	0.760	0.825	0.737
710	0.905	0.752	0.763	0.805	0.709
711	0.919	0.742	0.749	0.801	0.703
712	0.928	0.734	0.772	0.809	0.714
713	0.908	0.726	0.757	0.795	0.694
714	0.908	0.739	0.772	0.804	0.708
715	0.905	0.742	0.769	0.803	0.706
716	0.910	0.755	0.757	0.806	0.710
717	0.916	0.762	0.751	0.809	0.713
718	0.922	0.734	0.784	0.811	0.718
719	0.908	0.739	0.769	0.803	0.706
720	0.908	0.744	0.772	0.806	0.711
721	0.919	0.734	0.760	0.802	0.705
722	0.887	0.726	0.743	0.784	0.677
723	0.913	0.744	0.775	0.809	0.715
724	0.910	0.718	0.751	0.791	0.688
725	0.928	0.742	0.754	0.806	0.710
726	0.925	0.718	0.766	0.800	0.702
727	0.902	0.726	0.769	0.797	0.697
728	0.910	0.752	0.751	0.803	0.706
729	0.919	0.749	0.769	0.811	0.717
730	0.899	0.734	0.781	0.802	0.706
731	0.916	0.747	0.775	0.811	0.717
732	0.928	0.742	0.787	0.816	0.726
733	0.922	0.736	0.757	0.803	0.706
734	0.913	0.747	0.772	0.809	0.714
735	0.899	0.749	0.772	0.805	0.709
736	0.919	0.760	0.757	0.811	0.716
737	0.939	0.757	0.757	0.816	0.725
738	0.916	0.739	0.754	0.801	0.703
739	0.931	0.721	0.796	0.813	0.721
740	0.931	0.749	0.766	0.813	0.721

741	0.908	0.755	0.734	0.798	0.696
742	0.922	0.718	0.760	0.798	0.698
743	0.919	0.731	0.784	0.809	0.715
744	0.916	0.760	0.754	0.809	0.714
745	0.931	0.726	0.766	0.805	0.709
746	0.922	0.711	0.775	0.799	0.701
747	0.922	0.693	0.793	0.799	0.701
748	0.928	0.736	0.751	0.803	0.705
749	0.919	0.736	0.769	0.806	0.710
750	0.916	0.731	0.772	0.804	0.708
751	0.928	0.726	0.769	0.805	0.709
752	0.945	0.731	0.781	0.816	0.726
753	0.902	0.734	0.766	0.799	0.699
754	0.908	0.718	0.781	0.799	0.702
755	0.908	0.736	0.746	0.795	0.693
756	0.913	0.734	0.757	0.799	0.700
757	0.928	0.721	0.763	0.801	0.703
758	0.902	0.726	0.784	0.801	0.704
759	0.928	0.721	0.775	0.805	0.710
760	0.902	0.731	0.763	0.797	0.697
761	0.919	0.711	0.772	0.798	0.699
762	0.916	0.744	0.760	0.805	0.708
763	0.899	0.742	0.751	0.796	0.695
764	0.922	0.729	0.746	0.797	0.696
765	0.905	0.731	0.749	0.793	0.690
766	0.905	0.726	0.772	0.799	0.700
767	0.922	0.726	0.757	0.799	0.701
768	0.919	0.736	0.743	0.798	0.697
769	0.919	0.724	0.766	0.800	0.702
770	0.916	0.734	0.757	0.800	0.702
771	0.905	0.747	0.760	0.802	0.704
772	0.899	0.700	0.775	0.788	0.685
773	0.922	0.700	0.757	0.790	0.687
774	0.910	0.729	0.760	0.798	0.698
775	0.922	0.695	0.757	0.788	0.685
776	0.910	0.724	0.763	0.797	0.697
777	0.939	0.703	0.772	0.801	0.704
778	0.925	0.734	0.769	0.807	0.712
779	0.902	0.742	0.749	0.796	0.694
780	0.913	0.724	0.778	0.802	0.705
781	0.942	0.731	0.766	0.811	0.717
782	0.931	0.739	0.763	0.809	0.714

783	0.919	0.713	0.763	0.796	0.695
784	0.896	0.724	0.781	0.798	0.699
785	0.908	0.736	0.763	0.800	0.702
786	0.925	0.744	0.763	0.809	0.714
787	0.916	0.731	0.760	0.800	0.702
788	0.922	0.718	0.763	0.799	0.699
**SHAP by lightGBM**					
**Numbers of features**	**G1**	**G2/M**	**S**	**ACC**	**MCC**
1	0.913	0.643	0.578	0.710	0.566
2	0.942	0.693	0.671	0.767	0.650
3	0.939	0.685	0.653	0.757	0.636
4	0.942	0.695	0.710	0.780	0.670
5	0.957	0.682	0.704	0.778	0.668
6	0.957	0.685	0.728	0.786	0.682
7	0.942	0.713	0.731	0.793	0.690
8	0.962	0.749	0.751	0.819	0.729
9	0.948	0.780	0.760	0.828	0.743
10	0.960	0.793	0.802	0.850	0.775
11	0.954	0.788	0.790	0.843	0.764
12	0.965	0.811	0.778	0.851	0.776
13	0.962	0.817	0.790	0.856	0.783
14	0.962	0.824	0.790	0.858	0.788
15	0.965	0.819	0.790	0.858	0.786
16	0.960	0.809	0.802	0.856	0.784
17	0.971	0.811	0.784	0.855	0.782
18	0.977	0.804	0.823	0.866	0.800
19	0.971	0.809	0.787	0.855	0.782
20	0.971	0.806	0.808	0.860	0.791
21	0.968	0.804	0.808	0.858	0.788
22	0.983	0.804	0.811	0.864	0.797
23	0.977	0.809	0.811	0.864	0.796
24	0.977	0.801	0.814	0.862	0.794
25	0.971	0.798	0.820	0.861	0.793
26	0.960	0.806	0.814	0.858	0.788
27	0.965	0.798	0.817	0.858	0.788
28	0.971	0.806	0.817	0.863	0.795
29	0.974	0.824	0.802	0.866	0.799
30	0.965	0.811	0.799	0.858	0.786
31	0.965	0.806	0.826	0.864	0.797
32	0.965	0.817	0.805	0.861	0.792
33	0.962	0.806	0.814	0.859	0.790
34	0.965	0.783	0.805	0.849	0.775

35	0.968	0.775	0.799	0.845	0.769
36	0.977	0.775	0.793	0.846	0.770
37	0.974	0.791	0.790	0.850	0.775
38	0.971	0.786	0.787	0.846	0.770
39	0.974	0.786	0.787	0.847	0.771
40	0.971	0.775	0.793	0.844	0.767
41	0.968	0.778	0.778	0.840	0.760
42	0.977	0.783	0.793	0.849	0.774
43	0.965	0.780	0.811	0.850	0.776
44	0.974	0.814	0.775	0.854	0.780
45	0.968	0.806	0.787	0.853	0.779
46	0.980	0.798	0.817	0.863	0.795
47	0.957	0.824	0.790	0.857	0.785
48	0.971	0.832	0.790	0.864	0.796
49	0.977	0.827	0.781	0.861	0.792
50	0.971	0.819	0.799	0.862	0.793
51	0.977	0.814	0.799	0.862	0.794
52	0.968	0.829	0.793	0.863	0.795
53	0.965	0.824	0.787	0.858	0.788
54	0.980	0.829	0.790	0.866	0.799
55	0.962	0.829	0.799	0.863	0.795
56	0.962	0.824	0.790	0.858	0.788
57	0.965	0.824	0.796	0.861	0.792
58	0.960	0.832	0.790	0.860	0.790
59	0.971	0.824	0.787	0.860	0.790
60	0.965	0.824	0.802	0.863	0.795
61	0.974	0.814	0.784	0.857	0.785
62	0.965	0.817	0.811	0.863	0.795
63	0.960	0.804	0.784	0.848	0.772
64	0.968	0.801	0.778	0.848	0.772
65	0.968	0.796	0.784	0.848	0.772
66	0.968	0.796	0.781	0.847	0.771
67	0.965	0.791	0.793	0.848	0.773
68	0.968	0.801	0.775	0.847	0.771
69	0.977	0.796	0.778	0.849	0.774
70	0.974	0.793	0.778	0.847	0.771
71	0.974	0.793	0.781	0.848	0.772
72	0.965	0.798	0.790	0.850	0.775
73	0.968	0.783	0.778	0.842	0.763
74	0.974	0.801	0.757	0.843	0.765
75	0.971	0.798	0.772	0.846	0.769
76	0.971	0.798	0.811	0.858	0.788

77	0.971	0.793	0.814	0.858	0.787
78	0.971	0.798	0.796	0.854	0.781
79	0.977	0.791	0.790	0.851	0.777
80	0.971	0.788	0.796	0.850	0.776
81	0.974	0.791	0.799	0.853	0.780
82	0.974	0.804	0.799	0.858	0.786
83	0.968	0.780	0.802	0.848	0.773
84	0.974	0.804	0.796	0.857	0.785
85	0.971	0.791	0.805	0.854	0.781
86	0.974	0.783	0.826	0.858	0.789
87	0.971	0.791	0.799	0.852	0.778
88	0.968	0.749	0.808	0.839	0.760
89	0.971	0.755	0.802	0.840	0.761
90	0.965	0.762	0.799	0.840	0.761
91	0.971	0.752	0.814	0.843	0.766
92	0.968	0.739	0.817	0.838	0.759
93	0.962	0.752	0.814	0.840	0.761
94	0.974	0.757	0.811	0.844	0.768
95	0.974	0.729	0.796	0.829	0.746
96	0.980	0.755	0.784	0.837	0.756
97	0.968	0.747	0.802	0.836	0.756
98	0.971	0.739	0.799	0.833	0.752
99	0.968	0.757	0.796	0.838	0.758
100	0.971	0.744	0.808	0.838	0.759
101	0.971	0.747	0.805	0.838	0.759
102	0.968	0.734	0.811	0.834	0.754
103	0.962	0.755	0.784	0.831	0.748
104	0.968	0.736	0.796	0.830	0.748
105	0.974	0.757	0.787	0.837	0.756
106	0.968	0.742	0.802	0.834	0.753
107	0.974	0.736	0.808	0.836	0.756
108	0.974	0.749	0.802	0.839	0.760
109	0.974	0.749	0.796	0.837	0.757
110	0.974	0.747	0.793	0.835	0.754
111	0.971	0.752	0.799	0.838	0.758
112	0.965	0.760	0.811	0.843	0.765
113	0.960	0.734	0.817	0.833	0.753
114	0.977	0.734	0.805	0.835	0.755
115	0.983	0.752	0.799	0.842	0.764
116	0.968	0.755	0.802	0.839	0.760
117	0.965	0.757	0.784	0.833	0.751
118	0.965	0.755	0.805	0.839	0.760

119	0.971	0.736	0.817	0.838	0.759
120	0.971	0.739	0.802	0.834	0.753
121	0.968	0.757	0.799	0.839	0.760
122	0.968	0.752	0.790	0.834	0.752
123	0.974	0.739	0.811	0.838	0.759
124	0.962	0.757	0.802	0.838	0.758
125	0.951	0.775	0.808	0.843	0.765
126	0.962	0.760	0.823	0.845	0.770
127	0.960	0.765	0.811	0.843	0.765
128	0.962	0.767	0.808	0.843	0.766
129	0.962	0.775	0.814	0.848	0.773
130	0.962	0.767	0.796	0.840	0.760
131	0.957	0.762	0.829	0.846	0.772
132	0.962	0.736	0.820	0.836	0.757
133	0.968	0.760	0.829	0.849	0.776
134	0.957	0.770	0.802	0.841	0.762
135	0.960	0.775	0.805	0.844	0.768
136	0.960	0.770	0.790	0.838	0.758
137	0.974	0.778	0.829	0.858	0.788
138	0.965	0.760	0.829	0.848	0.774
139	0.954	0.778	0.808	0.844	0.768
140	0.971	0.739	0.826	0.842	0.765
141	0.962	0.747	0.814	0.838	0.759
142	0.951	0.742	0.832	0.838	0.760
143	0.962	0.762	0.814	0.843	0.767
144	0.960	0.775	0.820	0.849	0.775
145	0.957	0.744	0.835	0.842	0.766
146	0.962	0.775	0.802	0.844	0.768
147	0.960	0.775	0.817	0.848	0.774
148	0.951	0.773	0.814	0.843	0.767
149	0.960	0.757	0.817	0.842	0.764
150	0.957	0.778	0.790	0.840	0.760
151	0.968	0.757	0.826	0.847	0.773
152	0.962	0.731	0.847	0.843	0.768
153	0.957	0.760	0.817	0.842	0.764
154	0.962	0.770	0.799	0.842	0.763
155	0.962	0.780	0.805	0.847	0.772
156	0.957	0.752	0.802	0.834	0.753
157	0.954	0.770	0.805	0.841	0.762
158	0.962	0.775	0.814	0.848	0.773
159	0.960	0.767	0.823	0.847	0.773
160	0.960	0.773	0.817	0.847	0.772

161	0.960	0.786	0.793	0.844	0.767
162	0.962	0.765	0.811	0.843	0.767
163	0.957	0.780	0.823	0.851	0.778
164	0.962	0.770	0.817	0.847	0.772
165	0.960	0.765	0.811	0.843	0.765
166	0.965	0.762	0.814	0.844	0.768
167	0.960	0.783	0.805	0.847	0.772
168	0.957	0.780	0.811	0.847	0.772
169	0.960	0.765	0.799	0.839	0.759
170	0.960	0.762	0.796	0.837	0.757
171	0.965	0.760	0.796	0.838	0.758
172	0.954	0.755	0.817	0.839	0.760
173	0.960	0.770	0.820	0.847	0.773
174	0.962	0.778	0.793	0.843	0.764
175	0.954	0.773	0.811	0.843	0.766
176	0.954	0.749	0.808	0.834	0.753
177	0.957	0.762	0.817	0.843	0.766
178	0.960	0.755	0.808	0.838	0.759
179	0.948	0.762	0.814	0.839	0.760
180	0.960	0.773	0.796	0.841	0.762
181	0.962	0.770	0.793	0.840	0.760
182	0.951	0.783	0.811	0.846	0.770
183	0.960	0.770	0.814	0.845	0.769
184	0.957	0.783	0.841	0.858	0.788
185	0.960	0.780	0.832	0.855	0.784
186	0.960	0.770	0.823	0.848	0.774
187	0.960	0.775	0.820	0.849	0.775
188	0.960	0.770	0.826	0.849	0.775
189	0.957	0.786	0.799	0.845	0.769
190	0.960	0.775	0.817	0.848	0.774
191	0.960	0.773	0.814	0.846	0.771
192	0.960	0.775	0.841	0.856	0.785
193	0.957	0.775	0.826	0.850	0.777
194	0.957	0.783	0.811	0.848	0.773
195	0.962	0.762	0.826	0.847	0.773
196	0.954	0.778	0.808	0.844	0.768
197	0.957	0.767	0.805	0.841	0.762
198	0.962	0.744	0.832	0.843	0.767
199	0.954	0.765	0.796	0.836	0.755
200	0.965	0.724	0.796	0.825	0.740
201	0.968	0.742	0.766	0.823	0.735
202	0.957	0.739	0.790	0.826	0.740

203	0.965	0.755	0.790	0.834	0.752
204	0.962	0.744	0.784	0.828	0.743
205	0.965	0.734	0.787	0.826	0.740
206	0.962	0.744	0.796	0.831	0.749
207	0.968	0.744	0.772	0.826	0.739
208	0.965	0.765	0.778	0.834	0.752
209	0.974	0.747	0.778	0.830	0.747
210	0.971	0.736	0.775	0.825	0.738
211	0.971	0.762	0.775	0.834	0.752
212	0.965	0.749	0.790	0.832	0.750
213	0.971	0.744	0.763	0.824	0.736
214	0.971	0.718	0.805	0.828	0.744
215	0.977	0.736	0.778	0.828	0.743
216	0.965	0.744	0.778	0.827	0.741
217	0.974	0.744	0.766	0.826	0.739
218	0.971	0.747	0.787	0.832	0.750
219	0.974	0.757	0.769	0.831	0.747
220	0.968	0.726	0.778	0.821	0.733
221	0.960	0.734	0.772	0.819	0.730
222	0.965	0.742	0.775	0.825	0.738
223	0.968	0.716	0.784	0.819	0.731
224	0.962	0.747	0.793	0.831	0.748
225	0.965	0.747	0.793	0.832	0.750
226	0.968	0.736	0.760	0.819	0.729
227	0.968	0.744	0.781	0.828	0.744
228	0.965	0.744	0.787	0.829	0.745
229	0.960	0.721	0.793	0.821	0.734
230	0.962	0.729	0.811	0.830	0.748
231	0.957	0.736	0.793	0.826	0.740
232	0.968	0.739	0.778	0.826	0.740
233	0.962	0.739	0.787	0.827	0.741
234	0.962	0.736	0.805	0.831	0.749
235	0.965	0.734	0.814	0.834	0.754
236	0.968	0.744	0.784	0.829	0.746
237	0.960	0.734	0.802	0.828	0.745
238	0.954	0.724	0.796	0.821	0.734
239	0.951	0.742	0.808	0.830	0.748
240	0.945	0.731	0.814	0.827	0.743
241	0.954	0.742	0.790	0.826	0.740
242	0.960	0.734	0.826	0.836	0.757
243	0.957	0.734	0.802	0.828	0.744
244	0.954	0.749	0.784	0.827	0.741

245	0.957	0.726	0.814	0.828	0.746
246	0.962	0.739	0.787	0.827	0.742
247	0.957	0.744	0.796	0.829	0.746
248	0.960	0.716	0.808	0.824	0.739
249	0.960	0.742	0.817	0.836	0.756
250	0.965	0.729	0.793	0.826	0.741
251	0.954	0.755	0.793	0.831	0.748
252	0.954	0.726	0.808	0.826	0.741
253	0.962	0.736	0.805	0.831	0.749
254	0.954	0.724	0.817	0.828	0.745
255	0.965	0.716	0.805	0.825	0.741
256	0.957	0.736	0.793	0.826	0.740
257	0.962	0.742	0.814	0.836	0.756
258	0.954	0.713	0.817	0.824	0.740
259	0.951	0.739	0.802	0.828	0.744
260	0.957	0.744	0.796	0.829	0.746
261	0.960	0.729	0.790	0.823	0.736
262	0.954	0.734	0.793	0.824	0.738
263	0.965	0.718	0.817	0.829	0.748
264	0.965	0.729	0.787	0.824	0.738
265	0.954	0.742	0.799	0.828	0.745
266	0.960	0.721	0.781	0.817	0.728
267	0.965	0.726	0.802	0.828	0.744
268	0.962	0.731	0.796	0.827	0.742
269	0.954	0.731	0.802	0.826	0.741
270	0.968	0.742	0.799	0.833	0.751
271	0.968	0.736	0.811	0.835	0.755
272	0.962	0.739	0.796	0.829	0.746
273	0.960	0.744	0.793	0.829	0.746
274	0.960	0.736	0.784	0.824	0.737
275	0.965	0.729	0.784	0.823	0.736
276	0.965	0.739	0.796	0.830	0.747
277	0.968	0.731	0.805	0.831	0.749
278	0.965	0.716	0.790	0.820	0.732
279	0.965	0.718	0.814	0.828	0.746
280	0.962	0.734	0.775	0.821	0.733
281	0.962	0.700	0.805	0.818	0.731
282	0.965	0.718	0.796	0.823	0.737
283	0.960	0.729	0.802	0.827	0.742
284	0.957	0.724	0.802	0.824	0.738
285	0.960	0.721	0.817	0.828	0.746
286	0.954	0.721	0.817	0.827	0.743

287	0.962	0.729	0.823	0.834	0.754
288	0.971	0.718	0.811	0.829	0.747
289	0.962	0.705	0.817	0.824	0.740
290	0.960	0.731	0.799	0.827	0.742
291	0.962	0.731	0.799	0.828	0.744
292	0.960	0.729	0.805	0.828	0.744
293	0.962	0.698	0.796	0.814	0.725
294	0.960	0.711	0.817	0.825	0.741
295	0.968	0.726	0.826	0.836	0.757
296	0.960	0.731	0.793	0.825	0.739
297	0.951	0.724	0.808	0.824	0.739
298	0.962	0.721	0.808	0.827	0.743
299	0.957	0.726	0.808	0.827	0.743
300	0.965	0.724	0.796	0.825	0.740
301	0.951	0.742	0.808	0.830	0.748
302	0.971	0.726	0.826	0.837	0.759
303	0.962	0.724	0.820	0.831	0.750
304	0.948	0.726	0.790	0.818	0.729
305	0.965	0.731	0.796	0.828	0.743
306	0.957	0.718	0.790	0.818	0.729
307	0.971	0.724	0.802	0.828	0.745
308	0.965	0.729	0.808	0.830	0.748
309	0.954	0.731	0.805	0.827	0.742
310	0.965	0.718	0.817	0.829	0.748
311	0.960	0.726	0.796	0.824	0.738
312	0.957	0.739	0.787	0.825	0.739
313	0.939	0.721	0.814	0.821	0.735
314	0.936	0.734	0.784	0.815	0.725
315	0.936	0.729	0.805	0.820	0.733
316	0.954	0.742	0.766	0.818	0.728
317	0.957	0.731	0.814	0.830	0.748
318	0.951	0.726	0.826	0.830	0.749
319	0.954	0.747	0.805	0.832	0.750
320	0.951	0.742	0.832	0.838	0.760
321	0.945	0.744	0.802	0.828	0.743
322	0.954	0.721	0.811	0.825	0.740
323	0.954	0.734	0.817	0.831	0.750
324	0.951	0.739	0.805	0.828	0.745
325	0.960	0.700	0.784	0.811	0.719
326	0.965	0.742	0.793	0.830	0.747
327	0.951	0.713	0.820	0.824	0.739
328	0.960	0.736	0.799	0.828	0.745

329	0.957	0.747	0.814	0.836	0.756
330	0.960	0.734	0.793	0.826	0.740
331	0.957	0.729	0.793	0.823	0.736
332	0.957	0.731	0.802	0.827	0.742
333	0.957	0.755	0.796	0.833	0.751
334	0.962	0.734	0.805	0.830	0.748
335	0.957	0.729	0.802	0.826	0.741
336	0.962	0.755	0.802	0.837	0.757
337	0.954	0.721	0.796	0.820	0.733
338	0.971	0.718	0.796	0.825	0.740
339	0.962	0.734	0.769	0.819	0.730
340	0.965	0.742	0.820	0.839	0.761
341	0.951	0.731	0.817	0.829	0.747
342	0.954	0.747	0.781	0.825	0.738
343	0.957	0.736	0.814	0.832	0.751
344	0.954	0.734	0.820	0.832	0.751
345	0.960	0.721	0.811	0.827	0.743
346	0.951	0.734	0.808	0.828	0.744
347	0.957	0.718	0.817	0.827	0.743
348	0.948	0.721	0.817	0.825	0.740
349	0.954	0.742	0.802	0.829	0.746
350	0.948	0.744	0.796	0.827	0.741
351	0.957	0.724	0.805	0.825	0.740
352	0.965	0.721	0.802	0.826	0.741
353	0.948	0.742	0.808	0.829	0.746
354	0.951	0.752	0.793	0.829	0.745
355	0.954	0.726	0.811	0.827	0.743
356	0.957	0.731	0.805	0.828	0.744
357	0.962	0.739	0.799	0.830	0.747
358	0.960	0.716	0.796	0.820	0.733
359	0.968	0.736	0.805	0.833	0.752
360	0.951	0.736	0.805	0.828	0.743
361	0.957	0.736	0.817	0.833	0.752
362	0.945	0.724	0.802	0.820	0.733
363	0.942	0.742	0.817	0.830	0.748
364	0.957	0.729	0.814	0.829	0.747
365	0.957	0.739	0.823	0.836	0.757
366	0.962	0.731	0.817	0.833	0.752
367	0.951	0.747	0.802	0.830	0.747
368	0.957	0.736	0.817	0.833	0.752
369	0.962	0.731	0.784	0.823	0.736
370	0.945	0.739	0.784	0.820	0.731

371	0.936	0.747	0.787	0.821	0.733
372	0.948	0.749	0.820	0.836	0.756
373	0.939	0.736	0.808	0.825	0.739
374	0.945	0.757	0.799	0.831	0.748
375	0.939	0.752	0.778	0.821	0.732
376	0.951	0.742	0.808	0.830	0.747
377	0.948	0.760	0.799	0.833	0.751
378	0.951	0.739	0.769	0.817	0.727
379	0.957	0.744	0.796	0.829	0.745
380	0.954	0.744	0.787	0.826	0.740
381	0.951	0.734	0.787	0.821	0.733
382	0.945	0.739	0.787	0.821	0.733
383	0.942	0.744	0.802	0.827	0.742
384	0.939	0.739	0.799	0.823	0.736
385	0.945	0.724	0.802	0.820	0.733
386	0.954	0.747	0.757	0.817	0.726
387	0.934	0.749	0.787	0.821	0.733
388	0.948	0.726	0.823	0.828	0.746
389	0.948	0.736	0.823	0.832	0.751
390	0.942	0.734	0.790	0.819	0.730
391	0.948	0.739	0.790	0.823	0.736
392	0.942	0.749	0.772	0.819	0.729
393	0.942	0.747	0.790	0.824	0.737
394	0.948	0.752	0.802	0.831	0.748
395	0.945	0.726	0.793	0.818	0.729
396	0.954	0.749	0.793	0.829	0.745
397	0.951	0.734	0.823	0.832	0.751
398	0.951	0.742	0.781	0.822	0.734
399	0.954	0.736	0.790	0.824	0.737
400	0.962	0.698	0.799	0.815	0.727
401	0.960	0.729	0.796	0.825	0.739
402	0.962	0.734	0.781	0.823	0.736
403	0.962	0.747	0.787	0.829	0.745
404	0.939	0.734	0.796	0.820	0.732
405	0.951	0.724	0.781	0.815	0.725
406	0.960	0.711	0.799	0.819	0.731
407	0.960	0.747	0.772	0.824	0.736
408	0.951	0.749	0.772	0.822	0.734
409	0.957	0.713	0.760	0.807	0.712
410	0.962	0.734	0.796	0.828	0.743
411	0.960	0.736	0.754	0.814	0.722
412	0.957	0.703	0.778	0.809	0.716

413	0.951	0.721	0.766	0.810	0.716
414	0.968	0.718	0.772	0.816	0.726
415	0.951	0.721	0.772	0.812	0.719
416	0.948	0.718	0.769	0.809	0.715
417	0.965	0.705	0.784	0.814	0.724
418	0.954	0.736	0.799	0.827	0.742
419	0.954	0.708	0.793	0.814	0.725
420	0.957	0.731	0.754	0.812	0.718
421	0.954	0.739	0.757	0.814	0.722
422	0.954	0.726	0.799	0.823	0.737
423	0.957	0.711	0.790	0.815	0.726
424	0.960	0.729	0.769	0.816	0.726
425	0.960	0.724	0.775	0.816	0.726
426	0.960	0.703	0.787	0.813	0.722
427	0.954	0.713	0.778	0.812	0.720
428	0.960	0.736	0.766	0.818	0.728
429	0.945	0.731	0.778	0.815	0.725
430	0.942	0.744	0.757	0.813	0.719
431	0.951	0.716	0.793	0.816	0.727
432	0.951	0.731	0.778	0.817	0.727
433	0.948	0.726	0.787	0.817	0.728
434	0.957	0.721	0.778	0.815	0.725
435	0.960	0.705	0.799	0.817	0.729
436	0.957	0.726	0.775	0.816	0.726
437	0.957	0.713	0.787	0.815	0.725
438	0.957	0.716	0.778	0.813	0.722
439	0.962	0.724	0.787	0.821	0.734
440	0.957	0.721	0.772	0.813	0.722
441	0.945	0.716	0.796	0.815	0.726
442	0.939	0.718	0.799	0.815	0.726
443	0.936	0.724	0.766	0.806	0.710
444	0.957	0.708	0.743	0.799	0.700
445	0.962	0.739	0.781	0.825	0.739
446	0.960	0.729	0.790	0.823	0.737
447	0.960	0.726	0.763	0.813	0.722
448	0.960	0.726	0.784	0.820	0.732
449	0.939	0.736	0.781	0.816	0.726
450	0.957	0.708	0.784	0.813	0.722
451	0.954	0.716	0.775	0.812	0.719
452	0.957	0.726	0.817	0.829	0.747
453	0.962	0.724	0.784	0.820	0.732
454	0.951	0.700	0.802	0.813	0.724

455	0.951	0.711	0.778	0.810	0.717
456	0.945	0.739	0.802	0.826	0.740
457	0.942	0.708	0.790	0.810	0.717
458	0.948	0.739	0.790	0.823	0.736
459	0.954	0.739	0.763	0.816	0.725
460	0.957	0.747	0.790	0.828	0.744
461	0.936	0.744	0.754	0.810	0.715
462	0.962	0.755	0.772	0.828	0.742
463	0.954	0.731	0.769	0.815	0.724
464	0.948	0.747	0.787	0.825	0.738
465	0.925	0.752	0.790	0.820	0.731
466	0.934	0.729	0.787	0.813	0.722
467	0.934	0.755	0.772	0.818	0.728
468	0.910	0.718	0.790	0.803	0.707
469	0.916	0.700	0.790	0.799	0.701
470	0.934	0.752	0.796	0.825	0.738
471	0.919	0.739	0.778	0.810	0.716
472	0.936	0.757	0.799	0.828	0.744
473	0.936	0.713	0.766	0.802	0.705
474	0.931	0.744	0.778	0.815	0.724
475	0.934	0.729	0.802	0.818	0.729
476	0.948	0.739	0.778	0.819	0.730
477	0.951	0.757	0.760	0.821	0.732
478	0.922	0.752	0.781	0.816	0.725
479	0.939	0.752	0.793	0.826	0.740
480	0.934	0.726	0.781	0.811	0.718
481	0.928	0.739	0.787	0.815	0.724
482	0.939	0.739	0.775	0.815	0.724
483	0.928	0.742	0.787	0.816	0.726
484	0.922	0.760	0.784	0.820	0.731
485	0.931	0.721	0.784	0.809	0.715
486	0.939	0.760	0.781	0.825	0.738
487	0.934	0.724	0.823	0.823	0.738
488	0.936	0.744	0.775	0.816	0.725
489	0.928	0.747	0.781	0.816	0.726
490	0.936	0.757	0.763	0.817	0.726
491	0.931	0.755	0.775	0.818	0.728
492	0.945	0.747	0.796	0.827	0.741
493	0.925	0.742	0.772	0.811	0.717
494	0.939	0.760	0.775	0.823	0.735
495	0.913	0.752	0.769	0.810	0.715
496	0.922	0.755	0.778	0.816	0.725

497	0.934	0.749	0.772	0.816	0.725
498	0.942	0.713	0.784	0.810	0.717
499	0.945	0.698	0.799	0.810	0.718
500	0.934	0.713	0.772	0.803	0.707
501	0.942	0.747	0.799	0.827	0.741
502	0.942	0.711	0.790	0.811	0.718
503	0.942	0.747	0.784	0.822	0.734
504	0.942	0.744	0.784	0.821	0.732
505	0.936	0.749	0.772	0.817	0.727
506	0.939	0.752	0.778	0.821	0.732
507	0.951	0.731	0.769	0.814	0.723
508	0.957	0.752	0.793	0.831	0.748
509	0.939	0.736	0.793	0.820	0.732
510	0.925	0.705	0.802	0.807	0.714
511	0.934	0.721	0.808	0.817	0.729
512	0.945	0.757	0.796	0.830	0.747
513	0.939	0.736	0.802	0.823	0.736
514	0.928	0.729	0.778	0.809	0.715
515	0.939	0.726	0.808	0.821	0.734
516	0.939	0.729	0.799	0.819	0.731
517	0.936	0.752	0.793	0.825	0.738
518	0.945	0.736	0.802	0.825	0.739
519	0.922	0.739	0.808	0.820	0.732
520	0.948	0.749	0.802	0.830	0.747
521	0.942	0.731	0.790	0.818	0.729
522	0.942	0.744	0.790	0.823	0.736
523	0.954	0.721	0.793	0.819	0.731
524	0.925	0.721	0.793	0.810	0.717
525	0.945	0.726	0.790	0.817	0.728
526	0.934	0.742	0.787	0.818	0.729
527	0.936	0.729	0.796	0.817	0.728
528	0.919	0.742	0.802	0.818	0.729
529	0.931	0.713	0.790	0.808	0.714
530	0.922	0.752	0.793	0.820	0.731
531	0.910	0.747	0.790	0.813	0.722
532	0.916	0.718	0.766	0.798	0.698
533	0.931	0.705	0.793	0.806	0.712
534	0.931	0.739	0.787	0.816	0.726
535	0.916	0.718	0.799	0.808	0.714
536	0.919	0.742	0.769	0.808	0.713
537	0.931	0.731	0.775	0.810	0.716
538	0.919	0.739	0.751	0.801	0.703

539	0.916	0.711	0.799	0.805	0.711
540	0.928	0.718	0.787	0.808	0.714
541	0.922	0.742	0.775	0.811	0.717
542	0.925	0.736	0.799	0.817	0.728
543	0.922	0.731	0.793	0.813	0.721
544	0.910	0.713	0.784	0.799	0.702
545	0.916	0.693	0.775	0.791	0.690
546	0.913	0.726	0.778	0.803	0.707
547	0.931	0.734	0.746	0.801	0.702
548	0.910	0.690	0.796	0.795	0.696
549	0.936	0.724	0.772	0.808	0.713
550	0.919	0.708	0.787	0.801	0.705
551	0.931	0.757	0.772	0.818	0.728
552	0.925	0.731	0.784	0.811	0.718
553	0.910	0.705	0.802	0.802	0.707
554	0.925	0.713	0.784	0.804	0.709
555	0.919	0.731	0.781	0.808	0.714
556	0.908	0.721	0.778	0.799	0.701
557	0.922	0.747	0.760	0.808	0.713
558	0.919	0.744	0.737	0.799	0.698
559	0.919	0.736	0.760	0.803	0.706
560	0.916	0.762	0.754	0.810	0.715
561	0.925	0.695	0.778	0.796	0.697
562	0.925	0.739	0.740	0.799	0.700
563	0.928	0.744	0.751	0.806	0.710
564	0.936	0.726	0.781	0.812	0.719
565	0.908	0.739	0.784	0.808	0.714
566	0.925	0.739	0.775	0.811	0.717
567	0.922	0.752	0.760	0.810	0.715
568	0.931	0.755	0.787	0.822	0.734
569	0.908	0.726	0.766	0.798	0.698
570	0.925	0.734	0.769	0.807	0.712
571	0.916	0.711	0.766	0.795	0.695
572	0.934	0.739	0.757	0.808	0.713
573	0.928	0.734	0.751	0.802	0.704
574	0.893	0.705	0.772	0.787	0.684
575	0.925	0.734	0.757	0.803	0.706
576	0.925	0.721	0.737	0.792	0.689
577	0.919	0.739	0.781	0.811	0.718
578	0.922	0.708	0.778	0.799	0.702
579	0.931	0.705	0.781	0.802	0.706
580	0.936	0.739	0.760	0.810	0.715

581	0.931	0.736	0.734	0.799	0.698
582	0.913	0.726	0.772	0.801	0.704
583	0.913	0.721	0.766	0.798	0.698
584	0.928	0.713	0.775	0.802	0.705
585	0.919	0.739	0.763	0.805	0.709
586	0.913	0.703	0.793	0.799	0.703
587	0.922	0.726	0.784	0.808	0.714
588	0.919	0.747	0.772	0.811	0.717
589	0.942	0.731	0.781	0.815	0.725
590	0.939	0.718	0.781	0.810	0.717
591	0.919	0.747	0.746	0.802	0.704
592	0.916	0.708	0.763	0.793	0.691
593	0.916	0.744	0.749	0.801	0.703
594	0.925	0.724	0.754	0.799	0.699
595	0.913	0.708	0.778	0.797	0.698
596	0.913	0.744	0.772	0.808	0.713
597	0.919	0.716	0.757	0.795	0.694
598	0.928	0.711	0.775	0.801	0.704
599	0.922	0.742	0.766	0.808	0.713
600	0.925	0.734	0.757	0.803	0.706
601	0.925	0.736	0.760	0.805	0.708
602	0.913	0.721	0.781	0.802	0.706
603	0.922	0.734	0.760	0.803	0.706
604	0.908	0.729	0.769	0.799	0.701
605	0.916	0.739	0.769	0.806	0.710
606	0.928	0.700	0.766	0.795	0.695
607	0.919	0.729	0.746	0.796	0.694
608	0.939	0.736	0.749	0.806	0.709
609	0.925	0.729	0.772	0.806	0.711
610	0.913	0.718	0.754	0.793	0.691
611	0.913	0.718	0.760	0.795	0.694
612	0.925	0.721	0.766	0.801	0.704
613	0.925	0.729	0.766	0.804	0.707
614	0.945	0.734	0.766	0.813	0.720
615	0.908	0.729	0.769	0.799	0.701
616	0.908	0.742	0.751	0.799	0.699
617	0.919	0.718	0.772	0.800	0.703
618	0.934	0.716	0.763	0.801	0.704
619	0.925	0.739	0.751	0.803	0.706
620	0.928	0.700	0.766	0.795	0.695
621	0.925	0.744	0.775	0.813	0.720
622	0.925	0.718	0.749	0.795	0.693

623	0.919	0.726	0.760	0.799	0.701
624	0.931	0.724	0.763	0.803	0.706
625	0.910	0.726	0.757	0.796	0.695
626	0.925	0.721	0.746	0.795	0.693
627	0.910	0.729	0.763	0.799	0.699
628	0.922	0.736	0.772	0.808	0.713
629	0.922	0.742	0.757	0.805	0.708
630	0.925	0.724	0.746	0.796	0.695
631	0.919	0.731	0.749	0.798	0.697
632	0.922	0.742	0.757	0.805	0.708
633	0.931	0.693	0.778	0.797	0.698
634	0.928	0.736	0.746	0.801	0.702
635	0.945	0.726	0.775	0.813	0.721
636	0.916	0.729	0.766	0.801	0.704
637	0.934	0.718	0.775	0.806	0.711
638	0.928	0.726	0.754	0.800	0.701
639	0.916	0.736	0.766	0.804	0.707
640	0.922	0.713	0.772	0.799	0.701
641	0.910	0.721	0.754	0.793	0.691
642	0.922	0.747	0.760	0.808	0.712
643	0.910	0.716	0.787	0.801	0.705
644	0.905	0.705	0.769	0.790	0.688
645	0.922	0.731	0.749	0.799	0.699
646	0.931	0.700	0.769	0.797	0.697
647	0.916	0.731	0.766	0.802	0.705
648	0.931	0.747	0.751	0.808	0.712
649	0.931	0.716	0.772	0.803	0.707
650	0.925	0.726	0.760	0.801	0.703
651	0.925	0.729	0.737	0.795	0.693
652	0.913	0.744	0.757	0.803	0.706
653	0.919	0.713	0.772	0.799	0.700
654	0.928	0.726	0.769	0.805	0.709
655	0.934	0.734	0.746	0.802	0.704
656	0.942	0.729	0.763	0.809	0.714
657	0.908	0.724	0.769	0.798	0.698
658	0.942	0.711	0.775	0.806	0.711
659	0.916	0.731	0.781	0.807	0.712
660	0.919	0.731	0.775	0.806	0.711
661	0.908	0.703	0.775	0.792	0.691
662	0.922	0.747	0.775	0.813	0.720
663	0.936	0.729	0.754	0.804	0.707
664	0.925	0.708	0.749	0.791	0.688

665	0.919	0.724	0.760	0.799	0.699
666	0.934	0.718	0.769	0.804	0.708
667	0.899	0.742	0.746	0.794	0.692
668	0.931	0.739	0.769	0.811	0.717
669	0.928	0.734	0.754	0.803	0.706
670	0.936	0.734	0.751	0.805	0.708
671	0.916	0.742	0.757	0.803	0.705
672	0.925	0.742	0.763	0.808	0.713
673	0.925	0.718	0.784	0.806	0.711
674	0.925	0.744	0.778	0.813	0.721
675	0.931	0.731	0.754	0.803	0.706
676	0.908	0.716	0.763	0.793	0.691
677	0.913	0.729	0.757	0.798	0.698
678	0.925	0.708	0.772	0.799	0.700
679	0.928	0.734	0.757	0.804	0.707
680	0.931	0.742	0.763	0.810	0.715
681	0.936	0.721	0.772	0.807	0.712
682	0.922	0.721	0.775	0.803	0.707
683	0.934	0.726	0.763	0.805	0.709
684	0.919	0.742	0.760	0.805	0.708
685	0.934	0.752	0.769	0.816	0.725
686	0.925	0.739	0.737	0.799	0.698
687	0.936	0.729	0.763	0.807	0.712
688	0.925	0.716	0.751	0.795	0.694
689	0.919	0.729	0.740	0.794	0.692
690	0.934	0.731	0.763	0.807	0.712
691	0.936	0.703	0.751	0.794	0.692
692	0.922	0.742	0.766	0.808	0.713
693	0.942	0.729	0.772	0.812	0.719
694	0.925	0.729	0.763	0.803	0.706
695	0.928	0.739	0.760	0.807	0.711
696	0.931	0.749	0.746	0.807	0.711
697	0.928	0.752	0.719	0.799	0.697
698	0.934	0.724	0.778	0.809	0.715
699	0.913	0.721	0.754	0.794	0.692
700	0.913	0.736	0.778	0.807	0.712
701	0.916	0.734	0.781	0.808	0.714
702	0.908	0.757	0.743	0.801	0.702
703	0.928	0.731	0.772	0.808	0.713
704	0.936	0.718	0.772	0.806	0.711
705	0.928	0.749	0.743	0.805	0.708
706	0.910	0.736	0.760	0.800	0.702

707	0.919	0.718	0.760	0.797	0.697
708	0.925	0.716	0.763	0.799	0.699
709	0.925	0.724	0.751	0.798	0.698
710	0.919	0.729	0.760	0.800	0.702
711	0.922	0.734	0.740	0.797	0.696
712	0.928	0.729	0.769	0.806	0.711
713	0.916	0.726	0.763	0.799	0.701
714	0.928	0.729	0.772	0.807	0.712
715	0.925	0.731	0.760	0.803	0.706
716	0.931	0.726	0.757	0.802	0.705
717	0.931	0.713	0.760	0.799	0.699
718	0.945	0.739	0.749	0.809	0.714
719	0.910	0.721	0.754	0.793	0.691
720	0.945	0.752	0.746	0.813	0.719
721	0.931	0.747	0.749	0.807	0.711
722	0.919	0.713	0.757	0.794	0.692
723	0.913	0.747	0.760	0.805	0.709
724	0.910	0.736	0.757	0.799	0.700
725	0.931	0.742	0.772	0.813	0.720
726	0.916	0.726	0.751	0.796	0.695
727	0.922	0.711	0.763	0.796	0.695
728	0.928	0.729	0.775	0.808	0.713
729	0.913	0.739	0.766	0.804	0.707
730	0.908	0.716	0.740	0.785	0.679
731	0.931	0.703	0.760	0.795	0.694
732	0.919	0.739	0.749	0.800	0.701
733	0.913	0.726	0.766	0.799	0.701
734	0.925	0.711	0.763	0.797	0.697
735	0.919	0.703	0.751	0.788	0.684
736	0.913	0.726	0.749	0.794	0.692
737	0.928	0.724	0.743	0.796	0.694
738	0.902	0.739	0.763	0.799	0.700
739	0.928	0.744	0.743	0.803	0.705
740	0.913	0.734	0.751	0.798	0.697
741	0.928	0.729	0.751	0.800	0.701
742	0.928	0.726	0.778	0.808	0.714
743	0.928	0.713	0.763	0.799	0.700
744	0.925	0.721	0.766	0.801	0.704
745	0.910	0.729	0.757	0.797	0.696
746	0.925	0.721	0.760	0.799	0.700
747	0.908	0.729	0.763	0.798	0.698
748	0.908	0.713	0.757	0.790	0.687

749	0.908	0.721	0.799	0.806	0.712
750	0.916	0.718	0.766	0.798	0.698
751	0.908	0.713	0.772	0.795	0.694
752	0.902	0.731	0.775	0.800	0.703
753	0.916	0.731	0.746	0.796	0.694
754	0.908	0.724	0.760	0.795	0.694
755	0.908	0.744	0.734	0.794	0.691
756	0.910	0.724	0.784	0.803	0.707
757	0.919	0.744	0.757	0.805	0.708
758	0.908	0.726	0.772	0.799	0.701
759	0.910	0.713	0.772	0.796	0.696
760	0.913	0.708	0.781	0.798	0.699
761	0.919	0.724	0.772	0.802	0.705
762	0.910	0.721	0.766	0.797	0.697
763	0.922	0.703	0.799	0.804	0.710
764	0.916	0.724	0.781	0.804	0.708
765	0.910	0.713	0.763	0.793	0.691
766	0.916	0.721	0.784	0.804	0.709
767	0.905	0.724	0.772	0.798	0.699
768	0.913	0.716	0.763	0.795	0.694
769	0.910	0.742	0.760	0.802	0.704
770	0.919	0.726	0.775	0.804	0.708
771	0.905	0.718	0.766	0.794	0.693
772	0.910	0.700	0.784	0.795	0.696
773	0.910	0.713	0.775	0.797	0.698
774	0.913	0.729	0.781	0.805	0.710
775	0.905	0.713	0.802	0.803	0.709
776	0.908	0.739	0.766	0.802	0.705
777	0.931	0.708	0.778	0.802	0.706
778	0.899	0.731	0.766	0.797	0.697
779	0.931	0.693	0.784	0.799	0.701
780	0.899	0.711	0.778	0.793	0.692
781	0.893	0.726	0.769	0.794	0.693
782	0.905	0.734	0.772	0.801	0.704
783	0.896	0.718	0.781	0.796	0.696
784	0.896	0.713	0.784	0.795	0.695
785	0.916	0.716	0.740	0.788	0.683
786	0.908	0.742	0.766	0.803	0.706
787	0.890	0.729	0.778	0.797	0.697
788	0.922	0.721	0.775	0.803	0.707

(3) Performance of RF on top features yielded by three feature selection methods

**mRMR**

Numbers of features	G1	G2/M	S	ACC	MCC
1	0.908	0.636	0.611	0.716	0.575
2	0.945	0.829	0.722	0.833	0.749
3	0.957	0.817	0.757	0.843	0.765
4	0.954	0.855	0.740	0.851	0.776
5	0.954	0.863	0.746	0.856	0.783
6	0.942	0.858	0.751	0.852	0.777
7	0.948	0.858	0.772	0.860	0.790
8	0.942	0.850	0.757	0.851	0.776
9	0.945	0.842	0.763	0.851	0.776
10	0.951	0.840	0.743	0.845	0.768
11	0.957	0.845	0.766	0.857	0.785
12	0.954	0.848	0.760	0.855	0.782
13	0.951	0.863	0.746	0.855	0.782
14	0.951	0.855	0.757	0.856	0.783
15	0.951	0.860	0.757	0.858	0.786
16	0.951	0.858	0.769	0.860	0.790
17	0.954	0.863	0.760	0.860	0.790
18	0.951	0.858	0.778	0.863	0.794
19	0.948	0.863	0.766	0.860	0.790
20	0.951	0.850	0.772	0.858	0.787
21	0.957	0.866	0.775	0.867	0.800
22	0.945	0.863	0.778	0.863	0.794
23	0.962	0.863	0.787	0.872	0.807
24	0.960	0.868	0.796	0.875	0.813
25	0.960	0.876	0.784	0.874	0.811
26	0.965	0.873	0.784	0.875	0.813
27	0.951	0.884	0.784	0.874	0.811
28	0.960	0.879	0.778	0.873	0.810
29	0.951	0.863	0.784	0.867	0.800
30	0.968	0.866	0.796	0.877	0.816
31	0.962	0.868	0.805	0.879	0.818
32	0.971	0.871	0.799	0.881	0.821
33	0.962	0.876	0.805	0.882	0.823
34	0.957	0.866	0.802	0.875	0.813
35	0.968	0.873	0.814	0.886	0.828
36	0.965	0.881	0.799	0.883	0.824
37	0.965	0.894	0.796	0.887	0.830
38	0.965	0.886	0.787	0.881	0.821
39	0.974	0.881	0.808	0.888	0.832
40	0.962	0.889	0.802	0.886	0.828
41	0.957	0.886	0.802	0.883	0.824

42	0.974	0.881	0.796	0.885	0.827
43	0.965	0.886	0.796	0.884	0.826
44	0.965	0.886	0.790	0.882	0.823
45	0.962	0.881	0.805	0.884	0.825
46	0.968	0.886	0.799	0.886	0.828
47	0.960	0.886	0.811	0.887	0.830
48	0.968	0.876	0.817	0.888	0.831
49	0.957	0.886	0.793	0.880	0.820
50	0.962	0.891	0.802	0.887	0.830
51	0.960	0.879	0.817	0.886	0.828
52	0.965	0.881	0.805	0.885	0.827
53	0.965	0.899	0.811	0.893	0.840
54	0.960	0.889	0.802	0.885	0.827
55	0.960	0.891	0.814	0.889	0.834
56	0.957	0.891	0.814	0.888	0.832
57	0.960	0.894	0.817	0.891	0.837
58	0.960	0.886	0.820	0.889	0.834
59	0.962	0.894	0.823	0.894	0.841
60	0.965	0.904	0.808	0.894	0.841
61	0.957	0.902	0.820	0.894	0.841
62	0.965	0.899	0.823	0.897	0.845
63	0.965	0.881	0.832	0.893	0.839
64	0.965	0.902	0.823	0.898	0.847
65	0.968	0.894	0.832	0.899	0.848
66	0.960	0.886	0.817	0.888	0.832
67	0.971	0.894	0.814	0.894	0.841
68	0.968	0.907	0.817	0.899	0.848
69	0.977	0.897	0.814	0.897	0.845
70	0.971	0.891	0.820	0.895	0.842
71	0.974	0.904	0.817	0.900	0.849
72	0.968	0.899	0.829	0.900	0.849
73	0.965	0.904	0.817	0.897	0.845
74	0.968	0.904	0.823	0.900	0.849
75	0.971	0.889	0.811	0.891	0.837
76	0.962	0.899	0.811	0.892	0.838
77	0.965	0.894	0.805	0.889	0.834
78	0.974	0.891	0.826	0.898	0.847
79	0.971	0.894	0.826	0.898	0.847
80	0.962	0.904	0.823	0.898	0.847
81	0.968	0.897	0.823	0.897	0.845
82	0.968	0.894	0.826	0.897	0.845
83	0.971	0.891	0.817	0.894	0.841

84	0.965	0.891	0.820	0.893	0.839
85	0.962	0.894	0.823	0.894	0.841
86	0.965	0.894	0.826	0.896	0.844
87	0.968	0.902	0.823	0.899	0.848
88	0.974	0.899	0.826	0.901	0.851
89	0.965	0.899	0.829	0.899	0.848
90	0.962	0.889	0.826	0.893	0.839
91	0.974	0.889	0.814	0.893	0.840
92	0.968	0.899	0.832	0.901	0.851
93	0.974	0.897	0.832	0.902	0.852
94	0.971	0.894	0.838	0.902	0.852
95	0.974	0.894	0.808	0.893	0.840
96	0.965	0.904	0.832	0.902	0.852
97	0.977	0.891	0.826	0.899	0.848
98	0.965	0.891	0.823	0.894	0.841
99	0.957	0.897	0.817	0.891	0.837
100	0.962	0.894	0.817	0.892	0.838
101	0.965	0.897	0.823	0.896	0.844
102	0.965	0.891	0.805	0.888	0.833
103	0.968	0.897	0.820	0.896	0.844
104	0.968	0.891	0.832	0.898	0.847
105	0.962	0.891	0.826	0.894	0.841
106	0.965	0.891	0.811	0.890	0.835
107	0.968	0.902	0.832	0.902	0.852
108	0.968	0.889	0.829	0.896	0.844
109	0.962	0.897	0.817	0.893	0.840
110	0.971	0.886	0.823	0.894	0.841
111	0.971	0.891	0.823	0.896	0.844
112	0.962	0.899	0.835	0.900	0.849
113	0.971	0.894	0.826	0.898	0.847
114	0.957	0.894	0.805	0.887	0.830
115	0.968	0.889	0.844	0.901	0.851
116	0.960	0.889	0.814	0.888	0.832
117	0.965	0.873	0.832	0.890	0.835
118	0.968	0.884	0.835	0.896	0.844
119	0.960	0.884	0.820	0.888	0.832
120	0.965	0.904	0.841	0.904	0.856
121	0.968	0.899	0.829	0.900	0.849
122	0.960	0.897	0.832	0.897	0.845
123	0.962	0.889	0.820	0.891	0.837
124	0.974	0.899	0.823	0.900	0.849
125	0.962	0.891	0.823	0.893	0.839

126	0.965	0.897	0.829	0.898	0.847
127	0.965	0.899	0.832	0.900	0.849
128	0.960	0.899	0.832	0.898	0.847
129	0.957	0.889	0.829	0.892	0.838
130	0.960	0.894	0.841	0.899	0.848
131	0.965	0.894	0.829	0.897	0.845
132	0.965	0.889	0.820	0.892	0.838
133	0.968	0.881	0.832	0.894	0.841
134	0.962	0.884	0.817	0.888	0.832
135	0.962	0.889	0.838	0.897	0.845
136	0.960	0.889	0.829	0.893	0.839
137	0.971	0.881	0.820	0.891	0.837
138	0.962	0.891	0.814	0.890	0.835
139	0.965	0.894	0.829	0.897	0.845
140	0.965	0.891	0.823	0.894	0.841
141	0.971	0.884	0.826	0.894	0.841
142	0.965	0.886	0.826	0.893	0.839
143	0.962	0.886	0.838	0.896	0.844
144	0.965	0.894	0.823	0.895	0.842
145	0.960	0.884	0.796	0.881	0.821
146	0.968	0.902	0.805	0.893	0.840
147	0.962	0.884	0.820	0.889	0.834
148	0.962	0.886	0.820	0.890	0.835
149	0.960	0.891	0.814	0.889	0.834
150	0.974	0.897	0.811	0.895	0.843
151	0.965	0.884	0.802	0.885	0.827
152	0.971	0.886	0.817	0.892	0.838
153	0.957	0.891	0.814	0.888	0.832
154	0.968	0.894	0.817	0.894	0.841
155	0.965	0.897	0.832	0.899	0.848
156	0.960	0.886	0.817	0.888	0.832
157	0.960	0.889	0.823	0.891	0.837
158	0.962	0.902	0.817	0.895	0.842
159	0.962	0.894	0.799	0.887	0.830
160	0.965	0.876	0.823	0.888	0.832
161	0.971	0.891	0.820	0.895	0.842
162	0.965	0.891	0.820	0.893	0.839
163	0.971	0.889	0.826	0.896	0.844
164	0.977	0.886	0.817	0.894	0.841
165	0.968	0.876	0.826	0.890	0.835
166	0.971	0.873	0.820	0.888	0.832
167	0.968	0.891	0.826	0.896	0.844

168	0.977	0.897	0.823	0.900	0.849
169	0.962	0.897	0.820	0.894	0.841
170	0.962	0.889	0.826	0.893	0.839
171	0.965	0.879	0.826	0.890	0.835
172	0.965	0.897	0.823	0.896	0.844
173	0.974	0.897	0.835	0.903	0.854
174	0.971	0.884	0.826	0.894	0.841
175	0.965	0.894	0.808	0.890	0.835
176	0.971	0.894	0.805	0.891	0.837
177	0.968	0.894	0.826	0.897	0.845
178	0.968	0.894	0.826	0.897	0.845
179	0.960	0.889	0.838	0.896	0.844
180	0.960	0.894	0.832	0.896	0.844
181	0.965	0.889	0.826	0.894	0.841
182	0.962	0.879	0.835	0.892	0.838
183	0.977	0.886	0.835	0.900	0.849
184	0.962	0.884	0.823	0.890	0.835
185	0.962	0.886	0.823	0.891	0.837
186	0.965	0.884	0.829	0.893	0.839
187	0.968	0.897	0.814	0.894	0.841
188	0.971	0.889	0.829	0.897	0.845
189	0.957	0.879	0.826	0.888	0.831
190	0.962	0.884	0.826	0.891	0.837
191	0.971	0.889	0.838	0.900	0.849
192	0.968	0.891	0.841	0.901	0.851
193	0.965	0.897	0.817	0.894	0.841
194	0.974	0.894	0.835	0.902	0.852
195	0.971	0.897	0.850	0.906	0.859
196	0.971	0.881	0.820	0.891	0.837
197	0.968	0.884	0.832	0.895	0.842
198	0.965	0.884	0.823	0.891	0.837
199	0.971	0.886	0.835	0.898	0.846
200	0.962	0.879	0.838	0.893	0.839
201	0.968	0.884	0.832	0.895	0.842
202	0.977	0.886	0.838	0.901	0.851
203	0.960	0.886	0.829	0.892	0.838
204	0.965	0.876	0.826	0.889	0.834
205	0.962	0.881	0.829	0.891	0.837
206	0.962	0.884	0.817	0.888	0.832
207	0.968	0.884	0.838	0.897	0.845
208	0.965	0.889	0.835	0.897	0.845
209	0.962	0.889	0.823	0.892	0.838

210	0.965	0.886	0.832	0.895	0.842
211	0.968	0.884	0.829	0.894	0.841
212	0.971	0.894	0.826	0.898	0.847
213	0.974	0.889	0.838	0.901	0.851
214	0.962	0.889	0.823	0.892	0.838
215	0.968	0.884	0.826	0.893	0.839
216	0.968	0.884	0.820	0.891	0.837
217	0.957	0.894	0.823	0.892	0.838
218	0.968	0.886	0.844	0.900	0.849
219	0.965	0.879	0.811	0.886	0.828
220	0.960	0.876	0.835	0.890	0.835
221	0.965	0.884	0.835	0.895	0.842
222	0.971	0.881	0.823	0.892	0.838
223	0.965	0.876	0.826	0.889	0.834
224	0.962	0.894	0.832	0.897	0.845
225	0.962	0.884	0.829	0.892	0.838
226	0.960	0.881	0.820	0.888	0.831
227	0.962	0.879	0.823	0.888	0.832
228	0.962	0.876	0.835	0.891	0.837
229	0.960	0.886	0.832	0.893	0.839
230	0.962	0.889	0.829	0.894	0.841
231	0.971	0.889	0.823	0.895	0.842
232	0.968	0.881	0.820	0.890	0.835
233	0.968	0.884	0.826	0.893	0.839
234	0.962	0.886	0.826	0.892	0.838
235	0.962	0.881	0.835	0.893	0.839
236	0.965	0.873	0.835	0.891	0.837
237	0.965	0.886	0.820	0.891	0.837
238	0.971	0.886	0.835	0.898	0.847
239	0.968	0.884	0.832	0.895	0.842
240	0.971	0.884	0.838	0.898	0.846
241	0.962	0.894	0.835	0.898	0.846
242	0.968	0.886	0.820	0.892	0.838
243	0.971	0.897	0.826	0.899	0.848
244	0.968	0.897	0.835	0.901	0.851
245	0.968	0.884	0.829	0.894	0.841
246	0.971	0.884	0.826	0.894	0.841
247	0.960	0.873	0.823	0.886	0.828
248	0.965	0.884	0.832	0.894	0.841
249	0.968	0.886	0.829	0.895	0.842
250	0.974	0.876	0.829	0.893	0.839
251	0.962	0.884	0.847	0.898	0.846

252	0.971	0.897	0.844	0.904	0.856
253	0.971	0.881	0.850	0.901	0.851
254	0.962	0.884	0.835	0.894	0.841
255	0.968	0.884	0.835	0.896	0.844
256	0.965	0.881	0.829	0.892	0.838
257	0.965	0.855	0.850	0.889	0.834
258	0.965	0.879	0.838	0.894	0.841
259	0.971	0.886	0.823	0.894	0.841
260	0.971	0.873	0.838	0.894	0.841
261	0.968	0.879	0.823	0.890	0.835
262	0.965	0.881	0.838	0.895	0.842
263	0.960	0.881	0.838	0.893	0.839
264	0.971	0.881	0.838	0.897	0.845
265	0.965	0.889	0.838	0.898	0.846
266	0.957	0.876	0.820	0.885	0.827
267	0.968	0.891	0.838	0.900	0.849
268	0.962	0.876	0.829	0.889	0.834
269	0.968	0.889	0.829	0.896	0.844
270	0.962	0.897	0.829	0.897	0.845
271	0.965	0.876	0.838	0.893	0.839
272	0.968	0.879	0.838	0.895	0.842
273	0.965	0.886	0.829	0.894	0.841
274	0.968	0.881	0.838	0.896	0.844
275	0.977	0.889	0.820	0.896	0.844
276	0.968	0.886	0.829	0.895	0.842
277	0.968	0.881	0.826	0.892	0.838
278	0.968	0.881	0.838	0.896	0.844
279	0.965	0.884	0.826	0.892	0.838
280	0.968	0.886	0.817	0.891	0.837
281	0.965	0.889	0.838	0.898	0.846
282	0.962	0.886	0.838	0.896	0.844
283	0.965	0.886	0.832	0.895	0.842
284	0.965	0.881	0.829	0.892	0.838
285	0.962	0.889	0.829	0.894	0.841
286	0.974	0.876	0.841	0.897	0.845
287	0.960	0.876	0.838	0.891	0.837
288	0.960	0.886	0.823	0.890	0.835
289	0.968	0.889	0.823	0.894	0.841
290	0.968	0.891	0.829	0.897	0.845
291	0.965	0.884	0.826	0.892	0.838
292	0.965	0.899	0.838	0.902	0.852
293	0.968	0.884	0.823	0.892	0.838

294	0.974	0.879	0.829	0.894	0.841
295	0.957	0.886	0.838	0.894	0.841
296	0.962	0.873	0.832	0.889	0.834
297	0.965	0.881	0.841	0.896	0.844
298	0.957	0.891	0.838	0.896	0.844
299	0.965	0.881	0.850	0.899	0.848
300	0.962	0.881	0.829	0.891	0.837
301	0.974	0.873	0.835	0.894	0.841
302	0.962	0.879	0.838	0.893	0.839
303	0.957	0.876	0.832	0.888	0.832
304	0.962	0.879	0.832	0.891	0.837
305	0.968	0.858	0.826	0.884	0.825
306	0.974	0.868	0.838	0.893	0.840
307	0.971	0.871	0.832	0.891	0.837
308	0.962	0.884	0.847	0.898	0.846
309	0.968	0.881	0.826	0.892	0.838
310	0.965	0.891	0.835	0.898	0.846
311	0.968	0.871	0.844	0.894	0.841
312	0.962	0.879	0.850	0.897	0.845
313	0.954	0.891	0.844	0.897	0.845
314	0.968	0.879	0.838	0.895	0.842
315	0.960	0.871	0.847	0.892	0.838
316	0.962	0.881	0.841	0.895	0.842
317	0.962	0.889	0.838	0.897	0.845
318	0.968	0.873	0.841	0.894	0.841
319	0.968	0.871	0.850	0.896	0.844
320	0.968	0.879	0.841	0.896	0.844
321	0.965	0.879	0.844	0.896	0.844
322	0.962	0.886	0.835	0.895	0.842
323	0.965	0.889	0.841	0.899	0.848
324	0.965	0.876	0.841	0.894	0.841
325	0.962	0.881	0.832	0.892	0.838
326	0.960	0.881	0.835	0.892	0.838
327	0.968	0.884	0.826	0.893	0.839
328	0.960	0.886	0.829	0.892	0.838
329	0.965	0.879	0.847	0.897	0.845
330	0.960	0.873	0.832	0.888	0.832
331	0.954	0.884	0.835	0.891	0.837
332	0.960	0.871	0.838	0.889	0.834
333	0.960	0.881	0.832	0.891	0.837
334	0.968	0.884	0.844	0.899	0.848
335	0.965	0.881	0.832	0.893	0.839

336	0.971	0.879	0.823	0.891	0.837
337	0.951	0.873	0.844	0.889	0.834
338	0.965	0.894	0.832	0.898	0.846
339	0.965	0.886	0.826	0.893	0.839
340	0.962	0.889	0.826	0.893	0.839
341	0.962	0.889	0.847	0.900	0.849
342	0.960	0.891	0.826	0.893	0.839
343	0.968	0.876	0.835	0.893	0.839
344	0.971	0.876	0.844	0.897	0.845
345	0.962	0.871	0.835	0.889	0.834
346	0.957	0.873	0.838	0.889	0.834
347	0.962	0.879	0.838	0.893	0.839
348	0.957	0.866	0.838	0.887	0.830
349	0.965	0.884	0.838	0.896	0.844
350	0.957	0.871	0.835	0.888	0.831
351	0.960	0.879	0.835	0.891	0.837
352	0.957	0.868	0.817	0.881	0.821
353	0.977	0.886	0.823	0.896	0.844
354	0.968	0.879	0.832	0.893	0.839
355	0.960	0.868	0.832	0.887	0.830
356	0.962	0.889	0.838	0.897	0.845
357	0.971	0.884	0.832	0.896	0.844
358	0.962	0.873	0.838	0.891	0.837
359	0.962	0.871	0.838	0.890	0.835
360	0.960	0.868	0.823	0.884	0.825
361	0.962	0.881	0.826	0.890	0.835
362	0.968	0.876	0.832	0.892	0.838
363	0.977	0.889	0.844	0.903	0.855
364	0.971	0.881	0.844	0.899	0.848
365	0.965	0.871	0.850	0.895	0.842
366	0.965	0.879	0.823	0.889	0.834
367	0.962	0.886	0.850	0.900	0.849
368	0.965	0.881	0.841	0.896	0.844
369	0.965	0.881	0.838	0.895	0.842
370	0.965	0.879	0.823	0.889	0.834
371	0.965	0.873	0.841	0.893	0.839
372	0.965	0.873	0.853	0.897	0.845
373	0.962	0.886	0.832	0.894	0.841
374	0.962	0.886	0.826	0.892	0.838
375	0.957	0.876	0.838	0.890	0.835
376	0.968	0.868	0.844	0.893	0.839
377	0.965	0.881	0.847	0.898	0.846

378	0.968	0.873	0.841	0.894	0.841
379	0.974	0.873	0.847	0.898	0.847
380	0.974	0.879	0.850	0.901	0.851
381	0.965	0.873	0.847	0.895	0.842
382	0.957	0.884	0.838	0.893	0.839
383	0.968	0.884	0.829	0.894	0.841
384	0.962	0.876	0.832	0.890	0.835
385	0.960	0.876	0.847	0.894	0.841
386	0.962	0.876	0.844	0.894	0.841
387	0.957	0.876	0.832	0.888	0.832
388	0.968	0.860	0.838	0.888	0.832
389	0.971	0.871	0.844	0.895	0.842
390	0.965	0.884	0.841	0.897	0.845
391	0.968	0.879	0.844	0.897	0.845
392	0.974	0.876	0.835	0.895	0.842
393	0.971	0.868	0.838	0.892	0.838
394	0.962	0.884	0.838	0.895	0.842
395	0.971	0.873	0.841	0.895	0.842
396	0.957	0.881	0.841	0.893	0.839
397	0.962	0.884	0.853	0.900	0.849
398	0.965	0.876	0.832	0.891	0.837
399	0.962	0.879	0.829	0.890	0.835
400	0.968	0.891	0.850	0.903	0.855
401	0.957	0.863	0.844	0.888	0.831
402	0.962	0.889	0.835	0.896	0.844
403	0.968	0.876	0.844	0.896	0.844
404	0.971	0.881	0.838	0.897	0.845
405	0.960	0.879	0.847	0.895	0.842
406	0.957	0.881	0.850	0.896	0.844
407	0.960	0.884	0.844	0.896	0.844
408	0.962	0.873	0.838	0.891	0.837
409	0.968	0.876	0.844	0.896	0.844
410	0.957	0.873	0.847	0.892	0.838
411	0.960	0.879	0.844	0.894	0.841
412	0.962	0.884	0.847	0.898	0.846
413	0.962	0.886	0.850	0.900	0.849
414	0.965	0.868	0.832	0.888	0.832
415	0.960	0.873	0.841	0.891	0.837
416	0.957	0.884	0.838	0.893	0.839
417	0.962	0.881	0.838	0.894	0.841
418	0.965	0.889	0.850	0.902	0.852
419	0.968	0.871	0.847	0.895	0.842

420	0.960	0.886	0.838	0.895	0.842
421	0.962	0.881	0.850	0.898	0.846
422	0.968	0.860	0.853	0.893	0.840
423	0.962	0.884	0.847	0.898	0.846
424	0.962	0.873	0.826	0.888	0.831
425	0.971	0.871	0.838	0.893	0.839
426	0.968	0.881	0.838	0.896	0.844
427	0.965	0.879	0.835	0.893	0.839
428	0.965	0.876	0.835	0.892	0.838
429	0.965	0.881	0.844	0.897	0.845
430	0.977	0.884	0.844	0.902	0.852
431	0.954	0.881	0.832	0.889	0.834
432	0.968	0.876	0.838	0.894	0.841
433	0.965	0.858	0.844	0.888	0.832
434	0.977	0.876	0.838	0.897	0.845
435	0.971	0.873	0.847	0.897	0.845
436	0.968	0.871	0.835	0.891	0.837
437	0.968	0.886	0.850	0.902	0.852
438	0.968	0.884	0.844	0.899	0.848
439	0.968	0.876	0.853	0.899	0.848
440	0.960	0.879	0.838	0.892	0.838
441	0.965	0.881	0.847	0.898	0.846
442	0.965	0.871	0.847	0.894	0.841
443	0.974	0.871	0.847	0.897	0.845
444	0.965	0.884	0.838	0.896	0.844
445	0.962	0.879	0.856	0.899	0.848
446	0.962	0.881	0.841	0.895	0.842
447	0.965	0.871	0.832	0.889	0.834
448	0.974	0.876	0.841	0.897	0.845
449	0.971	0.871	0.844	0.895	0.842
450	0.968	0.873	0.832	0.891	0.837
451	0.960	0.879	0.841	0.893	0.839
452	0.971	0.886	0.859	0.905	0.858
453	0.971	0.871	0.847	0.896	0.844
454	0.971	0.879	0.856	0.902	0.852
455	0.968	0.879	0.841	0.896	0.844
456	0.968	0.881	0.853	0.901	0.851
457	0.965	0.884	0.844	0.898	0.846
458	0.968	0.881	0.853	0.901	0.851
459	0.968	0.873	0.850	0.897	0.845
460	0.971	0.879	0.850	0.900	0.849
461	0.962	0.873	0.838	0.891	0.837

462	0.971	0.879	0.844	0.898	0.846
463	0.971	0.884	0.847	0.901	0.851
464	0.968	0.871	0.841	0.893	0.840
465	0.971	0.879	0.829	0.893	0.839
466	0.962	0.868	0.844	0.891	0.837
467	0.968	0.863	0.844	0.891	0.837
468	0.965	0.868	0.856	0.896	0.844
469	0.962	0.879	0.844	0.895	0.842
470	0.960	0.879	0.847	0.895	0.842
471	0.965	0.886	0.850	0.901	0.851
472	0.960	0.884	0.850	0.898	0.846
473	0.968	0.868	0.841	0.892	0.838
474	0.968	0.884	0.838	0.897	0.845
475	0.965	0.884	0.844	0.898	0.846
476	0.965	0.876	0.844	0.895	0.842
477	0.965	0.871	0.841	0.892	0.838
478	0.962	0.879	0.859	0.900	0.849
479	0.971	0.881	0.856	0.903	0.854
480	0.965	0.871	0.844	0.893	0.839
481	0.962	0.871	0.853	0.895	0.842
482	0.962	0.873	0.847	0.894	0.841
483	0.962	0.866	0.856	0.894	0.841
484	0.965	0.886	0.838	0.897	0.845
485	0.968	0.879	0.850	0.899	0.848
486	0.954	0.868	0.847	0.889	0.834
487	0.962	0.886	0.826	0.892	0.838
488	0.962	0.866	0.841	0.889	0.834
489	0.968	0.879	0.856	0.901	0.851
490	0.957	0.871	0.850	0.892	0.838
491	0.960	0.866	0.841	0.888	0.832
492	0.968	0.871	0.844	0.894	0.841
493	0.962	0.873	0.838	0.891	0.837
494	0.965	0.866	0.850	0.893	0.840
495	0.962	0.884	0.841	0.896	0.844
496	0.968	0.891	0.838	0.900	0.849
497	0.962	0.868	0.841	0.890	0.835
498	0.954	0.876	0.841	0.890	0.835
499	0.965	0.881	0.847	0.898	0.846
500	0.971	0.868	0.832	0.890	0.835
501	0.960	0.881	0.850	0.897	0.845
502	0.962	0.879	0.829	0.890	0.835
503	0.977	0.873	0.844	0.898	0.847

504	0.968	0.879	0.841	0.896	0.844
505	0.968	0.871	0.841	0.893	0.839
506	0.965	0.868	0.841	0.891	0.837
507	0.965	0.873	0.850	0.896	0.844
508	0.965	0.873	0.823	0.888	0.831
509	0.962	0.889	0.850	0.901	0.851
510	0.962	0.868	0.841	0.890	0.835
511	0.968	0.873	0.826	0.889	0.834
512	0.968	0.879	0.826	0.891	0.837
513	0.965	0.879	0.844	0.896	0.844
514	0.968	0.886	0.829	0.895	0.842
515	0.968	0.868	0.844	0.893	0.839
516	0.965	0.879	0.835	0.893	0.839
517	0.965	0.879	0.841	0.895	0.842
518	0.971	0.886	0.835	0.898	0.847
519	0.960	0.876	0.820	0.886	0.828
520	0.971	0.879	0.838	0.896	0.844
521	0.965	0.868	0.826	0.887	0.830
522	0.971	0.876	0.835	0.894	0.841
523	0.968	0.881	0.832	0.894	0.841
524	0.960	0.879	0.838	0.892	0.838
525	0.971	0.868	0.823	0.888	0.831
526	0.974	0.871	0.847	0.897	0.845
527	0.965	0.881	0.847	0.898	0.846
528	0.965	0.876	0.847	0.896	0.844
529	0.965	0.873	0.829	0.889	0.834
530	0.968	0.881	0.826	0.892	0.838
531	0.968	0.881	0.841	0.897	0.845
532	0.962	0.879	0.844	0.895	0.842
533	0.971	0.876	0.832	0.893	0.839
534	0.974	0.873	0.832	0.893	0.839
535	0.968	0.871	0.832	0.890	0.835
536	0.968	0.873	0.835	0.892	0.838
537	0.962	0.873	0.832	0.889	0.834
538	0.957	0.863	0.841	0.887	0.830
539	0.968	0.879	0.844	0.897	0.845
540	0.965	0.871	0.829	0.888	0.832
541	0.971	0.879	0.832	0.894	0.841
542	0.965	0.881	0.832	0.893	0.839
543	0.968	0.881	0.850	0.900	0.849
544	0.968	0.876	0.838	0.894	0.841
545	0.962	0.863	0.823	0.883	0.824

546	0.974	0.881	0.823	0.893	0.839
547	0.965	0.873	0.838	0.892	0.838
548	0.962	0.871	0.823	0.886	0.828
549	0.968	0.881	0.841	0.897	0.845
550	0.965	0.860	0.844	0.889	0.834
551	0.960	0.876	0.814	0.884	0.825
552	0.957	0.886	0.841	0.895	0.842
553	0.968	0.879	0.832	0.893	0.839
554	0.965	0.884	0.844	0.898	0.846
555	0.962	0.879	0.844	0.895	0.842
556	0.968	0.873	0.844	0.895	0.842
557	0.962	0.871	0.829	0.888	0.831
558	0.968	0.884	0.841	0.898	0.846
559	0.965	0.881	0.847	0.898	0.846
560	0.968	0.873	0.838	0.893	0.839
561	0.960	0.884	0.829	0.891	0.837
562	0.968	0.871	0.826	0.888	0.832
563	0.968	0.868	0.835	0.890	0.835
564	0.971	0.879	0.844	0.898	0.846
565	0.960	0.871	0.826	0.886	0.828
566	0.962	0.879	0.841	0.894	0.841
567	0.968	0.879	0.826	0.891	0.837
568	0.962	0.876	0.832	0.890	0.835
569	0.957	0.876	0.835	0.889	0.834
570	0.965	0.879	0.835	0.893	0.839
571	0.965	0.879	0.826	0.890	0.835
572	0.965	0.881	0.832	0.893	0.839
573	0.968	0.868	0.832	0.889	0.834
574	0.968	0.873	0.838	0.893	0.839
575	0.968	0.873	0.832	0.891	0.837
576	0.965	0.866	0.853	0.894	0.841
577	0.960	0.873	0.832	0.888	0.832
578	0.962	0.873	0.841	0.892	0.838
579	0.971	0.868	0.844	0.894	0.841
580	0.962	0.876	0.835	0.891	0.837
581	0.960	0.863	0.838	0.887	0.830
582	0.962	0.881	0.835	0.893	0.839
583	0.965	0.873	0.835	0.891	0.837
584	0.962	0.868	0.835	0.888	0.832
585	0.962	0.871	0.841	0.891	0.837
586	0.968	0.879	0.832	0.893	0.839
587	0.968	0.881	0.826	0.892	0.838

588	0.965	0.879	0.841	0.895	0.842
589	0.962	0.881	0.832	0.892	0.838
590	0.962	0.879	0.841	0.894	0.841
591	0.962	0.884	0.844	0.897	0.845
592	0.974	0.873	0.844	0.897	0.845
593	0.968	0.868	0.835	0.890	0.835
594	0.974	0.886	0.844	0.902	0.852
595	0.965	0.881	0.832	0.893	0.839
596	0.968	0.889	0.829	0.896	0.844
597	0.960	0.866	0.829	0.885	0.827
598	0.957	0.881	0.844	0.894	0.841
599	0.962	0.871	0.829	0.888	0.831
600	0.968	0.873	0.835	0.892	0.838
601	0.971	0.876	0.847	0.898	0.846
602	0.962	0.886	0.835	0.895	0.842
603	0.965	0.873	0.844	0.894	0.841
604	0.965	0.879	0.838	0.894	0.841
605	0.965	0.881	0.838	0.895	0.842
606	0.965	0.876	0.838	0.893	0.839
607	0.965	0.873	0.823	0.888	0.831
608	0.960	0.873	0.838	0.890	0.835
609	0.965	0.860	0.838	0.888	0.831
610	0.965	0.876	0.826	0.889	0.834
611	0.974	0.873	0.832	0.893	0.839
612	0.968	0.881	0.835	0.895	0.842
613	0.968	0.871	0.841	0.893	0.839
614	0.960	0.881	0.829	0.890	0.835
615	0.962	0.876	0.829	0.889	0.834
616	0.965	0.884	0.829	0.893	0.839
617	0.965	0.866	0.844	0.891	0.837
618	0.968	0.889	0.838	0.899	0.848
619	0.960	0.866	0.832	0.886	0.828
620	0.974	0.886	0.841	0.901	0.851
621	0.965	0.876	0.844	0.895	0.842
622	0.960	0.881	0.829	0.890	0.835
623	0.960	0.873	0.835	0.889	0.834
624	0.974	0.866	0.844	0.894	0.841
625	0.962	0.863	0.838	0.888	0.831
626	0.968	0.868	0.838	0.891	0.837
627	0.968	0.886	0.829	0.895	0.842
628	0.962	0.886	0.841	0.897	0.845
629	0.960	0.871	0.823	0.885	0.827

630	0.971	0.868	0.838	0.892	0.838
631	0.965	0.868	0.823	0.886	0.828
632	0.957	0.863	0.859	0.892	0.838
633	0.965	0.871	0.838	0.891	0.837
634	0.960	0.866	0.832	0.886	0.828
635	0.962	0.873	0.838	0.891	0.837
636	0.974	0.868	0.829	0.890	0.835
637	0.977	0.881	0.820	0.893	0.839
638	0.971	0.879	0.844	0.898	0.846
639	0.968	0.876	0.835	0.893	0.839
640	0.965	0.863	0.832	0.887	0.830
641	0.968	0.871	0.841	0.893	0.839
642	0.957	0.873	0.832	0.888	0.831
643	0.962	0.876	0.829	0.889	0.834
644	0.968	0.873	0.823	0.888	0.832
645	0.971	0.884	0.832	0.896	0.844
646	0.965	0.881	0.841	0.896	0.844
647	0.968	0.889	0.835	0.898	0.846
648	0.965	0.871	0.823	0.887	0.830
649	0.968	0.881	0.838	0.896	0.844
650	0.968	0.876	0.826	0.890	0.835
651	0.965	0.868	0.841	0.891	0.837
652	0.968	0.871	0.850	0.896	0.844
653	0.954	0.876	0.835	0.888	0.832
654	0.965	0.860	0.838	0.888	0.831
655	0.971	0.876	0.826	0.891	0.837
656	0.968	0.871	0.835	0.891	0.837
657	0.968	0.876	0.832	0.892	0.838
658	0.954	0.881	0.841	0.892	0.838
659	0.968	0.881	0.835	0.895	0.842
660	0.974	0.866	0.826	0.888	0.832
661	0.974	0.879	0.835	0.896	0.844
662	0.965	0.873	0.847	0.895	0.842
663	0.965	0.868	0.835	0.889	0.834
664	0.965	0.866	0.823	0.885	0.827
665	0.968	0.876	0.814	0.887	0.830
666	0.974	0.879	0.838	0.897	0.845
667	0.957	0.886	0.844	0.896	0.844
668	0.965	0.876	0.826	0.889	0.834
669	0.974	0.873	0.829	0.892	0.838
670	0.962	0.881	0.841	0.895	0.842
671	0.968	0.871	0.835	0.891	0.837

672	0.962	0.891	0.844	0.900	0.849
673	0.965	0.871	0.826	0.888	0.831
674	0.974	0.873	0.835	0.894	0.841
675	0.960	0.866	0.829	0.885	0.827
676	0.968	0.879	0.841	0.896	0.844
677	0.962	0.876	0.838	0.892	0.838
678	0.968	0.873	0.835	0.892	0.838
679	0.962	0.873	0.841	0.892	0.838
680	0.965	0.871	0.832	0.889	0.834
681	0.965	0.876	0.844	0.895	0.842
682	0.962	0.868	0.829	0.887	0.830
683	0.971	0.868	0.829	0.889	0.834
684	0.962	0.873	0.841	0.892	0.838
685	0.971	0.866	0.835	0.890	0.835
686	0.971	0.866	0.838	0.891	0.837
687	0.968	0.881	0.826	0.892	0.838
688	0.968	0.873	0.844	0.895	0.842
689	0.971	0.884	0.829	0.895	0.842
690	0.971	0.876	0.832	0.893	0.839
691	0.971	0.876	0.832	0.893	0.839
692	0.962	0.858	0.844	0.888	0.831
693	0.968	0.881	0.835	0.895	0.842
694	0.957	0.876	0.829	0.888	0.831
695	0.968	0.889	0.829	0.896	0.844
696	0.971	0.879	0.835	0.895	0.842
697	0.962	0.884	0.844	0.897	0.845
698	0.957	0.860	0.841	0.886	0.828
699	0.962	0.879	0.844	0.895	0.842
700	0.962	0.868	0.829	0.887	0.830
701	0.965	0.879	0.826	0.890	0.835
702	0.965	0.873	0.820	0.887	0.830
703	0.965	0.876	0.838	0.893	0.839
704	0.968	0.866	0.835	0.889	0.834
705	0.960	0.871	0.829	0.887	0.830
706	0.960	0.886	0.838	0.895	0.842
707	0.974	0.871	0.841	0.895	0.842
708	0.968	0.879	0.841	0.896	0.844
709	0.965	0.876	0.832	0.891	0.837
710	0.962	0.879	0.838	0.893	0.839
711	0.960	0.879	0.835	0.891	0.837
712	0.965	0.891	0.832	0.897	0.845
713	0.954	0.876	0.832	0.888	0.831

714	0.965	0.863	0.832	0.887	0.830
715	0.962	0.866	0.829	0.886	0.828
716	0.968	0.868	0.820	0.886	0.828
717	0.968	0.873	0.847	0.896	0.844
718	0.960	0.876	0.838	0.891	0.837
719	0.957	0.863	0.835	0.885	0.827
720	0.954	0.879	0.841	0.891	0.837
721	0.957	0.866	0.856	0.892	0.838
722	0.957	0.873	0.847	0.892	0.838
723	0.968	0.876	0.841	0.895	0.842
724	0.960	0.876	0.850	0.895	0.842
725	0.965	0.873	0.844	0.894	0.841
726	0.957	0.873	0.844	0.891	0.837
727	0.971	0.863	0.838	0.890	0.835
728	0.965	0.879	0.838	0.894	0.841
729	0.965	0.879	0.838	0.894	0.841
730	0.971	0.871	0.835	0.892	0.838
731	0.971	0.873	0.844	0.896	0.844
732	0.962	0.866	0.838	0.888	0.832
733	0.962	0.879	0.841	0.894	0.841
734	0.965	0.873	0.829	0.889	0.834
735	0.960	0.876	0.823	0.887	0.830
736	0.960	0.886	0.817	0.888	0.832
737	0.962	0.868	0.835	0.888	0.832
738	0.968	0.866	0.835	0.889	0.834
739	0.971	0.873	0.832	0.892	0.838
740	0.962	0.881	0.838	0.894	0.841
741	0.974	0.863	0.847	0.894	0.841
742	0.962	0.886	0.838	0.896	0.844
743	0.965	0.884	0.850	0.900	0.849
744	0.971	0.871	0.823	0.888	0.832
745	0.960	0.873	0.838	0.890	0.835
746	0.957	0.884	0.841	0.894	0.841
747	0.968	0.871	0.823	0.888	0.831
748	0.971	0.863	0.841	0.891	0.837
749	0.960	0.863	0.832	0.885	0.827
750	0.971	0.871	0.838	0.893	0.839
751	0.962	0.876	0.850	0.896	0.844
752	0.977	0.879	0.838	0.898	0.846
753	0.962	0.863	0.841	0.888	0.832
754	0.965	0.871	0.826	0.888	0.831
755	0.954	0.876	0.829	0.887	0.830

756	0.962	0.860	0.847	0.889	0.834
757	0.962	0.876	0.841	0.893	0.839
758	0.974	0.871	0.844	0.896	0.844
759	0.954	0.868	0.838	0.887	0.830
760	0.968	0.873	0.829	0.890	0.835
761	0.965	0.866	0.841	0.890	0.835
762	0.968	0.858	0.847	0.890	0.835
763	0.965	0.881	0.832	0.893	0.839
764	0.968	0.876	0.826	0.890	0.835
765	0.962	0.879	0.826	0.889	0.834
766	0.960	0.863	0.847	0.889	0.834
767	0.971	0.876	0.853	0.900	0.849
768	0.957	0.873	0.844	0.891	0.837
769	0.968	0.884	0.850	0.901	0.851
770	0.965	0.873	0.826	0.888	0.832
771	0.968	0.876	0.826	0.890	0.835
772	0.960	0.871	0.838	0.889	0.834
773	0.974	0.881	0.820	0.892	0.838
774	0.954	0.873	0.832	0.887	0.830
775	0.962	0.873	0.838	0.891	0.837
776	0.960	0.876	0.841	0.892	0.838
777	0.971	0.860	0.832	0.888	0.831
778	0.960	0.879	0.844	0.894	0.841
779	0.965	0.871	0.832	0.889	0.834
780	0.957	0.873	0.850	0.893	0.839
781	0.962	0.876	0.841	0.893	0.839
782	0.968	0.871	0.847	0.895	0.842
783	0.965	0.881	0.847	0.898	0.846
784	0.960	0.876	0.835	0.890	0.835
785	0.960	0.873	0.847	0.893	0.839
786	0.968	0.871	0.835	0.891	0.837
787	0.965	0.860	0.844	0.889	0.834
788	0.962	0.876	0.826	0.888	0.832
**MCFS**					
**Numbers of features**	**G1**	**G2/M**	**S**	**ACC**	**MCC**
1	0.908	0.636	0.611	0.716	0.575
2	0.931	0.716	0.713	0.784	0.678
3	0.974	0.729	0.757	0.817	0.727
4	0.977	0.747	0.740	0.819	0.729
5	0.980	0.775	0.775	0.842	0.763
6	0.980	0.773	0.775	0.841	0.762
7	0.977	0.793	0.766	0.844	0.767

8	0.983	0.814	0.781	0.858	0.788
9	0.968	0.814	0.778	0.853	0.779
10	0.965	0.786	0.784	0.843	0.766
11	0.971	0.829	0.799	0.866	0.799
12	0.965	0.840	0.787	0.864	0.796
13	0.968	0.827	0.790	0.861	0.792
14	0.968	0.837	0.805	0.870	0.804
15	0.962	0.855	0.787	0.869	0.803
16	0.965	0.868	0.793	0.876	0.814
17	0.965	0.863	0.799	0.876	0.814
18	0.968	0.858	0.805	0.877	0.815
19	0.965	0.866	0.802	0.878	0.817
20	0.965	0.871	0.802	0.880	0.820
21	0.965	0.881	0.796	0.882	0.823
22	0.965	0.868	0.790	0.875	0.813
23	0.971	0.873	0.802	0.883	0.824
24	0.974	0.871	0.814	0.887	0.830
25	0.977	0.868	0.802	0.883	0.824
26	0.968	0.868	0.811	0.883	0.824
27	0.968	0.868	0.802	0.880	0.820
28	0.977	0.866	0.799	0.881	0.821
29	0.968	0.868	0.814	0.884	0.825
30	0.968	0.871	0.796	0.879	0.818
31	0.968	0.871	0.793	0.878	0.817
32	0.974	0.860	0.814	0.883	0.824
33	0.971	0.860	0.805	0.879	0.818
34	0.965	0.863	0.802	0.877	0.815
35	0.968	0.873	0.811	0.885	0.827
36	0.971	0.876	0.793	0.881	0.821
37	0.962	0.873	0.802	0.880	0.820
38	0.965	0.879	0.805	0.884	0.825
39	0.965	0.879	0.808	0.885	0.827
40	0.977	0.879	0.814	0.890	0.835
41	0.965	0.881	0.826	0.891	0.837
42	0.965	0.881	0.820	0.889	0.834
43	0.977	0.884	0.814	0.892	0.838
44	0.968	0.868	0.832	0.889	0.834
45	0.968	0.871	0.814	0.885	0.827
46	0.983	0.868	0.835	0.895	0.842
47	0.977	0.879	0.817	0.891	0.837
48	0.974	0.868	0.826	0.889	0.834
49	0.977	0.879	0.829	0.895	0.842

50	0.974	0.889	0.829	0.898	0.847
51	0.971	0.876	0.838	0.895	0.842
52	0.977	0.907	0.832	0.906	0.859
53	0.974	0.884	0.829	0.896	0.844
54	0.980	0.894	0.838	0.904	0.856
55	0.980	0.891	0.829	0.901	0.851
56	0.974	0.881	0.823	0.893	0.839
57	0.974	0.889	0.832	0.899	0.848
58	0.974	0.889	0.823	0.896	0.844
59	0.974	0.891	0.829	0.899	0.848
60	0.980	0.886	0.826	0.898	0.847
61	0.983	0.889	0.832	0.902	0.852
62	0.980	0.889	0.835	0.902	0.852
63	0.980	0.881	0.841	0.901	0.851
64	0.977	0.884	0.841	0.901	0.851
65	0.983	0.879	0.838	0.900	0.849
66	0.977	0.881	0.835	0.898	0.847
67	0.971	0.884	0.823	0.893	0.839
68	0.974	0.884	0.844	0.901	0.851
69	0.977	0.886	0.835	0.900	0.849
70	0.977	0.881	0.844	0.901	0.851
71	0.971	0.894	0.832	0.900	0.849
72	0.977	0.894	0.832	0.902	0.852
73	0.977	0.881	0.823	0.894	0.841
74	0.962	0.894	0.838	0.899	0.848
75	0.974	0.873	0.829	0.892	0.838
76	0.980	0.879	0.829	0.896	0.844
77	0.977	0.886	0.826	0.897	0.845
78	0.971	0.876	0.835	0.894	0.841
79	0.980	0.886	0.832	0.900	0.849
80	0.977	0.886	0.835	0.900	0.849
81	0.974	0.886	0.829	0.897	0.845
82	0.977	0.886	0.829	0.898	0.847
83	0.974	0.884	0.832	0.897	0.845
84	0.962	0.884	0.835	0.894	0.841
85	0.977	0.881	0.820	0.893	0.839
86	0.974	0.876	0.835	0.895	0.842
87	0.980	0.889	0.829	0.900	0.849
88	0.968	0.889	0.823	0.894	0.841
89	0.980	0.884	0.832	0.899	0.848
90	0.971	0.889	0.832	0.898	0.846
91	0.968	0.881	0.835	0.895	0.842

92	0.968	0.884	0.832	0.895	0.842
93	0.980	0.889	0.832	0.901	0.851
94	0.968	0.891	0.838	0.900	0.849
95	0.974	0.876	0.823	0.891	0.837
96	0.968	0.873	0.835	0.892	0.838
97	0.968	0.873	0.835	0.892	0.838
98	0.974	0.881	0.844	0.900	0.849
99	0.980	0.876	0.832	0.896	0.844
100	0.974	0.889	0.838	0.901	0.851
101	0.965	0.876	0.823	0.888	0.832
102	0.974	0.894	0.841	0.903	0.855
103	0.974	0.881	0.832	0.896	0.844
104	0.968	0.886	0.829	0.895	0.842
105	0.974	0.891	0.832	0.900	0.849
106	0.971	0.879	0.826	0.892	0.838
107	0.974	0.884	0.838	0.899	0.848
108	0.971	0.886	0.832	0.897	0.845
109	0.977	0.886	0.835	0.900	0.849
110	0.974	0.899	0.829	0.902	0.852
111	0.974	0.881	0.841	0.899	0.848
112	0.980	0.884	0.838	0.901	0.851
113	0.968	0.886	0.844	0.900	0.849
114	0.980	0.894	0.835	0.903	0.855
115	0.965	0.894	0.838	0.900	0.849
116	0.971	0.884	0.838	0.898	0.847
117	0.971	0.876	0.850	0.899	0.848
118	0.968	0.873	0.829	0.890	0.835
119	0.971	0.879	0.835	0.895	0.842
120	0.974	0.879	0.838	0.897	0.845
121	0.974	0.889	0.826	0.897	0.845
122	0.971	0.881	0.832	0.895	0.842
123	0.965	0.886	0.829	0.894	0.841
124	0.965	0.891	0.823	0.894	0.841
125	0.980	0.881	0.832	0.898	0.847
126	0.971	0.886	0.835	0.898	0.846
127	0.974	0.873	0.823	0.890	0.835
128	0.974	0.876	0.841	0.897	0.845
129	0.968	0.886	0.820	0.892	0.838
130	0.971	0.879	0.844	0.898	0.846
131	0.977	0.886	0.835	0.900	0.849
132	0.962	0.886	0.838	0.896	0.844
133	0.968	0.881	0.835	0.895	0.842

134	0.971	0.894	0.838	0.902	0.852
135	0.968	0.894	0.823	0.896	0.844
136	0.980	0.889	0.823	0.898	0.847
137	0.977	0.881	0.832	0.897	0.845
138	0.974	0.889	0.844	0.903	0.854
139	0.977	0.886	0.838	0.901	0.851
140	0.968	0.886	0.832	0.896	0.844
141	0.968	0.886	0.844	0.900	0.849
142	0.977	0.871	0.838	0.895	0.842
143	0.971	0.881	0.850	0.901	0.851
144	0.971	0.879	0.838	0.896	0.844
145	0.977	0.876	0.841	0.898	0.846
146	0.974	0.886	0.847	0.903	0.854
147	0.968	0.886	0.850	0.902	0.852
148	0.962	0.879	0.844	0.895	0.842
149	0.968	0.886	0.835	0.897	0.845
150	0.971	0.879	0.850	0.900	0.849
151	0.965	0.873	0.826	0.888	0.832
152	0.974	0.879	0.841	0.898	0.846
153	0.968	0.881	0.838	0.896	0.844
154	0.971	0.881	0.847	0.900	0.849
155	0.974	0.876	0.838	0.896	0.844
156	0.977	0.871	0.844	0.897	0.845
157	0.968	0.886	0.844	0.900	0.849
158	0.974	0.871	0.841	0.895	0.842
159	0.968	0.876	0.835	0.893	0.839
160	0.980	0.871	0.847	0.899	0.848
161	0.968	0.881	0.835	0.895	0.842
162	0.971	0.853	0.841	0.888	0.831
163	0.968	0.879	0.847	0.898	0.847
164	0.971	0.876	0.838	0.895	0.842
165	0.965	0.876	0.856	0.899	0.848
166	0.977	0.876	0.847	0.900	0.849
167	0.962	0.868	0.844	0.891	0.837
168	0.980	0.879	0.835	0.898	0.847
169	0.977	0.886	0.853	0.905	0.858
170	0.974	0.879	0.823	0.892	0.838
171	0.971	0.871	0.841	0.894	0.841
172	0.968	0.871	0.850	0.896	0.844
173	0.977	0.897	0.832	0.903	0.854
174	0.977	0.881	0.829	0.896	0.844
175	0.971	0.881	0.841	0.898	0.846

176	0.971	0.889	0.832	0.898	0.847
177	0.965	0.879	0.841	0.895	0.842
178	0.971	0.881	0.844	0.899	0.848
179	0.971	0.866	0.826	0.888	0.831
180	0.977	0.866	0.823	0.888	0.832
181	0.977	0.881	0.826	0.895	0.842
182	0.977	0.879	0.820	0.892	0.838
183	0.974	0.873	0.820	0.889	0.834
184	0.965	0.876	0.826	0.889	0.834
185	0.971	0.868	0.835	0.891	0.837
186	0.971	0.884	0.829	0.895	0.842
187	0.980	0.879	0.838	0.899	0.848
188	0.971	0.884	0.838	0.898	0.846
189	0.971	0.879	0.835	0.895	0.842
190	0.965	0.886	0.826	0.893	0.839
191	0.974	0.873	0.835	0.894	0.841
192	0.971	0.894	0.820	0.896	0.844
193	0.974	0.884	0.835	0.898	0.846
194	0.965	0.886	0.835	0.896	0.844
195	0.971	0.881	0.832	0.895	0.842
196	0.971	0.873	0.835	0.893	0.839
197	0.968	0.879	0.826	0.891	0.837
198	0.977	0.879	0.841	0.899	0.848
199	0.968	0.879	0.841	0.896	0.844
200	0.968	0.876	0.835	0.893	0.839
201	0.974	0.889	0.826	0.897	0.845
202	0.968	0.886	0.844	0.900	0.849
203	0.971	0.886	0.835	0.898	0.846
204	0.968	0.891	0.838	0.900	0.849
205	0.971	0.886	0.850	0.903	0.854
206	0.971	0.884	0.832	0.896	0.844
207	0.971	0.889	0.832	0.898	0.847
208	0.974	0.881	0.832	0.896	0.844
209	0.971	0.871	0.838	0.893	0.839
210	0.965	0.886	0.820	0.891	0.837
211	0.968	0.886	0.850	0.902	0.852
212	0.977	0.891	0.841	0.903	0.855
213	0.974	0.879	0.838	0.897	0.845
214	0.971	0.894	0.817	0.895	0.842
215	0.971	0.881	0.829	0.894	0.841
216	0.974	0.891	0.841	0.903	0.854
217	0.971	0.873	0.838	0.894	0.841

218	0.962	0.894	0.823	0.894	0.841
219	0.965	0.886	0.850	0.901	0.851
220	0.965	0.886	0.847	0.900	0.849
221	0.968	0.881	0.847	0.899	0.848
222	0.968	0.884	0.817	0.890	0.835
223	0.971	0.886	0.829	0.896	0.844
224	0.971	0.879	0.838	0.896	0.844
225	0.960	0.886	0.856	0.901	0.851
226	0.962	0.886	0.835	0.895	0.842
227	0.971	0.889	0.826	0.896	0.844
228	0.965	0.889	0.841	0.899	0.848
229	0.977	0.876	0.835	0.896	0.844
230	0.971	0.884	0.835	0.897	0.845
231	0.962	0.886	0.841	0.897	0.845
232	0.974	0.873	0.835	0.894	0.841
233	0.965	0.871	0.847	0.894	0.841
234	0.965	0.886	0.847	0.900	0.849
235	0.960	0.891	0.841	0.898	0.846
236	0.974	0.886	0.832	0.898	0.846
237	0.962	0.897	0.838	0.900	0.849
238	0.971	0.881	0.847	0.900	0.849
239	0.974	0.886	0.844	0.902	0.852
240	0.965	0.879	0.859	0.901	0.851
241	0.962	0.891	0.841	0.899	0.848
242	0.971	0.886	0.838	0.899	0.848
243	0.971	0.894	0.832	0.900	0.849
244	0.968	0.886	0.844	0.900	0.849
245	0.968	0.891	0.835	0.899	0.848
246	0.968	0.886	0.841	0.899	0.848
247	0.968	0.884	0.835	0.896	0.844
248	0.968	0.894	0.844	0.903	0.853
249	0.971	0.889	0.826	0.896	0.844
250	0.974	0.879	0.832	0.895	0.842
251	0.968	0.879	0.838	0.895	0.842
252	0.962	0.879	0.847	0.896	0.844
253	0.968	0.889	0.847	0.902	0.852
254	0.968	0.891	0.823	0.895	0.842
255	0.971	0.889	0.832	0.898	0.846
256	0.968	0.881	0.835	0.895	0.842
257	0.968	0.886	0.847	0.901	0.851
258	0.971	0.884	0.838	0.898	0.846
259	0.971	0.876	0.826	0.891	0.837

260	0.971	0.876	0.835	0.894	0.841
261	0.957	0.884	0.847	0.896	0.844
262	0.965	0.881	0.829	0.892	0.838
263	0.965	0.884	0.826	0.892	0.838
264	0.960	0.881	0.844	0.895	0.842
265	0.968	0.884	0.838	0.897	0.845
266	0.965	0.881	0.850	0.899	0.848
267	0.974	0.873	0.835	0.894	0.841
268	0.968	0.884	0.829	0.894	0.841
269	0.965	0.886	0.838	0.897	0.845
270	0.957	0.879	0.847	0.894	0.841
271	0.971	0.871	0.841	0.894	0.841
272	0.968	0.881	0.838	0.896	0.844
273	0.965	0.873	0.841	0.893	0.839
274	0.974	0.876	0.838	0.896	0.844
275	0.962	0.894	0.838	0.899	0.848
276	0.971	0.876	0.847	0.898	0.846
277	0.960	0.886	0.853	0.900	0.849
278	0.962	0.884	0.847	0.898	0.846
279	0.968	0.889	0.841	0.900	0.849
280	0.968	0.891	0.847	0.903	0.853
281	0.974	0.873	0.835	0.894	0.841
282	0.968	0.879	0.844	0.897	0.845
283	0.974	0.879	0.847	0.900	0.849
284	0.962	0.881	0.841	0.895	0.842
285	0.968	0.894	0.829	0.898	0.847
286	0.965	0.868	0.853	0.895	0.842
287	0.962	0.881	0.838	0.894	0.841
288	0.974	0.884	0.844	0.901	0.851
289	0.971	0.889	0.841	0.901	0.851
290	0.971	0.876	0.850	0.899	0.848
291	0.974	0.884	0.850	0.903	0.854
292	0.965	0.884	0.835	0.895	0.842
293	0.965	0.891	0.853	0.903	0.855
294	0.971	0.897	0.847	0.905	0.858
295	0.968	0.868	0.838	0.891	0.837
296	0.971	0.891	0.841	0.902	0.852
297	0.971	0.886	0.850	0.903	0.854
298	0.971	0.881	0.841	0.898	0.846
299	0.962	0.881	0.844	0.896	0.844
300	0.965	0.881	0.847	0.898	0.846
301	0.974	0.884	0.853	0.903	0.855

302	0.974	0.891	0.847	0.904	0.856
303	0.960	0.889	0.829	0.893	0.839
304	0.968	0.876	0.847	0.897	0.845
305	0.968	0.879	0.841	0.896	0.844
306	0.965	0.884	0.829	0.893	0.839
307	0.962	0.902	0.844	0.903	0.855
308	0.977	0.876	0.853	0.902	0.852
309	0.968	0.881	0.841	0.897	0.845
310	0.974	0.886	0.835	0.899	0.848
311	0.968	0.871	0.844	0.894	0.841
312	0.971	0.876	0.835	0.894	0.841
313	0.971	0.873	0.832	0.892	0.838
314	0.965	0.881	0.844	0.897	0.845
315	0.965	0.876	0.850	0.897	0.845
316	0.971	0.876	0.835	0.894	0.841
317	0.968	0.879	0.841	0.896	0.844
318	0.960	0.881	0.850	0.897	0.845
319	0.971	0.873	0.835	0.893	0.839
320	0.965	0.873	0.838	0.892	0.838
321	0.965	0.876	0.847	0.896	0.844
322	0.962	0.868	0.859	0.896	0.844
323	0.968	0.884	0.832	0.895	0.842
324	0.968	0.881	0.835	0.895	0.842
325	0.965	0.884	0.835	0.895	0.842
326	0.971	0.876	0.838	0.895	0.842
327	0.968	0.886	0.850	0.902	0.852
328	0.965	0.884	0.832	0.894	0.841
329	0.962	0.871	0.835	0.889	0.834
330	0.957	0.881	0.841	0.893	0.839
331	0.968	0.879	0.835	0.894	0.841
332	0.968	0.886	0.847	0.901	0.851
333	0.962	0.897	0.838	0.900	0.849
334	0.974	0.884	0.841	0.900	0.849
335	0.965	0.881	0.859	0.902	0.852
336	0.971	0.889	0.844	0.902	0.852
337	0.965	0.879	0.844	0.896	0.844
338	0.968	0.886	0.841	0.899	0.848
339	0.971	0.894	0.829	0.899	0.848
340	0.965	0.876	0.835	0.892	0.838
341	0.960	0.879	0.844	0.894	0.841
342	0.965	0.884	0.826	0.892	0.838
343	0.962	0.889	0.841	0.898	0.846

344	0.977	0.876	0.847	0.900	0.849
345	0.965	0.873	0.838	0.892	0.838
346	0.971	0.879	0.844	0.898	0.846
347	0.968	0.871	0.844	0.894	0.841
348	0.965	0.881	0.841	0.896	0.844
349	0.968	0.881	0.838	0.896	0.844
350	0.962	0.876	0.844	0.894	0.841
351	0.971	0.881	0.832	0.895	0.842
352	0.965	0.889	0.835	0.897	0.845
353	0.965	0.879	0.847	0.897	0.845
354	0.965	0.894	0.847	0.903	0.853
355	0.962	0.884	0.838	0.895	0.842
356	0.960	0.879	0.853	0.897	0.845
357	0.965	0.876	0.841	0.894	0.841
358	0.971	0.879	0.835	0.895	0.842
359	0.977	0.881	0.850	0.903	0.854
360	0.971	0.879	0.844	0.898	0.846
361	0.962	0.894	0.850	0.903	0.853
362	0.962	0.884	0.847	0.898	0.846
363	0.965	0.889	0.844	0.900	0.849
364	0.962	0.881	0.832	0.892	0.838
365	0.971	0.886	0.841	0.900	0.849
366	0.965	0.884	0.847	0.899	0.848
367	0.971	0.881	0.850	0.901	0.851
368	0.968	0.889	0.835	0.898	0.846
369	0.960	0.889	0.832	0.894	0.841
370	0.971	0.881	0.835	0.896	0.844
371	0.965	0.886	0.832	0.895	0.842
372	0.971	0.866	0.841	0.892	0.838
373	0.971	0.871	0.841	0.894	0.841
374	0.968	0.881	0.844	0.898	0.846
375	0.965	0.884	0.844	0.898	0.846
376	0.977	0.879	0.850	0.902	0.852
377	0.962	0.879	0.844	0.895	0.842
378	0.968	0.884	0.847	0.900	0.849
379	0.968	0.873	0.832	0.891	0.837
380	0.962	0.873	0.838	0.891	0.837
381	0.971	0.876	0.850	0.899	0.848
382	0.965	0.881	0.847	0.898	0.846
383	0.965	0.884	0.838	0.896	0.844
384	0.974	0.886	0.835	0.899	0.848
385	0.965	0.884	0.838	0.896	0.844

386	0.974	0.894	0.841	0.903	0.855
387	0.971	0.879	0.838	0.896	0.844
388	0.965	0.884	0.844	0.898	0.846
389	0.974	0.884	0.850	0.903	0.854
390	0.965	0.879	0.850	0.898	0.846
391	0.962	0.879	0.850	0.897	0.845
392	0.968	0.879	0.847	0.898	0.846
393	0.965	0.879	0.841	0.895	0.842
394	0.965	0.873	0.844	0.894	0.841
395	0.968	0.879	0.853	0.900	0.849
396	0.965	0.886	0.841	0.898	0.846
397	0.971	0.884	0.838	0.898	0.846
398	0.965	0.889	0.841	0.899	0.848
399	0.974	0.886	0.838	0.900	0.849
400	0.971	0.879	0.841	0.897	0.845
401	0.971	0.886	0.838	0.899	0.848
402	0.965	0.884	0.835	0.895	0.842
403	0.968	0.889	0.856	0.904	0.856
404	0.962	0.871	0.856	0.896	0.844
405	0.957	0.884	0.835	0.892	0.838
406	0.971	0.881	0.838	0.897	0.845
407	0.974	0.889	0.850	0.904	0.856
408	0.954	0.860	0.853	0.888	0.833
409	0.971	0.881	0.844	0.899	0.848
410	0.968	0.879	0.844	0.897	0.845
411	0.962	0.886	0.847	0.899	0.848
412	0.965	0.876	0.850	0.897	0.845
413	0.971	0.876	0.847	0.898	0.846
414	0.977	0.876	0.847	0.900	0.849
415	0.968	0.879	0.859	0.902	0.852
416	0.965	0.879	0.847	0.897	0.845
417	0.962	0.871	0.841	0.891	0.837
418	0.965	0.884	0.832	0.894	0.841
419	0.965	0.868	0.853	0.895	0.842
420	0.968	0.873	0.850	0.897	0.845
421	0.974	0.876	0.856	0.902	0.852
422	0.968	0.866	0.844	0.892	0.838
423	0.965	0.868	0.841	0.891	0.837
424	0.968	0.871	0.847	0.895	0.842
425	0.957	0.876	0.829	0.888	0.831
426	0.971	0.884	0.850	0.902	0.852
427	0.968	0.884	0.838	0.897	0.845

428	0.971	0.876	0.832	0.893	0.839
429	0.968	0.889	0.844	0.901	0.851
430	0.968	0.884	0.847	0.900	0.849
431	0.968	0.873	0.832	0.891	0.837
432	0.968	0.876	0.838	0.894	0.841
433	0.971	0.868	0.844	0.894	0.841
434	0.965	0.873	0.841	0.893	0.839
435	0.962	0.879	0.841	0.894	0.841
436	0.968	0.881	0.844	0.898	0.846
437	0.968	0.881	0.850	0.900	0.849
438	0.960	0.879	0.832	0.890	0.835
439	0.965	0.884	0.856	0.902	0.852
440	0.974	0.873	0.844	0.897	0.845
441	0.957	0.884	0.832	0.891	0.837
442	0.962	0.889	0.850	0.901	0.851
443	0.957	0.876	0.856	0.896	0.844
444	0.965	0.881	0.838	0.895	0.842
445	0.965	0.881	0.844	0.897	0.845
446	0.977	0.889	0.835	0.901	0.851
447	0.965	0.873	0.835	0.891	0.837
448	0.965	0.886	0.853	0.902	0.852
449	0.971	0.873	0.850	0.898	0.847
450	0.962	0.886	0.838	0.896	0.844
451	0.965	0.881	0.850	0.899	0.848
452	0.965	0.879	0.847	0.897	0.845
453	0.960	0.873	0.859	0.897	0.845
454	0.957	0.881	0.838	0.892	0.838
455	0.971	0.871	0.832	0.891	0.837
456	0.971	0.886	0.841	0.900	0.849
457	0.968	0.881	0.832	0.894	0.841
458	0.960	0.889	0.838	0.896	0.844
459	0.965	0.876	0.856	0.899	0.848
460	0.971	0.873	0.841	0.895	0.842
461	0.962	0.866	0.838	0.888	0.832
462	0.954	0.894	0.841	0.897	0.845
463	0.968	0.873	0.859	0.900	0.849
464	0.971	0.881	0.835	0.896	0.844
465	0.965	0.876	0.856	0.899	0.848
466	0.971	0.868	0.850	0.896	0.844
467	0.974	0.884	0.853	0.903	0.855
468	0.962	0.881	0.853	0.899	0.848
469	0.968	0.881	0.850	0.900	0.849

470	0.971	0.876	0.844	0.897	0.845
471	0.968	0.876	0.847	0.897	0.845
472	0.962	0.881	0.856	0.900	0.849
473	0.962	0.879	0.847	0.896	0.844
474	0.962	0.879	0.844	0.895	0.842
475	0.962	0.881	0.859	0.901	0.851
476	0.968	0.886	0.838	0.898	0.846
477	0.962	0.871	0.856	0.896	0.844
478	0.968	0.871	0.847	0.895	0.842
479	0.962	0.876	0.862	0.900	0.849
480	0.968	0.886	0.847	0.901	0.851
481	0.968	0.886	0.853	0.903	0.854
482	0.968	0.871	0.844	0.894	0.841
483	0.971	0.873	0.844	0.896	0.844
484	0.962	0.873	0.832	0.889	0.834
485	0.968	0.889	0.853	0.903	0.855
486	0.962	0.868	0.844	0.891	0.837
487	0.962	0.879	0.847	0.896	0.844
488	0.971	0.873	0.835	0.893	0.839
489	0.962	0.889	0.838	0.897	0.845
490	0.977	0.884	0.838	0.900	0.849
491	0.962	0.873	0.850	0.895	0.842
492	0.960	0.876	0.850	0.895	0.842
493	0.971	0.879	0.850	0.900	0.849
494	0.968	0.881	0.862	0.903	0.855
495	0.962	0.884	0.853	0.900	0.849
496	0.965	0.876	0.844	0.895	0.842
497	0.971	0.876	0.865	0.903	0.855
498	0.971	0.879	0.859	0.903	0.854
499	0.971	0.873	0.844	0.896	0.844
500	0.965	0.879	0.844	0.896	0.844
501	0.965	0.884	0.841	0.897	0.845
502	0.974	0.876	0.850	0.900	0.849
503	0.965	0.873	0.838	0.892	0.838
504	0.962	0.881	0.853	0.899	0.848
505	0.965	0.884	0.853	0.901	0.851
506	0.974	0.876	0.853	0.901	0.851
507	0.962	0.881	0.838	0.894	0.841
508	0.965	0.858	0.838	0.887	0.830
509	0.962	0.868	0.835	0.888	0.832
510	0.968	0.876	0.847	0.897	0.845
511	0.974	0.876	0.844	0.898	0.847

512	0.962	0.881	0.841	0.895	0.842
513	0.965	0.873	0.862	0.900	0.849
514	0.962	0.873	0.847	0.894	0.841
515	0.965	0.866	0.841	0.890	0.835
516	0.974	0.884	0.850	0.903	0.853
517	0.971	0.860	0.850	0.893	0.840
518	0.971	0.876	0.844	0.897	0.845
519	0.971	0.873	0.838	0.894	0.841
520	0.971	0.873	0.859	0.901	0.851
521	0.957	0.871	0.859	0.895	0.842
522	0.968	0.884	0.850	0.901	0.851
523	0.965	0.860	0.841	0.888	0.832
524	0.962	0.868	0.856	0.895	0.842
525	0.960	0.860	0.841	0.887	0.830
526	0.962	0.868	0.853	0.894	0.841
527	0.965	0.871	0.853	0.896	0.844
528	0.968	0.866	0.847	0.893	0.839
529	0.971	0.873	0.847	0.897	0.845
530	0.965	0.884	0.838	0.896	0.844
531	0.971	0.873	0.859	0.901	0.851
532	0.962	0.873	0.847	0.894	0.841
533	0.968	0.868	0.850	0.895	0.842
534	0.960	0.871	0.844	0.891	0.837
535	0.968	0.884	0.847	0.900	0.849
536	0.962	0.881	0.841	0.895	0.842
537	0.968	0.866	0.841	0.891	0.837
538	0.965	0.891	0.850	0.903	0.853
539	0.960	0.879	0.841	0.893	0.839
540	0.968	0.871	0.871	0.903	0.854
541	0.971	0.876	0.832	0.893	0.839
542	0.962	0.881	0.832	0.892	0.838
543	0.971	0.873	0.841	0.895	0.842
544	0.962	0.876	0.847	0.895	0.842
545	0.974	0.881	0.853	0.903	0.854
546	0.968	0.876	0.844	0.896	0.844
547	0.968	0.866	0.844	0.892	0.838
548	0.962	0.873	0.856	0.897	0.845
549	0.971	0.868	0.838	0.892	0.838
550	0.962	0.879	0.853	0.898	0.846
551	0.962	0.876	0.838	0.892	0.838
552	0.962	0.881	0.844	0.896	0.844
553	0.962	0.881	0.841	0.895	0.842

554	0.974	0.879	0.844	0.899	0.848
555	0.968	0.873	0.853	0.898	0.846
556	0.957	0.881	0.841	0.893	0.839
557	0.968	0.876	0.844	0.896	0.844
558	0.968	0.868	0.856	0.897	0.845
559	0.968	0.879	0.850	0.899	0.848
560	0.965	0.881	0.835	0.894	0.841
561	0.965	0.879	0.832	0.892	0.838
562	0.974	0.868	0.832	0.891	0.837
563	0.968	0.884	0.865	0.905	0.858
564	0.962	0.868	0.850	0.893	0.840
565	0.962	0.866	0.856	0.894	0.841
566	0.974	0.879	0.844	0.899	0.848
567	0.968	0.876	0.844	0.896	0.844
568	0.965	0.868	0.862	0.898	0.847
569	0.971	0.879	0.832	0.894	0.841
570	0.957	0.866	0.841	0.888	0.831
571	0.965	0.866	0.832	0.888	0.831
572	0.980	0.873	0.853	0.902	0.852
573	0.965	0.876	0.844	0.895	0.842
574	0.965	0.879	0.850	0.898	0.846
575	0.965	0.876	0.859	0.900	0.849
576	0.968	0.879	0.847	0.898	0.846
577	0.971	0.884	0.856	0.903	0.855
578	0.974	0.871	0.844	0.896	0.844
579	0.974	0.897	0.838	0.903	0.855
580	0.971	0.886	0.859	0.905	0.858
581	0.974	0.884	0.847	0.902	0.852
582	0.965	0.873	0.847	0.895	0.842
583	0.968	0.886	0.856	0.903	0.855
584	0.957	0.886	0.844	0.896	0.844
585	0.971	0.881	0.835	0.896	0.844
586	0.968	0.868	0.838	0.891	0.837
587	0.965	0.879	0.853	0.899	0.848
588	0.962	0.876	0.850	0.896	0.844
589	0.968	0.868	0.856	0.897	0.845
590	0.965	0.866	0.832	0.888	0.831
591	0.980	0.876	0.844	0.900	0.849
592	0.968	0.886	0.847	0.901	0.851
593	0.977	0.886	0.835	0.900	0.849
594	0.960	0.884	0.832	0.892	0.838
595	0.974	0.889	0.844	0.903	0.854

596	0.965	0.873	0.850	0.896	0.844
597	0.968	0.868	0.856	0.897	0.845
598	0.965	0.871	0.841	0.892	0.838
599	0.974	0.879	0.844	0.899	0.848
600	0.977	0.855	0.856	0.895	0.842
601	0.960	0.884	0.841	0.895	0.842
602	0.965	0.873	0.847	0.895	0.842
603	0.971	0.876	0.850	0.899	0.848
604	0.974	0.881	0.832	0.896	0.844
605	0.968	0.871	0.838	0.892	0.838
606	0.971	0.881	0.841	0.898	0.846
607	0.968	0.879	0.850	0.899	0.848
608	0.968	0.884	0.832	0.895	0.842
609	0.974	0.863	0.847	0.894	0.841
610	0.968	0.868	0.844	0.893	0.839
611	0.965	0.871	0.835	0.890	0.835
612	0.962	0.863	0.835	0.887	0.830
613	0.971	0.876	0.835	0.894	0.841
614	0.965	0.894	0.847	0.903	0.853
615	0.965	0.886	0.850	0.901	0.851
616	0.968	0.873	0.844	0.895	0.842
617	0.977	0.879	0.844	0.900	0.849
618	0.971	0.868	0.847	0.895	0.842
619	0.962	0.868	0.847	0.892	0.838
620	0.971	0.873	0.844	0.896	0.844
621	0.962	0.868	0.859	0.896	0.844
622	0.968	0.873	0.838	0.893	0.839
623	0.962	0.863	0.838	0.888	0.831
624	0.971	0.868	0.838	0.892	0.838
625	0.971	0.866	0.835	0.890	0.835
626	0.962	0.879	0.853	0.898	0.846
627	0.968	0.879	0.844	0.897	0.845
628	0.962	0.873	0.850	0.895	0.842
629	0.965	0.871	0.832	0.889	0.834
630	0.965	0.871	0.841	0.892	0.838
631	0.965	0.866	0.847	0.892	0.838
632	0.965	0.876	0.847	0.896	0.844
633	0.962	0.863	0.856	0.893	0.840
634	0.971	0.863	0.829	0.888	0.831
635	0.965	0.881	0.847	0.898	0.846
636	0.968	0.873	0.841	0.894	0.841
637	0.962	0.876	0.850	0.896	0.844

638	0.962	0.855	0.862	0.892	0.838
639	0.965	0.873	0.841	0.893	0.839
640	0.971	0.866	0.832	0.889	0.834
641	0.968	0.879	0.832	0.893	0.839
642	0.962	0.863	0.835	0.887	0.830
643	0.971	0.881	0.844	0.899	0.848
644	0.960	0.876	0.859	0.898	0.847
645	0.971	0.889	0.838	0.900	0.849
646	0.971	0.866	0.850	0.895	0.842
647	0.962	0.873	0.844	0.893	0.839
648	0.968	0.871	0.859	0.899	0.848
649	0.962	0.881	0.841	0.895	0.842
650	0.974	0.866	0.847	0.895	0.842
651	0.971	0.863	0.844	0.892	0.838
652	0.974	0.876	0.835	0.895	0.842
653	0.960	0.873	0.850	0.894	0.841
654	0.965	0.886	0.847	0.900	0.849
655	0.957	0.879	0.856	0.897	0.845
656	0.962	0.873	0.832	0.889	0.834
657	0.965	0.879	0.829	0.891	0.837
658	0.965	0.879	0.853	0.899	0.848
659	0.957	0.873	0.844	0.891	0.837
660	0.968	0.866	0.841	0.891	0.837
661	0.971	0.879	0.832	0.894	0.841
662	0.968	0.881	0.841	0.897	0.845
663	0.962	0.873	0.847	0.894	0.841
664	0.960	0.871	0.847	0.892	0.838
665	0.968	0.873	0.850	0.897	0.845
666	0.968	0.868	0.838	0.891	0.837
667	0.965	0.866	0.832	0.888	0.831
668	0.968	0.879	0.853	0.900	0.849
669	0.971	0.873	0.847	0.897	0.845
670	0.974	0.873	0.850	0.899	0.848
671	0.974	0.876	0.832	0.894	0.841
672	0.968	0.866	0.853	0.895	0.842
673	0.977	0.876	0.841	0.898	0.846
674	0.971	0.876	0.847	0.898	0.846
675	0.971	0.884	0.844	0.900	0.849
676	0.971	0.886	0.829	0.896	0.844
677	0.962	0.871	0.844	0.892	0.838
678	0.974	0.871	0.832	0.892	0.838
679	0.968	0.879	0.853	0.900	0.849

680	0.962	0.876	0.847	0.895	0.842
681	0.962	0.873	0.850	0.895	0.842
682	0.965	0.876	0.844	0.895	0.842
683	0.965	0.866	0.823	0.885	0.827
684	0.962	0.881	0.844	0.896	0.844
685	0.971	0.873	0.826	0.890	0.835
686	0.968	0.868	0.838	0.891	0.837
687	0.962	0.881	0.841	0.895	0.842
688	0.974	0.873	0.859	0.902	0.852
689	0.960	0.873	0.844	0.892	0.838
690	0.962	0.879	0.832	0.891	0.837
691	0.971	0.873	0.856	0.900	0.849
692	0.965	0.858	0.838	0.887	0.830
693	0.971	0.876	0.874	0.906	0.859
694	0.965	0.876	0.859	0.900	0.849
695	0.974	0.881	0.838	0.898	0.846
696	0.965	0.881	0.838	0.895	0.842
697	0.968	0.876	0.853	0.899	0.848
698	0.954	0.871	0.844	0.889	0.834
699	0.960	0.858	0.853	0.889	0.834
700	0.965	0.860	0.856	0.893	0.840
701	0.962	0.876	0.841	0.893	0.839
702	0.968	0.889	0.847	0.902	0.852
703	0.960	0.884	0.847	0.897	0.845
704	0.962	0.871	0.859	0.897	0.845
705	0.960	0.871	0.862	0.897	0.845
706	0.971	0.868	0.847	0.895	0.842
707	0.974	0.873	0.847	0.898	0.847
708	0.968	0.876	0.844	0.896	0.844
709	0.965	0.868	0.847	0.893	0.839
710	0.965	0.884	0.844	0.898	0.846
711	0.960	0.876	0.838	0.891	0.837
712	0.965	0.881	0.859	0.902	0.852
713	0.962	0.853	0.847	0.887	0.830
714	0.960	0.876	0.847	0.894	0.841
715	0.960	0.876	0.832	0.889	0.834
716	0.971	0.876	0.838	0.895	0.842
717	0.965	0.876	0.847	0.896	0.844
718	0.962	0.873	0.835	0.890	0.835
719	0.960	0.879	0.844	0.894	0.841
720	0.957	0.886	0.835	0.893	0.839
721	0.960	0.863	0.853	0.891	0.837

722	0.965	0.868	0.847	0.893	0.839
723	0.965	0.868	0.829	0.888	0.831
724	0.968	0.873	0.847	0.896	0.844
725	0.968	0.871	0.850	0.896	0.844
726	0.960	0.868	0.847	0.891	0.837
727	0.960	0.868	0.844	0.890	0.835
728	0.971	0.868	0.820	0.887	0.830
729	0.962	0.881	0.832	0.892	0.838
730	0.962	0.879	0.835	0.892	0.838
731	0.960	0.866	0.835	0.887	0.830
732	0.957	0.881	0.829	0.889	0.834
733	0.960	0.884	0.829	0.891	0.837
734	0.960	0.889	0.841	0.897	0.845
735	0.968	0.881	0.829	0.893	0.839
736	0.962	0.879	0.850	0.897	0.845
737	0.968	0.873	0.844	0.895	0.842
738	0.965	0.879	0.844	0.896	0.844
739	0.965	0.871	0.847	0.894	0.841
740	0.965	0.863	0.847	0.891	0.837
741	0.974	0.863	0.844	0.893	0.839
742	0.971	0.876	0.832	0.893	0.839
743	0.965	0.873	0.838	0.892	0.838
744	0.968	0.876	0.844	0.896	0.844
745	0.965	0.855	0.835	0.885	0.827
746	0.971	0.873	0.853	0.899	0.848
747	0.971	0.884	0.832	0.896	0.844
748	0.957	0.886	0.844	0.896	0.844
749	0.962	0.873	0.844	0.893	0.839
750	0.974	0.873	0.829	0.892	0.838
751	0.960	0.871	0.823	0.885	0.827
752	0.965	0.879	0.838	0.894	0.841
753	0.954	0.863	0.826	0.881	0.821
754	0.968	0.879	0.838	0.895	0.842
755	0.962	0.871	0.835	0.889	0.834
756	0.968	0.873	0.832	0.891	0.837
757	0.968	0.871	0.835	0.891	0.837
758	0.971	0.881	0.844	0.899	0.848
759	0.968	0.876	0.847	0.897	0.845
760	0.960	0.876	0.841	0.892	0.838
761	0.971	0.871	0.835	0.892	0.838
762	0.962	0.884	0.820	0.889	0.834
763	0.965	0.863	0.844	0.890	0.835

764	0.965	0.881	0.841	0.896	0.844
765	0.968	0.871	0.835	0.891	0.837
766	0.974	0.873	0.832	0.893	0.839
767	0.957	0.873	0.838	0.889	0.834
768	0.965	0.871	0.829	0.888	0.832
769	0.960	0.873	0.835	0.889	0.834
770	0.971	0.866	0.841	0.892	0.838
771	0.962	0.871	0.847	0.893	0.839
772	0.957	0.881	0.835	0.891	0.837
773	0.977	0.866	0.844	0.895	0.842
774	0.971	0.871	0.835	0.892	0.838
775	0.971	0.863	0.832	0.888	0.832
776	0.968	0.881	0.844	0.898	0.846
777	0.971	0.863	0.826	0.887	0.830
778	0.971	0.873	0.838	0.894	0.841
779	0.968	0.868	0.838	0.891	0.837
780	0.974	0.881	0.832	0.896	0.844
781	0.971	0.886	0.835	0.898	0.846
782	0.968	0.879	0.835	0.894	0.841
783	0.965	0.866	0.826	0.886	0.828
784	0.971	0.868	0.838	0.892	0.838
785	0.962	0.881	0.841	0.895	0.842
786	0.968	0.863	0.826	0.886	0.828
787	0.965	0.868	0.844	0.892	0.838
788	0.960	0.868	0.850	0.892	0.838

**SHAP by lightGBM**					
**Numbers of features**	**G1**	**G2/M**	**S**	**ACC**	**MCC**
1	0.908	0.636	0.611	0.716	0.575
2	0.977	0.690	0.743	0.799	0.701
3	0.971	0.685	0.719	0.788	0.683
4	0.971	0.783	0.793	0.847	0.771
5	0.965	0.801	0.793	0.852	0.778
6	0.971	0.837	0.802	0.870	0.804
7	0.974	0.858	0.826	0.886	0.828
8	0.965	0.891	0.814	0.891	0.837
9	0.968	0.899	0.820	0.897	0.845
10	0.965	0.891	0.808	0.889	0.834
11	0.968	0.912	0.817	0.901	0.851
12	0.974	0.907	0.823	0.903	0.854
13	0.974	0.912	0.826	0.905	0.858
14	0.968	0.910	0.835	0.905	0.858
15	0.971	0.902	0.808	0.895	0.842

16	0.962	0.907	0.823	0.899	0.848
17	0.968	0.912	0.823	0.903	0.854
18	0.968	0.902	0.805	0.893	0.840
19	0.980	0.907	0.814	0.902	0.852
20	0.977	0.897	0.823	0.900	0.849
21	0.977	0.910	0.808	0.900	0.850
22	0.977	0.910	0.817	0.903	0.854
23	0.980	0.912	0.826	0.907	0.861
24	0.980	0.910	0.817	0.903	0.855
25	0.980	0.894	0.826	0.901	0.851
26	0.974	0.910	0.844	0.910	0.865
27	0.980	0.904	0.826	0.904	0.857
28	0.977	0.902	0.835	0.905	0.858
29	0.977	0.915	0.823	0.906	0.859
30	0.977	0.922	0.829	0.911	0.867
31	0.980	0.910	0.835	0.909	0.864
32	0.983	0.917	0.838	0.914	0.871
33	0.971	0.904	0.817	0.899	0.848
34	0.974	0.904	0.820	0.901	0.851
35	0.980	0.907	0.832	0.907	0.861
36	0.974	0.915	0.838	0.910	0.865
37	0.971	0.897	0.844	0.904	0.856
38	0.971	0.904	0.835	0.904	0.856
39	0.971	0.904	0.844	0.907	0.861
40	0.977	0.907	0.826	0.904	0.856
41	0.974	0.902	0.826	0.902	0.852
42	0.971	0.904	0.838	0.905	0.858
43	0.974	0.904	0.826	0.903	0.854
44	0.974	0.907	0.838	0.907	0.861
45	0.971	0.915	0.844	0.911	0.866
46	0.980	0.910	0.841	0.911	0.866
47	0.977	0.899	0.832	0.903	0.855
48	0.980	0.917	0.841	0.914	0.871
49	0.980	0.912	0.838	0.911	0.866
50	0.971	0.917	0.832	0.908	0.862
51	0.977	0.912	0.835	0.909	0.864
52	0.974	0.904	0.844	0.908	0.862
53	0.977	0.910	0.832	0.907	0.861
54	0.977	0.897	0.847	0.907	0.861
55	0.968	0.899	0.844	0.904	0.856
56	0.968	0.907	0.844	0.907	0.861
57	0.980	0.910	0.829	0.907	0.861

58	0.980	0.899	0.850	0.910	0.865
59	0.974	0.894	0.844	0.904	0.856
60	0.974	0.897	0.832	0.902	0.852
61	0.977	0.891	0.829	0.900	0.849
62	0.988	0.899	0.835	0.908	0.862
63	0.977	0.904	0.823	0.903	0.854
64	0.974	0.910	0.838	0.908	0.862
65	0.977	0.899	0.859	0.912	0.868
66	0.974	0.904	0.835	0.905	0.858
67	0.974	0.904	0.841	0.907	0.861
68	0.968	0.902	0.844	0.905	0.858
69	0.971	0.897	0.844	0.904	0.856
70	0.977	0.907	0.847	0.911	0.866
71	0.968	0.902	0.841	0.904	0.856
72	0.977	0.910	0.841	0.910	0.865
73	0.977	0.907	0.835	0.907	0.861
74	0.968	0.910	0.838	0.906	0.859
75	0.974	0.899	0.829	0.902	0.852
76	0.968	0.894	0.841	0.902	0.852
77	0.980	0.899	0.832	0.904	0.856
78	0.971	0.899	0.856	0.909	0.863
79	0.971	0.894	0.847	0.904	0.856
80	0.974	0.907	0.853	0.912	0.868
81	0.974	0.912	0.856	0.915	0.872
82	0.968	0.907	0.850	0.909	0.863
83	0.971	0.907	0.862	0.914	0.870
84	0.980	0.897	0.859	0.912	0.868
85	0.977	0.902	0.847	0.909	0.863
86	0.971	0.910	0.853	0.912	0.868
87	0.977	0.915	0.856	0.917	0.875
88	0.968	0.907	0.853	0.910	0.865
89	0.971	0.904	0.832	0.903	0.855
90	0.974	0.902	0.847	0.908	0.862
91	0.968	0.910	0.859	0.913	0.869
92	0.968	0.904	0.853	0.909	0.863
93	0.977	0.910	0.838	0.909	0.864
94	0.968	0.902	0.832	0.902	0.852
95	0.980	0.894	0.856	0.910	0.865
96	0.977	0.904	0.847	0.910	0.865
97	0.980	0.902	0.856	0.913	0.869
98	0.977	0.907	0.853	0.913	0.869
99	0.962	0.899	0.838	0.901	0.851

100	0.968	0.902	0.853	0.908	0.862
101	0.962	0.907	0.844	0.905	0.858
102	0.965	0.904	0.835	0.903	0.854
103	0.971	0.904	0.844	0.907	0.861
104	0.962	0.904	0.856	0.908	0.862
105	0.974	0.899	0.832	0.903	0.854
106	0.962	0.899	0.844	0.903	0.853
107	0.962	0.904	0.859	0.909	0.863
108	0.965	0.910	0.832	0.903	0.855
109	0.962	0.899	0.838	0.901	0.851
110	0.971	0.904	0.850	0.909	0.863
111	0.971	0.904	0.847	0.908	0.862
112	0.980	0.886	0.850	0.905	0.858
113	0.968	0.907	0.847	0.908	0.862
114	0.974	0.897	0.850	0.907	0.861
115	0.971	0.907	0.844	0.908	0.862
116	0.968	0.894	0.841	0.902	0.852
117	0.971	0.907	0.841	0.907	0.861
118	0.965	0.910	0.835	0.904	0.856
119	0.968	0.907	0.859	0.912	0.868
120	0.965	0.904	0.841	0.904	0.856
121	0.971	0.910	0.850	0.911	0.866
122	0.968	0.899	0.832	0.901	0.851
123	0.974	0.904	0.844	0.908	0.862
124	0.971	0.891	0.850	0.904	0.856
125	0.971	0.897	0.847	0.905	0.858
126	0.980	0.899	0.847	0.909	0.863
127	0.974	0.899	0.844	0.906	0.859
128	0.962	0.904	0.841	0.903	0.855
129	0.971	0.904	0.859	0.912	0.868
130	0.968	0.910	0.841	0.907	0.861
131	0.968	0.899	0.844	0.904	0.856
132	0.974	0.902	0.847	0.908	0.862
133	0.968	0.897	0.853	0.906	0.859
134	0.971	0.902	0.856	0.910	0.865
135	0.968	0.891	0.841	0.901	0.851
136	0.957	0.897	0.847	0.901	0.851
137	0.965	0.907	0.841	0.905	0.858
138	0.968	0.904	0.838	0.904	0.856
139	0.965	0.904	0.841	0.904	0.856
140	0.977	0.910	0.847	0.912	0.868
141	0.965	0.902	0.856	0.908	0.862

142	0.960	0.910	0.856	0.909	0.863
143	0.971	0.894	0.853	0.906	0.859
144	0.968	0.902	0.841	0.904	0.856
145	0.971	0.899	0.862	0.911	0.866
146	0.968	0.902	0.835	0.903	0.854
147	0.968	0.902	0.853	0.908	0.862
148	0.968	0.897	0.841	0.903	0.854
149	0.974	0.881	0.850	0.902	0.852
150	0.968	0.897	0.853	0.906	0.859
151	0.971	0.910	0.844	0.909	0.863
152	0.974	0.899	0.841	0.905	0.858
153	0.971	0.899	0.847	0.906	0.859
154	0.960	0.886	0.847	0.898	0.846
155	0.965	0.902	0.853	0.907	0.861
156	0.974	0.902	0.838	0.905	0.858
157	0.968	0.902	0.862	0.911	0.866
158	0.971	0.912	0.850	0.912	0.868
159	0.968	0.899	0.859	0.909	0.863
160	0.971	0.891	0.850	0.904	0.856
161	0.974	0.904	0.856	0.912	0.868
162	0.971	0.894	0.847	0.904	0.856
163	0.965	0.899	0.853	0.906	0.859
164	0.971	0.912	0.862	0.916	0.873
165	0.971	0.891	0.856	0.906	0.859
166	0.957	0.891	0.859	0.903	0.853
167	0.974	0.897	0.847	0.906	0.859
168	0.974	0.897	0.847	0.906	0.859
169	0.974	0.897	0.859	0.910	0.865
170	0.968	0.897	0.844	0.903	0.855
171	0.971	0.894	0.844	0.903	0.855
172	0.968	0.897	0.844	0.903	0.855
173	0.965	0.902	0.847	0.905	0.858
174	0.974	0.889	0.844	0.903	0.854
175	0.974	0.904	0.844	0.908	0.862
176	0.965	0.899	0.856	0.907	0.861
177	0.965	0.902	0.853	0.907	0.861
178	0.971	0.894	0.853	0.906	0.859
179	0.965	0.904	0.844	0.905	0.858
180	0.968	0.899	0.865	0.911	0.866
181	0.957	0.889	0.847	0.898	0.846
182	0.974	0.889	0.871	0.911	0.866
183	0.960	0.891	0.868	0.906	0.859

184	0.962	0.899	0.838	0.901	0.851
185	0.962	0.884	0.841	0.896	0.844
186	0.960	0.899	0.841	0.901	0.851
187	0.968	0.902	0.844	0.905	0.858
188	0.977	0.897	0.841	0.905	0.858
189	0.962	0.897	0.853	0.904	0.856
190	0.971	0.902	0.841	0.905	0.858
191	0.968	0.897	0.838	0.902	0.852
192	0.971	0.894	0.850	0.905	0.858
193	0.962	0.886	0.838	0.896	0.844
194	0.960	0.891	0.841	0.898	0.846
195	0.977	0.884	0.859	0.906	0.859
196	0.962	0.907	0.832	0.902	0.852
197	0.957	0.891	0.844	0.898	0.846
198	0.962	0.902	0.844	0.903	0.855
199	0.968	0.897	0.835	0.901	0.851
200	0.957	0.897	0.850	0.902	0.852
201	0.960	0.886	0.853	0.900	0.849
202	0.977	0.907	0.850	0.912	0.868
203	0.965	0.894	0.844	0.902	0.852
204	0.971	0.894	0.835	0.901	0.851
205	0.968	0.897	0.844	0.903	0.855
206	0.971	0.894	0.847	0.904	0.856
207	0.968	0.897	0.838	0.902	0.852
208	0.974	0.889	0.835	0.900	0.849
209	0.968	0.894	0.853	0.905	0.858
210	0.971	0.897	0.841	0.903	0.855
211	0.968	0.894	0.841	0.902	0.852
212	0.965	0.873	0.850	0.896	0.844
213	0.960	0.899	0.841	0.901	0.851
214	0.968	0.889	0.853	0.903	0.855
215	0.983	0.894	0.847	0.908	0.862
216	0.971	0.894	0.844	0.903	0.855
217	0.962	0.899	0.850	0.904	0.856
218	0.971	0.891	0.844	0.903	0.854
219	0.960	0.891	0.847	0.900	0.849
220	0.968	0.889	0.841	0.900	0.849
221	0.977	0.902	0.844	0.908	0.862
222	0.962	0.891	0.847	0.901	0.851
223	0.965	0.894	0.850	0.903	0.855
224	0.965	0.897	0.850	0.904	0.856
225	0.968	0.897	0.844	0.903	0.855

226	0.962	0.891	0.850	0.902	0.852
227	0.971	0.897	0.844	0.904	0.856
228	0.974	0.899	0.829	0.902	0.852
229	0.971	0.889	0.835	0.899	0.848
230	0.957	0.894	0.862	0.904	0.856
231	0.974	0.891	0.850	0.905	0.858
232	0.971	0.886	0.841	0.900	0.849
233	0.977	0.884	0.847	0.903	0.854
234	0.968	0.889	0.853	0.903	0.855
235	0.977	0.881	0.853	0.903	0.855
236	0.968	0.894	0.853	0.905	0.858
237	0.968	0.897	0.850	0.905	0.858
238	0.971	0.894	0.856	0.907	0.861
239	0.968	0.886	0.856	0.903	0.855
240	0.971	0.891	0.841	0.902	0.852
241	0.962	0.894	0.850	0.903	0.853
242	0.968	0.886	0.844	0.900	0.849
243	0.974	0.889	0.838	0.901	0.851
244	0.974	0.876	0.844	0.898	0.847
245	0.962	0.884	0.853	0.900	0.849
246	0.968	0.891	0.853	0.904	0.856
247	0.968	0.899	0.856	0.908	0.862
248	0.962	0.891	0.850	0.902	0.852
249	0.968	0.899	0.859	0.909	0.863
250	0.974	0.889	0.856	0.906	0.859
251	0.968	0.902	0.862	0.911	0.866
252	0.962	0.884	0.862	0.903	0.854
253	0.965	0.897	0.847	0.903	0.855
254	0.957	0.891	0.862	0.903	0.855
255	0.971	0.884	0.832	0.896	0.844
256	0.968	0.894	0.868	0.910	0.865
257	0.977	0.889	0.838	0.902	0.852
258	0.968	0.879	0.847	0.898	0.847
259	0.962	0.904	0.853	0.907	0.861
260	0.965	0.886	0.862	0.904	0.856
261	0.965	0.889	0.862	0.905	0.858
262	0.971	0.899	0.853	0.908	0.862
263	0.960	0.897	0.850	0.903	0.853
264	0.971	0.889	0.859	0.906	0.859
265	0.968	0.910	0.850	0.910	0.865
266	0.962	0.889	0.847	0.900	0.849
267	0.971	0.897	0.859	0.909	0.863

268	0.968	0.884	0.838	0.897	0.845
269	0.962	0.884	0.847	0.898	0.846
270	0.962	0.897	0.844	0.902	0.852
271	0.971	0.897	0.859	0.909	0.863
272	0.965	0.891	0.859	0.905	0.858
273	0.977	0.881	0.856	0.904	0.856
274	0.971	0.894	0.859	0.908	0.862
275	0.962	0.894	0.841	0.900	0.849
276	0.960	0.894	0.844	0.900	0.849
277	0.951	0.884	0.847	0.894	0.841
278	0.974	0.886	0.847	0.903	0.854
279	0.968	0.902	0.847	0.906	0.859
280	0.960	0.886	0.856	0.901	0.851
281	0.965	0.891	0.832	0.897	0.845
282	0.968	0.891	0.844	0.902	0.852
283	0.974	0.894	0.841	0.903	0.855
284	0.960	0.879	0.844	0.894	0.841
285	0.977	0.894	0.832	0.902	0.852
286	0.965	0.891	0.844	0.901	0.851
287	0.974	0.904	0.844	0.908	0.862
288	0.974	0.891	0.847	0.904	0.856
289	0.962	0.889	0.853	0.902	0.852
290	0.968	0.897	0.862	0.909	0.863
291	0.960	0.884	0.844	0.896	0.844
292	0.960	0.873	0.847	0.893	0.839
293	0.965	0.899	0.844	0.903	0.855
294	0.962	0.899	0.847	0.903	0.855
295	0.962	0.897	0.838	0.900	0.849
296	0.971	0.884	0.844	0.900	0.849
297	0.968	0.886	0.847	0.901	0.851
298	0.962	0.889	0.856	0.903	0.853
299	0.957	0.889	0.844	0.897	0.845
300	0.968	0.891	0.838	0.900	0.849
301	0.968	0.881	0.856	0.902	0.852
302	0.962	0.886	0.853	0.901	0.851
303	0.971	0.897	0.856	0.908	0.862
304	0.960	0.881	0.850	0.897	0.845
305	0.974	0.891	0.841	0.903	0.854
306	0.954	0.876	0.844	0.891	0.837
307	0.965	0.894	0.850	0.903	0.855
308	0.965	0.899	0.856	0.907	0.861
309	0.968	0.889	0.859	0.905	0.858

310	0.968	0.904	0.847	0.907	0.861
311	0.960	0.889	0.841	0.897	0.845
312	0.965	0.886	0.838	0.897	0.845
313	0.962	0.889	0.847	0.900	0.849
314	0.968	0.889	0.847	0.902	0.852
315	0.971	0.886	0.838	0.899	0.848
316	0.968	0.881	0.841	0.897	0.845
317	0.968	0.884	0.850	0.901	0.851
318	0.968	0.881	0.844	0.898	0.846
319	0.974	0.897	0.841	0.904	0.856
320	0.968	0.889	0.841	0.900	0.849
321	0.971	0.897	0.865	0.911	0.866
322	0.965	0.899	0.850	0.905	0.858
323	0.971	0.894	0.847	0.904	0.856
324	0.965	0.894	0.862	0.907	0.861
325	0.965	0.902	0.850	0.906	0.859
326	0.971	0.902	0.841	0.905	0.858
327	0.968	0.873	0.853	0.898	0.847
328	0.968	0.889	0.850	0.903	0.853
329	0.962	0.886	0.847	0.899	0.848
330	0.968	0.884	0.847	0.900	0.849
331	0.965	0.897	0.844	0.903	0.854
332	0.965	0.891	0.859	0.905	0.858
333	0.968	0.881	0.850	0.900	0.849
334	0.965	0.884	0.853	0.901	0.851
335	0.960	0.891	0.844	0.899	0.848
336	0.968	0.884	0.847	0.900	0.849
337	0.971	0.891	0.853	0.905	0.858
338	0.968	0.889	0.856	0.904	0.856
339	0.968	0.881	0.862	0.903	0.855
340	0.971	0.886	0.844	0.901	0.851
341	0.960	0.886	0.844	0.897	0.845
342	0.965	0.894	0.844	0.902	0.852
343	0.968	0.894	0.847	0.903	0.855
344	0.962	0.876	0.856	0.898	0.846
345	0.968	0.886	0.856	0.903	0.855
346	0.962	0.889	0.853	0.902	0.852
347	0.971	0.889	0.865	0.908	0.862
348	0.968	0.884	0.847	0.900	0.849
349	0.974	0.902	0.853	0.910	0.865
350	0.968	0.884	0.853	0.902	0.852
351	0.968	0.876	0.838	0.894	0.841

352	0.971	0.889	0.853	0.904	0.856
353	0.954	0.876	0.847	0.892	0.838
354	0.965	0.889	0.856	0.903	0.855
355	0.968	0.884	0.847	0.900	0.849
356	0.968	0.884	0.862	0.904	0.856
357	0.960	0.894	0.841	0.899	0.848
358	0.974	0.881	0.865	0.906	0.859
359	0.962	0.891	0.838	0.898	0.846
360	0.962	0.899	0.838	0.901	0.851
361	0.968	0.899	0.862	0.910	0.865
362	0.965	0.879	0.853	0.899	0.848
363	0.957	0.894	0.850	0.901	0.851
364	0.971	0.886	0.853	0.903	0.855
365	0.968	0.894	0.847	0.903	0.855
366	0.965	0.897	0.853	0.905	0.858
367	0.965	0.897	0.859	0.907	0.861
368	0.965	0.884	0.847	0.899	0.848
369	0.971	0.902	0.841	0.905	0.858
370	0.962	0.884	0.862	0.903	0.853
371	0.971	0.884	0.847	0.901	0.851
372	0.962	0.886	0.841	0.897	0.845
373	0.971	0.897	0.835	0.902	0.852
374	0.962	0.886	0.841	0.897	0.845
375	0.971	0.897	0.850	0.906	0.859
376	0.968	0.891	0.847	0.903	0.853
377	0.965	0.902	0.841	0.903	0.855
378	0.974	0.894	0.859	0.909	0.863
379	0.962	0.884	0.841	0.896	0.844
380	0.971	0.886	0.844	0.901	0.851
381	0.974	0.873	0.856	0.901	0.851
382	0.962	0.894	0.835	0.898	0.846
383	0.965	0.889	0.853	0.903	0.853
384	0.968	0.889	0.856	0.904	0.856
385	0.971	0.894	0.847	0.904	0.856
386	0.974	0.897	0.850	0.907	0.861
387	0.957	0.881	0.850	0.896	0.844
388	0.957	0.889	0.850	0.899	0.848
389	0.968	0.891	0.847	0.903	0.853
390	0.960	0.876	0.841	0.892	0.838
391	0.962	0.889	0.850	0.901	0.851
392	0.965	0.876	0.850	0.897	0.845
393	0.968	0.884	0.853	0.902	0.852

394	0.962	0.879	0.838	0.893	0.839
395	0.968	0.884	0.862	0.904	0.856
396	0.968	0.876	0.841	0.895	0.842
397	0.965	0.889	0.847	0.901	0.851
398	0.957	0.889	0.850	0.899	0.848
399	0.977	0.897	0.847	0.907	0.861
400	0.968	0.879	0.844	0.897	0.845
401	0.965	0.881	0.850	0.899	0.848
402	0.971	0.876	0.838	0.895	0.842
403	0.971	0.891	0.835	0.900	0.849
404	0.971	0.871	0.844	0.895	0.842
405	0.977	0.886	0.856	0.906	0.859
406	0.968	0.897	0.850	0.905	0.858
407	0.965	0.884	0.847	0.899	0.848
408	0.965	0.889	0.844	0.900	0.849
409	0.962	0.897	0.865	0.908	0.862
410	0.968	0.891	0.835	0.899	0.848
411	0.968	0.889	0.847	0.902	0.852
412	0.962	0.891	0.853	0.903	0.853
413	0.965	0.889	0.847	0.901	0.851
414	0.965	0.894	0.850	0.903	0.855
415	0.965	0.876	0.838	0.893	0.839
416	0.962	0.886	0.853	0.901	0.851
417	0.968	0.891	0.835	0.899	0.848
418	0.974	0.881	0.835	0.897	0.845
419	0.962	0.904	0.844	0.904	0.856
420	0.965	0.873	0.853	0.897	0.845
421	0.962	0.889	0.847	0.900	0.849
422	0.960	0.871	0.844	0.891	0.837
423	0.968	0.897	0.841	0.903	0.854
424	0.968	0.873	0.853	0.898	0.846
425	0.962	0.897	0.844	0.902	0.852
426	0.960	0.891	0.844	0.899	0.848
427	0.965	0.881	0.847	0.898	0.846
428	0.971	0.889	0.844	0.902	0.852
429	0.962	0.886	0.853	0.901	0.851
430	0.968	0.884	0.850	0.901	0.851
431	0.962	0.894	0.847	0.902	0.852
432	0.960	0.876	0.847	0.894	0.841
433	0.962	0.891	0.865	0.906	0.859
434	0.974	0.868	0.853	0.898	0.847
435	0.962	0.886	0.835	0.895	0.842

436	0.965	0.876	0.844	0.895	0.842
437	0.962	0.894	0.838	0.899	0.848
438	0.965	0.884	0.847	0.899	0.848
439	0.968	0.884	0.838	0.897	0.845
440	0.962	0.879	0.853	0.898	0.846
441	0.968	0.886	0.844	0.900	0.849
442	0.962	0.897	0.841	0.901	0.851
443	0.974	0.894	0.850	0.906	0.859
444	0.962	0.871	0.844	0.892	0.838
445	0.968	0.884	0.841	0.898	0.846
446	0.968	0.891	0.838	0.900	0.849
447	0.960	0.884	0.844	0.896	0.844
448	0.962	0.884	0.850	0.899	0.848
449	0.962	0.879	0.844	0.895	0.842
450	0.971	0.891	0.835	0.900	0.849
451	0.957	0.879	0.841	0.892	0.838
452	0.965	0.897	0.835	0.900	0.849
453	0.971	0.881	0.844	0.899	0.848
454	0.968	0.881	0.850	0.900	0.849
455	0.965	0.886	0.826	0.893	0.839
456	0.962	0.876	0.850	0.896	0.844
457	0.962	0.881	0.844	0.896	0.844
458	0.968	0.884	0.844	0.899	0.848
459	0.965	0.886	0.850	0.901	0.851
460	0.968	0.889	0.847	0.902	0.852
461	0.968	0.889	0.841	0.900	0.849
462	0.962	0.866	0.853	0.893	0.839
463	0.968	0.886	0.850	0.902	0.852
464	0.960	0.886	0.844	0.897	0.845
465	0.968	0.889	0.829	0.896	0.844
466	0.965	0.876	0.844	0.895	0.842
467	0.965	0.879	0.832	0.892	0.838
468	0.971	0.879	0.844	0.898	0.847
469	0.971	0.894	0.829	0.899	0.848
470	0.965	0.879	0.847	0.897	0.845
471	0.968	0.899	0.832	0.901	0.851
472	0.971	0.873	0.835	0.893	0.839
473	0.971	0.879	0.832	0.894	0.841
474	0.962	0.879	0.829	0.890	0.835
475	0.960	0.879	0.853	0.897	0.845
476	0.960	0.873	0.850	0.894	0.841
477	0.971	0.881	0.841	0.898	0.846

478	0.965	0.884	0.841	0.897	0.845
479	0.968	0.879	0.844	0.897	0.845
480	0.962	0.884	0.847	0.898	0.846
481	0.968	0.889	0.844	0.901	0.851
482	0.968	0.876	0.832	0.892	0.838
483	0.965	0.873	0.847	0.895	0.842
484	0.968	0.881	0.838	0.896	0.844
485	0.968	0.868	0.853	0.896	0.844
486	0.965	0.884	0.853	0.901	0.851
487	0.968	0.891	0.838	0.900	0.849
488	0.960	0.879	0.853	0.897	0.845
489	0.965	0.889	0.835	0.897	0.845
490	0.971	0.884	0.847	0.901	0.851
491	0.968	0.886	0.841	0.899	0.848
492	0.965	0.881	0.838	0.895	0.842
493	0.960	0.873	0.841	0.891	0.837
494	0.965	0.879	0.853	0.899	0.848
495	0.968	0.891	0.841	0.901	0.851
496	0.968	0.884	0.853	0.902	0.852
497	0.962	0.884	0.841	0.896	0.844
498	0.965	0.889	0.832	0.896	0.844
499	0.965	0.868	0.841	0.891	0.837
500	0.962	0.873	0.841	0.892	0.838
501	0.965	0.876	0.844	0.895	0.842
502	0.962	0.886	0.838	0.896	0.844
503	0.962	0.879	0.832	0.891	0.837
504	0.965	0.881	0.841	0.896	0.844
505	0.960	0.889	0.847	0.899	0.848
506	0.954	0.889	0.853	0.899	0.848
507	0.965	0.884	0.844	0.898	0.846
508	0.960	0.879	0.847	0.895	0.842
509	0.965	0.894	0.847	0.903	0.853
510	0.971	0.891	0.841	0.902	0.852
511	0.954	0.897	0.844	0.899	0.848
512	0.960	0.884	0.835	0.893	0.839
513	0.962	0.889	0.841	0.898	0.846
514	0.957	0.881	0.847	0.895	0.842
515	0.960	0.881	0.841	0.894	0.841
516	0.968	0.871	0.835	0.891	0.837
517	0.960	0.881	0.847	0.896	0.844
518	0.965	0.871	0.838	0.891	0.837
519	0.960	0.873	0.853	0.895	0.842

520	0.971	0.881	0.841	0.898	0.846
521	0.962	0.889	0.847	0.900	0.849
522	0.962	0.884	0.841	0.896	0.844
523	0.960	0.884	0.847	0.897	0.845
524	0.962	0.876	0.853	0.897	0.845
525	0.965	0.889	0.844	0.900	0.849
526	0.965	0.884	0.835	0.895	0.842
527	0.962	0.876	0.835	0.891	0.837
528	0.968	0.884	0.838	0.897	0.845
529	0.957	0.876	0.847	0.893	0.839
530	0.965	0.873	0.847	0.895	0.842
531	0.960	0.881	0.835	0.892	0.838
532	0.968	0.881	0.832	0.894	0.841
533	0.965	0.897	0.838	0.901	0.851
534	0.965	0.879	0.853	0.899	0.848
535	0.965	0.897	0.841	0.902	0.852
536	0.965	0.884	0.850	0.900	0.849
537	0.957	0.897	0.847	0.901	0.851
538	0.960	0.866	0.847	0.890	0.835
539	0.965	0.886	0.850	0.901	0.851
540	0.971	0.886	0.838	0.899	0.848
541	0.965	0.868	0.838	0.890	0.835
542	0.960	0.873	0.847	0.893	0.839
543	0.974	0.884	0.844	0.901	0.851
544	0.962	0.884	0.841	0.896	0.844
545	0.968	0.873	0.850	0.897	0.845
546	0.962	0.876	0.844	0.894	0.841
547	0.971	0.876	0.838	0.895	0.842
548	0.968	0.891	0.853	0.904	0.856
549	0.954	0.886	0.847	0.896	0.844
550	0.965	0.876	0.835	0.892	0.838
551	0.962	0.881	0.838	0.894	0.841
552	0.974	0.886	0.853	0.904	0.856
553	0.977	0.873	0.853	0.901	0.851
554	0.965	0.884	0.835	0.895	0.842
555	0.960	0.868	0.835	0.888	0.831
556	0.965	0.889	0.841	0.899	0.848
557	0.974	0.873	0.844	0.897	0.845
558	0.965	0.879	0.838	0.894	0.841
559	0.957	0.876	0.835	0.889	0.834
560	0.962	0.876	0.841	0.893	0.839
561	0.965	0.881	0.835	0.894	0.841

562	0.965	0.886	0.844	0.899	0.848
563	0.968	0.886	0.832	0.896	0.844
564	0.971	0.886	0.832	0.897	0.845
565	0.962	0.884	0.838	0.895	0.842
566	0.965	0.881	0.841	0.896	0.844
567	0.971	0.889	0.826	0.896	0.844
568	0.965	0.884	0.832	0.894	0.841
569	0.962	0.881	0.844	0.896	0.844
570	0.960	0.866	0.844	0.889	0.834
571	0.962	0.881	0.826	0.890	0.835
572	0.974	0.886	0.847	0.903	0.854
573	0.962	0.876	0.841	0.893	0.839
574	0.968	0.879	0.844	0.897	0.845
575	0.965	0.860	0.844	0.889	0.834
576	0.962	0.886	0.844	0.898	0.846
577	0.971	0.881	0.832	0.895	0.842
578	0.971	0.873	0.844	0.896	0.844
579	0.974	0.876	0.829	0.893	0.839
580	0.968	0.884	0.847	0.900	0.849
581	0.980	0.876	0.826	0.894	0.841
582	0.965	0.871	0.838	0.891	0.837
583	0.968	0.866	0.841	0.891	0.837
584	0.965	0.879	0.832	0.892	0.838
585	0.962	0.876	0.838	0.892	0.838
586	0.965	0.881	0.823	0.890	0.835
587	0.962	0.889	0.832	0.895	0.842
588	0.965	0.889	0.847	0.901	0.851
589	0.974	0.879	0.838	0.897	0.845
590	0.965	0.879	0.850	0.898	0.846
591	0.968	0.881	0.826	0.892	0.838
592	0.971	0.889	0.838	0.900	0.849
593	0.968	0.876	0.844	0.896	0.844
594	0.974	0.876	0.841	0.897	0.845
595	0.957	0.876	0.832	0.888	0.832
596	0.974	0.879	0.835	0.896	0.844
597	0.977	0.876	0.835	0.896	0.844
598	0.965	0.881	0.835	0.894	0.841
599	0.968	0.871	0.844	0.894	0.841
600	0.974	0.884	0.826	0.895	0.842
601	0.965	0.879	0.844	0.896	0.844
602	0.971	0.873	0.850	0.898	0.846
603	0.971	0.871	0.844	0.895	0.842

604	0.971	0.876	0.847	0.898	0.846
605	0.971	0.881	0.838	0.897	0.845
606	0.965	0.868	0.835	0.889	0.834
607	0.968	0.876	0.838	0.894	0.841
608	0.974	0.871	0.838	0.894	0.841
609	0.974	0.873	0.844	0.897	0.845
610	0.965	0.871	0.844	0.893	0.839
611	0.971	0.873	0.838	0.894	0.841
612	0.965	0.873	0.841	0.893	0.839
613	0.971	0.868	0.847	0.895	0.842
614	0.974	0.871	0.844	0.896	0.844
615	0.962	0.886	0.835	0.895	0.842
616	0.974	0.876	0.832	0.894	0.841
617	0.968	0.871	0.850	0.896	0.844
618	0.965	0.886	0.838	0.897	0.845
619	0.971	0.879	0.829	0.893	0.839
620	0.971	0.868	0.832	0.890	0.835
621	0.971	0.876	0.838	0.895	0.842
622	0.968	0.876	0.838	0.894	0.841
623	0.977	0.858	0.850	0.894	0.841
624	0.971	0.884	0.859	0.904	0.856
625	0.965	0.881	0.835	0.894	0.841
626	0.965	0.873	0.844	0.894	0.841
627	0.971	0.884	0.826	0.894	0.841
628	0.965	0.879	0.829	0.891	0.837
629	0.974	0.881	0.838	0.898	0.846
630	0.965	0.884	0.835	0.895	0.842
631	0.974	0.884	0.838	0.899	0.848
632	0.977	0.884	0.832	0.898	0.847
633	0.974	0.876	0.841	0.897	0.845
634	0.968	0.876	0.838	0.894	0.841
635	0.971	0.881	0.826	0.893	0.839
636	0.965	0.868	0.832	0.888	0.832
637	0.971	0.889	0.835	0.899	0.848
638	0.971	0.894	0.835	0.901	0.851
639	0.971	0.863	0.829	0.888	0.831
640	0.968	0.871	0.829	0.889	0.834
641	0.962	0.871	0.820	0.885	0.827
642	0.971	0.879	0.844	0.898	0.846
643	0.971	0.879	0.841	0.897	0.845
644	0.965	0.871	0.838	0.891	0.837
645	0.974	0.879	0.817	0.890	0.835

646	0.974	0.894	0.835	0.902	0.852
647	0.971	0.876	0.835	0.894	0.841
648	0.974	0.873	0.829	0.892	0.838
649	0.965	0.879	0.835	0.893	0.839
650	0.965	0.879	0.838	0.894	0.841
651	0.971	0.879	0.835	0.895	0.842
652	0.971	0.879	0.832	0.894	0.841
653	0.974	0.879	0.835	0.896	0.844
654	0.962	0.873	0.835	0.890	0.835
655	0.965	0.876	0.829	0.890	0.835
656	0.971	0.881	0.835	0.896	0.844
657	0.968	0.879	0.835	0.894	0.841
658	0.962	0.881	0.835	0.893	0.839
659	0.971	0.868	0.841	0.893	0.839
660	0.968	0.886	0.844	0.900	0.849
661	0.965	0.866	0.829	0.887	0.830
662	0.971	0.873	0.838	0.894	0.841
663	0.965	0.879	0.844	0.896	0.844
664	0.971	0.863	0.838	0.890	0.835
665	0.977	0.863	0.835	0.891	0.837
666	0.962	0.863	0.838	0.888	0.831
667	0.965	0.868	0.841	0.891	0.837
668	0.962	0.868	0.838	0.889	0.834
669	0.974	0.886	0.844	0.902	0.852
670	0.968	0.871	0.841	0.893	0.839
671	0.965	0.873	0.841	0.893	0.839
672	0.974	0.889	0.829	0.898	0.846
673	0.962	0.879	0.841	0.894	0.841
674	0.962	0.871	0.829	0.888	0.831
675	0.980	0.866	0.847	0.897	0.845
676	0.971	0.873	0.835	0.893	0.839
677	0.971	0.886	0.847	0.902	0.852
678	0.971	0.884	0.829	0.895	0.842
679	0.962	0.868	0.829	0.887	0.830
680	0.965	0.876	0.838	0.893	0.839
681	0.980	0.871	0.847	0.899	0.848
682	0.974	0.876	0.838	0.896	0.844
683	0.974	0.886	0.838	0.900	0.849
684	0.965	0.873	0.847	0.895	0.842
685	0.971	0.866	0.835	0.890	0.835
686	0.962	0.868	0.832	0.888	0.831
687	0.974	0.871	0.832	0.892	0.838

688	0.965	0.881	0.838	0.895	0.842
689	0.962	0.871	0.841	0.891	0.837
690	0.968	0.884	0.829	0.894	0.841
691	0.965	0.886	0.841	0.898	0.846
692	0.962	0.879	0.841	0.894	0.841
693	0.965	0.881	0.838	0.895	0.842
694	0.974	0.879	0.853	0.902	0.852
695	0.971	0.871	0.823	0.888	0.832
696	0.965	0.881	0.826	0.891	0.837
697	0.960	0.876	0.835	0.890	0.835
698	0.968	0.881	0.841	0.897	0.845
699	0.974	0.873	0.823	0.890	0.835
700	0.971	0.879	0.838	0.896	0.844
701	0.968	0.889	0.832	0.897	0.845
702	0.968	0.876	0.850	0.898	0.846
703	0.968	0.876	0.841	0.895	0.842
704	0.965	0.879	0.832	0.892	0.838
705	0.974	0.871	0.832	0.892	0.838
706	0.965	0.868	0.835	0.889	0.834
707	0.968	0.881	0.841	0.897	0.845
708	0.968	0.886	0.832	0.896	0.844
709	0.968	0.863	0.829	0.887	0.830
710	0.965	0.871	0.850	0.895	0.842
711	0.974	0.873	0.844	0.897	0.845
712	0.968	0.866	0.844	0.892	0.838
713	0.977	0.879	0.835	0.897	0.845
714	0.971	0.871	0.844	0.895	0.842
715	0.968	0.858	0.841	0.888	0.832
716	0.968	0.866	0.841	0.891	0.837
717	0.968	0.884	0.826	0.893	0.839
718	0.962	0.881	0.841	0.895	0.842
719	0.962	0.876	0.826	0.888	0.832
720	0.968	0.876	0.826	0.890	0.835
721	0.962	0.881	0.826	0.890	0.835
722	0.974	0.879	0.835	0.896	0.844
723	0.968	0.884	0.844	0.899	0.848
724	0.974	0.860	0.844	0.892	0.838
725	0.974	0.884	0.844	0.901	0.851
726	0.957	0.881	0.838	0.892	0.838
727	0.971	0.886	0.847	0.902	0.852
728	0.965	0.866	0.844	0.891	0.837
729	0.968	0.866	0.838	0.890	0.835

730	0.954	0.879	0.829	0.888	0.831
731	0.957	0.881	0.844	0.894	0.841
732	0.960	0.886	0.823	0.890	0.835
733	0.965	0.876	0.829	0.890	0.835
734	0.960	0.871	0.829	0.887	0.830
735	0.962	0.884	0.847	0.898	0.846
736	0.962	0.876	0.832	0.890	0.835
737	0.965	0.871	0.823	0.887	0.830
738	0.960	0.871	0.835	0.888	0.832
739	0.968	0.868	0.841	0.892	0.838
740	0.968	0.881	0.841	0.897	0.845
741	0.962	0.879	0.838	0.893	0.839
742	0.957	0.884	0.826	0.889	0.834
743	0.960	0.884	0.832	0.892	0.838
744	0.962	0.884	0.838	0.895	0.842
745	0.962	0.881	0.838	0.894	0.841
746	0.962	0.879	0.841	0.894	0.841
747	0.965	0.886	0.838	0.897	0.845
748	0.962	0.884	0.838	0.895	0.842
749	0.962	0.866	0.847	0.891	0.837
750	0.962	0.868	0.835	0.888	0.832
751	0.962	0.879	0.832	0.891	0.837
752	0.971	0.876	0.832	0.893	0.839
753	0.968	0.863	0.838	0.889	0.834
754	0.960	0.881	0.835	0.892	0.838
755	0.954	0.876	0.853	0.894	0.841
756	0.968	0.868	0.832	0.889	0.834
757	0.965	0.868	0.844	0.892	0.838
758	0.962	0.855	0.829	0.882	0.823
759	0.965	0.876	0.838	0.893	0.839
760	0.965	0.881	0.826	0.891	0.837
761	0.962	0.873	0.835	0.890	0.835
762	0.977	0.879	0.832	0.896	0.844
763	0.965	0.876	0.838	0.893	0.839
764	0.960	0.876	0.844	0.893	0.839
765	0.971	0.879	0.835	0.895	0.842
766	0.965	0.873	0.853	0.897	0.845
767	0.968	0.871	0.820	0.887	0.830
768	0.965	0.858	0.829	0.884	0.825
769	0.968	0.871	0.844	0.894	0.841
770	0.965	0.868	0.844	0.892	0.838
771	0.954	0.879	0.841	0.891	0.837

772	0.957	0.860	0.826	0.881	0.821
773	0.968	0.868	0.838	0.891	0.837
774	0.971	0.876	0.829	0.892	0.838
775	0.971	0.871	0.838	0.893	0.839
776	0.974	0.876	0.832	0.894	0.841
777	0.971	0.868	0.835	0.891	0.837
778	0.962	0.871	0.838	0.890	0.835
779	0.960	0.871	0.832	0.888	0.831
780	0.968	0.873	0.832	0.891	0.837
781	0.968	0.881	0.853	0.901	0.851
782	0.957	0.871	0.838	0.888	0.832
783	0.965	0.873	0.826	0.888	0.832
784	0.960	0.873	0.826	0.887	0.830
785	0.960	0.876	0.829	0.888	0.832
786	0.960	0.866	0.838	0.888	0.831
787	0.962	0.873	0.841	0.892	0.838
788	0.960	0.866	0.841	0.888	0.832

(4) Performance of SVM on top features yielded by three feature selection methods

**mRMR**					
**Numbers of features**	**G1**	**G2/M**	**S**	**ACC**	**MCC**
1	0.951	0.641	0.695	0.758	0.641
2	0.957	0.879	0.659	0.835	0.754
3	0.957	0.871	0.683	0.840	0.761
4	0.954	0.876	0.692	0.843	0.766
5	0.957	0.866	0.698	0.843	0.764
6	0.957	0.863	0.713	0.846	0.770
7	0.960	0.860	0.710	0.845	0.768
8	0.957	0.840	0.710	0.837	0.755
9	0.960	0.853	0.701	0.840	0.760
10	0.962	0.824	0.740	0.843	0.763
11	0.962	0.824	0.740	0.843	0.763
12	0.962	0.835	0.751	0.850	0.775
13	0.962	0.832	0.751	0.849	0.773
14	0.962	0.835	0.757	0.852	0.778
15	0.954	0.809	0.790	0.850	0.775
16	0.954	0.804	0.787	0.847	0.771
17	0.951	0.804	0.799	0.850	0.775
18	0.954	0.804	0.796	0.850	0.775
19	0.957	0.809	0.796	0.853	0.779
20	0.960	0.811	0.793	0.854	0.781
21	0.962	0.811	0.796	0.856	0.784
22	0.960	0.814	0.805	0.858	0.788

23	0.980	0.788	0.826	0.862	0.795
24	0.980	0.788	0.826	0.862	0.795
25	0.980	0.788	0.826	0.862	0.795
26	0.980	0.793	0.817	0.861	0.793
27	0.980	0.791	0.820	0.861	0.793
28	0.977	0.824	0.814	0.871	0.806
29	0.977	0.824	0.814	0.871	0.806
30	0.977	0.824	0.814	0.871	0.806
31	0.977	0.822	0.811	0.869	0.803
32	0.980	0.824	0.814	0.872	0.807
33	0.980	0.824	0.808	0.870	0.805
34	0.977	0.827	0.814	0.872	0.807
35	0.977	0.845	0.805	0.875	0.813
36	0.971	0.860	0.814	0.882	0.823
37	0.971	0.858	0.808	0.879	0.818
38	0.971	0.858	0.805	0.878	0.817
39	0.974	0.858	0.808	0.880	0.820
40	0.974	0.858	0.808	0.880	0.820
41	0.974	0.858	0.808	0.880	0.820
42	0.974	0.858	0.805	0.879	0.818
43	0.977	0.855	0.814	0.882	0.823
44	0.977	0.855	0.817	0.883	0.824
45	0.977	0.855	0.808	0.880	0.820
46	0.974	0.855	0.802	0.877	0.815
47	0.977	0.855	0.799	0.877	0.815
48	0.977	0.855	0.802	0.878	0.817
49	0.977	0.855	0.802	0.878	0.817
50	0.977	0.855	0.799	0.877	0.815
51	0.977	0.855	0.799	0.877	0.815
52	0.977	0.855	0.802	0.878	0.817
53	0.977	0.845	0.811	0.877	0.816
54	0.977	0.845	0.808	0.876	0.814
55	0.977	0.855	0.802	0.878	0.817
56	0.977	0.853	0.802	0.877	0.816
57	0.977	0.853	0.802	0.877	0.816
58	0.974	0.855	0.811	0.880	0.820
59	0.974	0.855	0.811	0.880	0.820
60	0.974	0.858	0.811	0.881	0.821
61	0.974	0.863	0.808	0.882	0.823
62	0.980	0.837	0.817	0.877	0.816
63	0.980	0.837	0.817	0.877	0.816
64	0.980	0.837	0.817	0.877	0.816

65	0.980	0.837	0.820	0.878	0.817
66	0.977	0.853	0.817	0.882	0.823
67	0.986	0.850	0.811	0.882	0.823
68	0.986	0.850	0.811	0.882	0.823
69	0.991	0.804	0.844	0.877	0.817
70	0.991	0.814	0.844	0.881	0.822
71	0.991	0.814	0.844	0.881	0.822
72	0.991	0.814	0.844	0.881	0.822
73	0.991	0.817	0.844	0.882	0.824
74	0.991	0.817	0.844	0.882	0.824
75	0.991	0.819	0.844	0.883	0.825
76	0.991	0.819	0.844	0.883	0.825
77	0.991	0.819	0.844	0.883	0.825
78	0.991	0.819	0.844	0.883	0.825
79	0.988	0.819	0.844	0.882	0.824
80	0.988	0.819	0.844	0.882	0.824
81	0.988	0.819	0.844	0.882	0.824
82	0.988	0.819	0.844	0.882	0.824
83	0.988	0.819	0.844	0.882	0.824
84	0.988	0.814	0.835	0.877	0.817
85	0.988	0.814	0.835	0.877	0.817
86	0.988	0.814	0.838	0.878	0.818
87	0.988	0.814	0.838	0.878	0.818
88	0.988	0.806	0.835	0.874	0.813
89	0.988	0.806	0.835	0.874	0.813
90	0.988	0.806	0.835	0.874	0.813
91	0.988	0.817	0.838	0.879	0.819
92	0.988	0.811	0.838	0.877	0.817
93	0.988	0.811	0.838	0.877	0.817
94	0.988	0.811	0.838	0.877	0.817
95	0.988	0.817	0.832	0.877	0.816
96	0.988	0.824	0.835	0.881	0.822
97	0.988	0.822	0.835	0.880	0.821
98	0.988	0.824	0.835	0.881	0.822
99	0.988	0.824	0.835	0.881	0.822
100	0.988	0.824	0.835	0.881	0.822
101	0.988	0.824	0.835	0.881	0.822
102	0.988	0.824	0.835	0.881	0.822
103	0.988	0.824	0.835	0.881	0.822
104	0.988	0.822	0.835	0.880	0.821
105	0.988	0.822	0.835	0.880	0.821
106	0.988	0.822	0.835	0.880	0.821

107	0.988	0.817	0.835	0.878	0.818
108	0.988	0.822	0.835	0.880	0.821
109	0.988	0.822	0.835	0.880	0.821
110	0.988	0.814	0.835	0.877	0.817
111	0.988	0.814	0.835	0.877	0.817
112	0.988	0.814	0.835	0.877	0.817
113	0.988	0.814	0.835	0.877	0.817
114	0.988	0.814	0.835	0.877	0.817
115	0.988	0.814	0.835	0.877	0.817
116	0.988	0.814	0.835	0.877	0.817
117	0.988	0.814	0.835	0.877	0.817
118	0.988	0.814	0.835	0.877	0.817
119	0.988	0.814	0.835	0.877	0.817
120	0.988	0.817	0.835	0.878	0.818
121	0.988	0.822	0.844	0.883	0.825
122	0.988	0.822	0.844	0.883	0.825
123	0.988	0.822	0.844	0.883	0.825
124	0.988	0.824	0.844	0.884	0.826
125	0.988	0.824	0.844	0.884	0.826
126	0.988	0.817	0.844	0.881	0.822
127	0.988	0.814	0.844	0.880	0.821
128	0.980	0.765	0.856	0.863	0.798
129	0.980	0.765	0.856	0.863	0.798
130	0.980	0.765	0.856	0.863	0.798
131	0.980	0.765	0.856	0.863	0.798
132	0.980	0.767	0.856	0.864	0.799
133	0.980	0.767	0.856	0.864	0.799
134	0.980	0.765	0.856	0.863	0.798
135	0.983	0.775	0.853	0.867	0.803
136	0.980	0.791	0.844	0.869	0.805
137	0.980	0.791	0.844	0.869	0.805
138	0.980	0.791	0.844	0.869	0.805
139	0.980	0.791	0.844	0.869	0.805
140	0.980	0.791	0.844	0.869	0.805
141	0.980	0.791	0.844	0.869	0.805
142	0.980	0.791	0.844	0.869	0.805
143	0.980	0.791	0.844	0.869	0.805
144	0.980	0.791	0.844	0.869	0.805
145	0.980	0.791	0.844	0.869	0.805
146	0.980	0.791	0.844	0.869	0.805
147	0.980	0.791	0.844	0.869	0.805
148	0.980	0.791	0.844	0.869	0.805

149	0.980	0.791	0.844	0.869	0.805
150	0.980	0.791	0.844	0.869	0.805
151	0.980	0.791	0.844	0.869	0.805
152	0.980	0.791	0.844	0.869	0.805
153	0.980	0.791	0.844	0.869	0.805
154	0.980	0.791	0.844	0.869	0.805
155	0.980	0.791	0.844	0.869	0.805
156	0.980	0.791	0.844	0.869	0.805
157	0.980	0.791	0.844	0.869	0.805
158	0.980	0.791	0.844	0.869	0.805
159	0.980	0.791	0.844	0.869	0.805
160	0.980	0.791	0.844	0.869	0.805
161	0.980	0.801	0.835	0.870	0.805
162	0.980	0.801	0.835	0.870	0.805
163	0.983	0.801	0.835	0.871	0.807
164	0.983	0.801	0.835	0.871	0.807
165	0.983	0.804	0.835	0.872	0.808
166	0.983	0.804	0.835	0.872	0.808
167	0.986	0.804	0.832	0.872	0.808
168	0.986	0.804	0.832	0.872	0.808
169	0.986	0.809	0.832	0.873	0.811
170	0.986	0.809	0.832	0.873	0.811
171	0.986	0.809	0.832	0.873	0.811
172	0.986	0.809	0.832	0.873	0.811
173	0.986	0.809	0.832	0.873	0.811
174	0.986	0.809	0.835	0.874	0.812
175	0.986	0.806	0.835	0.873	0.811
176	0.986	0.809	0.835	0.874	0.812
177	0.986	0.806	0.835	0.873	0.811
178	0.986	0.804	0.835	0.873	0.810
179	0.986	0.806	0.835	0.873	0.811
180	0.986	0.806	0.835	0.873	0.811
181	0.986	0.804	0.835	0.873	0.810
182	0.986	0.809	0.835	0.874	0.812
183	0.986	0.809	0.835	0.874	0.812
184	0.986	0.809	0.835	0.874	0.812
185	0.986	0.809	0.835	0.874	0.812
186	0.986	0.809	0.835	0.874	0.812
187	0.986	0.809	0.835	0.874	0.812
188	0.986	0.809	0.835	0.874	0.812
189	0.986	0.804	0.835	0.873	0.810
190	0.986	0.804	0.835	0.873	0.810

191	0.986	0.804	0.835	0.873	0.810
192	0.986	0.804	0.835	0.873	0.810
193	0.986	0.804	0.835	0.873	0.810
194	0.986	0.804	0.835	0.873	0.810
195	0.986	0.804	0.835	0.873	0.810
196	0.986	0.809	0.835	0.874	0.812
197	0.986	0.809	0.835	0.874	0.812
198	0.986	0.809	0.835	0.874	0.812
199	0.986	0.809	0.835	0.874	0.812
200	0.986	0.809	0.835	0.874	0.812
201	0.986	0.809	0.835	0.874	0.812
202	0.986	0.809	0.835	0.874	0.812
203	0.986	0.809	0.835	0.874	0.812
204	0.986	0.809	0.835	0.874	0.812
205	0.986	0.809	0.832	0.873	0.811
206	0.986	0.809	0.832	0.873	0.811
207	0.986	0.809	0.832	0.873	0.811
208	0.986	0.809	0.832	0.873	0.811
209	0.986	0.809	0.832	0.873	0.811
210	0.986	0.809	0.832	0.873	0.811
211	0.986	0.809	0.832	0.873	0.811
212	0.986	0.809	0.832	0.873	0.811
213	0.986	0.809	0.832	0.873	0.811
214	0.986	0.809	0.832	0.873	0.811
215	0.986	0.809	0.832	0.873	0.811
216	0.986	0.809	0.832	0.873	0.811
217	0.986	0.809	0.832	0.873	0.811
218	0.988	0.801	0.820	0.868	0.802
219	0.988	0.801	0.820	0.868	0.802
220	0.988	0.801	0.820	0.868	0.802
221	0.988	0.801	0.820	0.868	0.802
222	0.988	0.801	0.820	0.868	0.802
223	0.988	0.801	0.820	0.868	0.802
224	0.988	0.801	0.820	0.868	0.802
225	0.988	0.801	0.823	0.869	0.804
226	0.988	0.801	0.823	0.869	0.804
227	0.988	0.801	0.823	0.869	0.804
228	0.988	0.801	0.823	0.869	0.804
229	0.988	0.801	0.823	0.869	0.804
230	0.988	0.801	0.823	0.869	0.804
231	0.988	0.801	0.823	0.869	0.804
232	0.988	0.801	0.823	0.869	0.804

233	0.988	0.801	0.823	0.869	0.804
234	0.988	0.801	0.823	0.869	0.804
235	0.988	0.801	0.823	0.869	0.804
236	0.988	0.801	0.823	0.869	0.804
237	0.988	0.832	0.820	0.879	0.819
238	0.988	0.832	0.820	0.879	0.819
239	0.988	0.832	0.820	0.879	0.819
240	0.988	0.832	0.820	0.879	0.819
241	0.988	0.832	0.820	0.879	0.819
242	0.988	0.832	0.820	0.879	0.819
243	0.988	0.832	0.820	0.879	0.819
244	0.988	0.832	0.820	0.879	0.819
245	0.988	0.827	0.820	0.877	0.816
246	0.988	0.827	0.820	0.877	0.816
247	0.988	0.827	0.820	0.877	0.816
248	0.988	0.827	0.820	0.877	0.816
249	0.988	0.827	0.820	0.877	0.816
250	0.988	0.827	0.820	0.877	0.816
251	0.988	0.827	0.820	0.877	0.816
252	0.988	0.827	0.820	0.877	0.816
253	0.988	0.827	0.820	0.877	0.816
254	0.988	0.827	0.820	0.877	0.816
255	0.988	0.827	0.820	0.877	0.816
256	0.988	0.827	0.820	0.877	0.816
257	0.988	0.824	0.820	0.876	0.815
258	0.988	0.824	0.820	0.876	0.815
259	0.988	0.824	0.820	0.876	0.815
260	0.988	0.824	0.820	0.876	0.815
261	0.988	0.824	0.820	0.876	0.815
262	0.988	0.824	0.820	0.876	0.815
263	0.988	0.824	0.820	0.876	0.815
264	0.988	0.824	0.820	0.876	0.815
265	0.988	0.824	0.820	0.876	0.815
266	0.988	0.827	0.820	0.877	0.816
267	0.988	0.827	0.820	0.877	0.816
268	0.988	0.824	0.820	0.876	0.815
269	0.988	0.824	0.820	0.876	0.815
270	0.986	0.822	0.820	0.874	0.812
271	0.986	0.822	0.817	0.873	0.810
272	0.986	0.822	0.817	0.873	0.810
273	0.986	0.822	0.817	0.873	0.810
274	0.986	0.822	0.817	0.873	0.810

275	0.986	0.822	0.817	0.873	0.810
276	0.986	0.822	0.817	0.873	0.810
277	0.986	0.822	0.817	0.873	0.810
278	0.986	0.822	0.817	0.873	0.810
279	0.986	0.822	0.817	0.873	0.810
280	0.986	0.822	0.817	0.873	0.810
281	0.986	0.822	0.817	0.873	0.810
282	0.986	0.822	0.817	0.873	0.810
283	0.986	0.822	0.817	0.873	0.810
284	0.986	0.822	0.817	0.873	0.810
285	0.986	0.822	0.817	0.873	0.810
286	0.986	0.822	0.817	0.873	0.810
287	0.986	0.822	0.817	0.873	0.810
288	0.986	0.822	0.817	0.873	0.810
289	0.986	0.822	0.817	0.873	0.810
290	0.986	0.822	0.820	0.874	0.812
291	0.986	0.822	0.820	0.874	0.812
292	0.986	0.822	0.820	0.874	0.812
293	0.988	0.822	0.823	0.876	0.815
294	0.988	0.819	0.823	0.875	0.813
295	0.988	0.819	0.820	0.874	0.812
296	0.988	0.819	0.820	0.874	0.812
297	0.988	0.819	0.823	0.875	0.813
298	0.988	0.819	0.823	0.875	0.813
299	0.988	0.819	0.823	0.875	0.813
300	0.988	0.819	0.823	0.875	0.813
301	0.988	0.819	0.823	0.875	0.813
302	0.988	0.819	0.823	0.875	0.813
303	0.988	0.819	0.826	0.876	0.815
304	0.986	0.822	0.820	0.874	0.812
305	0.986	0.822	0.820	0.874	0.812
306	0.986	0.822	0.820	0.874	0.812
307	0.986	0.822	0.820	0.874	0.812
308	0.986	0.822	0.820	0.874	0.812
309	0.986	0.822	0.820	0.874	0.812
310	0.986	0.819	0.823	0.874	0.812
311	0.986	0.819	0.823	0.874	0.812
312	0.986	0.819	0.823	0.874	0.812
313	0.986	0.819	0.823	0.874	0.812
314	0.986	0.804	0.832	0.872	0.808
315	0.986	0.804	0.832	0.872	0.808
316	0.986	0.804	0.832	0.872	0.808

317	0.986	0.804	0.832	0.872	0.808
318	0.986	0.804	0.832	0.872	0.808
319	0.986	0.804	0.832	0.872	0.808
320	0.986	0.804	0.832	0.872	0.808
321	0.986	0.804	0.832	0.872	0.808
322	0.986	0.804	0.832	0.872	0.808
323	0.986	0.804	0.832	0.872	0.808
324	0.986	0.804	0.832	0.872	0.808
325	0.986	0.804	0.832	0.872	0.808
326	0.986	0.804	0.832	0.872	0.808
327	0.986	0.804	0.832	0.872	0.808
328	0.986	0.804	0.832	0.872	0.808
329	0.986	0.804	0.832	0.872	0.808
330	0.986	0.804	0.832	0.872	0.808
331	0.986	0.804	0.832	0.872	0.808
332	0.986	0.804	0.832	0.872	0.808
333	0.986	0.804	0.832	0.872	0.808
334	0.986	0.804	0.832	0.872	0.808
335	0.986	0.804	0.832	0.872	0.808
336	0.986	0.804	0.832	0.872	0.808
337	0.986	0.804	0.832	0.872	0.808
338	0.986	0.804	0.832	0.872	0.808
339	0.983	0.806	0.829	0.871	0.807
340	0.983	0.806	0.829	0.871	0.807
341	0.983	0.806	0.829	0.871	0.807
342	0.983	0.806	0.829	0.871	0.807
343	0.983	0.806	0.829	0.871	0.807
344	0.983	0.806	0.829	0.871	0.807
345	0.983	0.804	0.829	0.870	0.805
346	0.983	0.804	0.829	0.870	0.805
347	0.983	0.804	0.829	0.870	0.805
348	0.983	0.804	0.829	0.870	0.805
349	0.983	0.804	0.829	0.870	0.805
350	0.983	0.804	0.829	0.870	0.805
351	0.986	0.793	0.838	0.870	0.806
352	0.986	0.791	0.844	0.871	0.807
353	0.986	0.791	0.838	0.869	0.805
354	0.986	0.791	0.838	0.869	0.805
355	0.986	0.791	0.838	0.869	0.805
356	0.991	0.791	0.838	0.871	0.807
357	0.991	0.791	0.838	0.871	0.807
358	0.991	0.791	0.838	0.871	0.807

359	0.991	0.791	0.838	0.871	0.807
360	0.991	0.791	0.838	0.871	0.807
361	0.991	0.791	0.838	0.871	0.807
362	0.983	0.783	0.844	0.867	0.802
363	0.983	0.780	0.844	0.866	0.801
364	0.983	0.780	0.844	0.866	0.801
365	0.983	0.780	0.844	0.866	0.801
366	0.983	0.778	0.850	0.867	0.802
367	0.983	0.778	0.850	0.867	0.802
368	0.983	0.778	0.850	0.867	0.802
369	0.983	0.778	0.850	0.867	0.802
370	0.983	0.778	0.850	0.867	0.802
371	0.983	0.778	0.850	0.867	0.802
372	0.986	0.778	0.853	0.869	0.805
373	0.986	0.778	0.853	0.869	0.805
374	0.986	0.778	0.853	0.869	0.805
375	0.986	0.778	0.853	0.869	0.805
376	0.986	0.778	0.853	0.869	0.805
377	0.986	0.778	0.853	0.869	0.805
378	0.986	0.778	0.853	0.869	0.805
379	0.986	0.778	0.853	0.869	0.805
380	0.986	0.778	0.853	0.869	0.805
381	0.986	0.778	0.853	0.869	0.805
382	0.986	0.778	0.853	0.869	0.805
383	0.986	0.778	0.853	0.869	0.805
384	0.986	0.778	0.853	0.869	0.805
385	0.986	0.778	0.853	0.869	0.805
386	0.986	0.778	0.850	0.868	0.804
387	0.986	0.778	0.850	0.868	0.804
388	0.986	0.778	0.847	0.867	0.802
389	0.986	0.778	0.850	0.868	0.804
390	0.986	0.778	0.850	0.868	0.804
391	0.986	0.778	0.850	0.868	0.804
392	0.986	0.778	0.850	0.868	0.804
393	0.986	0.778	0.850	0.868	0.804
394	0.986	0.778	0.850	0.868	0.804
395	0.986	0.778	0.853	0.869	0.805
396	0.986	0.778	0.853	0.869	0.805
397	0.986	0.778	0.853	0.869	0.805
398	0.986	0.778	0.853	0.869	0.805
399	0.986	0.778	0.853	0.869	0.805
400	0.986	0.778	0.841	0.865	0.799

401	0.986	0.778	0.841	0.865	0.799
402	0.986	0.778	0.841	0.865	0.799
403	0.986	0.778	0.841	0.865	0.799
404	0.986	0.778	0.841	0.865	0.799
405	0.986	0.778	0.841	0.865	0.799
406	0.986	0.778	0.841	0.865	0.799
407	0.986	0.778	0.841	0.865	0.799
408	0.986	0.778	0.841	0.865	0.799
409	0.986	0.778	0.841	0.865	0.799
410	0.986	0.778	0.841	0.865	0.799
411	0.986	0.778	0.841	0.865	0.799
412	0.986	0.778	0.841	0.865	0.799
413	0.986	0.778	0.841	0.865	0.799
414	0.986	0.778	0.841	0.865	0.799
415	0.983	0.775	0.847	0.865	0.800
416	0.983	0.775	0.847	0.865	0.800
417	0.983	0.775	0.847	0.865	0.800
418	0.983	0.775	0.847	0.865	0.800
419	0.983	0.775	0.847	0.865	0.800
420	0.983	0.775	0.847	0.865	0.800
421	0.983	0.775	0.847	0.865	0.800
422	0.980	0.767	0.850	0.862	0.796
423	0.980	0.767	0.850	0.862	0.796
424	0.980	0.770	0.850	0.863	0.797
425	0.980	0.770	0.850	0.863	0.797
426	0.980	0.770	0.850	0.863	0.797
427	0.980	0.770	0.850	0.863	0.797
428	0.980	0.770	0.850	0.863	0.797
429	0.980	0.770	0.850	0.863	0.797
430	0.980	0.770	0.850	0.863	0.797
431	0.980	0.770	0.850	0.863	0.797
432	0.986	0.770	0.847	0.864	0.798
433	0.986	0.770	0.847	0.864	0.798
434	0.986	0.770	0.847	0.864	0.798
435	0.986	0.770	0.847	0.864	0.798
436	0.983	0.773	0.847	0.864	0.798
437	0.983	0.773	0.847	0.864	0.798
438	0.986	0.773	0.847	0.865	0.800
439	0.986	0.773	0.847	0.865	0.800
440	0.986	0.773	0.847	0.865	0.800
441	0.986	0.773	0.847	0.865	0.800
442	0.986	0.773	0.850	0.866	0.801

443	0.986	0.773	0.850	0.866	0.801
444	0.986	0.773	0.850	0.866	0.801
445	0.986	0.773	0.847	0.865	0.800
446	0.988	0.765	0.850	0.864	0.799
447	0.988	0.765	0.850	0.864	0.799
448	0.988	0.765	0.850	0.864	0.799
449	0.988	0.765	0.850	0.864	0.799
450	0.988	0.765	0.850	0.864	0.799
451	0.988	0.762	0.847	0.862	0.796
452	0.988	0.762	0.847	0.862	0.796
453	0.988	0.762	0.850	0.863	0.797
454	0.988	0.762	0.850	0.863	0.797
455	0.988	0.762	0.850	0.863	0.797
456	0.988	0.762	0.850	0.863	0.797
457	0.988	0.762	0.850	0.863	0.797
458	0.988	0.762	0.850	0.863	0.797
459	0.988	0.762	0.850	0.863	0.797
460	0.988	0.762	0.850	0.863	0.797
461	0.988	0.762	0.850	0.863	0.797
462	0.988	0.762	0.850	0.863	0.797
463	0.988	0.765	0.850	0.864	0.799
464	0.988	0.762	0.847	0.862	0.796
465	0.988	0.762	0.847	0.862	0.796
466	0.988	0.762	0.847	0.862	0.796
467	0.988	0.762	0.847	0.862	0.796
468	0.988	0.767	0.850	0.865	0.800
469	0.988	0.767	0.850	0.865	0.800
470	0.988	0.767	0.850	0.865	0.800
471	0.988	0.767	0.850	0.865	0.800
472	0.988	0.767	0.850	0.865	0.800
473	0.988	0.767	0.850	0.865	0.800
474	0.988	0.767	0.850	0.865	0.800
475	0.988	0.767	0.850	0.865	0.800
476	0.988	0.767	0.850	0.865	0.800
477	0.988	0.767	0.850	0.865	0.800
478	0.988	0.767	0.850	0.865	0.800
479	0.988	0.767	0.850	0.865	0.800
480	0.988	0.767	0.850	0.865	0.800
481	0.988	0.767	0.850	0.865	0.800
482	0.986	0.762	0.853	0.863	0.797
483	0.986	0.762	0.853	0.863	0.797
484	0.986	0.762	0.853	0.863	0.797

485	0.986	0.762	0.853	0.863	0.797
486	0.986	0.762	0.853	0.863	0.797
487	0.986	0.762	0.853	0.863	0.797
488	0.980	0.767	0.853	0.863	0.797
489	0.980	0.765	0.856	0.863	0.797
490	0.980	0.765	0.856	0.863	0.797
491	0.980	0.765	0.856	0.863	0.797
492	0.980	0.765	0.856	0.863	0.797
493	0.980	0.762	0.859	0.863	0.798
494	0.980	0.752	0.859	0.859	0.792
495	0.980	0.760	0.862	0.863	0.798
496	0.980	0.757	0.862	0.862	0.797
497	0.983	0.755	0.862	0.862	0.797
498	0.983	0.755	0.862	0.862	0.797
499	0.983	0.755	0.862	0.862	0.797
500	0.974	0.742	0.856	0.853	0.783
501	0.974	0.742	0.853	0.852	0.781
502	0.974	0.742	0.853	0.852	0.781
503	0.974	0.742	0.853	0.852	0.781
504	0.974	0.742	0.853	0.852	0.781
505	0.974	0.742	0.853	0.852	0.781
506	0.974	0.739	0.853	0.851	0.780
507	0.974	0.742	0.853	0.852	0.781
508	0.974	0.742	0.853	0.852	0.781
509	0.974	0.742	0.853	0.852	0.781
510	0.974	0.742	0.853	0.852	0.781
511	0.974	0.742	0.853	0.852	0.781
512	0.974	0.739	0.853	0.851	0.780
513	0.974	0.742	0.853	0.852	0.781
514	0.974	0.739	0.853	0.851	0.780
515	0.974	0.739	0.856	0.852	0.782
516	0.974	0.739	0.856	0.852	0.782
517	0.974	0.742	0.856	0.853	0.783
518	0.974	0.742	0.856	0.853	0.783
519	0.974	0.742	0.856	0.853	0.783
520	0.974	0.744	0.856	0.854	0.784
521	0.974	0.744	0.856	0.854	0.784
522	0.977	0.739	0.862	0.855	0.786
523	0.977	0.739	0.862	0.855	0.786
524	0.977	0.739	0.862	0.855	0.786
525	0.977	0.739	0.862	0.855	0.786
526	0.977	0.739	0.862	0.855	0.786

527	0.977	0.739	0.862	0.855	0.786
528	0.977	0.739	0.862	0.855	0.786
529	0.977	0.739	0.862	0.855	0.786
530	0.977	0.739	0.862	0.855	0.786
531	0.977	0.742	0.862	0.856	0.787
532	0.977	0.742	0.862	0.856	0.787
533	0.977	0.744	0.862	0.857	0.789
534	0.977	0.747	0.865	0.858	0.791
535	0.977	0.747	0.865	0.858	0.791
536	0.974	0.742	0.859	0.854	0.784
537	0.974	0.742	0.859	0.854	0.784
538	0.974	0.742	0.859	0.854	0.784
539	0.971	0.752	0.856	0.856	0.787
540	0.971	0.752	0.856	0.856	0.787
541	0.971	0.752	0.856	0.856	0.787
542	0.971	0.755	0.856	0.857	0.788
543	0.971	0.755	0.856	0.857	0.788
544	0.971	0.755	0.856	0.857	0.788
545	0.971	0.755	0.856	0.857	0.788
546	0.971	0.757	0.853	0.857	0.788
547	0.971	0.752	0.853	0.855	0.785
548	0.971	0.752	0.853	0.855	0.785
549	0.971	0.752	0.853	0.855	0.785
550	0.971	0.752	0.853	0.855	0.785
551	0.971	0.752	0.853	0.855	0.785
552	0.971	0.752	0.853	0.855	0.785
553	0.971	0.752	0.853	0.855	0.785
554	0.971	0.752	0.853	0.855	0.785
555	0.971	0.752	0.853	0.855	0.785
556	0.971	0.752	0.853	0.855	0.785
557	0.971	0.752	0.853	0.855	0.785
558	0.971	0.752	0.853	0.855	0.785
559	0.971	0.752	0.853	0.855	0.785
560	0.971	0.752	0.853	0.855	0.785
561	0.971	0.752	0.853	0.855	0.785
562	0.971	0.752	0.853	0.855	0.785
563	0.971	0.752	0.853	0.855	0.785
564	0.971	0.752	0.853	0.855	0.785
565	0.971	0.752	0.853	0.855	0.785
566	0.971	0.752	0.853	0.855	0.785
567	0.971	0.752	0.853	0.855	0.785
568	0.971	0.752	0.856	0.856	0.787

569	0.971	0.752	0.856	0.856	0.787
570	0.971	0.752	0.856	0.856	0.787
571	0.971	0.749	0.856	0.855	0.785
572	0.971	0.749	0.862	0.857	0.788
573	0.971	0.749	0.862	0.857	0.788
574	0.971	0.749	0.862	0.857	0.788
575	0.971	0.749	0.862	0.857	0.788
576	0.971	0.749	0.862	0.857	0.788
577	0.971	0.749	0.862	0.857	0.788
578	0.971	0.749	0.862	0.857	0.788
579	0.971	0.749	0.862	0.857	0.788
580	0.971	0.749	0.862	0.857	0.788
581	0.971	0.749	0.862	0.857	0.788
582	0.971	0.747	0.859	0.855	0.786
583	0.971	0.747	0.859	0.855	0.786
584	0.971	0.747	0.859	0.855	0.786
585	0.971	0.747	0.859	0.855	0.786
586	0.971	0.744	0.859	0.854	0.784
587	0.971	0.744	0.859	0.854	0.784
588	0.971	0.744	0.859	0.854	0.784
589	0.971	0.744	0.859	0.854	0.784
590	0.971	0.744	0.859	0.854	0.784
591	0.971	0.747	0.859	0.855	0.786
592	0.971	0.747	0.859	0.855	0.786
593	0.971	0.747	0.859	0.855	0.786
594	0.971	0.747	0.859	0.855	0.786
595	0.971	0.744	0.859	0.854	0.784
596	0.971	0.747	0.859	0.855	0.786
597	0.971	0.744	0.859	0.854	0.784
598	0.971	0.744	0.859	0.854	0.784
599	0.971	0.744	0.859	0.854	0.784
600	0.971	0.744	0.859	0.854	0.784
601	0.971	0.744	0.859	0.854	0.784
602	0.971	0.744	0.859	0.854	0.784
603	0.971	0.744	0.859	0.854	0.784
604	0.971	0.744	0.859	0.854	0.784
605	0.971	0.744	0.859	0.854	0.784
606	0.971	0.744	0.859	0.854	0.784
607	0.971	0.744	0.859	0.854	0.784
608	0.971	0.744	0.859	0.854	0.784
609	0.971	0.744	0.859	0.854	0.784
610	0.971	0.749	0.847	0.852	0.781

611	0.971	0.749	0.847	0.852	0.781
612	0.971	0.749	0.847	0.852	0.781
613	0.974	0.752	0.847	0.854	0.783
614	0.974	0.752	0.847	0.854	0.783
615	0.974	0.752	0.847	0.854	0.783
616	0.974	0.752	0.847	0.854	0.783
617	0.974	0.752	0.847	0.854	0.783
618	0.974	0.752	0.847	0.854	0.783
619	0.974	0.752	0.847	0.854	0.783
620	0.971	0.752	0.847	0.853	0.782
621	0.971	0.752	0.847	0.853	0.782
622	0.971	0.752	0.847	0.853	0.782
623	0.971	0.752	0.847	0.853	0.782
624	0.971	0.752	0.847	0.853	0.782
625	0.971	0.752	0.847	0.853	0.782
626	0.971	0.752	0.847	0.853	0.782
627	0.971	0.752	0.847	0.853	0.782
628	0.971	0.752	0.847	0.853	0.782
629	0.971	0.752	0.847	0.853	0.782
630	0.971	0.752	0.847	0.853	0.782
631	0.971	0.752	0.847	0.853	0.782
632	0.971	0.752	0.847	0.853	0.782
633	0.971	0.752	0.847	0.853	0.782
634	0.971	0.752	0.847	0.853	0.782
635	0.971	0.752	0.847	0.853	0.782
636	0.971	0.752	0.847	0.853	0.782
637	0.971	0.752	0.847	0.853	0.782
638	0.971	0.752	0.847	0.853	0.782
639	0.971	0.752	0.847	0.853	0.782
640	0.971	0.752	0.847	0.853	0.782
641	0.971	0.752	0.847	0.853	0.782
642	0.971	0.752	0.847	0.853	0.782
643	0.971	0.752	0.847	0.853	0.782
644	0.971	0.752	0.847	0.853	0.782
645	0.971	0.752	0.847	0.853	0.782
646	0.971	0.752	0.847	0.853	0.782
647	0.971	0.752	0.847	0.853	0.782
648	0.971	0.752	0.847	0.853	0.782
649	0.971	0.752	0.847	0.853	0.782
650	0.971	0.752	0.847	0.853	0.782
651	0.971	0.752	0.847	0.853	0.782
652	0.971	0.752	0.847	0.853	0.782

653	0.971	0.752	0.847	0.853	0.782
654	0.974	0.752	0.847	0.854	0.783
655	0.974	0.752	0.847	0.854	0.783
656	0.974	0.752	0.847	0.854	0.783
657	0.977	0.752	0.847	0.855	0.785
658	0.977	0.752	0.847	0.855	0.785
659	0.977	0.752	0.847	0.855	0.785
660	0.977	0.752	0.847	0.855	0.785
661	0.977	0.752	0.847	0.855	0.785
662	0.977	0.752	0.847	0.855	0.785
663	0.977	0.752	0.847	0.855	0.785
664	0.977	0.752	0.847	0.855	0.785
665	0.977	0.752	0.847	0.855	0.785
666	0.977	0.752	0.847	0.855	0.785
667	0.977	0.752	0.847	0.855	0.785
668	0.977	0.752	0.847	0.855	0.785
669	0.977	0.752	0.847	0.855	0.785
670	0.977	0.752	0.847	0.855	0.785
671	0.977	0.752	0.847	0.855	0.785
672	0.977	0.752	0.847	0.855	0.785
673	0.977	0.752	0.847	0.855	0.785
674	0.977	0.752	0.847	0.855	0.785
675	0.977	0.752	0.847	0.855	0.785
676	0.977	0.752	0.847	0.855	0.785
677	0.977	0.752	0.847	0.855	0.785
678	0.977	0.752	0.847	0.855	0.785
679	0.977	0.752	0.847	0.855	0.785
680	0.977	0.752	0.847	0.855	0.785
681	0.977	0.752	0.847	0.855	0.785
682	0.977	0.752	0.847	0.855	0.785
683	0.977	0.752	0.847	0.855	0.785
684	0.977	0.752	0.847	0.855	0.785
685	0.977	0.752	0.847	0.855	0.785
686	0.977	0.752	0.847	0.855	0.785
687	0.977	0.752	0.847	0.855	0.785
688	0.977	0.752	0.847	0.855	0.785
689	0.977	0.752	0.847	0.855	0.785
690	0.977	0.752	0.847	0.855	0.785
691	0.977	0.752	0.847	0.855	0.785
692	0.977	0.752	0.847	0.855	0.785
693	0.977	0.752	0.847	0.855	0.785
694	0.977	0.752	0.847	0.855	0.785

695	0.977	0.752	0.847	0.855	0.785
696	0.977	0.752	0.847	0.855	0.785
697	0.977	0.752	0.847	0.855	0.785
698	0.977	0.752	0.850	0.856	0.786
699	0.977	0.752	0.850	0.856	0.786
700	0.977	0.752	0.850	0.856	0.786
701	0.977	0.752	0.850	0.856	0.786
702	0.977	0.752	0.850	0.856	0.786
703	0.977	0.752	0.847	0.855	0.785
704	0.974	0.752	0.847	0.854	0.784
705	0.974	0.752	0.847	0.854	0.784
706	0.974	0.752	0.847	0.854	0.784
707	0.974	0.752	0.847	0.854	0.784
708	0.974	0.752	0.847	0.854	0.784
709	0.974	0.752	0.847	0.854	0.784
710	0.974	0.752	0.847	0.854	0.784
711	0.974	0.752	0.847	0.854	0.784
712	0.974	0.752	0.847	0.854	0.784
713	0.974	0.752	0.847	0.854	0.784
714	0.974	0.752	0.850	0.855	0.785
715	0.974	0.752	0.847	0.854	0.784
716	0.974	0.752	0.850	0.855	0.785
717	0.974	0.752	0.850	0.855	0.785
718	0.974	0.752	0.850	0.855	0.785
719	0.971	0.752	0.850	0.854	0.784
720	0.971	0.752	0.847	0.853	0.782
721	0.974	0.752	0.847	0.854	0.783
722	0.971	0.752	0.847	0.853	0.782
723	0.974	0.752	0.847	0.854	0.784
724	0.974	0.752	0.847	0.854	0.784
725	0.974	0.752	0.847	0.854	0.784
726	0.974	0.752	0.847	0.854	0.784
727	0.974	0.752	0.847	0.854	0.784
728	0.974	0.752	0.847	0.854	0.784
729	0.977	0.752	0.847	0.855	0.785
730	0.974	0.752	0.847	0.854	0.784
731	0.977	0.752	0.847	0.855	0.785
732	0.974	0.752	0.847	0.854	0.784
733	0.974	0.752	0.847	0.854	0.784
734	0.974	0.752	0.847	0.854	0.784
735	0.974	0.752	0.847	0.854	0.784
736	0.974	0.752	0.847	0.854	0.784

737	0.974	0.752	0.847	0.854	0.784
738	0.974	0.752	0.847	0.854	0.784
739	0.980	0.749	0.847	0.855	0.785
740	0.980	0.749	0.847	0.855	0.785
741	0.980	0.749	0.847	0.855	0.785
742	0.980	0.749	0.847	0.855	0.785
743	0.980	0.749	0.847	0.855	0.785
744	0.980	0.749	0.847	0.855	0.785
745	0.980	0.749	0.847	0.855	0.785
746	0.980	0.749	0.847	0.855	0.785
747	0.980	0.749	0.847	0.855	0.785
748	0.980	0.749	0.847	0.855	0.785
749	0.980	0.749	0.847	0.855	0.785
750	0.980	0.749	0.847	0.855	0.785
751	0.980	0.749	0.847	0.855	0.785
752	0.980	0.749	0.847	0.855	0.785
753	0.980	0.749	0.847	0.855	0.785
754	0.980	0.749	0.847	0.855	0.785
755	0.980	0.749	0.847	0.855	0.785
756	0.980	0.749	0.847	0.855	0.785
757	0.980	0.749	0.847	0.855	0.785
758	0.980	0.749	0.847	0.855	0.785
759	0.980	0.749	0.847	0.855	0.785
760	0.971	0.731	0.859	0.849	0.778
761	0.971	0.731	0.859	0.849	0.778
762	0.971	0.731	0.859	0.849	0.778
763	0.971	0.731	0.859	0.849	0.778
764	0.971	0.731	0.859	0.849	0.778
765	0.971	0.731	0.859	0.849	0.778
766	0.971	0.731	0.859	0.849	0.778
767	0.971	0.731	0.859	0.849	0.778
768	0.971	0.729	0.859	0.848	0.777
769	0.971	0.734	0.859	0.850	0.779
770	0.971	0.734	0.859	0.850	0.779
771	0.971	0.734	0.859	0.850	0.779
772	0.971	0.731	0.859	0.849	0.778
773	0.971	0.731	0.859	0.849	0.778
774	0.971	0.731	0.859	0.849	0.778
775	0.971	0.731	0.859	0.849	0.778
776	0.971	0.731	0.859	0.849	0.778
777	0.971	0.731	0.859	0.849	0.778
778	0.971	0.731	0.859	0.849	0.778

779	0.971	0.736	0.859	0.851	0.780
780	0.971	0.736	0.859	0.851	0.780
781	0.971	0.734	0.859	0.850	0.779
782	0.971	0.734	0.859	0.850	0.779
783	0.971	0.734	0.859	0.850	0.779
784	0.971	0.734	0.859	0.850	0.779
785	0.971	0.731	0.859	0.849	0.778
786	0.971	0.734	0.859	0.850	0.779
787	0.971	0.736	0.859	0.851	0.780
788	0.971	0.736	0.859	0.851	0.780
**MCFS**					
**Numbers of features**	**G1**	**G2/M**	**S**	**ACC**	**MCC**
1	0.942	0.682	0.689	0.769	0.654
2	0.951	0.677	0.775	0.797	0.701
3	0.965	0.682	0.835	0.822	0.740
4	0.965	0.711	0.811	0.825	0.741
5	0.965	0.713	0.808	0.825	0.741
6	0.965	0.734	0.778	0.823	0.736
7	0.965	0.744	0.772	0.825	0.738
8	0.965	0.767	0.772	0.833	0.750
9	0.968	0.775	0.769	0.836	0.754
10	0.968	0.801	0.772	0.846	0.770
11	0.965	0.829	0.763	0.853	0.779
12	0.960	0.809	0.766	0.844	0.767
13	0.960	0.814	0.781	0.851	0.776
14	0.962	0.796	0.775	0.843	0.765
15	0.962	0.832	0.760	0.852	0.778
16	0.960	0.832	0.766	0.853	0.779
17	0.960	0.835	0.763	0.853	0.779
18	0.968	0.817	0.763	0.849	0.773
19	0.962	0.817	0.763	0.847	0.771
20	0.962	0.840	0.787	0.863	0.794
21	0.962	0.822	0.778	0.854	0.780
22	0.965	0.814	0.775	0.851	0.776
23	0.954	0.806	0.766	0.842	0.762
24	0.965	0.806	0.763	0.844	0.766
25	0.960	0.791	0.743	0.830	0.745
26	0.962	0.786	0.772	0.839	0.758
27	0.962	0.796	0.778	0.844	0.767
28	0.960	0.778	0.787	0.840	0.760
29	0.960	0.786	0.799	0.846	0.770
30	0.960	0.773	0.784	0.837	0.756

31	0.965	0.773	0.787	0.840	0.760
32	0.962	0.778	0.781	0.839	0.759
33	0.962	0.788	0.778	0.842	0.762
34	0.962	0.786	0.769	0.838	0.757
35	0.965	0.778	0.754	0.831	0.747
36	0.965	0.770	0.778	0.836	0.754
37	0.962	0.783	0.763	0.835	0.752
38	0.962	0.804	0.766	0.843	0.765
39	0.965	0.806	0.778	0.849	0.773
40	0.968	0.798	0.784	0.849	0.774
41	0.965	0.811	0.775	0.850	0.775
42	0.965	0.806	0.790	0.853	0.779
43	0.962	0.798	0.781	0.846	0.769
44	0.962	0.809	0.805	0.858	0.786
45	0.962	0.814	0.790	0.855	0.782
46	0.965	0.804	0.790	0.852	0.778
47	0.962	0.806	0.790	0.852	0.778
48	0.965	0.801	0.781	0.848	0.772
49	0.965	0.809	0.781	0.851	0.776
50	0.968	0.801	0.787	0.851	0.777
51	0.965	0.798	0.790	0.850	0.775
52	0.965	0.804	0.781	0.849	0.774
53	0.968	0.804	0.781	0.850	0.775
54	0.971	0.806	0.778	0.851	0.776
55	0.968	0.806	0.787	0.853	0.779
56	0.968	0.809	0.787	0.854	0.781
57	0.968	0.811	0.787	0.855	0.782
58	0.968	0.806	0.793	0.855	0.782
59	0.968	0.806	0.787	0.853	0.779
60	0.968	0.809	0.790	0.855	0.782
61	0.968	0.809	0.787	0.854	0.781
62	0.968	0.811	0.787	0.855	0.782
63	0.971	0.817	0.796	0.860	0.790
64	0.971	0.809	0.805	0.860	0.791
65	0.971	0.819	0.808	0.865	0.798
66	0.971	0.819	0.808	0.865	0.798
67	0.971	0.822	0.793	0.861	0.792
68	0.968	0.819	0.799	0.861	0.792
69	0.974	0.817	0.814	0.867	0.801
70	0.971	0.811	0.817	0.865	0.798
71	0.971	0.814	0.817	0.866	0.799
72	0.968	0.806	0.814	0.861	0.792

73	0.968	0.804	0.820	0.862	0.794
74	0.971	0.801	0.811	0.859	0.789
75	0.968	0.806	0.799	0.857	0.785
76	0.965	0.814	0.796	0.858	0.786
77	0.965	0.811	0.799	0.858	0.786
78	0.962	0.814	0.802	0.858	0.788
79	0.962	0.809	0.802	0.857	0.785
80	0.962	0.811	0.805	0.858	0.788
81	0.962	0.811	0.802	0.858	0.786
82	0.962	0.819	0.805	0.861	0.792
83	0.965	0.837	0.805	0.869	0.803
84	0.965	0.840	0.808	0.871	0.806
85	0.965	0.837	0.805	0.869	0.803
86	0.965	0.835	0.805	0.868	0.802
87	0.971	0.819	0.805	0.864	0.796
88	0.971	0.819	0.808	0.865	0.798
89	0.971	0.814	0.808	0.863	0.795
90	0.968	0.837	0.802	0.869	0.803
91	0.968	0.837	0.802	0.869	0.803
92	0.968	0.835	0.808	0.870	0.804
93	0.968	0.835	0.808	0.870	0.804
94	0.968	0.827	0.805	0.866	0.799
95	0.968	0.829	0.805	0.867	0.800
96	0.968	0.819	0.805	0.863	0.795
97	0.968	0.824	0.802	0.864	0.796
98	0.968	0.827	0.802	0.865	0.797
99	0.971	0.822	0.811	0.867	0.800
100	0.971	0.811	0.811	0.863	0.795
101	0.974	0.817	0.817	0.868	0.802
102	0.968	0.814	0.808	0.862	0.793
103	0.968	0.822	0.799	0.862	0.793
104	0.965	0.817	0.796	0.858	0.788
105	0.965	0.811	0.796	0.857	0.785
106	0.965	0.824	0.796	0.861	0.792
107	0.968	0.819	0.799	0.861	0.792
108	0.965	0.822	0.799	0.861	0.792
109	0.965	0.819	0.799	0.860	0.790
110	0.962	0.814	0.787	0.854	0.781
111	0.962	0.829	0.802	0.864	0.796
112	0.962	0.817	0.805	0.860	0.791
113	0.962	0.819	0.805	0.861	0.792
114	0.968	0.809	0.805	0.859	0.789

115	0.968	0.809	0.805	0.859	0.789
116	0.968	0.817	0.802	0.861	0.792
117	0.974	0.827	0.808	0.869	0.803
118	0.962	0.827	0.814	0.867	0.800
119	0.965	0.827	0.814	0.868	0.802
120	0.965	0.827	0.817	0.869	0.803
121	0.965	0.827	0.817	0.869	0.803
122	0.962	0.827	0.817	0.868	0.802
123	0.962	0.827	0.817	0.868	0.802
124	0.960	0.827	0.811	0.865	0.797
125	0.960	0.827	0.814	0.866	0.799
126	0.960	0.827	0.814	0.866	0.799
127	0.960	0.827	0.817	0.867	0.800
128	0.960	0.835	0.817	0.870	0.804
129	0.960	0.840	0.808	0.869	0.803
130	0.960	0.837	0.808	0.868	0.802
131	0.951	0.824	0.817	0.863	0.795
132	0.951	0.824	0.817	0.863	0.795
133	0.962	0.814	0.808	0.860	0.791
134	0.962	0.814	0.811	0.861	0.792
135	0.962	0.817	0.811	0.862	0.793
136	0.965	0.811	0.826	0.866	0.799
137	0.962	0.811	0.823	0.864	0.797
138	0.962	0.811	0.826	0.865	0.798
139	0.962	0.811	0.817	0.862	0.794
140	0.965	0.819	0.820	0.867	0.801
141	0.965	0.819	0.820	0.867	0.801
142	0.968	0.824	0.814	0.868	0.802
143	0.965	0.824	0.820	0.869	0.803
144	0.965	0.824	0.805	0.864	0.796
145	0.965	0.824	0.808	0.865	0.798
146	0.971	0.824	0.814	0.869	0.803
147	0.971	0.824	0.814	0.869	0.803
148	0.971	0.824	0.814	0.869	0.803
149	0.971	0.829	0.820	0.873	0.809
150	0.971	0.829	0.817	0.872	0.807
151	0.968	0.829	0.811	0.869	0.803
152	0.968	0.822	0.811	0.866	0.799
153	0.968	0.822	0.811	0.866	0.799
154	0.968	0.822	0.814	0.867	0.800
155	0.968	0.822	0.814	0.867	0.800
156	0.968	0.822	0.814	0.867	0.800

157	0.968	0.827	0.814	0.869	0.803
158	0.971	0.822	0.814	0.868	0.802
159	0.971	0.827	0.811	0.869	0.803
160	0.971	0.827	0.811	0.869	0.803
161	0.971	0.822	0.814	0.868	0.802
162	0.968	0.837	0.817	0.873	0.810
163	0.968	0.832	0.808	0.869	0.803
164	0.968	0.829	0.811	0.869	0.803
165	0.968	0.832	0.811	0.870	0.804
166	0.968	0.829	0.811	0.869	0.803
167	0.968	0.832	0.811	0.870	0.804
168	0.965	0.824	0.808	0.865	0.798
169	0.965	0.822	0.808	0.864	0.796
170	0.965	0.822	0.808	0.864	0.796
171	0.965	0.822	0.805	0.863	0.795
172	0.965	0.824	0.805	0.864	0.796
173	0.965	0.824	0.823	0.870	0.805
174	0.965	0.822	0.823	0.869	0.803
175	0.965	0.822	0.820	0.868	0.802
176	0.965	0.819	0.820	0.867	0.801
177	0.965	0.819	0.820	0.867	0.801
178	0.965	0.819	0.814	0.865	0.798
179	0.974	0.837	0.814	0.874	0.812
180	0.974	0.842	0.811	0.875	0.813
181	0.974	0.850	0.808	0.877	0.816
182	0.968	0.837	0.820	0.874	0.812
183	0.968	0.837	0.820	0.874	0.812
184	0.968	0.829	0.820	0.872	0.807
185	0.968	0.835	0.820	0.873	0.810
186	0.968	0.837	0.817	0.873	0.810
187	0.968	0.832	0.814	0.871	0.806
188	0.968	0.835	0.817	0.873	0.809
189	0.968	0.832	0.820	0.873	0.809
190	0.968	0.835	0.817	0.873	0.809
191	0.968	0.837	0.823	0.875	0.813
192	0.968	0.835	0.817	0.873	0.809
193	0.971	0.832	0.817	0.873	0.809
194	0.968	0.829	0.820	0.872	0.807
195	0.968	0.842	0.823	0.877	0.816
196	0.971	0.848	0.823	0.880	0.820
197	0.971	0.848	0.823	0.880	0.820
198	0.971	0.850	0.832	0.884	0.826

199	0.974	0.853	0.835	0.887	0.830
200	0.974	0.848	0.835	0.885	0.827
201	0.974	0.848	0.835	0.885	0.827
202	0.974	0.855	0.829	0.886	0.828
203	0.974	0.845	0.832	0.883	0.824
204	0.974	0.845	0.832	0.883	0.824
205	0.974	0.845	0.829	0.882	0.823
206	0.974	0.845	0.829	0.882	0.823
207	0.974	0.848	0.826	0.882	0.823
208	0.974	0.842	0.829	0.881	0.822
209	0.974	0.848	0.826	0.882	0.823
210	0.974	0.853	0.832	0.886	0.828
211	0.974	0.853	0.838	0.888	0.831
212	0.974	0.850	0.838	0.887	0.830
213	0.974	0.853	0.823	0.883	0.824
214	0.974	0.853	0.826	0.884	0.826
215	0.974	0.853	0.826	0.884	0.826
216	0.974	0.842	0.826	0.880	0.820
217	0.974	0.840	0.823	0.878	0.817
218	0.974	0.837	0.823	0.877	0.816
219	0.974	0.837	0.823	0.877	0.816
220	0.974	0.837	0.823	0.877	0.816
221	0.974	0.837	0.823	0.877	0.816
222	0.974	0.837	0.820	0.876	0.814
223	0.974	0.840	0.820	0.877	0.816
224	0.974	0.842	0.820	0.878	0.817
225	0.974	0.845	0.820	0.879	0.819
226	0.974	0.842	0.817	0.877	0.816
227	0.974	0.842	0.820	0.878	0.817
228	0.971	0.840	0.814	0.874	0.812
229	0.971	0.837	0.823	0.876	0.815
230	0.971	0.840	0.823	0.877	0.816
231	0.971	0.840	0.820	0.876	0.814
232	0.971	0.840	0.817	0.875	0.813
233	0.971	0.840	0.820	0.876	0.814
234	0.971	0.837	0.820	0.875	0.813
235	0.971	0.840	0.820	0.876	0.814
236	0.971	0.840	0.820	0.876	0.814
237	0.971	0.840	0.817	0.875	0.813
238	0.971	0.837	0.814	0.873	0.810
239	0.971	0.837	0.817	0.874	0.812
240	0.971	0.840	0.817	0.875	0.813

241	0.977	0.850	0.817	0.881	0.821
242	0.977	0.850	0.817	0.881	0.821
243	0.977	0.855	0.823	0.885	0.827
244	0.977	0.850	0.820	0.882	0.823
245	0.977	0.850	0.820	0.882	0.823
246	0.977	0.850	0.823	0.883	0.824
247	0.977	0.850	0.823	0.883	0.824
248	0.977	0.855	0.820	0.884	0.826
249	0.977	0.850	0.823	0.883	0.824
250	0.977	0.848	0.826	0.883	0.824
251	0.977	0.850	0.826	0.884	0.826
252	0.974	0.850	0.823	0.882	0.823
253	0.974	0.848	0.823	0.881	0.821
254	0.974	0.850	0.823	0.882	0.823
255	0.974	0.850	0.823	0.882	0.823
256	0.974	0.842	0.823	0.879	0.819
257	0.974	0.842	0.823	0.879	0.819
258	0.977	0.842	0.823	0.880	0.820
259	0.977	0.848	0.817	0.880	0.820
260	0.977	0.853	0.820	0.883	0.824
261	0.977	0.848	0.820	0.881	0.821
262	0.977	0.848	0.820	0.881	0.821
263	0.977	0.853	0.817	0.882	0.823
264	0.971	0.860	0.817	0.883	0.824
265	0.971	0.860	0.817	0.883	0.824
266	0.971	0.860	0.817	0.883	0.824
267	0.971	0.863	0.811	0.882	0.823
268	0.971	0.863	0.811	0.882	0.823
269	0.971	0.863	0.814	0.883	0.824
270	0.971	0.868	0.820	0.887	0.830
271	0.971	0.868	0.814	0.885	0.827
272	0.971	0.868	0.820	0.887	0.830
273	0.971	0.868	0.820	0.887	0.830
274	0.971	0.868	0.823	0.888	0.831
275	0.971	0.868	0.823	0.888	0.831
276	0.974	0.850	0.826	0.883	0.824
277	0.974	0.855	0.823	0.884	0.825
278	0.974	0.853	0.823	0.883	0.824
279	0.974	0.853	0.829	0.885	0.827
280	0.974	0.848	0.829	0.883	0.824
281	0.974	0.853	0.826	0.884	0.826
282	0.974	0.853	0.826	0.884	0.826

283	0.974	0.850	0.826	0.883	0.824
284	0.974	0.850	0.826	0.883	0.824
285	0.974	0.853	0.829	0.885	0.827
286	0.974	0.853	0.829	0.885	0.827
287	0.977	0.853	0.814	0.881	0.821
288	0.977	0.858	0.826	0.887	0.830
289	0.977	0.855	0.826	0.886	0.828
290	0.977	0.858	0.826	0.887	0.830
291	0.977	0.858	0.826	0.887	0.830
292	0.980	0.858	0.829	0.888	0.833
293	0.980	0.860	0.826	0.888	0.833
294	0.980	0.860	0.826	0.888	0.833
295	0.980	0.858	0.823	0.887	0.830
296	0.974	0.858	0.832	0.888	0.831
297	0.974	0.858	0.832	0.888	0.831
298	0.974	0.858	0.832	0.888	0.831
299	0.974	0.858	0.829	0.887	0.830
300	0.980	0.860	0.841	0.893	0.840
301	0.980	0.860	0.841	0.893	0.840
302	0.977	0.855	0.835	0.888	0.833
303	0.977	0.853	0.838	0.888	0.833
304	0.977	0.853	0.835	0.888	0.831
305	0.977	0.853	0.838	0.888	0.833
306	0.977	0.853	0.838	0.888	0.833
307	0.977	0.853	0.838	0.888	0.833
308	0.977	0.855	0.838	0.889	0.834
309	0.977	0.855	0.838	0.889	0.834
310	0.977	0.853	0.838	0.888	0.833
311	0.977	0.853	0.838	0.888	0.833
312	0.977	0.853	0.838	0.888	0.833
313	0.977	0.853	0.838	0.888	0.833
314	0.977	0.853	0.838	0.888	0.833
315	0.977	0.853	0.841	0.889	0.834
316	0.977	0.853	0.841	0.889	0.834
317	0.977	0.848	0.841	0.888	0.831
318	0.977	0.853	0.832	0.887	0.830
319	0.968	0.863	0.838	0.889	0.834
320	0.974	0.863	0.838	0.891	0.837
321	0.974	0.863	0.838	0.891	0.837
322	0.974	0.866	0.838	0.892	0.838
323	0.974	0.860	0.838	0.890	0.835
324	0.974	0.860	0.841	0.891	0.837

325	0.974	0.860	0.841	0.891	0.837
326	0.974	0.863	0.841	0.892	0.838
327	0.971	0.866	0.841	0.892	0.838
328	0.971	0.863	0.841	0.891	0.837
329	0.971	0.863	0.841	0.891	0.837
330	0.971	0.863	0.841	0.891	0.837
331	0.971	0.868	0.844	0.894	0.841
332	0.971	0.868	0.844	0.894	0.841
333	0.971	0.868	0.844	0.894	0.841
334	0.971	0.868	0.844	0.894	0.841
335	0.971	0.866	0.841	0.892	0.838
336	0.971	0.871	0.841	0.894	0.841
337	0.971	0.871	0.841	0.894	0.841
338	0.980	0.855	0.841	0.891	0.837
339	0.980	0.858	0.832	0.889	0.834
340	0.980	0.858	0.832	0.889	0.834
341	0.980	0.850	0.829	0.886	0.828
342	0.980	0.850	0.835	0.888	0.831
343	0.980	0.848	0.832	0.886	0.828
344	0.980	0.848	0.832	0.886	0.828
345	0.980	0.845	0.835	0.886	0.828
346	0.980	0.842	0.835	0.885	0.827
347	0.980	0.845	0.832	0.885	0.827
348	0.980	0.840	0.829	0.882	0.823
349	0.980	0.845	0.832	0.885	0.827
350	0.980	0.845	0.832	0.885	0.827
351	0.980	0.845	0.832	0.885	0.827
352	0.980	0.845	0.832	0.885	0.827
353	0.980	0.845	0.832	0.885	0.827
354	0.980	0.848	0.835	0.887	0.830
355	0.980	0.845	0.835	0.886	0.828
356	0.980	0.842	0.832	0.884	0.826
357	0.980	0.845	0.832	0.885	0.827
358	0.980	0.845	0.835	0.886	0.828
359	0.980	0.840	0.832	0.883	0.824
360	0.980	0.840	0.835	0.884	0.826
361	0.980	0.840	0.823	0.880	0.820
362	0.980	0.842	0.826	0.882	0.823
363	0.980	0.845	0.835	0.886	0.828
364	0.980	0.848	0.838	0.888	0.831
365	0.980	0.848	0.838	0.888	0.831
366	0.980	0.845	0.838	0.887	0.830

367	0.980	0.848	0.844	0.889	0.834
368	0.980	0.848	0.844	0.889	0.834
369	0.980	0.848	0.844	0.889	0.834
370	0.980	0.848	0.844	0.889	0.834
371	0.980	0.848	0.844	0.889	0.834
372	0.980	0.848	0.844	0.889	0.834
373	0.980	0.858	0.844	0.893	0.840
374	0.980	0.858	0.844	0.893	0.840
375	0.980	0.860	0.844	0.894	0.841
376	0.980	0.860	0.844	0.894	0.841
377	0.980	0.868	0.835	0.894	0.841
378	0.980	0.868	0.835	0.894	0.841
379	0.980	0.866	0.835	0.893	0.839
380	0.980	0.866	0.835	0.893	0.839
381	0.980	0.871	0.841	0.897	0.845
382	0.980	0.871	0.844	0.898	0.847
383	0.980	0.871	0.844	0.898	0.847
384	0.980	0.873	0.844	0.899	0.848
385	0.983	0.871	0.841	0.898	0.847
386	0.983	0.868	0.841	0.897	0.845
387	0.980	0.876	0.835	0.897	0.845
388	0.980	0.876	0.835	0.897	0.845
389	0.980	0.876	0.835	0.897	0.845
390	0.983	0.876	0.838	0.899	0.848
391	0.983	0.868	0.841	0.897	0.845
392	0.980	0.863	0.841	0.894	0.841
393	0.980	0.863	0.841	0.894	0.841
394	0.980	0.863	0.841	0.894	0.841
395	0.980	0.860	0.838	0.892	0.838
396	0.980	0.873	0.838	0.897	0.845
397	0.980	0.873	0.838	0.897	0.845
398	0.980	0.873	0.838	0.897	0.845
399	0.980	0.873	0.838	0.897	0.845
400	0.980	0.876	0.838	0.898	0.847
401	0.980	0.879	0.835	0.898	0.847
402	0.977	0.879	0.835	0.897	0.845
403	0.977	0.881	0.835	0.898	0.846
404	0.977	0.881	0.835	0.898	0.846
405	0.977	0.881	0.835	0.898	0.846
406	0.977	0.881	0.835	0.898	0.846
407	0.977	0.876	0.835	0.896	0.844
408	0.977	0.876	0.835	0.896	0.844

409	0.977	0.876	0.835	0.896	0.844
410	0.977	0.876	0.835	0.896	0.844
411	0.977	0.876	0.835	0.896	0.844
412	0.977	0.879	0.838	0.898	0.846
413	0.977	0.873	0.838	0.896	0.844
414	0.977	0.873	0.838	0.896	0.844
415	0.980	0.881	0.838	0.900	0.849
416	0.980	0.881	0.838	0.900	0.849
417	0.980	0.881	0.838	0.900	0.849
418	0.980	0.879	0.838	0.899	0.848
419	0.980	0.879	0.838	0.899	0.848
420	0.980	0.876	0.838	0.898	0.847
421	0.980	0.879	0.838	0.899	0.848
422	0.980	0.876	0.838	0.898	0.847
423	0.980	0.879	0.838	0.899	0.848
424	0.980	0.879	0.838	0.899	0.848
425	0.980	0.863	0.841	0.894	0.841
426	0.980	0.863	0.844	0.895	0.842
427	0.980	0.866	0.841	0.895	0.842
428	0.980	0.866	0.844	0.896	0.844
429	0.980	0.866	0.844	0.896	0.844
430	0.980	0.873	0.853	0.902	0.852
431	0.980	0.871	0.853	0.901	0.851
432	0.980	0.871	0.853	0.901	0.851
433	0.980	0.871	0.853	0.901	0.851
434	0.980	0.871	0.853	0.901	0.851
435	0.980	0.873	0.850	0.901	0.851
436	0.980	0.873	0.850	0.901	0.851
437	0.980	0.873	0.850	0.901	0.851
438	0.980	0.871	0.850	0.900	0.849
439	0.980	0.871	0.850	0.900	0.849
440	0.980	0.876	0.847	0.901	0.851
441	0.980	0.873	0.847	0.900	0.849
442	0.980	0.873	0.847	0.900	0.849
443	0.980	0.873	0.847	0.900	0.849
444	0.980	0.873	0.847	0.900	0.849
445	0.980	0.876	0.844	0.900	0.849
446	0.980	0.873	0.844	0.899	0.848
447	0.980	0.868	0.850	0.899	0.848
448	0.980	0.868	0.850	0.899	0.848
449	0.980	0.871	0.850	0.900	0.849
450	0.980	0.871	0.850	0.900	0.849

451	0.980	0.868	0.850	0.899	0.848
452	0.980	0.868	0.850	0.899	0.848
453	0.980	0.871	0.853	0.901	0.851
454	0.980	0.868	0.850	0.899	0.848
455	0.980	0.866	0.850	0.898	0.847
456	0.980	0.866	0.850	0.898	0.847
457	0.980	0.866	0.850	0.898	0.847
458	0.980	0.866	0.850	0.898	0.847
459	0.980	0.866	0.850	0.898	0.847
460	0.980	0.866	0.850	0.898	0.847
461	0.983	0.868	0.847	0.899	0.848
462	0.983	0.871	0.850	0.901	0.851
463	0.983	0.866	0.850	0.899	0.848
464	0.983	0.879	0.850	0.903	0.855
465	0.983	0.876	0.853	0.903	0.855
466	0.983	0.876	0.853	0.903	0.855
467	0.983	0.876	0.853	0.903	0.855
468	0.983	0.884	0.862	0.909	0.863
469	0.983	0.884	0.862	0.909	0.863
470	0.983	0.884	0.862	0.909	0.863
471	0.983	0.884	0.862	0.909	0.863
472	0.983	0.884	0.862	0.909	0.863
473	0.983	0.884	0.862	0.909	0.863
474	0.983	0.881	0.865	0.909	0.864
475	0.983	0.884	0.862	0.909	0.863
476	0.983	0.884	0.862	0.909	0.863
477	0.983	0.884	0.862	0.909	0.863
478	0.983	0.884	0.862	0.909	0.863
479	0.983	0.886	0.862	0.910	0.865
480	0.983	0.886	0.862	0.910	0.865
481	0.983	0.886	0.862	0.910	0.865
482	0.983	0.886	0.865	0.911	0.866
483	0.983	0.884	0.865	0.910	0.865
484	0.983	0.886	0.865	0.911	0.866
485	0.983	0.884	0.865	0.910	0.865
486	0.983	0.884	0.865	0.910	0.865
487	0.983	0.889	0.859	0.910	0.865
488	0.983	0.889	0.859	0.910	0.865
489	0.983	0.889	0.856	0.909	0.863
490	0.983	0.886	0.871	0.913	0.869
491	0.983	0.881	0.859	0.907	0.861
492	0.983	0.884	0.865	0.910	0.865

493	0.983	0.894	0.862	0.913	0.869
494	0.983	0.894	0.859	0.912	0.868
495	0.983	0.889	0.865	0.912	0.868
496	0.983	0.889	0.859	0.910	0.865
497	0.983	0.886	0.862	0.910	0.865
498	0.983	0.889	0.865	0.912	0.868
499	0.983	0.886	0.865	0.911	0.866
500	0.983	0.886	0.865	0.911	0.866
501	0.983	0.886	0.865	0.911	0.866
502	0.983	0.886	0.865	0.911	0.866
503	0.983	0.886	0.865	0.911	0.866
504	0.983	0.886	0.865	0.911	0.866
505	0.983	0.889	0.865	0.912	0.868
506	0.983	0.889	0.865	0.912	0.868
507	0.986	0.891	0.862	0.913	0.869
508	0.986	0.889	0.871	0.915	0.872
509	0.986	0.889	0.871	0.915	0.872
510	0.986	0.889	0.871	0.915	0.872
511	0.986	0.889	0.868	0.914	0.870
512	0.986	0.889	0.868	0.914	0.870
513	0.986	0.889	0.868	0.914	0.870
514	0.986	0.886	0.871	0.914	0.871
515	0.986	0.889	0.871	0.915	0.872
516	0.986	0.889	0.868	0.914	0.870
517	0.986	0.886	0.874	0.915	0.872
518	0.986	0.889	0.874	0.916	0.873
519	0.986	0.889	0.874	0.916	0.873
520	0.986	0.886	0.874	0.915	0.872
521	0.986	0.889	0.874	0.916	0.873
522	0.986	0.889	0.874	0.916	0.873
523	0.986	0.889	0.874	0.916	0.873
524	0.986	0.889	0.874	0.916	0.873
525	0.986	0.889	0.874	0.916	0.873
526	0.986	0.894	0.877	0.918	0.878
527	0.986	0.894	0.877	0.918	0.878
528	0.986	0.894	0.877	0.918	0.878
529	0.983	0.889	0.877	0.916	0.873
530	0.983	0.886	0.874	0.914	0.871
531	0.983	0.886	0.874	0.914	0.871
532	0.983	0.886	0.874	0.914	0.871
533	0.983	0.886	0.871	0.913	0.869
534	0.983	0.886	0.868	0.912	0.868

535	0.983	0.886	0.868	0.912	0.868
536	0.983	0.886	0.871	0.913	0.869
537	0.986	0.886	0.871	0.914	0.871
538	0.983	0.884	0.871	0.912	0.868
539	0.986	0.886	0.868	0.913	0.869
540	0.986	0.886	0.871	0.914	0.871
541	0.986	0.884	0.868	0.912	0.868
542	0.986	0.886	0.868	0.913	0.869
543	0.983	0.886	0.868	0.912	0.868
544	0.983	0.886	0.865	0.911	0.866
545	0.986	0.886	0.862	0.911	0.866
546	0.988	0.886	0.865	0.913	0.869
547	0.988	0.886	0.862	0.912	0.868
548	0.988	0.886	0.862	0.912	0.868
549	0.988	0.884	0.865	0.912	0.868
550	0.988	0.884	0.865	0.912	0.868
551	0.988	0.884	0.865	0.912	0.868
552	0.988	0.879	0.865	0.910	0.865
553	0.988	0.876	0.862	0.908	0.862
554	0.988	0.879	0.862	0.909	0.864
555	0.988	0.879	0.862	0.909	0.864
556	0.986	0.879	0.862	0.908	0.862
557	0.986	0.879	0.862	0.908	0.862
558	0.988	0.876	0.862	0.908	0.862
559	0.986	0.873	0.865	0.907	0.861
560	0.986	0.876	0.868	0.909	0.864
561	0.986	0.876	0.868	0.909	0.864
562	0.983	0.873	0.862	0.905	0.858
563	0.983	0.873	0.862	0.905	0.858
564	0.983	0.873	0.862	0.905	0.858
565	0.983	0.876	0.856	0.904	0.856
566	0.983	0.876	0.856	0.904	0.856
567	0.986	0.876	0.856	0.905	0.858
568	0.986	0.876	0.856	0.905	0.858
569	0.986	0.876	0.856	0.905	0.858
570	0.986	0.876	0.853	0.904	0.856
571	0.986	0.879	0.853	0.905	0.858
572	0.986	0.879	0.853	0.905	0.858
573	0.986	0.871	0.847	0.901	0.851
574	0.986	0.868	0.847	0.900	0.849
575	0.986	0.873	0.850	0.903	0.854
576	0.983	0.876	0.856	0.904	0.856

577	0.983	0.876	0.856	0.904	0.856
578	0.983	0.873	0.856	0.903	0.855
579	0.986	0.873	0.856	0.904	0.857
580	0.986	0.873	0.856	0.904	0.857
581	0.988	0.873	0.856	0.905	0.858
582	0.988	0.873	0.856	0.905	0.858
583	0.986	0.876	0.862	0.907	0.861
584	0.986	0.876	0.862	0.907	0.861
585	0.983	0.876	0.853	0.903	0.855
586	0.983	0.876	0.853	0.903	0.855
587	0.983	0.876	0.853	0.903	0.855
588	0.983	0.876	0.853	0.903	0.855
589	0.983	0.876	0.856	0.904	0.856
590	0.983	0.876	0.853	0.903	0.855
591	0.983	0.876	0.853	0.903	0.855
592	0.983	0.876	0.853	0.903	0.855
593	0.983	0.876	0.853	0.903	0.855
594	0.983	0.876	0.853	0.903	0.855
595	0.983	0.876	0.853	0.903	0.855
596	0.983	0.876	0.853	0.903	0.855
597	0.983	0.876	0.853	0.903	0.855
598	0.983	0.876	0.853	0.903	0.855
599	0.983	0.876	0.853	0.903	0.855
600	0.983	0.876	0.853	0.903	0.855
601	0.983	0.876	0.853	0.903	0.855
602	0.983	0.876	0.853	0.903	0.855
603	0.983	0.876	0.853	0.903	0.855
604	0.983	0.876	0.853	0.903	0.855
605	0.983	0.876	0.853	0.903	0.855
606	0.983	0.876	0.853	0.903	0.855
607	0.983	0.876	0.853	0.903	0.855
608	0.983	0.876	0.853	0.903	0.855
609	0.983	0.879	0.850	0.903	0.855
610	0.983	0.876	0.850	0.903	0.854
611	0.983	0.876	0.850	0.903	0.854
612	0.983	0.876	0.850	0.903	0.854
613	0.983	0.873	0.850	0.902	0.852
614	0.983	0.873	0.856	0.903	0.855
615	0.983	0.873	0.859	0.904	0.856
616	0.983	0.873	0.859	0.904	0.856
617	0.983	0.873	0.859	0.904	0.856
618	0.983	0.873	0.859	0.904	0.856

619	0.983	0.873	0.859	0.904	0.856
620	0.983	0.873	0.862	0.905	0.858
621	0.983	0.873	0.862	0.905	0.858
622	0.983	0.873	0.862	0.905	0.858
623	0.983	0.873	0.859	0.904	0.856
624	0.983	0.873	0.859	0.904	0.856
625	0.983	0.873	0.859	0.904	0.856
626	0.983	0.876	0.862	0.906	0.859
627	0.983	0.876	0.859	0.905	0.858
628	0.983	0.881	0.859	0.907	0.861
629	0.983	0.881	0.859	0.907	0.861
630	0.983	0.879	0.859	0.906	0.859
631	0.983	0.879	0.862	0.907	0.861
632	0.983	0.879	0.862	0.907	0.861
633	0.983	0.881	0.862	0.908	0.862
634	0.983	0.881	0.862	0.908	0.862
635	0.983	0.881	0.862	0.908	0.862
636	0.983	0.881	0.862	0.908	0.862
637	0.983	0.881	0.862	0.908	0.862
638	0.983	0.881	0.862	0.908	0.862
639	0.983	0.873	0.859	0.904	0.856
640	0.986	0.876	0.859	0.906	0.859
641	0.986	0.879	0.862	0.908	0.862
642	0.986	0.879	0.862	0.908	0.862
643	0.983	0.879	0.862	0.907	0.861
644	0.983	0.879	0.862	0.907	0.861
645	0.983	0.879	0.862	0.907	0.861
646	0.986	0.879	0.859	0.907	0.861
647	0.986	0.879	0.859	0.907	0.861
648	0.986	0.879	0.859	0.907	0.861
649	0.986	0.879	0.859	0.907	0.861
650	0.986	0.879	0.856	0.906	0.859
651	0.986	0.879	0.859	0.907	0.861
652	0.986	0.879	0.853	0.905	0.858
653	0.986	0.879	0.856	0.906	0.859
654	0.986	0.879	0.856	0.906	0.859
655	0.986	0.879	0.856	0.906	0.859
656	0.986	0.879	0.856	0.906	0.859
657	0.986	0.879	0.856	0.906	0.859
658	0.986	0.879	0.859	0.907	0.861
659	0.986	0.879	0.859	0.907	0.861
660	0.986	0.879	0.856	0.906	0.859

661	0.986	0.879	0.856	0.906	0.859
662	0.986	0.879	0.853	0.905	0.858
663	0.986	0.884	0.853	0.907	0.861
664	0.986	0.884	0.853	0.907	0.861
665	0.986	0.884	0.853	0.907	0.861
666	0.986	0.881	0.853	0.906	0.859
667	0.986	0.884	0.853	0.907	0.861
668	0.986	0.881	0.853	0.906	0.859
669	0.986	0.884	0.853	0.907	0.861
670	0.986	0.884	0.847	0.905	0.858
671	0.980	0.889	0.856	0.908	0.862
672	0.980	0.889	0.856	0.908	0.862
673	0.980	0.889	0.856	0.908	0.862
674	0.986	0.889	0.850	0.908	0.862
675	0.983	0.889	0.847	0.906	0.859
676	0.983	0.889	0.847	0.906	0.859
677	0.983	0.889	0.847	0.906	0.859
678	0.983	0.889	0.847	0.906	0.859
679	0.983	0.889	0.847	0.906	0.859
680	0.983	0.889	0.847	0.906	0.859
681	0.983	0.889	0.847	0.906	0.859
682	0.983	0.889	0.847	0.906	0.859
683	0.983	0.889	0.847	0.906	0.859
684	0.983	0.889	0.847	0.906	0.859
685	0.983	0.889	0.847	0.906	0.859
686	0.983	0.889	0.847	0.906	0.859
687	0.980	0.889	0.847	0.905	0.858
688	0.980	0.886	0.847	0.904	0.856
689	0.980	0.884	0.847	0.903	0.855
690	0.980	0.886	0.847	0.904	0.856
691	0.980	0.884	0.847	0.903	0.855
692	0.977	0.881	0.850	0.903	0.854
693	0.977	0.879	0.850	0.902	0.852
694	0.977	0.876	0.853	0.902	0.852
695	0.977	0.876	0.853	0.902	0.852
696	0.977	0.876	0.853	0.902	0.852
697	0.980	0.876	0.856	0.903	0.855
698	0.980	0.876	0.856	0.903	0.855
699	0.980	0.876	0.856	0.903	0.855
700	0.980	0.876	0.856	0.903	0.855
701	0.980	0.876	0.856	0.903	0.855
702	0.980	0.876	0.856	0.903	0.855

703	0.980	0.876	0.856	0.903	0.855
704	0.980	0.876	0.856	0.903	0.855
705	0.980	0.876	0.856	0.903	0.855
706	0.980	0.876	0.853	0.903	0.854
707	0.980	0.876	0.853	0.903	0.854
708	0.980	0.876	0.853	0.903	0.854
709	0.980	0.876	0.853	0.903	0.854
710	0.977	0.879	0.853	0.903	0.854
711	0.977	0.879	0.853	0.903	0.854
712	0.977	0.879	0.853	0.903	0.854
713	0.977	0.881	0.853	0.903	0.855
714	0.977	0.881	0.853	0.903	0.855
715	0.977	0.881	0.853	0.903	0.855
716	0.977	0.884	0.853	0.904	0.856
717	0.977	0.879	0.853	0.903	0.854
718	0.977	0.881	0.853	0.903	0.855
719	0.977	0.881	0.853	0.903	0.855
720	0.977	0.881	0.853	0.903	0.855
721	0.977	0.881	0.853	0.903	0.855
722	0.977	0.881	0.853	0.903	0.855
723	0.977	0.881	0.853	0.903	0.855
724	0.977	0.886	0.853	0.905	0.858
725	0.977	0.889	0.853	0.906	0.859
726	0.977	0.889	0.853	0.906	0.859
727	0.977	0.886	0.853	0.905	0.858
728	0.977	0.884	0.853	0.904	0.856
729	0.977	0.886	0.853	0.905	0.858
730	0.977	0.886	0.853	0.905	0.858
731	0.977	0.886	0.853	0.905	0.858
732	0.977	0.881	0.850	0.903	0.854
733	0.977	0.884	0.850	0.903	0.855
734	0.977	0.884	0.850	0.903	0.855
735	0.977	0.884	0.850	0.903	0.855
736	0.977	0.884	0.850	0.903	0.855
737	0.977	0.884	0.850	0.903	0.855
738	0.974	0.881	0.847	0.901	0.851
739	0.974	0.881	0.847	0.901	0.851
740	0.974	0.881	0.847	0.901	0.851
741	0.974	0.881	0.847	0.901	0.851
742	0.974	0.886	0.847	0.903	0.854
743	0.974	0.884	0.847	0.902	0.852
744	0.974	0.889	0.847	0.903	0.855

745	0.974	0.886	0.847	0.903	0.854
746	0.974	0.881	0.847	0.901	0.851
747	0.971	0.886	0.850	0.903	0.854
748	0.971	0.886	0.850	0.903	0.854
749	0.971	0.886	0.850	0.903	0.854
750	0.971	0.886	0.847	0.902	0.852
751	0.971	0.886	0.847	0.902	0.852
752	0.971	0.884	0.847	0.901	0.851
753	0.971	0.886	0.847	0.902	0.852
754	0.971	0.886	0.847	0.902	0.852
755	0.971	0.889	0.850	0.903	0.855
756	0.971	0.889	0.850	0.903	0.855
757	0.971	0.889	0.847	0.903	0.853
758	0.971	0.889	0.847	0.903	0.853
759	0.971	0.889	0.847	0.903	0.853
760	0.971	0.889	0.847	0.903	0.853
761	0.971	0.889	0.847	0.903	0.853
762	0.971	0.889	0.847	0.903	0.853
763	0.971	0.889	0.844	0.902	0.852
764	0.971	0.889	0.844	0.902	0.852
765	0.971	0.889	0.844	0.902	0.852
766	0.971	0.889	0.844	0.902	0.852
767	0.971	0.889	0.844	0.902	0.852
768	0.971	0.889	0.844	0.902	0.852
769	0.971	0.889	0.844	0.902	0.852
770	0.971	0.889	0.844	0.902	0.852
771	0.971	0.889	0.847	0.903	0.853
772	0.971	0.891	0.844	0.903	0.854
773	0.971	0.891	0.844	0.903	0.854
774	0.971	0.891	0.844	0.903	0.854
775	0.971	0.891	0.844	0.903	0.854
776	0.971	0.891	0.844	0.903	0.854
777	0.971	0.891	0.844	0.903	0.854
778	0.971	0.891	0.844	0.903	0.854
779	0.971	0.891	0.844	0.903	0.854
780	0.971	0.891	0.844	0.903	0.854
781	0.971	0.891	0.844	0.903	0.854
782	0.971	0.891	0.844	0.903	0.854
783	0.971	0.891	0.844	0.903	0.854
784	0.971	0.891	0.844	0.903	0.854
785	0.971	0.891	0.844	0.903	0.854
786	0.971	0.891	0.844	0.903	0.854

787	0.971	0.889	0.844	0.902	0.852
788	0.974	0.889	0.847	0.903	0.855
**SHAP by lightGBM**					
**Numbers of features**	**G1**	**G2/M**	**S**	**ACC**	**MCC**
1	0.942	0.682	0.689	0.769	0.654
2	0.971	0.612	0.847	0.802	0.716
3	0.971	0.628	0.838	0.805	0.718
4	0.971	0.778	0.829	0.857	0.787
5	0.965	0.780	0.829	0.856	0.785
6	0.974	0.814	0.802	0.862	0.794
7	0.960	0.832	0.799	0.863	0.795
8	0.962	0.873	0.775	0.872	0.807
9	0.962	0.876	0.778	0.873	0.810
10	0.968	0.886	0.802	0.887	0.830
11	0.968	0.868	0.805	0.881	0.821
12	0.962	0.829	0.772	0.855	0.782
13	0.960	0.837	0.766	0.855	0.782
14	0.960	0.837	0.790	0.862	0.793
15	0.960	0.850	0.784	0.865	0.797
16	0.960	0.855	0.787	0.868	0.801
17	0.968	0.889	0.781	0.881	0.821
18	0.968	0.889	0.790	0.884	0.825
19	0.974	0.879	0.784	0.880	0.820
20	0.968	0.884	0.784	0.880	0.820
21	0.974	0.884	0.796	0.886	0.828
22	0.971	0.881	0.823	0.892	0.838
23	0.971	0.881	0.826	0.893	0.839
24	0.957	0.855	0.823	0.878	0.817
25	0.974	0.868	0.835	0.892	0.838
26	0.971	0.863	0.838	0.890	0.835
27	0.968	0.863	0.835	0.888	0.832
28	0.971	0.876	0.829	0.892	0.838
29	0.968	0.868	0.811	0.883	0.824
30	0.968	0.863	0.817	0.883	0.824
31	0.974	0.866	0.832	0.890	0.835
32	0.977	0.871	0.826	0.891	0.837
33	0.974	0.873	0.829	0.892	0.838
34	0.962	0.871	0.811	0.882	0.823
35	0.960	0.871	0.826	0.886	0.828
36	0.962	0.879	0.823	0.888	0.832
37	0.968	0.871	0.835	0.891	0.837
38	0.974	0.853	0.826	0.884	0.826

39	0.971	0.863	0.832	0.888	0.833
40	0.974	0.866	0.826	0.888	0.832
41	0.974	0.860	0.823	0.886	0.828
42	0.971	0.860	0.835	0.888	0.833
43	0.974	0.860	0.826	0.887	0.830
44	0.974	0.858	0.847	0.892	0.838
45	0.974	0.858	0.850	0.893	0.840
46	0.974	0.863	0.850	0.895	0.843
47	0.965	0.860	0.823	0.883	0.824
48	0.968	0.858	0.832	0.886	0.828
49	0.965	0.860	0.841	0.888	0.833
50	0.968	0.855	0.829	0.884	0.826
51	0.968	0.850	0.832	0.883	0.824
52	0.968	0.855	0.829	0.884	0.826
53	0.974	0.850	0.835	0.886	0.828
54	0.974	0.850	0.835	0.886	0.828
55	0.965	0.860	0.841	0.888	0.833
56	0.971	0.866	0.838	0.891	0.837
57	0.968	0.855	0.844	0.888	0.833
58	0.968	0.863	0.841	0.890	0.835
59	0.977	0.853	0.829	0.886	0.828
60	0.977	0.855	0.832	0.888	0.831
61	0.977	0.866	0.820	0.888	0.831
62	0.977	0.868	0.820	0.888	0.833
63	0.965	0.863	0.841	0.889	0.834
64	0.965	0.863	0.841	0.889	0.834
65	0.965	0.868	0.841	0.891	0.837
66	0.965	0.871	0.838	0.891	0.837
67	0.965	0.873	0.841	0.893	0.839
68	0.962	0.871	0.847	0.893	0.840
69	0.962	0.871	0.847	0.893	0.840
70	0.980	0.871	0.847	0.899	0.848
71	0.974	0.868	0.835	0.892	0.838
72	0.980	0.866	0.838	0.894	0.841
73	0.977	0.868	0.832	0.892	0.838
74	0.977	0.871	0.838	0.895	0.842
75	0.977	0.871	0.856	0.901	0.851
76	0.977	0.868	0.850	0.898	0.847
77	0.980	0.868	0.856	0.901	0.851
78	0.977	0.868	0.865	0.903	0.854
79	0.977	0.873	0.859	0.903	0.854
80	0.977	0.876	0.853	0.902	0.852

81	0.977	0.868	0.859	0.901	0.851
82	0.971	0.863	0.856	0.896	0.844
83	0.974	0.866	0.859	0.899	0.848
84	0.974	0.866	0.859	0.899	0.848
85	0.971	0.866	0.859	0.898	0.847
86	0.974	0.871	0.865	0.903	0.854
87	0.974	0.876	0.865	0.904	0.857
88	0.968	0.858	0.832	0.886	0.828
89	0.968	0.858	0.832	0.886	0.828
90	0.968	0.860	0.826	0.885	0.827
91	0.968	0.860	0.826	0.885	0.827
92	0.968	0.866	0.823	0.886	0.828
93	0.968	0.863	0.823	0.885	0.827
94	0.971	0.863	0.820	0.885	0.827
95	0.971	0.863	0.826	0.887	0.830
96	0.971	0.860	0.823	0.885	0.827
97	0.971	0.863	0.829	0.888	0.831
98	0.971	0.866	0.829	0.888	0.832
99	0.971	0.866	0.829	0.888	0.832
100	0.971	0.858	0.829	0.886	0.828
101	0.971	0.855	0.841	0.888	0.833
102	0.974	0.853	0.838	0.888	0.831
103	0.971	0.858	0.844	0.890	0.835
104	0.974	0.860	0.844	0.892	0.838
105	0.974	0.855	0.835	0.888	0.831
106	0.974	0.850	0.850	0.890	0.836
107	0.974	0.850	0.850	0.890	0.836
108	0.974	0.848	0.847	0.888	0.833
109	0.974	0.848	0.847	0.888	0.833
110	0.977	0.858	0.844	0.892	0.838
111	0.977	0.863	0.856	0.898	0.847
112	0.977	0.855	0.856	0.895	0.843
113	0.974	0.858	0.856	0.895	0.843
114	0.974	0.858	0.853	0.894	0.841
115	0.974	0.860	0.856	0.896	0.844
116	0.977	0.871	0.859	0.902	0.852
117	0.977	0.871	0.859	0.902	0.852
118	0.974	0.876	0.853	0.901	0.851
119	0.971	0.884	0.850	0.902	0.852
120	0.971	0.884	0.853	0.903	0.854
121	0.971	0.879	0.853	0.901	0.851
122	0.971	0.879	0.853	0.901	0.851

123	0.971	0.876	0.859	0.902	0.852
124	0.968	0.868	0.868	0.901	0.851
125	0.968	0.866	0.868	0.900	0.850
126	0.968	0.868	0.868	0.901	0.851
127	0.968	0.866	0.862	0.898	0.847
128	0.968	0.873	0.862	0.901	0.851
129	0.968	0.879	0.850	0.899	0.848
130	0.968	0.876	0.856	0.900	0.849
131	0.968	0.873	0.859	0.900	0.849
132	0.968	0.873	0.859	0.900	0.849
133	0.971	0.886	0.859	0.905	0.858
134	0.968	0.879	0.862	0.903	0.854
135	0.974	0.879	0.859	0.903	0.855
136	0.974	0.881	0.862	0.905	0.858
137	0.974	0.881	0.859	0.904	0.856
138	0.974	0.884	0.856	0.904	0.856
139	0.974	0.884	0.862	0.906	0.859
140	0.974	0.889	0.862	0.908	0.862
141	0.974	0.886	0.862	0.907	0.861
142	0.974	0.889	0.862	0.908	0.862
143	0.971	0.891	0.862	0.908	0.862
144	0.971	0.894	0.865	0.910	0.865
145	0.971	0.884	0.859	0.904	0.856
146	0.971	0.881	0.856	0.903	0.854
147	0.971	0.881	0.856	0.903	0.854
148	0.971	0.881	0.856	0.903	0.854
149	0.968	0.881	0.862	0.903	0.855
150	0.968	0.881	0.862	0.903	0.855
151	0.974	0.884	0.868	0.908	0.862
152	0.974	0.886	0.868	0.909	0.863
153	0.974	0.884	0.868	0.908	0.862
154	0.971	0.884	0.868	0.907	0.861
155	0.965	0.886	0.868	0.906	0.859
156	0.968	0.884	0.868	0.906	0.859
157	0.965	0.884	0.868	0.905	0.858
158	0.971	0.881	0.877	0.909	0.864
159	0.968	0.879	0.877	0.907	0.861
160	0.965	0.881	0.877	0.907	0.861
161	0.965	0.886	0.874	0.908	0.862
162	0.965	0.884	0.871	0.906	0.859
163	0.962	0.891	0.874	0.909	0.863
164	0.962	0.891	0.874	0.909	0.863

165	0.962	0.886	0.874	0.907	0.861
166	0.962	0.884	0.874	0.906	0.859
167	0.965	0.886	0.859	0.903	0.855
168	0.965	0.886	0.859	0.903	0.855
169	0.965	0.886	0.859	0.903	0.855
170	0.965	0.886	0.859	0.903	0.855
171	0.968	0.897	0.868	0.911	0.866
172	0.968	0.899	0.865	0.911	0.866
173	0.968	0.904	0.862	0.912	0.868
174	0.968	0.904	0.859	0.911	0.866
175	0.968	0.902	0.868	0.913	0.869
176	0.968	0.902	0.868	0.913	0.869
177	0.968	0.902	0.868	0.913	0.869
178	0.968	0.897	0.859	0.908	0.862
179	0.968	0.894	0.853	0.905	0.858
180	0.965	0.897	0.853	0.905	0.858
181	0.962	0.899	0.859	0.907	0.861
182	0.962	0.899	0.865	0.909	0.863
183	0.962	0.899	0.865	0.909	0.863
184	0.962	0.899	0.859	0.907	0.861
185	0.962	0.902	0.868	0.911	0.866
186	0.962	0.902	0.865	0.910	0.865
187	0.962	0.902	0.865	0.910	0.865
188	0.962	0.899	0.862	0.908	0.862
189	0.962	0.899	0.862	0.908	0.862
190	0.965	0.894	0.862	0.907	0.861
191	0.962	0.894	0.862	0.906	0.859
192	0.962	0.894	0.862	0.906	0.859
193	0.965	0.894	0.859	0.906	0.859
194	0.965	0.894	0.859	0.906	0.859
195	0.965	0.894	0.862	0.907	0.861
196	0.962	0.894	0.868	0.908	0.862
197	0.962	0.894	0.868	0.908	0.862
198	0.968	0.884	0.871	0.907	0.861
199	0.968	0.881	0.871	0.906	0.859
200	0.971	0.879	0.865	0.904	0.857
201	0.971	0.879	0.865	0.904	0.857
202	0.968	0.884	0.859	0.903	0.855
203	0.974	0.873	0.865	0.903	0.855
204	0.974	0.873	0.865	0.903	0.855
205	0.974	0.873	0.865	0.903	0.855
206	0.974	0.876	0.868	0.905	0.858

207	0.974	0.876	0.868	0.905	0.858
208	0.974	0.876	0.868	0.905	0.858
209	0.974	0.876	0.868	0.905	0.858
210	0.974	0.873	0.877	0.907	0.861
211	0.974	0.876	0.877	0.908	0.862
212	0.974	0.876	0.877	0.908	0.862
213	0.974	0.873	0.874	0.906	0.859
214	0.974	0.873	0.874	0.906	0.859
215	0.974	0.871	0.871	0.904	0.857
216	0.974	0.873	0.871	0.905	0.858
217	0.974	0.873	0.874	0.906	0.859
218	0.974	0.879	0.877	0.909	0.864
219	0.974	0.881	0.877	0.910	0.865
220	0.980	0.884	0.883	0.915	0.872
221	0.980	0.884	0.880	0.914	0.871
222	0.980	0.886	0.886	0.917	0.875
223	0.980	0.886	0.886	0.917	0.875
224	0.980	0.886	0.886	0.917	0.875
225	0.977	0.881	0.871	0.909	0.864
226	0.977	0.881	0.865	0.907	0.861
227	0.977	0.881	0.865	0.907	0.861
228	0.977	0.881	0.868	0.908	0.862
229	0.977	0.881	0.868	0.908	0.862
230	0.977	0.881	0.868	0.908	0.862
231	0.977	0.884	0.868	0.909	0.863
232	0.977	0.879	0.868	0.907	0.861
233	0.977	0.879	0.868	0.907	0.861
234	0.965	0.871	0.865	0.900	0.849
235	0.965	0.879	0.865	0.903	0.854
236	0.962	0.879	0.865	0.902	0.852
237	0.965	0.876	0.868	0.903	0.854
238	0.965	0.876	0.868	0.903	0.854
239	0.965	0.876	0.868	0.903	0.854
240	0.968	0.879	0.862	0.903	0.854
241	0.968	0.884	0.862	0.904	0.856
242	0.968	0.881	0.862	0.903	0.855
243	0.968	0.881	0.862	0.903	0.855
244	0.968	0.881	0.862	0.903	0.855
245	0.968	0.881	0.862	0.903	0.855
246	0.968	0.881	0.862	0.903	0.855
247	0.968	0.881	0.862	0.903	0.855
248	0.968	0.876	0.865	0.903	0.854

249	0.968	0.876	0.865	0.903	0.854
250	0.968	0.876	0.868	0.903	0.855
251	0.968	0.876	0.868	0.903	0.855
252	0.968	0.876	0.868	0.903	0.855
253	0.968	0.873	0.868	0.903	0.854
254	0.968	0.873	0.865	0.902	0.852
255	0.968	0.876	0.868	0.903	0.855
256	0.968	0.873	0.868	0.903	0.854
257	0.968	0.873	0.868	0.903	0.854
258	0.971	0.876	0.865	0.903	0.855
259	0.974	0.879	0.862	0.904	0.856
260	0.971	0.871	0.862	0.901	0.851
261	0.971	0.871	0.862	0.901	0.851
262	0.971	0.871	0.862	0.901	0.851
263	0.971	0.868	0.862	0.900	0.849
264	0.971	0.866	0.862	0.899	0.848
265	0.971	0.868	0.862	0.900	0.849
266	0.971	0.868	0.862	0.900	0.849
267	0.971	0.868	0.862	0.900	0.849
268	0.971	0.866	0.862	0.899	0.848
269	0.971	0.866	0.862	0.899	0.848
270	0.968	0.871	0.862	0.900	0.849
271	0.968	0.871	0.856	0.898	0.847
272	0.968	0.876	0.856	0.900	0.849
273	0.968	0.876	0.853	0.899	0.848
274	0.965	0.876	0.877	0.905	0.858
275	0.965	0.873	0.877	0.904	0.857
276	0.965	0.873	0.877	0.904	0.857
277	0.971	0.881	0.877	0.909	0.864
278	0.971	0.879	0.877	0.908	0.862
279	0.971	0.879	0.877	0.908	0.862
280	0.971	0.879	0.877	0.908	0.862
281	0.971	0.879	0.877	0.908	0.862
282	0.971	0.876	0.877	0.907	0.861
283	0.971	0.879	0.877	0.908	0.862
284	0.971	0.879	0.877	0.908	0.862
285	0.971	0.879	0.877	0.908	0.862
286	0.971	0.879	0.877	0.908	0.862
287	0.971	0.876	0.877	0.907	0.861
288	0.971	0.881	0.880	0.910	0.865
289	0.971	0.881	0.880	0.910	0.865
290	0.983	0.879	0.877	0.912	0.868

291	0.983	0.879	0.877	0.912	0.868
292	0.980	0.876	0.874	0.909	0.864
293	0.980	0.879	0.877	0.911	0.866
294	0.980	0.879	0.877	0.911	0.866
295	0.980	0.879	0.877	0.911	0.866
296	0.980	0.873	0.871	0.907	0.861
297	0.977	0.876	0.871	0.907	0.861
298	0.977	0.873	0.871	0.906	0.859
299	0.977	0.873	0.871	0.906	0.859
300	0.977	0.873	0.871	0.906	0.859
301	0.977	0.873	0.871	0.906	0.859
302	0.974	0.873	0.871	0.905	0.858
303	0.974	0.876	0.871	0.906	0.859
304	0.974	0.876	0.871	0.906	0.859
305	0.974	0.876	0.874	0.907	0.861
306	0.974	0.876	0.874	0.907	0.861
307	0.971	0.879	0.874	0.907	0.861
308	0.971	0.879	0.874	0.907	0.861
309	0.971	0.876	0.874	0.906	0.859
310	0.971	0.879	0.874	0.907	0.861
311	0.971	0.879	0.874	0.907	0.861
312	0.971	0.879	0.874	0.907	0.861
313	0.971	0.879	0.859	0.903	0.854
314	0.971	0.879	0.859	0.903	0.854
315	0.971	0.879	0.859	0.903	0.854
316	0.971	0.881	0.859	0.903	0.855
317	0.971	0.879	0.856	0.902	0.852
318	0.971	0.884	0.865	0.906	0.859
319	0.971	0.884	0.862	0.905	0.858
320	0.971	0.884	0.862	0.905	0.858
321	0.971	0.886	0.862	0.906	0.859
322	0.974	0.881	0.865	0.906	0.859
323	0.974	0.884	0.865	0.907	0.861
324	0.986	0.884	0.865	0.911	0.866
325	0.986	0.884	0.865	0.911	0.866
326	0.986	0.884	0.862	0.910	0.865
327	0.986	0.889	0.862	0.912	0.868
328	0.986	0.889	0.862	0.912	0.868
329	0.986	0.889	0.862	0.912	0.868
330	0.986	0.886	0.862	0.911	0.866
331	0.986	0.886	0.862	0.911	0.866
332	0.983	0.889	0.862	0.911	0.866

333	0.983	0.889	0.862	0.911	0.866
334	0.983	0.889	0.862	0.911	0.866
335	0.983	0.886	0.856	0.908	0.862
336	0.983	0.884	0.856	0.907	0.861
337	0.983	0.886	0.859	0.909	0.863
338	0.983	0.884	0.859	0.908	0.862
339	0.983	0.884	0.859	0.908	0.862
340	0.983	0.876	0.859	0.905	0.858
341	0.983	0.873	0.856	0.903	0.855
342	0.983	0.879	0.856	0.905	0.858
343	0.983	0.881	0.862	0.908	0.862
344	0.983	0.876	0.862	0.906	0.859
345	0.986	0.873	0.859	0.905	0.858
346	0.986	0.871	0.859	0.904	0.857
347	0.986	0.881	0.859	0.908	0.862
348	0.986	0.881	0.868	0.911	0.866
349	0.986	0.881	0.868	0.911	0.866
350	0.986	0.884	0.868	0.912	0.868
351	0.986	0.884	0.868	0.912	0.868
352	0.986	0.884	0.868	0.912	0.868
353	0.986	0.881	0.868	0.911	0.866
354	0.986	0.879	0.865	0.909	0.864
355	0.986	0.881	0.865	0.910	0.865
356	0.986	0.881	0.865	0.910	0.865
357	0.986	0.884	0.865	0.911	0.866
358	0.986	0.879	0.859	0.907	0.861
359	0.986	0.876	0.859	0.906	0.859
360	0.986	0.876	0.859	0.906	0.859
361	0.986	0.876	0.862	0.907	0.861
362	0.986	0.876	0.862	0.907	0.861
363	0.986	0.873	0.865	0.907	0.861
364	0.986	0.873	0.862	0.906	0.859
365	0.986	0.871	0.862	0.905	0.858
366	0.986	0.871	0.862	0.905	0.858
367	0.986	0.871	0.862	0.905	0.858
368	0.986	0.868	0.862	0.904	0.857
369	0.986	0.871	0.862	0.905	0.858
370	0.986	0.876	0.868	0.909	0.864
371	0.986	0.876	0.868	0.909	0.864
372	0.986	0.873	0.868	0.908	0.862
373	0.986	0.873	0.868	0.908	0.862
374	0.986	0.873	0.868	0.908	0.862

375	0.986	0.873	0.868	0.908	0.862
376	0.986	0.873	0.868	0.908	0.862
377	0.986	0.873	0.868	0.908	0.862
378	0.986	0.873	0.868	0.908	0.862
379	0.986	0.873	0.868	0.908	0.862
380	0.986	0.871	0.874	0.909	0.864
381	0.986	0.871	0.874	0.909	0.864
382	0.986	0.871	0.874	0.909	0.864
383	0.986	0.871	0.874	0.909	0.864
384	0.986	0.871	0.874	0.909	0.864
385	0.986	0.871	0.871	0.908	0.862
386	0.986	0.871	0.871	0.908	0.862
387	0.986	0.871	0.871	0.908	0.862
388	0.986	0.873	0.871	0.909	0.864
389	0.986	0.868	0.871	0.907	0.861
390	0.986	0.868	0.871	0.907	0.861
391	0.986	0.868	0.871	0.907	0.861
392	0.986	0.868	0.871	0.907	0.861
393	0.986	0.868	0.871	0.907	0.861
394	0.986	0.868	0.871	0.907	0.861
395	0.986	0.868	0.871	0.907	0.861
396	0.986	0.868	0.871	0.907	0.861
397	0.986	0.868	0.871	0.907	0.861
398	0.983	0.894	0.856	0.911	0.866
399	0.983	0.886	0.862	0.910	0.865
400	0.983	0.886	0.862	0.910	0.865
401	0.983	0.889	0.862	0.911	0.866
402	0.983	0.889	0.862	0.911	0.866
403	0.983	0.891	0.862	0.912	0.868
404	0.983	0.889	0.862	0.911	0.866
405	0.983	0.889	0.862	0.911	0.866
406	0.986	0.886	0.859	0.910	0.865
407	0.986	0.886	0.859	0.910	0.865
408	0.986	0.886	0.859	0.910	0.865
409	0.986	0.886	0.865	0.912	0.868
410	0.986	0.886	0.865	0.912	0.868
411	0.986	0.886	0.865	0.912	0.868
412	0.986	0.886	0.865	0.912	0.868
413	0.986	0.886	0.865	0.912	0.868
414	0.986	0.886	0.862	0.911	0.866
415	0.986	0.886	0.862	0.911	0.866
416	0.986	0.886	0.862	0.911	0.866

417	0.986	0.886	0.868	0.913	0.869
418	0.986	0.886	0.868	0.913	0.869
419	0.986	0.886	0.865	0.912	0.868
420	0.986	0.891	0.868	0.915	0.872
421	0.986	0.891	0.865	0.914	0.871
422	0.986	0.889	0.865	0.913	0.869
423	0.986	0.889	0.865	0.913	0.869
424	0.986	0.889	0.865	0.913	0.869
425	0.986	0.889	0.865	0.913	0.869
426	0.986	0.886	0.865	0.912	0.868
427	0.986	0.884	0.865	0.911	0.866
428	0.986	0.881	0.862	0.909	0.864
429	0.986	0.881	0.862	0.909	0.864
430	0.986	0.873	0.862	0.906	0.859
431	0.986	0.873	0.862	0.906	0.859
432	0.986	0.873	0.862	0.906	0.859
433	0.986	0.873	0.862	0.906	0.859
434	0.986	0.873	0.862	0.906	0.859
435	0.986	0.873	0.862	0.906	0.859
436	0.986	0.873	0.862	0.906	0.859
437	0.986	0.873	0.862	0.906	0.859
438	0.986	0.873	0.862	0.906	0.859
439	0.986	0.873	0.862	0.906	0.859
440	0.986	0.873	0.862	0.906	0.859
441	0.986	0.873	0.859	0.905	0.858
442	0.986	0.873	0.859	0.905	0.858
443	0.986	0.873	0.859	0.905	0.858
444	0.986	0.876	0.859	0.906	0.859
445	0.983	0.881	0.859	0.907	0.861
446	0.983	0.881	0.856	0.906	0.859
447	0.986	0.884	0.853	0.907	0.861
448	0.986	0.884	0.850	0.906	0.859
449	0.986	0.884	0.850	0.906	0.859
450	0.986	0.884	0.850	0.906	0.859
451	0.986	0.884	0.850	0.906	0.859
452	0.986	0.884	0.853	0.907	0.861
453	0.986	0.884	0.853	0.907	0.861
454	0.986	0.884	0.853	0.907	0.861
455	0.986	0.884	0.853	0.907	0.861
456	0.986	0.884	0.853	0.907	0.861
457	0.986	0.879	0.859	0.907	0.861
458	0.986	0.879	0.859	0.907	0.861

459	0.983	0.881	0.862	0.908	0.862
460	0.983	0.884	0.865	0.910	0.865
461	0.983	0.884	0.865	0.910	0.865
462	0.983	0.886	0.862	0.910	0.865
463	0.983	0.886	0.865	0.911	0.866
464	0.983	0.886	0.865	0.911	0.866
465	0.980	0.881	0.862	0.907	0.861
466	0.980	0.881	0.862	0.907	0.861
467	0.983	0.884	0.862	0.909	0.863
468	0.983	0.884	0.859	0.908	0.862
469	0.983	0.884	0.859	0.908	0.862
470	0.983	0.884	0.859	0.908	0.862
471	0.983	0.884	0.859	0.908	0.862
472	0.983	0.884	0.862	0.909	0.863
473	0.983	0.886	0.862	0.910	0.865
474	0.983	0.886	0.862	0.910	0.865
475	0.983	0.886	0.862	0.910	0.865
476	0.983	0.886	0.865	0.911	0.866
477	0.980	0.876	0.862	0.905	0.858
478	0.980	0.879	0.871	0.909	0.864
479	0.980	0.879	0.868	0.908	0.862
480	0.980	0.881	0.868	0.909	0.864
481	0.980	0.884	0.868	0.910	0.865
482	0.980	0.884	0.868	0.910	0.865
483	0.980	0.884	0.868	0.910	0.865
484	0.980	0.884	0.868	0.910	0.865
485	0.980	0.889	0.859	0.909	0.863
486	0.980	0.889	0.859	0.909	0.863
487	0.980	0.889	0.859	0.909	0.863
488	0.980	0.886	0.856	0.907	0.861
489	0.980	0.886	0.856	0.907	0.861
490	0.980	0.886	0.859	0.908	0.862
491	0.980	0.886	0.859	0.908	0.862
492	0.980	0.884	0.859	0.907	0.861
493	0.980	0.884	0.859	0.907	0.861
494	0.980	0.884	0.859	0.907	0.861
495	0.980	0.884	0.856	0.906	0.859
496	0.980	0.884	0.856	0.906	0.859
497	0.980	0.886	0.856	0.907	0.861
498	0.980	0.886	0.856	0.907	0.861
499	0.980	0.879	0.856	0.904	0.856
500	0.980	0.884	0.856	0.906	0.859

501	0.980	0.884	0.856	0.906	0.859
502	0.980	0.881	0.856	0.905	0.858
503	0.983	0.884	0.856	0.907	0.861
504	0.983	0.884	0.856	0.907	0.861
505	0.980	0.884	0.856	0.906	0.859
506	0.980	0.886	0.856	0.907	0.861
507	0.986	0.884	0.847	0.905	0.858
508	0.986	0.884	0.847	0.905	0.858
509	0.986	0.884	0.847	0.905	0.858
510	0.986	0.879	0.847	0.903	0.855
511	0.986	0.889	0.844	0.906	0.859
512	0.986	0.889	0.847	0.907	0.861
513	0.986	0.886	0.847	0.906	0.859
514	0.986	0.886	0.847	0.906	0.859
515	0.986	0.886	0.844	0.905	0.858
516	0.986	0.884	0.844	0.904	0.856
517	0.986	0.884	0.847	0.905	0.858
518	0.986	0.884	0.847	0.905	0.858
519	0.986	0.884	0.847	0.905	0.858
520	0.986	0.884	0.847	0.905	0.858
521	0.986	0.884	0.847	0.905	0.858
522	0.986	0.884	0.847	0.905	0.858
523	0.986	0.884	0.844	0.904	0.856
524	0.986	0.886	0.841	0.904	0.856
525	0.986	0.886	0.844	0.905	0.858
526	0.986	0.886	0.847	0.906	0.859
527	0.986	0.886	0.844	0.905	0.858
528	0.986	0.886	0.844	0.905	0.858
529	0.986	0.886	0.847	0.906	0.859
530	0.986	0.886	0.847	0.906	0.859
531	0.986	0.884	0.844	0.904	0.856
532	0.986	0.884	0.844	0.904	0.856
533	0.986	0.884	0.844	0.904	0.856
534	0.983	0.884	0.844	0.903	0.855
535	0.986	0.881	0.850	0.905	0.858
536	0.986	0.881	0.847	0.904	0.856
537	0.986	0.884	0.847	0.905	0.858
538	0.986	0.879	0.844	0.903	0.854
539	0.986	0.879	0.844	0.903	0.854
540	0.986	0.879	0.844	0.903	0.854
541	0.986	0.879	0.847	0.903	0.855
542	0.986	0.879	0.847	0.903	0.855

543	0.986	0.879	0.844	0.903	0.854
544	0.986	0.879	0.844	0.903	0.854
545	0.983	0.881	0.850	0.904	0.856
546	0.983	0.886	0.850	0.906	0.859
547	0.983	0.886	0.850	0.906	0.859
548	0.983	0.886	0.850	0.906	0.859
549	0.983	0.886	0.850	0.906	0.859
550	0.983	0.886	0.847	0.905	0.858
551	0.980	0.884	0.841	0.902	0.852
552	0.977	0.881	0.844	0.901	0.851
553	0.977	0.884	0.841	0.901	0.851
554	0.977	0.881	0.841	0.900	0.849
555	0.977	0.884	0.841	0.901	0.851
556	0.977	0.881	0.841	0.900	0.849
557	0.977	0.886	0.838	0.901	0.851
558	0.977	0.889	0.835	0.901	0.851
559	0.977	0.886	0.832	0.899	0.848
560	0.977	0.889	0.835	0.901	0.851
561	0.977	0.886	0.838	0.901	0.851
562	0.977	0.886	0.838	0.901	0.851
563	0.977	0.886	0.835	0.900	0.849
564	0.977	0.886	0.835	0.900	0.849
565	0.977	0.886	0.835	0.900	0.849
566	0.977	0.886	0.835	0.900	0.849
567	0.977	0.886	0.835	0.900	0.849
568	0.977	0.886	0.835	0.900	0.849
569	0.971	0.886	0.847	0.902	0.852
570	0.971	0.886	0.847	0.902	0.852
571	0.971	0.889	0.847	0.903	0.854
572	0.971	0.889	0.844	0.902	0.852
573	0.971	0.889	0.844	0.902	0.852
574	0.971	0.889	0.844	0.902	0.852
575	0.971	0.889	0.844	0.902	0.852
576	0.971	0.889	0.844	0.902	0.852
577	0.971	0.889	0.841	0.901	0.851
578	0.971	0.889	0.841	0.901	0.851
579	0.971	0.889	0.841	0.901	0.851
580	0.971	0.886	0.841	0.900	0.849
581	0.971	0.889	0.841	0.901	0.851
582	0.971	0.889	0.841	0.901	0.851
583	0.971	0.886	0.841	0.900	0.849
584	0.971	0.886	0.844	0.901	0.851

585	0.971	0.886	0.844	0.901	0.851
586	0.971	0.886	0.844	0.901	0.851
587	0.971	0.886	0.844	0.901	0.851
588	0.971	0.886	0.844	0.901	0.851
589	0.971	0.886	0.844	0.901	0.851
590	0.971	0.886	0.844	0.901	0.851
591	0.971	0.886	0.844	0.901	0.851
592	0.971	0.886	0.844	0.901	0.851
593	0.971	0.886	0.844	0.901	0.851
594	0.971	0.886	0.847	0.902	0.852
595	0.971	0.886	0.841	0.900	0.849
596	0.971	0.886	0.841	0.900	0.849
597	0.971	0.886	0.841	0.900	0.849
598	0.971	0.886	0.841	0.900	0.849
599	0.971	0.886	0.841	0.900	0.849
600	0.971	0.884	0.841	0.899	0.848
601	0.971	0.897	0.847	0.905	0.858
602	0.971	0.899	0.847	0.906	0.859
603	0.971	0.899	0.847	0.906	0.859
604	0.971	0.902	0.847	0.907	0.861
605	0.971	0.902	0.847	0.907	0.861
606	0.971	0.902	0.847	0.907	0.861
607	0.971	0.902	0.847	0.907	0.861
608	0.971	0.904	0.850	0.909	0.863
609	0.971	0.904	0.850	0.909	0.863
610	0.971	0.904	0.850	0.909	0.863
611	0.974	0.902	0.850	0.909	0.863
612	0.974	0.902	0.850	0.909	0.863
613	0.974	0.902	0.850	0.909	0.863
614	0.974	0.902	0.847	0.908	0.862
615	0.974	0.902	0.850	0.909	0.863
616	0.974	0.899	0.850	0.908	0.862
617	0.974	0.899	0.850	0.908	0.862
618	0.974	0.899	0.853	0.909	0.863
619	0.974	0.897	0.850	0.907	0.861
620	0.974	0.897	0.850	0.907	0.861
621	0.974	0.897	0.850	0.907	0.861
622	0.974	0.897	0.850	0.907	0.861
623	0.974	0.897	0.850	0.907	0.861
624	0.974	0.889	0.844	0.903	0.854
625	0.974	0.889	0.844	0.903	0.854
626	0.974	0.889	0.844	0.903	0.854

627	0.974	0.894	0.844	0.904	0.856
628	0.974	0.891	0.844	0.903	0.855
629	0.974	0.891	0.844	0.903	0.855
630	0.974	0.891	0.844	0.903	0.855
631	0.974	0.889	0.844	0.903	0.854
632	0.974	0.889	0.844	0.903	0.854
633	0.974	0.889	0.844	0.903	0.854
634	0.974	0.889	0.844	0.903	0.854
635	0.974	0.889	0.844	0.903	0.854
636	0.974	0.889	0.844	0.903	0.854
637	0.974	0.889	0.844	0.903	0.854
638	0.974	0.891	0.844	0.903	0.855
639	0.974	0.889	0.841	0.902	0.852
640	0.974	0.889	0.841	0.902	0.852
641	0.974	0.886	0.841	0.901	0.851
642	0.974	0.886	0.838	0.900	0.849
643	0.974	0.886	0.841	0.901	0.851
644	0.974	0.886	0.841	0.901	0.851
645	0.974	0.886	0.841	0.901	0.851
646	0.974	0.886	0.844	0.902	0.852
647	0.974	0.886	0.841	0.901	0.851
648	0.974	0.886	0.841	0.901	0.851
649	0.974	0.886	0.841	0.901	0.851
650	0.974	0.886	0.844	0.902	0.852
651	0.974	0.886	0.844	0.902	0.852
652	0.974	0.886	0.844	0.902	0.852
653	0.974	0.889	0.844	0.903	0.854
654	0.974	0.889	0.841	0.902	0.852
655	0.974	0.889	0.844	0.903	0.854
656	0.974	0.889	0.844	0.903	0.854
657	0.974	0.897	0.847	0.906	0.859
658	0.974	0.899	0.850	0.908	0.862
659	0.974	0.894	0.841	0.903	0.855
660	0.974	0.894	0.841	0.903	0.855
661	0.974	0.894	0.841	0.903	0.855
662	0.974	0.894	0.841	0.903	0.855
663	0.974	0.894	0.841	0.903	0.855
664	0.974	0.894	0.841	0.903	0.855
665	0.974	0.894	0.841	0.903	0.855
666	0.974	0.894	0.841	0.903	0.855
667	0.974	0.894	0.841	0.903	0.855
668	0.974	0.894	0.841	0.903	0.855

669	0.974	0.894	0.841	0.903	0.855
670	0.974	0.894	0.841	0.903	0.855
671	0.974	0.894	0.841	0.903	0.855
672	0.974	0.894	0.841	0.903	0.855
673	0.974	0.894	0.841	0.903	0.855
674	0.971	0.894	0.841	0.903	0.854
675	0.971	0.894	0.841	0.903	0.854
676	0.974	0.894	0.841	0.903	0.855
677	0.974	0.894	0.841	0.903	0.855
678	0.971	0.894	0.841	0.903	0.854
679	0.971	0.894	0.841	0.903	0.854
680	0.971	0.891	0.847	0.903	0.855
681	0.971	0.891	0.847	0.903	0.855
682	0.974	0.891	0.850	0.905	0.858
683	0.974	0.891	0.850	0.905	0.858
684	0.974	0.891	0.850	0.905	0.858
685	0.971	0.889	0.850	0.903	0.855
686	0.971	0.889	0.850	0.903	0.855
687	0.971	0.886	0.850	0.903	0.854
688	0.974	0.886	0.850	0.903	0.855
689	0.971	0.886	0.850	0.903	0.854
690	0.974	0.889	0.850	0.904	0.856
691	0.974	0.889	0.850	0.904	0.856
692	0.974	0.889	0.850	0.904	0.856
693	0.974	0.889	0.850	0.904	0.856
694	0.974	0.889	0.850	0.904	0.856
695	0.974	0.889	0.853	0.905	0.858
696	0.974	0.889	0.853	0.905	0.858
697	0.974	0.889	0.853	0.905	0.858
698	0.977	0.886	0.850	0.904	0.856
699	0.977	0.889	0.853	0.906	0.859
700	0.977	0.889	0.853	0.906	0.859
701	0.977	0.889	0.853	0.906	0.859
702	0.977	0.889	0.853	0.906	0.859
703	0.977	0.889	0.853	0.906	0.859
704	0.974	0.886	0.853	0.904	0.856
705	0.974	0.886	0.853	0.904	0.856
706	0.971	0.886	0.853	0.903	0.855
707	0.971	0.886	0.853	0.903	0.855
708	0.974	0.891	0.856	0.907	0.861
709	0.974	0.891	0.856	0.907	0.861
710	0.974	0.891	0.856	0.907	0.861

711	0.974	0.891	0.856	0.907	0.861
712	0.974	0.891	0.856	0.907	0.861
713	0.974	0.891	0.856	0.907	0.861
714	0.974	0.891	0.856	0.907	0.861
715	0.974	0.891	0.856	0.907	0.861
716	0.974	0.889	0.856	0.906	0.859
717	0.974	0.889	0.856	0.906	0.859
718	0.974	0.889	0.856	0.906	0.859
719	0.974	0.891	0.850	0.905	0.858
720	0.974	0.891	0.850	0.905	0.858
721	0.974	0.891	0.850	0.905	0.858
722	0.974	0.891	0.850	0.905	0.858
723	0.974	0.891	0.850	0.905	0.858
724	0.974	0.891	0.850	0.905	0.858
725	0.974	0.891	0.853	0.906	0.859
726	0.974	0.891	0.853	0.906	0.859
727	0.974	0.891	0.853	0.906	0.859
728	0.974	0.891	0.853	0.906	0.859
729	0.974	0.891	0.853	0.906	0.859
730	0.974	0.891	0.853	0.906	0.859
731	0.974	0.891	0.853	0.906	0.859
732	0.974	0.891	0.853	0.906	0.859
733	0.974	0.891	0.853	0.906	0.859
734	0.974	0.891	0.853	0.906	0.859
735	0.974	0.891	0.853	0.906	0.859
736	0.974	0.891	0.853	0.906	0.859
737	0.974	0.891	0.853	0.906	0.859
738	0.974	0.891	0.853	0.906	0.859
739	0.974	0.891	0.853	0.906	0.859
740	0.974	0.889	0.853	0.905	0.858
741	0.974	0.889	0.850	0.904	0.856
742	0.974	0.889	0.850	0.904	0.856
743	0.974	0.889	0.850	0.904	0.856
744	0.974	0.889	0.850	0.904	0.856
745	0.974	0.889	0.850	0.904	0.856
746	0.974	0.889	0.853	0.905	0.858
747	0.974	0.889	0.853	0.905	0.858
748	0.971	0.886	0.856	0.904	0.856
749	0.971	0.886	0.856	0.904	0.856
750	0.971	0.886	0.856	0.904	0.856
751	0.971	0.886	0.856	0.904	0.856
752	0.971	0.886	0.856	0.904	0.856

753	0.971	0.886	0.856	0.904	0.856
754	0.971	0.886	0.856	0.904	0.856
755	0.974	0.889	0.850	0.904	0.856
756	0.974	0.889	0.850	0.904	0.856
757	0.974	0.889	0.850	0.904	0.856
758	0.974	0.889	0.850	0.904	0.856
759	0.974	0.889	0.850	0.904	0.856
760	0.974	0.889	0.850	0.904	0.856
761	0.974	0.889	0.850	0.904	0.856
762	0.974	0.886	0.850	0.903	0.855
763	0.974	0.889	0.850	0.904	0.856
764	0.974	0.889	0.850	0.904	0.856
765	0.974	0.889	0.850	0.904	0.856
766	0.974	0.889	0.850	0.904	0.856
767	0.974	0.889	0.850	0.904	0.856
768	0.974	0.889	0.850	0.904	0.856
769	0.974	0.889	0.850	0.904	0.856
770	0.974	0.889	0.850	0.904	0.856
771	0.974	0.889	0.850	0.904	0.856
772	0.974	0.889	0.850	0.904	0.856
773	0.974	0.889	0.850	0.904	0.856
774	0.974	0.886	0.850	0.903	0.855
775	0.974	0.889	0.850	0.904	0.856
776	0.974	0.889	0.850	0.904	0.856
777	0.974	0.889	0.850	0.904	0.856
778	0.974	0.889	0.850	0.904	0.856
779	0.974	0.889	0.850	0.904	0.856
780	0.974	0.886	0.850	0.903	0.855
781	0.974	0.886	0.850	0.903	0.855
782	0.974	0.886	0.850	0.903	0.855
783	0.974	0.886	0.850	0.903	0.855
784	0.974	0.886	0.850	0.903	0.855
785	0.974	0.889	0.847	0.903	0.855
786	0.974	0.889	0.847	0.903	0.855
787	0.974	0.889	0.847	0.903	0.855
788	0.974	0.889	0.847	0.903	0.855
