# Supplementary material for: Identification of Human Cell Cycle Phase Markers Based on Single-Cell RNA-Seq Data by Using Machine Learning Methods
Source: Biomed Res Int. 2022 Aug 13;2022:2516653. doi: 10.1155/2022/2516653 (PMC9393965; doi:10.1155/2022/2516653)
Supplement: Supplementary 3 — Table S3: feasible feature subsets selected by three feature selection methods and their intersection. [file 2516653.f3.pdf]

Table S3. Feasible feature subsets selected by three feature selection methods and their intersection.

(1) Feasible feature subsets selected by three feature selection methods

mRMR	MCFS	SHAP by LightGBM
ENSG00000170312(CDK1)	ENSG00000170312(CDK1)	ENSG00000170312(CDK1)
ENSG00000189057(FAM111B)	ENSG00000175063(UBE2C)	ENSG00000197061(H4C3)
ENSG00000112029(FBXO5)	ENSG00000197061(H4C3)	ENSG00000131652(THOC6)
ENSG00000131747(TOP2A)	ENSG00000123485(HJURP)	ENSG00000140451(PIF1)
ENSG00000166801(FAM111A)	ENSG00000131747(TOP2A)	ENSG00000175063(UBE2C)
ENSG00000115641(FHL2)	ENSG00000112029(FBXO5)	ENSG00000229089(ANKRD20A8P)
ENSG00000123485(HJURP)	ENSG00000186185(KIF18B)	ENSG00000150991(UBC)
ENSG00000171320(ESCO2)	ENSG00000101447(FAM83D)	ENSG00000189057(FAM111B)
ENSG00000131475(VPS25)	ENSG00000146670(CDCA5)	ENSG00000105173(CCNE1)
ENSG00000175063(UBE2C)	ENSG00000166801(FAM111A)	ENSG00000132646(PCNA)
ENSG00000051341(POLQ)	ENSG00000140451(PIF1)	ENSG00000101447(FAM83D)
ENSG00000112118(MCM3)	ENSG00000182481(KPNA2)	
ENSG00000178999(AURKB)	ENSG00000274997(H2AC12)	
ENSG00000198331(HYLS1)	ENSG00000169607(CKAP2L)	
ENSG00000171848(RRM2)	ENSG00000189057(FAM111B)	
ENSG00000085999(RAD54L)	ENSG00000171320(ESCO2)	
ENSG00000184661(CDCA2)	ENSG00000276368(H2AC14)	
ENSG00000177943(MAMDC4)	ENSG00000137807(KIF23)	
ENSG00000156802(ATAD2)	ENSG00000122952(ZWINT)	
ENSG00000093009(CDC45)	ENSG00000134057(CCNB1)	
ENSG00000140451(PIF1)	ENSG00000178999(AURKB)	

ENSG00000165304(MELK)		
ENSG00000197061(H4C3)		
ENSG00000186185(KIF18B)		
ENSG00000076003(MCM6)		
ENSG00000076248(UNG)		
ENSG00000146670(CDCA5)		
ENSG00000173207(CKS1B)		
ENSG00000102384(CENPI)		
ENSG00000274997(H2AC12)		
ENSG00000137807(KIF23)		
ENSG00000105011(ASF1B)		

(2) Intersection of three feasible feature subsets

Features appear in 3 methods	Features appear in 2 methods	Features appear in 1 method
ENSG00000140451(PIF1)	ENSG00000101447(FAM83D)	ENSG00000051341(POLQ)
ENSG00000170312(CDK1)	ENSG00000112029(FBXO5)	ENSG00000076003(MCM6)
ENSG00000175063(UBE2C)	ENSG00000123485(HJURP)	ENSG00000076248(UNG)
ENSG00000189057(FAM111B)	ENSG00000131747(TOP2A)	ENSG00000085999(RAD54L)
ENSG00000197061(H4C3)	ENSG00000137807(KIF23)	ENSG00000093009(CDC45)
	ENSG00000146670(CDCA5)	ENSG00000102384(CENPI)
	ENSG00000166801(FAM111A)	ENSG00000105011(ASF1B)
	ENSG00000171320(ESCO2)	ENSG00000105173(CCNE1)
	ENSG00000178999(AURKB)	ENSG00000112118(MCM3)
	ENSG00000186185(KIF18B)	ENSG00000115641(FHL2)
	ENSG00000274997(H2AC12)	ENSG00000122952(ZWINT)

		ENSG00000131475(VPS25)
		ENSG00000131652(THOC6)
		ENSG00000132646(PCNA)
		ENSG00000134057(CCNB1)
		ENSG00000150991(UBC)
		ENSG00000156802(ATAD2)
		ENSG00000165304(MELK)
		ENSG00000169607(CKAP2L)
		ENSG00000171848(RRM2)
		ENSG00000173207(CKS1B)
		ENSG00000177943(MAMDC4)
		ENSG00000182481(KPNA2)
		ENSG00000184661(CDCA2)
		ENSG00000198331(HYLS1)
		ENSG00000229089(ANKRD20A8P)
		ENSG00000276368(H2AC14)
