# Supplementary material for: Identification of Human Cell Cycle Phase Markers Based on Single-Cell RNA-Seq Data by Using Machine Learning Methods
Source: Biomed Res Int. 2022 Aug 13;2022:2516653. doi: 10.1155/2022/2516653 (PMC9393965; doi:10.1155/2022/2516653)
Supplement: Supplementary 4 — Table S4: classification rules generated by the optimal DT classifier based on the feature lists generated by three feature ranking algorithms. [file 2516653.f4.pdf]

Table S4. Classification rules generated by the optimal DT classifier based on the feature lists generated by three feature ranking algorithms

(1) Rules on features selected by mRMR

Rules_0	passed counts:314
node_0: feature_name=ENSG00000170312	feature_id[0].value <= threshold=120.56500244140625
node_1: feature_name=ENSG00000197061	feature_id[22].value <= threshold=794.760009765625
node_2: feature_name=ENSG00000091164	feature_id[113].value > threshold=29.735000610351562
node_10: feature_name=ENSG00000093009	feature_id[19].value <= threshold=198.45000457763672
node_11: feature_name=ENSG00000167553	feature_id[135].value > threshold=88.50500106811523
node_13: feature_name=ENSG00000025770	feature_id[131].value <= threshold=154.4499969482422
node_14: feature_name=ENSG00000007968	feature_id[80].value <= threshold=20.079999923706055
node_15: feature_name=ENSG00000105355	feature_id[116].value > threshold=24.335000038146973
Class: G1	
Rules_1	passed counts:227
node_0: feature_name=ENSG00000170312	feature_id[0].value > threshold=120.56500244140625
node_34: feature_name=ENSG00000140451	feature_id[20].value > threshold=8.110000133514404
node_92: feature_name=ENSG00000105173	feature_id[67].value <= threshold=98.07999801635742
node_93: feature_name=ENSG00000170312	feature_id[0].value > threshold=160.83999633789062
node_99: feature_name=ENSG00000070950	feature_id[108].value <= threshold=74.7750015258789
node_100: feature_name=ENSG00000175130	feature_id[152].value <= threshold=103.5099983215332
node_101: feature_name=ENSG00000117724	feature_id[111].value > threshold=42.60000038146973
node_115: feature_name=ENSG00000197061	feature_id[22].value > threshold=144.55500030517578
node_125: feature_name=ENSG00000122952	feature_id[57].value <= threshold=716.2649841308594
node_126: feature_name=ENSG00000198033	feature_id[149].value <= threshold=456.3699951171875

node_127: feature_name=ENSG00000178074	feature_id[96].value <= threshold=38.650001525878906
node_128: feature_name=ENSG00000006327	feature_id[95].value <= threshold=560.8050231933594
node_129: feature_name=ENSG00000175305	feature_id[49].value <= threshold=23.119999885559082
Class: G2M	
Rules_2	passed counts:187
node_0: feature_name=ENSG00000170312	feature_id[0].value > threshold=120.56500244140625
node_34: feature_name=ENSG00000140451	feature_id[20].value <= threshold=8.110000133514404
node_35: feature_name=ENSG00000100297	feature_id[54].value > threshold=111.95999908447266
node_57: feature_name=ENSG00000197061	feature_id[22].value > threshold=468.4400177001953
node_61: feature_name=ENSG00000012048	feature_id[36].value > threshold=23.5600004196167
node_73: feature_name=ENSG00000229089	feature_id[37].value <= threshold=106.125
node_74: feature_name=ENSG00000182481	feature_id[35].value <= threshold=505.8800048828125
node_75: feature_name=ENSG00000088325	feature_id[143].value > threshold=14.054999828338623
node_77: feature_name=ENSG00000088986	feature_id[83].value <= threshold=1100.9449768066406
node_78: feature_name=ENSG00000154473	feature_id[156].value > threshold=64.73999786376953
Class: S	
Rules_3	passed counts:34
node_0: feature_name=ENSG00000170312	feature_id[0].value > threshold=120.56500244140625
node_34: feature_name=ENSG00000140451	feature_id[20].value > threshold=8.110000133514404
node_92: feature_name=ENSG00000105173	feature_id[67].value <= threshold=98.07999801635742
node_93: feature_name=ENSG00000170312	feature_id[0].value > threshold=160.83999633789062
node_99: feature_name=ENSG00000070950	feature_id[108].value <= threshold=74.7750015258789
node_100: feature_name=ENSG00000175130	feature_id[152].value <= threshold=103.5099983215332

node_101: feature_name=ENSG00000117724	feature_id[111].value <= threshold=42.60000038146973
node_102: feature_name=ENSG00000128951	feature_id[123].value > threshold=160.25
node_112: feature_name=ENSG00000118193	feature_id[82].value > threshold=2.0850000381469727
Class: G2M	
Rules_4	passed counts:34
node_0: feature_name=ENSG00000170312	feature_id[0].value > threshold=120.56500244140625
node_34: feature_name=ENSG00000140451	feature_id[20].value <= threshold=8.110000133514404
node_35: feature_name=ENSG00000100297	feature_id[54].value <= threshold=111.95999908447266
node_36: feature_name=ENSG00000102384	feature_id[28].value <= threshold=15.289999961853027
node_37: feature_name=ENSG00000123473	feature_id[51].value <= threshold=74.30500030517578
node_38: feature_name=ENSG00000102362	feature_id[136].value <= threshold=11.420000076293945
node_39: feature_name=ENSG00000205352	feature_id[100].value <= threshold=243.77000427246094
node_40: feature_name=ENSG00000031691	feature_id[76].value <= threshold=47.04499912261963
Class: G2M	
Rules_5	passed counts:23
node_0: feature_name=ENSG00000170312	feature_id[0].value > threshold=120.56500244140625
node_34: feature_name=ENSG00000140451	feature_id[20].value <= threshold=8.110000133514404
node_35: feature_name=ENSG00000100297	feature_id[54].value <= threshold=111.95999908447266
node_36: feature_name=ENSG00000102384	feature_id[28].value > threshold=15.289999961853027
node_46: feature_name=ENSG00000169242	feature_id[155].value <= threshold=16.90499973297119
node_47: feature_name=ENSG00000156802	feature_id[18].value > threshold=32.334999084472656
node_51: feature_name=ENSG00000276368	feature_id[46].value <= threshold=156.26000213623047
node_52: feature_name=ENSG00000175063	feature_id[9].value > threshold=250.1449966430664

Class: S	
Rules_6	passed counts:20
node_0: feature_name=ENSG00000170312	feature_id[0].value > threshold=120.56500244140625
node_34: feature_name=ENSG00000140451	feature_id[20].value > threshold=8.110000133514404
node_92: feature_name=ENSG00000105173	feature_id[67].value > threshold=98.07999801635742
node_142: feature_name=ENSG00000173207	feature_id[27].value <= threshold=529.4750061035156
node_143: feature_name=ENSG00000112118	feature_id[11].value > threshold=74.27000045776367
Class: S	
Rules_7	passed counts:18
node_0: feature_name=ENSG00000170312	feature_id[0].value > threshold=120.56500244140625
node_34: feature_name=ENSG00000140451	feature_id[20].value > threshold=8.110000133514404
node_92: feature_name=ENSG00000105173	feature_id[67].value <= threshold=98.07999801635742
node_93: feature_name=ENSG00000170312	feature_id[0].value > threshold=160.83999633789062
node_99: feature_name=ENSG00000070950	feature_id[108].value <= threshold=74.7750015258789
node_100: feature_name=ENSG00000175130	feature_id[152].value <= threshold=103.5099983215332
node_101: feature_name=ENSG00000117724	feature_id[111].value > threshold=42.60000038146973
node_115: feature_name=ENSG00000197061	feature_id[22].value <= threshold=144.55500030517578
node_116: feature_name=ENSG00000235571	feature_id[119].value <= threshold=12.9350004196167
node_117: feature_name=ENSG00000112118	feature_id[11].value > threshold=30.804999351501465
Class: G2M	
Rules_8	passed counts:15
node_0: feature_name=ENSG00000170312	feature_id[0].value > threshold=120.56500244140625

node_34: feature_name=ENSG00000140451	feature_id[20].value > threshold=8.110000133514404
node_92: feature_name=ENSG00000105173	feature_id[67].value > threshold=98.07999801635742
node_142: feature_name=ENSG00000173207	feature_id[27].value > threshold=529.4750061035156
node_148: feature_name=ENSG00000106367	feature_id[60].value > threshold=167.65499877929688
Class: G2M	
Rules_9	passed counts:15
node_0: feature_name=ENSG00000170312	feature_id[0].value > threshold=120.56500244140625
node_34: feature_name=ENSG00000140451	feature_id[20].value > threshold=8.110000133514404
node_92: feature_name=ENSG00000105173	feature_id[67].value <= threshold=98.07999801635742
node_93: feature_name=ENSG00000170312	feature_id[0].value > threshold=160.83999633789062
node_99: feature_name=ENSG00000070950	feature_id[108].value <= threshold=74.7750015258789
node_100: feature_name=ENSG00000175130	feature_id[152].value <= threshold=103.5099983215332
node_101: feature_name=ENSG00000117724	feature_id[111].value <= threshold=42.60000038146973
node_102: feature_name=ENSG00000128951	feature_id[123].value <= threshold=160.25
node_103: feature_name=ENSG00000105011	feature_id[31].value <= threshold=61.09000015258789
node_104: feature_name=ENSG00000163808	feature_id[89].value <= threshold=9.610000133514404
node_105: feature_name=ENSG00000123975	feature_id[90].value <= threshold=1455.4949951171875
Class: G2M	
Rules_10	passed counts:14
node_0: feature_name=ENSG00000170312	feature_id[0].value > threshold=120.56500244140625
node_34: feature_name=ENSG00000140451	feature_id[20].value <= threshold=8.110000133514404
node_35: feature_name=ENSG00000100297	feature_id[54].value > threshold=111.95999908447266
node_57: feature_name=ENSG00000197061	feature_id[22].value > threshold=468.4400177001953

node_61: feature_name=ENSG00000012048	feature_id[36].value > threshold=23.5600004196167
node_73: feature_name=ENSG00000229089	feature_id[37].value <= threshold=106.125
node_74: feature_name=ENSG00000182481	feature_id[35].value > threshold=505.8800048828125
node_84: feature_name=ENSG00000146278	feature_id[64].value > threshold=0.7649999856948853
Class: S	
Rules_11	passed counts:11
node_0: feature_name=ENSG00000170312	feature_id[0].value > threshold=120.56500244140625
node_34: feature_name=ENSG00000140451	feature_id[20].value <= threshold=8.110000133514404
node_35: feature_name=ENSG00000100297	feature_id[54].value > threshold=111.95999908447266
node_57: feature_name=ENSG00000197061	feature_id[22].value > threshold=468.4400177001953
node_61: feature_name=ENSG00000012048	feature_id[36].value <= threshold=23.5600004196167
node_62: feature_name=ENSG00000135451	feature_id[43].value > threshold=42.31999969482422
node_66: feature_name=ENSG00000013573	feature_id[92].value <= threshold=54.625
node_67: feature_name=ENSG00000171793	feature_id[58].value <= threshold=282.51499938964844
Class: G2M	
Rules_12	passed counts:11
node_0: feature_name=ENSG00000170312	feature_id[0].value <= threshold=120.56500244140625
node_1: feature_name=ENSG00000197061	feature_id[22].value > threshold=794.760009765625
node_29: feature_name=ENSG00000164104	feature_id[50].value > threshold=51.71500015258789
Class: S	
Rules_13	passed counts:10
node_0: feature_name=ENSG00000170312	feature_id[0].value > threshold=120.56500244140625

node_34: feature_name=ENSG00000140451	feature_id[20].value > threshold=8.110000133514404
node_92: feature_name=ENSG00000105173	feature_id[67].value <= threshold=98.07999801635742
node_93: feature_name=ENSG00000170312	feature_id[0].value > threshold=160.83999633789062
node_99: feature_name=ENSG00000070950	feature_id[108].value <= threshold=74.7750015258789
node_100: feature_name=ENSG00000175130	feature_id[152].value <= threshold=103.5099983215332
node_101: feature_name=ENSG00000117724	feature_id[111].value <= threshold=42.60000038146973
node_102: feature_name=ENSG00000128951	feature_id[123].value <= threshold=160.25
node_103: feature_name=ENSG00000105011	feature_id[31].value > threshold=61.09000015258789
node_109: feature_name=ENSG00000136982	feature_id[112].value > threshold=17.335000038146973
Class: S	
Rules_14	passed counts:8
node_0: feature_name=ENSG00000170312	feature_id[0].value > threshold=120.56500244140625
node_34: feature_name=ENSG00000140451	feature_id[20].value <= threshold=8.110000133514404
node_35: feature_name=ENSG00000100297	feature_id[54].value > threshold=111.95999908447266
node_57: feature_name=ENSG00000197061	feature_id[22].value > threshold=468.4400177001953
node_61: feature_name=ENSG00000012048	feature_id[36].value <= threshold=23.5600004196167
node_62: feature_name=ENSG00000135451	feature_id[43].value <= threshold=42.31999969482422
node_63: feature_name=ENSG00000148773	feature_id[73].value > threshold=24.8149995803833
Class: S	
Rules_15	passed counts:7
node_0: feature_name=ENSG00000170312	feature_id[0].value > threshold=120.56500244140625
node_34: feature_name=ENSG00000140451	feature_id[20].value > threshold=8.110000133514404
node_92: feature_name=ENSG00000105173	feature_id[67].value <= threshold=98.07999801635742

node_93: feature_name=ENSG00000170312	feature_id[0].value > threshold=160.83999633789062
node_99: feature_name=ENSG00000070950	feature_id[108].value <= threshold=74.7750015258789
node_100: feature_name=ENSG00000175130	feature_id[152].value <= threshold=103.5099983215332
node_101: feature_name=ENSG00000117724	feature_id[111].value > threshold=42.60000038146973
node_115: feature_name=ENSG00000197061	feature_id[22].value <= threshold=144.55500030517578
node_116: feature_name=ENSG00000235571	feature_id[119].value > threshold=12.9350004196167
node_122: feature_name=ENSG00000142945	feature_id[86].value > threshold=156.95000457763672
Class: S	
Rules_16	passed counts:7
node_0: feature_name=ENSG00000170312	feature_id[0].value > threshold=120.56500244140625
node_34: feature_name=ENSG00000140451	feature_id[20].value <= threshold=8.110000133514404
node_35: feature_name=ENSG00000100297	feature_id[54].value > threshold=111.95999908447266
node_57: feature_name=ENSG00000197061	feature_id[22].value <= threshold=468.4400177001953
node_58: feature_name=ENSG00000127922	feature_id[94].value > threshold=402.9700012207031
Class: G1	
Rules_17	passed counts:5
node_0: feature_name=ENSG00000170312	feature_id[0].value > threshold=120.56500244140625
node_34: feature_name=ENSG00000140451	feature_id[20].value > threshold=8.110000133514404
node_92: feature_name=ENSG00000105173	feature_id[67].value <= threshold=98.07999801635742
node_93: feature_name=ENSG00000170312	feature_id[0].value <= threshold=160.83999633789062
node_94: feature_name=ENSG00000182481	feature_id[35].value > threshold=678.3799743652344
Class: G1	

Rules_18	passed counts:5
node_0: feature_name=ENSG00000170312	feature_id[0].value > threshold=120.56500244140625
node_34: feature_name=ENSG00000140451	feature_id[20].value <= threshold=8.110000133514404
node_35: feature_name=ENSG00000100297	feature_id[54].value > threshold=111.95999908447266
node_57: feature_name=ENSG00000197061	feature_id[22].value > threshold=468.4400177001953
node_61: feature_name=ENSG00000012048	feature_id[36].value > threshold=23.5600004196167
node_73: feature_name=ENSG00000229089	feature_id[37].value <= threshold=106.125
node_74: feature_name=ENSG00000182481	feature_id[35].value > threshold=505.8800048828125
node_84: feature_name=ENSG00000146278	feature_id[64].value <= threshold=0.7649999856948853
node_85: feature_name=ENSG00000025770	feature_id[131].value > threshold=91.17000198364258
Class: G2M	
Rules_19	passed counts:5
node_0: feature_name=ENSG00000170312	feature_id[0].value > threshold=120.56500244140625
node_34: feature_name=ENSG00000140451	feature_id[20].value <= threshold=8.110000133514404
node_35: feature_name=ENSG00000100297	feature_id[54].value <= threshold=111.95999908447266
node_36: feature_name=ENSG00000102384	feature_id[28].value > threshold=15.289999961853027
node_46: feature_name=ENSG00000169242	feature_id[155].value > threshold=16.90499973297119
Class: G2M	
Rules_20	passed counts:4
node_0: feature_name=ENSG00000170312	feature_id[0].value > threshold=120.56500244140625
node_34: feature_name=ENSG00000140451	feature_id[20].value > threshold=8.110000133514404
node_92: feature_name=ENSG00000105173	feature_id[67].value > threshold=98.07999801635742
node_142: feature_name=ENSG00000173207	feature_id[27].value <= threshold=529.4750061035156

node_143: feature_name=ENSG00000112118	feature_id[11].value <= threshold=74.27000045776367
node_144: feature_name=ENSG00000106211	feature_id[87].value > threshold=406.1999969482422
Class: G2M	
Rules_21	passed counts:4
node_0: feature_name=ENSG00000170312	feature_id[0].value <= threshold=120.56500244140625
node_1: feature_name=ENSG00000197061	feature_id[22].value > threshold=794.760009765625
node_29: feature_name=ENSG00000164104	feature_id[50].value <= threshold=51.71500015258789
node_30: feature_name=ENSG00000103995	feature_id[132].value > threshold=4.700000047683716
Class: G1	
Rules_22	passed counts:3
node_0: feature_name=ENSG00000170312	feature_id[0].value > threshold=120.56500244140625
node_34: feature_name=ENSG00000140451	feature_id[20].value > threshold=8.110000133514404
node_92: feature_name=ENSG00000105173	feature_id[67].value > threshold=98.07999801635742
node_142: feature_name=ENSG00000173207	feature_id[27].value > threshold=529.4750061035156
node_148: feature_name=ENSG00000106367	feature_id[60].value <= threshold=167.65499877929688
Class: S	
Rules_23	passed counts:3
node_0: feature_name=ENSG00000170312	feature_id[0].value > threshold=120.56500244140625
node_34: feature_name=ENSG00000140451	feature_id[20].value > threshold=8.110000133514404
node_92: feature_name=ENSG00000105173	feature_id[67].value <= threshold=98.07999801635742
node_93: feature_name=ENSG00000170312	feature_id[0].value > threshold=160.83999633789062
node_99: feature_name=ENSG00000070950	feature_id[108].value > threshold=74.7750015258789

Class: S	
Rules_24	passed counts:3
node_0: feature_name=ENSG00000170312	feature_id[0].value > threshold=120.56500244140625
node_34: feature_name=ENSG00000140451	feature_id[20].value > threshold=8.110000133514404
node_92: feature_name=ENSG00000105173	feature_id[67].value <= threshold=98.07999801635742
node_93: feature_name=ENSG00000170312	feature_id[0].value > threshold=160.83999633789062
node_99: feature_name=ENSG00000070950	feature_id[108].value <= threshold=74.7750015258789
node_100: feature_name=ENSG00000175130	feature_id[152].value > threshold=103.5099983215332
node_138: feature_name=ENSG00000101447	feature_id[32].value > threshold=154.69000244140625
Class: S	
Rules_25	passed counts:3
node_0: feature_name=ENSG00000170312	feature_id[0].value > threshold=120.56500244140625
node_34: feature_name=ENSG00000140451	feature_id[20].value > threshold=8.110000133514404
node_92: feature_name=ENSG00000105173	feature_id[67].value <= threshold=98.07999801635742
node_93: feature_name=ENSG00000170312	feature_id[0].value > threshold=160.83999633789062
node_99: feature_name=ENSG00000070950	feature_id[108].value <= threshold=74.7750015258789
node_100: feature_name=ENSG00000175130	feature_id[152].value <= threshold=103.5099983215332
node_101: feature_name=ENSG00000117724	feature_id[111].value <= threshold=42.60000038146973
node_102: feature_name=ENSG00000128951	feature_id[123].value <= threshold=160.25
node_103: feature_name=ENSG00000105011	feature_id[31].value <= threshold=61.09000015258789
node_104: feature_name=ENSG00000163808	feature_id[89].value > threshold=9.610000133514404
Class: S	

Rules_26	passed counts:3
node_0: feature_name=ENSG00000170312	feature_id[0].value > threshold=120.56500244140625
node_34: feature_name=ENSG00000140451	feature_id[20].value > threshold=8.110000133514404
node_92: feature_name=ENSG00000105173	feature_id[67].value <= threshold=98.07999801635742
node_93: feature_name=ENSG00000170312	feature_id[0].value <= threshold=160.83999633789062
node_94: feature_name=ENSG00000182481	feature_id[35].value <= threshold=678.3799743652344
node_95: feature_name=ENSG00000103995	feature_id[132].value > threshold=9.0
Class: S	
Rules_27	passed counts:3
node_0: feature_name=ENSG00000170312	feature_id[0].value > threshold=120.56500244140625
node_34: feature_name=ENSG00000140451	feature_id[20].value <= threshold=8.110000133514404
node_35: feature_name=ENSG00000100297	feature_id[54].value > threshold=111.95999908447266
node_57: feature_name=ENSG00000197061	feature_id[22].value > threshold=468.4400177001953
node_61: feature_name=ENSG00000012048	feature_id[36].value > threshold=23.5600004196167
node_73: feature_name=ENSG00000229089	feature_id[37].value <= threshold=106.125
node_74: feature_name=ENSG00000182481	feature_id[35].value > threshold=505.8800048828125
node_84: feature_name=ENSG00000146278	feature_id[64].value <= threshold=0.7649999856948853
node_85: feature_name=ENSG00000025770	feature_id[131].value <= threshold=91.17000198364258
node_86: feature_name=ENSG00000229089	feature_id[37].value > threshold=18.195000171661377
Class: S	
Rules_28	passed counts:3
node_0: feature_name=ENSG00000170312	feature_id[0].value > threshold=120.56500244140625
node_34: feature_name=ENSG00000140451	feature_id[20].value <= threshold=8.110000133514404

node_35: feature_name=ENSG00000100297	feature_id[54].value > threshold=111.95999908447266
node_57: feature_name=ENSG00000197061	feature_id[22].value > threshold=468.4400177001953
node_61: feature_name=ENSG00000012048	feature_id[36].value <= threshold=23.5600004196167
node_62: feature_name=ENSG00000135451	feature_id[43].value > threshold=42.31999969482422
node_66: feature_name=ENSG00000013573	feature_id[92].value > threshold=54.625
Class: S	
Rules_29	passed counts:3
node_0: feature_name=ENSG00000170312	feature_id[0].value > threshold=120.56500244140625
node_34: feature_name=ENSG00000140451	feature_id[20].value <= threshold=8.110000133514404
node_35: feature_name=ENSG00000100297	feature_id[54].value <= threshold=111.95999908447266
node_36: feature_name=ENSG00000102384	feature_id[28].value <= threshold=15.289999961853027
node_37: feature_name=ENSG00000123473	feature_id[51].value > threshold=74.30500030517578
Class: S	
Rules_30	passed counts:3
node_0: feature_name=ENSG00000170312	feature_id[0].value <= threshold=120.56500244140625
node_1: feature_name=ENSG00000197061	feature_id[22].value <= threshold=794.760009765625
node_2: feature_name=ENSG00000091164	feature_id[113].value > threshold=29.735000610351562
node_10: feature_name=ENSG00000093009	feature_id[19].value <= threshold=198.45000457763672
node_11: feature_name=ENSG00000167553	feature_id[135].value > threshold=88.50500106811523
node_13: feature_name=ENSG00000025770	feature_id[131].value <= threshold=154.4499969482422
node_14: feature_name=ENSG00000007968	feature_id[80].value <= threshold=20.079999923706055
node_15: feature_name=ENSG00000105355	feature_id[116].value <= threshold=24.335000038146973
node_16: feature_name=ENSG00000178999	feature_id[12].value <= threshold=10.694999694824219

Class: G1	
Rules_31	passed counts:3
node_0: feature_name=ENSG00000170312	feature_id[0].value <= threshold=120.56500244140625
node_1: feature_name=ENSG00000197061	feature_id[22].value <= threshold=794.760009765625
node_2: feature_name=ENSG00000091164	feature_id[113].value <= threshold=29.735000610351562
node_3: feature_name=ENSG00000137310	feature_id[71].value > threshold=4.164999961853027
Class: S	
Rules_32	passed counts:3
node_0: feature_name=ENSG00000170312	feature_id[0].value <= threshold=120.56500244140625
node_1: feature_name=ENSG00000197061	feature_id[22].value <= threshold=794.760009765625
node_2: feature_name=ENSG00000091164	feature_id[113].value <= threshold=29.735000610351562
node_3: feature_name=ENSG00000137310	feature_id[71].value <= threshold=4.164999961853027
node_4: feature_name=ENSG00000196230	feature_id[61].value > threshold=1130.8150024414062
Class: G1	
Rules_33	passed counts:3
node_0: feature_name=ENSG00000170312	feature_id[0].value <= threshold=120.56500244140625
node_1: feature_name=ENSG00000197061	feature_id[22].value <= threshold=794.760009765625
node_2: feature_name=ENSG00000091164	feature_id[113].value <= threshold=29.735000610351562
node_3: feature_name=ENSG00000137310	feature_id[71].value <= threshold=4.164999961853027
node_4: feature_name=ENSG00000196230	feature_id[61].value <= threshold=1130.8150024414062
node_5: feature_name=ENSG00000100097	feature_id[68].value <= threshold=13833.3251953125
Class: G2M	

Rules_34	passed counts:2
node_0: feature_name=ENSG00000170312	feature_id[0].value > threshold=120.56500244140625
node_34: feature_name=ENSG00000140451	feature_id[20].value > threshold=8.110000133514404
node_92: feature_name=ENSG00000105173	feature_id[67].value <= threshold=98.07999801635742
node_93: feature_name=ENSG00000170312	feature_id[0].value > threshold=160.83999633789062
node_99: feature_name=ENSG00000070950	feature_id[108].value <= threshold=74.7750015258789
node_100: feature_name=ENSG00000175130	feature_id[152].value <= threshold=103.5099983215332
node_101: feature_name=ENSG00000117724	feature_id[111].value > threshold=42.60000038146973
node_115: feature_name=ENSG00000197061	feature_id[22].value <= threshold=144.55500030517578
node_116: feature_name=ENSG00000235571	feature_id[119].value > threshold=12.9350004196167
node_122: feature_name=ENSG00000142945	feature_id[86].value <= threshold=156.95000457763672
Class: G2M	
Rules_35	passed counts:2
node_0: feature_name=ENSG00000170312	feature_id[0].value > threshold=120.56500244140625
node_34: feature_name=ENSG00000140451	feature_id[20].value > threshold=8.110000133514404
node_92: feature_name=ENSG00000105173	feature_id[67].value <= threshold=98.07999801635742
node_93: feature_name=ENSG00000170312	feature_id[0].value > threshold=160.83999633789062
node_99: feature_name=ENSG00000070950	feature_id[108].value <= threshold=74.7750015258789
node_100: feature_name=ENSG00000175130	feature_id[152].value <= threshold=103.5099983215332
node_101: feature_name=ENSG00000117724	feature_id[111].value <= threshold=42.60000038146973
node_102: feature_name=ENSG00000128951	feature_id[123].value > threshold=160.25
node_112: feature_name=ENSG00000118193	feature_id[82].value <= threshold=2.0850000381469727
Class: S	

Rules_36	passed counts:2
node_0: feature_name=ENSG00000170312	feature_id[0].value > threshold=120.56500244140625
node_34: feature_name=ENSG00000140451	feature_id[20].value <= threshold=8.110000133514404
node_35: feature_name=ENSG00000100297	feature_id[54].value > threshold=111.95999908447266
node_57: feature_name=ENSG00000197061	feature_id[22].value > threshold=468.4400177001953
node_61: feature_name=ENSG00000012048	feature_id[36].value > threshold=23.5600004196167
node_73: feature_name=ENSG00000229089	feature_id[37].value > threshold=106.125
Class: G2M	
Rules_37	passed counts:2
node_0: feature_name=ENSG00000170312	feature_id[0].value > threshold=120.56500244140625
node_34: feature_name=ENSG00000140451	feature_id[20].value <= threshold=8.110000133514404
node_35: feature_name=ENSG00000100297	feature_id[54].value > threshold=111.95999908447266
node_57: feature_name=ENSG00000197061	feature_id[22].value > threshold=468.4400177001953
node_61: feature_name=ENSG00000012048	feature_id[36].value <= threshold=23.5600004196167
node_62: feature_name=ENSG00000135451	feature_id[43].value > threshold=42.31999969482422
node_66: feature_name=ENSG00000013573	feature_id[92].value <= threshold=54.625
node_67: feature_name=ENSG00000171793	feature_id[58].value > threshold=282.51499938964844
node_69: feature_name=ENSG00000152253	feature_id[140].value <= threshold=84.25500106811523
Class: S	
Rules_38	passed counts:2
node_0: feature_name=ENSG00000170312	feature_id[0].value > threshold=120.56500244140625
node_34: feature_name=ENSG00000140451	feature_id[20].value <= threshold=8.110000133514404

node_35: feature_name=ENSG00000100297	feature_id[54].value > threshold=111.95999908447266
node_57: feature_name=ENSG00000197061	feature_id[22].value > threshold=468.4400177001953
node_61: feature_name=ENSG00000012048	feature_id[36].value <= threshold=23.5600004196167
node_62: feature_name=ENSG00000135451	feature_id[43].value <= threshold=42.31999969482422
node_63: feature_name=ENSG00000148773	feature_id[73].value <= threshold=24.8149995803833
Class: G1	
Rules_39	passed counts:2
node_0: feature_name=ENSG00000170312	feature_id[0].value > threshold=120.56500244140625
node_34: feature_name=ENSG00000140451	feature_id[20].value <= threshold=8.110000133514404
node_35: feature_name=ENSG00000100297	feature_id[54].value > threshold=111.95999908447266
node_57: feature_name=ENSG00000197061	feature_id[22].value <= threshold=468.4400177001953
node_58: feature_name=ENSG00000127922	feature_id[94].value <= threshold=402.9700012207031
Class: S	
Rules_40	passed counts:2
node_0: feature_name=ENSG00000170312	feature_id[0].value > threshold=120.56500244140625
node_34: feature_name=ENSG00000140451	feature_id[20].value <= threshold=8.110000133514404
node_35: feature_name=ENSG00000100297	feature_id[54].value <= threshold=111.95999908447266
node_36: feature_name=ENSG00000102384	feature_id[28].value > threshold=15.289999961853027
node_46: feature_name=ENSG00000169242	feature_id[155].value <= threshold=16.90499973297119
node_47: feature_name=ENSG00000156802	feature_id[18].value > threshold=32.334999084472656
node_51: feature_name=ENSG00000276368	feature_id[46].value > threshold=156.26000213623047
Class: G2M	

Rules_41	passed counts:2
node_0: feature_name=ENSG00000170312	feature_id[0].value > threshold=120.56500244140625
node_34: feature_name=ENSG00000140451	feature_id[20].value <= threshold=8.110000133514404
node_35: feature_name=ENSG00000100297	feature_id[54].value <= threshold=111.95999908447266
node_36: feature_name=ENSG00000102384	feature_id[28].value > threshold=15.289999961853027
node_46: feature_name=ENSG00000169242	feature_id[155].value <= threshold=16.90499973297119
node_47: feature_name=ENSG00000156802	feature_id[18].value <= threshold=32.334999084472656
node_48: feature_name=ENSG00000175573	feature_id[145].value <= threshold=92.84999656677246
Class: G1	
Rules_42	passed counts:2
node_0: feature_name=ENSG00000170312	feature_id[0].value > threshold=120.56500244140625
node_34: feature_name=ENSG00000140451	feature_id[20].value <= threshold=8.110000133514404
node_35: feature_name=ENSG00000100297	feature_id[54].value <= threshold=111.95999908447266
node_36: feature_name=ENSG00000102384	feature_id[28].value <= threshold=15.289999961853027
node_37: feature_name=ENSG00000123473	feature_id[51].value <= threshold=74.30500030517578
node_38: feature_name=ENSG00000102362	feature_id[136].value > threshold=11.420000076293945
Class: S	
Rules_43	passed counts:2
node_0: feature_name=ENSG00000170312	feature_id[0].value <= threshold=120.56500244140625
node_1: feature_name=ENSG00000197061	feature_id[22].value > threshold=794.760009765625
node_29: feature_name=ENSG00000164104	feature_id[50].value <= threshold=51.71500015258789
node_30: feature_name=ENSG00000103995	feature_id[132].value <= threshold=4.700000047683716
Class: S	

Rules_44	passed counts:2
node_0: feature_name=ENSG00000170312	feature_id[0].value <= threshold=120.56500244140625
node_1: feature_name=ENSG00000197061	feature_id[22].value <= threshold=794.760009765625
node_2: feature_name=ENSG00000091164	feature_id[113].value > threshold=29.735000610351562
node_10: feature_name=ENSG00000093009	feature_id[19].value > threshold=198.45000457763672
Class: S	
Rules_45	passed counts:1
node_0: feature_name=ENSG00000170312	feature_id[0].value > threshold=120.56500244140625
node_34: feature_name=ENSG00000140451	feature_id[20].value > threshold=8.110000133514404
node_92: feature_name=ENSG00000105173	feature_id[67].value > threshold=98.07999801635742
node_142: feature_name=ENSG00000173207	feature_id[27].value <= threshold=529.4750061035156
node_143: feature_name=ENSG00000112118	feature_id[11].value <= threshold=74.27000045776367
node_144: feature_name=ENSG00000106211	feature_id[87].value <= threshold=406.1999969482422
Class: S	
Rules_46	passed counts:1
node_0: feature_name=ENSG00000170312	feature_id[0].value > threshold=120.56500244140625
node_34: feature_name=ENSG00000140451	feature_id[20].value > threshold=8.110000133514404
node_92: feature_name=ENSG00000105173	feature_id[67].value <= threshold=98.07999801635742
node_93: feature_name=ENSG00000170312	feature_id[0].value > threshold=160.83999633789062
node_99: feature_name=ENSG00000070950	feature_id[108].value <= threshold=74.7750015258789
node_100: feature_name=ENSG00000175130	feature_id[152].value > threshold=103.5099983215332
node_138: feature_name=ENSG00000101447	feature_id[32].value <= threshold=154.69000244140625

Class: G2M	
Rules_47	passed counts:1
node_0: feature_name=ENSG00000170312	feature_id[0].value > threshold=120.56500244140625
node_34: feature_name=ENSG00000140451	feature_id[20].value > threshold=8.110000133514404
node_92: feature_name=ENSG00000105173	feature_id[67].value <= threshold=98.07999801635742
node_93: feature_name=ENSG00000170312	feature_id[0].value > threshold=160.83999633789062
node_99: feature_name=ENSG00000070950	feature_id[108].value <= threshold=74.7750015258789
node_100: feature_name=ENSG00000175130	feature_id[152].value <= threshold=103.5099983215332
node_101: feature_name=ENSG00000117724	feature_id[111].value > threshold=42.60000038146973
node_115: feature_name=ENSG00000197061	feature_id[22].value > threshold=144.55500030517578
node_125: feature_name=ENSG00000122952	feature_id[57].value > threshold=716.2649841308594
Class: S	
Rules_48	passed counts:1
node_0: feature_name=ENSG00000170312	feature_id[0].value > threshold=120.56500244140625
node_34: feature_name=ENSG00000140451	feature_id[20].value > threshold=8.110000133514404
node_92: feature_name=ENSG00000105173	feature_id[67].value <= threshold=98.07999801635742
node_93: feature_name=ENSG00000170312	feature_id[0].value > threshold=160.83999633789062
node_99: feature_name=ENSG00000070950	feature_id[108].value <= threshold=74.7750015258789
node_100: feature_name=ENSG00000175130	feature_id[152].value <= threshold=103.5099983215332
node_101: feature_name=ENSG00000117724	feature_id[111].value > threshold=42.60000038146973
node_115: feature_name=ENSG00000197061	feature_id[22].value > threshold=144.55500030517578
node_125: feature_name=ENSG00000122952	feature_id[57].value <= threshold=716.2649841308594
node_126: feature_name=ENSG00000198033	feature_id[149].value > threshold=456.3699951171875

Class: S	
Rules_49	passed counts:1
node_0: feature_name=ENSG00000170312	feature_id[0].value > threshold=120.56500244140625
node_34: feature_name=ENSG00000140451	feature_id[20].value > threshold=8.110000133514404
node_92: feature_name=ENSG00000105173	feature_id[67].value <= threshold=98.07999801635742
node_93: feature_name=ENSG00000170312	feature_id[0].value > threshold=160.83999633789062
node_99: feature_name=ENSG00000070950	feature_id[108].value <= threshold=74.7750015258789
node_100: feature_name=ENSG00000175130	feature_id[152].value <= threshold=103.5099983215332
node_101: feature_name=ENSG00000117724	feature_id[111].value > threshold=42.60000038146973
node_115: feature_name=ENSG00000197061	feature_id[22].value > threshold=144.55500030517578
node_125: feature_name=ENSG00000122952	feature_id[57].value <= threshold=716.2649841308594
node_126: feature_name=ENSG00000198033	feature_id[149].value <= threshold=456.3699951171875
node_127: feature_name=ENSG00000178074	feature_id[96].value > threshold=38.650001525878906
Class: S	
Rules_50	passed counts:1
node_0: feature_name=ENSG00000170312	feature_id[0].value > threshold=120.56500244140625
node_34: feature_name=ENSG00000140451	feature_id[20].value > threshold=8.110000133514404
node_92: feature_name=ENSG00000105173	feature_id[67].value <= threshold=98.07999801635742
node_93: feature_name=ENSG00000170312	feature_id[0].value > threshold=160.83999633789062
node_99: feature_name=ENSG00000070950	feature_id[108].value <= threshold=74.7750015258789
node_100: feature_name=ENSG00000175130	feature_id[152].value <= threshold=103.5099983215332
node_101: feature_name=ENSG00000117724	feature_id[111].value > threshold=42.60000038146973
node_115: feature_name=ENSG00000197061	feature_id[22].value > threshold=144.55500030517578

node_125: feature_name=ENSG00000122952	feature_id[57].value <= threshold=716.2649841308594
node_126: feature_name=ENSG00000198033	feature_id[149].value <= threshold=456.3699951171875
node_127: feature_name=ENSG00000178074	feature_id[96].value <= threshold=38.650001525878906
node_128: feature_name=ENSG00000006327	feature_id[95].value > threshold=560.8050231933594
Class: S	
Rules_51	passed counts:1
node_0: feature_name=ENSG00000170312	feature_id[0].value > threshold=120.56500244140625
node_34: feature_name=ENSG00000140451	feature_id[20].value > threshold=8.110000133514404
node_92: feature_name=ENSG00000105173	feature_id[67].value <= threshold=98.07999801635742
node_93: feature_name=ENSG00000170312	feature_id[0].value > threshold=160.83999633789062
node_99: feature_name=ENSG00000070950	feature_id[108].value <= threshold=74.7750015258789
node_100: feature_name=ENSG00000175130	feature_id[152].value <= threshold=103.5099983215332
node_101: feature_name=ENSG00000117724	feature_id[111].value > threshold=42.60000038146973
node_115: feature_name=ENSG00000197061	feature_id[22].value > threshold=144.55500030517578
node_125: feature_name=ENSG00000122952	feature_id[57].value <= threshold=716.2649841308594
node_126: feature_name=ENSG00000198033	feature_id[149].value <= threshold=456.3699951171875
node_127: feature_name=ENSG00000178074	feature_id[96].value <= threshold=38.650001525878906
node_128: feature_name=ENSG00000006327	feature_id[95].value <= threshold=560.8050231933594
node_129: feature_name=ENSG00000175305	feature_id[49].value > threshold=23.119999885559082
node_131: feature_name=ENSG00000115163	feature_id[117].value > threshold=62.974998474121094
Class: G2M	
Rules_52	passed counts:1
node_0: feature_name=ENSG00000170312	feature_id[0].value > threshold=120.56500244140625

node_34: feature_name=ENSG00000140451	feature_id[20].value > threshold=8.110000133514404
node_92: feature_name=ENSG00000105173	feature_id[67].value <= threshold=98.07999801635742
node_93: feature_name=ENSG00000170312	feature_id[0].value > threshold=160.83999633789062
node_99: feature_name=ENSG00000070950	feature_id[108].value <= threshold=74.7750015258789
node_100: feature_name=ENSG00000175130	feature_id[152].value <= threshold=103.5099983215332
node_101: feature_name=ENSG00000117724	feature_id[111].value > threshold=42.60000038146973
node_115: feature_name=ENSG00000197061	feature_id[22].value > threshold=144.55500030517578
node_125: feature_name=ENSG00000122952	feature_id[57].value <= threshold=716.2649841308594
node_126: feature_name=ENSG00000198033	feature_id[149].value <= threshold=456.3699951171875
node_127: feature_name=ENSG00000178074	feature_id[96].value <= threshold=38.650001525878906
node_128: feature_name=ENSG00000006327	feature_id[95].value <= threshold=560.8050231933594
node_129: feature_name=ENSG00000175305	feature_id[49].value > threshold=23.119999885559082
node_131: feature_name=ENSG00000115163	feature_id[117].value <= threshold=62.974998474121094
Class: S	
Rules_53	passed counts:1
node_0: feature_name=ENSG00000170312	feature_id[0].value > threshold=120.56500244140625
node_34: feature_name=ENSG00000140451	feature_id[20].value > threshold=8.110000133514404
node_92: feature_name=ENSG00000105173	feature_id[67].value <= threshold=98.07999801635742
node_93: feature_name=ENSG00000170312	feature_id[0].value > threshold=160.83999633789062
node_99: feature_name=ENSG00000070950	feature_id[108].value <= threshold=74.7750015258789
node_100: feature_name=ENSG00000175130	feature_id[152].value <= threshold=103.5099983215332
node_101: feature_name=ENSG00000117724	feature_id[111].value > threshold=42.60000038146973
node_115: feature_name=ENSG00000197061	feature_id[22].value <= threshold=144.55500030517578
node_116: feature_name=ENSG00000235571	feature_id[119].value <= threshold=12.9350004196167

node_117: feature_name=ENSG00000112118	feature_id[11].value <= threshold=30.804999351501465
node_118: feature_name=ENSG00000229089	feature_id[37].value > threshold=6.789999961853027
Class: S	
Rules_54	passed counts:1
node_0: feature_name=ENSG00000170312	feature_id[0].value > threshold=120.56500244140625
node_34: feature_name=ENSG00000140451	feature_id[20].value > threshold=8.110000133514404
node_92: feature_name=ENSG00000105173	feature_id[67].value <= threshold=98.07999801635742
node_93: feature_name=ENSG00000170312	feature_id[0].value > threshold=160.83999633789062
node_99: feature_name=ENSG00000070950	feature_id[108].value <= threshold=74.7750015258789
node_100: feature_name=ENSG00000175130	feature_id[152].value <= threshold=103.5099983215332
node_101: feature_name=ENSG00000117724	feature_id[111].value > threshold=42.60000038146973
node_115: feature_name=ENSG00000197061	feature_id[22].value <= threshold=144.55500030517578
node_116: feature_name=ENSG00000235571	feature_id[119].value <= threshold=12.9350004196167
node_117: feature_name=ENSG00000112118	feature_id[11].value <= threshold=30.804999351501465
node_118: feature_name=ENSG00000229089	feature_id[37].value <= threshold=6.789999961853027
Class: G1	
Rules_55	passed counts:1
node_0: feature_name=ENSG00000170312	feature_id[0].value > threshold=120.56500244140625
node_34: feature_name=ENSG00000140451	feature_id[20].value > threshold=8.110000133514404
node_92: feature_name=ENSG00000105173	feature_id[67].value <= threshold=98.07999801635742
node_93: feature_name=ENSG00000170312	feature_id[0].value > threshold=160.83999633789062
node_99: feature_name=ENSG00000070950	feature_id[108].value <= threshold=74.7750015258789
node_100: feature_name=ENSG00000175130	feature_id[152].value <= threshold=103.5099983215332

node_101: feature_name=ENSG00000117724	feature_id[111].value <= threshold=42.60000038146973
node_102: feature_name=ENSG00000128951	feature_id[123].value <= threshold=160.25
node_103: feature_name=ENSG00000105011	feature_id[31].value > threshold=61.09000015258789
node_109: feature_name=ENSG00000136982	feature_id[112].value <= threshold=17.335000038146973
Class: G2M	
Rules_56	passed counts:1
node_0: feature_name=ENSG00000170312	feature_id[0].value > threshold=120.56500244140625
node_34: feature_name=ENSG00000140451	feature_id[20].value > threshold=8.110000133514404
node_92: feature_name=ENSG00000105173	feature_id[67].value <= threshold=98.07999801635742
node_93: feature_name=ENSG00000170312	feature_id[0].value > threshold=160.83999633789062
node_99: feature_name=ENSG00000070950	feature_id[108].value <= threshold=74.7750015258789
node_100: feature_name=ENSG00000175130	feature_id[152].value <= threshold=103.5099983215332
node_101: feature_name=ENSG00000117724	feature_id[111].value <= threshold=42.60000038146973
node_102: feature_name=ENSG00000128951	feature_id[123].value <= threshold=160.25
node_103: feature_name=ENSG00000105011	feature_id[31].value <= threshold=61.09000015258789
node_104: feature_name=ENSG00000163808	feature_id[89].value <= threshold=9.610000133514404
node_105: feature_name=ENSG00000123975	feature_id[90].value > threshold=1455.4949951171875
Class: S	
Rules_57	passed counts:1
node_0: feature_name=ENSG00000170312	feature_id[0].value > threshold=120.56500244140625
node_34: feature_name=ENSG00000140451	feature_id[20].value > threshold=8.110000133514404
node_92: feature_name=ENSG00000105173	feature_id[67].value <= threshold=98.07999801635742
node_93: feature_name=ENSG00000170312	feature_id[0].value <= threshold=160.83999633789062

node_94: feature_name=ENSG00000182481	feature_id[35].value <= threshold=678.3799743652344
node_95: feature_name=ENSG00000103995	feature_id[132].value <= threshold=9.0
Class: G2M	
Rules_58	passed counts:1
node_0: feature_name=ENSG00000170312	feature_id[0].value > threshold=120.56500244140625
node_34: feature_name=ENSG00000140451	feature_id[20].value <= threshold=8.110000133514404
node_35: feature_name=ENSG00000100297	feature_id[54].value > threshold=111.95999908447266
node_57: feature_name=ENSG00000197061	feature_id[22].value > threshold=468.4400177001953
node_61: feature_name=ENSG00000012048	feature_id[36].value > threshold=23.5600004196167
node_73: feature_name=ENSG00000229089	feature_id[37].value <= threshold=106.125
node_74: feature_name=ENSG00000182481	feature_id[35].value > threshold=505.8800048828125
node_84: feature_name=ENSG00000146278	feature_id[64].value <= threshold=0.7649999856948853
node_85: feature_name=ENSG00000025770	feature_id[131].value <= threshold=91.17000198364258
node_86: feature_name=ENSG00000229089	feature_id[37].value <= threshold=18.195000171661377
Class: G1	
Rules_59	passed counts:1
node_0: feature_name=ENSG00000170312	feature_id[0].value > threshold=120.56500244140625
node_34: feature_name=ENSG00000140451	feature_id[20].value <= threshold=8.110000133514404
node_35: feature_name=ENSG00000100297	feature_id[54].value > threshold=111.95999908447266
node_57: feature_name=ENSG00000197061	feature_id[22].value > threshold=468.4400177001953
node_61: feature_name=ENSG00000012048	feature_id[36].value > threshold=23.5600004196167
node_73: feature_name=ENSG00000229089	feature_id[37].value <= threshold=106.125
node_74: feature_name=ENSG00000182481	feature_id[35].value <= threshold=505.8800048828125

node_75: feature_name=ENSG00000088325	feature_id[143].value > threshold=14.054999828338623
node_77: feature_name=ENSG00000088986	feature_id[83].value > threshold=1100.9449768066406
Class: G2M	
Rules_60	passed counts:1
node_0: feature_name=ENSG00000170312	feature_id[0].value > threshold=120.56500244140625
node_34: feature_name=ENSG00000140451	feature_id[20].value <= threshold=8.110000133514404
node_35: feature_name=ENSG00000100297	feature_id[54].value > threshold=111.95999908447266
node_57: feature_name=ENSG00000197061	feature_id[22].value > threshold=468.4400177001953
node_61: feature_name=ENSG00000012048	feature_id[36].value > threshold=23.5600004196167
node_73: feature_name=ENSG00000229089	feature_id[37].value <= threshold=106.125
node_74: feature_name=ENSG00000182481	feature_id[35].value <= threshold=505.8800048828125
node_75: feature_name=ENSG00000088325	feature_id[143].value > threshold=14.054999828338623
node_77: feature_name=ENSG00000088986	feature_id[83].value <= threshold=1100.9449768066406
node_78: feature_name=ENSG00000154473	feature_id[156].value <= threshold=64.73999786376953
node_79: feature_name=ENSG00000198763	feature_id[127].value > threshold=3568.635009765625
Class: G2M	
Rules_61	passed counts:1
node_0: feature_name=ENSG00000170312	feature_id[0].value > threshold=120.56500244140625
node_34: feature_name=ENSG00000140451	feature_id[20].value <= threshold=8.110000133514404
node_35: feature_name=ENSG00000100297	feature_id[54].value > threshold=111.95999908447266
node_57: feature_name=ENSG00000197061	feature_id[22].value > threshold=468.4400177001953
node_61: feature_name=ENSG00000012048	feature_id[36].value > threshold=23.5600004196167
node_73: feature_name=ENSG00000229089	feature_id[37].value <= threshold=106.125

node_74: feature_name=ENSG00000182481	feature_id[35].value <= threshold=505.8800048828125
node_75: feature_name=ENSG00000088325	feature_id[143].value > threshold=14.054999828338623
node_77: feature_name=ENSG00000088986	feature_id[83].value <= threshold=1100.9449768066406
node_78: feature_name=ENSG00000154473	feature_id[156].value <= threshold=64.73999786376953
node_79: feature_name=ENSG00000198763	feature_id[127].value <= threshold=3568.635009765625
Class: S	
Rules_62	passed counts:1
node_0: feature_name=ENSG00000170312	feature_id[0].value > threshold=120.56500244140625
node_34: feature_name=ENSG00000140451	feature_id[20].value <= threshold=8.110000133514404
node_35: feature_name=ENSG00000100297	feature_id[54].value > threshold=111.95999908447266
node_57: feature_name=ENSG00000197061	feature_id[22].value > threshold=468.4400177001953
node_61: feature_name=ENSG00000012048	feature_id[36].value > threshold=23.5600004196167
node_73: feature_name=ENSG00000229089	feature_id[37].value <= threshold=106.125
node_74: feature_name=ENSG00000182481	feature_id[35].value <= threshold=505.8800048828125
node_75: feature_name=ENSG00000088325	feature_id[143].value <= threshold=14.054999828338623
Class: G1	
Rules_63	passed counts:1
node_0: feature_name=ENSG00000170312	feature_id[0].value > threshold=120.56500244140625
node_34: feature_name=ENSG00000140451	feature_id[20].value <= threshold=8.110000133514404
node_35: feature_name=ENSG00000100297	feature_id[54].value > threshold=111.95999908447266
node_57: feature_name=ENSG00000197061	feature_id[22].value > threshold=468.4400177001953
node_61: feature_name=ENSG00000012048	feature_id[36].value <= threshold=23.5600004196167
node_62: feature_name=ENSG00000135451	feature_id[43].value > threshold=42.31999969482422

node_66: feature_name=ENSG00000013573	feature_id[92].value <= threshold=54.625
node_67: feature_name=ENSG00000171793	feature_id[58].value > threshold=282.51499938964844
node_69: feature_name=ENSG00000152253	feature_id[140].value > threshold=84.25500106811523
Class: G2M	
Rules_64	passed counts:1
node_0: feature_name=ENSG00000170312	feature_id[0].value > threshold=120.56500244140625
node_34: feature_name=ENSG00000140451	feature_id[20].value <= threshold=8.110000133514404
node_35: feature_name=ENSG00000100297	feature_id[54].value <= threshold=111.95999908447266
node_36: feature_name=ENSG00000102384	feature_id[28].value > threshold=15.289999961853027
node_46: feature_name=ENSG00000169242	feature_id[155].value <= threshold=16.90499973297119
node_47: feature_name=ENSG00000156802	feature_id[18].value > threshold=32.334999084472656
node_51: feature_name=ENSG00000276368	feature_id[46].value <= threshold=156.26000213623047
node_52: feature_name=ENSG00000175063	feature_id[9].value <= threshold=250.1449966430664
Class: G2M	
Rules_65	passed counts:1
node_0: feature_name=ENSG00000170312	feature_id[0].value > threshold=120.56500244140625
node_34: feature_name=ENSG00000140451	feature_id[20].value <= threshold=8.110000133514404
node_35: feature_name=ENSG00000100297	feature_id[54].value <= threshold=111.95999908447266
node_36: feature_name=ENSG00000102384	feature_id[28].value > threshold=15.289999961853027
node_46: feature_name=ENSG00000169242	feature_id[155].value <= threshold=16.90499973297119
node_47: feature_name=ENSG00000156802	feature_id[18].value <= threshold=32.334999084472656
node_48: feature_name=ENSG00000175573	feature_id[145].value > threshold=92.84999656677246
Class: G2M	

Rules_66	passed counts:1
node_0: feature_name=ENSG00000170312	feature_id[0].value > threshold=120.56500244140625
node_34: feature_name=ENSG00000140451	feature_id[20].value <= threshold=8.110000133514404
node_35: feature_name=ENSG00000100297	feature_id[54].value <= threshold=111.95999908447266
node_36: feature_name=ENSG00000102384	feature_id[28].value <= threshold=15.289999961853027
node_37: feature_name=ENSG00000123473	feature_id[51].value <= threshold=74.30500030517578
node_38: feature_name=ENSG00000102362	feature_id[136].value <= threshold=11.420000076293945
node_39: feature_name=ENSG00000205352	feature_id[100].value > threshold=243.77000427246094
Class: S	
Rules_67	passed counts:1
node_0: feature_name=ENSG00000170312	feature_id[0].value > threshold=120.56500244140625
node_34: feature_name=ENSG00000140451	feature_id[20].value <= threshold=8.110000133514404
node_35: feature_name=ENSG00000100297	feature_id[54].value <= threshold=111.95999908447266
node_36: feature_name=ENSG00000102384	feature_id[28].value <= threshold=15.289999961853027
node_37: feature_name=ENSG00000123473	feature_id[51].value <= threshold=74.30500030517578
node_38: feature_name=ENSG00000102362	feature_id[136].value <= threshold=11.420000076293945
node_39: feature_name=ENSG00000205352	feature_id[100].value <= threshold=243.77000427246094
node_40: feature_name=ENSG00000031691	feature_id[76].value > threshold=47.04499912261963
Class: S	
Rules_68	passed counts:1
node_0: feature_name=ENSG00000170312	feature_id[0].value <= threshold=120.56500244140625
node_1: feature_name=ENSG00000197061	feature_id[22].value <= threshold=794.760009765625

node_2: feature_name=ENSG00000091164	feature_id[113].value > threshold=29.735000610351562
node_10: feature_name=ENSG00000093009	feature_id[19].value <= threshold=198.45000457763672
node_11: feature_name=ENSG00000167553	feature_id[135].value > threshold=88.50500106811523
node_13: feature_name=ENSG00000025770	feature_id[131].value > threshold=154.4499969482422
node_23: feature_name=ENSG00000101104	feature_id[40].value > threshold=13.899999618530273
node_25: feature_name=ENSG00000121716	feature_id[47].value > threshold=54.21500015258789
Class: G2M	
Rules_69	passed counts:1
node_0: feature_name=ENSG00000170312	feature_id[0].value <= threshold=120.56500244140625
node_1: feature_name=ENSG00000197061	feature_id[22].value <= threshold=794.760009765625
node_2: feature_name=ENSG00000091164	feature_id[113].value > threshold=29.735000610351562
node_10: feature_name=ENSG00000093009	feature_id[19].value <= threshold=198.45000457763672
node_11: feature_name=ENSG00000167553	feature_id[135].value > threshold=88.50500106811523
node_13: feature_name=ENSG00000025770	feature_id[131].value > threshold=154.4499969482422
node_23: feature_name=ENSG00000101104	feature_id[40].value > threshold=13.899999618530273
node_25: feature_name=ENSG00000121716	feature_id[47].value <= threshold=54.21500015258789
Class: S	
Rules_70	passed counts:1
node_0: feature_name=ENSG00000170312	feature_id[0].value <= threshold=120.56500244140625
node_1: feature_name=ENSG00000197061	feature_id[22].value <= threshold=794.760009765625
node_2: feature_name=ENSG00000091164	feature_id[113].value > threshold=29.735000610351562
node_10: feature_name=ENSG00000093009	feature_id[19].value <= threshold=198.45000457763672
node_11: feature_name=ENSG00000167553	feature_id[135].value > threshold=88.50500106811523

node_13: feature_name=ENSG00000025770	feature_id[131].value > threshold=154.4499969482422
node_23: feature_name=ENSG00000101104	feature_id[40].value <= threshold=13.899999618530273
Class: G1	
Rules_71	passed counts:1
node_0: feature_name=ENSG00000170312	feature_id[0].value <= threshold=120.56500244140625
node_1: feature_name=ENSG00000197061	feature_id[22].value <= threshold=794.760009765625
node_2: feature_name=ENSG00000091164	feature_id[113].value > threshold=29.735000610351562
node_10: feature_name=ENSG00000093009	feature_id[19].value <= threshold=198.45000457763672
node_11: feature_name=ENSG00000167553	feature_id[135].value > threshold=88.50500106811523
node_13: feature_name=ENSG00000025770	feature_id[131].value <= threshold=154.4499969482422
node_14: feature_name=ENSG00000007968	feature_id[80].value > threshold=20.079999923706055
node_20: feature_name=ENSG00000198033	feature_id[149].value > threshold=300.86499786376953
Class: G1	
Rules_72	passed counts:1
node_0: feature_name=ENSG00000170312	feature_id[0].value <= threshold=120.56500244140625
node_1: feature_name=ENSG00000197061	feature_id[22].value <= threshold=794.760009765625
node_2: feature_name=ENSG00000091164	feature_id[113].value > threshold=29.735000610351562
node_10: feature_name=ENSG00000093009	feature_id[19].value <= threshold=198.45000457763672
node_11: feature_name=ENSG00000167553	feature_id[135].value > threshold=88.50500106811523
node_13: feature_name=ENSG00000025770	feature_id[131].value <= threshold=154.4499969482422
node_14: feature_name=ENSG00000007968	feature_id[80].value > threshold=20.079999923706055
node_20: feature_name=ENSG00000198033	feature_id[149].value <= threshold=300.86499786376953
Class: S	

Rules_73	passed counts:1
node_0: feature_name=ENSG00000170312	feature_id[0].value <= threshold=120.56500244140625
node_1: feature_name=ENSG00000197061	feature_id[22].value <= threshold=794.760009765625
node_2: feature_name=ENSG00000091164	feature_id[113].value > threshold=29.735000610351562
node_10: feature_name=ENSG00000093009	feature_id[19].value <= threshold=198.45000457763672
node_11: feature_name=ENSG00000167553	feature_id[135].value > threshold=88.50500106811523
node_13: feature_name=ENSG00000025770	feature_id[131].value <= threshold=154.4499969482422
node_14: feature_name=ENSG00000007968	feature_id[80].value <= threshold=20.079999923706055
node_15: feature_name=ENSG00000105355	feature_id[116].value <= threshold=24.335000038146973
node_16: feature_name=ENSG00000178999	feature_id[12].value > threshold=10.694999694824219
Class: S	
Rules_74	passed counts:1
node_0: feature_name=ENSG00000170312	feature_id[0].value <= threshold=120.56500244140625
node_1: feature_name=ENSG00000197061	feature_id[22].value <= threshold=794.760009765625
node_2: feature_name=ENSG00000091164	feature_id[113].value > threshold=29.735000610351562
node_10: feature_name=ENSG00000093009	feature_id[19].value <= threshold=198.45000457763672
node_11: feature_name=ENSG00000167553	feature_id[135].value <= threshold=88.50500106811523
Class: S	
Rules_75	passed counts:1
node_0: feature_name=ENSG00000170312	feature_id[0].value <= threshold=120.56500244140625
node_1: feature_name=ENSG00000197061	feature_id[22].value <= threshold=794.760009765625
node_2: feature_name=ENSG00000091164	feature_id[113].value <= threshold=29.735000610351562

node_3: feature_name=ENSG00000137310	feature_id[71].value <= threshold=4.164999961853027
node_4: feature_name=ENSG00000196230	feature_id[61].value <= threshold=1130.8150024414062
node_5: feature_name=ENSG00000100097	feature_id[68].value > threshold=13833.3251953125
Class: G1	

(2) Rules on features selected by MCFS

Rules_0	passed counts:317
node_0: feature_name=ENSG00000170312	feature_id[0].value <= threshold=120.56500244140625
node_1: feature_name=ENSG00000197061	feature_id[2].value <= threshold=794.760009765625
node_2: feature_name=ENSG00000091164	feature_id[188].value > threshold=29.735000610351562
node_10: feature_name=ENSG00000093009	feature_id[59].value <= threshold=198.45000457763672
node_11: feature_name=ENSG00000154640	feature_id[353].value <= threshold=326.40999603271484
node_12: feature_name=ENSG00000106462	feature_id[284].value <= threshold=37.8650016784668
node_13: feature_name=ENSG00000138376	feature_id[137].value <= threshold=85.30999755859375
node_14: feature_name=ENSG00000173207	feature_id[26].value <= threshold=800.375
node_15: feature_name=ENSG00000232388	feature_id[360].value <= threshold=253.9800033569336
Class: G1	
Rules_1	passed counts:239
node_0: feature_name=ENSG00000170312	feature_id[0].value > threshold=120.56500244140625
node_32: feature_name=ENSG00000140451	feature_id[10].value > threshold=8.110000133514404
node_80: feature_name=ENSG00000105173	feature_id[48].value <= threshold=98.07999801635742
node_81: feature_name=ENSG00000170312	feature_id[0].value > threshold=160.83999633789062
node_87: feature_name=ENSG00000167978	feature_id[187].value > threshold=37.79999923706055
node_93: feature_name=ENSG00000137135	feature_id[199].value > threshold=1.699999988079071

node_97: feature_name=ENSG00000130429	feature_id[361].value <= threshold=323.2150115966797
node_98: feature_name=ENSG00000116717	feature_id[135].value > threshold=13.164999961853027
node_100: feature_name=ENSG00000117724	feature_id[94].value > threshold=42.60000038146973
node_114: feature_name=ENSG00000145604	feature_id[390].value <= threshold=104.21000289916992
node_115: feature_name=ENSG00000183741	feature_id[331].value <= threshold=45.329999923706055
node_116: feature_name=ENSG00000122565	feature_id[346].value <= threshold=237.02999877929688
Class: G2M	
Rules_2	passed counts:205
node_0: feature_name=ENSG00000170312	feature_id[0].value > threshold=120.56500244140625
node_32: feature_name=ENSG00000140451	feature_id[10].value <= threshold=8.110000133514404
node_33: feature_name=ENSG00000100297	feature_id[51].value > threshold=111.95999908447266
node_51: feature_name=ENSG00000197061	feature_id[2].value > threshold=468.4400177001953
node_55: feature_name=ENSG00000012048	feature_id[99].value > threshold=23.5600004196167
node_63: feature_name=ENSG00000127564	feature_id[107].value > threshold=22.22499942779541
node_67: feature_name=ENSG00000167601	feature_id[242].value <= threshold=391.2899932861328
node_68: feature_name=ENSG00000259848	feature_id[68].value <= threshold=239.00499725341797
node_69: feature_name=ENSG00000117650	feature_id[84].value <= threshold=76.625
node_70: feature_name=ENSG00000185361	feature_id[58].value <= threshold=12.335000038146973
node_71: feature_name=ENSG00000121152	feature_id[33].value <= threshold=224.78500366210938
node_72: feature_name=ENSG00000151503	feature_id[451].value > threshold=0.26499998569488525
Class: S	
Rules_3	passed counts:45
node_0: feature_name=ENSG00000170312	feature_id[0].value > threshold=120.56500244140625

node_32: feature_name=ENSG00000140451	feature_id[10].value > threshold=8.110000133514404
node_80: feature_name=ENSG00000105173	feature_id[48].value <= threshold=98.07999801635742
node_81: feature_name=ENSG00000170312	feature_id[0].value > threshold=160.83999633789062
node_87: feature_name=ENSG00000167978	feature_id[187].value > threshold=37.79999923706055
node_93: feature_name=ENSG00000137135	feature_id[199].value > threshold=1.699999988079071
node_97: feature_name=ENSG00000130429	feature_id[361].value <= threshold=323.2150115966797
node_98: feature_name=ENSG00000116717	feature_id[135].value > threshold=13.164999961853027
node_100: feature_name=ENSG00000117724	feature_id[94].value <= threshold=42.60000038146973
node_101: feature_name=ENSG00000101003	feature_id[339].value <= threshold=18.390000343322754
node_102: feature_name=ENSG00000278259	feature_id[436].value <= threshold=66.98500061035156
node_103: feature_name=ENSG00000237649	feature_id[104].value > threshold=2.6549999713897705
node_107: feature_name=ENSG00000183726	feature_id[461].value > threshold=36.849998474121094
Class: G2M	
Rules_4	passed counts:37
node_0: feature_name=ENSG00000170312	feature_id[0].value > threshold=120.56500244140625
node_32: feature_name=ENSG00000140451	feature_id[10].value <= threshold=8.110000133514404
node_33: feature_name=ENSG00000100297	feature_id[51].value <= threshold=111.95999908447266
node_34: feature_name=ENSG00000150991	feature_id[194].value <= threshold=998.7099914550781
node_35: feature_name=ENSG00000175305	feature_id[38].value <= threshold=14.080000400543213
node_36: feature_name=ENSG00000127483	feature_id[191].value <= threshold=215.8800048828125
node_37: feature_name=ENSG00000171793	feature_id[183].value <= threshold=335.6300048828125
node_38: feature_name=ENSG00000130429	feature_id[361].value <= threshold=291.3249969482422
Class: G2M	

Rules_5	passed counts:20
node_0: feature_name=ENSG00000170312	feature_id[0].value > threshold=120.56500244140625
node_32: feature_name=ENSG00000140451	feature_id[10].value > threshold=8.110000133514404
node_80: feature_name=ENSG00000105173	feature_id[48].value > threshold=98.07999801635742
node_128: feature_name=ENSG00000173207	feature_id[26].value <= threshold=529.4750061035156
node_129: feature_name=ENSG00000112118	feature_id[49].value > threshold=74.27000045776367
Class: S	
Rules_6	passed counts:17
node_0: feature_name=ENSG00000170312	feature_id[0].value > threshold=120.56500244140625
node_32: feature_name=ENSG00000140451	feature_id[10].value <= threshold=8.110000133514404
node_33: feature_name=ENSG00000100297	feature_id[51].value <= threshold=111.95999908447266
node_34: feature_name=ENSG00000150991	feature_id[194].value > threshold=998.7099914550781
node_48: feature_name=ENSG00000205352	feature_id[211].value > threshold=73.43000030517578
Class: S	
Rules_7	passed counts:15
node_0: feature_name=ENSG00000170312	feature_id[0].value > threshold=120.56500244140625
node_32: feature_name=ENSG00000140451	feature_id[10].value > threshold=8.110000133514404
node_80: feature_name=ENSG00000105173	feature_id[48].value > threshold=98.07999801635742
node_128: feature_name=ENSG00000173207	feature_id[26].value > threshold=529.4750061035156
node_134: feature_name=ENSG00000106367	feature_id[195].value > threshold=167.65499877929688
Class: G2M	
Rules_8	passed counts:13

node_0: feature_name=ENSG00000170312	feature_id[0].value <= threshold=120.56500244140625
node_1: feature_name=ENSG00000197061	feature_id[2].value > threshold=794.760009765625
node_27: feature_name=ENSG00000168476	feature_id[217].value <= threshold=39.849998474121094
node_28: feature_name=ENSG00000034510	feature_id[424].value <= threshold=9527.485107421875
Class: S	
Rules_9	passed counts:12
node_0: feature_name=ENSG00000170312	feature_id[0].value > threshold=120.56500244140625
node_32: feature_name=ENSG00000140451	feature_id[10].value <= threshold=8.110000133514404
node_33: feature_name=ENSG00000100297	feature_id[51].value > threshold=111.95999908447266
node_51: feature_name=ENSG00000197061	feature_id[2].value > threshold=468.4400177001953
node_55: feature_name=ENSG00000012048	feature_id[99].value <= threshold=23.5600004196167
node_56: feature_name=ENSG00000135451	feature_id[93].value > threshold=42.31999969482422
node_60: feature_name=ENSG00000130429	feature_id[361].value <= threshold=215.33999633789062
Class: G2M	
Rules_10	passed counts:9
node_0: feature_name=ENSG00000170312	feature_id[0].value > threshold=120.56500244140625
node_32: feature_name=ENSG00000140451	feature_id[10].value <= threshold=8.110000133514404
node_33: feature_name=ENSG00000100297	feature_id[51].value <= threshold=111.95999908447266
node_34: feature_name=ENSG00000150991	feature_id[194].value <= threshold=998.7099914550781
node_35: feature_name=ENSG00000175305	feature_id[38].value > threshold=14.080000400543213
node_45: feature_name=ENSG00000198554	feature_id[457].value <= threshold=40.564998626708984
Class: S	

Rules_11	passed counts:8
node_0: feature_name=ENSG00000170312	feature_id[0].value > threshold=120.56500244140625
node_32: feature_name=ENSG00000140451	feature_id[10].value > threshold=8.110000133514404
node_80: feature_name=ENSG00000105173	feature_id[48].value <= threshold=98.07999801635742
node_81: feature_name=ENSG00000170312	feature_id[0].value > threshold=160.83999633789062
node_87: feature_name=ENSG00000167978	feature_id[187].value > threshold=37.79999923706055
node_93: feature_name=ENSG00000137135	feature_id[199].value > threshold=1.699999988079071
node_97: feature_name=ENSG00000130429	feature_id[361].value <= threshold=323.2150115966797
node_98: feature_name=ENSG00000116717	feature_id[135].value > threshold=13.164999961853027
node_100: feature_name=ENSG00000117724	feature_id[94].value <= threshold=42.60000038146973
node_101: feature_name=ENSG00000101003	feature_id[339].value > threshold=18.390000343322754
node_111: feature_name=ENSG00000198331	feature_id[61].value > threshold=33.454999923706055
Class: S	
Rules_12	passed counts:8
node_0: feature_name=ENSG00000170312	feature_id[0].value > threshold=120.56500244140625
node_32: feature_name=ENSG00000140451	feature_id[10].value <= threshold=8.110000133514404
node_33: feature_name=ENSG00000100297	feature_id[51].value > threshold=111.95999908447266
node_51: feature_name=ENSG00000197061	feature_id[2].value > threshold=468.4400177001953
node_55: feature_name=ENSG00000012048	feature_id[99].value <= threshold=23.5600004196167
node_56: feature_name=ENSG00000135451	feature_id[93].value <= threshold=42.31999969482422
node_57: feature_name=ENSG00000165502	feature_id[258].value <= threshold=488.7150115966797
Class: S	
Rules_13	passed counts:7

node_0: feature_name=ENSG00000170312	feature_id[0].value > threshold=120.56500244140625
node_32: feature_name=ENSG00000140451	feature_id[10].value <= threshold=8.110000133514404
node_33: feature_name=ENSG00000100297	feature_id[51].value > threshold=111.95999908447266
node_51: feature_name=ENSG00000197061	feature_id[2].value <= threshold=468.4400177001953
node_52: feature_name=ENSG00000125885	feature_id[136].value <= threshold=14.849999904632568
Class: G1	
Rules_14	passed counts:5
node_0: feature_name=ENSG00000170312	feature_id[0].value > threshold=120.56500244140625
node_32: feature_name=ENSG00000140451	feature_id[10].value > threshold=8.110000133514404
node_80: feature_name=ENSG00000105173	feature_id[48].value <= threshold=98.07999801635742
node_81: feature_name=ENSG00000170312	feature_id[0].value > threshold=160.83999633789062
node_87: feature_name=ENSG00000167978	feature_id[187].value > threshold=37.79999923706055
node_93: feature_name=ENSG00000137135	feature_id[199].value > threshold=1.699999988079071
node_97: feature_name=ENSG00000130429	feature_id[361].value > threshold=323.2150115966797
node_125: feature_name=ENSG00000011426	feature_id[309].value > threshold=152.47500610351562
Class: S	
Rules_15	passed counts:5
node_0: feature_name=ENSG00000170312	feature_id[0].value > threshold=120.56500244140625
node_32: feature_name=ENSG00000140451	feature_id[10].value > threshold=8.110000133514404
node_80: feature_name=ENSG00000105173	feature_id[48].value <= threshold=98.07999801635742
node_81: feature_name=ENSG00000170312	feature_id[0].value > threshold=160.83999633789062
node_87: feature_name=ENSG00000167978	feature_id[187].value > threshold=37.79999923706055
node_93: feature_name=ENSG00000137135	feature_id[199].value > threshold=1.699999988079071

node_97: feature_name=ENSG00000130429	feature_id[361].value <= threshold=323.2150115966797
node_98: feature_name=ENSG00000116717	feature_id[135].value > threshold=13.164999961853027
node_100: feature_name=ENSG00000117724	feature_id[94].value > threshold=42.60000038146973
node_114: feature_name=ENSG00000145604	feature_id[390].value > threshold=104.21000289916992
node_122: feature_name=ENSG00000134291	feature_id[338].value <= threshold=582.6800231933594
Class: G2M	
Rules_16	passed counts:5
node_0: feature_name=ENSG00000170312	feature_id[0].value > threshold=120.56500244140625
node_32: feature_name=ENSG00000140451	feature_id[10].value > threshold=8.110000133514404
node_80: feature_name=ENSG00000105173	feature_id[48].value <= threshold=98.07999801635742
node_81: feature_name=ENSG00000170312	feature_id[0].value > threshold=160.83999633789062
node_87: feature_name=ENSG00000167978	feature_id[187].value > threshold=37.79999923706055
node_93: feature_name=ENSG00000137135	feature_id[199].value <= threshold=1.699999988079071
node_94: feature_name=ENSG00000089693	feature_id[453].value <= threshold=316.0400085449219
Class: S	
Rules_17	passed counts:5
node_0: feature_name=ENSG00000170312	feature_id[0].value > threshold=120.56500244140625
node_32: feature_name=ENSG00000140451	feature_id[10].value > threshold=8.110000133514404
node_80: feature_name=ENSG00000105173	feature_id[48].value <= threshold=98.07999801635742
node_81: feature_name=ENSG00000170312	feature_id[0].value > threshold=160.83999633789062
node_87: feature_name=ENSG00000167978	feature_id[187].value <= threshold=37.79999923706055
node_88: feature_name=ENSG00000169490	feature_id[354].value > threshold=62.83999824523926
Class: S	

Rules_18	passed counts:5
node_0: feature_name=ENSG00000170312	feature_id[0].value > threshold=120.56500244140625
node_32: feature_name=ENSG00000140451	feature_id[10].value > threshold=8.110000133514404
node_80: feature_name=ENSG00000105173	feature_id[48].value <= threshold=98.07999801635742
node_81: feature_name=ENSG00000170312	feature_id[0].value <= threshold=160.83999633789062
node_82: feature_name=ENSG00000145604	feature_id[390].value <= threshold=68.21500015258789
Class: G1	
Rules_19	passed counts:5
node_0: feature_name=ENSG00000170312	feature_id[0].value > threshold=120.56500244140625
node_32: feature_name=ENSG00000140451	feature_id[10].value <= threshold=8.110000133514404
node_33: feature_name=ENSG00000100297	feature_id[51].value > threshold=111.95999908447266
node_51: feature_name=ENSG00000197061	feature_id[2].value > threshold=468.4400177001953
node_55: feature_name=ENSG00000012048	feature_id[99].value <= threshold=23.5600004196167
node_56: feature_name=ENSG00000135451	feature_id[93].value > threshold=42.31999969482422
node_60: feature_name=ENSG00000130429	feature_id[361].value > threshold=215.33999633789062
Class: S	
Rules_20	passed counts:4
node_0: feature_name=ENSG00000170312	feature_id[0].value > threshold=120.56500244140625
node_32: feature_name=ENSG00000140451	feature_id[10].value > threshold=8.110000133514404
node_80: feature_name=ENSG00000105173	feature_id[48].value > threshold=98.07999801635742
node_128: feature_name=ENSG00000173207	feature_id[26].value <= threshold=529.4750061035156
node_129: feature_name=ENSG00000112118	feature_id[49].value <= threshold=74.27000045776367

node_130: feature_name=ENSG00000166803	feature_id[98].value > threshold=474.2050018310547
Class: G2M	
Rules_21	passed counts:4
node_0: feature_name=ENSG00000170312	feature_id[0].value > threshold=120.56500244140625
node_32: feature_name=ENSG00000140451	feature_id[10].value <= threshold=8.110000133514404
node_33: feature_name=ENSG00000100297	feature_id[51].value <= threshold=111.95999908447266
node_34: feature_name=ENSG00000150991	feature_id[194].value <= threshold=998.7099914550781
node_35: feature_name=ENSG00000175305	feature_id[38].value > threshold=14.080000400543213
node_45: feature_name=ENSG00000198554	feature_id[457].value > threshold=40.564998626708984
Class: G2M	
Rules_22	passed counts:3
node_0: feature_name=ENSG00000170312	feature_id[0].value > threshold=120.56500244140625
node_32: feature_name=ENSG00000140451	feature_id[10].value > threshold=8.110000133514404
node_80: feature_name=ENSG00000105173	feature_id[48].value > threshold=98.07999801635742
node_128: feature_name=ENSG00000173207	feature_id[26].value > threshold=529.4750061035156
node_134: feature_name=ENSG00000106367	feature_id[195].value <= threshold=167.65499877929688
Class: S	
Rules_23	passed counts:3
node_0: feature_name=ENSG00000170312	feature_id[0].value > threshold=120.56500244140625
node_32: feature_name=ENSG00000140451	feature_id[10].value > threshold=8.110000133514404
node_80: feature_name=ENSG00000105173	feature_id[48].value <= threshold=98.07999801635742
node_81: feature_name=ENSG00000170312	feature_id[0].value > threshold=160.83999633789062

node_87: feature_name=ENSG00000167978	feature_id[187].value > threshold=37.79999923706055
node_93: feature_name=ENSG00000137135	feature_id[199].value > threshold=1.699999988079071
node_97: feature_name=ENSG00000130429	feature_id[361].value > threshold=323.2150115966797
node_125: feature_name=ENSG00000011426	feature_id[309].value <= threshold=152.47500610351562
Class: G2M	
Rules_24	passed counts:3
node_0: feature_name=ENSG00000170312	feature_id[0].value > threshold=120.56500244140625
node_32: feature_name=ENSG00000140451	feature_id[10].value > threshold=8.110000133514404
node_80: feature_name=ENSG00000105173	feature_id[48].value <= threshold=98.07999801635742
node_81: feature_name=ENSG00000170312	feature_id[0].value > threshold=160.83999633789062
node_87: feature_name=ENSG00000167978	feature_id[187].value > threshold=37.79999923706055
node_93: feature_name=ENSG00000137135	feature_id[199].value > threshold=1.699999988079071
node_97: feature_name=ENSG00000130429	feature_id[361].value <= threshold=323.2150115966797
node_98: feature_name=ENSG00000116717	feature_id[135].value > threshold=13.164999961853027
node_100: feature_name=ENSG00000117724	feature_id[94].value > threshold=42.60000038146973
node_114: feature_name=ENSG00000145604	feature_id[390].value > threshold=104.21000289916992
node_122: feature_name=ENSG00000134291	feature_id[338].value > threshold=582.6800231933594
Class: S	
Rules_25	passed counts:3
node_0: feature_name=ENSG00000170312	feature_id[0].value > threshold=120.56500244140625
node_32: feature_name=ENSG00000140451	feature_id[10].value > threshold=8.110000133514404
node_80: feature_name=ENSG00000105173	feature_id[48].value <= threshold=98.07999801635742
node_81: feature_name=ENSG00000170312	feature_id[0].value > threshold=160.83999633789062

node_87: feature_name=ENSG00000167978	feature_id[187].value > threshold=37.79999923706055
node_93: feature_name=ENSG00000137135	feature_id[199].value > threshold=1.699999988079071
node_97: feature_name=ENSG00000130429	feature_id[361].value <= threshold=323.2150115966797
node_98: feature_name=ENSG00000116717	feature_id[135].value > threshold=13.164999961853027
node_100: feature_name=ENSG00000117724	feature_id[94].value <= threshold=42.60000038146973
node_101: feature_name=ENSG00000101003	feature_id[339].value > threshold=18.390000343322754
node_111: feature_name=ENSG00000198331	feature_id[61].value <= threshold=33.454999923706055
Class: G2M	
Rules_26	passed counts:3
node_0: feature_name=ENSG00000170312	feature_id[0].value > threshold=120.56500244140625
node_32: feature_name=ENSG00000140451	feature_id[10].value > threshold=8.110000133514404
node_80: feature_name=ENSG00000105173	feature_id[48].value <= threshold=98.07999801635742
node_81: feature_name=ENSG00000170312	feature_id[0].value <= threshold=160.83999633789062
node_82: feature_name=ENSG00000145604	feature_id[390].value > threshold=68.21500015258789
node_84: feature_name=ENSG00000144554	feature_id[224].value <= threshold=28.510000228881836
Class: S	
Rules_27	passed counts:3
node_0: feature_name=ENSG00000170312	feature_id[0].value <= threshold=120.56500244140625
node_1: feature_name=ENSG00000197061	feature_id[2].value > threshold=794.760009765625
node_27: feature_name=ENSG00000168476	feature_id[217].value > threshold=39.849998474121094
Class: G1	
Rules_28	passed counts:3

node_0: feature_name=ENSG00000170312	feature_id[0].value <= threshold=120.56500244140625
node_1: feature_name=ENSG00000197061	feature_id[2].value <= threshold=794.760009765625
node_2: feature_name=ENSG00000091164	feature_id[188].value <= threshold=29.735000610351562
node_3: feature_name=ENSG00000205352	feature_id[211].value > threshold=60.20000076293945
Class: S	
Rules_29	passed counts:3
node_0: feature_name=ENSG00000170312	feature_id[0].value <= threshold=120.56500244140625
node_1: feature_name=ENSG00000197061	feature_id[2].value <= threshold=794.760009765625
node_2: feature_name=ENSG00000091164	feature_id[188].value <= threshold=29.735000610351562
node_3: feature_name=ENSG00000205352	feature_id[211].value <= threshold=60.20000076293945
node_4: feature_name=ENSG00000256618	feature_id[362].value > threshold=599.9349975585938
Class: G1	
Rules_30	passed counts:3
node_0: feature_name=ENSG00000170312	feature_id[0].value <= threshold=120.56500244140625
node_1: feature_name=ENSG00000197061	feature_id[2].value <= threshold=794.760009765625
node_2: feature_name=ENSG00000091164	feature_id[188].value <= threshold=29.735000610351562
node_3: feature_name=ENSG00000205352	feature_id[211].value <= threshold=60.20000076293945
node_4: feature_name=ENSG00000256618	feature_id[362].value <= threshold=599.9349975585938
node_5: feature_name=ENSG00000128294	feature_id[299].value <= threshold=1429.8699951171875
Class: G2M	
Rules_31	passed counts:2
node_0: feature_name=ENSG00000170312	feature_id[0].value > threshold=120.56500244140625

node_32: feature_name=ENSG00000140451	feature_id[10].value > threshold=8.110000133514404
node_80: feature_name=ENSG00000105173	feature_id[48].value <= threshold=98.07999801635742
node_81: feature_name=ENSG00000170312	feature_id[0].value > threshold=160.83999633789062
node_87: feature_name=ENSG00000167978	feature_id[187].value > threshold=37.79999923706055
node_93: feature_name=ENSG00000137135	feature_id[199].value > threshold=1.699999988079071
node_97: feature_name=ENSG00000130429	feature_id[361].value <= threshold=323.2150115966797
node_98: feature_name=ENSG00000116717	feature_id[135].value > threshold=13.164999961853027
node_100: feature_name=ENSG00000117724	feature_id[94].value <= threshold=42.60000038146973
node_101: feature_name=ENSG00000101003	feature_id[339].value <= threshold=18.390000343322754
node_102: feature_name=ENSG00000278259	feature_id[436].value > threshold=66.98500061035156
Class: S	
Rules_32	passed counts:2
node_0: feature_name=ENSG00000170312	feature_id[0].value > threshold=120.56500244140625
node_32: feature_name=ENSG00000140451	feature_id[10].value > threshold=8.110000133514404
node_80: feature_name=ENSG00000105173	feature_id[48].value <= threshold=98.07999801635742
node_81: feature_name=ENSG00000170312	feature_id[0].value > threshold=160.83999633789062
node_87: feature_name=ENSG00000167978	feature_id[187].value > threshold=37.79999923706055
node_93: feature_name=ENSG00000137135	feature_id[199].value > threshold=1.699999988079071
node_97: feature_name=ENSG00000130429	feature_id[361].value <= threshold=323.2150115966797
node_98: feature_name=ENSG00000116717	feature_id[135].value > threshold=13.164999961853027
node_100: feature_name=ENSG00000117724	feature_id[94].value <= threshold=42.60000038146973
node_101: feature_name=ENSG00000101003	feature_id[339].value <= threshold=18.390000343322754
node_102: feature_name=ENSG00000278259	feature_id[436].value <= threshold=66.98500061035156
node_103: feature_name=ENSG00000237649	feature_id[104].value <= threshold=2.6549999713897705

node_104: feature_name=ENSG00000167553	feature_id[101].value <= threshold=1953.925048828125
Class: S	
Rules_33	passed counts:2
node_0: feature_name=ENSG00000170312	feature_id[0].value > threshold=120.56500244140625
node_32: feature_name=ENSG00000140451	feature_id[10].value > threshold=8.110000133514404
node_80: feature_name=ENSG00000105173	feature_id[48].value <= threshold=98.07999801635742
node_81: feature_name=ENSG00000170312	feature_id[0].value > threshold=160.83999633789062
node_87: feature_name=ENSG00000167978	feature_id[187].value > threshold=37.79999923706055
node_93: feature_name=ENSG00000137135	feature_id[199].value > threshold=1.699999988079071
node_97: feature_name=ENSG00000130429	feature_id[361].value <= threshold=323.2150115966797
node_98: feature_name=ENSG00000116717	feature_id[135].value <= threshold=13.164999961853027
Class: S	
Rules_34	passed counts:2
node_0: feature_name=ENSG00000170312	feature_id[0].value > threshold=120.56500244140625
node_32: feature_name=ENSG00000140451	feature_id[10].value <= threshold=8.110000133514404
node_33: feature_name=ENSG00000100297	feature_id[51].value > threshold=111.95999908447266
node_51: feature_name=ENSG00000197061	feature_id[2].value > threshold=468.4400177001953
node_55: feature_name=ENSG00000012048	feature_id[99].value > threshold=23.5600004196167
node_63: feature_name=ENSG00000127564	feature_id[107].value > threshold=22.22499942779541
node_67: feature_name=ENSG00000167601	feature_id[242].value > threshold=391.2899932861328
Class: G2M	
Rules_35	passed counts:2

node_0: feature_name=ENSG00000170312	feature_id[0].value > threshold=120.56500244140625
node_32: feature_name=ENSG00000140451	feature_id[10].value <= threshold=8.110000133514404
node_33: feature_name=ENSG00000100297	feature_id[51].value > threshold=111.95999908447266
node_51: feature_name=ENSG00000197061	feature_id[2].value > threshold=468.4400177001953
node_55: feature_name=ENSG00000012048	feature_id[99].value > threshold=23.5600004196167
node_63: feature_name=ENSG00000127564	feature_id[107].value > threshold=22.22499942779541
node_67: feature_name=ENSG00000167601	feature_id[242].value <= threshold=391.2899932861328
node_68: feature_name=ENSG00000259848	feature_id[68].value > threshold=239.00499725341797
Class: G2M	
Rules_36	passed counts:2
node_0: feature_name=ENSG00000170312	feature_id[0].value > threshold=120.56500244140625
node_32: feature_name=ENSG00000140451	feature_id[10].value <= threshold=8.110000133514404
node_33: feature_name=ENSG00000100297	feature_id[51].value > threshold=111.95999908447266
node_51: feature_name=ENSG00000197061	feature_id[2].value > threshold=468.4400177001953
node_55: feature_name=ENSG00000012048	feature_id[99].value > threshold=23.5600004196167
node_63: feature_name=ENSG00000127564	feature_id[107].value <= threshold=22.22499942779541
node_64: feature_name=ENSG00000161800	feature_id[368].value > threshold=25.505000114440918
Class: G2M	
Rules_37	passed counts:2
node_0: feature_name=ENSG00000170312	feature_id[0].value > threshold=120.56500244140625
node_32: feature_name=ENSG00000140451	feature_id[10].value <= threshold=8.110000133514404
node_33: feature_name=ENSG00000100297	feature_id[51].value > threshold=111.95999908447266
node_51: feature_name=ENSG00000197061	feature_id[2].value > threshold=468.4400177001953

node_55: feature_name=ENSG00000012048	feature_id[99].value <= threshold=23.5600004196167
node_56: feature_name=ENSG00000135451	feature_id[93].value <= threshold=42.31999969482422
node_57: feature_name=ENSG00000165502	feature_id[258].value > threshold=488.7150115966797
Class: G1	
Rules_38	passed counts:2
node_0: feature_name=ENSG00000170312	feature_id[0].value > threshold=120.56500244140625
node_32: feature_name=ENSG00000140451	feature_id[10].value <= threshold=8.110000133514404
node_33: feature_name=ENSG00000100297	feature_id[51].value > threshold=111.95999908447266
node_51: feature_name=ENSG00000197061	feature_id[2].value <= threshold=468.4400177001953
node_52: feature_name=ENSG00000125885	feature_id[136].value > threshold=14.849999904632568
Class: S	
Rules_39	passed counts:2
node_0: feature_name=ENSG00000170312	feature_id[0].value > threshold=120.56500244140625
node_32: feature_name=ENSG00000140451	feature_id[10].value <= threshold=8.110000133514404
node_33: feature_name=ENSG00000100297	feature_id[51].value <= threshold=111.95999908447266
node_34: feature_name=ENSG00000150991	feature_id[194].value > threshold=998.7099914550781
node_48: feature_name=ENSG00000205352	feature_id[211].value <= threshold=73.43000030517578
Class: G2M	
Rules_40	passed counts:2
node_0: feature_name=ENSG00000170312	feature_id[0].value > threshold=120.56500244140625
node_32: feature_name=ENSG00000140451	feature_id[10].value <= threshold=8.110000133514404
node_33: feature_name=ENSG00000100297	feature_id[51].value <= threshold=111.95999908447266

node_34: feature_name=ENSG00000150991	feature_id[194].value <= threshold=998.7099914550781
node_35: feature_name=ENSG00000175305	feature_id[38].value <= threshold=14.080000400543213
node_36: feature_name=ENSG00000127483	feature_id[191].value > threshold=215.8800048828125
node_42: feature_name=ENSG00000135185	feature_id[409].value <= threshold=32.945000648498535
Class: G1	
Rules_41	passed counts:2
node_0: feature_name=ENSG00000170312	feature_id[0].value > threshold=120.56500244140625
node_32: feature_name=ENSG00000140451	feature_id[10].value <= threshold=8.110000133514404
node_33: feature_name=ENSG00000100297	feature_id[51].value <= threshold=111.95999908447266
node_34: feature_name=ENSG00000150991	feature_id[194].value <= threshold=998.7099914550781
node_35: feature_name=ENSG00000175305	feature_id[38].value <= threshold=14.080000400543213
node_36: feature_name=ENSG00000127483	feature_id[191].value <= threshold=215.8800048828125
node_37: feature_name=ENSG00000171793	feature_id[183].value > threshold=335.6300048828125
Class: S	
Rules_42	passed counts:2
node_0: feature_name=ENSG00000170312	feature_id[0].value <= threshold=120.56500244140625
node_1: feature_name=ENSG00000197061	feature_id[2].value <= threshold=794.760009765625
node_2: feature_name=ENSG00000091164	feature_id[188].value > threshold=29.735000610351562
node_10: feature_name=ENSG00000093009	feature_id[59].value > threshold=198.45000457763672
Class: S	
Rules_43	passed counts:1
node_0: feature_name=ENSG00000170312	feature_id[0].value > threshold=120.56500244140625

node_32: feature_name=ENSG00000140451	feature_id[10].value > threshold=8.110000133514404
node_80: feature_name=ENSG00000105173	feature_id[48].value > threshold=98.07999801635742
node_128: feature_name=ENSG00000173207	feature_id[26].value <= threshold=529.4750061035156
node_129: feature_name=ENSG00000112118	feature_id[49].value <= threshold=74.27000045776367
node_130: feature_name=ENSG00000166803	feature_id[98].value <= threshold=474.2050018310547
Class: S	
Rules_44	passed counts:1
node_0: feature_name=ENSG00000170312	feature_id[0].value > threshold=120.56500244140625
node_32: feature_name=ENSG00000140451	feature_id[10].value > threshold=8.110000133514404
node_80: feature_name=ENSG00000105173	feature_id[48].value <= threshold=98.07999801635742
node_81: feature_name=ENSG00000170312	feature_id[0].value > threshold=160.83999633789062
node_87: feature_name=ENSG00000167978	feature_id[187].value > threshold=37.79999923706055
node_93: feature_name=ENSG00000137135	feature_id[199].value > threshold=1.699999988079071
node_97: feature_name=ENSG00000130429	feature_id[361].value <= threshold=323.2150115966797
node_98: feature_name=ENSG00000116717	feature_id[135].value > threshold=13.164999961853027
node_100: feature_name=ENSG00000117724	feature_id[94].value > threshold=42.60000038146973
node_114: feature_name=ENSG00000145604	feature_id[390].value <= threshold=104.21000289916992
node_115: feature_name=ENSG00000183741	feature_id[331].value > threshold=45.329999923706055
Class: S	
Rules_45	passed counts:1
node_0: feature_name=ENSG00000170312	feature_id[0].value > threshold=120.56500244140625
node_32: feature_name=ENSG00000140451	feature_id[10].value > threshold=8.110000133514404
node_80: feature_name=ENSG00000105173	feature_id[48].value <= threshold=98.07999801635742

node_81: feature_name=ENSG00000170312	feature_id[0].value > threshold=160.83999633789062
node_87: feature_name=ENSG00000167978	feature_id[187].value > threshold=37.79999923706055
node_93: feature_name=ENSG00000137135	feature_id[199].value > threshold=1.699999988079071
node_97: feature_name=ENSG00000130429	feature_id[361].value <= threshold=323.2150115966797
node_98: feature_name=ENSG00000116717	feature_id[135].value > threshold=13.164999961853027
node_100: feature_name=ENSG00000117724	feature_id[94].value > threshold=42.60000038146973
node_114: feature_name=ENSG00000145604	feature_id[390].value <= threshold=104.21000289916992
node_115: feature_name=ENSG00000183741	feature_id[331].value <= threshold=45.329999923706055
node_116: feature_name=ENSG00000122565	feature_id[346].value > threshold=237.02999877929688
node_118: feature_name=ENSG00000111859	feature_id[246].value > threshold=4.139999866485596
Class: G2M	
Rules_46	passed counts:1
node_0: feature_name=ENSG00000170312	feature_id[0].value > threshold=120.56500244140625
node_32: feature_name=ENSG00000140451	feature_id[10].value > threshold=8.110000133514404
node_80: feature_name=ENSG00000105173	feature_id[48].value <= threshold=98.07999801635742
node_81: feature_name=ENSG00000170312	feature_id[0].value > threshold=160.83999633789062
node_87: feature_name=ENSG00000167978	feature_id[187].value > threshold=37.79999923706055
node_93: feature_name=ENSG00000137135	feature_id[199].value > threshold=1.699999988079071
node_97: feature_name=ENSG00000130429	feature_id[361].value <= threshold=323.2150115966797
node_98: feature_name=ENSG00000116717	feature_id[135].value > threshold=13.164999961853027
node_100: feature_name=ENSG00000117724	feature_id[94].value > threshold=42.60000038146973
node_114: feature_name=ENSG00000145604	feature_id[390].value <= threshold=104.21000289916992
node_115: feature_name=ENSG00000183741	feature_id[331].value <= threshold=45.329999923706055
node_116: feature_name=ENSG00000122565	feature_id[346].value > threshold=237.02999877929688

node_118: feature_name=ENSG00000111859	feature_id[246].value <= threshold=4.139999866485596
Class: S	
Rules_47	passed counts:1
node_0: feature_name=ENSG00000170312	feature_id[0].value > threshold=120.56500244140625
node_32: feature_name=ENSG00000140451	feature_id[10].value > threshold=8.110000133514404
node_80: feature_name=ENSG00000105173	feature_id[48].value <= threshold=98.07999801635742
node_81: feature_name=ENSG00000170312	feature_id[0].value > threshold=160.83999633789062
node_87: feature_name=ENSG00000167978	feature_id[187].value > threshold=37.79999923706055
node_93: feature_name=ENSG00000137135	feature_id[199].value > threshold=1.699999988079071
node_97: feature_name=ENSG00000130429	feature_id[361].value <= threshold=323.2150115966797
node_98: feature_name=ENSG00000116717	feature_id[135].value > threshold=13.164999961853027
node_100: feature_name=ENSG00000117724	feature_id[94].value <= threshold=42.60000038146973
node_101: feature_name=ENSG00000101003	feature_id[339].value <= threshold=18.390000343322754
node_102: feature_name=ENSG00000278259	feature_id[436].value <= threshold=66.98500061035156
node_103: feature_name=ENSG00000237649	feature_id[104].value > threshold=2.6549999713897705
node_107: feature_name=ENSG00000183726	feature_id[461].value <= threshold=36.849998474121094
Class: S	
Rules_48	passed counts:1
node_0: feature_name=ENSG00000170312	feature_id[0].value > threshold=120.56500244140625
node_32: feature_name=ENSG00000140451	feature_id[10].value > threshold=8.110000133514404
node_80: feature_name=ENSG00000105173	feature_id[48].value <= threshold=98.07999801635742
node_81: feature_name=ENSG00000170312	feature_id[0].value > threshold=160.83999633789062
node_87: feature_name=ENSG00000167978	feature_id[187].value > threshold=37.79999923706055

node_93: feature_name=ENSG00000137135	feature_id[199].value > threshold=1.699999988079071
node_97: feature_name=ENSG00000130429	feature_id[361].value <= threshold=323.2150115966797
node_98: feature_name=ENSG00000116717	feature_id[135].value > threshold=13.164999961853027
node_100: feature_name=ENSG00000117724	feature_id[94].value <= threshold=42.60000038146973
node_101: feature_name=ENSG00000101003	feature_id[339].value <= threshold=18.390000343322754
node_102: feature_name=ENSG00000278259	feature_id[436].value <= threshold=66.98500061035156
node_103: feature_name=ENSG00000237649	feature_id[104].value <= threshold=2.6549999713897705
node_104: feature_name=ENSG00000167553	feature_id[101].value > threshold=1953.925048828125
Class: G2M	
Rules_49	passed counts:1
node_0: feature_name=ENSG00000170312	feature_id[0].value > threshold=120.56500244140625
node_32: feature_name=ENSG00000140451	feature_id[10].value > threshold=8.110000133514404
node_80: feature_name=ENSG00000105173	feature_id[48].value <= threshold=98.07999801635742
node_81: feature_name=ENSG00000170312	feature_id[0].value > threshold=160.83999633789062
node_87: feature_name=ENSG00000167978	feature_id[187].value > threshold=37.79999923706055
node_93: feature_name=ENSG00000137135	feature_id[199].value <= threshold=1.699999988079071
node_94: feature_name=ENSG00000089693	feature_id[453].value > threshold=316.0400085449219
Class: G2M	
Rules_50	passed counts:1
node_0: feature_name=ENSG00000170312	feature_id[0].value > threshold=120.56500244140625
node_32: feature_name=ENSG00000140451	feature_id[10].value > threshold=8.110000133514404
node_80: feature_name=ENSG00000105173	feature_id[48].value <= threshold=98.07999801635742
node_81: feature_name=ENSG00000170312	feature_id[0].value > threshold=160.83999633789062

node_87: feature_name=ENSG00000167978	feature_id[187].value <= threshold=37.79999923706055
node_88: feature_name=ENSG00000169490	feature_id[354].value <= threshold=62.83999824523926
node_89: feature_name=ENSG00000135476	feature_id[129].value > threshold=13.0
Class: G1	
Rules_51	passed counts:1
node_0: feature_name=ENSG00000170312	feature_id[0].value > threshold=120.56500244140625
node_32: feature_name=ENSG00000140451	feature_id[10].value > threshold=8.110000133514404
node_80: feature_name=ENSG00000105173	feature_id[48].value <= threshold=98.07999801635742
node_81: feature_name=ENSG00000170312	feature_id[0].value > threshold=160.83999633789062
node_87: feature_name=ENSG00000167978	feature_id[187].value <= threshold=37.79999923706055
node_88: feature_name=ENSG00000169490	feature_id[354].value <= threshold=62.83999824523926
node_89: feature_name=ENSG00000135476	feature_id[129].value <= threshold=13.0
Class: G2M	
Rules_52	passed counts:1
node_0: feature_name=ENSG00000170312	feature_id[0].value > threshold=120.56500244140625
node_32: feature_name=ENSG00000140451	feature_id[10].value > threshold=8.110000133514404
node_80: feature_name=ENSG00000105173	feature_id[48].value <= threshold=98.07999801635742
node_81: feature_name=ENSG00000170312	feature_id[0].value <= threshold=160.83999633789062
node_82: feature_name=ENSG00000145604	feature_id[390].value > threshold=68.21500015258789
node_84: feature_name=ENSG00000144554	feature_id[224].value > threshold=28.510000228881836
Class: G2M	
Rules_53	passed counts:1

node_0: feature_name=ENSG00000170312	feature_id[0].value > threshold=120.56500244140625
node_32: feature_name=ENSG00000140451	feature_id[10].value <= threshold=8.110000133514404
node_33: feature_name=ENSG00000100297	feature_id[51].value > threshold=111.95999908447266
node_51: feature_name=ENSG00000197061	feature_id[2].value > threshold=468.4400177001953
node_55: feature_name=ENSG00000012048	feature_id[99].value > threshold=23.5600004196167
node_63: feature_name=ENSG00000127564	feature_id[107].value > threshold=22.22499942779541
node_67: feature_name=ENSG00000167601	feature_id[242].value <= threshold=391.2899932861328
node_68: feature_name=ENSG00000259848	feature_id[68].value <= threshold=239.00499725341797
node_69: feature_name=ENSG00000117650	feature_id[84].value > threshold=76.625
Class: G1	
Rules_54	passed counts:1
node_0: feature_name=ENSG00000170312	feature_id[0].value > threshold=120.56500244140625
node_32: feature_name=ENSG00000140451	feature_id[10].value <= threshold=8.110000133514404
node_33: feature_name=ENSG00000100297	feature_id[51].value > threshold=111.95999908447266
node_51: feature_name=ENSG00000197061	feature_id[2].value > threshold=468.4400177001953
node_55: feature_name=ENSG00000012048	feature_id[99].value > threshold=23.5600004196167
node_63: feature_name=ENSG00000127564	feature_id[107].value > threshold=22.22499942779541
node_67: feature_name=ENSG00000167601	feature_id[242].value <= threshold=391.2899932861328
node_68: feature_name=ENSG00000259848	feature_id[68].value <= threshold=239.00499725341797
node_69: feature_name=ENSG00000117650	feature_id[84].value <= threshold=76.625
node_70: feature_name=ENSG00000185361	feature_id[58].value > threshold=12.335000038146973
Class: G2M	
Rules_55	passed counts:1

node_0: feature_name=ENSG00000170312	feature_id[0].value > threshold=120.56500244140625
node_32: feature_name=ENSG00000140451	feature_id[10].value <= threshold=8.110000133514404
node_33: feature_name=ENSG00000100297	feature_id[51].value > threshold=111.95999908447266
node_51: feature_name=ENSG00000197061	feature_id[2].value > threshold=468.4400177001953
node_55: feature_name=ENSG00000012048	feature_id[99].value > threshold=23.5600004196167
node_63: feature_name=ENSG00000127564	feature_id[107].value > threshold=22.22499942779541
node_67: feature_name=ENSG00000167601	feature_id[242].value <= threshold=391.2899932861328
node_68: feature_name=ENSG00000259848	feature_id[68].value <= threshold=239.00499725341797
node_69: feature_name=ENSG00000117650	feature_id[84].value <= threshold=76.625
node_70: feature_name=ENSG00000185361	feature_id[58].value <= threshold=12.335000038146973
node_71: feature_name=ENSG00000121152	feature_id[33].value > threshold=224.78500366210938
Class: G2M	
Rules_56	passed counts:1
node_0: feature_name=ENSG00000170312	feature_id[0].value > threshold=120.56500244140625
node_32: feature_name=ENSG00000140451	feature_id[10].value <= threshold=8.110000133514404
node_33: feature_name=ENSG00000100297	feature_id[51].value > threshold=111.95999908447266
node_51: feature_name=ENSG00000197061	feature_id[2].value > threshold=468.4400177001953
node_55: feature_name=ENSG00000012048	feature_id[99].value > threshold=23.5600004196167
node_63: feature_name=ENSG00000127564	feature_id[107].value > threshold=22.22499942779541
node_67: feature_name=ENSG00000167601	feature_id[242].value <= threshold=391.2899932861328
node_68: feature_name=ENSG00000259848	feature_id[68].value <= threshold=239.00499725341797
node_69: feature_name=ENSG00000117650	feature_id[84].value <= threshold=76.625
node_70: feature_name=ENSG00000185361	feature_id[58].value <= threshold=12.335000038146973
node_71: feature_name=ENSG00000121152	feature_id[33].value <= threshold=224.78500366210938

node_72: feature_name=ENSG00000151503	feature_id[451].value <= threshold=0.26499998569488525
Class: G2M	
Rules_57	passed counts:1
node_0: feature_name=ENSG00000170312	feature_id[0].value > threshold=120.56500244140625
node_32: feature_name=ENSG00000140451	feature_id[10].value <= threshold=8.110000133514404
node_33: feature_name=ENSG00000100297	feature_id[51].value > threshold=111.95999908447266
node_51: feature_name=ENSG00000197061	feature_id[2].value > threshold=468.4400177001953
node_55: feature_name=ENSG00000012048	feature_id[99].value > threshold=23.5600004196167
node_63: feature_name=ENSG00000127564	feature_id[107].value <= threshold=22.22499942779541
node_64: feature_name=ENSG00000161800	feature_id[368].value <= threshold=25.505000114440918
Class: G1	
Rules_58	passed counts:1
node_0: feature_name=ENSG00000170312	feature_id[0].value > threshold=120.56500244140625
node_32: feature_name=ENSG00000140451	feature_id[10].value <= threshold=8.110000133514404
node_33: feature_name=ENSG00000100297	feature_id[51].value <= threshold=111.95999908447266
node_34: feature_name=ENSG00000150991	feature_id[194].value <= threshold=998.7099914550781
node_35: feature_name=ENSG00000175305	feature_id[38].value <= threshold=14.080000400543213
node_36: feature_name=ENSG00000127483	feature_id[191].value > threshold=215.8800048828125
node_42: feature_name=ENSG00000135185	feature_id[409].value > threshold=32.945000648498535
Class: S	
Rules_59	passed counts:1
node_0: feature_name=ENSG00000170312	feature_id[0].value > threshold=120.56500244140625

node_32: feature_name=ENSG00000140451	feature_id[10].value <= threshold=8.110000133514404
node_33: feature_name=ENSG00000100297	feature_id[51].value <= threshold=111.95999908447266
node_34: feature_name=ENSG00000150991	feature_id[194].value <= threshold=998.7099914550781
node_35: feature_name=ENSG00000175305	feature_id[38].value <= threshold=14.080000400543213
node_36: feature_name=ENSG00000127483	feature_id[191].value <= threshold=215.8800048828125
node_37: feature_name=ENSG00000171793	feature_id[183].value <= threshold=335.6300048828125
node_38: feature_name=ENSG00000130429	feature_id[361].value > threshold=291.3249969482422
Class: S	
Rules_60	passed counts:1
node_0: feature_name=ENSG00000170312	feature_id[0].value <= threshold=120.56500244140625
node_1: feature_name=ENSG00000197061	feature_id[2].value > threshold=794.760009765625
node_27: feature_name=ENSG00000168476	feature_id[217].value <= threshold=39.849998474121094
node_28: feature_name=ENSG00000034510	feature_id[424].value > threshold=9527.485107421875
Class: G1	
Rules_61	passed counts:1
node_0: feature_name=ENSG00000170312	feature_id[0].value <= threshold=120.56500244140625
node_1: feature_name=ENSG00000197061	feature_id[2].value <= threshold=794.760009765625
node_2: feature_name=ENSG00000091164	feature_id[188].value > threshold=29.735000610351562
node_10: feature_name=ENSG00000093009	feature_id[59].value <= threshold=198.45000457763672
node_11: feature_name=ENSG00000154640	feature_id[353].value > threshold=326.40999603271484
Class: S	
Rules_62	passed counts:1

node_0: feature_name=ENSG00000170312	feature_id[0].value <= threshold=120.56500244140625
node_1: feature_name=ENSG00000197061	feature_id[2].value <= threshold=794.760009765625
node_2: feature_name=ENSG00000091164	feature_id[188].value > threshold=29.735000610351562
node_10: feature_name=ENSG00000093009	feature_id[59].value <= threshold=198.45000457763672
node_11: feature_name=ENSG00000154640	feature_id[353].value <= threshold=326.40999603271484
node_12: feature_name=ENSG00000106462	feature_id[284].value > threshold=37.8650016784668
Class: S	
Rules_63	passed counts:1
node_0: feature_name=ENSG00000170312	feature_id[0].value <= threshold=120.56500244140625
node_1: feature_name=ENSG00000197061	feature_id[2].value <= threshold=794.760009765625
node_2: feature_name=ENSG00000091164	feature_id[188].value > threshold=29.735000610351562
node_10: feature_name=ENSG00000093009	feature_id[59].value <= threshold=198.45000457763672
node_11: feature_name=ENSG00000154640	feature_id[353].value <= threshold=326.40999603271484
node_12: feature_name=ENSG00000106462	feature_id[284].value <= threshold=37.8650016784668
node_13: feature_name=ENSG00000138376	feature_id[137].value > threshold=85.30999755859375
Class: S	
Rules_64	passed counts:1
node_0: feature_name=ENSG00000170312	feature_id[0].value <= threshold=120.56500244140625
node_1: feature_name=ENSG00000197061	feature_id[2].value <= threshold=794.760009765625
node_2: feature_name=ENSG00000091164	feature_id[188].value > threshold=29.735000610351562
node_10: feature_name=ENSG00000093009	feature_id[59].value <= threshold=198.45000457763672
node_11: feature_name=ENSG00000154640	feature_id[353].value <= threshold=326.40999603271484
node_12: feature_name=ENSG00000106462	feature_id[284].value <= threshold=37.8650016784668

node_13: feature_name=ENSG00000138376	feature_id[137].value <= threshold=85.30999755859375
node_14: feature_name=ENSG00000173207	feature_id[26].value > threshold=800.375
node_20: feature_name=ENSG00000186871	feature_id[455].value > threshold=14.990000247955322
Class: G1	
Rules_65	passed counts:1
node_0: feature_name=ENSG00000170312	feature_id[0].value <= threshold=120.56500244140625
node_1: feature_name=ENSG00000197061	feature_id[2].value <= threshold=794.760009765625
node_2: feature_name=ENSG00000091164	feature_id[188].value > threshold=29.735000610351562
node_10: feature_name=ENSG00000093009	feature_id[59].value <= threshold=198.45000457763672
node_11: feature_name=ENSG00000154640	feature_id[353].value <= threshold=326.40999603271484
node_12: feature_name=ENSG00000106462	feature_id[284].value <= threshold=37.8650016784668
node_13: feature_name=ENSG00000138376	feature_id[137].value <= threshold=85.30999755859375
node_14: feature_name=ENSG00000173207	feature_id[26].value > threshold=800.375
node_20: feature_name=ENSG00000186871	feature_id[455].value <= threshold=14.990000247955322
Class: G2M	
Rules_66	passed counts:1
node_0: feature_name=ENSG00000170312	feature_id[0].value <= threshold=120.56500244140625
node_1: feature_name=ENSG00000197061	feature_id[2].value <= threshold=794.760009765625
node_2: feature_name=ENSG00000091164	feature_id[188].value > threshold=29.735000610351562
node_10: feature_name=ENSG00000093009	feature_id[59].value <= threshold=198.45000457763672
node_11: feature_name=ENSG00000154640	feature_id[353].value <= threshold=326.40999603271484
node_12: feature_name=ENSG00000106462	feature_id[284].value <= threshold=37.8650016784668
node_13: feature_name=ENSG00000138376	feature_id[137].value <= threshold=85.30999755859375

node_14: feature_name=ENSG00000173207	feature_id[26].value <= threshold=800.375
node_15: feature_name=ENSG00000232388	feature_id[360].value > threshold=253.9800033569336
node_17: feature_name=ENSG00000101608	feature_id[330].value > threshold=883.2449951171875
Class: S	
Rules_67	passed counts:1
node_0: feature_name=ENSG00000170312	feature_id[0].value <= threshold=120.56500244140625
node_1: feature_name=ENSG00000197061	feature_id[2].value <= threshold=794.760009765625
node_2: feature_name=ENSG00000091164	feature_id[188].value > threshold=29.735000610351562
node_10: feature_name=ENSG00000093009	feature_id[59].value <= threshold=198.45000457763672
node_11: feature_name=ENSG00000154640	feature_id[353].value <= threshold=326.40999603271484
node_12: feature_name=ENSG00000106462	feature_id[284].value <= threshold=37.8650016784668
node_13: feature_name=ENSG00000138376	feature_id[137].value <= threshold=85.30999755859375
node_14: feature_name=ENSG00000173207	feature_id[26].value <= threshold=800.375
node_15: feature_name=ENSG00000232388	feature_id[360].value > threshold=253.9800033569336
node_17: feature_name=ENSG00000101608	feature_id[330].value <= threshold=883.2449951171875
Class: G1	
Rules_68	passed counts:1
node_0: feature_name=ENSG00000170312	feature_id[0].value <= threshold=120.56500244140625
node_1: feature_name=ENSG00000197061	feature_id[2].value <= threshold=794.760009765625
node_2: feature_name=ENSG00000091164	feature_id[188].value <= threshold=29.735000610351562
node_3: feature_name=ENSG00000205352	feature_id[211].value <= threshold=60.20000076293945
node_4: feature_name=ENSG00000256618	feature_id[362].value <= threshold=599.9349975585938
node_5: feature_name=ENSG00000128294	feature_id[299].value > threshold=1429.8699951171875

Class: G1	

(3) Rules on features selected by SHAP by LightGBM

Rules_0	passed counts:311
node_0: feature_name=ENSG00000170312	feature_id[0].value <= threshold=120.56500244140625
node_1: feature_name=ENSG00000197061	feature_id[1].value <= threshold=794.760009765625
node_2: feature_name=ENSG00000145425	feature_id[46].value <= threshold=4353.8848876953125
node_3: feature_name=ENSG00000127948	feature_id[39].value > threshold=4.385000109672546
node_9: feature_name=ENSG00000150991	feature_id[6].value > threshold=72.85999965667725
node_11: feature_name=ENSG00000014138	feature_id[17].value <= threshold=102.51499938964844
node_12: feature_name=ENSG00000087077	feature_id[71].value > threshold=35.76500129699707
node_16: feature_name=ENSG00000007968	feature_id[68].value <= threshold=20.079999923706055
Class: G1	
Rules_1	passed counts:233
node_0: feature_name=ENSG00000170312	feature_id[0].value > threshold=120.56500244140625
node_30: feature_name=ENSG00000140451	feature_id[3].value > threshold=8.110000133514404
node_88: feature_name=ENSG00000105173	feature_id[8].value <= threshold=98.07999801635742
node_89: feature_name=ENSG00000170312	feature_id[0].value > threshold=160.83999633789062
node_95: feature_name=ENSG00000167978	feature_id[48].value > threshold=37.79999923706055
node_101: feature_name=ENSG00000130429	feature_id[93].value <= threshold=323.2150115966797
node_102: feature_name=ENSG00000117724	feature_id[61].value > threshold=42.60000038146973
node_118: feature_name=ENSG00000276368	feature_id[29].value <= threshold=333.0249938964844
node_119: feature_name=ENSG00000122952	feature_id[54].value <= threshold=716.2649841308594
node_120: feature_name=ENSG00000092853	feature_id[75].value <= threshold=40.64500045776367

node_121: feature_name=ENSG00000177426	feature_id[37].value > threshold=32.579999923706055
node_125: feature_name=ENSG00000178913	feature_id[76].value <= threshold=114.54999923706055
node_126: feature_name=ENSG00000146670	feature_id[66].value > threshold=33.939998626708984
Class: G2M	
Rules_2	passed counts:201
node_0: feature_name=ENSG00000170312	feature_id[0].value > threshold=120.56500244140625
node_30: feature_name=ENSG00000140451	feature_id[3].value <= threshold=8.110000133514404
node_31: feature_name=ENSG00000100297	feature_id[28].value > threshold=111.95999908447266
node_51: feature_name=ENSG00000197061	feature_id[1].value > threshold=468.4400177001953
node_55: feature_name=ENSG00000012048	feature_id[12].value > threshold=23.5600004196167
node_67: feature_name=ENSG00000127564	feature_id[86].value > threshold=22.22499942779541
node_71: feature_name=ENSG00000167601	feature_id[30].value <= threshold=391.2899932861328
node_72: feature_name=ENSG00000229089	feature_id[5].value <= threshold=106.125
node_73: feature_name=ENSG00000117399	feature_id[21].value <= threshold=510.64500427246094
node_74: feature_name=ENSG00000185361	feature_id[97].value <= threshold=12.335000038146973
node_75: feature_name=ENSG00000146670	feature_id[66].value > threshold=38.46000099182129
node_79: feature_name=ENSG00000173207	feature_id[27].value <= threshold=653.6899719238281
Class: S	
Rules_3	passed counts:46
node_0: feature_name=ENSG00000170312	feature_id[0].value > threshold=120.56500244140625
node_30: feature_name=ENSG00000140451	feature_id[3].value > threshold=8.110000133514404
node_88: feature_name=ENSG00000105173	feature_id[8].value <= threshold=98.07999801635742
node_89: feature_name=ENSG00000170312	feature_id[0].value > threshold=160.83999633789062

node_95: feature_name=ENSG00000167978	feature_id[48].value > threshold=37.79999923706055
node_101: feature_name=ENSG00000130429	feature_id[93].value <= threshold=323.2150115966797
node_102: feature_name=ENSG00000117724	feature_id[61].value <= threshold=42.60000038146973
node_103: feature_name=ENSG00000101003	feature_id[65].value <= threshold=18.390000343322754
node_104: feature_name=ENSG00000112029	feature_id[18].value <= threshold=93.15499877929688
node_105: feature_name=ENSG00000114346	feature_id[74].value > threshold=8.815000057220459
node_107: feature_name=ENSG00000149548	feature_id[83].value <= threshold=6.200000047683716
node_108: feature_name=ENSG00000128340	feature_id[85].value > threshold=5.1000001430511475
Class: G2M	
Rules_4	passed counts:36
node_0: feature_name=ENSG00000170312	feature_id[0].value > threshold=120.56500244140625
node_30: feature_name=ENSG00000140451	feature_id[3].value <= threshold=8.110000133514404
node_31: feature_name=ENSG00000100297	feature_id[28].value <= threshold=111.95999908447266
node_32: feature_name=ENSG00000150991	feature_id[6].value <= threshold=998.7099914550781
node_33: feature_name=ENSG00000175305	feature_id[35].value <= threshold=14.080000400543213
node_34: feature_name=ENSG00000186767	feature_id[89].value <= threshold=22.6850004196167
node_35: feature_name=ENSG00000185361	feature_id[97].value <= threshold=16.954999923706055
node_36: feature_name=ENSG00000177426	feature_id[37].value <= threshold=286.0399932861328
Class: G2M	
Rules_5	passed counts:21
node_0: feature_name=ENSG00000170312	feature_id[0].value > threshold=120.56500244140625
node_30: feature_name=ENSG00000140451	feature_id[3].value > threshold=8.110000133514404
node_88: feature_name=ENSG00000105173	feature_id[8].value > threshold=98.07999801635742

node_140: feature_name=ENSG00000173207	feature_id[27].value <= threshold=529.4750061035156
node_141: feature_name=ENSG00000185361	feature_id[97].value <= threshold=7.180000066757202
node_142: feature_name=ENSG00000128340	feature_id[85].value > threshold=16.95500087738037
Class: S	
Rules_6	passed counts:17
node_0: feature_name=ENSG00000170312	feature_id[0].value > threshold=120.56500244140625
node_30: feature_name=ENSG00000140451	feature_id[3].value <= threshold=8.110000133514404
node_31: feature_name=ENSG00000100297	feature_id[28].value <= threshold=111.95999908447266
node_32: feature_name=ENSG00000150991	feature_id[6].value > threshold=998.7099914550781
node_48: feature_name=ENSG00000205352	feature_id[88].value > threshold=73.43000030517578
Class: S	
Rules_7	passed counts:15
node_0: feature_name=ENSG00000170312	feature_id[0].value > threshold=120.56500244140625
node_30: feature_name=ENSG00000140451	feature_id[3].value > threshold=8.110000133514404
node_88: feature_name=ENSG00000105173	feature_id[8].value > threshold=98.07999801635742
node_140: feature_name=ENSG00000173207	feature_id[27].value > threshold=529.4750061035156
node_146: feature_name=ENSG00000170540	feature_id[57].value > threshold=46.05500030517578
node_148: feature_name=ENSG00000087586	feature_id[47].value <= threshold=302.0350036621094
Class: G2M	
Rules_8	passed counts:13
node_0: feature_name=ENSG00000170312	feature_id[0].value <= threshold=120.56500244140625
node_1: feature_name=ENSG00000197061	feature_id[1].value > threshold=794.760009765625

node_25: feature_name=ENSG00000128340	feature_id[85].value <= threshold=131.6999969482422
node_26: feature_name=ENSG00000167978	feature_id[48].value <= threshold=178.33999633789062
Class: S	
Rules_9	passed counts:10
node_0: feature_name=ENSG00000170312	feature_id[0].value > threshold=120.56500244140625
node_30: feature_name=ENSG00000140451	feature_id[3].value > threshold=8.110000133514404
node_88: feature_name=ENSG00000105173	feature_id[8].value <= threshold=98.07999801635742
node_89: feature_name=ENSG00000170312	feature_id[0].value > threshold=160.83999633789062
node_95: feature_name=ENSG00000167978	feature_id[48].value > threshold=37.79999923706055
node_101: feature_name=ENSG00000130429	feature_id[93].value <= threshold=323.2150115966797
node_102: feature_name=ENSG00000117724	feature_id[61].value <= threshold=42.60000038146973
node_103: feature_name=ENSG00000101003	feature_id[65].value > threshold=18.390000343322754
node_115: feature_name=ENSG00000171421	feature_id[70].value <= threshold=163.31999969482422
Class: S	
Rules_10	passed counts:10
node_0: feature_name=ENSG00000170312	feature_id[0].value > threshold=120.56500244140625
node_30: feature_name=ENSG00000140451	feature_id[3].value <= threshold=8.110000133514404
node_31: feature_name=ENSG00000100297	feature_id[28].value > threshold=111.95999908447266
node_51: feature_name=ENSG00000197061	feature_id[1].value > threshold=468.4400177001953
node_55: feature_name=ENSG00000012048	feature_id[12].value <= threshold=23.5600004196167
node_56: feature_name=ENSG00000167978	feature_id[48].value > threshold=66.39500045776367
node_64: feature_name=ENSG00000189057	feature_id[7].value <= threshold=166.1999969482422
Class: G2M	

Rules_11	passed counts:10
node_0: feature_name=ENSG00000170312	feature_id[0].value > threshold=120.56500244140625
node_30: feature_name=ENSG00000140451	feature_id[3].value <= threshold=8.110000133514404
node_31: feature_name=ENSG00000100297	feature_id[28].value > threshold=111.95999908447266
node_51: feature_name=ENSG00000197061	feature_id[1].value > threshold=468.4400177001953
node_55: feature_name=ENSG00000012048	feature_id[12].value <= threshold=23.5600004196167
node_56: feature_name=ENSG00000167978	feature_id[48].value <= threshold=66.39500045776367
node_57: feature_name=ENSG00000007968	feature_id[68].value <= threshold=11.649999618530273
node_58: feature_name=ENSG00000124172	feature_id[95].value <= threshold=285.64500427246094
Class: S	
Rules_12	passed counts:8
node_0: feature_name=ENSG00000170312	feature_id[0].value > threshold=120.56500244140625
node_30: feature_name=ENSG00000140451	feature_id[3].value <= threshold=8.110000133514404
node_31: feature_name=ENSG00000100297	feature_id[28].value <= threshold=111.95999908447266
node_32: feature_name=ENSG00000150991	feature_id[6].value <= threshold=998.7099914550781
node_33: feature_name=ENSG00000175305	feature_id[35].value > threshold=14.080000400543213
node_43: feature_name=ENSG00000124172	feature_id[95].value > threshold=191.47000122070312
Class: S	
Rules_13	passed counts:8
node_0: feature_name=ENSG00000170312	feature_id[0].value <= threshold=120.56500244140625
node_1: feature_name=ENSG00000197061	feature_id[1].value <= threshold=794.760009765625
node_2: feature_name=ENSG00000145425	feature_id[46].value <= threshold=4353.8848876953125

node_3: feature_name=ENSG00000127948	feature_id[39].value <= threshold=4.385000109672546
node_4: feature_name=ENSG00000012048	feature_id[12].value <= threshold=37.64999961853027
node_5: feature_name=ENSG00000124172	feature_id[95].value <= threshold=544.1550140380859
Class: G1	
Rules_14	passed counts:7
node_0: feature_name=ENSG00000170312	feature_id[0].value > threshold=120.56500244140625
node_30: feature_name=ENSG00000140451	feature_id[3].value > threshold=8.110000133514404
node_88: feature_name=ENSG00000105173	feature_id[8].value <= threshold=98.07999801635742
node_89: feature_name=ENSG00000170312	feature_id[0].value > threshold=160.83999633789062
node_95: feature_name=ENSG00000167978	feature_id[48].value > threshold=37.79999923706055
node_101: feature_name=ENSG00000130429	feature_id[93].value <= threshold=323.2150115966797
node_102: feature_name=ENSG00000117724	feature_id[61].value > threshold=42.60000038146973
node_118: feature_name=ENSG00000276368	feature_id[29].value <= threshold=333.0249938964844
node_119: feature_name=ENSG00000122952	feature_id[54].value <= threshold=716.2649841308594
node_120: feature_name=ENSG00000092853	feature_id[75].value <= threshold=40.64500045776367
node_121: feature_name=ENSG00000177426	feature_id[37].value <= threshold=32.579999923706055
node_122: feature_name=ENSG00000160957	feature_id[77].value <= threshold=85.9000015258789
Class: G2M	
Rules_15	passed counts:7
node_0: feature_name=ENSG00000170312	feature_id[0].value > threshold=120.56500244140625
node_30: feature_name=ENSG00000140451	feature_id[3].value <= threshold=8.110000133514404
node_31: feature_name=ENSG00000100297	feature_id[28].value > threshold=111.95999908447266
node_51: feature_name=ENSG00000197061	feature_id[1].value <= threshold=468.4400177001953

node_52: feature_name=ENSG00000176890	feature_id[80].value <= threshold=1332.7100219726562
Class: G1	
Rules_16	passed counts:5
node_0: feature_name=ENSG00000170312	feature_id[0].value > threshold=120.56500244140625
node_30: feature_name=ENSG00000140451	feature_id[3].value > threshold=8.110000133514404
node_88: feature_name=ENSG00000105173	feature_id[8].value <= threshold=98.07999801635742
node_89: feature_name=ENSG00000170312	feature_id[0].value > threshold=160.83999633789062
node_95: feature_name=ENSG00000167978	feature_id[48].value > threshold=37.79999923706055
node_101: feature_name=ENSG00000130429	feature_id[93].value > threshold=323.2150115966797
node_137: feature_name=ENSG00000118680	feature_id[16].value <= threshold=2694.409912109375
Class: S	
Rules_17	passed counts:5
node_0: feature_name=ENSG00000170312	feature_id[0].value > threshold=120.56500244140625
node_30: feature_name=ENSG00000140451	feature_id[3].value > threshold=8.110000133514404
node_88: feature_name=ENSG00000105173	feature_id[8].value <= threshold=98.07999801635742
node_89: feature_name=ENSG00000170312	feature_id[0].value > threshold=160.83999633789062
node_95: feature_name=ENSG00000167978	feature_id[48].value <= threshold=37.79999923706055
node_96: feature_name=ENSG00000163918	feature_id[100].value > threshold=34.13000011444092
Class: S	
Rules_18	passed counts:5
node_0: feature_name=ENSG00000170312	feature_id[0].value > threshold=120.56500244140625
node_30: feature_name=ENSG00000140451	feature_id[3].value > threshold=8.110000133514404

node_88: feature_name=ENSG00000105173	feature_id[8].value <= threshold=98.07999801635742
node_89: feature_name=ENSG00000170312	feature_id[0].value <= threshold=160.83999633789062
node_90: feature_name=ENSG00000164924	feature_id[58].value > threshold=637.7749938964844
Class: G1	
Rules_19	passed counts:4
node_0: feature_name=ENSG00000170312	feature_id[0].value > threshold=120.56500244140625
node_30: feature_name=ENSG00000140451	feature_id[3].value <= threshold=8.110000133514404
node_31: feature_name=ENSG00000100297	feature_id[28].value <= threshold=111.95999908447266
node_32: feature_name=ENSG00000150991	feature_id[6].value <= threshold=998.7099914550781
node_33: feature_name=ENSG00000175305	feature_id[35].value > threshold=14.080000400543213
node_43: feature_name=ENSG00000124172	feature_id[95].value <= threshold=191.47000122070312
node_44: feature_name=ENSG00000101447	feature_id[10].value > threshold=25.724998950958252
Class: G2M	
Rules_20	passed counts:4
node_0: feature_name=ENSG00000170312	feature_id[0].value <= threshold=120.56500244140625
node_1: feature_name=ENSG00000197061	feature_id[1].value <= threshold=794.760009765625
node_2: feature_name=ENSG00000145425	feature_id[46].value <= threshold=4353.8848876953125
node_3: feature_name=ENSG00000127948	feature_id[39].value <= threshold=4.385000109672546
node_4: feature_name=ENSG00000012048	feature_id[12].value > threshold=37.64999961853027
Class: S	
Rules_21	passed counts:3
node_0: feature_name=ENSG00000170312	feature_id[0].value > threshold=120.56500244140625

node_30: feature_name=ENSG00000140451	feature_id[3].value > threshold=8.110000133514404
node_88: feature_name=ENSG00000105173	feature_id[8].value > threshold=98.07999801635742
node_140: feature_name=ENSG00000173207	feature_id[27].value <= threshold=529.4750061035156
node_141: feature_name=ENSG00000185361	feature_id[97].value > threshold=7.180000066757202
Class: G2M	
Rules_22	passed counts:3
node_0: feature_name=ENSG00000170312	feature_id[0].value > threshold=120.56500244140625
node_30: feature_name=ENSG00000140451	feature_id[3].value > threshold=8.110000133514404
node_88: feature_name=ENSG00000105173	feature_id[8].value <= threshold=98.07999801635742
node_89: feature_name=ENSG00000170312	feature_id[0].value > threshold=160.83999633789062
node_95: feature_name=ENSG00000167978	feature_id[48].value > threshold=37.79999923706055
node_101: feature_name=ENSG00000130429	feature_id[93].value > threshold=323.2150115966797
node_137: feature_name=ENSG00000118680	feature_id[16].value > threshold=2694.409912109375
Class: G2M	
Rules_23	passed counts:3
node_0: feature_name=ENSG00000170312	feature_id[0].value > threshold=120.56500244140625
node_30: feature_name=ENSG00000140451	feature_id[3].value > threshold=8.110000133514404
node_88: feature_name=ENSG00000105173	feature_id[8].value <= threshold=98.07999801635742
node_89: feature_name=ENSG00000170312	feature_id[0].value > threshold=160.83999633789062
node_95: feature_name=ENSG00000167978	feature_id[48].value > threshold=37.79999923706055
node_101: feature_name=ENSG00000130429	feature_id[93].value <= threshold=323.2150115966797
node_102: feature_name=ENSG00000117724	feature_id[61].value > threshold=42.60000038146973
node_118: feature_name=ENSG00000276368	feature_id[29].value <= threshold=333.0249938964844

node_119: feature_name=ENSG00000122952	feature_id[54].value <= threshold=716.2649841308594
node_120: feature_name=ENSG00000092853	feature_id[75].value <= threshold=40.64500045776367
node_121: feature_name=ENSG00000177426	feature_id[37].value > threshold=32.579999923706055
node_125: feature_name=ENSG00000178913	feature_id[76].value <= threshold=114.54999923706055
node_126: feature_name=ENSG00000146670	feature_id[66].value <= threshold=33.939998626708984
node_127: feature_name=ENSG00000130429	feature_id[93].value > threshold=175.65499877929688
Class: G2M	
Rules_24	passed counts:3
node_0: feature_name=ENSG00000170312	feature_id[0].value > threshold=120.56500244140625
node_30: feature_name=ENSG00000140451	feature_id[3].value > threshold=8.110000133514404
node_88: feature_name=ENSG00000105173	feature_id[8].value <= threshold=98.07999801635742
node_89: feature_name=ENSG00000170312	feature_id[0].value > threshold=160.83999633789062
node_95: feature_name=ENSG00000167978	feature_id[48].value > threshold=37.79999923706055
node_101: feature_name=ENSG00000130429	feature_id[93].value <= threshold=323.2150115966797
node_102: feature_name=ENSG00000117724	feature_id[61].value > threshold=42.60000038146973
node_118: feature_name=ENSG00000276368	feature_id[29].value <= threshold=333.0249938964844
node_119: feature_name=ENSG00000122952	feature_id[54].value <= threshold=716.2649841308594
node_120: feature_name=ENSG00000092853	feature_id[75].value <= threshold=40.64500045776367
node_121: feature_name=ENSG00000177426	feature_id[37].value <= threshold=32.579999923706055
node_122: feature_name=ENSG00000160957	feature_id[77].value > threshold=85.9000015258789
Class: S	
Rules_25	passed counts:3
node_0: feature_name=ENSG00000170312	feature_id[0].value > threshold=120.56500244140625

node_30: feature_name=ENSG00000140451	feature_id[3].value > threshold=8.110000133514404
node_88: feature_name=ENSG00000105173	feature_id[8].value <= threshold=98.07999801635742
node_89: feature_name=ENSG00000170312	feature_id[0].value > threshold=160.83999633789062
node_95: feature_name=ENSG00000167978	feature_id[48].value > threshold=37.79999923706055
node_101: feature_name=ENSG00000130429	feature_id[93].value <= threshold=323.2150115966797
node_102: feature_name=ENSG00000117724	feature_id[61].value <= threshold=42.60000038146973
node_103: feature_name=ENSG00000101003	feature_id[65].value > threshold=18.390000343322754
node_115: feature_name=ENSG00000171421	feature_id[70].value > threshold=163.31999969482422
Class: G2M	
Rules_26	passed counts:3
node_0: feature_name=ENSG00000170312	feature_id[0].value > threshold=120.56500244140625
node_30: feature_name=ENSG00000140451	feature_id[3].value > threshold=8.110000133514404
node_88: feature_name=ENSG00000105173	feature_id[8].value <= threshold=98.07999801635742
node_89: feature_name=ENSG00000170312	feature_id[0].value <= threshold=160.83999633789062
node_90: feature_name=ENSG00000164924	feature_id[58].value <= threshold=637.7749938964844
node_91: feature_name=ENSG00000145425	feature_id[46].value > threshold=1300.0050048828125
Class: S	
Rules_27	passed counts:3
node_0: feature_name=ENSG00000170312	feature_id[0].value > threshold=120.56500244140625
node_30: feature_name=ENSG00000140451	feature_id[3].value <= threshold=8.110000133514404
node_31: feature_name=ENSG00000100297	feature_id[28].value > threshold=111.95999908447266
node_51: feature_name=ENSG00000197061	feature_id[1].value > threshold=468.4400177001953
node_55: feature_name=ENSG00000012048	feature_id[12].value > threshold=23.5600004196167

node_67: feature_name=ENSG00000127564	feature_id[86].value > threshold=22.22499942779541
node_71: feature_name=ENSG00000167601	feature_id[30].value <= threshold=391.2899932861328
node_72: feature_name=ENSG00000229089	feature_id[5].value <= threshold=106.125
node_73: feature_name=ENSG00000117399	feature_id[21].value <= threshold=510.64500427246094
node_74: feature_name=ENSG00000185361	feature_id[97].value <= threshold=12.335000038146973
node_75: feature_name=ENSG00000146670	feature_id[66].value > threshold=38.46000099182129
node_79: feature_name=ENSG00000173207	feature_id[27].value > threshold=653.6899719238281
node_81: feature_name=ENSG00000170540	feature_id[57].value > threshold=37.79999923706055
Class: S	
Rules_28	passed counts:3
node_0: feature_name=ENSG00000170312	feature_id[0].value > threshold=120.56500244140625
node_30: feature_name=ENSG00000140451	feature_id[3].value <= threshold=8.110000133514404
node_31: feature_name=ENSG00000100297	feature_id[28].value > threshold=111.95999908447266
node_51: feature_name=ENSG00000197061	feature_id[1].value > threshold=468.4400177001953
node_55: feature_name=ENSG00000012048	feature_id[12].value <= threshold=23.5600004196167
node_56: feature_name=ENSG00000167978	feature_id[48].value > threshold=66.39500045776367
node_64: feature_name=ENSG00000189057	feature_id[7].value > threshold=166.1999969482422
Class: S	
Rules_29	passed counts:3
node_0: feature_name=ENSG00000170312	feature_id[0].value > threshold=120.56500244140625
node_30: feature_name=ENSG00000140451	feature_id[3].value <= threshold=8.110000133514404
node_31: feature_name=ENSG00000100297	feature_id[28].value <= threshold=111.95999908447266
node_32: feature_name=ENSG00000150991	feature_id[6].value <= threshold=998.7099914550781

node_33: feature_name=ENSG00000175305	feature_id[35].value <= threshold=14.080000400543213
node_34: feature_name=ENSG00000186767	feature_id[89].value > threshold=22.6850004196167
node_40: feature_name=ENSG00000223564	feature_id[26].value > threshold=12.195000171661377
Class: S	
Rules_30	passed counts:3
node_0: feature_name=ENSG00000170312	feature_id[0].value <= threshold=120.56500244140625
node_1: feature_name=ENSG00000197061	feature_id[1].value > threshold=794.760009765625
node_25: feature_name=ENSG00000128340	feature_id[85].value > threshold=131.6999969482422
Class: G1	
Rules_31	passed counts:3
node_0: feature_name=ENSG00000170312	feature_id[0].value <= threshold=120.56500244140625
node_1: feature_name=ENSG00000197061	feature_id[1].value <= threshold=794.760009765625
node_2: feature_name=ENSG00000145425	feature_id[46].value > threshold=4353.8848876953125
Class: G2M	
Rules_32	passed counts:2
node_0: feature_name=ENSG00000170312	feature_id[0].value > threshold=120.56500244140625
node_30: feature_name=ENSG00000140451	feature_id[3].value > threshold=8.110000133514404
node_88: feature_name=ENSG00000105173	feature_id[8].value > threshold=98.07999801635742
node_140: feature_name=ENSG00000173207	feature_id[27].value > threshold=529.4750061035156
node_146: feature_name=ENSG00000170540	feature_id[57].value <= threshold=46.05500030517578
Class: S	

Rules_33	passed counts:2
node_0: feature_name=ENSG00000170312	feature_id[0].value > threshold=120.56500244140625
node_30: feature_name=ENSG00000140451	feature_id[3].value > threshold=8.110000133514404
node_88: feature_name=ENSG00000105173	feature_id[8].value <= threshold=98.07999801635742
node_89: feature_name=ENSG00000170312	feature_id[0].value > threshold=160.83999633789062
node_95: feature_name=ENSG00000167978	feature_id[48].value > threshold=37.79999923706055
node_101: feature_name=ENSG00000130429	feature_id[93].value <= threshold=323.2150115966797
node_102: feature_name=ENSG00000117724	feature_id[61].value > threshold=42.60000038146973
node_118: feature_name=ENSG00000276368	feature_id[29].value <= threshold=333.0249938964844
node_119: feature_name=ENSG00000122952	feature_id[54].value <= threshold=716.2649841308594
node_120: feature_name=ENSG00000092853	feature_id[75].value <= threshold=40.64500045776367
node_121: feature_name=ENSG00000177426	feature_id[37].value > threshold=32.579999923706055
node_125: feature_name=ENSG00000178913	feature_id[76].value > threshold=114.54999923706055
node_131: feature_name=ENSG00000117724	feature_id[61].value > threshold=76.71999740600586
Class: G2M	
Rules_34	passed counts:2
node_0: feature_name=ENSG00000170312	feature_id[0].value > threshold=120.56500244140625
node_30: feature_name=ENSG00000140451	feature_id[3].value > threshold=8.110000133514404
node_88: feature_name=ENSG00000105173	feature_id[8].value <= threshold=98.07999801635742
node_89: feature_name=ENSG00000170312	feature_id[0].value > threshold=160.83999633789062
node_95: feature_name=ENSG00000167978	feature_id[48].value > threshold=37.79999923706055
node_101: feature_name=ENSG00000130429	feature_id[93].value <= threshold=323.2150115966797
node_102: feature_name=ENSG00000117724	feature_id[61].value <= threshold=42.60000038146973
node_103: feature_name=ENSG00000101003	feature_id[65].value <= threshold=18.390000343322754

node_104: feature_name=ENSG00000112029	feature_id[18].value > threshold=93.15499877929688
Class: S	
Rules_35	passed counts:2
node_0: feature_name=ENSG00000170312	feature_id[0].value > threshold=120.56500244140625
node_30: feature_name=ENSG00000140451	feature_id[3].value > threshold=8.110000133514404
node_88: feature_name=ENSG00000105173	feature_id[8].value <= threshold=98.07999801635742
node_89: feature_name=ENSG00000170312	feature_id[0].value > threshold=160.83999633789062
node_95: feature_name=ENSG00000167978	feature_id[48].value > threshold=37.79999923706055
node_101: feature_name=ENSG00000130429	feature_id[93].value <= threshold=323.2150115966797
node_102: feature_name=ENSG00000117724	feature_id[61].value <= threshold=42.60000038146973
node_103: feature_name=ENSG00000101003	feature_id[65].value <= threshold=18.390000343322754
node_104: feature_name=ENSG00000112029	feature_id[18].value <= threshold=93.15499877929688
node_105: feature_name=ENSG00000114346	feature_id[74].value > threshold=8.815000057220459
node_107: feature_name=ENSG00000149548	feature_id[83].value > threshold=6.200000047683716
node_111: feature_name=ENSG00000144354	feature_id[59].value <= threshold=6.335000157356262
Class: S	
Rules_36	passed counts:2
node_0: feature_name=ENSG00000170312	feature_id[0].value > threshold=120.56500244140625
node_30: feature_name=ENSG00000140451	feature_id[3].value > threshold=8.110000133514404
node_88: feature_name=ENSG00000105173	feature_id[8].value <= threshold=98.07999801635742
node_89: feature_name=ENSG00000170312	feature_id[0].value > threshold=160.83999633789062
node_95: feature_name=ENSG00000167978	feature_id[48].value > threshold=37.79999923706055
node_101: feature_name=ENSG00000130429	feature_id[93].value <= threshold=323.2150115966797

node_102: feature_name=ENSG00000117724	feature_id[61].value <= threshold=42.60000038146973
node_103: feature_name=ENSG00000101003	feature_id[65].value <= threshold=18.390000343322754
node_104: feature_name=ENSG00000112029	feature_id[18].value <= threshold=93.15499877929688
node_105: feature_name=ENSG00000114346	feature_id[74].value <= threshold=8.815000057220459
Class: S	
Rules_37	passed counts:2
node_0: feature_name=ENSG00000170312	feature_id[0].value > threshold=120.56500244140625
node_30: feature_name=ENSG00000140451	feature_id[3].value <= threshold=8.110000133514404
node_31: feature_name=ENSG00000100297	feature_id[28].value > threshold=111.95999908447266
node_51: feature_name=ENSG00000197061	feature_id[1].value > threshold=468.4400177001953
node_55: feature_name=ENSG00000012048	feature_id[12].value > threshold=23.5600004196167
node_67: feature_name=ENSG00000127564	feature_id[86].value > threshold=22.22499942779541
node_71: feature_name=ENSG00000167601	feature_id[30].value > threshold=391.2899932861328
Class: G2M	
Rules_38	passed counts:2
node_0: feature_name=ENSG00000170312	feature_id[0].value > threshold=120.56500244140625
node_30: feature_name=ENSG00000140451	feature_id[3].value <= threshold=8.110000133514404
node_31: feature_name=ENSG00000100297	feature_id[28].value > threshold=111.95999908447266
node_51: feature_name=ENSG00000197061	feature_id[1].value > threshold=468.4400177001953
node_55: feature_name=ENSG00000012048	feature_id[12].value > threshold=23.5600004196167
node_67: feature_name=ENSG00000127564	feature_id[86].value > threshold=22.22499942779541
node_71: feature_name=ENSG00000167601	feature_id[30].value <= threshold=391.2899932861328
node_72: feature_name=ENSG00000229089	feature_id[5].value > threshold=106.125

Class: G2M	
Rules_39	passed counts:2
node_0: feature_name=ENSG00000170312	feature_id[0].value > threshold=120.56500244140625
node_30: feature_name=ENSG00000140451	feature_id[3].value <= threshold=8.110000133514404
node_31: feature_name=ENSG00000100297	feature_id[28].value > threshold=111.95999908447266
node_51: feature_name=ENSG00000197061	feature_id[1].value > threshold=468.4400177001953
node_55: feature_name=ENSG00000012048	feature_id[12].value > threshold=23.5600004196167
node_67: feature_name=ENSG00000127564	feature_id[86].value <= threshold=22.22499942779541
node_68: feature_name=ENSG00000186767	feature_id[89].value <= threshold=19.394999504089355
Class: G2M	
Rules_40	passed counts:2
node_0: feature_name=ENSG00000170312	feature_id[0].value > threshold=120.56500244140625
node_30: feature_name=ENSG00000140451	feature_id[3].value <= threshold=8.110000133514404
node_31: feature_name=ENSG00000100297	feature_id[28].value > threshold=111.95999908447266
node_51: feature_name=ENSG00000197061	feature_id[1].value > threshold=468.4400177001953
node_55: feature_name=ENSG00000012048	feature_id[12].value <= threshold=23.5600004196167
node_56: feature_name=ENSG00000167978	feature_id[48].value <= threshold=66.39500045776367
node_57: feature_name=ENSG00000007968	feature_id[68].value > threshold=11.649999618530273
node_61: feature_name=ENSG00000171848	feature_id[45].value <= threshold=863.9649963378906
Class: G1	
Rules_41	passed counts:2
node_0: feature_name=ENSG00000170312	feature_id[0].value > threshold=120.56500244140625

node_30: feature_name=ENSG00000140451	feature_id[3].value <= threshold=8.110000133514404
node_31: feature_name=ENSG00000100297	feature_id[28].value > threshold=111.95999908447266
node_51: feature_name=ENSG00000197061	feature_id[1].value <= threshold=468.4400177001953
node_52: feature_name=ENSG00000176890	feature_id[80].value > threshold=1332.7100219726562
Class: S	
Rules_42	passed counts:2
node_0: feature_name=ENSG00000170312	feature_id[0].value > threshold=120.56500244140625
node_30: feature_name=ENSG00000140451	feature_id[3].value <= threshold=8.110000133514404
node_31: feature_name=ENSG00000100297	feature_id[28].value <= threshold=111.95999908447266
node_32: feature_name=ENSG00000150991	feature_id[6].value > threshold=998.7099914550781
node_48: feature_name=ENSG00000205352	feature_id[88].value <= threshold=73.43000030517578
Class: G2M	
Rules_43	passed counts:2
node_0: feature_name=ENSG00000170312	feature_id[0].value > threshold=120.56500244140625
node_30: feature_name=ENSG00000140451	feature_id[3].value <= threshold=8.110000133514404
node_31: feature_name=ENSG00000100297	feature_id[28].value <= threshold=111.95999908447266
node_32: feature_name=ENSG00000150991	feature_id[6].value <= threshold=998.7099914550781
node_33: feature_name=ENSG00000175305	feature_id[35].value <= threshold=14.080000400543213
node_34: feature_name=ENSG00000186767	feature_id[89].value <= threshold=22.6850004196167
node_35: feature_name=ENSG00000185361	feature_id[97].value > threshold=16.954999923706055
Class: G1	
Rules_44	passed counts:2

node_0: feature_name=ENSG00000170312	feature_id[0].value <= threshold=120.56500244140625
node_1: feature_name=ENSG00000197061	feature_id[1].value <= threshold=794.760009765625
node_2: feature_name=ENSG00000145425	feature_id[46].value <= threshold=4353.8848876953125
node_3: feature_name=ENSG00000127948	feature_id[39].value > threshold=4.385000109672546
node_9: feature_name=ENSG00000150991	feature_id[6].value > threshold=72.85999965667725
node_11: feature_name=ENSG00000014138	feature_id[17].value > threshold=102.51499938964844
node_21: feature_name=ENSG00000170425	feature_id[22].value > threshold=54.829999923706055
Class: G1	
Rules_45	passed counts:2
node_0: feature_name=ENSG00000170312	feature_id[0].value <= threshold=120.56500244140625
node_1: feature_name=ENSG00000197061	feature_id[1].value <= threshold=794.760009765625
node_2: feature_name=ENSG00000145425	feature_id[46].value <= threshold=4353.8848876953125
node_3: feature_name=ENSG00000127948	feature_id[39].value > threshold=4.385000109672546
node_9: feature_name=ENSG00000150991	feature_id[6].value > threshold=72.85999965667725
node_11: feature_name=ENSG00000014138	feature_id[17].value > threshold=102.51499938964844
node_21: feature_name=ENSG00000170425	feature_id[22].value <= threshold=54.829999923706055
Class: S	
Rules_46	passed counts:1
node_0: feature_name=ENSG00000170312	feature_id[0].value > threshold=120.56500244140625
node_30: feature_name=ENSG00000140451	feature_id[3].value > threshold=8.110000133514404
node_88: feature_name=ENSG00000105173	feature_id[8].value > threshold=98.07999801635742
node_140: feature_name=ENSG00000173207	feature_id[27].value > threshold=529.4750061035156
node_146: feature_name=ENSG00000170540	feature_id[57].value > threshold=46.05500030517578

node_148: feature_name=ENSG00000087586	feature_id[47].value > threshold=302.0350036621094
Class: S	
Rules_47	passed counts:1
node_0: feature_name=ENSG00000170312	feature_id[0].value > threshold=120.56500244140625
node_30: feature_name=ENSG00000140451	feature_id[3].value > threshold=8.110000133514404
node_88: feature_name=ENSG00000105173	feature_id[8].value > threshold=98.07999801635742
node_140: feature_name=ENSG00000173207	feature_id[27].value <= threshold=529.4750061035156
node_141: feature_name=ENSG00000185361	feature_id[97].value <= threshold=7.180000066757202
node_142: feature_name=ENSG00000128340	feature_id[85].value <= threshold=16.95500087738037
Class: G2M	
Rules_48	passed counts:1
node_0: feature_name=ENSG00000170312	feature_id[0].value > threshold=120.56500244140625
node_30: feature_name=ENSG00000140451	feature_id[3].value > threshold=8.110000133514404
node_88: feature_name=ENSG00000105173	feature_id[8].value <= threshold=98.07999801635742
node_89: feature_name=ENSG00000170312	feature_id[0].value > threshold=160.83999633789062
node_95: feature_name=ENSG00000167978	feature_id[48].value > threshold=37.79999923706055
node_101: feature_name=ENSG00000130429	feature_id[93].value <= threshold=323.2150115966797
node_102: feature_name=ENSG00000117724	feature_id[61].value > threshold=42.60000038146973
node_118: feature_name=ENSG00000276368	feature_id[29].value > threshold=333.0249938964844
Class: S	
Rules_49	passed counts:1
node_0: feature_name=ENSG00000170312	feature_id[0].value > threshold=120.56500244140625

node_30: feature_name=ENSG00000140451	feature_id[3].value > threshold=8.110000133514404
node_88: feature_name=ENSG00000105173	feature_id[8].value <= threshold=98.07999801635742
node_89: feature_name=ENSG00000170312	feature_id[0].value > threshold=160.83999633789062
node_95: feature_name=ENSG00000167978	feature_id[48].value > threshold=37.79999923706055
node_101: feature_name=ENSG00000130429	feature_id[93].value <= threshold=323.2150115966797
node_102: feature_name=ENSG00000117724	feature_id[61].value > threshold=42.60000038146973
node_118: feature_name=ENSG00000276368	feature_id[29].value <= threshold=333.0249938964844
node_119: feature_name=ENSG00000122952	feature_id[54].value > threshold=716.2649841308594
Class: S	
Rules_50	passed counts:1
node_0: feature_name=ENSG00000170312	feature_id[0].value > threshold=120.56500244140625
node_30: feature_name=ENSG00000140451	feature_id[3].value > threshold=8.110000133514404
node_88: feature_name=ENSG00000105173	feature_id[8].value <= threshold=98.07999801635742
node_89: feature_name=ENSG00000170312	feature_id[0].value > threshold=160.83999633789062
node_95: feature_name=ENSG00000167978	feature_id[48].value > threshold=37.79999923706055
node_101: feature_name=ENSG00000130429	feature_id[93].value <= threshold=323.2150115966797
node_102: feature_name=ENSG00000117724	feature_id[61].value > threshold=42.60000038146973
node_118: feature_name=ENSG00000276368	feature_id[29].value <= threshold=333.0249938964844
node_119: feature_name=ENSG00000122952	feature_id[54].value <= threshold=716.2649841308594
node_120: feature_name=ENSG00000092853	feature_id[75].value > threshold=40.64500045776367
Class: S	
Rules_51	passed counts:1
node_0: feature_name=ENSG00000170312	feature_id[0].value > threshold=120.56500244140625

node_30: feature_name=ENSG00000140451	feature_id[3].value > threshold=8.110000133514404
node_88: feature_name=ENSG00000105173	feature_id[8].value <= threshold=98.07999801635742
node_89: feature_name=ENSG00000170312	feature_id[0].value > threshold=160.83999633789062
node_95: feature_name=ENSG00000167978	feature_id[48].value > threshold=37.79999923706055
node_101: feature_name=ENSG00000130429	feature_id[93].value <= threshold=323.2150115966797
node_102: feature_name=ENSG00000117724	feature_id[61].value > threshold=42.60000038146973
node_118: feature_name=ENSG00000276368	feature_id[29].value <= threshold=333.0249938964844
node_119: feature_name=ENSG00000122952	feature_id[54].value <= threshold=716.2649841308594
node_120: feature_name=ENSG00000092853	feature_id[75].value <= threshold=40.64500045776367
node_121: feature_name=ENSG00000177426	feature_id[37].value > threshold=32.579999923706055
node_125: feature_name=ENSG00000178913	feature_id[76].value > threshold=114.54999923706055
node_131: feature_name=ENSG00000117724	feature_id[61].value <= threshold=76.71999740600586
Class: S	
Rules_52	passed counts:1
node_0: feature_name=ENSG00000170312	feature_id[0].value > threshold=120.56500244140625
node_30: feature_name=ENSG00000140451	feature_id[3].value > threshold=8.110000133514404
node_88: feature_name=ENSG00000105173	feature_id[8].value <= threshold=98.07999801635742
node_89: feature_name=ENSG00000170312	feature_id[0].value > threshold=160.83999633789062
node_95: feature_name=ENSG00000167978	feature_id[48].value > threshold=37.79999923706055
node_101: feature_name=ENSG00000130429	feature_id[93].value <= threshold=323.2150115966797
node_102: feature_name=ENSG00000117724	feature_id[61].value > threshold=42.60000038146973
node_118: feature_name=ENSG00000276368	feature_id[29].value <= threshold=333.0249938964844
node_119: feature_name=ENSG00000122952	feature_id[54].value <= threshold=716.2649841308594
node_120: feature_name=ENSG00000092853	feature_id[75].value <= threshold=40.64500045776367

node_121: feature_name=ENSG00000177426	feature_id[37].value > threshold=32.579999923706055
node_125: feature_name=ENSG00000178913	feature_id[76].value <= threshold=114.54999923706055
node_126: feature_name=ENSG00000146670	feature_id[66].value <= threshold=33.939998626708984
node_127: feature_name=ENSG00000130429	feature_id[93].value <= threshold=175.65499877929688
Class: S	
Rules_53	passed counts:1
node_0: feature_name=ENSG00000170312	feature_id[0].value > threshold=120.56500244140625
node_30: feature_name=ENSG00000140451	feature_id[3].value > threshold=8.110000133514404
node_88: feature_name=ENSG00000105173	feature_id[8].value <= threshold=98.07999801635742
node_89: feature_name=ENSG00000170312	feature_id[0].value > threshold=160.83999633789062
node_95: feature_name=ENSG00000167978	feature_id[48].value > threshold=37.79999923706055
node_101: feature_name=ENSG00000130429	feature_id[93].value <= threshold=323.2150115966797
node_102: feature_name=ENSG00000117724	feature_id[61].value <= threshold=42.60000038146973
node_103: feature_name=ENSG00000101003	feature_id[65].value <= threshold=18.390000343322754
node_104: feature_name=ENSG00000112029	feature_id[18].value <= threshold=93.15499877929688
node_105: feature_name=ENSG00000114346	feature_id[74].value > threshold=8.815000057220459
node_107: feature_name=ENSG00000149548	feature_id[83].value > threshold=6.200000047683716
node_111: feature_name=ENSG00000144354	feature_id[59].value > threshold=6.335000157356262
Class: G2M	
Rules_54	passed counts:1
node_0: feature_name=ENSG00000170312	feature_id[0].value > threshold=120.56500244140625
node_30: feature_name=ENSG00000140451	feature_id[3].value > threshold=8.110000133514404
node_88: feature_name=ENSG00000105173	feature_id[8].value <= threshold=98.07999801635742

node_89: feature_name=ENSG00000170312	feature_id[0].value > threshold=160.83999633789062
node_95: feature_name=ENSG00000167978	feature_id[48].value > threshold=37.79999923706055
node_101: feature_name=ENSG00000130429	feature_id[93].value <= threshold=323.2150115966797
node_102: feature_name=ENSG00000117724	feature_id[61].value <= threshold=42.60000038146973
node_103: feature_name=ENSG00000101003	feature_id[65].value <= threshold=18.390000343322754
node_104: feature_name=ENSG00000112029	feature_id[18].value <= threshold=93.15499877929688
node_105: feature_name=ENSG00000114346	feature_id[74].value > threshold=8.815000057220459
node_107: feature_name=ENSG00000149548	feature_id[83].value <= threshold=6.200000047683716
node_108: feature_name=ENSG00000128340	feature_id[85].value <= threshold=5.1000001430511475
Class: S	
Rules_55	passed counts:1
node_0: feature_name=ENSG00000170312	feature_id[0].value > threshold=120.56500244140625
node_30: feature_name=ENSG00000140451	feature_id[3].value > threshold=8.110000133514404
node_88: feature_name=ENSG00000105173	feature_id[8].value <= threshold=98.07999801635742
node_89: feature_name=ENSG00000170312	feature_id[0].value > threshold=160.83999633789062
node_95: feature_name=ENSG00000167978	feature_id[48].value <= threshold=37.79999923706055
node_96: feature_name=ENSG00000163918	feature_id[100].value <= threshold=34.13000011444092
node_97: feature_name=ENSG00000158246	feature_id[63].value > threshold=4.670000076293945
Class: G2M	
Rules_56	passed counts:1
node_0: feature_name=ENSG00000170312	feature_id[0].value > threshold=120.56500244140625
node_30: feature_name=ENSG00000140451	feature_id[3].value > threshold=8.110000133514404
node_88: feature_name=ENSG00000105173	feature_id[8].value <= threshold=98.07999801635742

node_89: feature_name=ENSG00000170312	feature_id[0].value > threshold=160.83999633789062
node_95: feature_name=ENSG00000167978	feature_id[48].value <= threshold=37.79999923706055
node_96: feature_name=ENSG00000163918	feature_id[100].value <= threshold=34.13000011444092
node_97: feature_name=ENSG00000158246	feature_id[63].value <= threshold=4.670000076293945
Class: G1	
Rules_57	passed counts:1
node_0: feature_name=ENSG00000170312	feature_id[0].value > threshold=120.56500244140625
node_30: feature_name=ENSG00000140451	feature_id[3].value > threshold=8.110000133514404
node_88: feature_name=ENSG00000105173	feature_id[8].value <= threshold=98.07999801635742
node_89: feature_name=ENSG00000170312	feature_id[0].value <= threshold=160.83999633789062
node_90: feature_name=ENSG00000164924	feature_id[58].value <= threshold=637.7749938964844
node_91: feature_name=ENSG00000145425	feature_id[46].value <= threshold=1300.0050048828125
Class: G2M	
Rules_58	passed counts:1
node_0: feature_name=ENSG00000170312	feature_id[0].value > threshold=120.56500244140625
node_30: feature_name=ENSG00000140451	feature_id[3].value <= threshold=8.110000133514404
node_31: feature_name=ENSG00000100297	feature_id[28].value > threshold=111.95999908447266
node_51: feature_name=ENSG00000197061	feature_id[1].value > threshold=468.4400177001953
node_55: feature_name=ENSG00000012048	feature_id[12].value > threshold=23.5600004196167
node_67: feature_name=ENSG00000127564	feature_id[86].value > threshold=22.22499942779541
node_71: feature_name=ENSG00000167601	feature_id[30].value <= threshold=391.2899932861328
node_72: feature_name=ENSG00000229089	feature_id[5].value <= threshold=106.125
node_73: feature_name=ENSG00000117399	feature_id[21].value > threshold=510.64500427246094

Class: G1	
Rules_59	passed counts:1
node_0: feature_name=ENSG00000170312	feature_id[0].value > threshold=120.56500244140625
node_30: feature_name=ENSG00000140451	feature_id[3].value <= threshold=8.110000133514404
node_31: feature_name=ENSG00000100297	feature_id[28].value > threshold=111.95999908447266
node_51: feature_name=ENSG00000197061	feature_id[1].value > threshold=468.4400177001953
node_55: feature_name=ENSG00000012048	feature_id[12].value > threshold=23.5600004196167
node_67: feature_name=ENSG00000127564	feature_id[86].value > threshold=22.22499942779541
node_71: feature_name=ENSG00000167601	feature_id[30].value <= threshold=391.2899932861328
node_72: feature_name=ENSG00000229089	feature_id[5].value <= threshold=106.125
node_73: feature_name=ENSG00000117399	feature_id[21].value <= threshold=510.64500427246094
node_74: feature_name=ENSG00000185361	feature_id[97].value > threshold=12.335000038146973
Class: G2M	
Rules_60	passed counts:1
node_0: feature_name=ENSG00000170312	feature_id[0].value > threshold=120.56500244140625
node_30: feature_name=ENSG00000140451	feature_id[3].value <= threshold=8.110000133514404
node_31: feature_name=ENSG00000100297	feature_id[28].value > threshold=111.95999908447266
node_51: feature_name=ENSG00000197061	feature_id[1].value > threshold=468.4400177001953
node_55: feature_name=ENSG00000012048	feature_id[12].value > threshold=23.5600004196167
node_67: feature_name=ENSG00000127564	feature_id[86].value > threshold=22.22499942779541
node_71: feature_name=ENSG00000167601	feature_id[30].value <= threshold=391.2899932861328
node_72: feature_name=ENSG00000229089	feature_id[5].value <= threshold=106.125
node_73: feature_name=ENSG00000117399	feature_id[21].value <= threshold=510.64500427246094

node_74: feature_name=ENSG00000185361	feature_id[97].value <= threshold=12.335000038146973
node_75: feature_name=ENSG00000146670	feature_id[66].value > threshold=38.46000099182129
node_79: feature_name=ENSG00000173207	feature_id[27].value > threshold=653.6899719238281
node_81: feature_name=ENSG00000170540	feature_id[57].value <= threshold=37.79999923706055
Class: G2M	
Rules_61	passed counts:1
node_0: feature_name=ENSG00000170312	feature_id[0].value > threshold=120.56500244140625
node_30: feature_name=ENSG00000140451	feature_id[3].value <= threshold=8.110000133514404
node_31: feature_name=ENSG00000100297	feature_id[28].value > threshold=111.95999908447266
node_51: feature_name=ENSG00000197061	feature_id[1].value > threshold=468.4400177001953
node_55: feature_name=ENSG00000012048	feature_id[12].value > threshold=23.5600004196167
node_67: feature_name=ENSG00000127564	feature_id[86].value > threshold=22.22499942779541
node_71: feature_name=ENSG00000167601	feature_id[30].value <= threshold=391.2899932861328
node_72: feature_name=ENSG00000229089	feature_id[5].value <= threshold=106.125
node_73: feature_name=ENSG00000117399	feature_id[21].value <= threshold=510.64500427246094
node_74: feature_name=ENSG00000185361	feature_id[97].value <= threshold=12.335000038146973
node_75: feature_name=ENSG00000146670	feature_id[66].value <= threshold=38.46000099182129
node_76: feature_name=ENSG00000259848	feature_id[13].value > threshold=17.149999618530273
Class: G2M	
Rules_62	passed counts:1
node_0: feature_name=ENSG00000170312	feature_id[0].value > threshold=120.56500244140625
node_30: feature_name=ENSG00000140451	feature_id[3].value <= threshold=8.110000133514404
node_31: feature_name=ENSG00000100297	feature_id[28].value > threshold=111.95999908447266

node_51: feature_name=ENSG00000197061	feature_id[1].value > threshold=468.4400177001953
node_55: feature_name=ENSG00000012048	feature_id[12].value > threshold=23.5600004196167
node_67: feature_name=ENSG00000127564	feature_id[86].value > threshold=22.22499942779541
node_71: feature_name=ENSG00000167601	feature_id[30].value <= threshold=391.2899932861328
node_72: feature_name=ENSG00000229089	feature_id[5].value <= threshold=106.125
node_73: feature_name=ENSG00000117399	feature_id[21].value <= threshold=510.64500427246094
node_74: feature_name=ENSG00000185361	feature_id[97].value <= threshold=12.335000038146973
node_75: feature_name=ENSG00000146670	feature_id[66].value <= threshold=38.46000099182129
node_76: feature_name=ENSG00000259848	feature_id[13].value <= threshold=17.149999618530273
Class: S	
Rules_63	passed counts:1
node_0: feature_name=ENSG00000170312	feature_id[0].value > threshold=120.56500244140625
node_30: feature_name=ENSG00000140451	feature_id[3].value <= threshold=8.110000133514404
node_31: feature_name=ENSG00000100297	feature_id[28].value > threshold=111.95999908447266
node_51: feature_name=ENSG00000197061	feature_id[1].value > threshold=468.4400177001953
node_55: feature_name=ENSG00000012048	feature_id[12].value > threshold=23.5600004196167
node_67: feature_name=ENSG00000127564	feature_id[86].value <= threshold=22.22499942779541
node_68: feature_name=ENSG00000186767	feature_id[89].value > threshold=19.394999504089355
Class: G1	
Rules_64	passed counts:1
node_0: feature_name=ENSG00000170312	feature_id[0].value > threshold=120.56500244140625
node_30: feature_name=ENSG00000140451	feature_id[3].value <= threshold=8.110000133514404
node_31: feature_name=ENSG00000100297	feature_id[28].value > threshold=111.95999908447266

node_51: feature_name=ENSG00000197061	feature_id[1].value > threshold=468.4400177001953
node_55: feature_name=ENSG00000012048	feature_id[12].value <= threshold=23.5600004196167
node_56: feature_name=ENSG00000167978	feature_id[48].value <= threshold=66.39500045776367
node_57: feature_name=ENSG00000007968	feature_id[68].value > threshold=11.649999618530273
node_61: feature_name=ENSG00000171848	feature_id[45].value > threshold=863.9649963378906
Class: G2M	
Rules_65	passed counts:1
node_0: feature_name=ENSG00000170312	feature_id[0].value > threshold=120.56500244140625
node_30: feature_name=ENSG00000140451	feature_id[3].value <= threshold=8.110000133514404
node_31: feature_name=ENSG00000100297	feature_id[28].value > threshold=111.95999908447266
node_51: feature_name=ENSG00000197061	feature_id[1].value > threshold=468.4400177001953
node_55: feature_name=ENSG00000012048	feature_id[12].value <= threshold=23.5600004196167
node_56: feature_name=ENSG00000167978	feature_id[48].value <= threshold=66.39500045776367
node_57: feature_name=ENSG00000007968	feature_id[68].value <= threshold=11.649999618530273
node_58: feature_name=ENSG00000124172	feature_id[95].value > threshold=285.64500427246094
Class: G2M	
Rules_66	passed counts:1
node_0: feature_name=ENSG00000170312	feature_id[0].value > threshold=120.56500244140625
node_30: feature_name=ENSG00000140451	feature_id[3].value <= threshold=8.110000133514404
node_31: feature_name=ENSG00000100297	feature_id[28].value <= threshold=111.95999908447266
node_32: feature_name=ENSG00000150991	feature_id[6].value <= threshold=998.7099914550781
node_33: feature_name=ENSG00000175305	feature_id[35].value > threshold=14.080000400543213
node_43: feature_name=ENSG00000124172	feature_id[95].value <= threshold=191.47000122070312

node_44: feature_name=ENSG00000101447	feature_id[10].value <= threshold=25.724998950958252
Class: S	
Rules_67	passed counts:1
node_0: feature_name=ENSG00000170312	feature_id[0].value > threshold=120.56500244140625
node_30: feature_name=ENSG00000140451	feature_id[3].value <= threshold=8.110000133514404
node_31: feature_name=ENSG00000100297	feature_id[28].value <= threshold=111.95999908447266
node_32: feature_name=ENSG00000150991	feature_id[6].value <= threshold=998.7099914550781
node_33: feature_name=ENSG00000175305	feature_id[35].value <= threshold=14.080000400543213
node_34: feature_name=ENSG00000186767	feature_id[89].value > threshold=22.6850004196167
node_40: feature_name=ENSG00000223564	feature_id[26].value <= threshold=12.195000171661377
Class: G2M	
Rules_68	passed counts:1
node_0: feature_name=ENSG00000170312	feature_id[0].value > threshold=120.56500244140625
node_30: feature_name=ENSG00000140451	feature_id[3].value <= threshold=8.110000133514404
node_31: feature_name=ENSG00000100297	feature_id[28].value <= threshold=111.95999908447266
node_32: feature_name=ENSG00000150991	feature_id[6].value <= threshold=998.7099914550781
node_33: feature_name=ENSG00000175305	feature_id[35].value <= threshold=14.080000400543213
node_34: feature_name=ENSG00000186767	feature_id[89].value <= threshold=22.6850004196167
node_35: feature_name=ENSG00000185361	feature_id[97].value <= threshold=16.954999923706055
node_36: feature_name=ENSG00000177426	feature_id[37].value > threshold=286.0399932861328
Class: S	
Rules_69	passed counts:1

node_0: feature_name=ENSG00000170312	feature_id[0].value <= threshold=120.56500244140625
node_1: feature_name=ENSG00000197061	feature_id[1].value > threshold=794.760009765625
node_25: feature_name=ENSG00000128340	feature_id[85].value <= threshold=131.6999969482422
node_26: feature_name=ENSG00000167978	feature_id[48].value > threshold=178.33999633789062
Class: G1	
Rules_70	passed counts:1
node_0: feature_name=ENSG00000170312	feature_id[0].value <= threshold=120.56500244140625
node_1: feature_name=ENSG00000197061	feature_id[1].value <= threshold=794.760009765625
node_2: feature_name=ENSG00000145425	feature_id[46].value <= threshold=4353.8848876953125
node_3: feature_name=ENSG00000127948	feature_id[39].value > threshold=4.385000109672546
node_9: feature_name=ENSG00000150991	feature_id[6].value > threshold=72.85999965667725
node_11: feature_name=ENSG00000014138	feature_id[17].value <= threshold=102.51499938964844
node_12: feature_name=ENSG00000087077	feature_id[71].value > threshold=35.76500129699707
node_16: feature_name=ENSG00000007968	feature_id[68].value > threshold=20.079999923706055
node_18: feature_name=ENSG00000167513	feature_id[73].value > threshold=23.320000648498535
Class: S	
Rules_71	passed counts:1
node_0: feature_name=ENSG00000170312	feature_id[0].value <= threshold=120.56500244140625
node_1: feature_name=ENSG00000197061	feature_id[1].value <= threshold=794.760009765625
node_2: feature_name=ENSG00000145425	feature_id[46].value <= threshold=4353.8848876953125
node_3: feature_name=ENSG00000127948	feature_id[39].value > threshold=4.385000109672546
node_9: feature_name=ENSG00000150991	feature_id[6].value > threshold=72.85999965667725
node_11: feature_name=ENSG00000014138	feature_id[17].value <= threshold=102.51499938964844

node_12: feature_name=ENSG00000087077	feature_id[71].value > threshold=35.76500129699707
node_16: feature_name=ENSG00000007968	feature_id[68].value > threshold=20.079999923706055
node_18: feature_name=ENSG00000167513	feature_id[73].value <= threshold=23.320000648498535
Class: G1	
Rules_72	passed counts:1
node_0: feature_name=ENSG00000170312	feature_id[0].value <= threshold=120.56500244140625
node_1: feature_name=ENSG00000197061	feature_id[1].value <= threshold=794.760009765625
node_2: feature_name=ENSG00000145425	feature_id[46].value <= threshold=4353.8848876953125
node_3: feature_name=ENSG00000127948	feature_id[39].value > threshold=4.385000109672546
node_9: feature_name=ENSG00000150991	feature_id[6].value > threshold=72.85999965667725
node_11: feature_name=ENSG00000014138	feature_id[17].value <= threshold=102.51499938964844
node_12: feature_name=ENSG00000087077	feature_id[71].value <= threshold=35.76500129699707
node_13: feature_name=ENSG00000131747	feature_id[19].value > threshold=50.72500038146973
Class: G1	
Rules_73	passed counts:1
node_0: feature_name=ENSG00000170312	feature_id[0].value <= threshold=120.56500244140625
node_1: feature_name=ENSG00000197061	feature_id[1].value <= threshold=794.760009765625
node_2: feature_name=ENSG00000145425	feature_id[46].value <= threshold=4353.8848876953125
node_3: feature_name=ENSG00000127948	feature_id[39].value > threshold=4.385000109672546
node_9: feature_name=ENSG00000150991	feature_id[6].value > threshold=72.85999965667725
node_11: feature_name=ENSG00000014138	feature_id[17].value <= threshold=102.51499938964844
node_12: feature_name=ENSG00000087077	feature_id[71].value <= threshold=35.76500129699707
node_13: feature_name=ENSG00000131747	feature_id[19].value <= threshold=50.72500038146973

Class: G2M	
Rules_74	passed counts:1
node_0: feature_name=ENSG00000170312	feature_id[0].value <= threshold=120.56500244140625
node_1: feature_name=ENSG00000197061	feature_id[1].value <= threshold=794.760009765625
node_2: feature_name=ENSG00000145425	feature_id[46].value <= threshold=4353.8848876953125
node_3: feature_name=ENSG00000127948	feature_id[39].value > threshold=4.385000109672546
node_9: feature_name=ENSG00000150991	feature_id[6].value <= threshold=72.85999965667725
Class: S	
Rules_75	passed counts:1
node_0: feature_name=ENSG00000170312	feature_id[0].value <= threshold=120.56500244140625
node_1: feature_name=ENSG00000197061	feature_id[1].value <= threshold=794.760009765625
node_2: feature_name=ENSG00000145425	feature_id[46].value <= threshold=4353.8848876953125
node_3: feature_name=ENSG00000127948	feature_id[39].value <= threshold=4.385000109672546
node_4: feature_name=ENSG00000012048	feature_id[12].value <= threshold=37.64999961853027
node_5: feature_name=ENSG00000124172	feature_id[95].value > threshold=544.1550140380859
Class: S	
